# Electronic cigarettes for smoking cessation

**DOI:** 10.1002/14651858.CD010216.pub10

**Published:** 2025-11-10

**Authors:** Nicola Lindson, Jonathan Livingstone-Banks, Ailsa R Butler, Hayden McRobbie, Christopher R Bullen, Peter Hajek, Angela Difeng Wu, Rachna Begh, Annika Theodoulou, Caitlin Notley, Nancy A Rigotti, Tari Turner, Thomas Fanshawe, Jamie Hartmann-Boyce

**Affiliations:** Nuffield Department of Primary Care Health SciencesUniversity of OxfordOxfordUK; Wolfson Institute of Population HealthQueen Mary University of LondonLondonUK; National Drug and Alcohol Research CentreUniversity of New South WalesSydneyAustralia; General Practice and Primary Care, School of Population HealthUniversity of AucklandAucklandNew Zealand; Wolfson Institute of Population HealthBarts & The London School of Medicine and Dentistry, Queen Mary University of LondonLondonUK; Norwich Medical SchoolUniversity of East AngliaNorwichUK; Tobacco Research and Treatment Center, Department of MedicineMassachusetts General Hospital and Harvard Medical SchoolBostonMassachusettsUSA; Cochrane AustraliaSchool of Public Health & Preventive Medicine, Monash UniversityMelbourneAustralia; Department of Health Promotion and PolicyUniversity of MassachusettsAmherstMAUSA

## Abstract

**Rationale:**

Electronic cigarettes (EC) are handheld electronic vaping devices that produce an aerosol by heating a liquid. People who smoke, healthcare providers, and regulators want to know if EC can help people quit smoking, and if they are safe to use for this purpose. This review update was conducted as part of a living systematic review.

**Objectives:**

To examine the safety, tolerability, and effectiveness of EC for helping people who smoke tobacco achieve long‐term smoking abstinence, in comparison to non‐nicotine EC, other smoking cessation treatments, and no treatment.

**Search methods:**

We searched the Cochrane Central Register of Controlled Trials (CENTRAL), MEDLINE, Embase, and PsycINFO to 1 March 2025, reference‐checked, and contacted study authors.

**Eligibility criteria:**

We included trials randomising people who smoked to an EC or control condition. We also included uncontrolled intervention studies where all participants received an EC intervention. Studies had to measure an eligible outcome.

**Outcomes:**

Critical outcomes were abstinence from smoking after at least six months, adverse events (AEs), and serious adverse events (SAEs). Important outcomes were biomarkers, toxicants/carcinogens, and longer‐term EC use.

**Risk of bias:**

We used the RoB 1 tool to assess risk of bias for each study and GRADE to assess evidence certainty.

**Synthesis methods:**

We followed standard Cochrane methods for screening and data extraction. Where appropriate, we pooled data using random‐effects models to calculate risk ratios (RRs) with 95% confidence intervals (CIs) for dichotomous outcomes. For continuous outcomes, we calculated mean differences with 95% CIs.

**Included studies:**

We included 104 completed studies (14 new to this update), representing 30,366 participants, of which 61 were randomised controlled trials (RCTs). We rated 11 included studies as being at low risk of bias, 70 at high risk (including all non‐randomised studies), and the remainder at unclear risk overall.

**Synthesis of results:**

Nicotine EC result in increased quit rates compared to nicotine replacement therapy (NRT) (high‐certainty evidence) (RR 1.55, 95% CI 1.28 to 1.88; I² = 0%; 9 studies, 2703 participants). In absolute terms, this might translate to an additional three quitters per 100 (95% CI 2 to 5 more). The rate of occurrence of AEs is probably similar between groups (moderate‐certainty evidence (limited by imprecision)) (RR 1.00, 95% CI 0.73 to 1.37; I² = 58%; 7 studies, 2241 participants). SAEs were rare, and there is insufficient evidence to determine whether rates differ between groups due to very serious imprecision (RR 1.22, 95% CI 0.73 to 2.03; I² = 30%; 8 studies, 2950 participants; low‐certainty evidence).

Nicotine EC probably result in increased quit rates compared to non‐nicotine EC (moderate‐certainty evidence, limited by imprecision) (RR 1.34, 95% CI 1.06 to 1.70; I² = 0%; 7 studies, 1918 participants). In absolute terms, this might lead to an additional two quitters per 100 (95% CI 0 to 4 more). There is probably little to no difference in the rate of AEs between these groups (moderate‐certainty evidence) (RR 1.01, 95% CI 0.95 to 1.08; I² = 0%; 5 studies, 840 participants). There is insufficient evidence to determine whether rates of SAEs differ between groups, due to very serious imprecision (RR 0.98, 95% CI 0.55 to 1.73; I² = 0%; 10 studies, 1717 participants; low‐certainty evidence).

Compared to behavioural support only or no support, quit rates may be higher for participants randomised to nicotine EC (low‐certainty evidence due to risk of bias) (RR 1.78, 95% CI 1.42 to 2.25; I² = 13%; 11 studies, 6819 participants). In absolute terms, this represents an additional three quitters per 100 (95% CI 2 to 5 more). There was some evidence that (non‐serious) AEs may be more common in people randomised to nicotine EC (RR 1.22, 95% CI 0.96 to 1.55; I² = 66%; 8 studies, 2485 participants; very low‐certainty evidence) but the evidence is uncertain and, again, there was insufficient evidence to determine whether rates of SAEs differed between groups (RR 0.93, 95% CI 0.67 to 1.29; I² = 0%; 15 studies, 4716 participants; very low‐certainty evidence).

Data from non‐randomised studies were consistent with RCT data. The most commonly reported AEs were throat/mouth irritation, headache, cough, and nausea, which tended to dissipate with continued EC use. Very few studies reported data on other outcomes or comparisons; hence, evidence for these is limited, with CIs often encompassing both clinically significant harm and benefit.

**Authors' conclusions:**

There is high‐certainty evidence that nicotine EC increase quit rates compared to NRT, and moderate‐certainty evidence that they probably increase quit rates compared to EC without nicotine. Evidence comparing nicotine EC with behavioural support or no support also suggests benefit, but is less certain due to risk of bias inherent in the study designs. CIs were, for the most part, wide for data on AEs, SAEs, and other safety markers, with no evidence of a difference in AEs between nicotine and non‐nicotine EC nor between nicotine EC and NRT, but low‐certainty evidence for increased AEs compared with behavioural support/no support. Overall incidence of SAEs was low across all study arms. We did not detect evidence of serious harm from nicotine EC, but longer, larger trials are needed to fully evaluate safety. Included studies tested regulated nicotine‐containing EC; illicit products and/or products containing other active substances (e.g. tetrahydrocannabinol (THC)) may have different harm profiles.

The main limitation of the evidence base remains imprecision for some comparisons and for safety outcomes due to the relatively small number of RCTs contributing, often with low event rates. Further RCTs are underway. To ensure the review continues to provide up‐to‐date information to decision‐makers, this is a living systematic review. We run and screen searches monthly, with the review updated when relevant new evidence becomes available. Please refer to the *Cochrane Database of Systematic Reviews* for the review's current status.

**Funding:**

Cancer Research UK (PICCTR‐2024/100012).

**Registration:**

Original 2012 protocol available via DOI: 10.1002/14651858.CD010216. Updated 2023 protocol available via DOI 10.17605/OSF.IO/ZWGSK (https://osf.io/ZWGSK/). 2025 updates to protocol available via DOI: 10.17605/OSF.IO/59M4U (https://osf.io/59M4U/) and DOI: 10.17605/OSF.IO/UPGJC (https://osf.io/UPGJC/).

## Plain language summary

**Can electronic cigarettes help people stop smoking, and do they have any unwanted effects when used for this purpose?**

**Key messages**

· Nicotine e‐cigarettes can help people to stop smoking for at least six months. Evidence shows they work better than nicotine replacement therapy, and probably better than e‐cigarettes without nicotine.

· They may work better than no support, or behavioural support alone, and may not be associated with serious unwanted effects.

· However, we still need more evidence, particularly about the effects of newer types of e‐cigarettes that have better nicotine delivery than older types, as better nicotine delivery might help more people quit smoking.

**What are electronic cigarettes?**

Electronic cigarettes (e‐cigarettes) or vapes are handheld devices that work by heating a liquid that usually contains nicotine and flavourings. E‐cigarettes allow users to inhale nicotine in a vapour rather than smoke. Because they do not burn tobacco, regulated e‐cigarettes do not expose users to the same levels of chemicals that can cause diseases in people who smoke conventional cigarettes.

Using an e‐cigarette is commonly known as 'vaping'. Many people use e‐cigarettes to help them to stop smoking tobacco. Here we focus primarily on e‐cigarettes containing nicotine.

**Why did we do this Cochrane review?**

Stopping smoking lowers the risk of many diseases. Many people find it difficult to stop smoking. We wanted to find out if using e‐cigarettes could help people to stop smoking, and if people using them for this purpose experience any unwanted effects.

**What did we do?**

We searched for studies that looked at the use of e‐cigarettes for stopping smoking.

We looked for randomised controlled trials, in which the treatments people received were decided at random. This type of study usually gives the most reliable evidence about treatment effects. We also looked for studies in which everyone received e‐cigarettes, and studies that gave e‐cigarettes to people who smoked and monitored their health even if there was no randomisation, so we could learn about their health effects.

We were interested in:

· how many people stopped smoking for at least six months; and

· how many people had unwanted effects, reported after at least one week of use.

**Search date**

We included evidence published up to 1 March 2025.

**What we found**

We found 104 studies including 30,366 adults who smoked. Studies compared nicotine e‐cigarettes with:

· nicotine replacement therapy (e.g. patches or gum);

· varenicline (a medicine to help people stop smoking);

· e‐cigarettes without nicotine;

· heated tobacco (products that heat tobacco to a high enough temperature to release vapour, without burning it or producing smoke; these differ from e‐cigarettes because they heat tobacco leaf/sheet);

· oral nicotine pouches (pouches that contain no tobacco but release nicotine when kept in the mouth);

· other types of nicotine‐containing e‐cigarettes (e.g. pod devices, newer devices);

· behavioural support (e.g. advice or counselling); or

· no support for stopping smoking.

Most studies took place in the USA (48 studies) and the UK (21).

**What are the results of our review?**

People are more likely to stop smoking for at least six months using nicotine e‐cigarettes than using nicotine replacement therapy (9 studies, 2703 people) or e‐cigarettes without nicotine (7 studies, 1918 people).

Nicotine e‐cigarettes may help more people to stop smoking than either no support or behavioural support only (11 studies, 6819 people).

For every 100 people using nicotine e‐cigarettes to stop smoking, 8 to 11 might successfully stop, compared with only 6 of 100 people using nicotine‐replacement therapy, 6 of 100 using e‐cigarettes without nicotine, or 4 of 100 people having no support or behavioural support only.

We are uncertain if there is a difference between how many unwanted effects occur using nicotine e‐cigarettes compared with nicotine replacement therapy, no support or behavioural support only. There was some evidence that non‐serious unwanted effects were more common in groups receiving nicotine e‐cigarettes compared to no support or only behavioural support, but the evidence is uncertain. Low numbers of unwanted effects, including serious unwanted effects, were reported in studies comparing nicotine e‐cigarettes to nicotine replacement therapy. There is probably no difference in how many non‐serious unwanted effects occur in people using nicotine e‐cigarettes compared to e‐cigarettes without nicotine.

The unwanted effects reported most often with nicotine e‐cigarettes were throat or mouth irritation, headache, cough, and nausea. These appear similar to what people experience when using NRT. Unwanted effects were reduced over time as people continued using nicotine e‐cigarettes.

**How reliable are these results?**

We found evidence that nicotine e‐cigarettes help more people to stop smoking than nicotine replacement therapy. Nicotine e‐cigarettes probably help more people to stop smoking than e‐cigarettes without nicotine, but more studies are still needed to confirm this.

Studies comparing nicotine e‐cigarettes with behavioural or no support also showed higher quit rates in people using nicotine e‐cigarettes, but provide less certain data because of issues with study design.

Most of our results for the unwanted effects could change when more evidence becomes available.

## Summary of findings

**Summary of findings 1:** Nicotine EC compared to NRT for smoking cessation

**Nicotine EC compared to NRT for smoking cessation**						
**Patient or population:** people who smoke cigarettes, aged 18 or older **Setting:** various settings **Intervention:** nicotine EC **Comparison:** NRT						
Outcomes	Anticipated absolute effects^*^ (95% CI)		Relative effect (95% CI)	№ of participants (studies)	Certainty of the evidence (GRADE)	Comments
	Events with NRT	Events with Nicotine EC				
Smoking cessation at 6+ months Preferentially assessed with biochemical validation	Study population		RR 1.51 (1.25 to 1.82)	2703 (9 RCTs)	⊕⊕⊕⊕ HIGH	‐
	6 per 100	9 per 100 (8 to 11)				
Adverse events at 4 weeks to 6 to 9 months Assessed by self‐report	Study population		RR 1.00 (0.73 to 1.37)	2241 (7 RCTs)	⊕⊕⊕⊝ MODERATE^a^	‐
	31 per 100	31 per 100 (23 to 42)				
Serious adverse events at 4 weeks to 1 year Assessed via self‐report and medical records	Study population		RR 1.22 (0.73 to 2.03)	2950 (8 RCTs)	⊕⊕⊝⊝ LOW^b^	2 studies reported no events; effect estimate based on the 5 studies in which events were reported
	7 per 100	9 per 100 (5 to 14)				
*The estimated number of events in the intervention group (and its 95% confidence interval) is based on the assumed number of events in the comparison group and the **relative effect** of the intervention (and its 95% CI). For cessation, the assumed number of events in the control group is based on assumed quit rates for NRT assuming receipt of limited behavioural stop‐smoking support (as per [1]). The assumed risk for adverse events and serious adverse events is a weighted mean average of quit rates across control groups in contributing studies. **CI:** confidence interval; **EC**: electronic cigarette; **NRT**: nicotine replacement therapy; **RCT:** randomised controlled trial; **RR:** risk ratio						
**GRADE Working Group grades of evidence** **High certainty:** We are very confident that the true effect lies close to that of the estimate of the effect. **Moderate certainty:** We are moderately confident in the effect estimate: the true effect is likely to be close to the estimate of the effect, but there is a possibility that it is substantially different. **Low certainty:** Our confidence in the effect estimate is limited: the true effect may be substantially different from the estimate of the effect. **Very low certainty:** We have very little confidence in the effect estimate: the true effect is likely to be substantially different from the estimate of effect.						

^a^Downgraded one level due to imprecision; CIs consistent with benefit and harm. ^b^Downgraded two levels due to imprecision; fewer than 300 events and CIs encompass clinically important harm and clinically important benefit.

**Summary of findings 2:** Nicotine EC compared to non‐nicotine EC for smoking cessation

**Nicotine EC compared to non‐nicotine EC for smoking cessation**						
**Patient or population:** people who smoke cigarettes, aged 18 or older **Setting:** various settings **Intervention:** nicotine EC **Comparison:** non‐nicotine EC						
Outcomes	Anticipated absolute effects^*^ (95% CI)		Relative effect (95% CI)	№ of participants (studies)	Certainty of the evidence (GRADE)	Comments
	Events with non‐nicotine EC	Events with nicotine EC				
Smoking cessation at 6+ months Preferentially assessed with biochemical validation	Study population		RR 1.34 (1.06 to 1.70)	1918 (7 RCTs)	⊕⊕⊕⊝ MODERATE^a,b^	‐
	6 per 100	8 per 100 (6 to 10)				
Adverse events at 1 week to 6 months Assessed via self‐report	Study population		RR 1.01 (0.95 to 1.08)	840 (5 RCTs)	⊕⊕⊕⊝ MODERATE^b^	‐
	46 per 100	46 per 100 (44 to 50)				
Serious adverse events at 1 week to 1 year Assessed via self‐report and medical records	Study population		RR 0.98 (0.55 to 1.73)	1717 (10 RCTs)	⊕⊕⊝⊝ LOW^c^	5 studies reported no events; effect estimate based on the 5 studies in which events were reported
	3 per 100	3 per 100 (2 to 5)				
*The estimated number of events in the intervention group (and its 95% confidence interval) is based on the assumed number of events in the comparison group and the **relative effect** of the intervention (and its 95% CI). For cessation, the assumed number of events in the control group is based on assumed quit rates for NRT assuming receipt of limited behavioural stop‐smoking support (as per [1]). The assumed risk for adverse events and serious adverse events is a weighted mean average of quit rates across control groups in contributing studies. **CI:** confidence interval; **EC**: electronic cigarette; **RCT:** randomised controlled trial; **RR:** risk ratio						
**GRADE Working Group grades of evidence** **High certainty:** We are very confident that the true effect lies close to that of the estimate of the effect **Moderate certainty:** We are moderately confident in the effect estimate: The true effect is likely to be close to the estimate of the effect, but there is a possibility that it is substantially different **Low certainty:** Our confidence in the effect estimate is limited: The true effect may be substantially different from the estimate of the effect **Very low certainty:** We have very little confidence in the effect estimate: The true effect is likely to be substantially different from the estimate of effect						

^a^Not downgraded for risk of bias. One of seven studies considered high risk of bias; removing this study increased the direction of the effect in favour of the intervention. ^b^Downgraded one level due to imprecision: confidence intervals encompass both harm and no difference. ^c^Downgraded two levels due to imprecision: confidence intervals encompass clinically significant harm as well as clinically significant benefit; < 300 events overall.

**Summary of findings 3:** Nicotine EC compared to behavioural support only/no support for smoking cessation

**Nicotine EC compared to behavioural support only/no support for smoking cessation**						
**Patient or population:** people who smoke cigarettes, aged 18 or older **Setting:** various settings **Intervention:** nicotine EC **Comparison:** behavioural support only/no support						
Outcomes	Anticipated absolute effects^*^ (95% CI)		Relative effect (95% CI)	№ of participants (studies)	Certainty of the evidence (GRADE)	Comments
	Events with behavioural support only/no support	Events with nicotine EC				
Smoking cessation at 6+ months Preferentially assessed using biochemical validation	Study population		RR 1.78 (1.42 to 2.25)	6819 (11 RCTs)	⊕⊕⊝⊝ LOW^a^	‐
	4 per 100	7 per 100 (6 to 9)				
Adverse events at 12 weeks to 6 months Assessed via self‐report	Study population		RR 1.22 (0.96 to 1.55)	2485 (8 RCTs)	⊕⊝⊝⊝ VERY LOW^a,b,c^	‐
	50 per 100	61 per 100 (48 to 78)				
Serious adverse events at 4 weeks to 8 months Assessed via self‐report and medical records	Study population		RR 0.93 (0.67 to 1.29)	4716 (15 RCTs)	⊕⊝⊝⊝ VERY LOW^a,d^	8 of the 15 studies reported no SAEs; MA is based on pooled results from 7 studies.
	4 per 100	4 per 100 (3 to 5)				
*The estimated number of events in the intervention group (and its 95% confidence interval) is based on the assumed number of events in the comparison group and the **relative effect** of the intervention (and its 95% CI). For cessation, the assumed number of events in the control group is based on assumed quit rates assuming receipt of limited behavioural stop‐smoking support (as per [1]). The assumed risk for adverse events and serious adverse events is a weighted mean average of quit rates across control groups in contributing studies. **CI:** confidence interval; **EC**: electronic cigarette; **MA:** meta‐analysis; **RCT:** randomised controlled trial; **RR:** risk ratio						
**GRADE Working Group grades of evidence** **High certainty:** We are very confident that the true effect lies close to that of the estimate of the effect **Moderate certainty:** We are moderately confident in the effect estimate: The true effect is likely to be close to the estimate of the effect, but there is a possibility that it is substantially different **Low certainty:** Our confidence in the effect estimate is limited: The true effect may be substantially different from the estimate of the effect **Very low certainty:** We have very little confidence in the effect estimate: The true effect is likely to be substantially different from the estimate of effect						

^a^Downgraded two levels due to risk of bias. Due to lack of blinding and differential support between arms, this domain was judged to be at high risk of bias. ^b^Not downgraded for inconsistency: despite moderate statistical heterogeneity (I^2^ = 66%), this was driven by magnitude rather than direction of effect. ^c^Downgraded one level due to imprecision. Confidence intervals incorporated no clinically significant difference and clinically significant harm. ^d^Downgraded two levels due to imprecision. Fewer than 300 events and confidence intervals incorporated clinically significant benefit and clinically significant harm.

## Background

Throughout this review, we discuss (1) combustible cigarettes and (2) electronic cigarettes. Electronic cigarettes are hand‐held and produce an aerosol for inhalation, formed by heating a liquid using a battery‐powered heating coil. In this review, all mention of smoking, smoking cessation, cigarette use, smoke intake, etc. concerns combustible tobacco cigarettes. When the text concerns electronic cigarettes, we use the abbreviation 'EC'. EC users are sometimes described as 'vapers', and EC use as 'vaping'. We refer to EC that do not contain nicotine as non‐nicotine EC; these can also be conceptualised as placebo EC, but we are using the term non‐nicotine EC, as they can be conceptualised as an intervention in themselves. This review does not address the use of vaping devices to inhale substances other than nicotine, such as cannabis.

### Description of the condition

Stopping smoking tobacco is associated with large health benefits. Despite most people who smoke wanting to quit, many find it difficult to succeed in the long term. Almost half who try to quit without support will not manage to stop for even a week, and fewer than five per cent remain abstinent one year after quitting [2].

Behavioural support and medications such as nicotine patches or gum increase the chances of quitting through providing nicotine to help alleviate withdrawal symptoms, but even with such support, long‐term quit rates remain low [3, 4, 5, 6, 7, 8]. One of the limitations of traditional nicotine replacement therapy (NRT) is that, apart from providing nicotine more slowly and at lower levels than smoking, none adequately addresses the sensory, behavioural, and/or social aspects of smoking that people who have smoked miss when they stop (e.g. holding a cigarette in their hands, taking a puff, enjoyment of smoking, feeling part of a group). EC may offer a way to overcome these limitations [9], and have become a popular consumer choice for smoking cessation support where regulations allow [10].

There is no doubt that people can become dependent on tobacco, and many find it difficult to stop smoking, primarily because of nicotine and its actions on the brain's reward system [11]. However, developing dependence on tobacco smoking is a complex biopsychosocial process [12, 13]. Other tobacco chemicals, such as acetaldehyde and monoamine oxidase (MAO) inhibitors, seem to potentiate the effects of nicotine [13]. In addition, sensory and behavioural cues provide additional reinforcement of smoking behaviour [14, 15] and may over time become almost as rewarding as nicotine. There are several lines of evidence to support this. Firstly, people who smoke appear to have a preference for cigarette smoke compared to other forms of nicotine delivery. This is partly related to the speed of nicotine delivery through smoke inhalation. However, even when nicotine is administered intravenously, it does not provide the same level of satisfaction or reward as smoking [15, 16]. Secondly, the local sensory effects of smoking (e.g. the ‘scratch’ in the back of the throat) may be important for enjoyment and reward. Numbing the sensations of cigarette smoke by anaesthetising the upper and lower respiratory tract leads to less enjoyment of smoking [17]. Conversely, products that mimic the sensory effects of smoking on the mouth and throat (such as citric acid, black pepper, and ascorbic acid) reduce craving and some withdrawal symptoms, at least in the short term [18, 19, 20]. Thirdly, very low nicotine content cigarettes (VLNC), which have less nicotine (e.g. 0.08 mg) than the 1 mg in regular cigarettes, and so have negligible or no central effects, have also been investigated for their role in aiding smoking cessation [21]. Despite delivering low levels of nicotine, VLNC are satisfying over the initial few days of abstinence from nicotine [15, 22, 23, 24]. They also reduce tobacco withdrawal symptoms, including urges to smoke and low mood [15, 25, 26, 27, 28], and have been shown to improve long‐term continuous abstinence rates [29]. Social aspects of smoking, such as feeling part of a like‐minded group, or including smoking behaviour as part of one's social identity, are also elements of cigarette smoking that some people who smoke report to be drivers of cigarette use [30].

Considering the other factors that contribute to tobacco dependence, there is interest in developing smoking cessation products that not only help relieve the unpleasant effects of nicotine withdrawal, but that also act as effective substitutes for smoking behaviour and the rituals and sensations that accompany smoking, without the health risks associated with the inhalation of tobacco smoke. The only pharmaceutical treatment with some of these characteristics is the nicotine inhalator. However, these do not have greater cessation efficacy than other NRT products [1, 31]. This may, in part, be due to the considerable effort (e.g. 20 minutes of continuous puffing) needed to provide nicotine blood concentrations consistent with other NRT products [32]. Adherence to correct use of the inhalator is low compared to other types of NRT [31]. It is therefore possible that any advantage of sensorimotor replacement is diminished by low nicotine delivery and limited similarities between inhalator use and the sensations of smoking [33].

### Description of the intervention and how it might work

The liquid used in EC, usually comprising propylene glycol and glycerol, with or without nicotine and flavours, is stored in disposable or refillable cartridges or a reservoir or 'pod'. The commonly used term for this aerosol is vapour, which we use throughout this review. EC are marketed as consumer products. Although routes are in place for licencing them as medicine or medical devices in some areas, no country yet has a licenced medicinal EC.

EC provide sensations similar to smoking a cigarette. The vapour looks like tobacco smoke, but is only visible when the user exhales after drawing on the mouthpiece, not when the device is being held. In qualitative studies, users report a sense of shared identity with other users, similar to tobacco‐smoking identity, and also report pleasure and enjoyment of use, suggesting that EC may be viewed less as medical cessation aids but rather as acceptable alternatives to tobacco smoking [30, 34].

There are many different brands and models of EC available. Variation exists both in the device ('product') and consumable (liquid). There is a wide variation in the composition of EC liquids (e.g. nicotine content; flavours) [35, 36], with some users choosing to mix their own liquids [37]. Initial studies showed that early models of EC delivered very low amounts of nicotine to naïve users [33, 38, 39]. Later studies, that have measured nicotine pharmacokinetics in both experienced and naïve EC users, have found that some EC users can achieve blood nicotine levels similar to those achieved with smoking, albeit more slowly, and that their ability to do so often improves over time [40, 41, 42, 43, 44].

Early in their development, EC were designed to look like cigarettes and used disposable cartridges. These models were often called 'cig‐a‐likes'. The nicotine delivery from these products was low [45]. The later refillable, or 'tank', products have a larger battery and a transparent container that users fill with a liquid of their choice, and usually provide faster and more efficient nicotine delivery, allowing a wider choice of flavours and nicotine concentrations. They have more typically been used by experienced vapers, who reportedly managed to switch to vaping completely [46, 47, 48, 49, 50]. More recently, smaller 'pod' devices that use nicotine salt solutions have become available. This nicotine formulation reduces irritant effects and allows the delivery of higher nicotine levels that closely mimic the pharmacokinetic profile of nicotine delivery from cigarettes, despite the low battery power of the devices [51]. In qualitative studies, pod devices have been highly rated by users in terms of satisfaction, usability (simple to use), affordability, and availability [52]. The nicotine salts used in pods allow for high nicotine delivery; this may increase the likelihood that adults who smoke are able to transition completely from conventional cigarettes [53]. Average nicotine concentrations in EC sold in the United States increased overall during 2013‐2018, for all flavour categories, and for rechargeable EC [54]. The EU Tobacco Products Directive [55] does not allow sales of liquids with nicotine content higher than 20 mg/mL, and so the US version of the Juul pod device (59 mg/nl nicotine) is not legally available within the EU [56, 57]. Most recently, there has been rapid growth in the use of disposable and single‐use devices [58, 59]. These are available in a range of attractive flavours, generally have a high nicotine content, are low cost, and have a closed system that is designed to be disposed of following use. Disposable EC are receiving increased regulatory attention, and have recently been banned in the UK [60].

Different device types may differ significantly in their efficacy in helping people who smoke to quit, as they differ in delivery of nicotine. Nicotine itself, when delivered through mechanisms and doses similar to that delivered in traditional NRT, is not considered harmful [1]. The safety profile of the different types of nicotine EC may be similar as they use the same constituents, although within the generic range of EC types there is some evidence to suggest EC providing less nicotine may pose higher risks. This is because low‐nicotine delivery devices need to be puffed with higher intensity to provide users with the nicotine levels they seek, and more intensive puffing is accompanied by increased inhalation of potential toxicants [61, 62, 63].

There is no one agreed classification system for EC devices, and product development has moved so quickly that the definitions used within trials of the devices tested may no longer necessarily be fit for purpose. In this review, the definitions used are based on those drawn from the included trials. We currently label different types of EC as 'cartridges' for devices with disposable cartridges and – typically, but not always – low nicotine delivery (e.g. cig‐a‐likes); refillable EC for devices that people fill with their own choice of liquids; pods for the small devices with disposable pods that commonly use nicotine salts; and disposable for closed system devices designed to be disposed of after use.

### Why it is important to do this review

Regulatory approaches being used for EC currently vary widely, from no regulation to partial and complete bans [64]. Within the USA, for example, the Food and Drug Administration (FDA) has classified EC as tobacco products and laws include prohibition of EC use indoors, a requirement for retailers to have a licence to sell, and prohibition of sales to minors. Laws prohibiting sales to minors apply nationwide, but other laws vary by state [65]. The European Union includes EC in their Tobacco Products Directive, except where therapeutic claims are made or in instances where they contain over 20 mg/nl of nicotine [55].

Categorical statements about the toxicity of EC are not possible because of the large number of devices and liquids available and the frequent addition of new products to the market. In 2019, cases of severe lung injury associated with EC use were reported in the USA and, by February 2020, there were around 2800 hospitalised cases and 68 deaths [66]. This illness, which was termed E‐cigarette or Vaping‐Associated Lung Injury (EVALI), caused concern throughout the world [67] and a negative change in people's perception of the risks of EC use compared to smoking [68]. These cases were somewhat at odds with data from trials and cohort studies, and it was later found that these injuries were related to use of tetrahydrocannabinol (THC)‐containing products adulterated with vitamin E acetate [69, 70].

Amongst those brands of nicotine EC that have been tested, levels of toxicants have been found to be substantially lower than in cigarettes [64, 71]. Long‐term effects beyond 12 months are unclear, although based on what is known about liquid and vapour constituents and patterns of use, a report from the UK's Royal College of Physicians has concluded that using an EC is likely to be considerably safer than smoking [72]. The US National Academies of Sciences, Engineering, and Medicine (NASEM) concluded that EC are likely to be far less harmful than continuing to smoke cigarettes, with the caveat that the long‐term health effects of EC use are not yet known [73].

Despite general acknowledgement that EC use exposes the user to fewer toxicants and at lower levels than smoking cigarettes [64, 73, 74, 75], in some countries and settings there remains hesitancy in making these products available to people who smoke as a harm reduction tool or smoking cessation aid (e.g. [76]). Concerns include the issue that the long‐term effects of EC use on health are not yet known, the possible harms of second‐hand EC vapour inhalation, the lack of quality control measures, and that EC may undermine smoke‐free legislation if used in smoke‐free spaces [64]. Of concern is also the involvement of the tobacco industry and that EC may be a gateway to smoking initiation or nicotine dependence amongst nicotine‐naïve users, or may prolong continued dual use of tobacco amongst people who smoke cigarettes [64], and some research investigates this [77]. A report from the US Preventive Services Taskforce concluded "that the current evidence is insufficient to assess the balance of benefits and harms of electronic cigarettes (e‐cigarettes) for tobacco cessation in adults" [78]. However, others suggest that potential benefits outweigh potential disadvantages [49, 64, 71, 73, 74, 75].

People who smoke, healthcare providers, and regulators are interested in knowing if EC can help people to quit and if it is safe to use them to do so. In particular, healthcare providers have an urgent need to know what they should recommend to people to help them to stop smoking. The largest health gains are achieved from stopping smoking completely, as opposed to reducing cigarette consumption and, as such, this review focusses on the effectiveness of EC in aiding complete smoking cessation.

This review was first published in 2014, and updated in 2016, 2020, 2021, 2022, 2024 and 2025. We published an update to the protocol in 2023 (see [79]) and in 2025 (https://osf.io/59m4u/).

Following publication of the 2020 update of this review, we are maintaining it as a living systematic review [80]. This means we are continually running searches and incorporating new evidence into the review. For more information about the living systematic review methods being used, see Supplementary material 8. A living systematic review approach is appropriate for this review for three reasons. Firstly, the review addresses an important public health issue: the role of EC in enabling people who smoke to stop smoking, with the potential for substantial ongoing individual and societal benefits, depending on the extent of effectiveness. Secondly, there remains uncertainty in the existing evidence; more studies are needed to confirm the degree of benefit for different comparisons and product types, and there is considerable uncertainty about adverse events and other markers of safety. Thirdly, we are aware of multiple ongoing trials that are likely to have an important impact on the conclusions of the review.

## Objectives

To examine the safety, tolerability, and effectiveness of using electronic cigarettes (EC) to help people who smoke tobacco achieve long‐term smoking abstinence, in comparison with other smoking cessation treatments, non‐nicotine EC, and no treatment.

## Methods

We followed the Methodological Expectations of Cochrane Intervention Reviews (MECIR) when conducting the review [81], and PRISMA 2020 for the reporting [82]. As this is a living review, methods are periodically updated between review updates to ensure that the review remains relevant and reliable. Differences in methods from the protocol and between review updates are documented in Supplementary material 10. Additional changes may be made to the methods for future updates, which will also be documented.

### Criteria for considering studies for this review

#### Types of studies

We include randomised controlled trials (RCTs) and randomised cross‐over trials in which people who smoke are randomised to EC or to a control condition. RCTs are the best available primary evidence to fulfil our objectives. We also include uncontrolled intervention studies in which all participants are given an EC intervention. These studies have the potential to provide additional information on harms and longer‐term use. In the next update of this review, in response to editorial feedback from Cochrane and because of the growth in the RCT evidence base, we will no longer include single‐arm studies where all participants receive EC (see September 2025 protocol update).

We include studies regardless of their publication status or language of publication.

#### Types of participants

Participants are people defined as currently smoking cigarettes at enrolment into the studies. Participants could be of any age, motivated or unmotivated to quit, and we include studies that recruited pregnant people.

Should a study meet all other criteria, but include only a subset of eligible participants (e.g. a study on people who currently smoke and people who formerly smoked), we would only include data on the subgroup of participants who met our inclusion criteria. If these data were not available, we would include the study if at least 80% of participants met our inclusion criteria and would test the exclusion of the study in a sensitivity analysis.

#### Types of interventions

Any type of EC or intervention intended to promote EC use for smoking cessation, including studies that do not measure smoking cessation but provide EC with the instruction that they be used as a complete substitute for cigarette use. EC may or may not contain nicotine.

##### Types of comparators

We compare nicotine EC with alternative smoking cessation aids, including NRT or no intervention, with EC without nicotine, and EC added to standard smoking cessation treatment (behavioural or pharmacological, or both) with standard treatment alone. We also compare different types of EC (refillable, cartridge, nicotine salt, free‐base), different nicotine doses, and different flavours.

### Outcome measures

#### Critical outcomes

- Cessation at the longest follow‐up point, at least six months from the start of the intervention, measured on an intention‐to‐treat basis using the strictest definition of abstinence, preferring biochemically validated results, where reported;
- Number of participants reporting any type of adverse event(s) at one week or longer (as defined by study authors);
- Number of participants reporting any type of serious adverse event(s) at one week or longer (as defined by study authors).

#### Important outcomes

Number of people still using the study product (EC or pharmacotherapy) at longest follow‐up (at least six months). The product could be that provided by the study, or could be the same product type but bought independently by the participant.

Changes in the following measures at longest follow‐up (one week or longer):

- Carbon monoxide (CO), measured through breath or blood;
- Blood pressure;
- Heart rate;
- Blood oxygen saturation;
- Lung function measures;
- Known toxicants/carcinogens, measured through blood, urine or saliva (toxicant names and abbreviations are listed in Supplementary material 9).

Studies had to set out to measure one of the critical or important outcomes above to be eligible for inclusion. If a study set out to measure an eligible outcome but did not measure and/or report results on this outcome, we would still include this study and flag its missing data in the results section.

We intended to include any measure of an association between withdrawal symptoms and smoking cessation at six months or longer, as long as withdrawal was measured using a validated scale designed explicitly to investigate smoking withdrawal or craving. We added this because British guidelines now specify that efforts should be made to provide EC in a way that will reduce symptoms of withdrawal in people who smoke [83]. However, no studies provided data on this.

### Search methods for identification of studies

#### Electronic searches

Searches are conducted monthly. This update includes results from searches conducted up to 1 March 2025:

- Cochrane Tobacco Addiction Group Specialized Register (CRS‐Web up to 1 February 2023);
- Cochrane Central Register of Controlled Trials (CENTRAL; 2025, Issue 2) via CRS‐Web;
- MEDLINE (OVID SP; 1 January 2004 to 1 March 2025);
- Embase (OVID SP; 1 January 2004 to 1 March 2025);
- PsycINFO (OVID SP; 1 January 2004 to 1 March 2025);
- ClinicalTrials.gov (via CENTRAL; 2025, Issue 2);
- WHO International Clinical Trials Registry Platform (ICTRP: www.who.int/ictrp/en/, via CENTRAL; 2025, Issue 2).

We did not search the Cochrane Tobacco Addiction Group Specialized Register beyond 1 February 2023 as it ceased to be maintained. At the time of the last search, the Register included the results of searches of MEDLINE (via OVID) to update 20221222; Embase (via OVID) to week 202251; and PsycINFO (via OVID) to update 20221219. See the Tobacco Addiction Group website for full search strategies and a list of other resources searched.

For the first version of the review, we also searched CINAHL (EBSCO Host) (2004 to July 2014). We did not search this database from 2016 onwards, as it did not contribute additional search results to the first version of the review. The search terms were broad and included 'e‐cig$' OR 'elect$ cigar$' OR 'electronic nicotine'. The search for the 2016 update added the terms 'vape' or 'vaper' or 'vapers' or 'vaping'. The 2020 searches added further terms, including the MESH heading 'Electronic Nicotine Delivery Systems' and terms to limit by study design. The current and previous search strategies are listed in Supplementary material 1. The search date parameters of the original searches were limited to 2004 to the present, as EC were not available before 2004.

As part of our monthly screening process, all new records related to each included study are incorporated into our records for that study. We searched for post‐publication amendments and examined any relevant retraction statements and errata for included studies (e.g. through PubMed and the Retraction Watch Database; retractionwatch.com/retraction‐watch‐database‐user‐guide/), as errata could reveal important limitations or even serious flaws in the included trials [84]. We are confident our search strategy will have caught any post‐publication amendments currently published, including expressions of concern, errata, corrigenda and retractions.

#### Searching other resources

We searched the reference lists of eligible studies found in the literature search and contacted authors of known trials and other published EC studies. We also searched for abstracts from the Society for Research on Nicotine and Tobacco (SRNT) Annual Meetings up to 1 March 2025.

### Data collection and analysis

#### Selection of studies

Two review authors (for this update from: ADW, ARB, CN, JHB, NL, AT, TT) independently pre‐screened all titles and abstracts obtained from the search, using a screening checklist, and then independently screened full‐text versions of the potentially relevant papers for inclusion. We resolved any disagreements by discussion or with a third review author (from authors named above).

#### Data extraction and management

One review author extracted data on study characteristics (ARB), whereas two review authors (for this update: ARB, ADW, AT, CN, RB) independently extracted outcome data, effect modifiers, and the information needed to make risk of bias judgements. We used a pre‐piloted data extraction form, and checked the form for inconsistencies. We resolved any disagreements by discussion or with a third review author (NL or JHB). We extracted data on the following:

- Author(s);
- Date and place of publication;
- Study dates;
- Study design;
- Inclusion and exclusion criteria;
- Setting;
- Summary of study participant characteristics;
- Summary of intervention and control conditions;
- Number of participants in each arm;
- Smoking cessation outcomes;
- Type of biochemical validation (if any);
- Adverse events (AEs), serious adverse events (SAEs), number of people still using the study product, and relevant biomarkers;
- Continued EC use or pharmaceutical intervention (PI) use at longest follow‐up;
- Data investigating the association between withdrawal and smoking cessation;
- Assessment of time points;
- Study funding source;
- Author declarations of interest;
- Risk of bias in the domains specified below;
- Additional comments.

We adopted a broad focus to detect a variety of adverse events.

There were no papers that required translation*;* should there be in the future, we would use online translation software in the first instance, and seek a translator to assist us where necessary.

For studies that received tobacco or EC industry funding, the study name is followed by an asterisk (*).

One review author (NL for this update) entered the data contributing to meta‐analysis into RevMan Web software for analysis [85], and another checked them (JHB for this update).

#### Risk of bias assessment in included studies

Two review authors (for this update: ARB, ADW, AT, CN, RB) independently assessed the risks of bias for each included study, using the Cochrane risk of bias tool (RoB 1) [86]. We resolved any disagreements by discussion or with a third review author (NL or JHB). This approach uses a domain‐based evaluation that addresses seven different areas: random sequence generation; allocation concealment; blinding of participants and providers; blinding of outcome assessment; incomplete outcome data; selective outcome reporting; and other potential sources of bias. We assigned a grade (low, high, or unclear) for risk of bias for each domain. We resolved disagreements by discussion or by consulting a third review author.

Specific considerations about judgements for individual domains in this review are outlined below:

- Random sequence generation/allocation concealment: We rated all non‐randomised studies as being at high risk in these domains.
- Blinding of participants and personnel: We did not evaluate this domain for non‐randomised studies, as we considered it not to be applicable. For randomised studies that did not use blinding, we considered studies to be at low risk in this domain if the intervention was compared to an active control of similar intensity, as we judged performance bias to be unlikely in this circumstance. If studies were unblinded and the comparator group was a minimal‐intervention control or of lower intensity than the intervention group, we considered the study to be at high risk of bias in this domain.
- Following the standard methods of the Cochrane Tobacco Addiction Group [87], we considered studies to be at low risk of detection bias (blinding of outcome assessment) if our primary outcome was objectively measured or if the intensity of the intervention was similar between groups, or both. For studies where cessation was measured, our judgement was based on whether cessation was biochemically verified. Where cessation was not measured, we judged this domain based on adverse or serious adverse events.
- Again, following the standard methods of the Cochrane Tobacco Addiction Group, we rated studies as being at high risk of attrition bias if loss to follow‐up was greater than 50% overall or if there was a difference in follow‐up rates of more than 20% between study arms.

We judged studies to be at high risk of bias overall if they were rated at high risk in at least one domain, and at low risk of bias overall if they were judged to be at low risk across all domains evaluated. We judged the remaining studies to be at unclear risk of bias overall.

#### Measures of treatment effect

We analysed dichotomous data by calculating the risk ratio (RR) with a 95% confidence interval (CI), for each outcome for each individual study. For example, for cessation, we calculated the RR as (number of events in intervention condition/intervention denominator)/(number of events in control condition/control denominator), using data at the longest follow‐up period reported.

We analysed continuous data (other measures of tobacco exposure) by comparing the difference between the mean change from baseline to follow‐up in the intervention and comparator groups, or by comparing absolute data at follow‐up where insufficient data were available on mean change. Outcomes are reported as mean differences (MD) with 95% CI.

For outcomes other than cessation, where data were reported at multiple time points, we used data at the longest follow‐up point at which EC were still being provided, or their use was encouraged.

#### Unit of analysis issues

In the case of trials with multiple arms, we did not combine data between arms, unless this was the way it was presented by study authors, or there was no evidence of difference between similar trial arms for the outcome of interest. We note in our analyses where this is the case.

For all but one study, the unit of assignment was the individual. Dawkins 2020 [88, 89] assigned the condition based on a homeless support service; this was a small pilot study with very few events and, hence, we judged clustering to have very little impact on our overall result. If larger cluster‐randomised trials are eligible in the future, we will assess whether the study authors have adjusted for this clustering, and whether this had an impact on the overall result. When clustering appears to have had little impact on the results, we will use unadjusted quit‐rate data; however, when clustering does appear to have an impact on results, we will adjust for this using the intraclass correlation (ICC).

For randomised cross‐over trials, we report results at the end of the first assignment period where available and where sufficiently long to meet our inclusion criteria for outcomes. All other outcomes from randomised cross‐over trials are reported narratively. We offer a narrative synthesis of data from non‐randomised studies and outcomes from comparative trials that are not reported with sufficient data for meta‐analysis, using effect direction plots as described in the *Cochrane Handbook for Systematic Reviews of Interventions,* where possible [90].

#### Dealing with missing data

For smoking cessation, we use a conservative approach, as is standard for the Cochrane Tobacco Addiction Group, treating participants with missing data as still smoking. We base the proportion of people affected by adverse events on the number of people available for follow‐up, and not the number randomised. For all other outcomes, we also use complete‐case data and do not attempt to impute missing values.

#### Reporting bias assessment

Reporting bias can be assessed using funnel plots, where 10 or more RCTs contribute to an outcome. Where studies were included in an analysis but did not contribute data to the pooled effect (as zero events were reported), these were not included in the count of included studies when deciding whether to generate funnel plots. Therefore, there were only two analyses with sufficient studies to support this approach.

#### Synthesis methods

We provide a narrative summary of the included studies. We have grouped studies by comparison type and outcome to carry out syntheses.

Where appropriate, we pooled data from RCTs in meta‐analyses. For dichotomous data, we used random‐effects Mantel‐Haenszel models to calculate the pooled RR with a 95% CI, in accord with the standard methods of the Cochrane Tobacco Addiction Group for cessation studies. For continuous outcomes, we calculated mean differences or standardised mean differences (as appropriate for studies using different measures for the same construct), using the inverse variance approach (also with a 95% CI). We calculated confidence intervals using the Hartung‐Knapp‐Sidik‐Jonkman method in analyses with at least three studies, and the heterogeneity was greater than zero. In analyses of two studies, or where the estimate of heterogeneity was zero, we used the Wald‐type method.

We assessed the clinical and methodological diversity between studies to guide our decision about whether data should be pooled. We were also guided by the degree of statistical heterogeneity, assessed using the I^2^ statistic [91], calculated using the DerSimonian and Laird estimator for dichotomous outcome analyses, and the Restricted Maximum Likelihood (REML) estimator for continuous outcome analyses. We considered a value greater than 50% as evidence of substantial heterogeneity. We did not present pooled results where I^2^ values exceeded 75%.

Where studies were not pooled, but we had numerical data, we still provide effect estimates for individual RCTs and generated forest plots. Where there was insufficient data to calculate effect estimates, we summarised the information available and presented this information in effect direction plots. This is also the case when data has been presented per type of AE, rather than for all types together.

Data from single‐armed intervention studies are also summarised in effect direction plots.

Previous updates included network meta‐analyses which we have removed, as there is a more comprehensive network meta‐analysis available [7]. Differences in methods from the protocol and between review updates are documented in Supplementary material 10.

#### Investigation of heterogeneity and subgroup analysis

We had planned to undertake subgroup analyses to investigate differences between studies, such as the following:

- Intensity of behavioural support used (as this could potentially influence our critical outcome: smoking cessation);
- Type of EC, e.g. cartridge; refillable; pod; disposable (as this could potentially influence all outcomes due to different delivery mechanisms);
- Instructions for EC use, e.g. study provision, length of provision, whether participants had a role in product choice (as this could potentially influence all outcomes, given variation in available devices and liquids);
- Type of participants (this could potentially influence all outcomes, depending on, e.g. pre‐existing conditions, previous experience with EC).

However, there were too few studies to conduct such analyses. Should further studies become available in the future, we will follow this approach. For continuous outcomes, we will subgroup data based on whether absolute values or change scores were available. We will create separate subgroups for pregnant study populations because pregnancy affects nicotine metabolism.

In the absence of sufficient data for subgroup analyses on EC type, in the text we specify the type of nicotine EC when reporting pooled results for cessation.

##### Equity‐related assessment

We did not plan to investigate health inequity in this review, as this is explored in a separate Cochrane review [92].

#### Sensitivity analysis

We conducted sensitivity analyses to detect whether pooled results were sensitive to the removal of studies judged to be at high risk of bias overall, and to the removal of studies reporting funding by the tobacco and/or vaping industry. We did this for all outcomes.

#### Certainty of the evidence assessment

Following standard Cochrane methodology, we created summary of findings tables for our three main comparisons using [93]: nicotine EC versus non‐nicotine EC; nicotine EC versus NRT; and nicotine EC versus behavioural support only/no support. We selected these comparisons a priori as being the most clinically relevant.

In the summary of findings tables, we present data on our primary outcomes (cessation at longest follow‐up, at least six months from baseline, and adverse events and serious adverse events at one week or longer, at the longest follow‐up at which participants were still being provided or encouraged to use EC) for these main comparisons.

Following standard Cochrane methodology, we used the five GRADE considerations (study limitations, consistency of effect, imprecision, indirectness, and publication bias) to assess the certainty of the body of evidence for each outcome, and to draw conclusions about the certainty of evidence within the text of the review. GRADE assessments were carried out by JHB and NL.

### Consumer involvement

Panels (size range: five to fifteen) of people with diverse vaping and smoking experiences from different social backgrounds have reviewed the methods of this review and attended periodic workshops to discuss the findings of review updates, how to disseminate these, outcomes measured and any potential changes to review methods.

## Results

### Description of studies

#### Results of the search

For this update, our bibliographic database searches identified 2300 non‐duplicate records (See Figure for PRISMA flow diagram). We screened all records and retrieved the full‐text papers of 131 potentially relevant articles. After screening and checking the full texts, we included 14 new completed studies (Avila 2024 [94, 95]; Higgins 2024 [96, 97, 98, 99, 100, 101, 102]; Hoeppner 2024 [103]; Ikonomidis 2024 [104, 105]; Kale 2025 [106, 107]; Katz 2025 [108, 109]; Kouroutzoglou 2024 [110]; NCT03113136 [111, 112]; Pericot‐Valverde 2025 [113, 114]; Rabenstein 2024 [115]; Sifat 2024 [116]; Smith 2025 [117, 118, 119]; Tuisku 2024 [120, 121]; Vojjala 2025 [122, 123, 124, 125, 126]), 45 new articles linked to studies already included, and 13 new ongoing studies (see Supplementary material 5). Secondary study reports are linked to primary study papers in the reference section.

**1:**
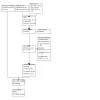
PRISMA diagram for 2025 update

#### Included studies

In total, we have included 104 studies. Key features of these included studies are summarised below and in Table. Further details on each included study can be found in the characteristics of included studies tables (Supplementary material 2). This update includes information on five new comparisons: nicotine EC vs oral nicotine pouches (outcomes: CO, AEs, SAEs); nicotine EC vs non‐nicotine EC + varenicline (outcomes: AEs, SAEs); nicotine EC vs NRT + bupropion (outcomes: abstinence, continued study product use); high vs low wattage EC (outcomes: abstinence, AEs, SAEs, CO); and nicotine EC + very low nicotine cigarettes (VLNC) vs VLNC (outcomes: AEs, SAEs, CO, NNAL). Data have been added to six existing comparisons: nicotine EC vs NRT (outcomes: cessation, continued EC use, CO, AEs, SAEs, respiratory health); nicotine EC vs behavioural support only/no support (outcomes: cessation, CO, NNAL, respiratory and cardiac health); nicotine EC vs non‐nicotine EC (outcomes: cessation, continued EC use, AEs, SAEs, respiratory and cardiac health); nicotine EC vs heated tobacco (outcomes: CO, AEs, SAEs); choice of EC flavour vs tobacco flavour EC (outcomes: AEs, SAEs, CO, NNAL); and higher nicotine EC vs lower nicotine EC (outcomes: respiratory and cardiac health).

**1:** Overview of included studies

Study ID	Number randomised	Study arms	Multiple arms or single arm or cross‐over study	Length follow‐up (months)	Motivated to quit smoking	Specific population characteristics (e.g. pregnant or HIV+ve)	Overall RoB judgement	Tobacco or EC industy‐ funded	Country
Adriaens 2014	48	1) Nicotine EC1. 2) Nicotine EC2 3) Control	Multiple	6			Unclear	No	Belgium
Auer 2024 [360, 361, 362, 363, 364, 365, 366, 367, 368, 369, 370, 371, 372, 373, 374, 375, 376, 377, 378, 379, 380, 381, 382, 383, 384, 385]	1246	1) Nicotine EC. 2) Control	Multiple	24			High	No	Switzerland
Avila 2024	45	1) Nicotine EC. 2) Nicotine pouch. 3) Control (smoking as usual)	Multiple	2	No	Lower economic status	High	No	USA
Baldassarri 2018	40	1) Nicotine EC + NRT (patch) + counselling. 2) Non‐nicotine EC + NRT (patch) + counselling)	Multiple	6	Yes ('willing to quit smoking')	[Recruitment: outpatient pulmonary and primary care clinics, Tobacco Treatment Service, referrals from medical providers]	High	No	USA
Begh 2021	325	1) Nicotine EC. 2) Control (standard care)	Multiple	8	No	Diagnosed with 1 or more of the following chronic conditions: ischaemic heart disease, peripheral vascular disease, hypertension, diabetes mellitus (Type 1 and Type 2), stroke, asthma, COPD, chronic kidney disease, depression, schizophrenia, bipolar disorder or other psychoses	High	No	UK
Bonafont Reyes 2022	48	1) Nicotine EC. 2) NRT	Multiple	3	Yes	Moderate COPD	Unclear	Not reported	USA
Bullen 2013	657	1) Nicotine EC. 2) Nicotine patch (NRT). 3) Non‐nicotine EC. plus all participants referred to quitline	Multiple	6	Yes		Low	No	New Zealand
Caponnetto 2013a*	300	1) Nicotine EC 7.2 mg for 12 weeks. 2) Nicotine EC 7.2 mg for 6 weeks, then 5.4 mg for 6 weeks. 3) Non‐nicotine EC	Multiple	12	No (not currently or intending to quit smoking in the next 30 days)	In good health	Unclear	Yes	Italy
Caponnetto 2013b*	14	1) Nicotine EC	Single	12	No	Diagnosis of schizophrenia	High	Yes	Italy
Caponnetto 2021*	40	1) Nicotine EC	Single	6	No	Diagnosis of schizophrenia	High	Yes	Italy
Caponnetto 2023*	220	1) Nicotine EC. 2) Heated tobacco products (HTP)	Multiple	6	No		High	Yes	Italy
Carpenter 2017 [386, 387, 388]	68	1 Nicotine EC. 2) CC	Multiple	4	Medium interest in quitting smoking		High	No	USA
Carpenter 2023 [389, 390, 391, 392, 393, 394, 395]	638	1) Nicotine EC. 2) No intervention	Multiple	6	Limited interest in quitting smoking		High	No	USA
Cobb 2021	520	1) Nicotine EC (36 mg). 2) Nicotine EC (8 mg). 3) Non‐nicotine EC. 4) Cigarette substitute	Multiple	8.5	No (but interested in reducing)		Low	No	USA
Coffey 2020 [396]	1022	1) Nicotine EC (1 arm, 2 strengths, 4 flavours)	Single	1	Yes	Socially deprived area	High	No	UK
Czoli 2019	48	1) EC to CC to no product. 2) CC to EC to no product (within participant cross‐over)	Cross‐over	0.75	No	Dual users of EC and CC	High	No	Canada
Dawkins 2020	80	1) Nicotine EC. 2) Usual care	Multiple	6	Varied considerably	People accessing homeless support services on a regular basis	High	No	UK
Edmiston 2022*	450	1) Nicotine EC (tobacco flavour). 2) Nicotine EC (menthol flavour). 3) No intervention	Multiple	3	Willing to replace CC with EC		High	Yes	USA
Edwards 2023	30	1) Nicotine EC	Single	6	Willing to attempt to quit	People living with HIV	High	No	Australia
Eisenberg 2020	376	1) Nicotine EC + counselling. 2) Non‐nicotine EC + counselling. 3) Counselling	Multiple	12	Yes		Low	No	Canada
Eisenhofer 2015	11	1) Nicotine EC. 2) NRT	Multiple	0.75		Veterans	Unclear	No	USA
Elling 2023	331	1) Tailored EC information. 2) Control (no tailored EC information)	Multiple	6	Yes (within 5 years)		High	No	Netherlands
Ely 2013	48	1) Nicotine EC. All used EC, 16 also used buproprion, 2 also used varenicline + '5 A's' model and transtheoretical model for smoking cessation	Single	6	Yes (or switch to EC)		High	NR	USA
Felicione 2019	25	1) Nicotine EC. 2) Non‐nicotine EC	Multiple	1	Quit ladder score average: 5.6 (range 1 to 10)	Opioid dependency	Unclear	NR	USA
George 2019	145	1) Nicotine EC. 2) Non‐nicotine EC. 3) CC	Multiple	1	Yes (in nicotine EC and non‐nicotine EC groups)		High	No	UK
Goniewicz 2017	22	1) Nicotine EC	Single	0.5	Yes		High	No	Poland
Bonevski 2021 [397, 398, 399]	100	1) Nicotine EC. 2) NRT	Multiple	3	Median (SD) = 7.3 (2.4) on 1 to 10 scale with 10 "highly motivated"		High	No	Australia
Hajek 2015a	100	Nicotine EC and stop‐smoking medication (NRT, varenicline) were offered, with weekly support. Not randomised	Single	1	Yes		High	No	UK
Hajek 2019	886	1) Nicotine EC. 2) NRT	Multiple	12	Yes		Low	No	UK
Hajek 2022	1140	1) Nicotine EC. 2) NRT.	Multiple	6+	Yes	Pregnant women	Low	No	UK
Halpern 2018	6006	1) Usual care, quit‐smoking programme (Vitality). 2) as (1) plus Nicotine EC. 3) as (2) plus access to free NRT, bupropion or varenicline. 4) as (3) plus incentives for quitting. 5) as (4) plus money at start and lose money if participant did not test negative across 6 months	Multiple	12	Yes 28%. No 9%. Quit later 62%	Employees and spouses at 54 companies that used Vitality wellness programmes	High	No	USA
Hatsukami 2020	264	1) Nicotine EC complete substitution for CC. 2) Nicotine EC partial substitution for CC. 3). NRT complete substitution for CC. 4) CC	Multiple	2	No		Unclear	No	USA
Hickling 2019	50	1) Nicotine EC	Single	6	No	People with severe mental illness (schizophrenia or bipolar diagnosis)	High	No	UK
Higgins 2024	326	1) Nicotine EC (tobacco flavour) + VLNC. 2) Nicotine EC (preferred flavour) + VLNC. 3) VLNV. 4) CC	Multiple	4	No	Vulnerable populations: people with affective disorders; opioid use; women of reproductive age with maximum educational attainment of graduating from high school	Unclear	No	USA
Hoeppner 2024	29	1) Nicotine EC	Single	3	Motivation to quit 7.9 (2.2 SD)	Socioeconomically disadvantaged	High	No	USA
Holliday 2019 [400, 401, 402]	80	1) Nicotine EC + standard stop‐smoking advice. 2) Standard stop‐smoking advice only	Multiple	6	NR	People diagnosed with periodontitis (setting dental clinic)	High	No	UK
Humair 2014	17	1) Nicotine EC	Single	12	Yes		High	NR	Switzerland
Ikonomidis 2018	90	1) Nicotine EC	Single	1	Yes (attending smoking cessation clinic)		High	No	Greece
Ikonomidis 2020a	40	1) Nicotine EC. 2) CC	Multiple	4	Yes (attending smoking cessation clinic)		Unclear	No	Greece
Ikonomidis 2020b [403]	40	1) Nicotine EC. 2) CC	Multiple	1	Yes (attending smoking cessation clinic)		High	No	Greece
Ikonomidis 2024	100	1) Nicotine EC. 2) Heat not burn cigarette. 3) Control	Multiple	1	Yes		Unclear	No	Greece
Ioakeimidis 2018	54	1) Nicotine EC + low intensity counselling. 2) Varenicline + low level counselling	Multiple	6	Yes	Participants hospitalised with acute coronary syndrome	High	NR	Greece
Kale 2025	43	1) Nicotine EC. 2) Usual care	Multiple	1	Willing to quit.	All participants had a diagnosed mental health condition and were receiving treatment in primary or secondary care	High	No	UK
Kanobe 2022*	125	1) Nicotine EC (Vuse solo 4.8%, 57.4 mg/mL. 2) Nicotine EC (Vuse ciro 1.5%). 3) Nicotine EC (Vuse vibe 3%, 36 mg/mL). 4) Abstinence	Multiple	0.25	No		High	Yes	USA
Katz 2025	21	Two 2‐week phases. CC phase. EC phase	Cross‐over	0.5	NR	COPD diagnosis	High	No	USA
Kerr 2020	55	1) Nicotine EC + behavioural support. 2) NRT + behavioural support	Multiple	3	Willing to quit		Low	No	UK
Kimber 2021	50	1) Nicotine EC cig‐a‐like 18 mg/mL. 2) Nicotine EC tank 18 mg/mL. 3) Nicotine EC tank 6 mg/mL	Multiple	0.5	Willing to quit		High	No	UK
Klonizakis 2022	248	1) Nicotine EC. 2) Non‐nicotine EC. 3) Referral to NHS stop‐smoking clinic (NRT + behavioural support)	Multiple	6	Yes		Unclear	No	UK
Kouroutzoglou 2024	57	1) Nicotine EC. 2) NRT. 3) NRT + buproprion	Multiple	6	NR	People with obesity	Unclear	No	Greece
Kumral 2016	98	1) Nicotine EC. 2) Cognitive behaviour therapy	Multiple	3	Willing to quit		High	NR	Turkey
Lee 2018	30	1) Nicotine EC. 2) NRT	Multiple	6	NR	Veterans awaiting surgery	Low	No	USA
Lee 2019	150	1) Nicotine EC. 2) NRT. Both arms received a 50‐minute smoking cessation education session	Multiple	6	Yes	All male (motor company)	Low	No	Korea
Lucchiari 2022	210	1) Nicotine EC. 2) Non‐nicotine EC	Multiple	12	Yes	Participants in the early lung cancer detection programme (Cosmos II)	High	No	Italy
Martinez 2021	2896	1) Smoking cessation self‐help booklet targeted to dual users. 2) Generic smoking cessation self‐help booklet. 3) Assessment only	Multiple	24	Not required to be motivated to quit	Dual users of EC and CC	Low	No	USA
Martner 2019 [404]	12	1) Nicotine EC	Single	1	Yes		High	No	USA
McRobbie 2015 [405, 406, 407]	40	1) Nicotine EC + standard behavioural support	Single	1	Yes		High	No	UK
Meier 2017	24	1) Nicotine EC. 2) Non‐nicotine EC	Multiple	0.5	No		Unclear	No	USA
Morphett 2022a	1712	1) Usual care standard cessation advice + NRT (short term). 2) Quit or substitute advice + NRT (advice to use NRT longer term). 3) Quit or substitute advice + NRT and /or EC	Multiple	12	58% wanted to quit a lot		High	No	Australia
Morphett 2022b	355	1) Quitline + NRT + EC. 2) Quitline + NRT At 6 months, arm 2 participants still smoking switched to EC (nicotine vaporiser) intervention.	Multiple	24	Yes (referred to quitline)	Diagnosed with/treatment for HIV or hepatitis C (HCV) or receiving opioid substitution therapy (OST) or receiving treatment for priority health conditions	Unclear	No	Australia
Morris 2022*	79	1) Nicotine EC	Single	0.5	No		High	Yes	USA
Myers‐Smith 2022	135	1) Nicotine EC. 2) NRT. Both groups: minimal behavioural support	Multiple	6		People who find quitting difficult	Low	No	UK
NCT02648178	19	1) Nicotine EC	Single	3	No	People with smoking‐related cancers	High	No	USA
NCT02918630 [408]	7	1) Nicotine EC. 2) NRT	Multiple	1	No	Diagnosis of schizophrenia	Unclear	No	USA
NCT03113136	372	1) Nicotine EC (low wattage). 2) Nicotine EC (high wattage). 3) CC	Multiple	12	No		High	No	USA
Nides 2014*	29	1) Nicotine EC	Single	0.5	No		High	Yes	USA
Okuyemi 2022 [409, 410]	234	1) Nicotine EC. 2) Non‐nicotine EC	Multiple	3	No	African‐American	Unclear	No	USA
Oncken 2015	27	Cross‐over study. Nicotine EC tobacco flavour. Nicotine EC tobacco and menthol flavour	Cross‐over	0.5	No		Unclear	No	USA
Ozga‐Hess 2019	60	1) Nicotine EC. 2) CC	Multiple	2	Yes		High	No	USA
Pacifici 2015	34	1) Nicotine EC	Single	8	No		High	No	Italy
Pericot‐Valverde 2025	30	1) Nicotine EC	Single	2	NR	Adults with opioid use disorder on buprenorphine	High	No	USA
Piper 2025	209	1) EC + active patch (NRT) week 1, placebo patch week 2. 2) EC + placebo patch week 1, active patch week 2. 3) VLNC + active patch week 1, placebo patch week 2. 4) VLNC + placebo patch week 1, active patch week 2. 5) Placebo patch week 1, active patch week 2. 6) Active patch week 1, placebo patch week 2	Multiple	1	No		High	No	USA
Polosa 2011*	40	1) Nicotine EC	Single	24	No		High	Yes	Italy
Polosa 2014b*	50	1) Nicotine EC	Single	6	No		High	Yes	Italy
Polosa 2015*	71	1) Nicotine EC	Single	12	NR	Participants making first purchase at participating vape shop	High	Yes	Italy
Pope 2024	972	1) Nicotine EC. 2) Brief smoking cessation advice. 3) Referral to stop‐smoking services	Multiple	6	NR	People attending the Emergency Department	High	No	UK
Pratt 2016	19	1) Nicotine EC	Single	1	No	Diagnosis of serious mental illness	High	No	USA
Pratt 2022	240	1) Nicotine EC. 2) Assessment only	Multiple	6	No	Diagnosis of schizophrenia, schizoaffective disorder, or bipolar disorder	High	No	USA
Price 2022	871	1) Nicotine EC	Single	12	Self‐presented. Assumed interest in quitting /free EC		High	No	UK
Pulvers 2018	40	1) Nicotine EC	Single	1	55% not intending to quit CC		High	No	USA
Pulvers 2020	186	1) Nicotine EC. 2) CC	Multiple	6	NR. Willing to switch to EC for 6 weeks		High	No	USA
Rabenstein 2024	80	1) Nicotine EC. 2) Cognitive behaviour therapy smoking cessation programme + NRT	Multiple	3	Yes		High	'No funding sources'	Germany
Rose 2023*	94	1) Nicotine EC + nicotine patch. 2) Non‐nicotine EC + nicotine patch	Multiple	2	NR		Unclear	Yes	USA
Russell 2021*	426	1) Nicotine salt EC. 2) Nicotine free‐based EC. 3) NRT	Multiple	6	NR		Unclear	Yes	UK
Scheibein 2020	23	1) Nicotine EC	Single	3	Yes	People accessing a homeless supported temporary accommodation service	High	No	Ireland
Sifat 2024	60	1) Nicotine EC. 2) Nicotine EC + financial incentives	Multiple	2	NR. Willing to switch to EC	People accessing shelter services	High	No	USA
Skelton 2022	66	1) Nicotine EC abrupt CC cessation. 2) Nicotine EC gradual CC cessation	Multiple	3	Yes	Clients of alcohol or drug centre	High	No	Australia
Smith 2020 [411]	30	1) Nicotine EC PG/VG ratio 70/30. 2) Nicotine EC PG/VG ratio 50/50. 3) Nicotine EC PG/VG ratio 0/100	Multiple	0.25	NR		Unclear	No	USA
Smith 2025	30	1) Nicotine EC. 2) NRT	Multiple	1	Yes	People who have failed to quit with pharmacotherapy	Unclear	No	USA
Stein 2016 [412]	12	1) Nicotine EC	Single	2	Yes	People receiving methadone‐maintained treatment for opoid use disorder	High	No	USA
Strasser 2016 [413]	24	1) Nicotine EC (5 brands (4 analysed))	Multiple (factorial trial)	0.3	No		High	No	USA
Tattan‐Birch 2023	92	1) EC + varenicline. 2) Varenicline only	Multiple	3	Yes		High	No	UK
Tseng 2016	99	1) Nicotine EC. 2) Non‐nicotine EC	Multiple	0.75	Willing to reduce CC		Unclear	No	USA
Tuisku 2024	458	1) Nicotine EC + placebo varenicline tablets. 2) Varenicline + non‐nicotine EC. 3) Non‐nicotine EC + placebo tablets. All arms offered 8 motivational interview sessions	Multiple	12	Yes		Low	No	Finland
Valentine 2018	50	1) Nicotine EC	Single	2	No	People receiving psychiatric services from Department of Veterans Affairs healthcare system	High	No	USA
Van Staden 2013*	15	1) Nicotine EC	Single	0.5	NR		High	Yes	South Africa
Vickerman 2022	110	1) Enhanced EC coaching quitline, NRT available + EC advice only. 2) Quitline treatment‐as‐usual, NRT available	Multiple	3	Yes	Dual users of EC and CC	Unclear	No	USA
Vojjala 2025	121	1) Nicotine EC + counselling. 2) NRT + counselling	Multiple	6	Yes	Patients with COPD/chronic disease	High	No	USA
Wadia 2016 [414]	20	1) Nicotine EC	Single	0.5	No	Dental patients	High	NR	UK
Wagener 2023	350	1) Nicotine EC + counselling. 2) Quitline treatment‐as‐usual + NRT + counselling	Multiple	3	Participated in tobacco helpline	Quitline recent treatment failure	Unclear	No	USA
Walele 2018*	209	Phase 1 (RCT): 1) Nicotine EC. 2) CC. 3 months Phase 2 (single arm): 1) Nicotine EC. Follows for 24 months	Multiple (phase 1). Single (phase 2)	24	No		High	Yes	UK
Walker 2020	1124	1) Nicotine EC + nicotine patch. 2) Non‐nicotine EC + nicotine patch. 3) Nicotine patch	Multiple	12	Yes		High	No	New Zealand
White 2022	50	1) CC + moderate nicotine liquid (1.8% free‐based nicotine) + tobacco flavours 2) CC + low nicotine liquid (0.3% free‐based nicotine) + tobacco flavours (0.3% free‐based nicotine) 3) CC + moderate nicotine liquid + varied flavours 4) CC + low nicotine liquid + varied flavours 5) VLNC CC + moderate nicotine liquid + tobacco flavour 6) VLNC CC + low nicotine liquid + tobacco flavours 7) VLNC CC + moderate nicotine liquid + varied flavours 8) VLNC CC + low nicotine liquid + varied flavours	Multiple	3	NR		High	No	USA
Xu 2023*	837	1) Nicotine EC tobacco flavour. 2) Nicotine EC flavour choice. 3) Quit advice	Multiple	12	No		High	Yes	USA
Yingst 2020	17	1) Nicotine EC cig‐a‐like. 2) Nicotine EC refillable	Multiple	0.75	No	Documented history of positive HIV status	Unclear	No	USA

Abbreviations: CC: combustible cigarettes COPD: chronic obstructive pulmonary disease EC: electronic cigarettes HCV: hepatitis C HIV: human immunodeficiency virus HTP: heated tobacco products NHS: National Health Service NRT: nicotine replacement therapy OST: opioid substitution therapy PG: propylene glycol  RoB: risk of bias SD: standard deviation VG: vegetable glycerine VLNC: very low nicotine content

##### Participants

The 104 included studies represent 30,366 participants. Forty‐eight studies were conducted in the USA, 21 in the UK, nine in Italy, six in Greece, five in Australia, two each in New Zealand, Switzerland, and Canada, and one each in Belgium, Finland, Germany, Ireland, the Netherlands, Poland, the Republic of Korea, South Africa, and Turkey. All studies were conducted in adults who smoked. Thirty studies exclusively recruited participants who were not motivated to quit smoking, and 50 studies exclusively recruited participants motivated to quit; motivation was not specified for the other studies. Thirty‐seven studies recruited from specific population groups; these included eleven studies that recruited participants based on a physical health condition (heart attack, cancer, HIV, periodontitis, awaiting surgery, smoking‐related chronic disease, obesity), six studies that recruited participants with serious mental illness, five studies that recruited participants on treatment or having recently completed treatment for alcohol or other drug use, and three studies in dual users of EC and combustible cigarettes. Three studies recruited people accessing homeless centres or using supported temporary accommodation, and a further four recruited at‐risk populations or those specifically within socially deprived areas. One study each recruited: people aged 55 or older; young adults; people who self‐identified as African‐American; pregnant women; people who had recently made a failed attempt to quit smoking; black and Latino participants; and people attending the emergency department.

##### Interventions and comparators

Three studies recruited dual users of combustible cigarettes and EC at baseline, and instructed them to continue using their own EC devices (Czoli 2019 [127]; Martinez 2021 [128, 129, 130, 131, 132, 133, 134]; Vickerman 2022 [135, 136, 137]). One study recruited users of combustible cigarettes only and provided information on using EC, but did not provide them with EC (Elling 2023 [138, 139, 140]). The remaining studies all provided some form of nicotine EC.

In two studies where nicotine EC were provided on their own, nicotine levels were judged to be so low as to be clinically comparable to non‐nicotine EC (Lee 2019 [141, 142]; Van Staden 2013* [143]); we include these studies in non‐nicotine EC comparisons. Twelve studies compared nicotine EC with non‐nicotine EC, 28 studies compared nicotine EC to behavioural support only or to no support, and 24 studies compared nicotine EC to NRT. Six studies compared high‐ versus low‐nicotine EC devices (Caponnetto 2013a* [144, 145, 146, 147, 148, 149, 150, 151]; Cobb 2021 [152, 153, 154, 155, 156, 157, 158, 159, 160, 161, 162]; Kanobe 2022* [163, 164, 165] Kimber 2021 [166]; Morris 2022* [167, 168, 169]; White 2022 [170, 171, 172]), five studies included comparisons based on flavours (Edmiston 2022* [173]; Higgins 2024; Morris 2022*; White 2022; Xu 2023* [174]), two studies directly compared device types (Kimber 2021; Yingst 2020 [175]), two studies directly compared a free‐based nicotine to a salt‐based nicotine device (Morris 2022*; Russell 2021* [176]), and one compared higher versus lower wattage EC (NCT03113136). Results from these studies are reported by comparison in Synthesis of results. Further details on the intervention and comparator groups (where applicable) for each study can be found in the Supplementary material 2 table.

Where reported in the primary research publications, details of the devices tested can also be found in the characteristics of included studies tables (Supplementary material 2). Of the studies with sufficient data with which to judge, 32 used cartridge devices, 38 used refillable devices, four used both types, 15 used a pod device, and one used disposable EC. The remainder did not report device type.

##### Outcomes

Of the 104 included studies:

- 45 reported data on abstinence at six months or longer;
- 70 reported data on adverse events;
- 53 reported data on serious adverse events;
- 57 reported data on carbon monoxide;
- 12 reported data on heart rate;
- 15 reported data on blood pressure;
- 4 reported data on blood oxygen saturation;
- 19 reported data on at least one known toxicant/carcinogen;
- 10 reported data on at least one measure of lung function;
- 21 reported data on study product use at six months or longer.

One study measured safety outcomes but did not report them in the text available at the time of writing; hence, this study currently does not contribute any data to this review (Skelton 2022 [177, 178, 179]).

##### Study types

Sixty‐one studies were RCTs, 32 of which contributed to cessation analyses. Nine studies used randomised cross‐over designs, and the remainder were uncontrolled cohort studies.

##### Funding

Of the 97 studies that reported funding information, 16 reported support from the tobacco or vaping industry, or that authors had received tobacco or vaping industry support outside the study being conducted, and 81 had no tobacco or EC industry funding or support. Below, we detail the industry funding from the 16 studies that report tobacco or EC industry funding or support. An asterisk (*) indicates studies that received tobacco or EC industry funding.

Six studies received funding from the Lega Italiana AntiFumo (Caponnetto 2013a*; Caponnetto 2013b* [180, 181]; Caponnetto 2021* [182]; Polosa 2011* [183, 184, 185]; Polosa 2014b* [186, 187]; Polosa 2015* [188]). Caponnetto 2013b* and Polosa 2011* also received free “Categoria” EC kits from the Arbi Group Srl (Milano, Italy). Caponnetto 2021* received free JUUL EC from the manufacturer, PAX Labs (became JUUL Labs in 2017). Altria Group (formerly, Philip Morris Companies) acquired a 35% stake in JUUL Labs on 20 December 2018; the study was completed before Altria invested in JUUL. Polosa 2014b* thank FlavourArt (Oleggio, NO, Italy; www.flavourart.it), an EC flavour company.

Caponnetto 2023* [189, 190, 191] was funded by Philip Morris Product Société Anonyme.

Edmiston 2022* was funded by Altria Client Services LLC. Altria is the parent company of Philip Morris USA (producer of Marlboro cigarettes), John Middleton, Inc., U.S. Smokeless Tobacco Company, Inc., and Philip Morris Capital Corporation.

Kanobe 2022* was funded by RAI Services Company. The parent company is Reynolds American. Reynolds American manufacture and market a variety of tobacco products, including cigarettes (Newport, Camel, Pall Mall, Kent, Doral, Misty, Capri, and Natural American Spirit brands), EC (Vuse brand), and moist snuff (Grizzly and Kodiak brands).

Morris 2022* was funded entirely by Fontem US LLC, a subsidiary of Imperial Brands PLC.

Nides 2014* [192, 193] was funded by NJOY, Inc., Scottsdale, AZ, part of the EC/alternative nicotine products industry.

Rose 2023* [194, 195, 196] was funded by the National Institute on Drug Abuse (NIDA). However, the lead author declared research support from Foundation for a Smoke‐Free World (which has links to the tobacco industry), Philip Morris International, Altria, Embera Neuro Therapeutics, Inc., Otsuka Pharmaceutical, JUUL Labs, consulting with Revive Pharmaceuticals, and consulting and patent purchase agreements with Philip Morris International.

Russell 2021*was funded by the e‐cigarette/alternative nicotine products industry.

Van Staden 2013* was funded by eGo e‐cigarette packs by Twisp.

Walele 2018* [197, 198, 199, 200] was funded and supported by Fontem Ventures B.V. Imperial Brands plc (Imperial Tobacco plc) is the parent company of Fontem Ventures B.V., the manufacturer of the EC prototype used in their study.

Xu 2023* was funded by JUUL Labs, Inc.

#### Excluded studies

We list 33 studies excluded at full‐text stage across all updates of this review (this does not cover all studies ever excluded, but those that are potentially most likely to require explanation), along with reasons for exclusion, in the characteristics of excluded studies table (Supplementary material 3). For this update specifically, after the reference being a duplicate, the most common reason for exclusion was that studies did not include outcomes relevant to this review. Two studies are listed as awaiting classification, as there is insufficient information to judge their eligibility (Supplementary material 4).

### Risk of bias in included studies

Overall, we judged 11 studies to be at low risk of bias (Bullen 2013 [201, 202, 203, 204, 205]; Cobb 2021; Eisenberg 2020 [206, 207, 208, 209, 210]; Hajek 2019 [211, 212, 213, 214]; Hajek 2022 [215, 216, 217, 218, 219, 220]; Kerr 2020 [221, 222]; Lee 2018 [223, 224, 225, 226]; Lee 2019; Martinez 2021; Myers‐Smith 2022 [227, 228]; Tuisku 2024), 23 to be at unclear risk, and the remaining 70 at high risk of bias (this includes the 34 non‐randomised studies, which we deemed to be at high risk due to lack of randomisation).

Details of the risk of bias judgements for each domain for each included study can be found in the characteristics of included studies tables (Supplementary material 2). Figure and Figure illustrate our judgements across included studies.

**2:**
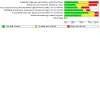
Risk of bias graph

**3:**
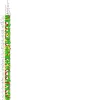
Risk of bias summary

We judged 30 studies to be at high risk of selection bias for randomisation of allocation; for the majority of cases, this is because the study was not randomised. We rated a pilot cluster‐randomised trial to be at high risk, as randomisation was not carried out as intended for pragmatic reasons (Dawkins 2020). We judged 42 studies to be at low risk, and the remainder to be at unclear risk as there was insufficient information with which to judge. For allocation concealment, we rated 27 studies as being at high risk of bias, 35 at low risk, and the remaining studies at unclear risk.

In all, we assessed 76 studies for performance bias and 79 for detection bias (see Methods for why this was not assessed for all studies). For performance bias, we rated 40 to be at low risk, 25 at high risk, and 11 at unclear risk. For detection bias, we rated 57 as low risk, and 12 as high risk. In these studies, blinding was not used and different levels of support were provided; this alone, or in conjunction with the outcome measures being used (subjective rather than objective measures), meant that we thought there was a high risk of bias being introduced. We judged the rest to be at unclear risk, or ineligible for this domain due to single‐arm design.

We judged most studies (74 out of 104) to be at low risk of attrition bias. We rated 12 studies with substantial loss to follow‐up as being at high risk of attrition bias. The remainder did not provide sufficient data on which to judge, and hence we judged them to be at unclear risk.

Of the 104 studies, we considered 52 to be at low risk of reporting bias, as all prespecified or expected outcomes were reported. We rated 12 as being at high risk, as data were not available as specified in the original protocols (note, in some cases these are recent studies, and judgement on these may change as more publications emerge). We judged the rest to be at unclear risk, due to insufficient information with which to make a judgement.

We considered Ioakeimidis 2018 [229] to be at high risk of other bias; data were from a conference poster and the associated abstract, and quit rates in the intervention arm differed between the two sources. We considered five further studies to be at unclear risk in this domain.

### Synthesis of results

Data on our outcomes of interest are summarised below and in our Table, Table, and Table. Forest plots are available through 'analysis' links; for some outcomes, benefit is plotted on the right; for others it is plotted on the left. This is due to direction of effect, e.g. an increase in cessation is a benefit, whereas an increase in a carcinogen is not. Axes are labelled accordingly.

#### Direct comparisons between nicotine EC and other pharmacotherapies

Comparisons reported here include nicotine EC versus NRT, nicotine EC versus varenicline, and EC versus combination therapy such as NRT plus bupropion, and non‐nicotine EC plus varenicline. Only RCTs contributed data.

##### Cessation

Pooled data from nine studies (two cartridges, four refillable, one pod, one disposable, one not specified), five of which were rated as being at low risk of bias (Bullen 2013; Hajek 2019; Hajek 2022; Lee 2018; Myers‐Smith 2022), three as unclear (Klonizakis 2022 [230, 231, 232, 233, 234]; Kouroutzoglou 2024; Russell 2021*) and one as high risk (Vojjala 2025), showed increased quit rates in people randomised to nicotine EC when compared with NRT (RR 1.55, 95% CI 1.28 to 1.88; I² = 0%; 2703 participants; Figure). The certainty of evidence is high and has not been downgraded. One study included in this analysis, Hajek 2022, was conducted in pregnant women. There was no evidence of a subgroup difference between this study and studies in participants not selected on the basis of pregnancy (P = 0.81, I^2^ for subgroup differences = 0%). Follow‐up time was based on the end of pregnancy, and our analysis included only those participants with follow‐up of at least six months. Results were not sensitive to the exclusion of the one study that received industry funding (Russell 2021*) or the one study at high risk of bias (Vojjala 2025); when each study was removed, the point estimates were 1.60 and 1.54 respectively, and the CIs remained consistent with those from the main analysis.

**4:**
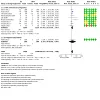
**Analysis 1.1: EC vs NRT ‐ Smoking cessation**

One study, Ioakeimidis 2018, available as a conference presentation only and considered at high risk of bias due to inconsistencies in the data reported, favoured varenicline for quitting compared with nicotine EC (cartridge) (RR 0.31, 95% CI 0.11 to 0.82; 54 participants). In another study, Tuisku 2024 (refillable; low risk of bias), more people quit when randomised to receive a combination of non‐nicotine EC plus varenicline compared with nicotine EC (RR 0.73, 95% CI 0.53 to 1.01; 305 participants), though the CI included the potential for no difference. Ioakeimidis 2018 and Tuisku 2024 were not pooled as in the latter a non‐nicotine EC was provided alongside varenicline, and evidence suggests non‐nicotine EC is more effective than no treatment.

One study, Kouroutzoglou 2024 (device type not specified; unclear risk of bias), available as a conference presentation only and considered at unclear risk of bias, did not find a difference in quit rates between nicotine EC and combination NRT plus bupropion (RR 0.89, 95% CI 0.44 to 1.81; 38 participants).

##### Adverse events

Pooled data from seven studies (2 cig‐a‐like, 2 refillable, 3 pod; four considered as being at low risk of bias (Bullen 2013; Hajek 2022; Lee 2018; Myers‐Smith 2022), two at unclear risk (Smith 2025; Wagener 2023 [235]), and one at high risk (Piper 2025 [236, 237, 238, 239, 240, 241])) showed that there is probably no difference in the number of participants reporting adverse events (AEs) between nicotine EC and NRT arms (RR 1.00, 95% CI 0.73 to 1.37; I² = 58%; 2241 participants; Figure). The certainty of evidence is moderate, downgraded one level due to imprecision; the CIs were consistent with both benefit and harm. None of the studies contributing data to this analysis received funding from the vaping or tobacco industries.

**5:**
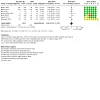
**Analysis 1.2: EC vs NRT ‐ Adverse events**

Hajek 2019 (refillable; low risk of bias) and Bonafont Reyes 2022 [242] (device type not specified; unclear risk of bias) did not contribute data to this meta‐analysis due to the way in which events were recorded. In Hajek 2019's prespecified adverse reactions of interest, nausea was more frequent in the NRT group, throat/mouth irritation was more frequent in the nicotine EC group, and there was little difference in other reactions (see Supplementary material 11 for more detail). Bonafont Reyes 2022 recruited participants with chronic obstructive pulmonary disease (COPD) and reported "a trend towards decreased dyspnoea and COPD symptoms ... in the EC arm compared to the NRT arm", but did not provide further detail.

In Ioakeimidis 2018 (device type not specified; high risk of bias), reports of sleep disorders were evenly distributed between groups, and nausea was more common in the varenicline arm than in the nicotine EC arm (see Supplementary material 11 for more detail). Tuisku 2024 (refillable; low risk of bias) reported more AEs leading to discontinuation of study treatment in the non‐nicotine EC plus varenicline arm (27, 17.6%) compared with the EC arm (15, 9.9%).

##### Serious adverse events

Eight studies (2 cig‐a‐like, 3 refillable, 3 pod; five at low risk of bias (Bullen 2013; Hajek 2019; Hajek 2022; Lee 2018; Myers‐Smith 2022), two at unclear risk (Smith 2025; Wagener 2023), and one at high risk (Piper 2025)) comparing nicotine EC with NRT provided data on serious adverse events (SAEs). In some studies, no events occurred. Pooled results showed that there may be a slight increase in SAEs in the nicotine EC arms compared with NRT. There is low certainty of evidence for this outcome, downgraded two levels due to imprecision; there were fewer than 300 events and wide CIs incorporating no difference, as well as clinically significant harm and clinically significant benefit (RR 1.22, 95% CI 0.73 to 2.03; I² = 30%; 2950 participants; Figure). None of the studies contributing data to this analysis received funding from the vaping or tobacco industries. In Hajek 2022 (conducted in pregnant women), the authors reported no evidence of a difference in birth outcomes overall. However, low birthweight (< 2500 g) was less frequent in the EC than the NRT arm (14.8% versus 9.6%; RR 0.65, 95% CI 0.47 to 0.90).

**6:**
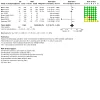
**Analysis 1.3: EC vs NRT ‐ Serious adverse events**

No SAEs occurred in Ioakeimidis 2018 (device type not specified; high risk of bias; Analysis 2.2). In Tuisku 2024 (refillable; low risk of bias), two people reported SAEs in the nicotine EC arm and none in the non‐nicotine EC plus varenicline arm (RR 5.03, 95% CI 0.24 to 103.97; 305 participants).

##### Carbon monoxide (CO)

Pooled data from five studies (Hatsukami 2020 [243, 244]; Kerr 2020; Klonizakis 2022; Lee 2018; Smith 2025; two cig‐a‐like, two refillable, one pod), none of which received tobacco/vaping industry funding and none of which were considered as being at high risk of bias, compared nicotine EC with NRT. CO levels decreased more in those randomised to nicotine EC; however, the CIs incorporated the possibility of no meaningful between‐group difference (MD ‐1.98, 95% CI ‐3.78 to ‐0.18; I² = 0%; 385 participants). A fourth, small study (Eisenhofer 2015 [245]; n = 11; cartridge; unclear risk of bias) was reported as a conference abstract and hence had limited data available. At three weeks, this study showed that both EC and NRT groups had "significantly reduced" CO, but between‐group differences were not reported.

##### Heart rate, blood pressure, and oxygen saturation

Pooled data from two studies comparing nicotine EC with NRT (166 participants; one study judged to be at unclear risk of bias (Hatsukami 2020; cig‐a‐like) and one at low risk (Kerr 2020; refillable), neither in receipt of vaping/tobacco industry funding) showed no clear evidence of a clinically meaningful difference in heart rate (MD 0.53, 95% CI ‐1.76 to 2.83; I² = 0%; 166 participants), systolic blood pressure (MD ‐1.62, 95% CI ‐3.59 to 0.36; I² = 0%; 166 participants), or blood oxygen saturation (MD ‐0.14, 95% CI ‐0.59 to 0.30; I² = 0%; 165 participants), although CIs were wide.

##### Toxicants

Only Hatsukami 2020 (cig‐a‐like; unclear risk of bias, no tobacco/vaping industry funding, n = 111, comparison with NRT) contributed data for these outcomes. For 3‐HPMA, 2‐HPMA, and HMPMA, point estimates favoured EC but CIs included no difference (Analysis 1.8; Analysis 1.10; Analysis 1.11). There was no evidence of a difference for NNAL (nitrosamine 4‐(methylnitrosamino)‐1‐(3‐pyridyl)‐1‐ butanol) but CIs were again wide (Analysis 1.9). For PheT, CEMA, and AAMA (Analysis 1.12; Analysis 1.13; Analysis 1.14), point estimates favoured NRT but CIs included no difference.

##### Lung function

Lee 2018 and Kerr 2020 (one cig‐a‐like and one refillable; both low risk of bias; no tobacco/vaping industry funding, comparison with NRT) measured change in FEV1 (forced expiratory volume) and FEV1/FVC (forced vital capacity) (both low risk of bias; n = 81). High statistical heterogeneity (I^2^ = 89%) precluded pooling for FEV1 (Analysis 1.15). The point estimate for Lee 2018 favoured EC and for Kerr 2020 favoured NRT; for Kerr 2020, the CIs included no difference. There was no evidence of a difference for FEV1/FVC, but there was moderate unexplained statistical heterogeneity and, again, CIs were wide (MD 10.15, 95% CI ‐24.36 to 44.67; I² = 51%; 81 participants). For PEF (peak expiratory flow), only one study contributed to this analysis (Kerr 2020, n = 55). The point estimate favoured NRT but CIs were wide and included no difference (MD ‐3.00, 95% CI ‐27.09 to 21.09).

##### Study product use

Five studies (two refillable, two cig‐a‐like, one pod; none at high risk of bias; one, Russell 2021*, with vaping industry funding) reported study product use at six months or longer, but statistical heterogeneity precluded pooling (I^2^ = 95%). Whereas Russell 2021* (pod device; unclear risk of bias) and Lee 2018 (cig‐a‐like; low risk of bias) found no difference between the EC and NRT arms, in the other three studies, people in the EC arm were more likely to continue to use the study product (EC) than those in the NRT arm (Analysis 1.18). A companion publication explored long‐term rates in more detail [246].

#### Nicotine EC versus other tobacco/nicotine products used for stopping combustible tobacco use

Two studies (Caponnetto 2023*: n = 220; refillable; high risk of bias; tobacco/vaping industry‐funded; Ikonomidis 2024: n = 100; unclear risk of bias), compared nicotine EC with heated tobacco. We considered Caponnetto 2023* to be at high risk of bias due to a lack of blinding alongside strong participant product preferences; Ikonomidis 2024 was at unclear risk. Caponnetto 2023* reported on AEs, SAEs, expired carbon monoxide (eCO), and VO_2_ max as a measure of lung function, heart rate, and blood pressure at 12 weeks follow‐up. Ikonomidis 2024 reported eCO levels at one month. The effect estimate demonstrated no clear evidence of a difference in AEs between the nicotine EC and heated tobacco group (RR 0.86, 95% CI 0.68 to 1.10; I² not applicable; 1 study, 220 participants). There were no SAEs reported in either arm, so an effect estimate could not be calculated (Analysis 5.2). There was no clear evidence of a between‐group difference in eCO levels (MD 1.00, 95% CI ‐1.04 to 3.03; I² = 6%; 2 studies, 267 participants), or VO_2_ max (MD 6.20, 95% CI ‐2.01 to 14.41; 1 study, 211 participants). The following was reported on heart rate and blood pressure and is reported in Supplementary material 14 and Supplementary material 15: “No significant changes in the mean resting heart rate, blood pressure, and BMI during product use were observed between and within study groups.”

One study (Avila 2024; n = 26; cartridge; high risk of bias) compared nicotine EC with oral nicotine pouches (ONPs). This study, at high risk of bias because of potential selective reporting, as urine and blood samples were not analysed, reported on SAEs and CO. No SAEs were reported in either study arm (Analysis 6.1), and CO was lower in the EC arm than in the ONP arm, though CI included the potential for greater CO from EC (MD ‐12.44, 95% CI ‐28.82 to 3.94; 26 participants). Avila 2024 reported participants in the ONP arm were more likely than those in the EC arm to report cough (ONP: 5/12; EC: 3/14) and shortness of breath (ONP: 3/12; EC: 1/14), though the frequency of cough and shortness of breath decreased and was similar between arms by week four (Supplementary material 11).

#### Nicotine EC alone or versus control

Comparisons reported here include nicotine EC versus non‐nicotine EC, and nicotine EC compared to behavioural support only or no support. In this section, we also report results from studies in which all participants received nicotine EC (cohort studies and randomised studies that did not differ across arms in EC provision, device type, or nicotine content).

##### Cessation

###### Randomised controlled trials

At six months or longer, quit rates were higher in nicotine EC groups than in comparator groups. Compared to EC without nicotine (placebo EC), pooled results showed nicotine EC probably produced higher quit rates (RR 1.34, 95% CI 1.06 to 1.70; I² = 0%; 1918 participants; Figure; 5 studies of cartridge and 2 studies of refillable devices). There is moderate‐certainty evidence that nicotine EC probably increases quit rates compared to non‐nicotine EC. The certainty has been downgraded one level due to imprecision; there are fewer than 300 events overall. It has not been downgraded for risk of bias: removing the one study considered at high risk of bias increased the direction of the effect in favour of nicotine EC. The interpretation of the effect remained the same when we removed the one study at high risk of bias (Lucchiari 2022 [247, 248, 249, 250, 251, 252, 253]) and when we removed the one study with tobacco/vaping industry funding (Caponnetto 2013a*). The effect may be more pronounced when comparing nicotine EC to behavioural support only or to no support (RR 1.78, 95% CI 1.42 to 2.25; I² = 13%; 6819 participants; Figure; 11 studies (5 refillable, 3 cartridges, 3 pods)). As this involved unblinded comparisons with unequal levels of support, we judged all data contributing to this outcome to be at high risk of bias (the certainty of the evidence was low, downgraded two levels). One of the studies contributing data to this comparison reported tobacco/vaping industry funding (Xu 2023*). The removal of this study in a sensitivity analysis did not change the interpretation of the effect (Table).

**7:**
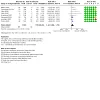
Analysis 7.1: Nicotine EC vs non‐nicotine EC ‐ Smoking cessation

**8:**
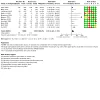
Analysis 8.1: EC vs behavioural support only/no support ‐ Smoking cessation

**2:** Sensitivity analysis for all studies

Comparison	Analysis number	Sensitivity analysis removing studies at high risk of bias	Sensitivity analysis removing industry‐funded studies
Nicotine EC versus NRT	Analysis 1.1	RR 1.59 (1.31, 1.93), I^2^ = 0%	RR 1.66 (1.33, 2.07), I^2^ = 0%
	Analysis 1.2	RR 0.94 (0.70, 1.27), I^2^ = 49%	N/A 1
	Analysis 1.3	RR 1.22 (0.73, 2.03), I^2^ = 30%	N/A 1
	Analysis 1.4 to Analysis 1.17	N/A 1	N/A 1
	Analysis 1.18	N/A 3	N/A 3
Nicotine EC versus varenicline	Analysis 2.1 to Analysis 2.2	N/A 2	N/A 1
Nicotine EC versus non‐nicotine EC + varenicline	Analysis 3.1 to Analysis 3.2	N/A 1	N/A 1
Nicotine EC versus NRT + bupropion	Analysis 4.1	N/A 1	N/A 1
Nicotine EC versus heated tobacco	Analysis 5.1 to Analysis 5.2	N/A 2	N/A 2
	Analysis 5.3	MD ‐0.20 (‐3.23, 2.83), I^2^ not estimable	MD ‐0.20 (‐3.23, 2.83), I^2^ not estimable
	Analysis 5.4	N/A 2	N/A 2
Nicotine EC versus oral nicotine pouches	Analysis 6.1 to Analysis 6.2	N/A 2	N/A 1
Nicotine EC versus non‐nicotine EC	Analysis 7.1	RR 1.41 (1.09, 1.82), I^2^ = 0%	RR 1.29 (1.01, 1.65), I^2^ = 0%
	Analysis 7.2	N/A 1	RR 1.01 (0.95, 1.07), I^2^ = 0%
	Analysis 7.3	RR 0.98 (0.55, 1.73), I^2^ = 0%	RR 0.94 (0.53, 1.67), I^2^ = 0%
	Analysis 7.4	N/A 3	N/A 3
	Analysis 7.5	N/A 1	MD ‐0.60 (‐3.52, 2.32), I^2^ not estimable
	Analysis 7.6	N/A 1	MD 3.11 (‐0.46, 6.68), I^2^ not estimable
	Analysis 7.7	N/A 1	N/A 1
	Analysis 7.8	N/A 1	N/A 2
	Analysis 7.9	N/A 3	N/A 3
	Analysis 7.10	N/A 1	MD 0.29 (‐1.73, 2.31), I^2^ not estimable
	Analysis 7.11 to Analysis 7.14	N/A 1	N/A 1
Nicotine EC versus behavioural support only/no support	Analysis 8.1	RR 4.73 (0.56, 39.88), I^2^ not estimable	RR 1.54 (1.31, 1.82), I^2^ = 0%
	Analysis 8.2	RR 1.29 (1.15, 1.45), I^2^ not estimable	RR 1.26 (0.90, 1.76), I^2^ = 68%
	Analysis 8.3	RR 0.71 (0.16, 3.10), I^2^ not estimable	RR 0.91 (0.66, 1.27), I^2^ = 0%
	Analysis 8.4	N/A 3	N/A 3
	Analysis 8.5	N/A 1	N/A 1
	Analysis 8.6	MD 1.11 (‐25.99, 28.21), I² = 0%	N/A 1
	Analysis 8.7	N/A 1	N/A 1
	Analysis 8.8	SMD ‐0.30 (‐0.74, 0.13), I^2^ not estimable	SMD ‐0.30 (‐0.74, 0.13), I^2^ not estimable
	Analysis 8.9	N/A 3	N/A 3
	Analysis 8.10 to Analysis 8.14	N/A 1	N/A 1
	Analysis 8.15 to Analysis 8.16	N/A 2	N/A 2
	Analysis 8.17	N/A 2	MD ‐0.14 (‐0.28, 0.00), I^2^ not estimable
	Analysis 8.18 to Analysis 8.19	N/A 2	N/A 2
Higher versus lower nicotine content	Analysis 9.1	N/A 1	N/A 1
	Analysis 9.2	N/A 2	N/A 2
	Analysis 9.3	N/A 1	RR 1.51 (0.51, 4.42), I^2^ not estimable
	Analysis 9.4	MD ‐0.90 (‐1.70, ‐0.10), I² = 0%	MD ‐1.15 (‐2.05, ‐0.24), I² = 0%
	Analysis 9.5	N/A 1	MD 1.92 (‐0.89, 4.73), I^2^ not estimable
	Analysis 9.6	N/A 1	MD 1.13 (‐2.76, 5.02), I^2^ not estimable
	Analysis 9.7	N/A 1	N/A 2
	Analysis 9.8	N/A 1	MD 0.15 (‐0.04, 0.34), I^2^ not estimable
	Analysis 9.9	N/A 1	MD ‐0.12 (‐0.36, 0.12), I^2^ not estimable
	Analysis 9.10	N/A 1	MD 0.89 (‐1.56, 3.34), I^2^ not estimable
	Analysis 9.11 to Analysis 9.14	N/A 1	N/A 1
Choice of flavours vs. tobacco flavour only	Analysis 10.1 to Analysis 10.2	N/A 2	N/A 2
	Analysis 10.3 to Analysis 10.6	N/A 1	N/A 1
Tobacco vs. menthol flavour	Analysis 11.1 to Analysis 11.4	N/A 2	N/A 2
Refillable versus cartridge	Analysis 12.1	N/A 2	N/A 1
Nicotine salt EC versus free‐based nicotine EC	Analysis 13.1 to Analysis 13.2	N/A 1	N/A 2
Higher versus lower wattage	Analysis 14.1 to Analysis 14.3	N/A 2	N/A 1
Non‐nicotine EC versus behavioural support only/no support	Analysis 15.1	RR 2.86 (0.30, 27.10), I^2^ not estimable	N/A 1
	Analysis 15.2	N/A 1	N/A 1
	Analysis 15.3	RR 1.19 (0.33, 4.33), no change as the study at high risk of bias was not estimable	N/A 1
Non‐nicotine EC + NRT versus NRT	Analysis 16.1 to Analysis 16.3	N/A 2	N/A 1
Non‐nicotine EC versus NRT	Analysis 17.1 to Analysis 17.4	N/A 1	N/A 1
Advice to use e‐cigarettes compared to no advice to use e‐cigarettes	Analysis 18.1	RR 1.04 (0.89, 1.22), I^2^ not estimable	N/A 1
	Analysis 18.2 to Analysis 18.3	N/A 1	N/A 1
	Analysis 18.4	N/A 2	N/A 1
Nicotine EC + NRT versus non‐nicotine EC + NRT	Analysis 19.1	N/A 2	N/A 1
	Analysis 19.2	RR 1.25 (0.78, 1.99), I^2^ not estimable	RR 1.09 (0.90, 1.31), I^2^ not estimable
	Analysis 19.3	RR 0.59 (0.11, 3.34), I^2^ not estimable	RR 0.67 (0.37, 1.19), I^2^ not estimable
	Analysis 19.4	MD ‐9.10 (‐15.83, ‐2.37), I^2^ not estimable	MD ‐1.40 (‐4.26, 1.46), I^2^ not estimable
	Analysis 19.5 to Analysis 19.8	N/A 2	N/A 1
Nicotine EC + NRT versus NRT	Analysis 20.1	RR 3.85 (1.91, 7.74), I^2^ not estimable	N/A 1
	Analysis 20.2	N/A 3	N/A 3
	Analysis 20.3	N/A 2	N/A 1
Nicotine EC + varenicline vs. varenicline	Analysis 21.1 to Analysis 21.2	N/A 2	N/A 1
Nicotine EC + VLNC versus VLNC	Analysis 22.1 to Analysis 22.4	N/A 1	N/A 1

N/A 1 = no studies at high risk of bias/no industry‐funded studies N/A 2 = all studies at high risk of bias/industry‐funded N/A 3 = results not pooled EC: electronic cigarette(s); MD: mean difference; NRT: nicotine replacement therapy; RR: risk ratio; SMD: standardised mean difference; VLNC: very low nicotine content

Pulvers 2020 [254, 255, 256, 257, 258, 259, 260, 261, 262, 263, 264, 265, 266, 267] (pod device; high risk of bias) measured cessation at six months in the intervention group only, using self‐report. As they did not measure cessation at six months in the comparator group, we could not include these data in our meta‐analysis. At six months, 23 (24%) intervention participants were exclusively using EC and 10 (10.4%) reported using neither EC nor combustible cigarettes (creating a combined quit rate of 34.4% in the intervention arm at six months).

###### Data from other studies

Ten studies provided all participants with nicotine EC and assessed abstinence at six months or longer (Table; 2 refillable, 6 cartridges, 1 pod, 1 not specified). The highest proportion of quitters at six months was observed in Ely 2013 [268] (cartridge), in which all participants (n = 48) used EC and 18 used additional pharmacotherapy; 44% of participants were abstinent at six months. The lowest quit rates were seen in Caponnetto 2013b* (cig‐a‐like), where 14% of participants were abstinent at 12 months, and Price 2022 [269] (type not specified), where 5% of participants were abstinent at 12 months. In the former, participants were unmotivated to quit smoking and, in the latter, motivation was unclear and participants were recruited from a socially deprived area on the basis of receiving a free nicotine EC.

**3:** Summary of proportion of participants abstinent from smoking at 6+ months follow‐up: cohort studies of nicotine EC

**Study**	**Motivated or unmotivated to quit smoking?**	**% abstinent**				
**Cohort studies**		**6‐month**	**12‐month**	**18‐month**	**24‐month**	**Notes**
Adriaens 2014 [358]^a^	Unmotivated to quit	19.6% (10/51)	‐	‐	‐	Data from 8‐month follow‐up
Edwards 2023	"Willing to attempt to quit"	26.6% (8/30)	‐	‐	‐	‐
Caponnetto 2013b*	Unmotivated to quit	‐	14% (2/14)	‐	‐	‐
Caponnetto 2021*	Unmotivated to quit	35% (14/40)	‐	‐	‐	‐
Ely 2013^b^	Motivated to quit	44% (21/48)	‐	‐	‐	‐
Pacifici 2015 [359]	Unmotivated to quit	‐	53% (18/34)	‐	‐	‐
Polosa 2011*	Unmotivated to quit	23% (9/40)	‐	15% (6/40)	13% (5/40)	‐
Polosa 2014b*	Unmotivated to quit	36% (18/50)	‐	‐	‐	‐
Polosa 2015*	Not defined	42% (30/71)	41% (29/71)	‐	‐	‐
Price 2022	Not defined	‐	5% (42/871)	‐	‐	‐

^a^Technically an RCT but observational for purposes of EC analysis ^b^All participants (N = 48) used an EC, but 16 also used bupropion and 2 used varenicline.

##### Adverse events

###### Randomised controlled trials

Pooled data from five studies (none at high risk of bias, one reporting tobacco/vaping industry funding) showed that there is probably no difference in the number of participants experiencing adverse events when comparing nicotine EC to non‐nicotine EC (RR 1.01, 95% CI 0.95 to 1.08; I² = 0%; 840 participants; Figure); this is moderate‐certainty evidence, downgraded one level due to imprecision (fewer than 300 events overall). Removing the one study linked to industry funding had no effect on the interpretation of the result (Table). When comparing nicotine EC to behavioural support only or to no support, evidence suggests more people in the groups randomised to nicotine EC may experience adverse events (RR 1.22, 95% CI 0.96 to 1.55; I² = 66%; 8 studies, 2485 participants; Figure). As this involved unblinded comparisons with unequal levels of support, we judged all data contributing to this outcome to be at high risk of bias (very low‐certainty evidence downgraded two levels due to risk of bias and imprecision). Interpretation of the outcome was not sensitive to the inclusion of the one study with tobacco/vaping industry support (Walele 2018*).

**9:**
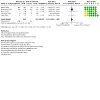
Analysis 7.2: Nicotine EC vs non‐nicotine EC ‐ Adverse events

**10:**
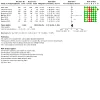
Analysis 8.2: EC vs behavioural support only/no support ‐ Adverse events

A further 11 RCTs provided AE or related data for this comparison, but could not be included in the meta‐analysis due to the way in which data were presented (see Supplementary material 11). In the studies comparing nicotine EC to non‐nicotine EC, one found similar event rates across arms (Caponnetto 2013a*; cig‐a‐like; unclear risk of bias), and two reported more events in the nicotine EC arms (Felicione 2019 [270]; Tseng 2016 [271, 272, 273, 274]; one cig‐a‐like and one refillable; unclear risk of bias). In a further study comparing nicotine to non‐nicotine EC, events were reported by type, with an increase in some seen in the nicotine group and an increase in others seen in the non‐nicotine group (Lucchiari 2022; cig‐a‐like; high risk of bias). In the seven studies comparing nicotine EC to behavioural support only or traditional cigarettes, Kumral 2016 [275] (device type not specified; high risk of bias) found an increase in sinonasal symptoms in the group receiving nicotine EC compared to behavioural support only, and Ozga‐Hess 2019 [276] (refillable; high risk of bias) found that throat irritation, cough, and dry mouth increased in the e‐cigarette group relative to the traditional cigarette group. By contrast, Pulvers 2020 (pod device; high risk of bias) found a reduction in respiratory symptoms in the EC group compared to the traditional cigarette group, and Pope 2024 [277, 278, 279, 280, 281, 282, 283, 284, 285, 286, 287] (pod device; high risk of bias) found no clear difference in the number of participants reporting dry cough and throat and mouth irritation between the EC arm and the referral information arm. Begh 2021 [288, 289, 290, 291, 292] (refillable; high risk of bias) found an increase in throat irritation, palpitations, and dizziness in the EC group, but decreases in cough, headache, nausea, dry mouth, shortness of breath, and stomach pain. Edmiston 2022* (cartridge; high risk of bias) did not break down AEs by group but reported that three participants experienced a non‐serious AE definitely related to the study product. Pratt 2022 [293, 294, 295, 296, 297, 298] (cartridge; high risk of bias) reported no statistically significant between‐group difference in AEs.

###### Data from other studies

Nineteen studies provided all participants with nicotine EC and assessed AEs at one week or longer. One RCT reported AEs reported in the nicotine EC group only (see Supplementary material 11). Of the eight studies that tracked event rates over time, six showed AEs reducing over time (Caponnetto 2013b*; Edwards 2023 [299, 300, 301]; Goniewicz 2017 [302]; Polosa 2011*; Polosa 2014b*; Pratt 2016 [303]). Hickling 2019 [304, 305] (cig‐a‐like; high risk of bias) showed no change. Sifat 2024 (pod device; high risk of bias) was a small study (n = 60) and reported no AEs. The most commonly reported AEs were throat/mouth irritation, headache, cough, and nausea.

##### Serious adverse events

###### Randomised controlled trials

Ten studies compared nicotine EC with non‐nicotine EC and reported data on SAEs; in five of these (including one tobacco/vaping industry study, Caponnetto 2013a*), no events occurred, so results could not contribute to the meta‐analysis, although they are included in the forest plots for descriptive purposes. In the five studies (four low risk of bias, one unclear) where events occurred, there may be little to no difference between groups, but CIs were wide (RR 0.98, 95% CI 0.55 to 1.73; I² = 0%; 1717 participants; Figure). The evidence was of low certainty; this was downgraded two levels due to imprecision: the confidence intervals encompassed clinically significant harm as well as clinically significant benefit, and there were fewer than 300 events overall. One of these studies had links to industry funding (Rose 2023*); removing it from the analysis changed the effect estimate to 0.94 but the 95% CI remained wide (0.53 to 1.67) and so the interpretation of the result remained the same.

**11:**
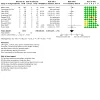
Analysis 7.3: Nicotine EC vs non‐nicotine EC ‐ Serious adverse events

Fifteen studies compared nicotine EC with behavioural support only or no support and reported data on SAEs; in eight of these, no events occurred. Pooled results from the seven studies in which events occurred showed very uncertain evidence about the difference between arms, and CIs were wide (RR 0.93, 95% CI 0.67 to 1.29; I² = 0%; 4716 participants; Figure). Here the certainty of evidence was very low; this was downgraded due to risk of bias (lack of blinding and differential support between arms, judged to be at high risk of bias) and imprecision (CI incorporated both clinically significant benefit and clinically significant harm). Removing the one study with tobacco/vaping industry support did not affect the interpretation of the results (Walele 2018*; Table).

**12:**
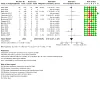
Analysis 8.3: EC vs behavioural support only/no support ‐ Serious adverse events

In a study in people experiencing homelessness (Dawkins 2020; refillable; high risk of bias), SAEs were not reported, but authors reported that four to seven participants in the usual care arm and five to seven participants in the nicotine EC arm visited Accident & Emergency services at a hospital. The authors reported that these visits were unrelated to study treatment and were assessed to gather data for future economic evaluation. Further detail can be found in Supplementary material 12.

###### Data from other studies

Ten studies provided all participants with nicotine EC and reported SAEs at a week or longer (Supplementary material 12). In seven of these, authors reported that no SAEs occurred (Caponnetto 2013b*; Caponnetto 2021*; Edwards 2023; Humair 2014 [306]; Kanobe 2022*; Polosa 2011*; Sifat 2024; Valentine 2018 [307]). In NCT02648178 [308] (cig‐a‐like and refillable; high risk of bias; 19 participants), one death occurred (no further detail provided). Hickling 2019 (cig‐a‐like; high risk of bias; 50 participants) recruited participants from mental health settings; five SAEs were recorded during the study, all of which were psychiatric hospitalisations. None were considered related to study treatment.

##### Carbon monoxide

###### Randomised controlled trials

High statistical heterogeneity (I^2^ = 80%) precluded pooling CO data from six trials (n = 677, none considered to be at high risk of bias) comparing nicotine EC with non‐nicotine EC (Analysis 7.4). Point estimates from four studies (one reporting links to industry funding; Rose 2023*) favoured nicotine EC and from two (one reporting industry funding; Caponnetto 2013a*) favoured non‐nicotine EC, but in all cases, CIs were consistent with no clinically meaningful difference. Three further randomised studies measured CO levels in those assigned to nicotine EC and those assigned to non‐nicotine EC, but did not present data in a way that could be pooled: George 2019 [309, 310] (cig‐a‐like; high risk of bias) did not compare data by group; Tseng 2016 (cig‐a‐like; unclear risk of bias) reported no between‐group differences but no analysable data; and Meier 2017 [311] (cig‐a‐like; unclear risk of bias) found a slightly higher CO reading in those using nicotine EC, but the clinical and statistical significance of this difference was not clear (see Supplementary material 13 for more detail).

Pooled data from 14 studies comparing nicotine EC with behavioural support or no support resulted in a high I^2^ value (93%); thus, pooled results are not presented here (see Analysis 8.4 for individual study data). None of these studies reported tobacco/vaping industry funding. The funnel plot did not show asymmetry (Figure). Heterogeneity was primarily driven by magnitude rather than direction of effect, with results in 13 of 14 studies favouring nicotine EC. Five further trials reported data that could not be included in the meta‐analysis. Of those studies comparing nicotine EC to combustible cigarettes, Walele 2018* (cig‐a‐like; high risk of bias) found that CO levels declined in the EC group and remained similar to baseline in the cigarette group, and George 2019 (cig‐a‐like; high risk of bias) reported that the lowest tertile of CO at end of study was amongst those with the best compliance with EC and least dual use. Czoli 2019 (high risk of bias) instructed baseline dual users to spend periods only using EC or only using traditional cigarettes; CO measured during sole EC use was lower than baseline and lower than during cigarette‐only periods. Further details can be found in Supplementary material 13. NCT03113136 reported reduced CO levels in dual users of combustible tobacco and EC compared with exclusive combustible tobacco users.

**13:**
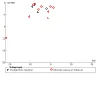
Funnel plot. Comparison: Nicotine EC vs behavioural/no support. Outcome: Carbon monoxide (ppm)

###### Data from other studies

Twenty‐three studies provided all participants with nicotine EC and reported data on CO at one week or longer. In the 21 studies that presented change over time, all but Hoeppner 2024 (refillable; high risk of bias) reported a decline in CO from baseline, although in Ikonomidis 2018 [312, 313] (device type not specified; unclear risk of bias), CO levels were equivalent to baseline again at 24 weeks, and in Polosa 2014b* (refillable; high risk of bias), a decline was observed in people who quit smoking or reduced cigarette consumption by at least half, but not in those who continued smoking at least half as many cigarettes as they had from baseline. Hoeppner 2024 reported a mean increase of 5.1 ppm at three months from baseline.

##### Heart rate

###### Randomised controlled trials

Two trials (Caponnetto 2013a*, Cobb 2021, n = 401; one cig‐a‐like, one cartridge; one industry funded, neither at high risk of bias) provided data on heart rate and compared nicotine EC with non‐nicotine EC. While the effect estimate indicated lower mean heart rate in the nicotine EC arm, the CI included the potential for no clinically significant between‐group difference (MD ‐1.23, 95% CI ‐3.55 to 1.08; I² = 0%; 401 participants). Removing the study with industry support did not affect the interpretation of the results (Table). One RCT (Hatsukami 2020, cig‐a‐like; unclear risk of bias, n = 90) compared nicotine EC with no pharmacotherapy and found no evidence of a clinically significant difference (MD 1.17, 95% CI ‐4.27 to 6.61).

A further two RCTs provided data on heart rate that could not be used to calculate effect estimates. George 2019 (cig‐a‐like; high risk of bias) compared nicotine to non‐nicotine EC and reported no difference in heart rate between arms; Walele 2018* (cig‐a‐like; high risk of bias) compared nicotine EC with traditional cigarettes and reported "no clinically significant changes". See Supplementary material 14 for further information.

###### Data from other studies

Six studies in which all participants received a nicotine EC also reported data on heart rate; for five, changes were minimal and directions of effect were mixed, and for Caponnetto 2021* (n = 40; pod device; high risk of bias), the rate reduced by 9 bpm at 12 weeks (see Supplementary material 14).

##### Blood pressure

While the pooled effect estimate from two trials (Caponnetto 2013a*; Cobb 2021, n = 401; one cig‐a‐like, one cartridge; one industry funded, neither at high risk of bias) indicated higher systolic blood pressure (BP) in the nicotine EC arm, the CI included the potential for no clinically significant difference in BP between nicotine EC and non‐nicotine EC arms (MD 2.50, 95% CI ‐0.45 to 5.44; I² = 0%). Removing the study with industry support did not affect the interpretation of the results (Table). Three studies (one at high risk of bias, two at unclear risk of bias, none reporting tobacco/vaping industry funding) compared nicotine EC to behavioural support only and reported data on systolic BP; there was a small difference favouring the EC arms, but the CI included the potential for no clinically significant difference (MD ‐1.64, 95% CI ‐7.97 to 4.70; I² = 23%; 298 participants). Removing the high risk of bias study changed the direction of effect, but the CI still included the potential for no clinically significant difference (Table). Three further RCTs measured change in blood pressure but presented results in a way that could not be pooled. George 2019 (cig‐a‐like; high risk of bias) compared nicotine EC and non‐nicotine EC and combined data from both groups; BP declined over time. Walele 2018* (cig‐a‐like; high risk of bias) found "no clinically significant changes" when comparing nicotine EC to a conventional cigarette at two weeks. Katz 2025 (pod device; unclear risk of bias) assigned participants to nicotine EC and combustible cigarettes in consecutive two‐week phases and reported lower diastolic BP after the EC phase than after the combustible cigarette phase.

Six studies that provided nicotine EC to all participants reported changes in BP; results were clinically insignificant except for Caponnetto 2021* (pod device; high risk of bias) in which systolic BP reduced by 12 mmHg (from 134 to 122) at 12 weeks (see Supplementary material 15 for further details on all studies reporting this outcome).

##### Oxygen saturation

Hatsukami 2020 (cig‐a‐like; unclear risk of bias) found no evidence of a difference in blood oxygen saturation when comparing nicotine EC to cigarettes (MD 0.20, 95% CI ‐0.30 to 0.70; 89 participants). Van Staden 2013* (high risk of bias), a short‐term pre‐post study, which measured outcomes after two weeks of EC use, found that people who smoked and switched to EC had significant improvement in blood oxygen saturation (96.2% (standard deviation (SD) 1.8) to 97.5% (SD 1.3); 1.3% increase, 95% CI 0.6 to 2.1; P = 0.002).

##### Toxicants

Unless stated otherwise, all RCTs measuring these outcomes compared nicotine EC with no pharmacotherapy.

Two trials measuring change in 3‐HPMA (one at high risk of bias) reported decreased measures in the EC arm (SMD ‐0.46, 95% CI ‐0.66 to ‐0.26; I² = 0%; 474 participants). Removing Walele 2018* (cig‐a‐like; both at high risk of bias and industry funded) did not affect the interpretation of the results (Table). Five further studies, in which all participants were given nicotine EC, measured 3‐HPMA; all found reductions over time (Supplementary material 16).

Five trials measured change in NNAL and provided sufficient data to calculate summary effects (four at high risk of bias, two industry funded; Analysis 8.9). Three of the five studies found results favouring nicotine EC, but the final two indicated no difference; statistical heterogeneity was high (I^2^ = 96%), so pooled results were not presented. One study comparing nicotine EC to no treatment described their findings narratively and stated that *"NNAL decreased more over time in the e‐cigarette group ... the e‐cigarette group had significantly lower NNAL at 4 weeks (estimate = 0.54; SE = 0.23; t = 2.37; P < 0.02), but the group difference was attenuated at 8 weeks (estimate = 0.42; SE = 0.23; t = 1.83; P < 0.07)"* (Pratt 2022; cartridge; high risk of bias). Pulvers 2018 [314] (refillable; high risk of bias) and Morris 2022* (pod device; high risk of bias), which provided all participants with nicotine EC, found a reduction in NNAL over time and Czoli 2019 (choice of device; high risk of bias), which was a cross‐over trial, found NNAL decreased when using nicotine EC compared to using traditional cigarettes (Supplementary material 16). An additional two RCTs (one refillable and one cartridge; one unclear and one low risk of bias; none reporting tobacco/vaping industry funding) compared nicotine EC to non‐nicotine EC and found a mean difference of 4.13 pmol/mg creatinine, with wide CI and moderate statistical heterogeneity (MD 4.13, 95% CI ‐9.21 to 17.48; I² = 54%; 363 participants).

One trial (Hatsukami 2020; n = 90, cig‐a‐like; unclear risk of bias) found non‐statistically significant lower levels of 2‐HPMA, HMPMA, PhET, and AAMA in nicotine EC arms compared to control (Analysis 8.10; Analysis 8.11; Analysis 8.12; Analysis 8.14). A further two studies, in which all participants received nicotine EC, found reductions in 2‐HPMA and AAMA measures over time (Supplementary material 16). Hatsukami 2020 found no difference in CEMA (Analysis 8.13).

One trial (Walele 2018*; cig‐a‐like; high risk of bias) found reductions in S‐PMA compared to control (MD ‐1371.00, 95% CI ‐1995.23 to ‐746.77; 384 participants); this was consistent with the two studies in which all participants received nicotine EC that measured S‐PMA, where levels declined over time (Supplementary material 16).

Of the 33 remaining measurements in single studies where all participants received a nicotine EC, 28 were reduced over time and five increased (Supplementary material 16).

##### Lung function

In Caponnetto 2013a* (cig‐a‐like; unclear risk of bias), FeNO increased more in the nicotine EC than the non‐nicotine EC group (MD 2.35, 95% CI 1.78 to 2.92; 90 participants). Two trials measured FEV1 and FEV1/FVC (Caponnetto 2013a*; Cobb 2021; one cig‐a‐like, one cartridge; unclear and low risk of bias respectively). Heterogeneity precluded pooling of FEV1 measurements (I^2^ = 78% and studies found different directions of effect), but individual results are presented in Analysis 7.9. No difference was found between nicotine and non‐nicotine EC for FEV1/FVC (Analysis 7.10), and sensitivity analysis, removing Caponnetto 2013a*, did not change this. Cobb 2021 also measured FVC, PEF (peak expiratory flow) and FEF (forced expiratory flow 25‐75) and found no evidence of difference between nicotine and non‐nicotine EC (Analysis 7.11; Analysis 7.12; Analysis 7.13).

In the comparison of nicotine EC to behavioural support only/no support, pooled results from two studies (one cig‐a‐like and one cartridge; both high risk of bias, both tobacco/vaping industry funded) found improvements in FEV1 but with moderate statistical heterogeneity and CI including no difference (SMD 0.15, 95% CI ‐0.14 to 0.44; I² = 70%; 714 participants). Pooled data from two studies (one cig‐a‐like and one cartridge; both high risk of bias, one reporting tobacco/vaping industry funding (Walele 2018*)) showed no difference in FEF 25‐75, with substantial levels of statistical heterogeneity (MD ‐0.03, 95% CI ‐0.27 to 0.20; I² = 73%; 2 studies, 555 participants). In a sensitivity analysis removing Walele 2018*, the result was still consistent with no difference, though the point estimate was greater in magnitude. Data from one study at high risk of bias showed no difference in PEF (peak expiratory flow 25‐75 (litres/minute)) (MD ‐7.10, 95% CI ‐29.14 to 14.94; 387 participants). The one study reporting FEV1/FVC (Edmiston 2022*; high risk of bias) favoured nicotine EC (MD 1.72, 95% CI 0.74 to 2.70; 327 participants).

Katz 2025 (pod device; unclear risk of bias), which randomised participants to nicotine EC or continued combustible cigarettes, measured changes in objective (spirometry, oscillometry) and self‐reported (CAT, SGRQ‐C) lung function and reported no significant differences (Supplementary material 17).

Two studies, which provided all participants with nicotine EC, measured change in lung function over time: Hickling 2019 (cig‐a‐like; high risk of bias) found an increase in peak flow, and Oncken 2015 [315, 316, 317] (cig‐a‐like; unclear risk of bias) found "no significant differences" in airway function (Supplementary material 17).

##### Study product use

Three trials (two cig‐a‐like and one cartridge; all low risk of bias; none industry funded), comparing nicotine EC with non‐nicotine EC, reported the number of participants still using EC at six months or longer. Slightly more participants were still using EC in the nicotine EC arms, but CI were wide and included no difference (RR 1.14, 95% CI 0.77 to 1.69; I² = 30%; 874 participants). Data on this outcome from single‐arm studies or RCTs, where a study product (i.e. EC) was only provided in one arm, can be found in a companion publication (up to date to November 2021) [246] and Supplementary material 18.

#### Direct comparisons between nicotine EC

Studies reported in this section are only those where participants were randomised to different nicotine EC conditions.

##### Comparisons based on nicotine dose

Six trials provided data comparing different doses of nicotine in EC (although other studies provided a range of doses, these were not randomly assigned). Only one study provided data on abstinence; in Cobb 2021 (cartridge; low risk of bias), quit rates were higher in the higher‐dose arm but the 95% CI included no difference (RR 2.50, 95% CI 0.80 to 7.77; 260 participants).

Four studies (all at high risk of bias) provided data on AEs, three of which provided data in such a way that the studies could not be pooled. Kimber 2021 (cartridge and refillable; high risk of bias) reported "no changes over time or differences between condition", and Pratt 2022 (cartridge; high risk of bias) and Morris 2022* (pod device; high risk of bias) did not compare AEs by nicotine strength (see Supplementary material 11). Kanobe 2022* (cig‐a‐like and cartridge; high risk of bias) found slightly more participants in the lower‐dose group reported AEs; however, 95% CI incorporated the null and also the possibility that more people experienced AEs in the higher‐dose arm (RR 0.90, 95% CI 0.58 to 1.40; 68 participants).

In Caponnetto 2013a* (cig‐a‐like; unclear risk of bias), no SAEs were reported in either arm; in Cobb 2021 (cartridge; low risk of bias), there were more events in the higher‐dose arm but CIs were wide (RR 1.51, 95% CI 0.51 to 4.42; 239 participants). In Morris 2022* (pod device; high risk of bias), no SAEs occurred (Supplementary material 12).

Point estimates favoured higher‐dose EC and CI excluded no difference for CO and FEV1/FVC (MD ‐0.92, 95% CI ‐1.71 to ‐0.13; I² = 0%; 3 studies, 348 participants; one high risk of bias study), (MD 0.91, 95% CI 0.18 to 1.64; I² = 0%; 2 studies, 350 participants; no high risk of bias studies). Interpretation of Analysis 9.4 did not change when excluding the one study at high risk of bias (Kimber 2021), or the one study with tobacco/vaping industry funding (Caponnetto 2013a*); excluding the same study from Analysis 9.10 maintained the same direction of effect, but the CI widened to cross the null. There were no clear differences between arms for heart rate, BP, other lung function measures, or NNAL (Analysis 9.5; Analysis 9.6; Analysis 9.7; Analysis 9.8; Analysis 9.9; Analysis 9.13; all included Caponnetto 2013a* (cig‐a‐like; unclear risk of bias) and Cobb 2021 (cartridge; low risk of bias), except for Analysis 9.7 which included Caponnetto 2013a* alone and Analysis 9.13, which included Cobb 2021 alone). More participants in the higher‐dose nicotine group were still using EC at six months or longer, but data were from one study and CI were wide and included no difference (RR 1.27, 95% CI 0.95 to 1.68; 260 participants). In Yingst 2020 (cross‐over, comparing different doses and different devices; cig‐a‐like; refillable; unclear risk of bias), exhaled CO and reported nausea did not differ between devices; self‐reported dizziness was low overall but slightly higher in the higher‐dose arm. Further details can be found in Supplementary material 11 and Supplementary material 13. Morris 2022* (pod device; high risk of bias) measured a range of toxicants but did not compare these based on nicotine level assignments (Supplementary material 16).

One further study, White 2022 (refillable; high risk of bias), also included comparisons based on nicotine levels (1.8% free‐base nicotine, designated by the authors as 'moderate', and 0.3% free‐base nicotine, designated by the authors as 'low'). This was a factorial trial which, in addition to EC liquid nicotine content, also manipulated cigarette nicotine content and EC liquid flavour availability. The authors reported no significant main effects for nicotine content on CO or CEMA, and no statistically significant interactions for these conditions. There also appear to have been no differences in the proportions of people experiencing AEs, but the study terminated early and was likely underpowered to detect differences.

##### Comparisons based on flavour

One study randomised participants to different flavour conditions (1. tobacco flavour only; 2. a choice of flavours) and followed up participants for six months or longer (Xu 2023*, n = 566, industry‐funded, high risk of bias, pod EC). Quit rates were lower in the choice compared to the tobacco arm, but the CIs were wide and incorporated no difference and a clinically significant increase relative to tobacco flavour (choice versus tobacco, RR 0.80, 95% CI 0.54 to 1.16; 566 participants). Xu 2023* also reported on product use at six months or longer; again, there was no clear evidence of a difference, but CIs were wide (choice versus tobacco, RR 1.10, 95% CI 0.86 to 1.40).

Higgins 2024 (n = 146, pod device; unclear risk of bias) randomised participants to either tobacco‐flavoured EC or choice of flavour, alongside very low nicotine content (VLNC) combustible cigarettes. This study did not find evidence of difference between groups in AEs (RR 1.01, 95% CI 0.88 to 1.15; 158 participants), SAEs (RR 0.44, 95% CI 0.08 to 2.34; 158 participants), CO (MD ‐4.52, 95% CI ‐11.06 to 2.02; 124 participants) or NNAL (MD ‐0.09, 95% CI ‐1.26 to 1.08; 100 participants).

One study (Edmiston 2022*, n = 300, cartridge; high risk of bias, vaping/tobacco industry funding) randomised participants to different flavours (tobacco versus menthol) and provided SAE data in a way that could have been used to compute risk ratios, although no SAEs occurred in either arm (Analysis 11.1). NNAL, FEV1/FVC, and FEV1 were lower in the tobacco flavour group, but CIs were wide and included no difference (MD ‐26.10, 95% CI ‐66.73 to 14.53; 232 participants; MD ‐0.46, 95% CI ‐1.67 to 0.75; 212 participants; MD ‐0.67, 95% CI ‐2.34 to 1.00; 212 participants). No other outcomes from this paper were eligible for inclusion in our review.

Morris 2022* (pod device; high risk of bias), an industry‐funded, randomised, cross‐over trial, tested the effect of 10 different flavours (as well as nicotine strengths and salt versus free‐base nicotine). Only their data on AE and SAE were eligible for inclusion in our review, but analyses were not reported by flavour (Supplementary material 11; Supplementary material 12).

White 2022 (refillable; high risk of bias) also contributed data to this comparison, with conditions being tobacco flavours only, or tobacco, fruit, dessert, and mint flavours. No significant main effects or interactions were found for flavours on the outcomes relevant to this review, namely CO and CEMA, and no difference was discernable in the occurrence of AEs. However, as noted above, the study terminated early and hence was underpowered to detect differences.

More information on flavour choices from the studies in this review can be found in a companion publication [318, 319].

##### Comparisons based on device type

Kimber 2021 (high risk of bias) is the only study to directly compare device types (cartridge versus refillable). Outcomes eligible for this review were CO and AE. There was no difference between arms for CO, but CIs were wide (MD 0.70, 95% CI ‐4.98 to 6.38; 32 participants). The authors reported *"no changes over time or differences between condition"* for AEs (see Supplementary material 11).

##### Nicotine salt versus free‐based nicotine

One study (Russell 2021*, pod device; unclear risk of bias, tobacco/vaping industry funding) contributed data to this comparison. Quit rates and study product use were both similar between arms (RR 1.25, 95% CI 0.85 to 1.83; 285 participants; and RR 1.07, 95% CI 0.82 to 1.41; 227 participants, respectively).

As described above, Morris 2022* (pod device; high risk of bias) also tested salt versus free‐based nicotine, but did not provide data broken down by these characteristics for our outcomes of interest (Supplementary material 11; Supplementary material 12).

##### Higher versus lower wattage EC

One study (NCT03113136, high risk of bias, n = 267; device type not specified) compared higher wattage EC with lower wattage EC. Quit rates were similar between study arms, and the CI included the potential for benefit from either (RR 0.72, 95% CI 0.30 to 1.74; 267 participants). The study did not find evidence of a difference in rates of AEs (RR 0.92, 95% CI 0.79 to 1.06; 267 participants) or SAEs (RR 0.99, 95% CI 0.14 to 6.94; 267 participants).

#### Non‐nicotine EC

Although non‐nicotine EC served as a 'control group' in our primary analysis, due to its behavioural properties, it can also be considered an intervention. Comparisons included here are: non‐nicotine EC versus NRT; non‐nicotine EC versus behavioural support/no treatment; and non‐nicotine EC as an adjunct to NRT. All contributing data were from RCTs. None of these studies reported data on change in heart rate, BP, oxygen saturation, toxicants, or lung function.

##### Cessation

When comparing non‐nicotine EC to behavioural or no support, pooled results from two studies (n = 388; both cig‐a‐like; one at high risk of bias, neither reporting tobacco/vaping industry funding) found higher quit rates in participants randomised to non‐nicotine EC, but the CI included the possibility of no difference (RR 1.59, 95% CI 0.80 to 3.19; I² = 0%; 2 studies, 388 participants). When evaluating non‐nicotine EC as an adjunct to NRT, Walker 2020 [320, 321, 322] (refillable; high risk of bias) also found higher quit rates in participants randomised to non‐nicotine EC, although again the CI included no difference (RR 1.67, 95% CI 0.50 to 5.53; 624 participants).

Two studies (n = 314, refillable; neither at high risk of bias, neither reporting tobacco/vaping industry funding) compared non‐nicotine EC with NRT (Klonizakis 2022; Lee 2019). The pooled estimate showed no clear evidence of a difference in quit rates between the two interventions (RR 0.99, 95% CI 0.64 to 1.54; I² = 36%; 314 participants).

##### Adverse events

Eisenberg 2020 (cig‐a‐like; low risk of bias) found a higher rate of adverse events in the non‐nicotine EC arm than in behavioural support only, with CI excluding no difference (RR 1.28, 95% CI 1.13 to 1.44; 248 participants). Also comparing non‐nicotine EC to behavioural support, Lucchiari 2022 (cig‐a‐like; high risk of bias) reported that some AEs were lower in the non‐nicotine EC arm, some higher, and others were reported at similar rates to the behavioural support arm (overall AE rates were not reported) (Supplementary material 11).

Walker 2020 (refillable; high risk of bias) found fewer AEs in participants receiving non‐nicotine EC + NRT compared to NRT alone, with the CI excluding no difference (RR 0.70, 95% CI 0.53 to 0.91; 344 participants). Lee 2019 (refillable; low risk of bias) also found that fewer participants receiving non‐nicotine EC reported AEs than those receiving NRT, with the CI excluding no difference (RR 0.33, 95% CI 0.12 to 0.87; 132 participants).

##### Serious adverse events

Two studies reported on rates of SAEs when comparing non‐nicotine EC with behavioural support. Lucchiari 2022 (cig‐a‐like; high risk of bias) reported no SAEs in either arm (RR not estimable), whereas Eisenberg 2020 (cig‐a‐like; low risk of bias) found a higher rate of SAEs in the non‐nicotine EC arm than in the behavioural support‐only arm. However, the CI was wide and incorporated clinically significant benefit and clinically significant harm (RR 1.19, 95% CI 0.33 to 4.33; 388 participants). In Walker 2020 (refillable; high risk of bias), more SAEs occurred in the group randomised to non‐nicotine EC + NRT than in the NRT‐alone group, but the CI included no difference as well as the potential for a clinically significant difference in favour of the intervention (RR 1.69, 95% CI 0.60 to 4.74; 624 participants). No SAEs were reported in either arm of Lee 2019 (refillable; low risk of bias; non‐nicotine EC versus NRT) (Analysis 17.3).

##### Carbon monoxide

One study investigating the comparison between non‐nicotine EC and NRT reported change in CO between baseline and six‐month follow‐up (Klonizakis 2022; refillable; unclear risk of bias). The point estimate favoured NRT; however, the CI encompassed both benefit and harm of the intervention (MD 2.00, 95% CI ‐0.50 to 4.50; 164 participants).

#### Advice to use EC to stop smoking

Three studies did not provide EC, but instead provided participants with advice on how to use EC to stop smoking; none reported tobacco/vaping industry funding. Czoli 2019 (high risk of bias) and Vickerman 2022 (unclear risk of bias) were short‐term studies and contributed data to Supplementary material 13 and Supplementary material 16 only. However, Martinez 2021 (low risk of bias) and Elling 2023 (high risk of bias) provided sufficient data from long‐term follow‐up to include these studies in meta‐analysis. In both cases, people received self‐help smoking cessation interventions with information on how to use EC to stop smoking compared to a smoking cessation intervention without the recommendation to use EC. However, Martinez 2021 specifically recruited people using both combustible cigarettes and EC (dual users) at baseline and Elling 2023 only required participants to be combustible cigarette users at baseline. Pooled smoking cessation rates provided no clear evidence of a difference between the two types of intervention provided (RR 1.02, 95% CI 0.88 to 1.19; I² = 0%; 2 studies, 2652 participants). In Vickerman 2022, more AEs occurred in the group receiving advice to use EC to stop smoking; however, the CI included no difference (RR 1.27, 95% CI 0.72 to 2.26; 52 participants). No SAEs were reported, so RRs were not estimable (Analysis 18.3). Elling 2023 and Martinez 2021 also reported on EC use at six‐month follow‐up. Data from Elling 2023 suggested higher rates of long‐term EC use in the EC advice arm; however, the 95% CI also encompassed the possibility of lower long‐term EC use in the intervention arm (RR 1.77, 95% CI 0.83 to 3.79; 331 participants). Martinez 2021 reported that 64% in the targeted booklet arm, and 66% in the generic booklet arm were still using EC. The latter data could not be incorporated into a meta‐analysis due to uncertainty about the denominator used to calculate percentages.

#### EC as an adjunct to other interventions

##### Nicotine EC and NRT

This section covers two comparisons: studies in which all arms received NRT and participants were randomised to nicotine EC or non‐nicotine EC, and studies in which all participants received NRT and one arm was randomised to nicotine EC, in addition. All studies contributing data were RCTs. No studies in this group reported data on heart rate, BP, oxygen, or toxicants.

###### Cessation

Two trials (both at high risk of bias, both testing refillable devices, neither reporting tobacco/vaping industry funding) in which all participants received NRT compared nicotine EC to non‐nicotine EC. The pooled results favoured nicotine EC, with the CI excluding no difference (RR 1.77, 95% CI 1.07 to 2.94; I² = 0%; 1039 participants).

Three studies (two high risk of bias, one unclear risk; two refillable, one cartridge; none reporting tobacco/vaping industry funding) also compared nicotine EC + NRT to NRT alone. Pooling results from all three studies resulted in high statistical heterogeneity, precluding meta‐analysis (I^2^ = 83%). This heterogeneity was driven by one study (Morphett 2022a [323, 324, 325]; high risk of bias). This study tested a cartridge device, and historically, cartridge devices have had poorer nicotine delivery than refillables. Once this study was removed, heterogeneity disappeared (I^2^ = 0%), but only two studies remained. In these two studies, pooled results showed more people quit in the refillable nicotine EC + NRT arm than in the NRT alone arm (RR 3.57, 95% CI 1.96 to 6.51; I² = 0%; 980 participants). In two of these studies, participants in both groups received nicotine patches but, in Morphett 2022b [326, 327] (refillable; unclear risk of bias), participants in the NRT‐only arm also received a short‐acting form of NRT.

###### Adverse events

Three trials in which nicotine EC were compared to non‐nicotine EC (both with NRT as an adjunct) reported data on AEs. Baldassarri 2018 [328, 329, 330] (refillable; high risk of bias) reported results combined across groups but noted *"no significant differences by treatment group"* (Supplementary material 11). Pooled data from the other two studies (one refillable and one pod; one at high risk of bias; one reporting tobacco/vaping industry funding: Rose 2023*) also showed no clear evidence of difference (RR 1.11, 95% CI 0.93 to 1.32; I² = 0%; 677 participants). Sensitivity analyses, removing the study at high risk of bias (Walker 2020) and removing the industry‐funded study (Rose 2023*), did not affect the interpretation of this result (Table).

The four trials comparing nicotine EC + NRT to NRT alone contributing data to this outcome were all at high risk of bias; none reported tobacco/vaping industry funding. Pooling results from all three studies resulted in high statistical heterogeneity, precluding meta‐analysis (I^2^ = 78%). Individual findings are presented in Analysis 20.2.

###### Serious adverse events

Pooled data from two studies (one refillable and one pod; one high risk, one unclear; one reporting tobacco/vaping industry funding), comparing nicotine EC with non‐nicotine EC as adjuncts to NRT, showed fewer SAEs in the nicotine EC group than in the non‐nicotine EC group, but the CI included no difference (RR 0.66, 95% CI 0.38 to 1.14; I² = 0%; 1069 participants). Removing the study with industry funding (Rose 2023*) had no effect on interpretation.

Five studies (three refillable, one cartridge and one pod; all high risk of bias; none reporting tobacco/vaping industry funding) provided data on SAEs and compared nicotine EC + NRT to NRT alone. The pooled estimate favoured the NRT‐alone group, but only two studies reported events and the CI was wide and included no difference (RR 1.24, 95% CI 0.45 to 3.41; I² = 0%; 2352 participants).

###### Carbon monoxide

Walker 2020 (refillable; high risk of bias; comparing nicotine EC + NRT, non‐nicotine EC + NRT, and NRT alone) measured change in CO levels but did not report data in a way that could be pooled. CO declined over time, with the greatest reduction seen in the nicotine EC group (see Supplementary material 13). Pooled data from two studies (one refillable and one pod; one high risk of bias, one unclear; one reporting tobacco/vaping industry funding), comparing nicotine and non‐nicotine EC as adjuncts to NRT, found a greater reduction in CO in the nicotine EC group. However, the CI included the potential for greater reduction from the non‐nicotine EC arm (MD ‐4.62, 95% CI ‐12.07 to 2.82; I² = 77%; 70 participants) between groups and there was substantial statistical heterogeneity. We have pooled these studies despite the high I^2^ as the individual study effects both showed a benefit of nicotine EC, with the difference being in the magnitude of effect. Removing the study at high risk of bias (Baldassarri 2018; refillable) left only Rose 2023* (pod device), with the following effect estimate: MD ‐9.10, 95% CI ‐15.83 to ‐2.37; whereas removing the study with industry funding (Rose 2023*) left only Baldassarri 2018, with the following effect estimate (MD ‐1.40, 95% CI ‐4.26 to 1.46).

###### Lung function

Baldassarri 2018 (refillable; high risk of bias), compared nicotine EC to non‐nicotine EC, with both groups receiving NRT. They found no between‐group differences in FeNO, FEV1, or FVC (Analysis 19.5; Analysis 19.6; Analysis 19.7); CIs were wide for all outcomes.

###### Study product use

In Walker 2020 (refillable; high risk of bias), at six months, 40% of the patches‐only arm (n = 52) were still using patches and in the patches + nicotine EC group (n = 317), 22% were using patches only, 45% were using EC only, and 11% were using both patch and EC. In the patches + non‐nicotine EC group (n = 308), 29% were still using patches, 36% were using EC only, and 13% were using both patches and EC. In Baldassarri 2018 (refillable; high risk of bias), there was no difference between arms in product use, but only nine participants contributed data (RR 1.25, 95% CI 0.29 to 5.35; 9 participants).

##### Nicotine EC and varenicline

One study, Tattan‐Birch 2023 [331, 332] (refillable; high risk of bias, 92 participants), evaluated nicotine EC and varenicline compared to varenicline alone. The study terminated early due to varenicline supply issues (an international recall), and the only data eligible for inclusion in this review related to AEs and SAEs. There was no evidence of a difference in AEs, though the CI was wide (RR 1.18, 95% CI 0.84 to 1.67; 92 participants). No SAEs occurred (Analysis 21.2).

##### Nicotine EC and very low nicotine content cigarettes

Higgins 2024 (pod device; unclear risk of bias, 243 participants) tested nicotine EC as an adjunct to very low nicotine content (VLNC) combustible cigarettes compared with VLNC alone. There was no evidence of a difference in AEs (RR 0.97, 95% CI 0.88 to 1.07; 243 participants) or SAEs (RR 0.81, 95% CI 0.23 to 2.78; 243 participants). This study reported four SAEs that were "related, probably related or possibly related" to the study arm intervention. One event occurred in the EC plus VLNC arm (hypertension), and the three events in the normal nicotine combustible cigarettes arm were OASIS score increase, irritability, and hypertension. While there was decreased CO in the nicotine EC plus VLNC arm compared with VLNC alone (MD ‐7.15, 95% CI ‐13.07 to ‐1.23; 132 participants), there was no evidence of difference in NNAL between study arms (MD 0.29, 95% CI ‐1.83 to 2.41; 110 participants).

#### Reporting biases

We were able to create two funnel plots. One for EC versus behavioural/no support showed that smoking cessation showed some evidence of asymmetry (Analysis 8.1; Figure). A funnel plot for nicotine EC versus behavioural or no support showed that carbon monoxide did not show evidence of asymmetry (Analysis 8.4; Figure).

**14:**
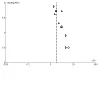
Funnel plot. Comparison: Nicotine EC vs behavioural/no support. Outcome: smoking cessation

## Discussion

### Summary of main results

This update includes a further fourteen studies published since the last version (January 2025) of this review. Our three main comparisons, nicotine EC compared to NRT, nicotine EC compared to non‐nicotine EC, and nicotine EC compared to behavioural support only/no support, continue to show increased quit rates in people assigned to nicotine EC arms. This conclusion has high certainty for the comparison with NRT, moderate certainty for the comparison with non‐nicotine EC, and low certainty for the comparison with behavioural support only/no support (Table; Table; Table). In absolute terms, pooled data suggest an additional two to five people for every 100 users of the intervention would quit smoking with nicotine EC compared to NRT, an additional zero to four people for every 100 would quit smoking with nicotine EC compared to non‐nicotine EC, and an additional two to five people for every 100 would quit smoking with nicotine EC compared to behavioural support only or no support for smoking cessation. Most data come from studies of cartridges and refillable devices, although the number of studies investigating pod devices is increasing, with the two new included studies providing pod devices.

There remains moderate certainty of no evidence of a difference in rates of adverse events (AEs) with nicotine EC compared to non‐nicotine EC, and moderate certainty of no evidence of a difference in rates of AEs with nicotine EC compared to NRT. Evidence on AEs and SAEs was of low to very low certainty across all other comparisons, due to a paucity of data. Many of the studies that measured SAEs reported no such events in either study arm. For nicotine EC compared to non‐nicotine EC, pooled data suggest no evidence of a difference in the number of people experiencing AEs or SAEs. Conversely, data from comparisons between nicotine EC and behavioural support alone or no support suggest an additional 11 people per 100 assigned to nicotine EC may experience AEs (compared with 50 per hundred receiving behavioural or no support), but with no evidence of a difference in SAEs; this evidence was of low and very low certainty, respectively. As with AEs from other smoking cessation treatments (e.g. NRT, [1]), AEs in these studies typically related to irritation at site (e.g. dry mouth, cough) and resolved over time. Only one study (Higgins 2024) reported any SAEs that were "related, probably related or possibly related" to the study product in an EC arm. However, it should be noted that participants in this arm also received very low nicotine content (VLNC) combustible cigarettes.

Beyond AEs and SAEs, we consider data on a range of safety‐ and health‐related outcomes, including CO and other toxicants, lung function, BP, pulse, and oxygen levels. Data on all of these outcome measures were limited; for most outcomes within most comparisons, only one or two studies currently contributed data. A companion paper (up to date to January 2022) provides more data on the measured toxicants, analysing studies based on actual use of EC and combustible cigarettes [333]. Consistent with findings from this review, the companion paper found that most measured toxicants were lower in people exclusively using EC than those exclusively smoking or those both smoking and using EC. Most measured toxicants were lower in people using both EC and smoking compared to smoking only.

We also have data from studies testing nicotine EC as adjuncts to other stop‐smoking treatments. Pooled data from two studies in which all participants received NRT showed that nicotine EC led to higher quit rates than non‐nicotine EC, but we judged both studies to be at high risk of bias, meaning the effect remains uncertain. Three studies compared nicotine EC + NRT to NRT alone. Statistical heterogeneity precluded meta‐analysis, but two out of three studies showed promise. It is well‐established that combining short‐ and long‐acting forms of NRT ('combined NRT') leads to greater success than single‐form NRT [334] but, of note, one of the studies showing a benefit of nicotine EC in this comparison compared nicotine EC + patch to short‐acting NRT + patch, suggesting that it is not just the 'combined NRT' effect that is driving increased effectiveness.

This review also includes data on the proportion of participants still using the study product (EC or pharmacotherapy) at six months or longer. There remains no clear evidence of a between‐group difference for this outcome, which is also explored further in a companion publication (up to date until 2022) [246]. We also searched for information investigating any association between withdrawal and smoking cessation, but no studies met our inclusion criteria for this outcome.

### Limitations of the evidence included in the review

We consider the certainty of the evidence as it relates to primary outcomes for our three main comparisons: nicotine EC versus NRT; nicotine EC versus non‐nicotine EC; nicotine EC versus behavioural support only/no support (Table; Table; Table).

Our summary of findings tables and assessments of certainty are based on the evidence from randomised controlled trials (RCTs). The cohort studies that we include were all automatically classified as having high risk of bias on the basis of non‐random treatment allocation, irrespective of other methodological considerations. Data presented from these studies need to be interpreted with caution. However, data from cohort studies were consistent with data from RCTs.

Risk of bias did not impact on the certainty of evidence for comparisons between nicotine and non‐nicotine EC, or between nicotine EC and NRT. For the latter, we judged six out of seven studies to be at low or unclear risk of bias overall. For the former, removing one study at high risk of bias increased the effect estimate for our efficacy outcome. Risk of bias decreased our certainty in the effect estimates for our nicotine EC versus behavioural support only/no support comparison as, due to the nature of the comparison, blinding was not possible and different levels of support could lead to bias.

All but two of our primary outcomes for our main comparisons were downgraded for imprecision, due to wide confidence intervals and few events. Other than the risk of bias and imprecision, we identified no other issues that decreased the certainty of the primary outcomes for our main comparisons.

Due to the small number of studies contributing to individual analyses, we were unable to assess publication bias in most cases and cannot rule this out. For the comparison nicotine EC versus behavioural/no support, two of our outcomes had more than 10 studies contributing to meta‐analysis and, therefore, we were able to generate funnel plots. The funnel plot for the exhaled CO outcome did not show any evidence of asymmetry (Figure). However, the funnel plot for smoking cessation showed evidence of asymmetry (Figure), influenced by large estimated intervention effects in some studies that had either a small sample size or a very low cessation event rate in both study arms. This apparent asymmetry may be explained either by some level of publication bias (e.g. smaller studies that show no benefit of nicotine EC remaining unpublished), or by heterogeneity between studies in either the intervention effect or the underlying outcome event rate. We carried out a sensitivity analysis removing the two studies that showed the most marked effects of EC (Dawkins 2020; Halpern 2018 [335, 336, 337]), and this did not change the interpretation of the pooled result. Therefore, we did not downgrade the certainty of the evidence for publication bias; however, we will continue to monitor this as the evidence is updated.

This field of research and EC devices themselves continue to evolve rapidly. This is the fifth update conducted as part of our 'living systematic review' approach, which will proceed until at least 2027, meaning we can continue to rapidly incorporate new evidence (see Supplementary material 8).

This update incorporates data from 1 March 2024 to 1 March 2025. Subsequent monthly searches will keep the evidence in this review current. Although studies predominantly came from the USA and UK, this review covers data from 16 countries. Geographical range in studies may be particularly important, due to the marked differences in EC regulation between countries; for example, studies conducted in countries that limit nicotine dose in EC, or allow only certain EC devices to be tested, may observe less pronounced effects on quitting. This review includes studies on some under‐researched populations, including people not motivated to quit smoking, people with substance misuse disorders, people with serious mental health conditions, people living in socially deprived areas and people experiencing homelessness. Quit rates in these groups are traditionally lower, but these groups may particularly stand to benefit from EC if they are effective because, in absolute terms, conventional cessation methods are often not as effective for them.

As well as the rapid pace of research in this field, evolutions in EC technology pose a challenge when considering the applicability of our evidence to the present. We had downgraded the certainty of our data in the 2016 update, as the devices tested in the trials were first‐generation 'cig‐a‐like' devices which did not deliver nicotine well, meaning the studies may have yielded more conservative estimates than would be seen with newer models, as newer devices and models have tended towards improved nicotine delivery. Nicotine delivery is also relevant to the comparator NRT arms tested; use of both a shorter‐ and a longer‐acting form of NRT show the highest success, and it is important that, where possible, this be the comparator chosen for such trials [334]. We no longer downgrade the evidence on this basis as studies with newer device types are now included, although there will always be a time lag between current devices and the research evidence available. Within our primary comparisons, none of the analyses of our primary outcomes signified substantial levels of statistical heterogeneity, despite the fact that different devices were used in the included studies. However, this could be because CIs were wide for individual studies, and does not rule out clinically significant differences in effects between EC types. As further data emerge, we hope to be able to formally test for differences in subgroup analyses, and in head‐to‐head comparisons of different device types. A companion paper explores available data on flavours in more detail, but is up‐to‐date until February 2024 and so not as up‐to‐date as this review [318; 319].

The AEs described in both the RCT and cohort studies continue to look similar, regardless of the brand of EC used or nicotine content, with placebo and nicotine‐containing EC showing similar numbers and types of AEs in direct comparisons. They also reflect what is reported in survey data [48, 338].

The structure of our analyses follows the standard practice of the Cochrane Tobacco Addiction Group, i.e. evaluating outcomes on an intention‐to‐treat basis, meaning our pooled results represent the effect of *offering an EC intervention.* This is different from evaluating the per‐protocol effect, or the effect only on those who use the EC to quit smoking entirely, or continue to smoke whilst also using EC. Although pragmatic and hopefully of use to those designing and delivering interventions, we acknowledge that our intention‐to‐treat approach limits the ability to use the data presented here to draw conclusions about biomarkers in subgroups of participants based on subsequent EC use/smoking profiles. A companion publication, up to date to January 2022, attempts to address this deficit [333].

#### Cessation

All three comparisons found effect estimates favouring nicotine EC for smoking cessation. For nicotine EC versus NRT, we continue to judge the evidence to be of high certainty, meaning we are very confident that the true effect lies close to the estimate of the effect. For nicotine EC versus non‐nicotine EC, we continue to judge the evidence to be of moderate certainty, meaning we think the true effect is likely to be close to the estimate of effect. For nicotine EC versus behavioural support only/no support, we continue to judge the evidence to be of low certainty, meaning we have limited confidence in the effect estimate. Nicotine EC versus non‐nicotine EC comparisons isolate the effect of nicotine as provided by an EC, and nicotine EC versus NRT comparisons isolate the effect of the sensorimotor elements provided by an EC. Both of these comparisons find a benefit of nicotine EC for smoking cessation. Therefore, it might logically follow that the comparison between nicotine EC and behavioural support only/no support would find a benefit in favour of nicotine EC, since this comparison would capture both pharmacological and sensorimotor mechanisms of effect. This increases our confidence in the effect of nicotine EC when compared to behavioural support alone or to no support. NRT has also been shown to be more effective than behavioural support alone, further supporting the likelihood that nicotine EC would be more effective than behavioural support alone [1].

#### Adverse and serious adverse events

Moderate‐certainty evidence does not show a difference in adverse events for nicotine EC compared to NRT, as well as for non‐nicotine EC. For all other outcomes in this category, evidence is of low or very low certainty. Imprecision remains a key issue for these outcomes, and particularly for SAEs. None of the analyses signalled serious harm, nor did complementary data from cohort studies but, unlike our cessation analyses, many of the CIs encompassed the possibility of both clinically significant harm and clinically significant benefit, and longer‐term health effects are unknown. This uncertainty should reduce as more studies become available.

### Limitations of the review processes

We consider the review process we used to be robust. For outcome assessment, we followed the standard methods used for Cochrane Tobacco Addiction Review Group cessation reviews. Our search strategy included CENTRAL, which incorporates findings from trial registries, and we were able to capture a number of ongoing studies. However, there may be unpublished data that our searches did not uncover. We also considered participants lost to follow‐up as continuing to smoke, which is standard practice in this field. There are concerns that frequently updating meta‐analyses can lead to issues with multiple testing; we followed Cochrane guidance in conducting this living systematic review and hence do not adjust for multiple testing [80].

Six of our review authors are authors of the included studies. These authors were not involved in the decisions about inclusion of their studies, or in risk of bias assessment for these studies; this approach is standard across all Cochrane reviews (regardless of subject area) and has been approved by the Cochrane editorial office as sufficient to avoid bias.

Our review includes studies funded by the tobacco/vaping industry – Cochrane guidelines (not tobacco addiction‐specific) mandate that studies be included regardless of funder, in order that the reviews remain transparent and rigorous. As noted throughout the results section, we removed studies with tobacco or vaping industry funding in sensitivity analyses; our conclusions were unchanged when we did this. This means that studies funded by tobacco or vaping industries *did not influence* our conclusions. We do not receive any funding from tobacco or vaping industries, and maintain a firm stance of independence.

### Agreements and disagreements with other studies or reviews

An overview of reviews of RCTs conducted in 2024 found that the majority of existing systematic reviews of trial data reached similar conclusions to those presented in this review regarding the effectiveness of nicotine‐containing EC for smoking cessation [339]. Reviews by Hanewinkel and colleagues and Li and colleagues found that nicotine EC were more effective than NRT, with risk ratios (RRs) of 1.58 (95% confidence interval (CI): 1.20 to 2.08) and 1.67 (95% CI: 1.21 to 2.28), respectively [340, 341]. Levett and colleagues reported greater cessation with nicotine EC compared to non‐nicotine EC (RR: 1.56, 95% CI: 1.13 to 2.15) and to non‐EC interventions (RR: 1.77, 95% CI: 1.29 to 2.44) [342]. Thomas and colleagues, in a large network meta‐analysis, found that high‐dose nicotine EC were more effective than placebo (odds ratio (OR): 3.22, 95% credible interval (CrI): 1.63 to 6.36) [343]. Chan and colleagues, in another network meta‐analysis, reported benefits of nicotine EC over non‐nicotine EC (RR: 2.09, 95% CI: 1.46 to 2.99) and over NRT (RR: 1.49, 95% CI: 1.09 to 2.04) [344]. Lindson and colleagues conducted a component network meta‐analysis (CNMA) and found nicotine EC significantly more effective than placebo (OR 2.37, 95% CrI: 1.73 to 3.24), with high‐certainty evidence [7]. Overall, the reviews were consistent in both the direction and magnitude of effect, supporting the conclusion that nicotine EC improve quit rates at six months or longer.

Some reviews reported greater uncertainty. Quigley and colleagues found an RR of 1.17 (95% CrI: 0.65 to 1.86) for nicotine EC compared with NRT, with the credible interval including the possibility of no difference [345]. Pound (2021) reported low or very low certainty evidence for an RR of 1.42 (95% CI: 0.97 to 2.09) for the same comparison [346]. Patnode and colleagues conducted a narrative synthesis, reviewing five studies comparing nicotine EC to non‐EC interventions and concluded that evidence was mixed and insufficient to draw firm conclusions [347]. Khoudigian and colleagues, in the earliest review included, reported a point estimate favouring nicotine EC over non‐nicotine EC (RR: 2.02, 95% CI: 0.97 to 4.22) [348], though the CI included the null. Differences in search dates, number of included trials, and analytic methods, such as reliance on point prevalence rather than sustained abstinence, are likely to have contributed to variation in findings across these reviews. Huang and colleagues conducted a review including participants at high risk of lung cancer, found a benefit for smoking cessation from nicotine EC over behavioural support alone (RR: 1.51, 95% CI: 1.03 to 2.21), but raised methodological concerns, including potential double counting of participants [349].

Findings related to safety outcomes were more variable. Of the reviews that meta‐analysed SAEs, two reported statistically significant increases in SAEs amongst EC users [342, 350]. Vanderkam and colleagues compared EC with NRT (RR 1.53, 95% CI: 1.02 to 2.30) [350]. However, in Levett, the increased SAEs were observed amongst users of non‐nicotine EC compared with non‐EC conventional smoking cessation interventions [342]. Most other reviews, including those by Li and colleagues and Ibrahim and colleagues, found no significant differences or were underpowered to detect rare events [341, 351].

Across the reviews, the certainty of evidence for SAE outcomes was generally low or very low, due to imprecision and short follow‐up durations. These inconsistencies reflect variation in study quality, reporting practices and low event rates. In contrast to the relative consistency of evidence on effectiveness, evidence on safety remains limited and uncertain, highlighting the need for longer‐term studies with sufficient power to assess harms.

Some reviews have examined the evidence on the role of EC flavours in quit rates. A systematic review by Liber and colleagues concluded that the evidence was inconclusive, reflecting highly heterogeneous study definitions and methodological limitations, and called for more high‐quality evidence, ideally from RCTs [352]. An overview of reviews examining the impact of EC flavours on any outcome reported that current evidence was inconclusive on the effect of flavours on quit rates [353].

## Authors' conclusions

### Implications for practice

Evidence suggesting that nicotine electronic cigarettes (EC) can aid in smoking cessation is consistent across several comparisons. There is high‐certainty evidence that EC with nicotine increase quit rates at six months or longer compared to nicotine replacement therapy (NRT), and moderate‐certainty evidence (limited by imprecision) that EC with nicotine probably increases quit rates at six months or longer compared to non‐nicotine EC. There is also low‐certainty evidence (limited by risk of bias) that EC with nicotine may increase quit rates compared to behavioural support alone or no support.

None of the evidence synthesised provides a clear indication that serious adverse events are increased by EC use. However, more long‐term data are needed, and this conclusion relates specifically to people using EC to stop smoking and not to people who have never smoked. The most commonly reported adverse effects are throat/mouth irritation, headache, cough, and nausea, which tend to dissipate with continued use. In some studies, reduced toxicant concentrations and biomarkers of harm were observed in people who smoked and switched to vaping, consistent with reductions seen in people who stopped smoking without EC.

### Implications for research

Further randomised controlled trials of nicotine EC are needed. All studies (including uncontrolled intervention cohort studies) should aim to assess the safety profile of EC for as long as possible (the current review only includes data up to two years), and ideally be powered to detect differences in safety outcomes, particularly serious adverse events.

Studies with active comparators (i.e. comparing nicotine EC to frontline smoking cessation pharmacotherapies, particularly those other than nicotine replacement therapy) are likely to be of particular use to decision‐makers, as are those testing EC as an adjunct to existing stop‐smoking pharmacotherapies; in particular, those testing combinations of traditional nicotine replacement therapy with e‐cigarettes (e.g. patch plus e‐cigarettes).

Studies should offer recent devices with good nicotine delivery to participants to be most representative of what will be on the market at the time results are released. Studies should also monitor and collect data on participants switching use of other devices during trials, and use of different flavours and nicotine strengths. Protocols and statistical analysis plans should be registered in advance and openly available.

Further RCTs need to be adequately powered. Further trials of pods and newer disposable devices would be of particular value, as would RCTs providing EC in a way that would be used in real‐world settings (e.g. taking into account individual preferences for strengths and flavours of EC liquids and even EC devices, and also allowing for changes in preferences over time). Further studies directly comparing nicotine EC based on characteristics including nicotine content and delivery, flavour, and device type, and reporting outcomes including cessation at six months or longer, would also be particularly useful.

## Supporting Information

Supplementary materials are available with the online version of this article: 10.1002/14651858.CD010216.pub10.

Supplementary materials are published alongside the article and contain additional data and information that support or enhance the article. Supplementary materials may not be subject to the same editorial scrutiny as the content of the article and Cochrane has not copyedited, typeset or proofread these materials. The material in these sections has been supplied by the author(s) for publication under a Licence for Publication and the author(s) are solely responsible for the material. Cochrane accordingly gives no representations or warranties of any kind in relation to, and accepts no liability for any reliance on or use of, such material.

**Supplementary material 1** Search strategies

**Supplementary material 2** Characteristics of included studies

**Supplementary material 3** Characteristics of excluded studies

**Supplementary material 4** Characteristics of studies awaiting classification

**Supplementary material 5** Characteristics of ongoing studies

**Supplementary material 6** Analyses

**Supplementary material 7** Data package

**Supplementary material 8** Protocol for living systematic review

**Supplementary material 9** Toxins/carcinogen names and abbreviations

**Supplementary material 10** Changes in methods between living review updates

**Supplementary material 11** Adverse events data not contributing to meta‐analyses

**Supplementary material 12** Serious adverse events data not contributing to meta‐analyses

**Supplementary material 13** Carbon monoxide data not contributing to meta‐analyses

**Supplementary material 14** Heart rate data not contributing to meta‐analyses

**Supplementary material 15** Blood pressure data not contributing to meta‐analyses

**Supplementary material 16** Data on known toxicants/carcinogens from studies not contributing to meta‐analyses

**Supplementary material 17** Data on lung function from studies not contributing to meta‐analyses

**Supplementary material 18** Product use at 6+ months for data not contributing to meta‐analyses

## Figures and Tables

**Table:** 

**Date**	**Event**	**Description**
10 November 2025	New search has been performed	Updated to include fourteen new studies; searches to 1 March 2025
10 November 2025	New citation required but conclusions have not changed	Main conclusions remain unchanged. Update triggered as first study comparing nicotine EC with non‐nicotine EC and varenicline reporting abstinence at six months or longer was published (results inconclusive).

**Table:** 

**Date**	**Event**	**Description**
29 January 2025	New search has been performed	Updated to include two new studies; searches to 1 February 2024
29 January 2025	New citation required but conclusions have not changed	Main conclusions remain unchanged. Update triggered as first study comparing flavours and reporting abstinence at six months or longer was published (results inconclusive).
8 January 2024	New search has been performed	This is a living systematic review. In this update, we incorporate data to 1st July 2023.
8 January 2024	New citation required and conclusions have changed	Certainty of evidence for cessation outcome for comparison with behavioural support/no support upgraded from very low to low
15 March 2023	Amended	This is a Living Systematic Review. We run and screen searches monthly. Last search date: 1st March 2023. In addition to the studies identified from August 2022 to February 2023, we found one new reference linked to a previously identified study. We will incorporate this into the review as part of a future update. We have also fixed a typo in the plain language summary. For future monthly search results, please see 'Monthly search results' via the following link: https://www.cebm.ox.ac.uk/research/electronic-cigarettes-for-smoking-cessation-cochrane-living-systematic-review-1.
4 February 2023	Amended	This is a Living Systematic Review. We run and screen searches monthly. Last search date: 1st February 2023. In addition to the studies identified from August 2022 to January 2023, we found one new included study, one new ongoing study and 2 linked references. We will incorporate these into the review as part of a future update. The DOI for the 1 new included study (Kanobe 2022) is: https://doi.org/10.1038/s41598-022-25054-z.
5 January 2023	Amended	This is a Living Systematic Review. We run and screen searches monthly. Last search date: 3rd January 2023. In addition to the studies identified from August 2022 to December 2022, we found one new ongoing study. We will incorporate these into the review as part of a future update. In addition, some minor corrections were made to the Characteristics of Included Studies table for Hajek 2022 based on a published correction to the study's primary manuscript (https://doi.org/10.1038/s41591-022-02099-1).
12 December 2022	Amended	This is a Living Systematic Review. We run and screen searches monthly. Last search date: 1st December 2022. In addition to the studies identified from August 2022 to November 2022, we found one new ongoing study and 3 records linked to previously identified studies. We will incorporate these into the review as part of a future update.
25 November 2022	Amended	This is a Living Systematic Review. We run and screen searches monthly. Last search date: 1st November 2022. We found no new eligible references. As part of this amendment, we also updated the citation for additional reference [318], and corrected a slight error in wording in the Discussion section.
19 October 2022	New search has been performed	17 new included studies. Incorporates evidence up to the 1st July 2022.
19 October 2022	New citation required and conclusions have changed	Certainty changes for some of the primary outcomes.
7 October 2022	Amended	This is a Living Systematic Review. We run and screen searches monthly. Last search date: 1st October 2022. In addition to the studies identified from June 2021 to September 2022, we found one new included study, 3 new ongoing studies and 1 record linked to a previously identified study. The DOI for the 1 new included study is: Klonizakis 2022 (https://doi.org/10.1186/s12916-022-02451-9). We will incorporate these into the review as part of a future update.
27 September 2022	Amended	This is a Living Systematic Review. We run and screen searches monthly. Last search date: 1st September 2022. In addition to the studies identified from June 2021 to August 2022, we found two records linked to previously identified studies. We will incorporate these into the review as part of a future update.
17 August 2022	Amended	This is a Living Systematic Review. We run and screen searches monthly. Last search date: 1st August 2022. In addition to the studies identified from June 2021 to July 2022, we found two new included studies, 1 new ongoing study and 3 records linked to previously identified studies. The DOIs for the 2 new included studies are: Coffey 2020 (DOI: 10.1177/1757913920912436) and Price 2022 (DOI: https://doi.org/10.1186/s12889-022-13711-x). We will incorporate these into the review as part of a future update.
8 July 2022	Amended	This is a Living Systematic Review. We run and screen searches monthly. Last search date: 1st July 2022. In addition to the studies identified from June 2021 to June 2022, we found four new included studies, 1 new ongoing study and 8 records linked to previously identified studies. The DOIs for 3 of the new included studies are: Edmiston 2022 (DOI: 10.1093/ntr/ntac029); Tattan‐Birch 2022 (DOI: 10.1093/ntr/ntac149) and Morphett 2022a (DOI: 10.1093/ntr/ntab266). The fourth new included study was presented at SRNT 2022 (abstract reference: SYM17‐4). We will incorporate these into the review as part of a future update.
15 June 2022	Amended	This is a Living Systematic Review. We run and screen searches monthly. Last search date: 1st June 2022. In addition to the studies identified from June 2021 to May 2022, we found three new included studies (all previously listed as ongoing studies) and 2 records linked to a previously identified study. The DOIs for the new included studies are: Hajek 2022 (https://doi.org/10.1038/s41591-022-01808-0); Bonafont Reyes 2022 (https://doi.org/10.1111/jgs.17755) and Vickerman 2022 (https://doi.org/10.1093/ntr/ntac129). We will incorporate these into the review as part of a future update.
6 May 2022	Amended	This is a Living Systematic Review. We run and screen searches monthly. Last search date: 1st May 2022. In addition to the studies identified from June 2021 to April 2022, we found two new included studies (previously listed as ongoing studies), 3 new ongoing studies and 2 records linked to previously identified studies. The DOIs for the new included studies are: Skelton 2022 (doi: 10.1016/j.addbeh.2022.107328); Pratt 2022 (doi: 10.1093/ntr/ntac082). We will incorporate these into the review as part of a future update.
6 April 2022	Amended	This is a Living Systematic Review. We run and screen searches monthly. Last search date: 1st April 2022. In addition to the studies identified from June 2021 to March 2022, we found 4 new ongoing studies. We will incorporate these into the review as part of a future update.
7 March 2022	Amended	This is a Living Systematic Review. We run and screen searches monthly. Last search date: 1st March 2022. In addition to the studies identified from June 2021 to February 2022, we found 1 record linked to a study already identified as ongoing. We will incorporate these into the review as part of a future update.
11 February 2022	Amended	This is a Living Systematic Review. We run and screen searches monthly. Last search date: 1st February 2022. In addition to the studies identified from June 2021 to January 2022, we found 2 ongoing studies and 2 records linked to studies already included in the review. We will incorporate these into the review as part of a future update.
12 January 2022	Amended	This is a Living Systematic Review. We run and screen searches monthly. Last search date: 1st January 2022. In addition to the studies identified from June to December 2021, we found 4 ongoing studies and 1 record linked to a study already included in the review. We will incorporate these into the review as part of a future update.[Enter text here]
16 December 2021	Amended	This is a Living Systematic Review. We run and screen searches monthly. Last search date: 1st December 2021. In addition to the studies identified from June to November 2021, we found six new included studies, 15 ongoing studies and 18 records linked to studies already included in the review. The DOI or trial IDs for the new included studies are: NCT02433015; NCT03111537; NCT03185546; NCT03358953; Caponnetto 2021 (DOI: 10.1093/ntr/ntab005); Lum 2021 (DOI: 10.1016/j.addbeh.2021.107097). We will incorporate these into the review as part of a future update.
3 November 2021	Amended	This is a Living Systematic Review. We run and screen searches monthly. Last search date 1st November 2021. In addition to the studies identified from June to October 2021, we found one new included study. The DOI for the new included study (Okuyemi 2021) is 10.1093/ntr/ntab212. We will incorporate these into the review as part of a future update.
20 October 2021	Amended	This is a Living Systematic Review. We run and screen searches monthly. Last search date 1st October 2021. In addition to the studies identified from June to September 2021, we found one new included study two reports linked to studies already in the review, and one new ongoing. The DOI for the new included study (Morris 2021) is https://doi.org/10.1007/s11739-021-02813-w. We will incorporate these into the review as part of a future update.
16 September 2021	Amended	Change made to correct data; SAE data from Cobb 2021 moved from comparison with NRT to comparison with no‐nicotine EC. No changes to conclusions.
6 September 2021	New search has been performed	Updated with five new included studies. Incorporates evidence up to 1 May 2021.
6 September 2021	New search has been performed	This is a Living Systematic Review. We run and screen searches monthly. Last search update 1st September 2021. We found no new studies for inclusion this month; however results from searches carried out from June to August 2021 will be incorporated into a future update of the review.
6 September 2021	New citation required and conclusions have changed	New secondary outcome added (continued product use), first study of pod device contributing data to cessation meta‐analysis added, two new comparisons added (nicotine salt EC versus freebase nicotine EC; advice on how to quit smoking using EC versus no EC advice). Conclusions for primary outcomes remain largely unchanged.
5 August 2021	Amended	This is a Living Systematic Review. We run and screen searches monthly. Last search date 2nd August 2021. In addition to the studies identified from March to July 2021, we found two new ongoing studies and one report linked to a study already in the review. We will incorporate these into the review as part of a future update.
7 July 2021	Amended	This is a Living Systematic Review. We run and screen searches monthly. Last search date 1st July 2021. In addition to the studies identified from March to June 2021, we found two new included studies and two reports linked to studies already in the review. DOIs for the two new included studies are as follows: Myers‐Smith 2021: https://doi.org/10.1111/add.15628 & Kimber 2021: 10.1016/j.addbeh.2021.106909. We will incorporate these into the review as part of a future update.
9 June 2021	Amended	This is a Living Systematic Review. We run and screen searches monthly. Last search date 1st June 2021. In addition to the studies identified from March to May 2021, we found one report linked to a study already in the review, one ongoing study, and one potentially new study that we are looking into further. We will incorporate these into the review as part of a future update. As part of this new update we will also include a new outcome ‐ proportion of people still using e‐cigarettes or other pharmacotherapy at longest follow‐up.
12 May 2021	Amended	This is a Living Systematic Review. We run and screen searches monthly. Last search date 4th May 2021. In addition to the studies identified from March and April 2021, we found four new ongoing studies. We will incorporate these into the review as part of a future update.
15 April 2021	New search has been performed	Updated with six new included studies and new data from one previously included study. Most recent search 1 Feb 2021.
15 April 2021	New citation required and conclusions have changed	6 new included studies added (Czoli 2019; Ikonomidis 2020a [355]; Ozga‐Hess 2019; Pulvers 2020; Scheibein 2020 [356, 357]; Yingst 2020), certainty in finding of no difference in adverse events between nicotine EC and non‐nicotine EC updated to moderate (from low). First study of pod EC device included.
1 April 2021	Amended	This is a Living Systematic Review. We run and screen searches monthly. Last search date 1st April 2021. In addition to the studies identified from March 2021 we found two new ongoing studies and one paper linked to a study already included in the review. We will incorporate these into the review as part of a future update.
17 March 2021	Amended	This is a Living Systematic Review. We run and screen searches monthly. Last search date 1st March 2021. Studies identified in March are not included in this version of the review, but will be incorporated into a subsequent version. We found four new included studies, five new ongoing studies and five papers linked to studies already included in the review. The four new included studies were all conference abstracts; three of which were identified from the SRNT 2021 abstract book (SYM2A, SYM2B, PH‐353; www.srnt.org/page/2021_Meeting). The fourth is available here: dx.doi.org/10.1016/j.drugalcdep.2015.07.1091.
4 February 2021	Amended	This is a Living Systematic Review. We run and screen searches monthly. Last search date 1st February 2021. In addition to the studies identified from our December 2020 and January 2021 searches we found one paper linked to a study already included in the review (Lucchiari 2022), and have preliminary results from a study listed as ongoing (Begh 2021). We will incorporate this paper and data into the review as part of a future update.
20 January 2021	Amended	This is a Living Systematic Review. Searches are run and screened monthly. Last search date 4th January 2021. In addition to the studies identified from our December 2020 searches we found four new completed studies, one new ongoing study and one paper linked to a study already included in the review. These studies and papers will be incorporated into the review at the next update. DOIs for the four new included studies are as follows: Ozga‐Hess et al. 2019: 10.1016/j.addbeh.2019.106105; Pulvers et al. 2020: 10.1001/jamanetworkopen.2020.26324; Scheibein 2020: 10.1186/s12954‐020‐00406‐y; Yingst et al. 2020: 10.1080/09540121.2019.1687835
15 December 2020	Amended	This is a Living Systematic Review. Searches are run and screened monthly. Last search date 1st December 2020. Searches found 3 new completed studies, 11 new ongoing studies and 9 papers linked to studies already included in the review. These studies and papers will be incorporated into the review at the next update. DOIs for the three new included studies are as follows: Czoli et al:10.1093/ntr/nty174; Bonevski et al: 10.1093/ntr/ntaa143; Eisenberg et al: 10.1001/jama.2020.18889.
20 July 2020	New search has been performed	New searches run January 2020. 35 new studies added. Living systematic review protocol incorporated
20 July 2020	New citation required and conclusions have changed	Strength of evidence increased for existing comparisons; new comparisons added
14 December 2016	Amended	Clarification on outcome data from Adriaens ‐ no changes to conclusions
23 June 2016	New search has been performed	Update search run January 2016, 11 new included studies added. Reduction removed as outcome, now covered in Harm Reduction review.
23 June 2016	New citation required but conclusions have not changed	11 new included studies added; no changes to conclusions.

## Additional information

### Registration and protocol

Original 2012 protocol available via DOI: 10.1002/14651858.CD010216. Updated 2023 protocol available via DOI 10.17605/OSF.IO/ZWGSK. 2025 updates to protocol available via DOI: 10.17605/OSF.IO/59M4U and DOI 10.17605/OSF.IO/UPGJC.

### Data, code and other materials

As part of the published Cochrane review, the following is made available for users of the Cochrane Library: our search strategies Supplementary material 1; full citations for all included studies, all ongoing studies, relevant excluded studies, and studies awaiting classification in the reference section of the review; study data, including study information, study arms and risk of bias assessments in our characteristics of studies tables (Supplementary material 2; Supplementary material 3; Supplementary material 4; Supplementary material 5); analysis data, including overall estimates, subgroup estimates, and individual data rows (all the rows in all the analyses) is in the main review and in Supplementary material 6. Data supporting the results of this systematic review are from published information and are available in the review. All analyses have been conducted within RevMan. For details of the computational methods, see https://documentation.cochrane.org/revman‐kb/statistical‐methods‐210600101.html. Analyses were conducted within Cochrane’s authoring tool, RevMan, using the inbuilt computation methods. Data were extracted into Microsoft Word and are available from the authors on reasonable request. The data are shared within the published review directly from RevMan Supplementary material 7.

### History

Protocol first published: Issue 11, 2012 Review first published: Issue 12, 2014

## References

[1] Hartmann-Boyce J, Chepkin SC, Ye W, Bullen C, Lancaster T. Nicotine replacement therapy versus control for smoking cessation. Cochrane Database of Systematic Reviews 2018, Issue 5. Art. No: CD000146. [DOI: 10.1002/14651858.CD000146.pub5] PMC6353172 29852054

[2] Hughes JR, Keely J, Naud S. Shape of the relapse curve and long-term abstinence among untreated smokers. Addiction (Abingdon, England) 2004;99(1):29-38. 10.1111/j.1360-0443.2004.00540.x 14678060

[3] Hartmann-Boyce J, Hong B, Livingstone-Banks J, Wheat H, Fanshawe TR. Additional behavioural support as an adjunct to pharmacotherapy for smoking cessation. Cochrane Database of Systematic Reviews 2019, Issue 6. Art. No: CD009670. [DOI: 10.1002/14651858.CD009670.pub4] PMC6549450 31166007

[4] Hartmann-Boyce J, Livingstone-Banks J, Ordonez-Mena J, Fanshawe TR, Lindson N, Freeman SC, et al. Behavioural interventions for smoking cessation: an overview and network meta-analysis. Cochrane Database of Systematic Reviews 2021, Issue 1. Art. No: CD013229. [DOI: 10.1002/14651858.CD013229.pub2] PMC11354481 33411338

[5] Hartmann-Boyce J, Ordonez-Mena J, Livingstone-Banks J, Fanshawe T, Lindson N, Freeman S, et al. Behavioural programmes for cigarette smoking cessation: investigating interactions between behavioural, motivational and delivery components in a systematic review and component network meta-analysis. Addiction (Abingdon, England) 2022;117(8):2145-56. [DOI: 10.1111/add.15791] 34985167

[6] Livingstone-Banks J, Fanshawe TR, Thomas KH, Theodoulou A, Hajizadeh A, Hartman L, et al. Nicotine receptor partial agonists for smoking cessation. Cochrane Database of Systematic Reviews 2023, Issue 6. Art. No: CD006103. [DOI: 10.1002/14651858.CD006103.pub9] PMC10169257 37142273

[7] Lindson N, Theodoulou A, Ordóñez-Mena JM, Fanshawe TR, Sutton AJ, Livingstone-Banks J, et al. Pharmacological and electronic cigarette interventions for smoking cessation in adults: component network meta-analyses. Cochrane Database of Systematic Reviews 2023, Issue 9. Art. No: CD015226. [DOI: 10.1002/14651858.CD015226.pub2] PMC10495240 37696529

[8] Livingstone-Banks J, Lindson N, Hartmann-Boyce J. Effects of interventions to combat tobacco addiction: Cochrane update of 2021 to 2023 reviews. Addiction (Abingdon, England) 2024;119(12):2101–15. [DOI: 10.1111/add.16624] 39231467

[9] Notley C, Ward E, Dawkins L, Holland R. The unique contribution of e-cigarettes for tobacco harm reduction in supporting smoking relapse prevention. Harm Reduction Journal 2018;15(1):31. 10.1186/s12954-018-0237-7 PMC6011187 29921278

[10] Buss B, Kock L, Wesr R, Beard E, Kale D, Brown J. Trends in electronic cigarette use in England, smoking in England. https://smokinginengland.info/graphs/e-cigarettes-latest-trends; July 2023 (accessed 27 September 2023).

[11] Balfour D. The neurobiology of tobacco dependence: a preclinical perspective on the role of dopamine projections to the nucleus. Nicotine & Tobacco Research 2004;6(6):899-912. 10.1080/14622200412331324965 15801566

[12] Benowitz NL. Nicotine addiction. New England Journal of Medicine 2010;362(24):2295-303. 10.1056/NEJMra0809890 PMC2928221 20554984

[13] Rose JE. Nicotine and non-nicotine factors in cigarette addiction. Psychopharmacology 2006;184(3-4):274-85. 10.1007/s00213-005-0250-x 16362402

[14] Rose JE, Behm FM, Levin ED. Role of nicotine dose and sensory cues in the regulation of smoke intake. Pharmacology Biochemistry & Behavior 1993;44(4):891-900. 10.1016/0091-3057(93)90021-k 8469698

[15] Rose JE, Behm FM, Westman EC, Johnson M. Dissociating nicotine and nonnicotine components of cigarette smoking. Pharmacology Biochemistry & Behavior 2000;67(1):71-81. 10.1016/s0091-3057(00)00301-4 11113486

[16] Westman EC, Behm FM, Rose JE. Dissociating the nicotine and airway sensory effects of smoking. Pharmacology Biochemistry & Behavior 1996;53(2):309-15. 10.1016/0091-3057(95)02027-6 8808137

[17] Rose JE, Tashkin DP, Ertle A, Zinser MC, Lafer R. Sensory blockade of smoking satisfaction. Pharmacology Biochemistry & Behavior 1985;23(2):289-93. 10.1016/0091-3057(85)90572-6 4059314

[18] Levin ED, Behm F, Carnahan E, LeClair R, Shipley R, Rose JE. Clinical trials using ascorbic acid aerosol to aid smoking cessation. Drug and Alcohol Dependence 1993;33(3):211-23. 10.1016/0376-8716(93)90108-3 8261886

[19] Rose JE, Behm FM. Inhalation of vapor from black pepper extract reduces smoking withdrawal symptoms. Drug and Alcohol Dependence 1994;34(3):225-9. 10.1016/0376-8716(94)90160-0 8033760

[20] Westman EC, Behm FM, Rose JE. Airway sensory replacement combined with nicotine replacement for smoking cessation. A randomized, placebo-controlled trial using a citric acid inhaler. Chest 1995;107(5):1358-64. 10.1378/chest.107.5.1358 7750331

[21] Przulj D, McRobbie H, Hajek P. The effect of sensorimotor replacement on smoking cessation and craving. Open Addiction Journal 2013;5:41-50.

[22] Donny EC, Houtsmuller E, Stitzer ML. Smoking in the absence of nicotine: behavioral, subjective and physiological effects over 11 days. Addiction (Abingdon, England) 2007;102(2):324-34. 10.1111/j.1360-0443.2006.01670.x 17222288

[23] Donny EC, Denlinger RL, Tidey JW, Koopmeiners JS, Benowitz NL, Vandrey RG, et al. Randomized trial of reduced-nicotine standards for cigarettes. New England Journal of Medicine 2015;373(14):1340-9. 10.1056/NEJMsa1502403 PMC4642683 26422724

[24] Pickworth WB, Fant RV, Nelson RA, Rohrer MS, Henningfield JE. Pharmacodynamic effects of new de-nicotinized cigarettes. Nicotine & Tobacco Research 1999;1(4):357-64. 10.1080/14622299050011491 11072433

[25] Barrett SP. The effects of nicotine, denicotinized tobacco, and nicotine-containing tobacco on cigarette craving, withdrawal, and self-administration in male and female smokers. Behavorial Pharmacology 2010;21(2):144-52. 10.1097/FBP.0b013e328337be68 20168213

[26] Donny EC, Jones M. Prolonged exposure to denicotinized cigarettes with or without transdermal nicotine. Drug and Alcohol Dependence 2009;104(1-2):23-33. 10.1016/j.drugalcdep.2009.01.021 PMC2726800 19446968

[27] McRobbie H, Przulj D, Smith KM, Cornwall D. Complementing the standard multicomponent treatment for smokers with denicotinized cigarettes: a randomized trial. Nicotine & Tobacco Research 2016;18(5):1134-41. 10.1093/ntr/ntv122 26045250

[28] Perkins KA, Karelitz JL, Conklin CA, Sayette MA, Giedgowd GE. Acute negative affect relief from smoking depends on the affect situation and measure but not on nicotine. Biological Psychology 2010;67(8):707-14. 10.1016/j.biopsych.2009.12.017 PMC5367382 20132927

[29] Walker N, Howe C, Bullen C, Grigg M, Glover M, McRobbie H, et al. The combined effect of very low nicotine content cigarettes, used as an adjunct to usual Quitline care (nicotine replacement therapy and behavioural support), on smoking cessation: a randomized controlled trial. Addiction (Abingdon, England) 2012;107(10):1857-67. 10.1111/j.1360-0443.2012.03906.x 22594651

[30] Notley C, Colllins R. Redefining smoking relapse as recovered social identity - secondary qualitative analysis of relapse narratives. Journal of Substance Use 2018;23(6):660-6.

[31] Hajek P, West R, Foulds J, Nilsson F, Burrows S, Meadow A. Randomized comparative trial of nicotine polacrilex, a transdermal patch, nasal spray, and an inhaler. Archives of Internal Medicine 1999;159(17):2033-8. 10.1001/archinte.159.17.2033 10510989

[32] Schneider NG, Olmstead RE, Franzon MA, Lunell E. The nicotine inhaler: clinical pharmacokinetics and comparison with other nicotine treatments. Clinical Pharmcokinetics 2001;40(9):661-84. 10.2165/00003088-200140090-00003 11605715

[33] Bullen C, McRobbie H, Thornley S, Glover M, Lin R, Laugesen M. Effect of an electronic nicotine delivery device (e cigarette) on desire to smoke and withdrawal, user preferences and nicotine delivery: randomised cross-over trial. Tobacco Control 2010;19(2):98-103. [DOI: 10.1136/tc.2009.031567] 20378585

[34] Cox S, Jakes S. Nicotine and e-cigarettes: rethinking addiction in the context of reduced harm. International Journal of Drug Policy 2017;44:84-5. 10.1016/j.drugpo.2017.03.009 28454011

[35] Goniewicz ML, Kuma T, Gawron M, Knysak J, Kosmider L. Nicotine levels in electronic cigarettes. Nicotine & Tobacco Research 2012;15(1):158-66. 10.1093/ntr/nts103 22529223

[36] Goniewicz ML, Hajek P, McRobbie H. Nicotine content of electronic cigarettes, its release in vapour and its consistency across batches: regulatory implications. Addiction (Abingdon, England) 2014;109(3):500-7. 10.1111/add.12410 24345184

[37] Cox S, Leigh NJ, Vanderbush TS, Choo E, Goniewicz ML, Dawkins L. An exploration into "do-it-yourself" (DIY) e-liquid mixing: sers' motivations, practices and product laboratory analysis. Addictive Behaviors Reports 2019;9:100151. 10.1016/j.abrep.2018.100151 PMC6542371 31193745

[38] Eissenberg T. Electronic nicotine delivery devices: ineffective nicotine delivery and craving suppression after acute administration. Tobacco Control 2010;19(1):87-8. [DOI: 10.1136/tc.2009.033498] PMC3208854 20154061

[39] Vansickel AR, Cobb CO, Weaver MF, Eissenberg TE. A clinical laboratory model for evaluating the acute effects of electronic "cigarettes": nicotine delivery profile and cardiovascular and subjective effects. Cancer Epidemiology, Biomarkers & Prevention 2010;19(8):1945-53. [DOI: 10.1158/1055-9965.EPI-10-0288] PMC2919621 20647410

[40] Hajek P, Goniewicz ML, Phillips A, Myers Smith K, West O, McRobbie H. Nicotine intake from electronic cigarettes on initial use and after 4 weeks of regular use. Nicotine & Tobacco Research 2015;17(2):175-9. 10.1093/ntr/ntu153 PMC4892703 25122503

[41] Vansickel AR, Weaver MF, Eissenberg T. Clinical laboratory assessment of the abuse liability of an electronic cigarette. Addiction (Abingdon, England) 2012;107(8):1483-500. [DOI: 10.1111/j.1360-0443.2012.03791.x] PMC3330136 22229871

[42] Vansickel AR, Eissenberg T. Electronic cigarettes: effective nicotine delivery after acute administration. Nicotine & Tobacco Research 2013;15(1):267-70. [DOI: 10.1093/ntr/ntr316] PMC3524053 22311962

[43] Yingst JM, Foulds J, Veldheer S, Hrabovsky S, Trushin N, Eissenberg TT, et al. Nicotine absorption during electronic cigarette use among regular users. PloS One 2019;14(7):e0220300. 10.1371/journal.pone.0220300 PMC6657878 31344110

[44] Yingst JM, Hrabovsky S, Hobkirk A, Trushin N, Richie JP Jr, Foulds J. Nicotine absorption profile among regular users of a pod-based electronic nicotine delivery system. JAMA Network Open 2019;2(11):e1915494. 10.1001/jamanetworkopen.2019.15494 PMC6902801 31730180

[45] Hajek P, Przulj D, Phillips A, Anderson R, McRobbie H. Nicotine delivery to users from cigarettes and from different types of e-cigarettes. Psychopharmacology 2017;234(5):773-9. 10.1007/s00213-016-4512-6 PMC5306435 28070620

[46] Action on Smoking and Health. Use of vapes (e-cigarettes) among adults in Great Britain. ash.org.uk/resources/view/use-of-e-cigarettes-among-adults-in-great-britain (accessed 11 September 2025).

[47] Chen C, Zhuang YL, Zhu SH. E-cigarette design preference and smoking cessation: a U.S. population study. American Journal of Preventive Medicine 2016;51(3):356-63. [DOI: 10.1016/j.amepre.2016.02.002] PMC4992632 27005984

[48] Dawkins L, Turner J, Roberts A, Soar K. 'Vaping' profiles and preferences: an online survey of electronic cigarette users. Addiction (Abingdon, England) 2013;108(6):1115-25. 10.1111/add.12150 23551515

[49] Farsalinos KE, Polosa R. Safety evaluation and risk assessment of electronic cigarettes as tobacco cigarette substitutes: a systematic review. Therapeutic Advances in Drug Safety 2014;5(2):67-86. 10.1177/2042098614524430 PMC4110871 25083263

[50] Zavala-Arciniega L, Hirschtick JL, Meza R, Fleischer NL. E-cigarette characteristics and cigarette smoking cessation behaviors among U.S. adult dual users of cigarettes and e-cigarettes. Preventive Medicine Reports 2022;26(101748):1-6. [DOI: 10.1016/j.pmedr.2022.101748] PMC8897705 35256927

[51] Hajek P, Pittaccio K, Pesola F, Myers Smith K, Phillips-Waller A, Przulj D. Nicotine delivery and users' reactions to Juul compared with cigarettes and other e-cigarette products. Addiction (Abingdon, England) 2020;115(6):1141-8. 10.1111/add.14936 PMC7318270 31994254

[52] Belderson P, Ward E, Pope I, Notley C. Selecting an e-cigarette for use in smoking cessation interventions and healthcare services: findings from patient and public consultation for the COSTED trial. BMJ Open 2024;14(3):e078677. [DOI: 10.1136/bmjopen-2023-078677] [PMID: 38443079] PMC11146363

[53] Prochaska JJ, Vogel EA, Benowitz N. Nicotine delivery and cigarette equivalents from vaping a JUULpod. Tobacco Control 2022;31(e1):e88-93. [DOI: 10.1136/tobaccocontrol-2020-056367] PMC8460696 33762429

[54] Romberg AR, Miller Lo EJ, Cuccia AF, Willett JG, Xiao H, Hair EC, et al. Patterns of nicotine concentrations in electronic cigarettes sold in the United States, 2013-2018. Drug and Alcohol Dependence 2019;203:1-7. [DOI: 10.1016/j.drugalcdep.2019.05.029] PMC6765364 31386973

[55] European Parliament and Council of the European Union. Directive 2014/40/EU of the European Parliament and of the Council of 3 April 2014 on the approximation of the laws, regulations and administrative provisions of the Member States concerning the manufacture, presentation and sale of tobacco and related products and repealing Directive 2001/37/EC. EUR-Lex - 32014L0040 - EN - EUR-Lex (europa.eu) 2014 (accessed 25 November 2014).

[56] Huang J, Duan Z, Kwok J, Binns S, Vera LE, Kim Y, et al. Vaping versus JUULing: how the extraordinary growth and marketing of JUUL transformed the US retail e-cigarette market. Tobacco Control 2019;28(2):146-51. 10.1136/tobaccocontrol-2018-054382 PMC6274629 29853561

[57] Talih S, Salman R, El-Hage R, Karam E, Salam S, Karaoghlanian N, et al. A comparison of the electrical characteristics, liquid composition, and toxicant emissions of JUUL USA and JUUL UK e-cigarettes. Scientific Reports 2020;10(1):7322. 10.1038/s41598-020-64414-5 PMC7192936 32355323

[58] Tattan-Birch H, Jackson SE, Kock L, Dockrell M, Brown J. Rapid growth in disposable e-cigarette vaping among young adults in Great Britain from 2021 to 2022: a repeat cross-sectional survey. Addiction (Abingdon, England) 2023;118(2):382-6. [DOI: 10.1111/add.16044] PMC10086805 36065820

[59] Tattan-Birch H, Brown J, Shahab L, Beard E, Jackson S. Trends in vaping and smoking following the rise of disposable e-cigarettes: a repeat cross-sectional study in England between 2016 and 2023. Lancet Regional Health - Europe 2024;42:100924. [DOI: 10.1016/j.lanepe.2024.100924] PMC11281926 39070753

[60] UK Government. Single-use vapes banned from 1 June 2025. www.gov.uk/government/news/single-use-vapes-banned-from-1-june-2025 (accessed 05 Sept 2025).

[61] Dawkins LE, Kimber CF, Doig M, Feyerabend C, Corcoran O. Self-titration by experienced e-cigarette users: blood nicotine delivery and subjective effects. Psychopharmacology 2016;233(14-16):2933-41. [DOI: 10.1007/s00213-016-4338-2] 27235016

[62] Dawkins L, Cox S, Goniewicz M, McRobbie H, Kimber C, Doig M, et al. 'Real-world' compensatory behaviour with low nicotine concentration e-liquid: subjective effects and nicotine, acrolein and formaldehyde exposure. Addiction (Abingdon, England) 2018;113(10):1874-82. 10.1111/add.14271 PMC6150437 29882257

[63] Smets J, Baeyens F, Chaumont M, Adriaens K, Van Gucht D. When less is more: vaping low-nicotine vs. high-nicotine e-liquid is compensated by increased wattage and higher liquid consumption. International Journal of Environmental Research and Public Health 2019;16(5):723. 10.3390/ijerph16050723 PMC6427796 30823395

[64] McNeill A, Simonavičius E, Brose LS, Taylor E, East K, Zuikova E, et al. Nicotine vaping in England: an evidence update including health risks and perceptions. A report commissioned by the Office for Health Improvement and Disparities. London: Office for Health Improvement and Disparities. assets.publishing.service.gov.uk/government/uploads/system/uploads/attachment_data/file/1107701/Nicotine-vaping-in-England-2022-report.pdf (accessed prior to 3 November 2022).

[65] Du Y, Liu B, Xu G, Rong S, Sun Y, Wu Y, et al. Association of electronic cigarette regulations with electronic cigarette use among adults in the United States. JAMA Network Open 2020;3(1):e1920255. 10.1001/jamanetworkopen.2019.20255 PMC7042861 32003818

[66] Centres for Disease Control and Prevention. Outbreak of lung injury associated with the use of e-cigarette, or vaping, products. www.cdc.gov/tobacco/basic_information/e-cigarettes/severe-lung-disease.html#latest-outbreak-information; February 2020 (accessed 12 August 2020).

[67] Hall W, Gartner C, Bonevski B. Lessons from the public health responses to the US outbreak of vaping-related lung injury. Addiction 2020 [Epub ahead of print]. 10.1111/add.15108 32364274

[68] Tattan-Birch H, Brown J, Shahab L, Jackson SE. Association of the US outbreak of vaping-associated lung injury with perceived harm of e-cigarettes compared with cigarettes. JAMA Network Open 2020;3(6):e206981. 10.1001/jamanetworkopen.2020.6981 PMC7296387 32539148

[69] Blount BC, Karwowski MP, Shields PG, Morel-Espinosa M, Valentin-Blasini L, Gardner M, et al. Vitamin E acetate in bronchoalveolar-lavage fluid associated with EVALI. New England Journal of Medicine 2020;382(8):697-705. 10.1056/NEJMoa1916433 PMC7032996 31860793

[70] Hartnett KP, Kite-Powell A, Patel MT, Haag BL, Sheppard MJ, Dias TP, et al. Syndromic surveillance for e-cigarette, or vaping, product use-associated lung injury. New England Journal of Medicine 2020;382(8):766-72. 10.1056/NEJMsr1915313 PMC10613510 31860794

[71] Hajek P, Etter J-F, Benowitz N, Eissenberg T, McRobbie H. Electronic cigarettes: review of use, content, safety, effects on smokers and potential for harm and benefit. Addiction (Abingdon, England) 2014;109(11):1801-10. 10.1111/add.12659 PMC4487785 25078252

[72] Tobacco Advisory Group of the Royal College of Physicians. Nicotine Without Smoke: Tobacco Harm Reduction. London: Royal College of Physicians, 2016.

[73] National Academies of Sciences, Engineering, & Medicine; Health and Medicine Division; Board on Population Health and Public Health Practice. Public Health Consequences of E-Cigarettes. https://pubmed.ncbi.nlm.nih.gov/29894118/ 2018 (accessed prior to 30 September 2025).

[74] McNeill A, Brose LS, Calder R, Bauld L, Robson D. Vaping in England: an evidence update including vaping for smoking cessation, February 2021: a report commissioned by Public Health England London, UK: Public Health England. assets.publishing.service.gov.uk/government/uploads/system/uploads/attachment_data/file/962221/Vaping_in_England_evidence_update_February_2021.pdf (accessed prior to 3 November 2022).

[75] Royal College of Physicians. E-cigarettes and harm reduction: an evidence review, 2024. https://www.rcp.ac.uk/policy-and-campaigns/policy-documents/e-cigarettes-and-harm-reduction-an-evidence-review (accessed 1 August 2024).

[76] McDonald CF, Jones S, Beckert L, Bonevski B, Buchanan T, Bozier J, et al. Electronic cigarettes: a position statement from the Thoracic Society of Australia and New Zealand. Respirology 2020 [Epub ahead of print]. 10.1111/resp.13904 PMC7540297 32713105

[77] Begh R, Conde M, Fanshawe TR, Kneale D, Shahab L, Zhu S, et al. Electronic cigarettes and subsequent cigarette smoking in young people: a systematic review. Addiction (Abingdon, England) 2025;120(6):1090-111. [DOI: 10.1111/add.16773] PMC12046492 39888213

[78] US Preventive Services Task Force. Interventions for tobacco smoking cessation in adults,including pregnant persons. www.uspreventiveservicestaskforce.org/uspstf/recommendation/tobacco-use-in-adults-and-pregnant-women-counseling-and-interventions (accessed prior to 3 November 2022). 10.1001/jama.2020.25019 33464343

[79] Lindson N, Butler A, McRobbie H, Bullen C, Begh R, Theodoulou A, et al. Update to protocol of the Cochrane review of electronic cigarettes for smoking cessation. Open Science Framework 2023 1-3. [DOI: 10.17605/OSF.IO/ZWGSK]

[80] Brooker J, Synnot A, McDonald S, Elliott J, Turner T, Hodder R, et al. Guidance for the production and publication of Cochrane living systematic reviews: Cochrane Reviews in living mode; December 2019. community.cochrane.org/sites/default/files/uploads/inline-files/Transform/201912_LSR_Revised_Guidance.pdf (accessed 30 July 2020).

[81] Higgins JP, Lasserson T, Thomas J, Flemyng E, Churchill R. Methodological Expectations of Cochrane Intervention Reviews. www.cochrane.org/authors/handbooks-and-manuals/mecir-manual 2021 (accessed prior to 30 September 2025).

[82] Page MJ, McKenzie JE, Bossuyt PM, Boutron I, Hoffmann TC, Mulrow CD, et al. The PRISMA 2020 statement: an updated guideline for reporting systematic reviews. BMJ (Clinical Research Ed.) 2021;372(71):1-9. [DOI: 10.1136/bmj.n71] PMC8005924 33782057

[83] National Institute for Health and Care Excellence. Tobacco: preventing uptake, promoting quitting and treating dependence (guideline). https://www.nice.org.uk/guidance/ng209 2021, updated 2023;NG209(London):UK. 36745727

[84] Lefebvre C, Glanville J, Briscoe S, Featherstone R, Littlewood A, Metzendorf M-I, et al. Technical Supplement to Chapter 4: Searching for and selecting studies [last updated September 2024]. In: Higgins JP, Thomas J, Chandler J, Cumpston M, Li T, Page MJ, et al, editor(s). Cochrane Handbook for Systematic Reviews of Interventions Version 6.5. Cochrane, 2024. Available from https://www.cochrane.org/handbook.

[85] Review Manager Web (RevMan Web). Version 4.12.0. The Cochrane Collaboration, 2022. Available at revman.cochrane.org.

[86] Higgins JP, Green S (editors). Cochrane Handbook for Systematic Reviews of Interventions Version 5.1.0 (updated March 2011). The Cochrane Collaboration, 2011. Available from training.cochrane.org/handbook/archive/v5.1/.

[87] Hartmann-Boyce J, Lindson N. Assessing and minimizing risk of bias in randomized controlled trials of tobacco cessation interventions: guidance from the Cochrane Tobacco Addiction Group. Addiction (Abingdon, England) 2013;118:1811–6. [DOI: 10.1111/add.16220] 37132075

[88] Dawkins L, Bauld L, Ford A, Robson D, Hajek P, Parrott S, et al. A cluster feasibility trial to explore the uptake and use of e-cigarettes versus usual care offered to smokers attending homeless centres in Great Britain. PloS One 2020;15(10):e0240968. 10.1371/journal.pone.0240968 PMC7584191 33095798

[89] ISRCTN14140672. Exploring the use and uptake of e-cigarettes for homeless smokers. www.isrctn.com/ISRCTN14140672 (first received 7 November 2018).

[90] Higgins JP, Thomas J, Chandler J, Cumpston M, Li T, Page MJ, et al (editors). Cochrane Handbook for Systematic Reviews of Interventions version 6.2 (updated February 2021). The Cochrane Collaboration, 2021. Available from www.training.cochrane.org/handbook/archive/v6.2.

[91] Higgins JP, Thompson SG, Deeks JJ, Altman DG. Measuring inconsistency in meta-analyses. BMJ (Clinical Research Ed.) 2003;327(7414):557-60. 10.1136/bmj.327.7414.557 PMC192859 12958120

[92] Theodoulou A, Fanshawe TR, Leavens E, Theodoulou E, Wu AD, Heath L, et al. Differences in the effectiveness of individual-level smoking cessation interventions by socioeconomic status. Cochrane Database of Systematic Reviews 2025, Issue 1. Art. No: CD015120. [DOI: 10.1002/14651858.CD015120.pub2] PMC11770844 39868569

[93] GRADEpro GDT. Version accessed 30 July 2020. Hamilton (ON): McMaster University (developed by Evidence Prime), 2020. Available at gradepro.org.

[94] Avila JC, Maglalang DD, Nollen N, Lee SC, Suh R, Malone M, et al. Using pod based e-cigarettes and nicotine pouches to reduce harm for adults with low socioeconomic status who smoke: a pilot randomized controlled trial. Nicotine & Tobacco Research 2024;26(9):1150-8. [DOI: 10.1093/ntr/ntae047] PMC12104494 38447095

[95] NCT05327439. Using alternative nicotine delivery systems (ANDS) to reduce harm for low SES cigarette smokers (Tri-PEC study). clinicaltrials.gov/ct2/show/NCT05327439 (first received 14 April 2022).

[96] Higgins ST, Sigmon SC, Tidey JW, Heil SH, Gaalema DE, Lee DC, et al. Reduced nicotine cigarettes and e-cigarettes in high-risk populations: 3 randomized clinical trials. JAMA Network Open 2024;7(9):e2431731. [DOI: 10.1001/jamanetworkopen.2024.31731] PMC11380105 39240566

[97] NCT04092387. Low nicotine content cigarettes in vulnerable populations: women of reproductive age. Clinicaltrials.gov/Ct2/Show/NCT04092387 (first received 17 September 2019).

[98] Higgins ST, Tidey J, Sigmon S, Gaalema DE, Lee D, Heil S, et al. Availability of e-cigarettes and flavoured liquids may enhance the effects of a nicotine product standard on smoking in vulnerable populations. In: SRNT 30th Annual Meeting, March 2024, Edinburgh UK. PPS7-2. 2024.

[99] NCT04090879. Low nicotine content cigarettes in vulnerable populations: affective disorders. clinicaltrials.gov/ct2/show/NCT04090879 (first received 16 September 2019).

[100] NCT04092101. Low nicotine content cigarettes in vulnerable populations: opioid use disorder. Clinicaltrials.gov/Ct2/Show/NCT04092101 (first received 17 September 2019).

[101] DeAtley T, Harrison A, Camacho B, Cassidy R, Kuo C, Higgins ST, et al. Subjective experiences, contexts, and risk perceptions of very low content cigarettes and electronic cigarettes among people with affective disorders who smoke. In: Society for Research on Nicotine and Tobacco Annual Meeting, 2022 March; Baltimore USA. 2022.

[102] DeAtley T, Harrison A, Cassidy R, Kuo C, Higgins ST, Tidey JW. Subjective experiences, contexts, and risk perceptions of very low nicotine content cigarettes and electronic cigarettes among people with depression and anxiety disorders who smoke. Drug and Alcohol Dependence 2023;244:109767. 10.1016/j.drugalcdep.2023.109767 PMC9974802 36638679

[103] Hoeppner BB, Eddie D, Schick M, Hoeppner SS, Kelly L, Kelly JF. Feasibility of and reactivity to ecological momentary assessment (EMA) during electronic cigarette use initiation in adults who smoke daily. European Journal of Psychiatry 2024;38(3):100247. [DOI: 10.1016/j.ejpsy.2023.100247] PMC11927963 40124422

[104] Ikonomidis I, Katogiannis K, Kourea K, Kostelli G, Pavlidis G, Thymis J, et al. Differential effects of heat-not-burn, electronic cigarettes and conventional cigarettes on endothelial glycocalyx. European Heart Journal 2024;45(Suppl 1):ehae666.3388. [DOI: 10.1093/eurheartj/ehae666.3388] PMC11195715 39044785

[105] Ikonomidis I, Katogiannis K, Kourea K, Kostelli G, Pavlidis G, Thymis J, et al. Differential effects of heat-not-burn, electronic, and conventional cigarettes on endothelial glycocalyx. European Heart Journal. Imaging Methods & Practice 2023;7(1):1. [DOI: 10.1093/ehjimp/qyad008] PMC11195715 39044785

[106] Kale D, Beard E, Marshall A-M, Pervin J, Wu Q, Ratschen E, et al. Providing an e-cigarette starter kit for smoking cessation and reduction as adjunct to usual care to smokers with a mental health condition: findings from the ESCAPE feasibility study. BMC Psychiatry 2025;25(1):13. [DOI: 10.1186/s12888-024-06387-7] PMC11699696 39754165

[107] ISRCTN17691451. ESCAPE: E-cigarettes for smoking cessation and reduction in people with mental illness [E-cigarettes for smoking cessation and reduction in people with mental illness - a randomised pilot feasibility trial]. isrctn.com/ISRCTN17691451 (first received 30 September 2021). [DOI: 10.1186/ISRCTN17691451]

[108] Katz B, Medina N, O'Connor S, Coleman S, Irvin C, Kaminsky D, et al. Comparison of cardiopulmonary effects of cigarettes and e-cigarettes in individuals with chronic obstructive pulmonary disease (COPD). Drug and Alcohol Dependence 2025;267(Suppl):112271.

[109] NCT05610514. Pulmonary and cardiac effects of e-cigarette use in pulmonary patients who smoke cigarettes. clinicaltrials.gov/ct2/show/NCT05610514 first received 9 November 2022.

[110] Kouroutzoglou A, Ioakeimidis N, Terentes-Printzios D, Tsabras T, Katsi V, Lazaros G, et al. Smoking cessation medicines and e-cigarettes in smokers with obesity. European Heart Journal 2024;45(Suppl 1):ehae666.2955. [DOI: 10.1093/eurheartj/ehae666.2955]

[111] NCT03113136. Examination of low wattage and high wattage e-cigarettes (SWITCH). clinicaltrials.gov/ct2/show/study/NCT03113136 (first received 13 April 2017).

[112] Ganapathy V, Chinnaiyan M, Brobst D, Sadhasivam B, Nshimiyimana JD, Zhao Y, et al. Measuring oral DNA damage to evaluate electronic cigarette use as a tobacco harm reduction strategy. Cancer Research 2024;84(7):LB086. [DOI: 10.1158/1538-7445]

[113] Pericot-Valverde I, Heo M, Nahvi S, Barron J, Voss S, Ortiz EG, et al. Effects of e-cigarettes on combustible cigarette smoking among adults with opioid use disorder on buprenorphine: single arm ERASER pilot trial. Nicotine & Tobacco Research 2025;27(5):865-3. [DOI: 10.1093/ntr/ntae260] PMC13032066 39484988

[114] NCT06277271. E-cigarettes as a harm reduction strategy. https://clinicaltrials.gov/study/NCT06277271 (first received 19 January 2024).

[115] Rabenstein A, Czermak L, Fischer E, Kahnert K, Pogarell O, Jorres RA, et al. Implications of switching from conventional to electronic cigarettes on quality of life and smoking behaviour: results from the EQualLife Trial. European Addiction Research 2024;30(4):207-15. [DOI: 10.1159/000536255] 38626733

[116] Sifat MS, Alexander AC, Businelle MS, Frank-Pearce SG, Boozary LK, Wagener TL, et al. E-Cigarette switching and financial incentives to promote combustible cigarette cessation among adults accessing shelter services: a pilot study. Drug and Alcohol Dependence Reports 2024;13:1-9. [DOI: 10.1016/j.dadr.2024.100295] PMC11702002 39764385

[117] Smith TT, Crawford A, Wahlquist AE, Cummings KM, Rojewski AM, McClure EA, et al. Pilot study to test the feasibility for a randomized controlled trial of e-cigarettes as harm reduction tools among people who smoke and previously failed to quit with pharmacotherapy. Nicotine & Tobacco Research 2025;27(3):553-7. [DOI: 10.1093/ntr/ntae212] 39233579

[118] Smith TT, Wahlquist AE, Cummings KM, Rojewski AM, McClure EA, Toll BA, et al. Pilot investigation of e-cigarettes as a harm reduction tool among smokers who previously failed to quit with pharmacotherapy. In: SRNT 30th Annual Meeting, March 2024, Edinburgh, UK. 2024:PPS25-2A.

[119] NCT05525078. Quit or switch: e-cigarette study. clinicaltrials.gov/ct2/show/NCT05525078 (first received 1 September 2022).

[120] Tuisku A, Rahkola M, Nieminen P, Toljamo T. Electronic cigcarettes vs varenicline for smoking cessation in adults: a randomized clinical trial. JAMA Internal Medicine 2024;184(8):915-21. [DOI: 10.1001/jamainternmed.2024.1822] PMC11184496 38884987

[121] Tuisku A, Toljamo T, Nieminen P. Relevant data missing in electronic cigarette vs varenicline trial - reply. JAMA Internal Medicine 2024;184(12):1483. 10.1001/jamainternmed.2024.5196 39432275

[122] Vojjala M, Stevens ER, Nicholson A, Morgan T, Kaneria A, Xiang G, et al. Switching to e-cigarettes as harm reduction among individuals with chronic disease who currently smoke: results of a pilot randomized controlled trial. Nicotine & Tobacco Research 2025;27(1):36-45. [DOI: 10.1093/ntr/ntae158] 38995184

[123] Vojjala M, Wilker OG, Nicholson A, Morgan T, Rozon I, Stevens ER, et al. Electronic cigarettes and counseling as a harm reduction strategy among patients with COPD, asthma, CAD/PAD. In: SRNT 29th Annual Meeting; 2023 Mar 1-4; San Antonio (TX), USA. 2023:206 (POS4-100).

[124] Vojjala M, Stevens ER, Nicholson A, Morgan T, Kaneria A, Xiang G, et al. Switching to e-cigarettes as a harm reduction tool among individuals with chronic disease who currently smoke: results of a pilot randomised controlled trial. In: SRNT 30th Annual Meeting, 12-15 March 2025, Edinburgh, UK. 2024:POS5-147. 10.1093/ntr/ntae158 38995184

[125] Stevens ER, Lei L, Cleland CM, Berger KM, Sherman SE, Vojjala M. Electronic cigarettes as a harm reduction strategy among patients with COPD: protocol for an open-label two arm randomized controlled pilot trial. Addiction Science & Clinical Practice 2022;17(1):2. [DOI: 10.1186/s13722-021-00284-0] PMC8734340 34991693

[126] NCT04465318. Electronic cigarettes as a harm reduction strategy among patients with COPD. clinicaltrials.gov/ct2/show/NCT04465318 (first received 10 July 2020).

[127] Czoli CD, Fong GT, Goniewicz ML, Hammond D. Biomarkers of exposure among "dual users" of tobacco cigarettes and electronic cigarettes in Canada. Nicotine & Tobacco Research 2019;21(9):1259-66. 10.1093/ntr/nty174 PMC6698946 30203076

[128] Martinez U, Simmons VN, Sutton SK, Drobes DJ, Meltzer LR, Brandon KO, et al. Targeted smoking cessation for dual users of combustible and electronic cigarettes: a randomised controlled trial. Lancet. Public Health 2021;6(7):e500-9. 10.1016/S2468-2667(20)30307-8 PMC8281505 34175001

[129] Byrne M, Simmons V, Martinez U, Sutton S, Brandon K, Brandon T. Cost effectiveness of a smoking cessation intervention for dual users of combustible and electronic cigarettes. In: Society for Research on Nicotine and Tobacco, Annual Meeting, March 2022, Baltimore USA. 2022.

[130] Martinez U, Simmons VN, Brandon KO, Quinn GP, Brandon TH. Examining smoking and vaping behaviors, expectancies, and cessation outcomes between bisexual and heterosexual individuals. Behavioral Medicine (Washington, D.C.) 2023;49(4):392-401. [DOI: 10.1080/08964289.2022.2077295] PMC9691792 35614523

[131] NCT02416011. Smoking cessation self-help for dual users of tobacco cigarettes and e-cigarettes. clinicaltrials.gov/ct2/show/NCT02416011 (first received 18 August 2020).

[132] Sutton SK, Brandon KO, Harrell PT, Martínez Ú, Simmons VN, Gore LR, et al. Identifying prospective subpopulations of combustible and electronic cigarette dual users in the United States via finite mixture modeling. Addiction (Abingdon, England) 2022;117(9):2493-503. [DOI: 10.1111/add.15906] PMC9795793 35491736

[133] Meltzer LR, Simmons VN, Sutton SK, Drobes DJ, Quinn GP, Meade CD, et al. A randomized controlled trial of a smoking cessation self-help intervention for dual users of tobacco cigarettes and e-cigarettes: intervention development and research design. Contemporary Clinical Trials 2017;60:56-62. 10.1016/j.cct.2017.06.014 PMC5559662 28648969

[134] Gore LR, Sutton SK, Martinez U, Brandon KO, Simmons VN, Brandon TH. Mediators of initial abstinence for an extended self help smoking cessation intervention with dual users of combustible and electronic cigarettes. In: Society for Research on Nicotine and Tobacco, Annual Meeting, March 2022, Baltimore USA. PS3-7. 2022.

[135] Vickerman KA, Carpenter KM, Miles LN, Hsu JM, Watt KA, Brandon TH, et al. A randomized pilot of a tailored smoking cessation quitline intervention for individuals who smoke and vape. Nicotine & Tobacco Research 2022;24(11):1811-20. [DOI: 10.1093/ntr/ntac129] PMC9596999 35575085

[136] NCT03575468. Enhanced e-cigarette coaching intervention for dual users of cigarettes and e-cigarettes. clinicaltrials.gov/ct2/show/NCT03575468 (first received 2 July 2018).

[137] Vickerman KA, Carpenter KM, Watt K, Miles L, Hsu J, Brandon T, et al. A behavioral smoking cessation intervention for Quitline callers who also use e-cigarettes. In: Society for Research on Nicotine and Tobacco (SRNT) 2021 Annual Meeting; February 24-27 2021 (virtual). 2021:50 (POD14-1).

[138] Elling JM, Crutzen R, Talhout R, De Vries H. Effects of providing tailored information about e-cigarettes in a digital smoking cessation intervention: randomized controlled trial. Health Education Research 2023;38(2):150-62. [DOI: 10.1093/her/cyad004] PMC10035064 36727168

[139] Elling JM, Crutzen R, Talhout R, De Vries H. Effects of providing tailored information about e-cigarettes in a web-based smoking cessation intervention: protocol for a randomised control trial. Journal of Medical Internet Research, Research Protocols 2021;10(5):e27088. [DOI: 10.2196/27088] PMC8164120 33988520

[140] NL8330. Communication about e-cigarettes in a digital smoking cessation intervention [Effect of communication about e-cigarettes in a digital smoking cessation intervention on smoking reduction, smoking cessation, and informed decision making]. trialsearch.who.int/?TrialID=NL8330 (first received 24 January 2020).

[141] Lee SH, Ahn SH, Cheong YS. Effect of electronic cigarettes on smoking reduction and cessation in Korean male smokers: a randomized controlled study. Journal of the American Board of Family Medicine 2019;32(4):567-74. 10.3122/jabfm.2019.04.180384 31300577

[142] KCT0001277. Effect of an electronic cigarette for smoking reduction and cessation in Korean male smokers: a randomized, controlled study. https://cris.nih.go.kr/cris/search/detailSearchEn.do?seq=KCT0001277 (first received 2014). 10.3122/jabfm.2019.04.180384 31300577

[143] Van Staden SR, Groenewald M, Engelbrecht R, Becker PJ, Hazelhurst LT. Carboxyhaemoglobin levels, health and lifestyle perceptions in smokers converting from tobacco cigarettes to electronic cigarettes. South African Medical Journal 2013;103(11):865-8. 10.7196/samj.6887 24148175

[144] Caponnetto P, Campagna D, Cibella F, Morjaria JB, Caruso M, Russo C, et al. EffiCiency and safety of an eLectronic cigAreTte (ECLAT) as tobacco cigarettes substitute: a prospective 12-month randomized control design study. PloS One 2013;8(6):e66317. 10.1371/journal.pone.0066317 PMC3691171 23826093

[145] Farsalinos K, Cibella F, Caponnetto P, Campagna D, Morjaria JB, Battaglia E, et al. Effect of continuous smoking reduction and abstinence on blood pressure and heart rate in smokers switching to electronic cigarettes. Internal and Emergency Medicine 2016;11(1):85-94. 10.1007/s11739-015-1361-y PMC4747988 26749533

[146] NCT01164072. Efficacy and safety of an electronic nicotine delivery device (e-cigarette). clinicaltrials.gov/ct2/show/NCT01164072 (first received 16 July 2010).

[147] NCT01194583. Efficacy and safety of an electronic nicotine delivery device (e-cigarette) without nicotine cartridges. clinicaltrials.gov/ct2/show/NCT01194583 (first received 3 September 2010).

[148] Cibella F, Campagna D, Caponnetto P, Amaradio MD, Caruso M, Russo C, et al. Lung function and respiratory symptoms in a randomized smoking cessation trial of electronic cigarettes. Clinical Science 2016;130(21):1929-37. 10.1042/CS20160268 27543458

[149] Russo C, Cibella F, Caponnetto P, Campagna D, Maglia M, Frazzetto E, et al. Evaluation of post cessation weight gain in a 1-year randomized smoking cessation trial of electronic cigarettes. Scientific Reports 2016;6:18763. 10.1038/srep18763 PMC4700433 26729619

[150] Campagna D, Cibella F, Caponnetto P, Amaradio MD, Caruso M, Morjaria JB, et al. Changes in breathomics from a 1-year randomized smoking cessation trial of electronic cigarettes. European Journal of Clinical Investigation 2016;46(8):698-706. 10.1111/eci.12651 27322745

[151] NCT01188239. A structured protocol to evaluate efficacy and safety of a popular electronic nicotine delivery device (e-cigarette) efficacy and safety of a popular electronic nicotine delivery device (e-cigarette). https://clinicaltrials.gov/ct2/show/NCT01188239 (first received 25 AUgust 2010).

[152] Cobb CO, Foulds J, Yen M-S, Veldheer S, Lopez AA, Yingst JM, et al. Effect of an electronic nicotine delivery system with 0, 8, or 36 mg/mL liquid nicotine versus a cigarette substitute on tobacco-related toxicant exposure: a four-arm, parallel-group, randomised, controlled trial. Lancet Respiratory Medicine 2021;9(8):840-50. [DOI: 10.1016/ S2213-2600(21)00022-9] 10.1016/S2213-2600(21)00022-9 PMC8349799 33857436

[153] Foulds J, Cobb C, Yen M-S, Veldheer S, Brosnan P, Yingst J, et al. Effect of electronic nicotine delivery systems on cigarette abstinence in smokers with no plans to quit: exploratory analysis of a randomized placebo-controlled trial. Nicotine Tobacco Research 2022;24(7):955-61. [DOI: 10.1093/ntr/ntab247] PMC9391685 34850164

[154] Dahal S, Yingst J, Wang X, Cobb CO, Carrillo M, Hrabovsky S, et al. Changes in cardiovascular disease risk, lung function, and other clinical health outcomes when people who smoke use e-cigarettes to reduce cigarette smoking: an exploratory analysis from a randomized placebo-controlled trial. MedRxiv: the Preprint Server for Health Sciences 2024. [DOI: 10.1101/2024.12.14.24319048] PMC12182122 40533223

[155] Yingst J, Wang X, Anderson A, Breland A, Soule E, Barnes A, et al. Evaluation of nicotine dependence among smokers using electronic cigarettes to reduce cigarette smoke. In: Society for Research on Nicotine and Tobacco, Annual Meeting, March 2022, Baltimore USA. 2022.

[156] Lopez AA, Cobb CO, Yingst JM, Veldheer S, Hrabovsky S, Yen MS, et al. A transdisciplinary model to inform randomized clinical trial methods for electronic cigarette evaluation. BMC Public Health 2016;16(1):217. [PMCID:: PMC4778292] [PMID: 26941050] 10.1186/s12889-016-2792-8 PMC4778292

[157] NCT02342795. Randomized controlled trial methods for novel tobacco products evaluation. clinicaltrials.gov/show/NCT02342795 (first received 16 December 2014).

[158] Veldheer S, Yingst J, Midya V, Hummer B, Lester C, Krebs N. Pulmonary and other health effects of electronic cigarette use among adult smokers participating in a randomized controlled smoking reduction trial. Addictive Behaviors 2019;91:95-101. 10.1016/j.addbeh.2018.10.041 PMC6358505 30393015

[159] Yingst J, Wang Xi, Lopez AA, Breland A, Soule E, Barnes A, et al. Randomized control trial methods workgroup of the center for the study of tobacco products, changes in nicotine dependence among smokers using electronic cigarettes to reduce cigarette smoking in a randomized controlled trial. Nicotine & Tobacco Research 2023;25(3):372-8. [DOI: 10.1093/ntr/ntac153] PMC9910150 35752091

[160] Dahal S, Yingst J, Wang X, Cobb C, Hrabovsky S, Bascom R, et al. Changes in cardiopulmonary and other clinical health outcomes among adult smokers using electronic cigarettes: an exploratory analysis from a randomized controlled smoking reduction trial. In: SRNT Rapids 30th Annual Meeting, March 2024, New Orleans USA. POS5-5. 2024.

[161] Yingst J, Foulds J, Veldheer S, Cobb CO, Yen M, Hrabovsky S, et al. Measurement of electronic cigarette frequency of use among smokers participating in a randomized controlled trial. Nicotine & Tobacco Research 2020;22(5):699-704. 10.1093/ntr/nty233 PMC7171268 30365024

[162] Yingst J, Midya V, White A, Foulds J, Cobb CO, Veldheer S, et al. Effects of liquid nicotine concentration and flavour on the acceptability of electronic nicotine delivery systems (ENDS) among people who smoke participating in a randomised controlled trial to reduce cigarette consumption. Tobacco Control 2025;34(4):496-505. [DOI: 10.1136/tc-2023-058282] PMC11408707 38471776

[163] Kanobe MN, Jones BA, Nelson P, Brown BG, Chen P, Makena P, et al. Part three: a randomized study to assess biomarker changes in cigarette smokers switched to Vuse Solo or abstinence. Scientific Reports 2022;12:20658. [DOI: 10.1038/s41598-022-25054-z] PMC9712618 36450821

[164] Kanobe MN, Nelson PR, Brown BG, Chen P, Patrudu M, Caraway JW, et al. Changes in biomarkers of exposure and potential harm in smokers switched to Vuse Vibe or Vuse Ciro electronic nicotine delivery systems. Toxics 2023;11(7):564. [DOI: 10.3390/toxics11070564] PMC10384956 37505530

[165] NCT03170674. CSD170501: Study to assess biomarkers of tobacco exposure in smokers during in-clinic confinement switch to an electronic cigarette. clinicaltrials.gov/ct2/show/NCT03170674 (first received 31 May 2017).

[166] Kimber CF, Soar K, Dawkins LE. Changes in puffing topography and subjective effects over a 2-week period in e-cigarette naive smokers: effects of device type and nicotine concentrations. Addictive Behaviors 2021;118:106909. [DOI: 10.1016/j.addbeh.2021.106909] 33756301

[167] Morris P, McDermott S, Chapman F, Verron T, Cahours X, Stevenson M, et al. Reductions in biomarkers of exposure to selected harmful and potentially harmful constituents following exclusive and partial switching from combustible cigarettes to myblu TM electronic nicotine delivery systems (ENDS). Internal and Emergency Medicine 2022;17(2):397-410. [DOI: 10.1007/s11739-021-02813-w] PMC8964552 34435305

[168] NCT04429932. A study to evaluate nicotine uptake and biomarkers in adult smokers using mybluTM electronic cigarettes. clinicaltrials.gov/ct2/show/NCT04429932 (first received 7 June 2021).

[169] NCT04430634. A study to evaluate nicotine uptake and biomarkers in smokers using mybluTM electronic cigarettes [An open-label, randomized, crossover study to assess nicotine uptake, tobacco-related biomarkers of exposure, biomarkers of potential harm, and puff topography with use of mybluTM electronic cigarettes in adult smokers]. Clinicaltrials.gov/Ct2/Show/NCT04430634 (first received 7 June 2021).

[170] White CM, Tessier KM, Koopmeiners JS, Denlinger-Apte RL, Cobb CO, Lane T, et al. Preliminary evidence on cigarette nicotine reduction with concurrent access to an e-cigarette: manipulating cigarette nicotine content, e-liquid nicotine content, and e-liquid flavor availability. Preventive Medicine 2022;165(Pt B):107213. [DOI: 10.1016/j.ypmed.2022.107213] PMC10080461 35995103

[171] NCT03185546. Project 2 cigarette and e-cigarette nicotine content and e-liquid flavors. clinicaltrials.gov/ct2/show/NCT03185546 (first received 14 May 2021).

[172] White CM, Denlinger-Apte RL, Tessier KM, Koopmeiners JS, Hatsukami DK, Strasser AA. Cigarette nicotine reduction in the presence of an alternative: investigating how cigarette nicotine content and e-liquid content affect smoking. In: Society for Research on Nicotine and Tobacco (SRNT) 2021 Annual Meeting, February 24-27 2021 (virtual). 2021:2-3 (SYM2C).

[173] Edmiston JS, Webb KM, Wang J, Oliveri D, Liang Q, Sarkar M. Biomarkers of exposure and biomarkers of potential harm in adult smokers who switch to e-vapor products relative to cigarette smoking in a 24-week, randomized, clinical trial. Nicotine & Tobacco Research 2022;24(7):1047–54. [DOI: 10.1093/ntr/ntac029] PMC9199942 35134961

[174] Xu Y, Goldenson NI, Prakash S, Augustson EM, Shiffman S. Randomized trial assessing the effect of the JUUL system on switching away from cigarettes and smoking reduction among U.S. adults who smoke cigarettes. Experimental and Clinical Psychopharmacology 2024;32(1):3-15. [DOI: 10.1037/pha0000698] 38127516

[175] Yingst J, Foulds J, Zurlo J, Steinberg MB, Eissenberg T, Du P. Acceptability of electronic nicotine delivery systems (ENDS) among HIV positive smokers. AIDS Care 2020;32(10):1224-8. 10.1080/09540121.2019.1687835 PMC7202989 31698920

[176] Russell C, McKeganey N, Katsampouris E, Satchwell A, Haseen F. A randomised community-based trial of a closed-system pod e-vapour product and nicotine replacement therapy for cigarette abstinence and reduction. In: Society for Research on Nicotine and Tobacco (SRNT) 2021 Annual Meeting, February 24-27 2021, virtual. 2021:230 (PH-353).

[177] Skelton E, Lum A, Robinson M, Dunlop A, Guillaumier A, Baker A, et al. A pilot randomised controlled trial of abrupt versus gradual smoking cessation in combination with vaporised nicotine products for people receiving alcohol and other drug treatment. Addictive Behaviors 2022;131:107328. [DOI: 10.1016/j.addbeh.2022.107328] 35405479

[178] ACTRN12617000905369. Smoking reduction interventions for smoking cessation. ncbi.nlm.nih.gov/pmc/articles/PMC6953262/ (first received 9 June 2017). [DOI: 10.1002/14651858.CD013183.pub2]

[179] Lum A, Skelton E, Robinson M, Guillaumier A, Wynne O, Gartner C, et al. Barriers and facilitators to using vaporised nicotine products as smoking cessation aids among people receiving treatment for substance use disorder. Addictive Behaviors 2021;124:107097. [DOI: 10.1016/j.addbeh.2021.107097] 34536632

[180] Caponnetto P, Auditore R, Russo C, Cappello GC, Polosa R. Impact of an electronic cigarette on smoking reduction and cessation in schizophrenic smokers: a prospective 12-month pilot study. International Journal of Environmental Research and Public Health 2013;10(2):446-61. 10.3390/ijerph10020446 PMC3635154 23358230

[181] Minutolo G, Caponnetto P, Auditore R, Russo C, Polosa R. Management of smoking reduction and cessation in inpatients with schizophrenia: impact of electronic cigarettes. European Neuropsychopharmacology 2013;23:S581-2.

[182] Caponnetto P, DiPiazza J, Kim J, Maglia M, Polosa R. A single-arm, open-label pilot, and feasibility study of a high nicotine strength e-cigarette intervention for smoking cessation or reduction for people with schizophrenia spectrum disorders who smoke cigarettes. Nicotine & Tobacco Research 2021;23(7):1113-22. [DOI: 10.1093/ntr/ntab005] PMC8186418 33723598

[183] Polosa R, Caponnetto P, Morjaria JB, Papale G, Campagna D, Russo C. Effect of an electronic nicotine delivery device (e-cigarette) on smoking reduction and cessation: a prospective 6-month pilot study. BMC Public Health 2011;11:786. 10.1186/1471-2458-11-786 PMC3203079 21989407

[184] NCT01195597. Smoking cessation and reduction with an electronic nicotine delivery device (ENDD). clinicaltrials.gov/ct2/show/NCT01195597 (first received 6 September 2010).

[185] Polosa R, Morjaria JB, Caponnetto P, Campagna D, Russo C, Alamo A, et al. Effectiveness and tolerability of electronic cigarette in real-life: a 24-month prospective observational study. Internal and Emergency Medicine 2014;9(5):537-46. 10.1007/s11739-013-0977-z 23873169

[186] Polosa R, Caponnetto P, Maglia M, Morjaria JB, Russo C. Success rates with nicotine personal vaporizers: a prospective 6-month pilot study of smokers not intending to quit. BMC Public Health 2014;14:1159. 10.1186/1471-2458-14-1159 PMC4247211 25380748

[187] NCT02124200. High cessation rates in smokers using personal vaporizers (VAPECIG). clinicaltrials.gov/ct2/show/NCT02124200 (first received 28 April 2014).

[188] Polosa R, Caponnetto P, Cibella F, Le-Houezec J. Quit and smoking reduction rates in vape shop consumers: a prospective 12-month survey. International Journal of Environmental Research and Public Health 2015;12(4):3428-38. 10.3390/ijerph120403428 PMC4410194 25811767

[189] Caponnetto P, Campagna D, Maglia M, Benfatto F, Emma R, Caruso M, et al. Comparing the effectiveness, tolerability, and acceptability of heated tobacco products and refillable electronic cigarettes for cigarette substitution (CEASEFIRE): randomized controlled trial. Public Health Surveillance 2023;9:e42628. [DOI: 10.2196/42628] PMC10131829 37014673

[190] NCT03569748. Heated tobacco products vs electronic cigarettes. clinicaltrials.gov/ct2/show/NCT03569748 (first received 25 June 2021).

[191] Caponnetto P, Caruso M, Maglia M, Emma R, Saitta D, Busa B, et al. Non-inferiority trial comparing cigarette consumption, adoption rates, acceptability, tolerability, and tobacco harm reduction potential in smokers switching to Heated Tobacco Products or electronic cigarettes: study protocol for a randomized controlled trial. Contemporary Clinical Trials Communications 2020;8:17. 10.1016/j.conctc.2020.100518 PMC6962654 31956726

[192] Nides MA, Leischow SJ, Bhatter M, Simmons M. Nicotine blood levels and short-term smoking reduction with an electronic nicotine delivery system. American Journal of Health Behavior 2014;38(2):265-74. 10.5993/AJHB.38.2.12 24629555

[193] NCT01898169. Evaluation of short-term safety and use patterns of an electronic nicotine delivery system. clinicaltrials.gov/ct2/show/NCT01898169 (first received 12 July 2013).

[194] Rose JE, Frisbee S, Campbell D, Salley A, Claerhout S, Davis JM. Smoking reduction using electronic nicotine delivery systems in combination with nicotine skin patches. Psychopharmacology 2023;240:1901-9. [DOI: 10.1007/s00213-023-06401-y] PMC10471641 37458789

[195] NCT03492463. The role of nicotine dose and route of delivery in affecting adoption of e-cigarettes and reducing exposure to toxic combustion products (ENDS-Switch). clinicaltrials.gov/ct2/show/study/NCT03492463 (first received 10 April 2018).

[196] Rose JE, Frisbee S, Campbell D, Salley A, Claerhout S, Davis JM. Correction to: Smoking reduction using electronic nicotine delivery systems in combination with nicotine skin patch. Psychopharmacology 2025;Advance online publication:no pagination. [PMID: 10.1007/s00213-025-06868-x] PMC12675545 40736541

[197] Walele T, Bush J, Koch A, Savioz R, Martin C, O'Connell G. Evaluation of the safety profile of an electronic vapour product used for two years by smokers in a real-life setting. Regulatory Toxicology and Pharmacology 2018;92:226-38. [DOI: 10.1016/j.yrtph.2017.12.010] 29248487

[198] NCT02029196. A randomised, parallel group, multi-centre study to evaluate the safety profile of the ITG EVP G1 product. clinicaltrials.gov/show/NCT02029196 (first received 19 December 2013).

[199] NCT02143310. A multi-centre study to evaluate the safety of use of electronic vapour products for two years. clinicaltrials.gov/show/NCT02143310 (first received 9 May 2014).

[200] Cravo AS, Bush J, Sharma G, Savioz R, Martin C, Craige S, et al. A randomised, parallel group study to evaluate the safety profile of an electronic vapour product over 12 weeks. Regulatory Toxicology and Pharmacology 2016;81:S1-S14. 10.1016/j.yrtph.2016.10.003 27769828

[201] Bullen C, Howe C, Laugesen M, McRobbie H, Parag V, Williman J, et al. Electronic cigarettes for smoking cessation: a randomised controlled trial. Lancet 2013;382(9905):1629-37. 10.1016/S0140-6736(13)61842-5 24029165

[202] Bullen C, Howe C, Laugesen M, McRobbie H, Parag V, Williman J, et al. Electronic cigarettes and smoking cessation: a quandary? - Authors' reply. Lancet 2014;383(9915):408-9. 10.1016/S0140-6736(14)60146-X 24485579

[203] Bullen C, Howe C, Laugesen M, McRobbie H, Parag V, Williman J. Do electronic cigarettes help smokers quit? Results from a randomised controlled trial [Abstract]. In: European Respiratory Society Annual Congress, 2013 September 7-11, Barcelona, Spain. Vol. 42. 2013:215s-[P1047].

[204] Bullen C, Williman J, Howe C, Laugesen M, McRobbie H, Parag V, et al. Study protocol for a randomised controlled trial of electronic cigarettes versus nicotine patch for smoking cessation. BMC Public Health 2013;13:210. 10.1186/1471-2458-13-210 PMC3602285 23496861

[205] O'Brien B, Knight-West O, Walker N, Parag V, Bullen C. E-cigarettes versus NRT for smoking reduction or cessation in people with mental illness: secondary analysis of data from the ASCEND trial. Tobacco Induced Diseases 2015;13(1):5. 10.1186/s12971-015-0030-2 PMC4374189 25814920

[206] Eisenberg MJ, Hébert-Losier M, Windle SB, Greenspoon T, Brandys T, Fülöp T. Effect of e-cigarettes plus counseling vs counseling alone on smoking cessation: a randomized clinical trial. JAMA 2020;324(18):1844-54. 10.1001/jama.2020.18889 PMC7656286 33170240

[207] Lyzwinski L, Dong M, Wolfinger RD, Filion KB, Eisenberg MJ. E-cigarettes, smoking cessation, and weight change: retrospective secondary analysis of the evaluating the efficacy of e-cigarette use for smoking cessation trial. JMIR Public Health and Surveillance 2024;10:e58260. [DOI: 10.2196/58260] PMC11443201 39283667

[208] Prell C, Hebert-Losier A, Filion KB, Reynier P, Eisenberg MJ. Evaluating the impact of varying expired carbon monoxide thresholds on smoking relapse identification: insights from the E3 trial on e-cigarette efficacy for smoking cessation. BMJ Open 2023;13(13):10. [DOI: 10.1136/bmjopen-2022-071099] PMC10583027 37832989

[209] Hebert-Losier A, Filion KB, Windle SB, Eisenberg MJ. A randomized controlled trial evaluating the efficacy of e-cigarette use for smoking cessation in the general population: E3 trial design. Canadian Journal of Cardiology Open 2020;2(3):168-75. 10.1016/j.cjco.2020.03.006 PMC7242503 32462131

[210] NCT02417467. Evaluating the efficacy of e-cigarette use for smoking cessation (E3) trial. clinicaltrials.gov/show/NCT02417467 (first received 1 April 2015).

[211] Hajek P, Phillips-Waller A, Przulj D, Pesola F, Myers Smith K, Bisal N, et al. A randomized trial of e-cigarettes versus nicotine-replacement therapy. New England Journal of Medicine 2019;380(7):629-37. 10.1056/NEJMoa1808779 30699054

[212] ISRCTN60477608. The efficacy of e-cigarettes compared with nicotine replacement therapy, when used within the UK stop smoking service. www.isrctn.com/ISRCTN60477608 (first received 2 April 2015).

[213] Li J, Hajek P, Pesola F, Wu Q, Phillips-Waller A, Przulj D, et al. Cost-effectiveness of e-cigarettes compared with nicotine replacement therapy in stop smoking services in England (TEC study): a randomized controlled trial. Addiction (Abingdon, England) 2020;115(3):507-17. 10.1111/add.14829 PMC7318206 31597207

[214] Hajek P, Phillips-Waller A, Przulj D, Pesola F, Smith KM, Bisal N, et al. E-cigarettes compared with nicotine replacement therapy within the UK Stop Smoking Services: the TEC RCT. Health Technology Assessment 2019;23(43):1-82. 10.3310/hta23430 PMC6732716 31434605

[215] Hajek P, Przulj D, Pesola F, Griffiths C, Walton R, McRobbie H, et al. Electronic cigarettes versus nicotine patches for smoking cessation in pregnancy: a randomized controlled trial. Nature Medicine 2022;28:958-64. [DOI: 10.1038/s41591-022-01808-0] PMC9117131 35577966

[216] Przulj D, Pesola F, Smith KM, McRobbie H, Coleman T, Lewis S, et al. Helping pregnant smokers quit: a multicentre randomised controlled trial of electronic cigarettes versus nicotine replacement therapy. Health Technology Assessment 2023;27(13):1-53. [DOI: 10.3310/AGTH6901] PMC10599072 37840301

[217] Pesola F, Smith KM, Phillips-Waller A, Przulj D, Griffiths C, Walton R, et al. Safety of e-cigarettes and nicotine patches as stop-smoking aids in pregnancy: secondary analysis of the Pregnancy Trial of E-cigarettes and Patches (PREP) randomized controlled trial. Addiction (Abingdon, England) 2024;119(5):875-84. [DOI: 10.1111/add.16422] 38229538

[218] Ford A, Uny I, Lowes J, Naughton F, Cooper S, Coleman T. A qualitative study of factors influencing adherence among pregnant women taking part in a trial of e-cigarettes for smoking cessation. International Journal of Environmental Research and Public Health 2021;18(2):430. 10.3390/ijerph18020430 PMC7827544 33430407

[219] ISRCTN62025374. Helping pregnant smokers quit: a multi-centre study of electronic cigarettes and nicotine patches. www.isrctn.com/ISRCTN62025374 (first received 21 March 2017). [DOI: 10.1186/ISRCTN62025374]

[220] Hajek P, Przulj D, Pesola F, Griffiths C, Walton R, McRobbie H, et al. Author Correction: Electronic cigarettes versus nicotine patches for smoking cessation in pregnancy: a randomized controlled trial. Nature Medicine 2023;29(11):2957. [DOI: 10.1038/s41591-022-02099-1] PMC10667089 36344702

[221] Kerr D. Chapter 5: A randomised controlled trial investigating the cardiovascular effects of e-cigaretttes in comparison to nicotine replacement patches. In: Studies into the Cardiovascular and Respiratory Effects of Electronic Cigarettes (PhD Thesis). Publisher not available, 27 September 2020.

[222] NCT03358953. The cardiovascular impacts of electronic cigarettes in comparison to the use of nicotine replacement patches (VAPOUR). clinicaltrials.gov/ct2/show/NCT03358953 (first received 2 December 2017).

[223] Lee SM, Tenney R, Wallace AW, Arjomandi M. E-cigarettes versus nicotine patches for perioperative smoking cessation: a pilot randomized trial. PeerJ 2018;6(9):e5609. 10.7717/peerj.5609 PMC6166615 30280019

[224] NCT02482233. A pilot randomized controlled clinical trial - "Electronic nicotine delivery device (e-cigarette) for perioperative smoking cessation in veterans". clinicaltrials.gov/show/NCT02482233 (first received 17 June 2015).

[225] Lee SM, Tenney R, Wallace A, Arjojmandi M. The end perioperative smoking pilot study: a randomized trial comparing e-cigarettes versus nicotine patch. Canadian Journal of Anesthesia 2017;64(1 Suppl 1):S48-9.

[226] Lee SM, Tenney R, Wallace A, Arjomandi M. Exploring the teachable moment: facilitators and barriers to perioperative smoking cessation, a qualitative study. Canadian Journal of Anesthesia (Conference: 2017 Annual Meeting of the Canadian Anesthesiologists' Society, Canada) 2017;61(Suppl 1):S46-7.

[227] Myers Smith K, Phillips-Waller A, Pesola F, McRobbie H, Przulj D, Orzol M, et al. E-cigarettes versus nicotine replacement treatment as harm reduction interventions for smokers who find quitting difficult: randomised controlled trial. Addiction (Abingdon, England) 2022;117(1):224-33. [DOI: 10.1111/add.15628] 34187081

[228] ISRCTN13288677. Can electronic cigarettes and nicotine replacement treatment help reduce smoking in smokers who struggle to quit? www.isrctn.com/ISRCTN13288677 (first received 17 March 2017).

[229] Ioakeimidis N, Vlachopoulos C, Georgakopoulos C, Abdelrasoul M, Skliros N, Katsi V, et al. Smoking cessation rates with varenicline and electronic cigarettes in relapsed smokers with a history of acute coronary syndrome. European Heart Journal 2018;39(Suppl 1):242.

[230] Klonizakis M, Gumber A, McIntosh E, Brose LS. Medium-and longer-term cardiovascular effects of e-cigarettes in adults making a stop-smoking attempt: a randomized controlled trial. BMC Medicine 2022;20(1):276. [DOI: 10.1186/s12916-022-02451-9] PMC9380327 35971150

[231] NCT03061253. E-cigarettes and cardiovascular function (ISME-NRT). clinicaltrials.gov/ct2/show/NCT03061253 (first received 23 February 2017).

[232] Klonizakis M, Gumber A, McIntosh E, Brose LS. Short-term cardiovascular effects of e-cigarettes in adults making a stop-smoking attempt: a randomized controlled trial. Biology 2021;10(11):1208. [DOI: 10.3390/biology10111208] PMC8614829 34827200

[233] Klonizakis M, Crank H, Gumber A, Brose LS. Smokers making a quit attempt using e-cigarettes with or without nicotine or prescription nicotine replacement therapy: impact on cardiovascular function (ISME-NRT) - a study protocol. BMC Public Health 2017;17(1):293. 10.1186/s12889-017-4206-y PMC5379660 28376818

[234] Jones G, McIntosh E, Brose LS, Klonizakis M. Participant experiences of a quit smoking attempt through either Nicotine Replacement Therapy (NRT) methods or the use of an e-cigarette. Journal of Addiction Medicine 2022;16(3):272-7. 10.1097/ADM.0000000000000881 34128486

[235] Wagener T, Beebe L, Businelle M, Carpenter M, Hinton A, Hart J, et al. E-cigarette versus combination nicotine replacement therapy delivered through state quitlines on smoking abstinence following a recent failed quit attempt: a randomized trial. In: Society for Research on Nicotine and Tobacco (SRNT) 29th Annual Meeting; 2023 Mar 1-4; San Antonio (TX), USA. 2023:SYM5-2.

[236] Piper ME, Schlam TR, Donny EC, Kobinsky K, Matthews J, Piasecki TM, et al. The impact of three alternate nicotine-delivery products on combusted cigarette use: a randomized controlled trial. Nicotine & Tobacco Research 2025;27(2):317-25. [DOI: 10.1093/ntr/ntae014] PMC11750741 38348917

[237] Piasecki T, Sluts WS. The effects of three alternative nicotine delivery products on own-cigarette abstinence in a randomized controlled switching trial. In: Society for Research on Nicotine and Tobacco (SRNT) 30th Annual Meeting, 20-23 March 2024, Edinburgh UK. 2024:POS5-125 Rapids.

[238] Piasecki TM, Slutske WS, Bolt DM, Jorenby DE, Piper ME. Effects of very low nicotine cigarettes, e-cigarettes, and nicotine patches on daily own-cigarette abstinence in a randomized controlled switching trial. Drug and Alcohol Dependence 2025;268:112576. [DOI: 10.1016/j.drugalcdep.2025.112576] PMC11837730 39914192

[239] NCT04084210. Impact of alternative nicotine-delivery products on combustible cigarette use [Understanding the real-world impact of the use of three alternate nicotine-delivery products on combustible cigarette use]. clinicaltrials.gov/ct2/show/NCT04084210 (first received 10 September 2019).

[240] Piper M. What motivates e-cigarette or very low nicotine cigarette use during a switching study? In: Society for Research on Nicotine and Tobacco (SRNT), 20-23 March 2024, Edinburgh UK. 2024:POS3-108.

[241] Piper M, Schlam T, Donny E, Jorenby D. The real-world impact of three alternative nicotine delivery products on combustible cigarette use. In: Society for Research on Nicotine and Tobacco (SRNT) 29th Annual Meeting, 1-4 March. 2023:22 SYM17-2.

[242] Bonafont Reyes BV, Stevens E, Nicholson A, Lei L, Vojjala M, Sherman S. Understanding racial and ethnic differences in switching from combustible cigarettes to e-cigarettes in COPD patients. Journal of the American Geriatrics Society, Annual Scientific Meeting 2022;170(S1):S1-S345.

[243] Hatsukami D, Meier E, Lindgren BR, Anderson A, Reisinger S, Norton K, et al. A randomized clinical trial examining the effects of instructions for electronic cigarette use on smoking-related behaviors, and biomarkers of exposure. Nicotine & Tobacco Research 2020;22(9):1524-32. [DOI: 10.1093/ntr/ntz233] PMC7443587 31828315

[244] NCT03111537. Methods project 4: clinical trial - amended (COMET). https://clinicaltrials.gov/ct2/show/NCT03111537 (first received 21 May 2015).

[245] Eisenhofer J, Makanjuola T, Martinez V, Thompson-Lake DG, Rodgman C, DeBrule DS, et al. Efficacy of electronic cigarettes for smoking cessation in veterans. Drug and Alcohol Dependence 2015;156:e63-4.

[246] Butler AR, Lindson N, Fanshawe TR, Theodoulou A, Begh R, Hajek P, et al. Longer-term use of electronic cigarettes when provided as a stop smoking aid: systematic review with meta-analyses. Preventive Medicine 2022;165 (Pt B):107182. [DOI: 10.1016/j.ypmed.2022.107182] [PMID: 35933001]

[247] Lucchiari C, Masiero M, Mazzocco K, Veronesi G, Maisonneuve P, Jemos C, et al. Nicotine-free e-cigarettes might promote tobacco smoking reduction better than nicotine delivery devices: results of a double-blind randomized controlled trial at 1 year. Current Oncology (Toronto, Ont.) 2022;29(11):8579-90. [DOI: 10.3390/curroncol29110676] PMC9717729 36421329

[248] Masiero M, Lucchiari C, Mazzocco K, Veronesi G, Maisonneuve P, Jemos C, et al. E-cigarettes may support smokers with high smoking-related risk awareness to stop smoking in the short run: preliminary results by randomized controlled trial. Nicotine & Tobacco Research 2019;21(1):119-26. 10.1093/ntr/nty047 29660034

[249] Masiero M, Lucchiari C, Mazzocco K, Veronesi G, Maisonneuve P, Jemos C, et al. Corrigendum: E-cigarettes may support smokers with high smoking-related risk awareness to stop smoking in the short run: preliminary results by randomized controlled trial. Nicotine & Tobacco Research 2020;22(4):594-5. 10.1093/ntr/nty175 30239904

[250] Pravettoni G, Masiero M, Lucchiari C, Maisenneuve P, Mazzocco K, Veronesi G. The role of electronic cigarettes in smoking cessation among heavy smokers undergoing a lung cancer screening program: preliminary results of a randomized controlled study. Psycho-Oncology 2016;25:72. [DOI: 10.1002/pon.4082]

[251] NCT02422914. Benefits of tobacco free cigarette among heavy smokers undergoing a lung cancer screening program: a randomized controlled study. clinicaltrials.gov/show/NCT02422914 (first received 14 January 2015).

[252] Lucchiari C, Masiero M, Mazzocco K, Veronesi G, Maisonneauve P, Jemos C, et al. Benefits of e-cigarettes in smoking reduction and in pulmonary health among chronic smokers undergoing a lung cancer screening program at 6 months. Addictive Behaviours 2020;103:106222. 10.1016/j.addbeh.2019.106222 31838445

[253] Lucchiari C, Masiero M, Veronesi G, Maisonneuve P, Spina S, Jemos C, et al. Benefits of e-cigarettes among heavy smokers undergoing a lung cancer screening program: randomized controlled trial protocol. JMIR Research Protocols 2016;5(1):e21. [PMID: 26842790] 10.2196/resprot.4805 PMC4757781

[254] Pulvers K, Nollen NL, Rice M, Schmid CH, Qu K, Benowitz NL, et al. Effect of pod e-cigarettes vs cigarettes on carcinogen exposure among African American and Latinx smokers: a randomized clinical trial. JAMA Network Open 2020;3(11):e2026324. 10.1001/jamanetworkopen.2020.26324 PMC7675102 33206193

[255] Leavens EL, Nollen N, Ahluwalia J, Mayo M, Rice M, Brett E, et al. Changes in dependence, withdrawal, and craving among adult smokers who switch to nicotine salt pod-based e-cigarettes. In: Society for Research on Nicotine and Tobacco (SRNT) 2021 Annual Meeting, February 24-27 2021, virtual. Publisher not available as virtual session, 2021:96 (POD53-1), 116 (C-6).

[256] Rubenstein D, Sokolovsky AW, Aston ER, Nollen NL, Schmid CH, Rice M, et al. Predictors of smoking reduction among African American and Latinx smokers in a randomized controlled trial of JUUL e-cigarettes. Addictive Behaviors 2021;122:107037. [DOI: 10.1016/j.addbeh.2021.107037] PMC8330147 34284312

[257] Nollen NL, Leavens EL, Ahluwalia JS, Rice M, Mayo MS, Pulvers K. Menthol versus non-menthol flavouring and switching to e-cigarettes in black and Latinx adult menthol combustible cigarette smokers: secondary analyses from a randomised clinical trial. Tobacco Control 2022;EPub:no pagination. [DOI: 10.1136/tobaccocontrol-2021-057180] PMC10246471 35351805

[258] Arnold MJ, Nollen NL, Mayo MS, Ahluwalia JS, Leavens EL, Zhang G, et al. Harm reduction associated with dual use of cigarettes and e-cigarettes in Black and Latino smokers: secondary analyses from a randomized controlled e-cigarette switching trial. Nicotine & Tobacco Research 2021;23(11):1972-6. [DOI: 10.1093/ntr/ntab069] PMC8562357 33837422

[259] Leavens E, Nollen N, Ahluwalia J, Mayo M, Rice M, Brett E, et al. Changes in dependence, withdrawal, and craving among adult smokers who switch to nicotine salt pod-based e-cigarettes. Journal of Clinical and Translational Science 2021;5(Suppl 1):85-6. [DOI: 10.1017/cts.2021.622] PMC8655025 34105208

[260] Arnold M, Nollen N, Mayo M, Ahluwalia J, Leavens E, Zhang G, et al. Dual use of cigarettes and e-cigarettes in African American and LatinX smokers: secondary analyses from a randomised controlled e-cigarettes switching trial. In: Society for Research on Nicotine and Tobacco (SRNT) 2021 Annual Meeting, February 24-27 2021, virtual. Publisher not available as virtual session, 2021:31 (POD4-2).

[261] Rice M, Nollen N, Woodcock A, Benowitz N, Ahluwalia J, Pulvers K. Effect of marijuana use on smokers switching to e-cigarettes in a randomised control trial. In: Society for Research on Nicotine and Tobacco (SRNT) 2021 Annual Meeting, February 24-27 2021, virtual. 2021:125 (SRNT C-44). 10.1093/ntr/ntac008 PMC9199934 35022796

[262] Rice M, Nollen NL, Ahluwalia JS, Benowitz N, Woodcock A, Pulvers K. Effects of marijuana use on smokers switching to e-cigarettes in a randomized clinical trial. Nicotine & Tobacco Research 2022;24(7):994-1002. [DOI: 10.1093/ntr/ntac008] PMC9199934 35022796

[263] Rubenstein D, Sokolovsky A, Aston E, Nollen N, Schmid C, Pulvers K, et al. Predictors of smoking reduction among African American and LatinX smokers in a randomised controlled trial of Juul e-cigarettes. In: Society for Research on Nicotine and Tobacco (SRNT) 2021 Annual Meeting, February 24-27 2021, virtual. 2021:129 (C-56). 10.1016/j.addbeh.2021.107037 PMC8330147 34284312

[264] Leavens EL, Nollen N, Ahluwalia JS, Mayo MS, Rice M, Brett EI, et al. Changes in dependence, withdrawal, and craving among adult smokers who switch to nicotine salt pod-based e-cigarettes. Addiction (Abingdon, England) 2021;117(1):207-15. [DOI: 10.1111/add.15597] PMC8655025 34105208

[265] Nollen N, Lambert L, Leavens E, Ahluwalia J, Rice M, Pulvers K. Does menthol flavoring help black and LatinX adult menthol combustible cigarette smokers switch to e-cigarettes?: secondary analyses from a randomized clinical trial. In: Society for Research on Nicotine and Tobacco, Annual Meeting, March 2022, Baltimore USA. 2022.

[266] Lee SC, Maglalang DD, Avila JC, Leavens EL, Nollen NL, Pulvers K, et al. Change in e-cigarette risk perception and smoking behavior of Black and Latinx individuals who smoke. Drug and Alcohol Dependence 2023;245:109824. [DOI: 10.1016/j.drugalcdep.2023.109824] PMC10033448 36857841

[267] NCT03511001. Effects among smokers who use and do not use e-cigarettes. https://clinicaltrials.gov/ct2/show/NCT03511001 (first received 10 April 2018).

[268] Ely J. Evaluation of the use of electric cigarettes in a rural smoking cessation program. digscholarship.unco.edu/cgi/viewcontent.cgi?article=1001&context=capstones 2013 (accessed 1 November 2014).

[269] Price AD, Coffey M, Houston L, Cook PA. Evaluation of a pharmacy supported e-cigarette smoking cessation intervention in Northwest England. BMC Public Health 2022;22:1326. [DOI: 10.1186/s12889-022-13711-x] PMC9273914 35820869

[270] Felicione NJ, Enlow P, Elswick D, Long D, Sullivan CR, Blank MD. A pilot investigation of the effect of electronic cigarettes on smoking behavior among opioid-dependent smokers. Addictive Behaviors 2019;91:45-50. 10.1016/j.addbeh.2018.07.003 30006020

[271] Tseng TY, Ostroff JS, Campo A, Gerard M, Kirchner T, Rotrosen J, et al. A randomized trial comparing the effect of nicotine versus placebo electronic cigarettes on smoking reduction among young adult smokers. Nicotine & Tobacco Research 2016;18(10):1937-43. 10.1093/ntr/ntw017 PMC5016841 26783292

[272] Guttentag A. Do e-cigarettes "Work" for smokers as a step on the way to cessation? An analysis of the temporal and spatial correlates of switching in a harm reduction trial among adult smokers. Dissertation Abstracts International: Section B: The Sciences and Engineering 2021;82:8-B.

[273] Guttentag A, Tuo-Yen T, Shelley D, Thomas K. Analyzing trajectories of acute cigarette reduction post-introduction of an e-cigarette using ecological momentary assessment data. International Journal of Environmental Research and Public Health 2022;19(12):7452. [DOI: 10.3390/ijerph19127452] PMC9223631 35742698

[274] NCT02628964. Assessing the use of electronic cigarettes (e-cigarettes) as a harm reduction strategy. clinicaltrials.gov/show/NCT02628964 (first received 28 September 2015).

[275] Kumral TL, Saltürk Z, Yildirim G, Uyar Y, Berkiten G, Atar Y, et al. How does electronic cigarette smoking affect sinonasal symptoms and nasal mucociliary clearance? Royal Belgian Society for Ear, Nose, Throat, Head and Neck Surgery (B-ENT) 2016;12(1):17-21. 27097389

[276] Ozga-Hess JE, Felicione NJ, Ferguson SG, Dino G, Elswick D, Whitworth C, et al. Piloting a clinical laboratory method to evaluate the influence of potential modified risk tobacco products on smokers' quit-related motivation, choice, and behaviour. Addictive Behaviors 2019;99:106105. 10.1016/j.addbeh.2019.106105 PMC6791779 31470240

[277] Pope I, Clark LV, Clark A, Ward E, Belderson P, Stirling S, et al. CessatiOn of Smoking Trial in the Emergency Department (COSTED): a multicentre randomised controlled trial. Emergency Medicine Journal 2024;41(5):276-82. [DOI: 10.1136/emermed-2023-213824] PMC11041600 38531658

[278] Ward E, Belderson P, Clark A, Stirling S, Clark L, Pope I, et al. How do people quit smoking using e-cigarettes? A mixed-methods exploration of participant smoking pathways following receiving an opportunistic e-cigarette-based smoking cessation intervention. Addiction (Abingdon, England) 2024;119:2185-96. [DOI: 10.1111/add.16633] 39252616

[279] Belderson P, Ward E, Clark L, Stirling S, Clark A, Notley C, et al. Cessation of smoking trial in the emergency department (COSTED): a multi-centre, randomised controlled trial. In: SRNT 30th Annual Meeting, 2024 March 20-23, Edinburgh, UK. 2024:POS2-167.

[280] Li J, Wu Q, Parrott S, Pope I, Clark LV, Clark A, et al. Cost-utility analysis of provision of e-cigarette starter kits for smoking cessation in emergency departments: an economic evaluation of a randomized controlled trial. Addiction (Abingdon, England) 2025;120(2):368-79. [DOI: 10.1111/add.16698] PMC11707313 39482840

[281] Belderson P, Ward E, Pope I, Notley C. Selecting an e-cigarette for use in smoking cessation interventions and healthcare services: findings from patient and public consultation for the COSTED trial. BMJ Open 2024;13(3):1-9. [DOI: 10.1136/bmjopen-2023-078677] PMC11146363 38443079

[282] Pope I, Notley C. Cessation of smoking trial in the emergency department. In: SRNT 29th Annual Meeting; 2023 Mar 1-4; San Antonio (TX), USA. 2023:119-20 POS2-14.

[283] Pope I, Notley C, Boyle A. Results of the cessation of smoking trial in the emergency department (COSTED). Emergency Medicine Journal 2023;40(12):873-4.

[284] Notley C, Belderson P, Ward E, Clark LV, Clark A, Stirling S, et al. The context of the emergency department as a location for a smoking cessation intervention - process evaluation findings from the cessation of smoking trial in the emergency department trial. Nicotine & Tobacco Research 2025;27(5):909-16. [DOI: 10.1093/ntr/ntae223] PMC12012237 39505370

[285] Notley C, Clark L, Belderson P, Ward E, Clark AB, Parrott S, et al. Cessation of smoking trial in the emergency department (CoSTED): protocol for a multicentre randomised controlled trial. BMJ Open 2023;13(1):e064585. 10.1136/bmjopen-2022-064585 PMC9853266 36657751

[286] Pope I, Rashid S, Iqbal H, Belderson P, Ward E, Clark L, et al. Engagement with stop smoking services after referral or signposting: a mixed methods study. Nicotine & Tobacco Research 2025;27(2):360-3. [DOI: 10.1093/ntr/ntae159] PMC11750743 38955669

[287] NCT04854616. Cessation of smoking trial in the emergency department (COSTED). clinicaltrials.gov/ct2/show/NCT04854616 (date first received 22 April 2021).

[288] Begh R, Bateman P, Williams N, Grabey J, Stevens R. Examining the effectiveness of general practitioner and nurse promotion of electronic cigarettes versus standard care for smoking reduction and abstinence in hardcore smokers with smoking-related chronic disease: a randomised controlled trial. Statistical Analysis Report, Version 2.0 2021 1-56. 10.1186/s13063-019-3850-1 PMC6883522 31779689

[289] Begh R. Training general practitioners and nurses to deliver brief advice about e-cigarettes for smoking reduction: lessons learned from a randomised controlled trial. In: SRNT E-Conference, SY3-04. 2021.

[290] ISRCTN59404712. GP/nurse promotion of e-cigarettes in supporting reduced smoking and cessation in smokers. www.isrctn.com/ISRCTN59404712 (first received 28 November 2017).

[291] Albury C, Barnes R, Ferrey A, Coleman T, Gilbert H, Naughton F, et al. The old and familiar meets the new and unknown: patient and clinician perceptions on e-cigarettes for smoking reduction in UK general practice, a qualitative interview study. Addiction 2022;117(5):1427-37. [DOI: 10.1111/add.15760] PMC9306504 34859526

[292] Begh R, Coleman T, Yardley L, Barnes R, Naughton F, Gilbert H, et al. Examining the effectiveness of general practitioner and nurse promotion of electronic cigarettes versus standard care for smoking reduction and abstinence in hardcore smokers with smoking-related chronic disease: protocol for a randomised controlled trial. Trials 2019;20(1):659. 10.1186/s13063-019-3850-1 PMC6883522 31779689

[293] Pratt SI, Brunette MF, Ferron JC, Santos M, Sargent J, Xie H. E-cigarette provision to promote switching in cigarette smokers with serious mental illness - a randomized trial. Nicotine & Tobacco Research 2022;6(24):1405-12. [DOI: 10.1093/ntr/ntac082] PMC9356685 35363874

[294] Pratt S, Ferron J, Sargent J, Brunette M. Assessing the effect of e-cigarettes versus usual smoking on NNAL among chronic smokers with serious mental illness. In: Society for Research on Nicotine and Tobacco (SRNT) 29th Annual Meeting, 1-4 March 2023. Publisher not available, 2023:73 (PPS22-5).

[295] Sargent JD, Pratt SI, Brunette MF, Ferron JC, Santos MM, Stoolmiller M. Level and timing of product substitution in a trial of e-cigarettes for smokers not interested in quitting. Tobacco Induced Diseases 2024;22:101201591. [DOI: 10.18332/tid/189220] PMC11170977 38873183

[296] Santos M. Novel evaluation of the relationship between trajectories of e-cigarette initiation and cigarette reduction while switching for harm reduction. In: Society for Research on Nicotine and Tobacco (SRNT), 20-23 March 2024, Edinburgh UK. Publisher not available, 2024.

[297] Ferron J. What predicts harm reduction in adults with severe mental illness provided e-cigarettes? In: Society for Research on Nicotine and Tobacco (SRNT), 20-23 March 2024, Edinburgh UK. 2024.

[298] NCT03050853. The appeal and impact of e-cigarettes in smokers with SMI. clinicaltrials.gov/ct2/show/NCT03050853 (first received 13 February 2017).

[299] Edwards S, Puljevic C, Dean JA, Gilks C, Boyd MA, Baker P, et al. Tobacco harm reduction with vaporised nicotine (THRiVe): a feasibility trial of nicotine vaping products for smoking cessation among people living with HIV. AIDS and Behavior 2023;27(2):618-27. [DOI: 10.1007/s10461-022-03797-0] PMC9908735 35869375

[300] ACTRN12616001641482. Tobacco harm reduction with vaporised nicotine (THRiVe): feasibility study. www.anzctr.org.au/Trial/Registration/TrialReview.aspx?ACTRN=12616001641482 (first received 28 November 2016).

[301] Bell S, Dean J, Gilks C, Boyd MA, Fitzgerald L, Mutch A, et al. Tobacco harm reduction with vaporised nicotine (THRiVe): the study protocol of an uncontrolled feasibility study of novel nicotine replacement products among people living with HIV who smoke. International Journal of Environmental Research and Public Health 2017;14(7):799. 10.3390/ijerph14070799 PMC5551237 28718828

[302] Goniewicz ML, Gawron M, Smith DM, Peng M, Jacob P 3rd, Benowitz NL. Exposure to nicotine and selected toxicants in cigarette smokers who switched to electronic cigarettes: a longitudinal within-subjects observational study. Nicotine & Tobacco Research 2017;19(2):160-7. 10.1093/ntr/ntw160 PMC5234360 27613896

[303] Pratt SI, Sargent J, Daniels L, Santos MM, Brunette M. Appeal of electronic cigarettes in smokers with serious mental illness. Addictive Behaviors 2016;59:30-4. 10.1016/j.addbeh.2016.03.009 27043170

[304] Hickling LM, Perez-Iglesias R, McNeill A, Dawkins L, Moxham J, Ruffell T, et al. A pre-post pilot study of electronic cigarettes to reduce smoking in people with severe mental illness. Psychological Medicine 2019;49(6):1033-40. 10.1017/S0033291718001782 29986786

[305] NCT02212041. Acceptability, patterns of use and safety of electronic cigarette in people with mental illness: a pilot study. clinicaltrials.gov/show/NCT02212041 (first received 6 August 2014).

[306] Humair J-P, Tango R. Can e-cigarette help patients to reduce or stop smoking in primary care practice? Journal of General Internal Medicine 2014;29:S480.

[307] Valentine GW, Hefner K, Jatlow PI, Rosenheck RA, Gueorguieva R, Sofuoglu M. Impact of e-cigarettes on smoking and related outcomes in veteran smokers with psychiatric comorbidity. Journal of Dual Diagnosis 2018;14(1):2-13. 10.1080/15504263.2017.1384877 PMC7131866 29083287

[308] NCT02648178. Evaluation of appeal and impact of e-cigarettes among chronic smokers with smoking-related cancers. clinicaltrials.gov/show/NCT02648178 (first received 5 January 2016).

[309] George J, Hussain M, Vadiveloo T, Ireland S, Hopkinson P, Struthers AD, et al. Cardiovascular effects of switching from tobacco cigarettes to electronic cigarettes. Journal of the American College of Cardiology 2019;74(25):3112-20. 10.1016/j.jacc.2019.09.067 PMC6928567 31740017

[310] NCT02878421. Vascular EffectS of regUlar cigarettes Versus electronIc cigarette USe (VESUVIUS). clinicaltrials.gov/ct2/show/NCT02878421 (first received 25 August 2016).

[311] Meier E, Wahlquist AE, Heckman BW, Cummings KM, Froeliger B, Carpenter MJ. A pilot randomized crossover trial of electronic cigarette sampling among smokers. Nicotine & Tobacco Research 2017;19(2):176-82. 10.1093/ntr/ntw157 PMC5234361 27613880

[312] Ikonomidis I, Vlastos D, Kourea K, Kostelli G, Varoudi M, Pavlidis G, et al. Electronic cigarette smoking increases arterial stiffness and oxidative stress to a lesser extent than a single conventional cigarette: an acute and chronic study. Circulation 2018;137(3):303-6. 10.1161/CIRCULATIONAHA.117.029153 29335291

[313] NCT03039920. The effects of electronic cigarette smoking on the arterial wall and endothelial glycocalyx properties of smokers. clinicaltrials.gov/ct2/show/NCT03039920 (first received 1 February 2017).

[314] Pulvers K, Emami AS, Nollen NL, Romero DR, Strong DR, Benowitz NL, et al. Tobacco consumption and toxicant exposure of cigarette smokers using electronic cigarettes. Nicotine & Tobacco Research 2018;20(2):206-14. 10.1093/ntr/ntw333 PMC6251645 28003511

[315] Oncken CA, Litt MD, McLaughlin LD, Burki NA. Nicotine concentrations with electronic cigarette use: effects of sex and flavor. Nicotine & Tobacco Research 2015;17(4):473-8. 10.1093/ntr/ntu232 25762758

[316] Swedeh MA, Oncken C, Burki NK. Acute effects of electronic cigarettes on airway function in human subjects. In: American Thoracic Society International Conference Abstracts. Vol. 189. 2014:A4089.

[317] NCT01775787. Effects of electronic cigarettes on nicotine concentrations (ECIG). clinicaltrials.gov/ct2/show/NCT01775787 (first received 25 January 2013).

[318] Lindson N, Butler AR, Liber A, Levy D, Barnett P, Theodoulou A, et al. An exploration of flavours in studies of e-cigarettes for smoking cessation: secondary analyses of a systematic review with meta-analyses. Addiction 2023;118(4):634–45. [DOI: 10.1111/add.16091] PMC10952306 36399154

[319] Lindson N, Livingstone-Banks J, Butler AR, Levy DT, Barnett P, Theodoulou A, et al. An update of a systematic review and meta-analyses exploring flavours in intervention studies of e-cigarettes for smoking cessation. Addiction (Abingdon, England) 2025;120(4):770-8. [DOI: 10.1111/add.16736] PMC11907327 39702981

[320] Walker N, Parag V, Verbiest M, Laking G, Laugesen M, Bullen C. Nicotine patches used in combination with e-cigarettes (with and without nicotine) for smoking cessation: a pragmatic, randomised trial. Lancet Respiratory Medicine 2020;8(1):54-64. [DOI: 10.1016/S2213-2600(19)30269-3] 31515173

[321] NCT02521662. A randomised-controlled clinical trial to evaluate the effectiveness and safety of combining nicotine patches with e-cigarettes (with and without nicotine) plus behavioural support, on smoking abstinence. clinicaltrials.gov/show/NCT02521662 (first received 9 August 2015).

[322] Walker N, Verbiest M, Kurdziel T, Laking G, Laugesen M, Parag V, et al. Effectiveness and safety of nicotine patches combined with e-cigarettes (with and without nicotine) for smoking cessation: study protocol for a randomised controlled trial. BMJ Open 2019;9(2):e023659. 10.1136/bmjopen-2018-023659 PMC6398670 30808668

[323] Morphett K, Fraser D, Borland R, Hall W, Walker N, Bullen C, et al. A pragmatic randomized comparative trial of e-cigarettes and other nicotine products for quitting or long-term substitution in smokers. Nicotine & Tobacco Research 2022;24(7):1079-88. [DOI: 10.1093/ntr/ntab266] 34929031

[324] Fraser D, Borland R, Gartner C. Protocol for a randomised pragmatic policy trial of nicotine products for quitting or long-term substitution in smokers. BMC Public Health 2015;15:1026. 10.1186/s12889-015-2366-1 PMC4596390 26444980

[325] ACTRN12612001210864. Can using nicotine as a long-term substitute enhance smoking cessation over using it only as a cessation aid? [An open-label randomised pragmatic policy trial examining effectiveness of short-term use of Nicotine Replacement Therapy (NRT) vs short- or long-term use of NRT vs short- or long-term use of NRT or electronic nicotine delivery systems for smoking cessation in cigarette smokers]. https://www.anzctr.org.au/Trial/Registration/TrialReview.aspx?ACTRN=12612001210864 (first received 15 November 2012).

[326] Morphett K, Boyd M, Puljevic C, Gilks C, Bonevski B, Borland R, et al. Do nicotine vaping products increase quitting among priority population groups over standard smoking cessation therapy? A pragmatic randomised partial cross-over trial. In: Society for Research on Nicotine and Tobacco (SRNT), Annual Meeting 2022. SYM17-4. 2022.

[327] ACTRN12618000408280. Cessation And Relapse Prevention (CARP) trial: nicotine vaporisers compared to standard nicotine replacement therapy for smoking cessation among people with co-morbidities. www.anzctr.org.au/ACTRN12618000408280.aspx (first received 21 March 2018).

[328] Baldassarri SR, Bernstein SL, Chupp GL, Slade MD, Fucito LM, Toll BA. Electronic cigarettes for adults with tobacco dependence enrolled in a tobacco treatment program: a pilot study. Addictive Behaviors 2018;80:1-5. [DOI: 10.1016/j.addbeh.2017.11.033] PMC6463885 29304395

[329] Baldassarri SR, Bernstein SL, Chupp GL, Toll BA. Electronic cigarettes for adults with tobacco dependence undergoing a tobacco treatment program: a pilot prospective study. American Journal of Respiratory and Critical Care Medicine, Meeting Abstracts 2016;193:A6511.

[330] NCT02498145. Short term effects of electronic cigarettes in tobacco dependent adults. clinicaltrials.gov/show/NCT02498145 (first received 13 July 2015).

[331] Tattan-Birch H, Kock L, Brown J, Beard E, Bauld L, West R, et al. E-cigarettes to augment stop smoking in-person support and treatment with varenicline (E-ASSIST): a pragmatic randomised controlled trial. Nicotine & Tobacco Research 2023;25(3):395-403. [DOI: 10.1093/ntr/ntac149] PMC9384384 35738868

[332] ISRCTN16931827. A trial to assess the benefit of offering an e-cigarette starter kit to smokers attempting to stop smoking with varenicline. isrctn.com/ISRCTN16931827 (first received 23 August 2018).

[333] Hartmann-Boyce J, Butler AR, Theodoulou A, Onakpoya IJ, Hajek P, Bullen C, et al. Biomarkers of potential harm in people switching from smoking tobacco to exclusive e-cigarette use, dual use, or abstinence: secondary analysis of Cochrane systematic review of trials of e-cigarettes for smoking cessation. Addiction (Abingdon, England) 2023;118(3):539-45. [DOI: 10.1111/add.16063] PMC10092879 36208090

[334] Theodoulou A, Chepkin SC, Ye W, Fanshawe TR, Bullen C, Hartmann-Boyce J, et al. Different doses, durations and modes of delivery of nicotine replacement therapy for smoking cessation. Cochrane Database of Systematic Reviews 2023, Issue 6. Art. No: CD013308. [DOI: 10.1002/14651858.CD013308.pub2] PMC10278922 37335995

[335] Halpern SD, Harhay MO, Saulsgiver K, Brophy C, Troxel AB, Volpp KG. A pragmatic trial of e-cigarettes, incentives, and drugs for smoking cessation. New England Journal of Medicine 2018;378(24):2302-10. 10.1056/NEJMsa1715757 29791259

[336] NCT02328794. Randomized clinical trial to reduce harm from tobacco. clinicaltrials.gov/show/NCT02357173 (first received 3 October 2014).

[337] Harhay MO, Troxel AB, Brophy C, Saulsgiver K, Volpp KG, Halpern SD. Financial incentives promote smoking cessation directly, not by increasing use of cessation aids. Annals of the American Thoracic Society 2019;16(2):280-2. [DOI: 10.1513/AnnalsATS.201808-574RL] PMC6376943 30290121

[338] Etter JF, Bullen C. Electronic cigarette: users profile, utilization, satisfaction and perceived efficacy. Addiction (Abingdon, England) 2011;106(11):2017-28. 10.1111/j.1360-0443.2011.03505.x 21592253

[339] Wu A, Conde M, Butler A, Knight E, Lindson N, Livingstone-Banks J, et al. Electronic cigarettes for smoking cessation: an overview of systematic reviews and evidence and gap map. Unpublished manuscript under review. 10.1111/add.70388 PMC13357930 41887411

[340] Hanewinkel R, Niederberger K, Pedersen A, Unger JB, Galimov A. E-cigarettes and nicotine abstinence: a meta-analysis of randomised controlled trials. European Respiratory Review 2022;31(163):210215-5. [DOI: 10.1183/16000617.0215-2021] PMC9488503 35321930

[341] Li J, Hui X, Fu J, Ahmed MM, Yao L, Yang K. Electronic cigarettes versus nicotine-replacement therapy for smoking cessation: a systematic review and meta-analysis of randomized controlled trials. Tobacco Induced Diseases 2022;20:1-13. [DOI: 10.18332/tid/154075] PMC9582581 36339933

[342] Levett JY, Filion KB, Reynier P, Prell C, Eisenberg MJ. Efficacy and safety of e-cigarette use for smoking cessation: a systematic review and meta-analysis of randomized controlled trials. American Journal of Medicine 2023;136(8):804-13. 10.1016/j.amjmed.2023.04.014 37148992

[343] Thomas KH, Dalili MN, López‐López JA, Keeney E, Phillippo DM, Munafò MR, et al. Comparative clinical effectiveness and safety of tobacco cessation pharmacotherapies and electronic cigarettes: a systematic review and network meta-analysis of randomized controlled trials. Addiction (Abingdon, England) 2022;117(4):861-76. 10.1111/add.15675 PMC9293179 34636108

[344] Chan GC, Stepanovic D, Lim C, Sun T, Shanmuga Anandan AS, Connor JP, et al. A systematic review of randomised controlled trials and network meta-analysis of e-cigarettes for smoking cessation. Addictive Behaviours 2021;119:106912. [DOI: 10.1016/j.addbeh.2021.106912] 33798919

[345] Quigley JM, Walsh C, Lee C, Long J, Kennelly H, McCarthy A, et al. Efficacy and safety of electronic cigarettes as a smoking cessation intervention: a systematic review and network meta-analysis. Tobacco Prevention & Cessation 2021;7(69):1-14. [DOI: 10.18332/tpc/143077] PMC8607936 34877438

[346] Pound CM, Zhang JZ, Kodua AT, Sampson M. Smoking cessation in individuals who use vaping as compared with traditional nicotine replacement therapies: a systematic review and meta-analysis. BMJ Open 2021;11(2):e044222. [DOI: 10.1136/bmjopen-2020-044222] PMC7903126 33619197

[347] Patnode CD, Henderson JT, Coppola EL, Melnikow J, Durbin S, Thomas RG. Interventions for tobacco cessation in adults, including pregnant persons: updated evidence report and systematic review for the US Preventive Services Task Force. JAMA 2021;325(3):280-98. 10.1001/jama.2020.23541 PMC13475361 33464342

[348] Khoudigian S, Devji T, Lytvyn L, Campbell K, Hopkins R, O’Reilly D. The efficacy and short-term effects of electronic cigarettes as a method for smoking cessation: a systematic review and a meta-analysis. International Journal of Public Health 2016;61(2):257-67. [DOI: 10.1007/s00038-016-0786-z] 26825455

[349] Huang S, Tang O, Zheng X, Li H, Wu Y, Yang L. Effectiveness of smoking cessation on the high-risk population of lung cancer with early screening: a systematic review and meta-analysis of randomized controlled trials until January 2022. Archives of Public Health 2023;81(1):101-1. [DOI: 10.1186/s13690-023-01111-5] PMC10239152 37268972

[350] Vanderkam P, Bonneau A, Kinouani S, Dzeraviashka P, Castera P, Besnier M, et al. Duration of the effectiveness of nicotine electronic cigarettes on smoking cessation and reduction: systematic review and meta-analysis. Frontiers in Psychiatry (Frontiers Research Foundation) 2022;4(13):915946. 10.3389/fpsyt.2022.915946 PMC9386078 35990084

[351] Ibrahim S, Habiballah M, Sayed IE. Efficacy of electronic cigarettes for smoking cessation: a systematic review and meta-analysis. American Journal of Health Promotion 2020;35(3):442-55. [DOI: 10.1177/0890117120980289] 33327728

[352] Liber AC, Knoll M, Cadham CJ, Issabakhsh M, Oh H, Cook S. The role of flavored electronic nicotine delivery systems in smoking cessation: a systematic review. Drug and Alcohol Dependence Reports 2023;7:100143. [DOI: 10.1016/j.dadr.2023.100143] PMC10066538 37012981

[353] Livingstone-Banks J, Travis N, Conde M, Chen Y(C), Zi P, Jarman H, et al. The impacts of e-cigarette flavours: an overview of systematic reviews. Addiction 2025 [Epub ahead of print]. [DOI: 10.1111/add.70017] PMC12128567 39999998

[354] Hajek P, Corbin L, Ladmore D, Spearing E. Adding e-cigarettes to specialist stop-smoking treatment: City of London pilot project. Journal of Addiction Research & Therapy 2015;6(244):(online ahead of print). [DOI: 10.4172/2155-6105.1000244]

[355] Ikonomidis I, Katogiannis K, Kostelli G, Kourea K, Kyriakou E, Kypraiou A, et al. Effects of electronic cigarette on platelet and vascular function after four months of use. Food and Chemical Toxicology 2020;141:111389. 10.1016/j.fct.2020.111389 32343994

[356] Scheibein F, McGirr K, Morrison A, Roche W, Wells JS. An exploratory non-randomized study of a 3-month electronic nicotine delivery system (ENDS) intervention with people accessing a homeless supported temporary accommodation service (STA) in Ireland. Harm Reduction Journal 2020;17(1):73. 10.1186/s12954-020-00406-y PMC7549237 33046080

[357] Scheibein F, McGirr K, Morrison A, Roche W, Wells JS. Correction to: an exploratory non-randomized study of a 3-month electronic nicotine delivery system (ENDS) intervention with people accessing a homeless supported temporary accommodation service (STA) in Ireland. Harm Reduction Journal 2021;18(1):113. [DOI: 10.1186/s12954-021-00549-6] PMC8579632 34753488

[358] Adriaens K, Van Gucht D, Declerck P, Baeyens F. Effectiveness of the electronic cigarette: an eight-week Flemish study with six-month follow-up on smoking reduction, craving and experienced benefits and complaints. International Journal of Environmental Research and Public Health 2014;11(11):11220-48. 10.3390/ijerph111111220 PMC4245610 25358095

[359] Pacifici R, Pichini S, Graziano S, Pellegrini M, Massaro G, Beatrice F. Successful nicotine intake in medical assisted use of e-cigarettes: a pilot study. International Journal of Environmental Research and Public Health 2015;12(7):7638-46. 10.3390/ijerph120707638 PMC4515680 26184244

[360] Auer R, Schoeni A, Humair J-P, Jacot-Sadowski I, Berlin I, Stuber MJ, et al. Electronic nicotine-delivery systems for smoking cessation. New England Journal of Medicine 2024;390(7):601-10. [DOI: 10.1056/NEJMoa2308815] 38354139

[361] Scharf T, Rihs 1, Schoeni A, Maire M, Tal K, Jakob J, et al. Effect of electronic nicotine delivery systems for smoking cessation on sleep quality: secondary analysis of a randomised controlled trial. In: Society for Research on Nicotine and Tobacco (SRNT) 20-23 March 2024 Edinburgh UK. POS4-66. 2024. 10.1093/sleep/zsag028 PMC13357506 41641973

[362] Nyilas S, Bauman G, Korten I, Pusterla O, Singer F, Ith M. MRI shows lung perfusion changes after vaping and smoking. Radiology 2022;304(1):195-204. [DOI: 10.1148/radiol.211327] 35380498

[363] Jakob J, Schoeni A, Tal K, Jacot-Sadowski I, Clair C, Humair JP, et al. Effects of electronic nicotine delivery systems (ENDS) for smoking cessation on changes in weight- secondary analysis of the ESTXENDS trial. In: SRNT 29th Annual Meeting; 2023 Mar 1-4; San Antonio (TX), USA. 2023:POS3-8.

[364] Baggio S, Schoeni A, Abolhassani N, Tal K, Pohle S, Vetsch J, et al. Efficacy of electronic nicotine delivery systems (ENDS) for smoking cessation in populations with psychiatric and substance use problems: secondary analysis of a randomized controlled trial. In: SRNT 30th Annual Meeting, March 2024, Edinburgh UK. 2024:PPS24-1.

[365] Auer R, Schoeni A, Humair JP, Jacot-Sadowski I, Berlin I, Stuber M, et al. Efficacy, safety and toxicology of electronic nicotine delivery systema (ESTXENDS) as an aid for smoking cessation, a randomised controlled trial. In: SRNT 29th Annual Meeting; 2023 Mar 1-4; San Antonio (TX), USA. 2023:POS3-5.

[366] Mosimann AF, Guttinger EM, Tal K, Schoeni A, Baggio S, Sambiagio N, et al. E-liquid flavors and nicotine concentration choices over 6 months after a smoking cessation attempt with ENDS: secondary analyses of a randomized controlled trial. Tobacco Prevention & Cessation 2025;11:101693412. [DOI: 10.18332/tpc/196136] PMC11734314 39816168

[367] Rihs A, Schoeni A, Scharf T, Jakob J, Tal K, Jacot-Sadowski I, et al. Effect of e-cigarettes for smoking cessation on depressive and anxiety symptoms: secondary analysis of a randomized controlled trial. General Hospital Psychiatry 2025;93:67-72. [DOI: 10.1016/j.genhosppsych.2025.01.011] 39827790

[368] Schoeni A, Baggio S, Jakob J, Humair J-P, Jacot-Sadowski I, Berlin I, et al. Long-term efficacy outcomes of electronic nicotine delivery systems (ENDS) for smoking cessation: 12- and 24-months follow-up of the efficacy, safety and toxicology of ENDS (ESTxENDS) randomized controlled trial. In: Society for Research on Nicotine and Tobacco (SRNT) 20-23 March 2024 Edinburgh UK. PPS25-1. 2024.

[369] NCT04236791. The ESTxENDS trial - electronic nicotine delivery systems as an aid for smoking cessation - extension of follow-up. clinicaltrials.gov/show/NCT04236791 (first received 22 January 2020).

[370] NCT03612336. The ESTxENDS trial - metabolic effects of using Electronic Nicotine Delivery Systems (ENDS/Vaporizer/E-cig) (ESTxENDS). clinicaltrials.gov/show/NCT03612336 (first received 2 August 2018).

[371] NCT03589989. The ESTxENDS trial - electronic nicotine delivery systems (ENDS/vaporizer/e-cigarette) as an aid for smoking cessation (ESTxENDS). clinicaltrials.gov/ct2/show/record/NCT03589989 (first received 18 July 2018).

[372] NCT03612544. The ESTxENDS trial - toxins from using Electronic Nicotine Delivery Systems (ENDS/Vaporizer/E-cig) (ESTxENDS). clinicaltrials.gov/show/NCT03612544 (first received 2 August 2018).

[373] NCT03938298. The ESTxENDS trial: pulmonary function substudy (PulmENDS). clinicaltrials.gov/ct2/show/nct03938298 (first received 6 May 2019).

[374] NCT03603340. The ESTxENDS trial - effects of using Electronic Nicotine Delivery Systems (ENDS/Vaporizer/E-cig) on depression (ESTxENDS). clinicaltrials.gov/show/NCT03603340 (first received 27 July 2018).

[375] NCT03603353. The ESTxENDS trial - effects of using Electronic Nicotine Delivery Systems (ENDS/Vaporizer/E-cig) on sleep quality (ESTxENDS). clinicaltrials.gov/show/NCT03603353 (first received 27 July 2018).

[376] NCT03612375. ESTxENDS trial - oxidative stress induced by Electronic Nicotine Delivery Systems (ENDS/Vaporizer/E-cig) measured in urine (ESTxENDS). clinicaltrials.gov/show/NCT03612375 (first received 2 August 2018).

[377] NCT03612453. ESTxENDS Trial - oxidative stress induced by Electronic Nicotine Delivery Systems (ENDS/Vaporizer/E-cig) measured in EBC (ESTxENDS). clinicaltrials.gov/show/NCT03612453 (first received 2 August 2018).

[378] NCT03632421. The ESTxENDS trial - effects of using Electronic Nicotine Delivery Systems (ENDS/Vaporizer/E-cig) on respiratory symptoms (ESTxENDS). clinicaltrials.gov/show/NCT03632421 (first received 15 August 2018).

[379] NCT04244773. ESTxENDS trial: MN substudy - micronuclei in buccal epithelium, a surrogate measure of future cancer risk, induced by Electronic Nicotine Delivery Systems (ENDS/Vaporizer/E-cig). clinicaltrials.gov/show/NCT04244773 (first received 28 January 2020).

[380] NCT04617444. The ESTxENDS trial - effects of using Electronic Nicotine Delivery Systems (ENDS/Vaporizer/E-cig) on olfactory function. clinicaltrials.gov/show/NCT04617444 (first received 05 November 2020).

[381] Mosimann AF, Güttinger EM, Tal K, Baggio S, Schoeni A, Jacot-Sadowski I, et al. Change in e-liquids flavor use and nicotine concentration over 6 months in participants of a smoking cessation trial with electronic nicotine delivery systems (ENDS): the ESTxENDS Trial. In: SRNT 29th Annual Meeting; 2023 Mar 1-4; San Antonio (TX), USA. 2023:46 (PPS10-5).

[382] Güttinger E, Mosimann A, Tal K, Schoeni A, Jacot-Sadowski I, Humair JP, et al. Prevalence of vaping-associated symptoms over six months among participants of a randomized controlled trial on smoking cessation with electronic nicotine delivery systems (ENDS). In: SRNT 29th Annual Meeting; 2023 Mar 1-4; San Antonio (TX), USA. 2023:PPS14-6.

[383] Baggio S, Bruggmann P, Schoeni A, Abolhassani N, Tal K, Pohle S, et al. Efficacy of e-cigarettes for smoking cessation in populations with psychiatric and/or substance use problems: a secondary analysis of a randomized controlled trial. Tobacco Prevention & Cessation 2025;11:1-10. [DOI: 10.18332/tpc/199473] PMC11788852 39902147

[384] Rihs A, Schoeni A, Scharf T, Jakob J, Rodondi N, Jacot-Sadowski I, et al. Effect of e-cigarettes for smoking cessation on depressive and anxiety symptoms: secondary analyses of a randomised controlled trial. In: Society for Research on Nicotine and Tobacco (SRNT) 29th Annual Meeting 1-4 March 2023. PPS10-6. 2023:46.

[385] Schoeni A, Baggio S, Jakob J, Humair J-P, Jacot-Sadowski I, Berlin I, et al. Long term efficacy outcomes of electronic nicotine delivery systems (ENDS) for smoking cessation: 12- and 24- months follow-up of the efficacy, safety and toxicology of ENDS (ESTxENDS) randomized controlled trial. Swiss Medical Weekly 2024;154:15S-16S. [DOI: 10.57187/s.3896]

[386] Carpenter MJ, Heckman BW, Wahlquist AE, Wagener TL, Goniewicz ML, Gray KM, et al. A naturalistic, randomized pilot trial of e-cigarettes: uptake, exposure, and behavioral effects. Cancer Epidemiology, Biomarkers & Prevention 2017;26(12):1795-803. 10.1158/1055-9965.EPI-17-0460 PMC5713898 29127080

[387] NCT02357173. A trial of e-cigarettes: natural uptake, patterns and impact of use. clinicaltrials.gov/show/NCT02357173 (first received 3 October 2014).

[388] Smith TT, Wahlquist AE, Heckman BW, Cummings KM, Carpenter MJ. Impact of e-cigarette sampling on cigarette dependence and reinforcement value. Nicotine & Tobacco Research 2020;22(2):297-301. 10.1093/ntr/nty258 PMC7297108 30500925

[389] Carpenter MJ, Wahlquist A, Cummings KM, Smith T. Complementary approaches to examining relationships between e-cigarettes and smoking: novel data from the US. In: SRNT 29th Annual Meeting; 2023 Mar 1-4; San Antonio (TX), USA. SYM5-1. 2023:5-6.

[390] Hunter JN, Fahey MC, Wahlquist A, Smith T, Carpenter M. E-cigarette expectations and use among non-treatment seeking veteran smokers in a small nationawide veteran population. In: SRNT 30th Annual Meeting March 2024, Edinburgh UK. POS3-122. 2024.

[391] O'Neal RA, Carpenter MJ, Wahlquist AE, Leavens EL, Smith TT, Fahey MC. The prospective relationship between a-priori intentions for and patterns of e-cigarette use among adults who smoke cigarettes. Addictive Behaviors 2024;156:108067. [DOI: 10.1016/j.addbeh.2024.108067] PMC11589789 38823347

[392] Smith TT, Wahlquist AE, Wagener TL, Cummings KM, Carpenter MJ. The impact of non-tobacco e-cigarette flavoring on e-cigarette uptake, cigarette smoking reduction, and cessation: a secondary analysis of a nationwide clinical trial. Addictive Behaviors 2025;163:108240. [DOI: 10.1016/j.addbeh.2024.108240] PMC13202483 39787830

[393] Carpenter MJ, Wahlquist AE, Dahne J, Gray KM, Cummings KM, Warren G, et al. Effect of unguided e-cigarette provision on uptake, use, and smoking cessation among adults who smoke in the USA: a naturalistic, randomised, controlled clinical trial. EClinicalMedicine 2023;63:102142. [DOI: 10.1016/j.eclinm.2023.102142] PMC10518503 37753443

[394] NCT03453385. Clinical outcomes of a nationwide, naturalistic E-cig trial (CONNECT). clinicaltrials.gov/ct2/show/NCT03453385 (first received 5 March 2018).

[395] O'Neal R, Fahey MC, Wahlquist A, Smith T, Carpenter M. Demographic and smoking history correlates of anticipated reasons for ENDS use. In: SRNT 29th Annual Meeting; 2023 Mar 1-4; San Antonio (TX), USA. POS2-18. 2023:121.

[396] Coffey M, Cooper-Ryan AM, Houston L, Thompson K, Cook PA. Using e-cigarettes for smoking cessation: evaluation of a pilot project in the North West of England. Perspectives in Public Health 2020;140(6):351-61. [DOI: 10.1177/1757913920912436] PMC7683886 32389072

[397] Bonevski B, Manning V, Wynne O, Gartner C, Borland R, Baker A, et al. QuitNic: a pilot randomised controlled trial comparing nicotine vaping products with nicotine replacement therapy for smoking cessation following residential detoxification. Nicotine & Tobacco Research 2021;23(3):462-70. 10.1093/ntr/ntaa143 PMC7885782 32770246

[398] ACTRN12617000849392. The QuitNic Study: a pilot study of electronic nicotine devices for smoking cessation with drug and alcohol clients. www.anzctr.org.au/Trial/Registration/TrialReview.aspx?id=372198 (first received 8 June 2017).

[399] Guillaumier A, Manning V, Wynne O, Gartner C, Borland R, Baker AL, et al. Electronic nicotine devices to aid smoking cessation by alcohol- and drug-dependent clients: protocol for a pilot randomised controlled trial. Trials 2018;19:415. 10.1186/s13063-018-2786-1 PMC6090830 30071863

[400] Holliday R, Preshaw PM, Ryan V, Sniehotta FF, McDonald S, Bauld L, et al. A feasibility study with embedded pilot randomised controlled trial and process evaluation of electronic cigarettes for smoking cessation in patients with periodontitis. Pilot and Feasibility Studies 2019;5:74. 10.1186/s40814-019-0451-4 PMC6547559 31171977

[401] ISRCTN17731903. Feasibility study of e-cigarettes in periodontitis. www.isrctn.com/ISRCTN17731903 (first received 27 September 2016).

[402] Holliday R, McColl E, Ryan V, McDonald S, Sniehotta F, Bauld L, et al. E-cigarettes for smoking cessation in patients with periodontitis: pilot RCT. In: IADR International Association for Dental Research, Vancouver, Canada. 2019.

[403] Ikonomidis I, Katogiannis K, Kostelli G, Kourea K, Kyriakou E, Kypraiou A, et al. Effects of electronic cigarette on platelet and vascular function after one month of use. European Heart Journal; Conference: European Society of Cardiology Congress, ESC 2020 2020;41(Suppl 2):2359.

[404] Martner SG, Dallery J. Technology-based contingency management and e-cigarettes during the initial weeks of a smoking quit attempt. Journal of Applied Behavior Analysis 2019;52(4):928-43. 10.1002/jaba.641 PMC7153483 31578724

[405] McRobbie H, Phillips A, Goniewicz ML, Smith KM, Knight-West O, Przulj D, et al. Effects of switching to electronic cigarettes with and without concurrent smoking on exposure to nicotine, carbon monoxide, and acrolein. Cancer Prevention Research (Philadelphia, Pa.) 2015;8(9):873-8. 10.1158/1940-6207.CAPR-15-0058 26333731

[406] McRobbie H, Goniewicz M, Phillips A, Myers-Smith K, West O, Hajek P. Effects of the use of electronic cigarettes with and without concurrent smoking on acrolein delivery. In: Society for Research on Nicotine and Tobacco, 20th Annual Meeting, Seattle, Washington. POS4-33. 2014:13.

[407] NCT01714778. Toxins and delivery in E-cigarette users (TADEUS). clinicaltrials.gov/ct2/show/NCT01714778 (first received 26 October 2012).

[408] NCT02918630. E-cigarettes to promote smoking reduction among individuals with schizophrenia. clinicaltrials.gov/ct2/show/NCT02918630 (first received 5 July 2018).

[409] Okuyemi KS, Ojo-Fati O, Aremu TO, Friedrichsen CS, Grude L, Oyenuga M, et al. A randomized trial of nicotine vs. no-nicotine e-cigarettes among African American smokers: changes in smoking and tobacco biomarkers. Nicotine & Tobacco Research 2022;24(4):555-63. [DOI: 10.1093/ntr/ntab212] 34669956

[410] NCT03084315. Changes in biomarkers associated with use of electronic cigarettes [Changes in biomarkers associated with use of electronic cigarettes among African American menthol and non-menthol smokers]. clinicaltrials.gov/ct2/show/NCT03084315 (first received 20 March 2017).

[411] Smith TT, Heckman BW, Wahlquist AE, Cummings KM, Carpenter MJ. The impact of e-liquid propylene glycol and vegetable glycerin ratio on ratings of subjective effects, reinforcement value, and use in current smokers. Nicotine & Tobacco Research 2020;22(5):791-7. 10.1093/ntr/ntz130 PMC7171278 31403695

[412] Stein MD, Caviness C, Grimone K, Audet D, Anderson BJ, Bailey GL. An open trial of electronic cigarettes for smoking cessation among methadone-maintained smokers. Nicotine & Tobacco Research 2016;18(5):1157-62. 10.1093/ntr/ntv267 PMC5896835 26712843

[413] Strasser AA, Souprountchouk V, Kaufmann A, Blazekovic S, Leone F, Benowitz NL, et al. Nicotine replacement, topography, and smoking phenotypes of e-cigarettes. Tobacco Regulatory Science 2016;2(4):352-62. 10.18001/TRS.2.4.7 PMC5142626 27942543

[414] Wadia R, Booth V, Yap HF, Moyes DL. A pilot study of the gingival response when smokers switch from smoking to vaping. British Dental Journal 2016;221(11):722-6. 10.1038/sj.bdj.2016.914 27932811

