# Supplementary material for: Electronic cigarettes for smoking cessation
Source: Cochrane Database Syst Rev. 2025 Nov 10;2025(11):CD010216. doi: 10.1002/14651858.CD010216.pub10 (PMC12599494; doi:10.1002/14651858.CD010216.pub10)
Supplement: Supplementary file 1 — Supplementary material 1 Search strategies [file CD010216-SUP-01-searchStrategy.html]

Search strategies


# Supplementary material 1 to: Electronic cigarettes for smoking cessation

Lindson N, Livingstone-Banks J, Butler AR, McRobbie H, Bullen CR, Hajek P, Wu AD, Begh R, Theodoulou A, Notley C, Rigotti NA, Turner T, Fanshawe T, Hartmann-Boyce J
  
https://doi.org/10.1002/14651858.CD010216.pub10

The material in this section has been supplied by the author(s) for publication under a Licence for Publication and the author(s) are solely responsible for the material. Cochrane has peer reviewed this material in accordance with its editorial policies, but Cochrane has not copyedited, formatted or proofread. Cochrane accordingly gives no representations or warranties of any kind in relation to, and accepts no liability for any reliance on or use of, such material.

Back to top

# Search strategies

## Search strategies - 2020 update onwards

**Ovid databases (MEDLINE, Embase, PsycINFO)**

1. exp case control studies/ or exp cohort studies/ or Case control.tw. or (cohort adj (study or studies)).tw. or Cohort analy$.tw. or (Follow up adj (study or studies)).tw. or (observational adj (study or studies)).tw. or Longitudinal.tw.

2. (e-cig$ or ecig$ or electr$ cigar$ or electronic nicotine).mp. or (vape or vapes or vaporizer or vapourizer or vaporiser or vapouriser or vaper or vapers or vaping).ti,ab. or exp Electronic Nicotine Delivery Systems/

3. (randomized controlled trial or controlled clinical trial).pt. or randomized.ab. or placebo.ab. or clinical trials as topic.sh. or randomly.ab. or trial.ti.

4. exp animals/ not human/

5. 3 not 4

6. 2 and 5

7. 1 and 2

8. 6 or 7

9. smoking cessation.mp. or exp Smoking Cessation/

10. tobacco cessation.mp. or "Tobacco-Use-Cessation"/

11. (nicotine dependence or tobacco dependence).mp.

12. exp Smoking/th

13. "Tobacco-Use-Disorder"/

14. Smoking reduction/ or Smoking reduction.mp.

15. exp Pipe smoking/ or exp Tobacco smoking/ or exp Tobacco Products/

16. ((quit$ or stop$ or ceas$ or giv$ or abstain\* or abstinen\*) adj5 (smoking or smoke\* or tobacco)).ti,ab.

17. exp Tobacco/ or exp Nicotine/

18. 9 or 10 or 11 or 12 or 13 or 14 or 15 or 16 or 17

19. 8 and 18

**CENTRAL (via CRS-Web), (also used to search CTAG Specialised Register until March 2023)**

1. (e-cig\* or ecig\* or electr\* cigar\* or electronic nicotine):ti,ab,KY,MH,EMT,KW,XKY,EH,KY

2. (vape or vapes or vaporizer or vapourizer or vaporiser or vapouriser or vaper or vapers or vaping):ti,ab,KY,MH,EMT,KW,XKY,EH,KY

3. MESH DESCRIPTOR Electronic Nicotine Delivery Systems EXPLODE ALL

4. #3 OR #2 OR #1

## MEDLINE search strategy - pre-2020

1. e-cig$.mp. [mp=title, abstract, original title, name of substance word, subject heading word, protocol supplementary concept, rare disease supplementary concept, unique identifier]
2. electr$ cigar$.mp.
3. electronic nicotine.mp.
4. (vape or vaper or vapers or vaping).ti,ab.
5. 1 OR 2 OR 3 OR 4

Identical terms used for other databases.

Line 4 added to search strategy for 2016 update.
