# Supplementary material for: Electronic cigarettes for smoking cessation
Source: Cochrane Database Syst Rev. 2025 Nov 10;2025(11):CD010216. doi: 10.1002/14651858.CD010216.pub10 (PMC12599494; doi:10.1002/14651858.CD010216.pub10)
Supplement: Supplementary file 2 — Supplementary material 2 Characteristics of included studies [file CD010216-SUP-02-characteristicsOfIncludedStudies.html]

Characteristics of included studies


# Supplementary material 2 to: Electronic cigarettes for smoking cessation

Lindson N, Livingstone-Banks J, Butler AR, McRobbie H, Bullen CR, Hajek P, Wu AD, Begh R, Theodoulou A, Notley C, Rigotti NA, Turner T, Fanshawe T, Hartmann-Boyce J
  
https://doi.org/10.1002/14651858.CD010216.pub10

The material in this section has been supplied by the author(s) for publication under a Licence for Publication and the author(s) are solely responsible for the material. Cochrane has reviewed this material, but Cochrane has not copyedited, formatted or proofread. Cochrane accordingly gives no representations or warranties of any kind in relation to, and accepts no liability for any reliance on or use of, such material.

Back to top

# Characteristics of included studies

## Table of contents

- Studies ordered by Study ID
  - Adriaens 2014
  - Auer 2024
  - Avila 2024
  - Baldassarri 2018
  - Begh 2021
  - Bonafont Reyes 2022
  - Bonevski 2021
  - Bullen 2013
  - Caponnetto 2013a\*
  - Caponnetto 2013b\*
  - Caponnetto 2021\*
  - Caponnetto 2023\*
  - Carpenter 2017
  - Carpenter 2023
  - Cobb 2021
  - Coffey 2020
  - Czoli 2019
  - Dawkins 2020
  - Edmiston 2022\*
  - Edwards 2023
  - Eisenberg 2020
  - Eisenhofer 2015
  - Elling 2023
  - Ely 2013
  - Felicione 2019
  - George 2019
  - Goniewicz 2017
  - Hajek 2015a
  - Hajek 2019
  - Hajek 2022
  - Halpern 2018
  - Hatsukami 2020
  - Hickling 2019
  - Higgins 2024
  - Hoeppner 2024
  - Holliday 2019
  - Humair 2014
  - Ikonomidis 2018
  - Ikonomidis 2020a
  - Ikonomidis 2020b
  - Ikonomidis 2024
  - Ioakeimidis 2018
  - Kale 2025
  - Kanobe 2022\*
  - Katz 2025
  - Kerr 2020
  - Kimber 2021
  - Klonizakis 2022
  - Kouroutzoglou 2024
  - Kumral 2016
  - Lee 2018
  - Lee 2019
  - Lucchiari 2022
  - Martinez 2021
  - Martner 2019
  - McRobbie 2015
  - Meier 2017
  - Morphett 2022a
  - Morphett 2022b
  - Morris 2022\*
  - Myers-Smith 2022
  - NCT02648178
  - NCT02918630
  - NCT03113136
  - Nides 2014\*
  - Okuyemi 2022
  - Oncken 2015
  - Ozga-Hess 2019
  - Pacifici 2015
  - Pericot-Valverde 2025
  - Piper 2025
  - Polosa 2011\*
  - Polosa 2014b\*
  - Polosa 2015\*
  - Pope 2024
  - Pratt 2016
  - Pratt 2022
  - Price 2022
  - Pulvers 2018
  - Pulvers 2020
  - Rabenstein 2024
  - Rose 2023\*
  - Russell 2021\*
  - Scheibein 2020
  - Sifat 2024
  - Skelton 2022
  - Smith 2020
  - Smith 2025
  - Stein 2016
  - Strasser 2016
  - Tattan-Birch 2023
  - Tseng 2016
  - Tuisku 2024
  - Valentine 2018
  - Van Staden 2013\*
  - Vickerman 2022
  - Vojjala 2025
  - Wadia 2016
  - Wagener 2023
  - Walele 2018\*
  - Walker 2020
  - White 2022
  - Xu 2023\*
  - Yingst 2020
- Footnotes
- References to studies

## Studies ordered by Study ID

Adriaens 2014

| ***Study characteristics*** | | |
| Methods | Design: 3-armed RCT; with all participants then assigned to nicotine EC (treated as cohort in this review)  Recruitment: advertisement on university website, flyers on university campuses, emails to personnel, and advertisement in local newspaper  Setting: community and laboratory, Belgium  Study start date/end date: not stated | |
| Participants | Total N: 48 provided data  Randomized to: EC1 16; EC2 17; control 17  Inclusion criteria: smoke ≥ 3 yrs; ≥ 10 cpd; not intending to quit in the near future but willing to try a less unhealthy alternative  Exclusion criteria: diabetes; severe allergies; asthma or other respiratory diseases; psychiatric problems; dependence on chemicals other than nicotine; pregnancy; breastfeeding; hypertension; CV disease; currently using any kind of smoking cessation therapy; prior use of EC  56% women, mean age 44, mean cpd 19, mean FTCD 5.79, all unwilling to quit with no baseline EC use | |
| Interventions | **EC: Refillable**  **Intervention:** 2 intervention groups (EC1 and EC2) provided with EC and instructed to use EC or smoke ad libitum (EC1 group provided with Joyetech eGO-C, EC2 group provided with Kanger T2-CC) and provided guidance on EC use. For both types, provided 30 mL bottles of tobacco-flavoured e-liquid (Dekang “Turkish Blend”), containing 18 mg/mL of nicotine. 4 bottles at baseline replenished at 4 weeks, keep any remaining after 8 weeks  **Control:** 6 bottles for 2 months at week 8 (half offered EC1, half offered EC2); no guidance on use | |
| Outcomes | 3 lab sessions over 2 months (weeks 1, 4, and 8), plus online questionnaires, further follow-up at 3 and 6 m after last lab session  Cessation: measured but definition not provided, validated with eCO 5 ppm or less  Adverse events and biomarkers: eCO, salivary cotinine measured during lab sessions. Also collected craving and withdrawal symptoms via lab sessions, “benefits and complaints”, mood, EC usage | |
| Study funding | "No external funding for this study was obtained. Electronic cigarettes and e-liquids were purchased at E-cig4U (`t Rond 10, 4285 DE Woudrichem, The Netherlands; http://www.e-cig4u.nL) with balances of previous research funds obtained by Frank Baeyens." | |
| Author declarations | The authors declare no conflict of interest. | |
| Notes | Randomization was for short-term outcomes only.  Additional data provided from authors | |
| ***Risk of bias*** | | |
| **Bias** | **Authors' judgement** | **Support for judgement** |
| Random sequence generation (selection bias) | Low risk | Block randomization was performed by using a randomization tool available on the website www.randomizer.org. |
| Allocation concealment (selection bias) | Unclear risk | Not specified |
| Blinding of participants and personnel (performance bias) All outcomes | Low risk | Unblinded but as this review only included data on objective measurements and not cessation, judged unlikely to affect outcomes |
| Blinding of outcome assessment (detection bias) All outcomes | Low risk | Unblinded but as this review only included data on objective measurements and not cessation, judged unlikely to affect outcomes |
| Incomplete outcome data (attrition bias) All outcomes | Low risk | 36 out of 48 completed follow-up (11/16 in EC1 group, 12/17 in EC2 group, 13/17 in control group) |
| Selective reporting (reporting bias) | Unclear risk | Outcome reporting somewhat non-traditional; for example, collecting complaints but not explicitly adverse events, and incidence of AEs not reported. Unable to find prospectively registered protocol |

Auer 2024

| ***Study characteristics*** | | |
| Methods | Randomized, parallel-assignment, open-label trial  Setting: 5 sites in Switzerland: Bern, Geneva, Lausanne, St Gallen, Zurich  Recruitment: free and paid advertisements (lay press, social media, healthcare facilities, on public transport)  Study July 2018 to June 2021 (NCT record April 2022) | |
| Participants | Total: 1246 (intervention group 622, control group 624)  47% female; mean age 41.1 (median age 38 (IQR 29 to 51)); median cigarettes per day 15 (IQR 10 to 20); FTND 4.3 (SD 2.3)  Inclusion criteria:   - Informed consent as documented by signature - Persons aged 18 or older - Currently smoking 5 or more cigarettes a day for at least 12 months - Willing to try to quit smoking within the next 3 months - Persons providing a valid phone number, a valid email address, and/or a valid postal address   Exclusion criteria:   - Known hypersensitivity or allergy to contents of the e-liquid - Participation in another study with investigational drug within the 30 days preceding the baseline visit and during the present study where interactions are to be expected - Women who are pregnant or breastfeeding - Intention to become pregnant during the course of the scheduled study intervention, i.e. within the first 6 months of the study - Persons having used ENDS regularly in the 3 months preceding the baseline visit - Persons having used nicotine replacement therapy (NRT) or other medications with demonstrated efficacy as an aid for smoking cessation such as varenicline or bupropion within the 3 months preceding the baseline visit - Persons who cannot attend the 6-month follow-up visit for any reason - Cannot understand instructions delivered in person or by phone, or otherwise unable to participate in study procedures   E-cigarette use at baseline: not using e-cigarette regularly in last 3 months. 15.4% of the control and 17.2% of the intervention group had previously used an e-cigarette.  Motivated to quit: ‘wanted to set a quit date’. Eligible participants asked to set a quit date. | |
| Interventions | a) ENDS (vaporizer/e-cig) and smoking cessation counselling will receive:   - ENDS and nicotine-containing e-liquids, which they will be allowed to use ad libitum. Two e-cigarette starter kits (Innokin Endura T20-S) and 5 spare 0.8-ohm coils. Participants could choose among 6 flavours (2 tobacco, 1 menthol, and 3 fruity) and 4 nicotine concentrations (19.6 mg, 11 mg, 6 mg, and 0 mg per millilitre). Trial nurses gave participants no more than 10 e-liquid bottles at the baseline visit. - Smoking cessation counselling: provided in person at the first clinical visit and then over the phone at the target quit date 1 week later and again at weeks 2, 4, and 8 after the target quit date. After 6 months, participants will be asked to come to a final clinical visit. - Participants will be allowed to additionally use nicotine replacement therapy. NRT is not free.   b) Control group will receive smoking cessation counselling only as provided for a). Participants will be allowed to additionally use nicotine replacement therapy. | |
| Outcomes | Baseline and 6 months  Primary outcome: continuous smoking abstinence at 6 months post-quit date measured by:   - Self-report of having smoked no cigarettes from quit date, validated by urinary levels of anabasine. If anabasine is missing, validation by exhaled carbon monoxide (CO)   Secondary outcomes:   - Continuous smoking abstinence at 6 months post-quit date   - Self-report of having smoked no cigarettes from quit date, validated by urinary levels of NNAL (4-(methylnitrosamino)-1-(3-pyridyl)-1-butanol). If NNAL is missing, validation by urinary levels of anabasine or exhaled carbon monoxide (CO). - Self-reported smoking abstinence allowing a 2-week grace period at 4, 8 weeks, and 6 months post-quit date. - Validated smoking abstinence allowing a 2-week grace period at 6 months post-quit date:   - validated by urinary levels of anabasine; if anabasine is missing validation by exhaled CO;   - validated by urinary levels of NNAL (4-(methylnitrosamino)-1-(3-pyridyl)-1-butanol); if NNAL is missing, validation by urinary levels of anabasine or exhaled CO. - Self-reported smoking abstinence allowing up to 5 cigarettes at 1, 2, 4, 8 weeks, and 6 months post-quit date. - Validated smoking abstinence allowing up to 5 cigarettes at 6 months post-quit date:   - validated by urinary levels of anabasine; if anabasine is missing validation by exhaled CO;   - validated by urinary levels of NNAL (4-(methylnitrosamino)-1-(3-pyridyl)-1-butanol); if NNAL is missing, validation by urinary levels of anabasine or exhaled CO. - Self-reported 7-day PPA at 1, 2, 4, 8 weeks, and 6 months post-quit date. - Validated 7-day PPA at 6 months post-quit date   - Confirmation of having smoked no cigarettes in the past 7 days, validated by urinary levels of anabasine. If anabasine is missing validation by exhaled CO.   - Confirmation of having smoked no cigarettes in the past 7 days, validated by urinary levels of NNAL (4-(methylnitrosamino)-1-(3-pyridyl)-1-butanol). If NNAL is missing, validation by urinary levels of anabasine or exhaled CO. - Number of cpd at baseline, target quit date, 1, 2, 4, 8 weeks, and 6 months post-quit date, self-reported. - Change in number of cpd at baseline, 6 months post-quit date, self-reported. Successful reduction defined as 50% reduction in cpd. - Use of any other smoking cessation products (NRT) at 1, 2, 4, 8 weeks, and 6 months post-quit date, self-reported. - Withdrawal at baseline and 6 months assessed with the Minnesota Tobacco Withdrawal Scale (MTWS-R). - Fagerström Test for Nicotine Dependence at baseline and 6 months. - Swiss EQ-5D at baseline and 6 months. - Use of any ENDS at 1, 2, 4, 8 weeks, and 6 months post-quit date, self-reported. - Respiratory symptoms (COPD Assessment Test). - AEs defined as any untoward medical occurrence in participants who are administered an intervention during clinical investigation; these events were not necessarily caused by the intervention. Study nurses proactively collected AEs at each FU visit; participants could report AEs to the research team at any time. - SAE defined as any event that: required inpatient treatment of at least 24 hours or extended a current hospital stay; caused permanent or significant incapacity or disability; was life-threatening or caused death; caused a congenital anomaly or birth defect. Collected proactively at each FU visit. Participants could report SAEs at any time. - Study product use baseline to 6-month FU. | |
| Study funding | For Auer 2024: Supported by a grant (173552) from the Swiss National Sci[1]ence Foundation, a grant (19.017477) from the Swiss Tobacco Prevention Fund, a grant (KFS4744-02-2019) from Swiss Cancer Research, and Lunge Zürich. | |
| Author declarations | The funding bodies had no role in the trial design; the collection, monitoring, analysis, or interpretation of the data; or the writing of the manuscript. There was no industry involvement in the trial.  Reto Auer, Stephanie Baggio, Florent Baty, Aurelie Berthet, Philip Briggmann, Rodrigo casagrande Tango, Martin Feller, Anja Frei, Moa Haller, Nancy Hopf, Jean-Paul Humair,Isabelle Jacot Sadowski, Julian Jakob, Nicolas Rodondi, Nicolas Sambiagio, Anna Schoeni, Alexandra Strassmann, Mirah Stuber, Kali Tal do not have any interests to disclose.  Ivan Berlin: speaker at meetings (Pfizer). Member DSMB phase II trial Kinnov therapeutics.  Martin Brutsche: invited scientific talks (Astra Zeneca Schweiz). Scientific advisory board GlaxoSmithKline. Head of Department Kantonsspital. Consultant Merck Sharp & Dohme. Consultant Novartis Pharma AG.  See: https://www.nejm.org/doi/suppl/10.1056/NEJMoa2308815/suppl\_file/nejmoa2308815\_disclosures.pdf | |
| Notes | Linked trial registry IDs: NCT03603340; NCT03603353; NCT03612336; NCT03612375; NCT03612453; NCT03612544; NCT03632421; NCT03938298. Based on the common study name ESTxENDS study, we assume that the listed trial IDs and the numerous listed references are linked to one study. However, limited information is currently available and we will revisit this as necessary as more information becomes available.  The abstract reports "participants in the control group will receive smoking cessation counselling only. Participants will be allowed to additionally use NRT." As NRT was not provided by the study, we classed this comparator arm as "behavioural support only." | |
| ***Risk of bias*** | | |
| **Bias** | **Authors' judgement** | **Support for judgement** |
| Random sequence generation (selection bias) | Low risk | An automated, centralized, online randomization system in a protected environment at the Clinical Trials Unit in Bern, Switzerland, then generated randomization sequences in a 1:1 ratio. |
| Allocation concealment (selection bias) | Low risk | An automated, centralized, online randomization system in a protected environment at the Clinical Trials Unit in Bern, Switzerland, then generated randomization sequences in a 1:1 ratio. |
| Blinding of participants and personnel (performance bias) All outcomes | High risk | Blinding was not possible as a placebo was not provided in the comparator group |
| Blinding of outcome assessment (detection bias) All outcomes | Low risk | Cessation biochemically verified |
| Incomplete outcome data (attrition bias) All outcomes | Low risk | Data on smoking status and serious adverse events at 6 months were available for 90.8% of participants. Less than 20% difference and less than 50% attrition |
| Selective reporting (reporting bias) | Low risk | Outcomes reported |

Avila 2024

| ***Study characteristics*** | | |
| Methods | RCT  Center for Alcohol and Addiction Studies, Brown University School of Public Health, USA | |
| Participants | 45 participants  EC: N = 18; ONP: N = 18; CC: N = 9  Mean age 50.1 (SD 10.7); average CPD 13.9 (SD 10.1)  Inclusion criteria: household income < 250% federal poverty level (FPL); past 6 months daily smoking of ≥ 5 cigarettes/day (exhaled CO ≥ 6 ppm at baseline); willingness to substitute combustible cigarettes for EC or NPs; aged 21+ years  Exclusion criteria: intention to quit smoking during the next 30 days or current or past 30-day engagement in smoking cessation; current use of EC or NP ≥ 4 days per month or self-report of primarily using tobacco products that are not combustible cigarettes; hospitalization for a psychiatric issue in the past 30 days or visible instability; heart-related event in the past 30 days; pregnancy  Note: Cannabis use will be assessed but not excluded.  For full list, see NCT record.  Inclusion based specific population characteristic: lower socioeconomic status, household income < 250% federal poverty level  No EC use at baseline.  Not motivated to quit. | |
| Interventions | EC: 4th generation electronic cigarette device and disposable cartridges (5% nicotine e-liquid cartridges)  Nicotine pouch, 4 mg  Arm 1: Electronic cigarette  Participants in this experimental condition will be provided with a 4th generation EC device (VUSE Alto) and disposable cartridges. Participants will be provided with EC and 5% nicotine e-liquid cartridges (pods) for 8 weeks and encouraged at in-person and phone assessments to use the EC any time they would normally smoke. Participants will be able to choose one of two e-liquid flavours (tobacco, menthol) at baseline.  Arm 2: Nicotine pouch  Participants in this experimental condition will be provided with nicotine pouches.  Participants will be provided with on! 4 mg nicotine pouches for 8 weeks and encouraged at in-person and phone assessments to use the nicotine pouches any time they would normally smoke. Participants will be able to choose one of two nicotine pouch flavours (tobacco, mint) at baseline.  Arm 3: No intervention: smoking-as-usual  Participants in this assessment-only condition will continue smoking-as-usual.  All groups were advised to quit smoking at the end of the study with a brief advice and a handout on how to contact the Quitline. | |
| Outcomes | Baseline, 8 weeks  Change in cigarettes per day from baseline to week 8. Within and between-group difference in past week average cigarettes per day assessed using timeline follow-back (TLFB)  Change in cigarette dependence from baseline to week 8  Cigarette abstinence at week 8. Past week any-use of cigarettes assessed using timeline follow-back (TLFB)  Change in carbon monoxide; cotinine; NNAL; 8-isoprostane from baseline to week 8  Change in carbon monoxide reported.  Feasibility and acceptability | |
| Study funding | “The study is funded by the Brown University Office of Vice President Research. The authors JCA, DDM, and JSA are partially or fully funded by an NIH-funded Center of Biomedical Research Excellence (COBRE) (P20GM130414).” | |
| Author declarations | “JSA serves as a consultant and has equity in the start-up company Qnovia. This is a start-up company developing smoking cessation prescription medications through the FDA. They currently do not have a product that is available for sale. The other authors have no conflict of interest to disclose.” | |
| Notes | Avila 2024 moved to new study in 2025. NCT record (NCT05327439) was added to ongoing studies in 2022 update | |
| ***Risk of bias*** | | |
| **Bias** | **Authors' judgement** | **Support for judgement** |
| Random sequence generation (selection bias) | Low risk | “Participants were randomized using a computer-generated randomization program stratified by sex and age” |
| Allocation concealment (selection bias) | Low risk | “Randomization was conducted by the same re search assistants who enrolled participants in the study, and both participants and research assistants were not blinded to the study condition.” |
| Blinding of participants and personnel (performance bias) All outcomes | Low risk | 2 arms received equally intensive interventions (EC or pouch). Control arm not equally intensive. |
| Blinding of outcome assessment (detection bias) All outcomes | Low risk | CO - “assessed via Smokerlyzer” |
| Incomplete outcome data (attrition bias) All outcomes | Low risk | Low at 8 weeks: EC 14/18= 77.8%; ONP 12/18 = 66.7%; CC 7/9 = 77.8%. Data is at 8 weeks. [Would be high for ONP at 16 weeks] |
| Selective reporting (reporting bias) | High risk | Urine and blood samples were not analysed. |

Baldassarri 2018

| ***Study characteristics*** | | |
| Methods | Design: randomized, parallel-assignment, double-blind trial  Recruitment: outpatient pulmonary and primary care clinics, Tobacco Treatment Service, referrals from medical providers  Setting: Hospital outpatient and primary care clinics, USA  Study start date: October 2014; Study end date: June 2014 | |
| Participants | Total N: 40  N per arm: non-nicotine: 20; nicotine EC: 20  Inclusion criteria: ≥ 18 years; ≥ 1 cpd; willing to quit smoking  Exclusion criteria: unstable psychiatric or medical conditions requiring hospitalization within the past 4 months; acute coronary syndromes or stroke within the past 30 days; history of allergic reactions to adhesives; women who were pregnant, nursing, or not practising effective contraception; current use of an EC for the purpose of stopping tobacco cigarette smoking  Women: 52.5%; mean age: 53 mean cpd: 17 mean FTND: 5.9; motivated to quit  E-cigarette use at baseline: not reported | |
| Interventions | **EC: Refillable**  Both groups received standard care (8 weeks nicotine patch and counselling) and were randomized to **nicotine EC** or **non-nicotine EC**.  EC: eGO style EC (650 mAh battery, EVOD clearomizer, 3.7 V, 1.8 Ω single bottom coil), provided with e-liquid purchased from an online vape shop (0 mg/mL or 24 mg/mL nicotine strength, 70/30 propylene glycol/vegetable glycerin, tobacco flavour); instructed to use it as needed as a substitute for tobacco to try to satisfy cravings to smoke. If the patch alone proved adequate to prevent withdrawal and smoking cravings, the participant was advised not to use the EC. Additional EC devices, replacement coils, and liquid were provided as needed for the first 8 weeks of the study. | |
| Outcomes | Questionnaires and CO measurements taken at baseline, treatment visits at week 2, 4, 6, 8, and follow-up at week 24  Cessation: 7-day point prevalence abstinence, eCO ≤ 6 ppm  Adverse events and biomarkers: side effects were measured, although it is unclear whether a questionnaire with prespecified symptoms was used  Spirometry and FeNO at baseline and 6-month follow-up  Other outcomes: change in reported number of cpd at weeks 8 and 24; change in per cent predicted FEV1 and FVC from baseline to week 24, and EC use patterns | |
| Study funding | "Funding for this study was provided by the Yale School of Medicine, Section of Pulmonary, Critical Care, and Sleep Medicine and the National Heart, Lung, and Blood Institute grant T32HL007778. NHLBI had no role in the study design, collection, analysis, or interpretation of the data, writing the manuscript, or the decision to submit the paper for publication." | |
| Author declarations | "Dr. Toll received a grant from Pfizer for medicine only for a research study, and he receives funding as an expert witness in litigation filed against the tobacco industry. Dr. Chupp received grants from NIH, Genetech, Glaxo Smith Kline, Astra Zeneca/Medimmune and Boston Scientific. He received consulting/speaking fees from Genetech, Astra Zeneca/Medimmune, Mannkind, and Boston Scientific. There are no other conflicts of interest for the remaining authors." | |
| Notes | New for 2020 update. Study listed as ongoing study NCT02498145 in 2016 review update  Additional data provided from authors | |
| ***Risk of bias*** | | |
| **Bias** | **Authors' judgement** | **Support for judgement** |
| Random sequence generation (selection bias) | Low risk | Quote: “Participants were randomized using a random number generator with 1:1 blocked randomization (block size n = 8).” |
| Allocation concealment (selection bias) | Unclear risk | Both groups received standard care (nicotine patch and counselling) and were randomized to: nicotine EC or non-nicotine EC (no further detail given). |
| Blinding of participants and personnel (performance bias) All outcomes | Low risk | Quote: “Treatment assignment was blinded to both the investigators and participants”. |
| Blinding of outcome assessment (detection bias) All outcomes | Low risk | CO biochemically validated |
| Incomplete outcome data (attrition bias) All outcomes | High risk | Quote: “The study had a modest loss to follow-up (20%) at week 24.”  Number lost to follow-up in each group was not reported in the paper.  Week 24 retention rate: Nicotine EC group: 19/20 (95%); non-nicotine EC group: 13/20 (65%); > 20% difference between groups |
| Selective reporting (reporting bias) | Low risk | Outcomes reported align with those listed in the clinicaltrials.gov record (registered 2015; prior to study completion in 2016). |

Begh 2021

| ***Study characteristics*** | | |
| Methods | Individually randomized, blinded, 2-arm trial  Setting: 39 general practices, England  Recruitment: primary care registries | |
| Participants | 325 (164 intervention; 161 intervention)  47.4% female; mean age 57.8; mean cpd 20.1; mean FTCD 4.2  Inclusion criteria: current smoker ≥ 10 ppm for exhaled CO and smokes a minimum of 8 cigarettes/8 grams of tobacco per day (including pipe, cigars, or tobacco roll-ups) with no intention of stopping immediately or seeking cessation support. Diagnosed with 1 or more of the following chronic conditions: ischaemic heart disease, peripheral vascular disease, hypertension, diabetes mellitus (Type 1 and Type 2), stroke, asthma, COPD, chronic kidney disease, depression, schizophrenia, bipolar disorder or other psychoses. Informed consent. ≥ 18 years  Exclusion criteria: GP believes that switching to EC would not benefit the patient, given their current medical condition; currently using EC, NRT or other cessation therapies (e.g. bupropion, nortriptyline, or varenicline); plans to stop smoking before or at the annual review; currently enrolled in another smoking-related study or other study where the aims of the studies are incompatible; cannot consent due to mental incapacity; pregnancy, breastfeeding | |
| Interventions | EC type: refillable  Control: no additional support beyond standard care  Intervention: practitioners gave brief advice about EC and offered participants a free EC for the purpose of switching from smoking to vaping. The instruction was to reduce their smoking. If the offer was accepted, participants received: a starter pack containing an Aspire PockeX all-in-one e-cigarette, 2 x 0.6 ohm coils and 1 x 1.2 ohm coil, 3 nicotine e-liquids in 18 mg/mL (blueberry, menthol) and 12 mg/mL (mixed fruit) strengths and an accompanying practical support booklet developed by the study team. The practical support booklet contained information on how to set up the device, correct ways to vape, common issues with use and a list of local vape shops. It included motivational support to reinforce practitioners’ advice about EC, including the benefits of cutting down on cigarettes through e-cigarette use and addressing perceived risks and concerns. It included links to a study-dedicated website with video demonstrations on how to use EC and testimonials. Participants could opt into receiving an introductory telephone call from an experienced vaper in the first week of receiving their EC, to guide them on technical aspects of EC use (not behavioural support). Thereafter, participants could contact the vaper by telephone for up to 2 months after receiving their kit.  All: practitioners offered routine smoking cessation support to all participants. Although this varied across practices, standard care typically involved brief advice about stopping smoking and assistance to do so either by referral to the NHS stop-smoking services or offer of pharmacotherapy. If the participant declined standard care, they were randomized by the practitioner to either the intervention or control arm. In the control arm, participants received no further support beyond standard care. | |
| Outcomes | 0 months, consultation visit, 2 months post-consultation, 8 months post-consultation  "Patients attended four visits at their GP practice: a baseline visit, a therapeutic visit (‘annual review’) with their GP or nurse and two follow-up visits two months and eight months post-consultation."  Primary outcomes:   - 7-day PPA from smoked tobacco at 2 months, defined as complete self-reported abstinence from smoking – not even a puff – in the past 7 days, accompanied by a salivary anabasine concentration of < 1 ng/mL   If there are technical issues with the analysis of saliva samples (e.g. if there is not enough saliva present in the sample for anabasine analysis), we will use exhaled CO as verification of abstinence (CO < 10 ppm).  (Deviation from Statistical Analysis Plan: CO used due to imprecision of values for anabasine)   - Reduction in cigarette consumption at 2 months, defined as at least a 50% reduction in self-reported cigarettes per day on each of the last 7 days at 2 months compared with baseline consumption, accompanied by evidence of reduced smoke intake indicated by a CO measurement lower than baseline   Secondary outcomes:   - 7-day PPA measured at 8 months, biochemically confirmed by an exhaled CO of < 10 ppm - 6-month prolonged abstinence using the Russell standard criteria, defined as smoking < 5 cigarettes between 2- and 8-month follow-ups, confirmed by an anabasine concentration of < 1 ng/mL at 2 months and an exhaled CO concentration of < 1 ng/mL at 8 months if CO measurement unavailable - Mean change in salivary anabasine concentration and CO from baseline to 2 months - Percentage reduction in self-reported cigarettes per day from baseline to 2 months; and from baseline to 8 months   SAEs and AEs reported. AEs: throat/mouth irritation; cough; headache; palpitations; nausea; dry mouth; dizziness; shortness of breath; stomach pain | |
| Study funding | NIHR Postdoctoral Fellowship and NIHR School for Primary Care Research funded randomized controlled trial | |
| Author declarations | All authors declare no competing interests. | |
| ***Risk of bias*** | | |
| **Bias** | **Authors' judgement** | **Support for judgement** |
| Random sequence generation (selection bias) | Low risk | Quote: "Participants were randomised to intervention or control with a 1:1 allocation ratio. A randomisation list was generated by the trial statistician using the current version of Stata and validated by a second statistician within the Primary Care Clinical Trials Unit (PC-CTU). The randomisation was stratified by practice and used varying block sizes to ensure allocation concealment." |
| Allocation concealment (selection bias) | Low risk | Quote: "The randomisation list was passed to someone independent of the trial who created the randomisation envelopes. The trial statisticians were blinded to the treatment allocation during analyses." |
| Blinding of participants and personnel (performance bias) All outcomes | High risk | Due to the nature of the trial, GPs and practice nurses were aware of the participant’s treatment allocation to ensure that the correct intervention was given. Therefore, practitioners who delivered the intervention could not be blinded to treatment.  While participants knew whether they had been offered support to cut down by using an e-cigarette or not by their GP or nurse, the participant was not informed that the study investigated this specifically and, therefore, was in some respects blind to allocation.  Groups not matched for face-to-face contact time |
| Blinding of outcome assessment (detection bias) All outcomes | Low risk | Quote: “7-day point-prevalence abstinence from smoked tobacco at two months, defined as complete self-reported abstinence from smoking – not even a puff – in the past seven days, accompanied by a salivary anabasine concentration of < 1 ng/mL” or exhaled CO as verification of abstinence (CO < 10 ppm)" |
| Incomplete outcome data (attrition bias) All outcomes | Low risk | At 8 months: control 144/161; intervention 148/164 |
| Selective reporting (reporting bias) | Low risk | Used CO above anabasine, but reported both  (Deviation from Statistical Analysis Plan: CO used due to imprecision of values for anabasine)  All predefined outcomes listed in the published protocol and clinical trial register were reported. |

Bonafont Reyes 2022

| ***Study characteristics*** | | |
| Methods | Design: "A mixed methods study"  Recruitment: recruited patients with COPD, aged 21 to 75, listed as current smokers in the NYU Langone Health electronic health record by phone, mail, and MyChart  Participants: patients with COPD  Setting: NYU, USA  Study start date: not reported | |
| Participants | Total N: 48  Inclusion criteria: moderate COPD (based on the COPD Assessment Test score (CAT)); interested in quitting  Exclusion criteria: not reported  Female 54%; mean age 60 (SD 8.2)  E-cigarette use at baseline: not reported  Motivated to quit: yes | |
| Interventions | EC: no detail reported  Arm 1 EC  Arm 2 NRT  Both groups: over 12 weeks, participants received 5 counselling sessions and were asked about their COPD symptoms, CC use, EC use, and nicotine withdrawal symptoms. We used Ecological Momentary Assessment (four text messages/day) to assess current EC/NRT and CC use. | |
| Outcomes | 12 weeks  Combustible cigarette use measured  Dyspnoea  COPD symptoms  Mixed methods study assessing the relationship between race/ethnicity and switching from CC to EC; evaluated whether it is mediated by social norms, risk perception, and overall opinions of CC and EC | |
| Study funding | Not reported | |
| Author declarations | Not reported | |
| Notes | Student presentation  New to 2022 update | |
| ***Risk of bias*** | | |
| **Bias** | **Authors' judgement** | **Support for judgement** |
| Random sequence generation (selection bias) | Unclear risk | No detail: “We randomized participants to EC or nicotine replacement therapy (NRT) for switching from CC.” |
| Allocation concealment (selection bias) | Unclear risk | No detail |
| Blinding of participants and personnel (performance bias) All outcomes | Unclear risk | Two active interventions. No detail |
| Blinding of outcome assessment (detection bias) All outcomes | Unclear risk | No detail |
| Incomplete outcome data (attrition bias) All outcomes | Unclear risk | Still collecting data. Outcome data not reported. Numbers not reported |
| Selective reporting (reporting bias) | Unclear risk | Still collecting data |

Bonevski 2021

| ***Study characteristics*** | | |
| Methods | Design: pragmatic, open-label, single-centre, 2-arm randomized controlled trial  Recruitment: withdrawal service in Melbourne, Australia  Setting: substance use disorder treatment setting, and following discharge, community setting, Melbourne, Australia  Study start date: 1 August 2017; Study end date: April 2019 | |
| Participants | Total N: 100  N per arm: EC intervention = 50; NRT control = 50  Inclusion criteria: ≥ 18 years; tobacco smoker on entering the residential service; capacity to consent and able to understand the participant materials  Exclusion criteria: used an END containing nicotine in the past month; pregnancy/breastfeeding; currently enrolled in another study; scheduled to be transferred to a long-term rehabilitation unit following discharge from the residential withdrawal unit.  Inclusion based on specific population characteristics: participants were discharged from a smoke-free alcohol or other drugs (AOD) residential withdrawal service.  32% women; mean age 40.9; mean cpd 21  Motivated to quit: median (SD) = 7.3 (2.4) on the 1 to 10 scale with 10 "highly motivated" | |
| Interventions | **EC**: Refillable  Up to an hour training session, information pack. Innokin Endura T22 starter kit and refill liquid (Nicophar). 4-week supply of liquid nicotine, with further supplies of liquid nicotine mailed twice at 4- week intervals. Dosing schedule of e-liquid dependent nicotine dependence score: high-nicotine-dependence category assigned initial 4-week e-liquid supply (total 8 × 10 ml bottles) consisting of: 2 × 10 ml bottles of 18 mg e-liquid and 6 × 10 ml bottles of 12 mg e-liquid. The second and third batches = 8 × 10 ml bottles of 12 mg e-liquid only. Participants scoring in the moderate- and low-dependence categories: three 4-week supplies of 8 × 10 ml bottles of 12 mg e-liquid. Participants given 1-week supply of nicotine patches for use while getting used to the EC.  **NRT control**: information pack, 12 weeks NRT on the same schedule as for ENDs. 4-week supply of patches plus a nicotine spray and inhaler, followed by refills including patches plus inhaler, gum, and lozenges.  Both groups received proactive referral to quitline counselling (call-back service), which provides calls on pre-discharge and on days 1, 3, 7, 14, and 28 post-discharge, with an emphasis on relapse prevention. Counsellors trained on the use of ENDs | |
| Outcomes | Week 6, 12; self-report  Adverse events collected  Other outcomes measured:   - Acceptability and feasibility of interventions - Treatment adherence - Cigarettes smoked per day - Heaviness of Smoking Index - Frequency of cravings - Minnesota Nicotine Withdrawal Scale (MNWS) - 10-item Kessler Psychological Distress Scale (Kessler-10) - Quitting self-efficacy, motivation to quit and the Heaviness of Smoking Index were assessed at baseline. | |
| Study funding | "The study is supported by a VicHealth Innovation Research Grant (2016–0096). AG is supported by a post-doctoral fellowship from the Heart Foundation. ALB is supported by an Australian National Health and Medical Research Council (NHMRC) senior research fellowship and a Faculty of Health and Medicine, University of Newcastle Gladys M Brawn senior research fellowship. BB is supported by an Australian NHMRC career development fellowship (GNT1063206) and a Faculty of Health and Medicine, University of Newcastle Gladys M Brawn career development fellowship."  "This study was supported by a VicHealth Innovation Research Grant (2016-0096)." | |
| Author declarations | "The authors declare that they have no competing interests."  "None to declare." | |
| Notes | New for 2020 update; additional data originally provided by authors and subsequently published | |
| ***Risk of bias*** | | |
| **Bias** | **Authors' judgement** | **Support for judgement** |
| Random sequence generation (selection bias) | Low risk | Quote: “Upon completing the baseline survey, participants were randomised 1:1 to an intervention via a computer-sequenced 4–6 block randomisation embedded in the tablet device software.” |
| Allocation concealment (selection bias) | Low risk | Quote: “At the end of the baseline survey, participants will be randomised 1:1 to an intervention via a computer-sequenced 4–6 block randomisation embedded in the iPad.” |
| Blinding of participants and personnel (performance bias) All outcomes | High risk | Quote: “Participants were informed of their intervention group by the RA and provided with a training session of up to one hour.”  “Due to the nature of the intervention, neither participants nor staff can be blinded to allocation. However, the data safety monitoring committee and the statistician responsible for the data analysis will be blinded.” |
| Blinding of outcome assessment (detection bias) All outcomes | High risk | No biochemical validation, self-report data |
| Incomplete outcome data (attrition bias) All outcomes | Low risk | Quote: “At 6 and 12-weeks, 63 participants (63%) and 50 participants (50%) were followed up, respectively. While slightly higher retention rates were evidenced in the VNP group at 6-weeks (68% vs 58% in NRT group; P = 0.30), there were no differences between groups at 12-weeks (25 re-contacted in both arms; i.e. 50%).” |
| Selective reporting (reporting bias) | Low risk | Unpublished findings provided by authors reported on all outcomes mentioned in the protocol. |

Bullen 2013

| ***Study characteristics*** | | |
| Methods | Design: 3 parallel groups RCT  Recruitment: people who smoke recruited from the community, via newspaper advertisements  Setting: research unit, New Zealand  Study start date: 6 September 2011; study end date: 5 July 2013 | |
| Participants | Total N: 657. 289 nicotine EC (NEC), 295 patch, 73 non-nicotine EC (PEC)  Inclusion criteria: ≥ 18 years; smoked 10 or more cpd over past year; wanted to stop smoking  Exclusion criteria: pregnancy and breastfeeding; using cessation medicines or using other support to quit; heart attack, stroke, severe angina in the last 2 weeks; poorly controlled medical disorder; allergies, other chemical dependence  62% women, mean age 42, ⅓ NZ Maori, smoking 18 cpd, mean FTND score 5.5  Motivated to quit  E-cigarette use at baseline: not specified | |
| Interventions | **EC: Cig-a-like**  Randomized to NEC, PATCH, or PEC use for 13 weeks (from 1 week prior to TQD)   - **NEC:** Elusion brand 16 mg cartridges; sent product via courier - **PATCH:** 21 mg/24-hour patch; sent voucher to exchange for NRT at pharmacy (dispensing costs covered) - **PEC:** as per EC, but 0 mg cartridges   All participants referred to Quitline and received an invitation to access phone- or text-based support. This was accessed by ≤ 40% of participants. | |
| Outcomes | Sustained (≤ 5 cigarettes allowed) validated (exhaled breath CO < 10 ppm) abstinence at 6 months  ≥ 50% self-reported reduction in baseline cigarettes at 6 months  Participants reporting any adverse events  Proportion of AEs that were serious  Proportion of unrelated AEs | |
| Study funding | Health Research Council of New Zealand | |
| Author declarations | "We declare that we have received no support from any companies for the submitted work and have no non-financial interests that might be relevant to the submitted work. ML, via his company Health New Zealand, previously did research funded by Ruyan (an e-cigarette manufacturer). CB and HM have done research on Ruyan e-cigarettes funded by Health New Zealand, independently of Ruyan. HM has received honoraria for speaking at research symposia, has received benefits in kind and travel support from, and has provided consultancy to, the manufacturers of smoking cessation drugs. NW has provided consultancy to the manufacturers of smoking cessation drugs, received honoraria for speaking at a research meeting and received benefits in kind and travel support from a manufacturer of smoking cessation drugs. JW has provided consultancy to the manufacturers of smoking cessation medications." | |
| Notes | Accessed support: NEC: 115/289; PATCH: 106/295; PEC: 26/73 | |
| ***Risk of bias*** | | |
| **Bias** | **Authors' judgement** | **Support for judgement** |
| Random sequence generation (selection bias) | Low risk | Computerized block randomization |
| Allocation concealment (selection bias) | Low risk | Computerized via study statistician |
| Blinding of participants and personnel (performance bias) All outcomes | Low risk | NEC and PEC were blind to treatment condition in relation to one another. No blinding for NEC/PEC vs PATCH conditions, but as NEC and PATCH were both active treatments, performance bias judged unlikely |
| Blinding of outcome assessment (detection bias) All outcomes | Low risk | Biochemical validation used |
| Incomplete outcome data (attrition bias) All outcomes | Low risk | 22% lost to follow-up (all considered to be smoking). Patch group had a higher loss to follow-up and withdrawal than EC (loss to follow-up 17% NEC, 27% patches, 22% PEC). However, minimal differences in per-protocol and ITT analyses |
| Selective reporting (reporting bias) | Low risk | All prespecified outcomes reported |

Caponnetto 2013a\*

| ***Study characteristics*** | | |
| Methods | Design: 3-arm, double-blind randomized controlled trial: EC with 7.2 mg nicotine for 12 weeks; same for 6 weeks followed by 5.2 mg for 6 weeks: EC with no nicotine for 12 weeks  Recruitment: newspaper advertisements  Setting: outpatient clinic, Italy  Study start date: April 2010; Study end date: April 2012 | |
| Participants | Total N: 300  Inclusion criteria: smoked ≥ 10 cpd for past 5 years; age 18 to 70; in good health; not currently or intending to quit smoking in the next 30 days  Exclusion criteria: symptomatic cardiovascular or respiratory disease; regular psychotropic medicine use; current or past history of alcohol abuse; use of smokeless tobacco or NRT; pregnancy or breastfeeding  36% women; mean age 44 (SD 12.5); mean cpd 20 (IQR: 15 to 25)  Not currently or intending to quit smoking in the next 30 days  E cigarette use at baseline: not specified | |
| Interventions | **EC: Cig-a-like**  EC presented as a healthier alternative to tobacco smoke and could be freely used, ad libitum (up to 4 cartridges a day) for 12 weeks, as a tobacco substitute  EC used: 'Categoria' (model 401) with disposable cartridges   - **Grp A**: 12 weeks of 7.2 mg capsules ('Original') - **Grp B**: 6 weeks 7.2 mg ('Original'), then 6 weeks 5.4 mg ('Categoria') - **Grp C**: 12 weeks of 0 mg ('Original')   Baseline visit and up to 7 follow-up visits to receive more cartridges, hand-in diaries, measure CO and vital signs | |
| Outcomes | Abstinence at 12 months (complete self-reported abstinence from tobacco smoking since previous visit at 6 months, confirmed with CO < 7 ppm at 12 months)  ≥ 50% reduction in baseline cigarettes at 12 months  Recorded AEs thought to be related to tobacco smoking and EC at baseline and at each study visit (7 follow-up visits over 12 weeks, plus at 24 and 52 weeks) | |
| Study funding | "This research was supported by a grant-in-aid from Lega Italiana AntiFumo. The study sponsor had no involvement in the study design, collection, analysis, and interpretation of data, the writing of the manuscript or the decision to submit the manuscript for publication. RP and PC are currently funded by the University of Catania, Italy. The e-cigarette supplier had no involvement in the study design, collection, analysis, and interpretation of data, the writing of the manuscript or the decision to submit the manuscript for publication." | |
| Author declarations | "RP has received lecture fees and research funding from Pfizer and GlaxoSmithKline, manufacturers of stop smoking medications. He has served as a consultant for Pfizer and Arbi Group Srl, the distributor of the CategoriaTM e-Cigarette. The other authors have no relevant conflict of interest to declare in relation to this work." | |
| Notes | Additional data provided from authors | |
| ***Risk of bias*** | | |
| **Bias** | **Authors' judgement** | **Support for judgement** |
| Random sequence generation (selection bias) | Low risk | Computer-generated, block size 15 (5:5:5 ratio) |
| Allocation concealment (selection bias) | Low risk | Randomization carried out by pharmacy, which did not have direct contact with the participants |
| Blinding of participants and personnel (performance bias) All outcomes | Low risk | Double-blind  Quote: “Blinding was ensured by the identical external appearance of the cartridges. The hospital pharmacy was in charge of randomization and packaging of the cigarettes”. |
| Blinding of outcome assessment (detection bias) All outcomes | Low risk | Biochemical validation used |
| Incomplete outcome data (attrition bias) All outcomes | Low risk | 211 (70.3%) and 183 (61%) attended 6- and 12-month follow-up (at 12 m, 35% lost in 7.2 group; 37% lost in 5.4 group; 45% lost in no-nicotine group). |
| Selective reporting (reporting bias) | Unclear risk | Unclear if original intention was to combine groups A + B or not. In sample size calculation, they compared A + B with C, but results were not always reported in this way. |

Caponnetto 2013b\*

| ***Study characteristics*** | | |
| Methods | Design: prospective cohort  Recruitment and setting: inpatients at a psychiatric institution in Italy  Study start date/end date: not specified | |
| Participants | Total N: 14  Inclusion criteria: smoked ≥ 20 cpd for at least the past 10 years; diagnosis of schizophrenia  Exclusion criteria: alcohol and illicit drug use; recent myocardial infarction; angina pectoris; high blood pressure (BP > 140 mmHg systolic or 90 mmHg diastolic, or both); diabetes mellitus; severe allergies; poorly controlled asthma or other airway diseases; inclusion based on specific population characteristic: diagnosis of schizophrenia  57% women; mean age 44.6 (SD 12.5); mean pack-years smoked 28.8 (SD 12.9)  Motivated to quit: not motivated to quit  E cigarette use at baseline: not specified | |
| Interventions | **EC: Cig-a-like**  Seen at baseline, given EC ('Categoria' brand) with an initial 4-week supply of 7.4 mg nicotine cartridges. Instructed to use ad libitum up to 4 cartridges a day. EC cartridges supplied at months 1, 2, and 3  No instruction on cessation or reduction was provided. | |
| Outcomes | Follow-up at 1, 2, 3, 6, and 12 months when cigarette consumption, CO, AEs, and positive and negative symptoms of schizophrenia were measured  Sustained reduction of ≥ 50% for at least 30 days at 12 months  30-day point prevalence CO-validated abstinence at 12 months  Adverse events | |
| Study funding | "We wish to thank Arbi Group Srl (Milano, Italy) for the free supplies of “Categoria” e-cigarette kits and nicotine cartridges as well as their support. We would also like to thank LIAF (Lega Italiana AntiFumo) for the collaboration." | |
| Author declarations | "Pasquale Caponnetto, Roberta Auditore, Cristina Russo and Giorgio Carlo Cappello declare no conflict of interest. Riccardo Polosa has received lecture fees and research funding from Pfizer and GlaxoSmithKline, manufacturers of stop smoking medications. He has served as a consultant for Pfizer and Arbi Group Srl (Milano, Italy), the distributor of the CategoriaTM e-cigarette." | |
| ***Risk of bias*** | | |
| **Bias** | **Authors' judgement** | **Support for judgement** |
| Random sequence generation (selection bias) | High risk | Prospective cohort; no randomization |
| Allocation concealment (selection bias) | High risk | Not randomized |
| Incomplete outcome data (attrition bias) All outcomes | Low risk | 0/14 lost to follow-up |
| Selective reporting (reporting bias) | Unclear risk | Unable to determine prespecified outcomes |

Caponnetto 2021\*

| ***Study characteristics*** | | |
| Methods | Design: single-arm pilot study  Recruitment: authors wrote to physicians, psychiatrists, and other health care providers to inform them about the study. Flyers were posted within and outside of the Smoking Cessation Center of Catania University (Centro per la Prevenzione e Cura del Tabagismo — CPCT), at the Policlinico Vittorio Emanuele. Participants were recruited from Catania outpatient psychiatric clinics by researchers of CPCT. Clinicians from outpatient psychiatric clinics identified suitable participants and drew their attention to the study flyers.  Setting: Catania, Italy  Study start date: 2017. End date not stated. Recruitment September 2017 to October 2017 | |
| Participants | Total N: 40 (single-arm)  All participants: individuals with schizophrenia spectrum disorders who smoke cigarettes  Inclusion criteria:  Adults attending psychiatric outpatient clinics in Catania who smoked 20 or more cigarettes daily were included.  Able to meet the criteria for a schizophrenia spectrum disorder diagnosis without evidence of current exacerbation of illness  Exclusion criteria:  Pregnancy, breastfeeding, myocardial infarction, or angina pectoris within the past 3 months, current poorly controlled asthma or chronic obstructive pulmonary disease  Female 35%; mean age 48.3 (SD 12.1); mean CPD 28 (SD 9); mean FTND 8.3 (SD 1.8)  E-cigarette use at baseline: 12 (30%) had used EC either regularly or had tried.  Motivated to quit: no | |
| Interventions | EC: **pod**  JUUL pod e-cigarette, PAX Labs, a closed pod e-cigarette product. The pod contains 0.7 mL of e-liquid and up to 5% nicotine by weight.  At the BL visit, participants were given a free e-cigarette starter kit containing one JUUL device with a charger and 5% nicotine pods, Virginia tobacco flavour with instructions on how to charge, activate, and use the e-cigarette. A 4-week supply of pods equivalent to their current cigarette-smoking behaviour, according to the manufacturer’s guidelines, was supplied to each participant (one pod for every packet of 20 cigarettes; mean 128, minimum 80, and maximum 200).  Support: Eligible participants were invited to use a JUUL e-cigarette for at least 12 weeks and were followed up prospectively for 24 weeks. Participants received a 4-week supply of pods on three occasions, BL, week 4 (study visit 2), and week 8 (study visit 3). Participants were informed that the product was potentially less harmful than combustible cigarettes and could be used as a cigarette substitute as much as they liked. Limited behavioural support was provided as part of the intervention and included behaviour substitution of combustible cigarettes with e-cigarettes and self-monitoring of combustible cigarette consumption through the use of study diaries. Phone contact at week 2, 6, and 10. Participants attended a total of 5 study visits. | |
| Outcomes | Baseline, week 4 (study visit 2), week 8 (study visit 3), 12 weeks, 24-week FU  Cessation: CO  Reduction: CPD  AEs: dry cough, headache, throat irritation  Other outcomes: vital signs (BP and HR), weight, and mental health, subjective effects (e.g. satisfied, no aversion), acceptability | |
| Study funding | The e-cigarettes used in the study were donated by the manufacturer, PAX Labs (on June 13, 2017 the company became known as JUUL Labs).  Acknowledgements: The authors wish also to thank PAX Labs (on June 13, 2017 the company became known as JUUL Labs) for the free supplies of JUUL e-cigarette kits and pods. At the time the research was conducted, JUUL Labs were not part owned by Altria, a tobacco company. PAX Labs agreed also to supply pods for a further 3 months after the end of the pilot to participants who expressed a wish to continue using as JUUL was not available in Italy when this study was conducted and not currently available at the 5% nicotine strength. No separate funding was secured for the study.  Altria Group (formerly Philip Morris Companies) acquired a 35% stake in JUUL Labs on December 20, 2018, but the study was completed before Altria invested in JUUL. | |
| Author declarations | MM is fixed-term researcher at Centro per la Prevenzione e Cura del Tabagismo, University of Catania. JD is full-time employee of City University of New York (United States). JK is full-time employee of Weill Medical College of Cornell University, New York (United States). RP is full-time employee of the University of Catania, Italy. In relation to his work in the area of tobacco control and respiratory diseases, RP has received lecture fees and research funding from Pfizer, Inc., GlaxoSmithKline plc, CV Therapeutics, NeuroSearch A/S, Sandoz, MSD, Boehringer Ingelheim, Novartis, Duska Therapeutics, and Forest Laboratories. He has also served as a consultant for Pfizer, Inc., Global Health Alliance for treatment of tobacco dependence, CV Therapeutics, NeuroSearch A/S, Boehringer Ingelheim, Duska Therapeutics, Forest Laboratories, ECITA (Electronic Cigarette Industry Trade Association, in the United Kingdom), Health Diplomat (consulting company that delivers solutions to global health problems with special emphasis on harm minimization), and Pharmacielo. RP was awarded an Investigator-Initiated Study award programme established by Philip Morris International in 2017, but subsequently resigned from the role of Principal Investigator in 2018, before the trial began. Lecture fees from a number of European EC industry and trade associations (including Fédération Interprofessionnelle de la VAPE in France and Federazione Italiana Esercenti Svapo Elettronico in Italy) were directly donated to vaper advocacy no-profit organizations. RP is the Founder of the Center of Excellence for the acceleration of Harm Reduction at the University of Catania (CoEHAR), which has received a grant from Foundation for a Smoke Free World to develop and carry out eight research projects. RP is also currently involved in the following pro bono activities: scientific advisor for LIAF, Lega Italiana Anti Fumo (Italian acronym for Italian Anti Smoking League) and Chair of the European Technical Committee for standardization on Requirements and test methods for emissions of electronic cigarettes (CEN/TC 437; WG4). PC is paid by the University of Catania as an external part-time researcher and adjunct professor of clinical, addiction, and general psychology. He has been affiliated to the CoEHAR since December 2019 in a pro bono role. He is coauthor of a protocol paper supported by an Investigator-Initiated Study award programme established by Philip Morris International in 2017. The other authors have no conflict of interests to declare. | |
| Notes | New to 2022 update | |
| ***Risk of bias*** | | |
| **Bias** | **Authors' judgement** | **Support for judgement** |
| Random sequence generation (selection bias) | High risk | Single-arm, open-label |
| Allocation concealment (selection bias) | High risk | Single-arm, open-label |
| Incomplete outcome data (attrition bias) All outcomes | Low risk | Quote: 37 (92.5%) participants completed all study visits and attended their follow-up visit. |
| Selective reporting (reporting bias) | Unclear risk | No detail |

Caponnetto 2023\*

| ***Study characteristics*** | | |
| Methods | Design: randomized non-inferiority switching trial  Setting: Italy  Recruitment: recruited among hospital/university staff, via social media, or through word of mouth  Study start date: May 2019. Study end date May 2020 | |
| Participants | 220 healthy people who smoke tobacco cigarettes  EC arm 110, HTP arm 110  % female: EC arm 70% (63.6), HTP arm 56% (50.9). Mean age 41.3 (EC arm 41.3 (SD 16.9), HTP arm 41.3 (SD 16.1)). Mean CPD: EC arm 22.8 (10.9), HTP arm 22.6 (SD 10.1). Mean FTND: EC arm 6 (SD 2.2), HTP arm 5.8 (SD 2.1)  Motivated to quit: No | |
| Interventions | E-cigarettes (EC) arm: JustFog Q16 Starter Kit consisting of a battery and a 1.9-mL refillable tank fitted with a 1.6-Ohm nichrome coil, 16 mg, 3 flavours 50% propylene glycol/40% vegetable glycerin/10% H2O. EC study arm, 50.9% (56/110) chose Puff Riserva Country, 30.9% (34/110) chose Puff Riserva Tuscan, and 18.2% (20/110) chose Puff Artic e-liquid.  Heated Tobacco Products (HTPs) arm: IQOS 2.4 Plus consisting of a pen-like holder into which a tobacco stick is inserted and heated, and a battery case to recharge the holder after each use. IQOS 2.4 Plus was the only HTP available on the Italian market when this trial was designed. The device is to be used with tobacco sticks specifically processed and manufactured for IQOS (named HEETS). Participants could choose from 3 varieties of tobacco sticks (HEETS Amber, rich tobacco; HEETS Yellow, smooth tobacco; and HEETS Turquoise, menthol-flavoured tobacco), which were available for sale on the Italian market at the time of the study. HTP study arm, 56.4% (62/110) chose HEETS Amber, 33.6% (37/110) chose HEETS Yellow, and 10.0% (11/110) chose HEETS Turquoise tobacco sticks.  Both groups: “Motivational counseling was offered throughout the study to maximize study product adherence, to favor transition away from combustible tobacco cigarettes, and to prevent relapse back to smoking.” | |
| Outcomes | 12-week study. Follow-up 24 weeks. Baseline, 1, 2, 4, 8, 12, and 24 weeks.  Biochemically verified self-reported continuous abstinence at 12 weeks from the previous visit. Continuous abstinence rate (CAR) weeks 4 to 12). Abstinence from smoking was defined as eCO-verified (< 10 ppm). 7-day point prevalence of abstinence at week 12.  Secondary outcomes include: smoking reduction from baseline, adoption rates and product acceptability, tolerability, changes in step test values and in the level of selected biomarkers of exposure in exhaled breath (i.e. eCO) and in spot urine samples  A follow-up visit at 24 weeks to review product usage and smoking behaviour under naturalistic conditions of use | |
| Study funding | NCT record: Collaborators: Philip Morris Société Anonyme  Caponnetto 2020: This research is supported by an Investigator-Initiated Study award by Philip Morris Products Société Anonyme (PMI.IIS.2016.006). | |
| Author declarations | EM, DS and RP are full-time employees of the University of Catania, Italy. PC, MC and RE are fixed-term researchers at University of Catania, Italy. MM is a fixed-term researcher at Centro per la Prevenzione e Cura del Tabagismo, University of Catania. BB is a full-time employee of ARNAS Garibaldi, Catania, Italy. AP is a full-time employee of Casa di Cura Musumeci-Gecas, Gravina di Catania, Italy. UP is a full-time employee of Ospedale “San Vincenzo” - Taormina, Italy.  In relation to his work in the area of tobacco control, RP has received lecture fees and research funding from Pfizer and GlaxoSmithKline, manufacturers of stop smoking medications. He has also received support from The Consumer Advocates for Smoke-free Alternatives (CASAA) for publication and open access costs of one paper. He has also served as a consultant for Pfizer, Global Health Alliance for treatment of tobacco dependence, ECITA (Electronic Cigarette Industry Trade Association, in the UK), Arbi Group Srl., and Health Diplomats (consulting company that delivers solutions to global health problems with special emphasis on harm minimization). Lectures fees from a number of European electronic cigarette industry and trade associations (including FIVAPE in France and FIESEL in Italy) were directly donated to vapers advocacy no-profit organizations. He is also currently involved in the following pro bono activities: scientific advisor for LIAF, Lega Italiana Anti Fumo (Italian acronym for Italian Anti Smoking League) and for The Consumer Advocates for Smoke-free Alternatives (CASAA); Chair of the European Technical Committee for standardization on “Requirements and test methods for emissions of electronic cigarettes” (CEN/TC 437; WG4).  The other authors have no conflict of interests to declare. | |
| Notes |  | |
| ***Risk of bias*** | | |
| **Bias** | **Authors' judgement** | **Support for judgement** |
| Random sequence generation (selection bias) | Low risk | NCT record: The randomization sequence will be computer-generated by using blocks size of 5, with an allocation ratio of 1:1 for each of the study products (IQOS, JustFog-EC). This was not reported in the paper. |
| Allocation concealment (selection bias) | Low risk | From Caponnetto 2020: “assignment to one of the two groups must be made on the basis of a simple list. The “dummy” randomization list will be generated by the statistician through an SAS program (Statistical Analysis System Version 9.4). The randomization list will consist of 220 numbers of 3 digits in the form nnn (where “nnn” is a sequential number from 001 to 220). In derogation from the protocol (Study Design and Study Plan section) and in order to make it impossible to identify the subject's own arm to be randomized (before the number is assigned), the size of the blocks will not be 4 but will be variable in blocks of 4 and 6. The sequence of blocks will also be randomized and blind.” |
| Blinding of participants and personnel (performance bias) All outcomes | High risk | “Obviously, product assignment could not be blinded, and strong product preference (IQOS-HTP vs JustFog-EC) could have introduced an allocation bias.” Authors aware of a strong product preference |
| Blinding of outcome assessment (detection bias) All outcomes | Low risk | Self-reported AEs. VO2 max measured by the Chester step test. eCO measured |
| Incomplete outcome data (attrition bias) All outcomes | Low risk | EC 101/110 12 weeks; 94/110 at 24 weeks |
| Selective reporting (reporting bias) | High risk | Study completed in May 2020 but 24-week outcome data not reported in publication or in the trial register record in which 12-week outcomes were reported |

Carpenter 2017

| ***Study characteristics*** | | |
| Methods | Design: randomized, parallel-assignment, open-label trial  Recruitment: recruitment from local urban community in southeastern USA, using various media outlets  Setting: community, southeastern USA  Study start date: November 2014; Study end date: May 2016 | |
| Participants | Total N: 68  N per arm: Control group: 22; ENDS group: 46 (split into 2 non-randomized groups: BluCig 16 mg: 25; BluCig 24 mg: 21)  Inclusion criteria:   - Age 18+ years - Current smoker of ≥ 5 cpd for ≥ 1 year - No recent history of cardiovascular distress, COPD, cancer (any non-dermatologic), or uncontrolled diabetes mellitus - Neither pregnant nor breastfeeding (verified) - Absence of any major current psychiatric impairment, including current alcohol/drug abuse/dependence - Current, active use of email - At least some concern for health effects of smoking (> none at all on a Likert scale) - Not used any ENDS product in the past 6 months - Never purchased an ENDS product   Exclusion criteria:   - Use of non-cigarette tobacco products (e.g. cigarillos) in the last 30 days - Current use of any smoking cessation medications - Current enrolment in a smoking cessation treatment study   Women: 59.7%; mean age: 42.2; mean cpd: 15.3; heaviness of smoking (0 to 6): 2.9  EC use: Control: 9%; ENDS 16 mg group: 4%; ENDS 24 mg group: 33%  Motivation to quit smoking in next month (0 to 10): Control: 4.0; ENDS 16 mg: 5.0; ENDS 24 mg: 4.4 | |
| Interventions | **EC: Cig-a-like**  **Intervention**: At study start, choice of tobacco or menthol flavour Blu Starter Pack EC, with 16 mg/mL nicotine. Midway through study, the manufacturer of Blu altered the product and discontinued availability of the device, replaced with BluPlusþ, with 24 mg/mL nicotine. 3-week sampling period, given up to 7 cartridges at each of 3 weekly visits. Instructions on usage "kept minimal to preserve naturalistic intent." The study team suggested that ENDS could be used "as you wish, to cut down or quit smoking, help manage smoking restrictions, or both."  **Control**: own brand of cigarettes | |
| Outcomes | Weeks 2, 3, 4, 8, 12, and 16  Carbon monoxide, NNAL  Other outcomes: cessation (< 6 months), product evaluation, EMA | |
| Study funding | "Support was provided by NIH R21 DA037407 (to M.J. Carpenter), P01 CA200512 (to K.M. Cummings, M.J. Carpenter, and M.L. Goniewicz), UL1 TR001450, and P30 CA138313. M.L. Goniewicz's laboratory is supported via P30 CA016056. B.W. Heckman is supported via K12 DA031794 and K23 DA041616. T.L. Wagener's effort is partially supported by the Oklahoma Tobacco Research Center, which is funded by the Oklahoma Tobacco Settlement Endowment Trust." | |
| Author declarations | "M.L. Goniewicz is a consultant/advisory board member for Johnson & Johnson. K.M. Cummings reports receiving a commercial research grant from and is a consultant/advisory board member for Pfizer Inc., and has provided expert witness testimony for various plaintiffs in lawsuits involving cigarette manufacturers. No potential conflicts of interest were disclosed by the other authors." | |
| Notes | New for 2020 update. Listed as ongoing study NCT02357173 in 2016 review update. Additional data provided from authors  In all, 25 participants (54%) received the Blu Starter Pack (16 mg), and 21 participants (46%) received BluPlusþ (24 mg); no switches were made within participants. Note: this is not included in our analysis of higher v lower as assignment to nicotine dose was not done at random; 24 mg and 16 mg merged in our main analysis | |
| ***Risk of bias*** | | |
| **Bias** | **Authors' judgement** | **Support for judgement** |
| Random sequence generation (selection bias) | Unclear risk | Quote: “Randomization to group was stratified by motivation to quit in the next 30 days (0–6 vs. 7–10 on a VAS scale) but proportioned 2:1 (ENDS:control) to increase precision estimates for e-cigarette uptake and usage.” |
| Allocation concealment (selection bias) | Unclear risk | Not specified |
| Blinding of participants and personnel (performance bias) All outcomes | High risk | Not blinded and included non-active control |
| Blinding of outcome assessment (detection bias) All outcomes | High risk | CO biochemically verified, but abstinence not used as outcome in this review, so rated based on adverse event reporting. Self-report, no blinding of participants |
| Incomplete outcome data (attrition bias) All outcomes | Low risk | Retention rate:  Week 4: control:19/22 (86%); ENDS 16 mg: 23/25 (92%); ENDS 24 mg: 20/21 (95%)  Week 16: control: 16/22 (73%); ENDS 16 mg: 19/25 (76%); ENDS 24 mg: 15/21 (71%) |
| Selective reporting (reporting bias) | Unclear risk | Not specified |
| Other bias | Low risk | Midway through the study, the manufacturer of Blu altered the product and discontinued availability of the device, replaced with BluPlusþ, with 24 mg/mL nicotine, again offered in both tobacco and menthol flavourings, and with improved battery duration (4-watt battery for both devices). In all, 25 participants (54%) received the Blu Starter Pack (16 mg), and 21 participants (46%) received BluPlusþ (24 mg); no switches were made within participants. The change in product (IRB approved) allowed us the unexpected opportunity to assess what impact, if any, the change in product design had on study outcomes. Note that the manufacturer, style of device, and packaging did not change, nor did our messaging to participants. The only difference was the strength of product. Thus, trial outcomes are reported across 3 groups: control versus 16 mg versus 24 mg ENDS. We have not rated this as high risk of bias as our analyses did not compare on nicotine strength and both nicotine arms were combined in our main analysis. |

Carpenter 2023

| ***Study characteristics*** | | |
| Methods | Design: RCT  Setting: USA  Recruitment: participants will be recruited nationally, but a subset (N = 120) will be recruited locally to allow for biomarker collection.  Study start date: 24 May 2018. Study end date: 8 September 2022 | |
| Participants | Total N: 638  427 = NJoy Pre-Filled Tank  211 = control  53.6% female; mean age 42.3 (SD = 11.5), 68.5% white (18.5% black; 14.1% Hispanic), 30.9% ≤ HS education, and moderately dependent (mean Heaviness of Smoking Index = 14.8; SD = 7.2)  Motivated to quit: limited interest in quitting smoking (mean motivation to quit on 0 to 10 VAS = 4.3; SD = 3.2; 24.1% making quit attempt in prior year). There is no requirement to quit smoking in this study, nor is there any requirement to use e-cigarettes.  Inclusion criteria:  Age 21+; current smoker; regular use of email OR capacity to receive SMS text and internet access; additional smoking and health criteria determined at screening; minimal history of vaping | |
| Interventions | E-cigarette: NJoy Pre-Filled Tank, 3 mL pre-filled tank, 15 mg/mL of nicotine. E-cigarette samples are inclusive of a battery and self-contained tanks of assorted flavours to last up to 4 weeks. Minimal instructions on use. Continue smoking their usual cigarettes as much or as little as they would like.  Control arm: no intervention. Participants will not receive an NJOY electronic cigarette to sample and will continue smoking their usual cigarettes as much or as little as they would like. | |
| Outcomes | Baseline, 1, 2, 3, 4, 12, 24 weeks   1. Uptake. Mean days of use over 4-week sampling period (study enrolment through end of 4-week intervention) 2. Conversion. Percentage of participants who purchase an e-cigarette (study enrolment through end of 6-month follow-up) 3. Smoking cessation. Point prevalence abstinence from conventional smoking (6-month follow-up) 4. AEs categorised as mild, moderate, or severe. The AEs most commonly reported were: cough, headaches, increased phlegm. Information collected between study enrolment and 6-month FU. 5. SAEs information collected between study enrolment and 6-month FU. 6. Study product use at 4, 12, and 24 weeks   Participants will be asked to provide smoking diary data, captured electronically, daily for 4 weeks. More substantive phone assessment will track smoking and related behaviours at baseline (day 0) and +10, +17, and +24 days (weekly during initial 3 weeks, following brief lag for delays in product mailing), and at +1, +3, and +6 months. | |
| Study funding | Funding: NCI R01 210625 | |
| Author declarations | Neither the tobacco industry nor any e-cigarette manufacturer provides support of any kind for this study. | |
| ***Risk of bias*** | | |
| **Bias** | **Authors' judgement** | **Support for judgement** |
| Random sequence generation (selection bias) | Low risk | "Randomization occurred via stratified, mixed block design, stratifying on local vs. national enrollment and on desire to quit smoking: 0–6 vs. 7–10 on a VAS scale." |
| Allocation concealment (selection bias) | Low risk | Allocation was uploaded into REDCap meaning the research team were blinded to the sequence. |
| Blinding of participants and personnel (performance bias) All outcomes | High risk | Open-label with comparator group with minimal control. Randomization did not include any masking. |
| Blinding of outcome assessment (detection bias) All outcomes | High risk | Self-reported. |
| Incomplete outcome data (attrition bias) All outcomes | Low risk | EC: 292/427 (68%)  Control: 163/211 (77%) |
| Selective reporting (reporting bias) | Low risk | All relevant prespecified outcomes provided by authors |

Cobb 2021

| ***Study characteristics*** | | |
| Methods | Design: randomized, parallel-assignment, double-blind trial  Setting: USA. Penn State Medical Center in Hershey, Pennsylvania (n = 300); Virginia Commonwealth University in Richmond, Virginia (n = 220)  Recruitment: community advertisements  Study start date: June 2015; Study end date: June 2018 | |
| Participants | Total N: 520 (though Veldheer paper only reports 263)  N per arm: 130 per arm  Inclusion criteria: age 21 to 65; smoke > 9 cigarettes per day; smoke regular filtered cigarettes or machine-rolled cigarettes with a filter; CO measurement > 9 ppm at baseline; not planning to quit in the next 6 months; interested in reducing cigarette consumption; no serious quit attempt in last month, or use of FDA-approved smoking cessation medication (varenicline, bupropion (used specifically as a quitting aid), patch, gum, lozenge, inhaler, and nasal spray)  Exclusion criteria: unstable or significant medical condition in the past 12 months (recent heart attack or some other heart conditions, stroke, severe angina including high blood pressure if systolic > 159 or diastolic > 99 observed during screening); immune system disorders, respiratory diseases (exacerbations of asthma or COPD, require oxygen, require oral prednisone), kidney (dialysis) or liver diseases (cirrhosis), or any medical disorder/medication; use of any non-cigarette nicotine delivery product (pipe, cigar, dip, chew, snus, hookah, e-cigs, strips, sticks) in the past 7 days; uncontrolled mental illness or substance abuse or inpatient treatment for these in the past 6 months; difficulty providing blood samples; no surgery requiring general anaesthesia in the past 6 weeks; use of EC for ≥5 in the past 28 days or any use in the past 7 days; use of marijuana or any illicit drug/prescription drugs for non-medical use daily/almost daily, or weekly in the past 3 months per NIDA Quick Screen; hand-rolled, roll-your-own cigarettes; allergy to propylene glycol/vegetable glycerin; pregnancy/breastfeeding  58.8% women; mean age 47; mean cpd 18; mean FTND: not specified  Motivated to quit: interested in reducing cigarette intake but not planning to quit in next 6 months  EC use at baseline: none | |
| Interventions | **EC: Cartridge**  For 24 weeks:  1) **Cigarette substitute**: QuitSmart cigarette substitute - plastic tube looks like a real cigarette, designed to provide the same draw resistance as a smoker's usual cigarette. No drug delivery. 2 cigarette substitutes and a product manual are provided to participants following randomization and replacement products are provided throughout the intervention period (24 weeks). At baseline, associated user manual, research staff explain how to use product. Reduction goal to 50% at weeks 0 and 1, 75% at weeks 2 and 4, continue reducing onwards from there  2) **EC with no nicotine**: EGO e-cigarette. Cartomizers containing 0 mg/mL nicotine provided throughout the intervention period (24 weeks). Associated user manual, research staff explain how to use product.  3) As (2) but 8 mg/mL nicotine  4) As (2) but 36 mg/mL nicotine | |
| Outcomes | 0, 1, 2, 4, 8, 12, 16, 20, 24, 28, and 36 weeks  Provision of the condition-specific product lasted for 24 weeks (intervention period). There was a 12-week follow-up period after the intervention period for each condition (36 weeks).  NNAL collected at baseline 4, 12, and 24 weeks  Cessation: (a) intent-to-treat, self-reported 7-day point prevalence cigarette abstinence (PPA), biochemically confirmed by exhaled CO < 10ppm (7-day PPA) for each visit up to 24 weeks after randomization (last visit of randomized phase of the trial), with those not attending visits counted as smoking. Additional outcomes included (b) self-reported 28 or more days of cigarette abstinence at week 24 (biochemically validated by exhaled CO < 10 ppm at weeks 20 and 24), (c) the number (%) of participants in each group who reported at least one full day without smoking a cigarette (no biochemical verification), from week 1 to week 24, and (d) the total number of days on which participants self-reported being abstinent from cigarettes from week 1 to week 24.   - “tobacco-related toxicant exposure to the potent lung carcinogen NNK, as indexed by the sum of its urinary metabolite 4-(methylnitrosamino)- 1-(3-pyridyl)-1-butanol (NNAL) and its glucuronides (total NNAL; pg/mg creatinine) collected at randomisation and at 4, 12, and 24 weeks.” - Urine cotinine (ng/mg creatinine) at 4, 12, and 24 weeks - Glutathione and 8-Isoprostanes - Exhaled CO was measured at each in-person visit - Pulmonary function tests were done at randomization, 4, 12, 24, and 36 weeks - Self-reported number of cigarettes smoked per day and daily study product use were assessed at each in-person visit using a 7-day timeline follow-back procedure supplemented with paper diaries completed daily - Adverse events and serious AEs - Blood pressure, heart rate   Other outcomes measured   - Drug/alcohol measures: Alcohol AUDIT-C; NIDA Quick Screen - Cigarette measures: MNWS; confidence to quit, Stage of Change, Environmental smoke, Smoking urges, 7-day TLFB and current tobacco use - Cigarette dependence - Study product dependence and measures - Psych measures: Kessler 6; perceived stress; CES-D - Health measures: Interheart, Clinical COPD Questionnaire - Biomeasures: waist/hip ratio, weight - Blood samples: complete metabolic panel, haematology panel, lipid panel, c-reactive protein | |
| Study funding | This research was supported by grants P50DA036105 and U54DA036105 from the National Institute on Drug Abuse of the National Institutes of Health and the Center for Tobacco Products of the US Food and Drug Administration. Data collection was supported by UL1TR002649 at Virginia Commonwealth University and by UL1TR002014 at Penn State University from the National Center for Advancing Translational Sciences of the National Institutes of Health. The content of this manuscript is solely the responsibility of the authors and does not necessarily represent the official views of the National Institutes of Health or the US Food and Drug Administration. | |
| Author declarations | COC reports grants from National Institute on Drug Abuse and US Food and Drug Administration, during the conduct of the study. JF reports grants from National Institute on Drug Abuse and US Food and Drug Administration, during the conduct of the study, and grants, personal fees, and non-financial support from Pfizer, outside of the submitted work. AAL reports grants from National Institute on Drug Abuse and US Food and Drug Administration, during the conduct of the study. JMY reports grants from National Institute on Drug Abuse and US Food and Drug Administration, during the conduct of the study. LK reports grants from National Institute on Drug Abuse and US Food and Drug Administration, during the conduct of the study. SV reports grants from National Institute on Drug Abuse and US Food and Drug Administration, during the conduct of the study. CB has previously undertaken trials of electronic cigarettes for smoking cessation (with electronic cigarettes purchased from an online retailer [NZVAPOR], electronic cigarette liquid for one trial purchased from Nicopharm, Australia, and nicotine patches supplied by the New Zealand Government via their contract with Novartis [Sydney, Australia]). Neither NZVAPOR nor Nicopharm have links with the tobacco industry. None of these parties had any role in the design, conduct, analysis, or interpretation of the trial findings, or writing of this publication. TE reports grants from National Institute on Drug Abuse and US Food and Drug Administration, during the conduct of the study, and is a paid consultant in litigation against the tobacco industry and also the electronic cigarette industry and is named on one patent for a device that measures the puffing behaviour of electronic cigarette users and on another patent for a smartphone application that determines electronic cigarette device and liquid characteristics. M-SY reports grants from the National Institute on Drug Abuse and the US Food and Drug Administration, during the conduct of the study. | |
| Notes | Study listed as ongoing study Lopez 2016 in the 2016 review update and as Veldheer 2019 in 2020 and April 2021 updates.  Cessation data from Foulds, which is pre-print only | |
| ***Risk of bias*** | | |
| **Bias** | **Authors' judgement** | **Support for judgement** |
| Random sequence generation (selection bias) | Low risk | "The study statistician (M-SY) prepared site-specific randomisation lists using the sample function in R version 3.2.0 (blocks of eight). These lists were uploaded onto a study-specific website that interfaced with the data collection and management system (REDCap)." |
| Allocation concealment (selection bias) | Low risk | “Once a participant has been confirmed eligible for randomization, a computer procedure will assign the participant to the next condition on the list automatically.” "Only unmasked researchers at each site with no participant contact accessed their list to prepare cartomisers for dispensing." |
| Blinding of participants and personnel (performance bias) All outcomes | Low risk | Not blinded for non-EC arms but, given similar level of support/product, performance bias judged unlikely |
| Blinding of outcome assessment (detection bias) All outcomes | Low risk | Not blinded for non-EC arms but, given similar level of support/product, differential misreport judged unlikely |
| Incomplete outcome data (attrition bias) All outcomes | Low risk | "188 (36%) of 520 participants were lost to follow-up by week 24; attrition did not differ by group (39 [30%] of 130 in the cigarette substitute group, 56 [43%] of 130 in the ENDS with 0 mg/mL nicotine group, 49 [38%] of 130 in the ENDS with 8 mg/mL nicotine group, and 44 [34%] of 130 in the ENDS with 36 mg/mL nicotine group; P = 0·15)." |
| Selective reporting (reporting bias) | Low risk | All specified outcomes available or being written up |

Coffey 2020

| ***Study characteristics*** | | |
| Methods | Design: 1-armed intervention study  All interested offered EC intervention. Pilot to encourage people wanting to stop using CCs to swap from CCs to ECs, conducted in 2018. The pilot was designed to enable current smokers to obtain a free e-cigarette, charger, nicotine liquid, and support.  Recruitment: E-cigarette vouchers were provided through websites and also advertised and distributed by social housing providers and other community organizations, including pharmacies and stop smoking services.  Setting: socially deprived area of the NW. Greater Manchester, NW England, UK  Inclusion criteria: person using combustible cigarettes and wanting to quit  Study start date: January 2018 for 3 months  Study end date: March/April 2018 | |
| Participants | Total N: 1022 (single arm)  Mean age 44.7 years  Mean CPD: 19.14  Motivated to quit: yes | |
| Interventions | All interested offered free EC intervention: 3 different ECs, different colours, 1 with longer battery life. Charger plug  Two different strengths (1.0% and 1.6%) and 4 different flavours (tobacco, rolling tobacco, menthol, and mixed fruits) provided. 4 weeks' supply, distributed in 2 batches, 5 bottles at baseline and 5 bottles at week 2  E-cigarettes were purchased from the only Independent British Vape Trading Association (IBVTA) registered e-cigarette provider in the area (see https://www.ibvta.org.uk/about-us).  Individuals obtained their e-cigarette from a community stop-smoking service or a pharmacy, which ensured that they obtained advice and guidance around stopping smoking and using the e-cigarette, in addition to the device itself. People were required to return at 2 weeks in order to get their additional liquid, which helped to ensure that contact with the service could be maintained. A £10 high street shopping voucher was also offered as an alternative incentive to reduce bias in the population that returned for follow-up. | |
| Outcomes | Baseline, 4 weeks  CO ppm for all participants at 2 and 4 weeks follow-up  Continued EC use at 4-week follow-up | |
| Study funding | This work was commissioned by Salford City Council and carried out independently by the University of Salford. While Salford City Council and Great Manchester Health and Social Care Partnership were given the opportunity to input into the final report and the drafting of this paper, their input was limited to the context and background of the pilot, rather than the analysis or interpretation of the findings.  This work was supported by funding from the Greater Manchester Health and Social Care Partnership. | |
| Author declarations | The author(s) disclosed receipt of the following financial support for the research, authorship, and/or publication of this article: This work was supported by funding by Greater Manchester Health and Social Care Partnership. | |
| ***Risk of bias*** | | |
| **Bias** | **Authors' judgement** | **Support for judgement** |
| Random sequence generation (selection bias) | High risk | Not randomized |
| Allocation concealment (selection bias) | High risk | No allocation concealment, single arm  Uncontrolled study |
| Incomplete outcome data (attrition bias) All outcomes | Low risk | 614/1022 (60%) overall LOW RISK  Pharmacy: 167/362 (46.2% HIGH RISK)  Community: 447/660 (67.85 LOW RISK)  CO reading at 4 weeks 567/1022 = 55.5% |
| Selective reporting (reporting bias) | Unclear risk | No protocol/clinical record. Analysis of secondary data |

Czoli 2019

| ***Study characteristics*** | | |
| Methods | Design: non-blinded, within-participant cross-over  Recruitment: advertisements placed in newspapers, online, and in local vape shops, and received CAD 295 for participating in the study  Setting: Kitchener−Waterloo and Toronto, Ontario, Canada  Study start date: September 2015. Study end date: NR | |
| Participants | Total N: 48  29.2% female; mean age 35.9 (SD 11.7); mean cpd NR; dual EC users at baseline; not motivated to quit  Inclusion criteria: > 18+ years; dual users of tobacco cigarettes and EC  Exclusion criteria: serious intentions to quit smoking in the next 6 months; tobacco products, NRT, any smoking cessation medications, participation counselling programmes for smoking cessation in the past 7 days; serious cardiac health issues; heart attack or stroke within the last 3 months; cancer within the last year; asthma, chronic obstructive pulmonary disease, a seizure disorder, or any life-threatening medical conditions with a prognosis of ≤ a year; history of psychosis, schizophrenia, bipolar disorder, or suicidal thoughts | |
| Interventions | **EC: own choice (mainly tank)**  3 consecutive 7-day periods in which the use of tobacco cigarettes and e-cigarettes was experimentally manipulated  4 study conditions: dual use (e-cigarette and tobacco cigarette); tobacco cigarette; E-cigarette; no product use  Virtually all dual users reported using tank systems (92%) and e-cigarettes with nicotine (94%).  To control for order effects, participants were randomly assigned to 1 of 2 condition orders, A or B.  Following the baseline condition of dual use:  Group A participants switched to e-cigarette use, then to tobacco cigarette use, and finally to no product use.  Group B participants switched to tobacco cigarette use, then to e-cigarette use, and finally to no product use. | |
| Outcomes | Baseline (visit 1) and after each of the 7-day periods (visit 2 (week 1), visit 3 (week 2), visit 4 (week 3))  Carbon monoxide  Urinary concentration of cotinine  Urinary concentrations of 1-hydroxypyrene (1-HOP) and 4-(methylnitrosamino)-1-(3-pyridyl)-1-butanol (NNAL) | |
| Study funding | This research was supported by an Ontario Ministry of Health and LongTerm Care Health System Research Fund grant (#06697 awarded to DH). Additional support was provided by the Canadian Institutes of Health Research (CIHR), the Vanier Canada Graduate Scholarship (CDC), a CIHR and Public Health Agency of Canada, Applied Public Health Chair (DH), and an Ontario Institute for Cancer Research Investigator Award (GTF). | |
| Author declarations | MLG reports grants from and served as an advisory board member to pharmaceutical companies that manufacture smoking cessation drugs. DH has provided paid expert testimony in tobacco litigation on behalf of governments and class-action plaintiffs on issues related to tobacco product science and regulation. The other authors have no competing interests to declare. | |
| ***Risk of bias*** | | |
| **Bias** | **Authors' judgement** | **Support for judgement** |
| Random sequence generation (selection bias) | Unclear risk | No details of randomization method given |
| Allocation concealment (selection bias) | High risk | No blinding |
| Blinding of participants and personnel (performance bias) All outcomes | High risk | No blinding |
| Blinding of outcome assessment (detection bias) All outcomes | Unclear risk | Not reported |
| Incomplete outcome data (attrition bias) All outcomes | Low risk | All followed up |
| Selective reporting (reporting bias) | Low risk | No evidence of selective reporting |

Dawkins 2020

| ***Study characteristics*** | | |
| Methods | Design: prospective cohort 4-centre pragmatic cluster feasibility trial  Recruitment: at homeless centres  Setting: 4 homeless centres in the UK  Study start date: 1 October 2018; Study end date: 31 March 2020 | |
| Participants | Total N: 80  N per arm: EC 48; UC 32  Inclusion criteria: adults (≥ 18 years) who smoke accessing homeless support services on a regular basis and also known to staff; daily smokers; smoking status was also biochemically verified by exhaled CO breath  Exclusion criteria: non-smokers, or using another smoking cessation aid; pregnancy, or unable to consent, e.g. currently intoxicated or unable to speak English; not well known to centre staff  Inclusion based on specific population characteristics: people accessing homeless centres  35% women; mean age 42.7; mean cpd 20; mean FTND: FTCD 5.51  Motivated to quit: “varied considerably; large majority expressed a desire to quit smoking in the near future”  EC use at baseline: not specified | |
| Interventions | **EC: Refillable**  **Usual care**: written information on quitting smoking (adapted from NHS Choices); signposting to the local stop-smoking service (SSS) by centre staff  **Intervention**: as usual care, plus refillable EC provided once with e-liquid provided 1 x wk for 4 weeks, Aspire PockeX (tank style), choice of 3 flavours (fruit, menthol, tobacco) and 2 nicotine strengths (12 mg/mL or 18 mg/mL). Written info for EC use and support from centre staff, who met once a week to provide e-liquid and troubleshoot EC use | |
| Outcomes | Weeks: 4, 12, 24; clinic visits and self-report  Cessation: CO-validated sustained at 24 weeks  Adverse events and biomarkers: self-reported negative effects in EC arm only – each participant asked to rate on a scale so we could not meta-analyse; exhaled CO; unintended consequences  Other outcomes measured:  Qualitative process evaluation; costs; self-reported positive and negative affects; recruitment rates; retention; EC/other tobacco/nicotine product use at study end; HRQoL; healthcare service utilization; other drug use/dependence; unintended consequences | |
| Study funding | This study is funded by the National Institute for Health Research Public Health (project reference: 17/44/29). | |
| Author declarations | SC, AF, JL, CB, AT, DR, IU, LB, SP have no competing interests. PH has received a research grant from and provided consultancy to Pfizer. LD has provided consultancy for the pharmaceutical industry relating to the development of smoking cessation products. | |
| Notes | New for 2021 update. Authors provided information prior to peer review. | |
| ***Risk of bias*** | | |
| **Bias** | **Authors' judgement** | **Support for judgement** |
| Random sequence generation (selection bias) | High risk | Intention was to randomize but were unable to due to practical constraints  Quote: “Thus the actual allocation of centres to each arm was a pragmatic decision based on centre readiness and staff/researcher availability though we balanced potential confounders and differences in environment by ensuring each cluster (EC and UC) contained one day centre and one residential unit.” |
| Allocation concealment (selection bias) | Unclear risk | Quote: “Participants joined after cluster randomisation… Allocation was concealed to participants until after the baseline assessment.”  Comment: But unclear if allocation was concealed for those recruiting, and allocation would have been known to new participants |
| Blinding of participants and personnel (performance bias) All outcomes | High risk | Not blinded and different levels of support between arms, so performance bias cannot be ruled out |
| Blinding of outcome assessment (detection bias) All outcomes | Low risk | Cessation (primary outcome) biochemically validated |
| Incomplete outcome data (attrition bias) All outcomes | High risk | 13/48 (27.1%) lost to follow-up in the intervention arm and 20/32 (62.5%) lost to follow-up in the control arm at 24 weeks |
| Selective reporting (reporting bias) | Low risk | All anticipated outcomes reported |

Edmiston 2022\*

| ***Study characteristics*** | | |
| Methods | Design: RCT  Recruitment: participants were recruited to 10 clinical sites via site databases and using IRB-approved radio and print ads.  Setting: USA  Study start date: 17 January 2017. Study end date: 6 November 2018 | |
| Participants | Total N: 450  EC Test group 1: classic (tobacco) = 150  EC Test group 2: menthol = 150  Control = 150  Inclusion criteria: smoked ≥ 10 years, smoked an average of ≥ 10 manufactured cigarettes pd for 12 months. Willing and able to replace their cigarettes for 12 weeks with the assigned test e-Vapor product. Age 30 to 65 years.  Exclusion criteria: health condition that would jeopardize the safety of the participant or impact the validity of the study results; currently taking medication for depression, asthma or diabetes  For a full list of inclusion and exclusion criteria, see publication.  Female 51%; mean age 44.4 (SD 9.73); mean CPD 17.6 (SD 4.95) | |
| Interventions | EC: **cartridge**  Arm 1 Experimental: Test 1 EC classic (tobacco)  Exclusive ad libitum use of test product MarkTen Bold Classic (test product 1) 4.0% nicotine by weight without use of any other type of tobacco/nicotine-containing product, for the entire duration of study participation. This replaced test e-Vapor Product NuMark LLC, MarkTen® XL Bold CLASSIC\* (as no longer sold).  Arm 2 Experimental: Test 2 EC menthol  Exclusive ad libitum use of test products MarkTen Bold Menthol (test product 2) 4.0% nicotine by weight  Arm 1 and 2 participants were to completely replace their cigarettes with the test EVPs. Participants had 7 days to switch to EVPs prior to clinic visits.  Arm 3 No Intervention: control  Assigned to continue smoking their own brand cigarettes under ad libitum conditions | |
| Outcomes | Baseline, weeks 1, 3, 6, 9, and 12  Baseline weeks 1, 6 and 12 (blood and urine). Weeks 1, 3, 6, 9, and 12 (eCO)  Lung function was assessed at screening, baseline, at week 12 of study 1, (and at weeks 18 and 24 of study 2): FEV1, per cent of predicted FEV1, FVC, percent of predicted FVC, FEV1/FVC, and percent of predicted FEV1/FVC, forced expiratory flow at 25% to 75% (FEF).  NNAL urinary total 4-(methylnitrosamino)-1-(3-pyridyl)-1-butanol and its glucuronides (ng/g creatinine), WBC, COHb, HDLC  Adverse events (AEs) and medications were recorded and monitored throughout the study. | |
| Study funding | This study was funded by Altria Client Services LLC.  (Altria is the parent company of Philip Morris USA (producer of Marlboro cigarettes), John Middleton, Inc., U.S. Smokeless Tobacco Company, Inc., and Philip Morris Capital Corporation.) | |
| Author declarations | All authors were employees of Altria Client Services LLC at the time of the study. | |
| Notes | Paper reported 2 studies; only 1 study was eligible.  New to 2022 update | |
| ***Risk of bias*** | | |
| **Bias** | **Authors' judgement** | **Support for judgement** |
| Random sequence generation (selection bias) | Unclear risk | Randomised – no further information in paper or supplementary materials |
| Allocation concealment (selection bias) | Unclear risk | No detail provided |
| Blinding of participants and personnel (performance bias) All outcomes | High risk | Open-label |
| Blinding of outcome assessment (detection bias) All outcomes | Unclear risk | No detail provided |
| Incomplete outcome data (attrition bias) All outcomes | Low risk | < 50% attrition |
| Selective reporting (reporting bias) | Low risk | Outcomes reported |

Edwards 2023

| ***Study characteristics*** | | |
| Methods | Design: pragmatic, uncontrolled, mixed-methods trial  Recruitment: targeted settings for people with HIV  Setting: community, Brisbane, Australia  Study start date: 21 February 2017; study end date: 26 October 2017 | |
| Participants | Total N: 30  Inclusion criteria: diagnosis of HIV; ≥ 18 years; ≥ 5 cpd at the time of enrolment into the trial; smoking ≥ 12 months; willing to attempt to quit tobacco smoking after study enrolment  Exclusion criteria: participating in a smoking-cessation programme; pregnancy or breastfeeding; experiencing chest pain, or another cardiovascular event or procedure in the last month; being treated with oxygen therapy  Inclusion based on specific population characteristics: people living with HIV  29 participants identified as male, and 1 participant did not identify as male or female; mean age: 42; mean cpd: 18  EC use at baseline: 46.7% (n = 14) never tried; 50% (n = 15) tried, never used for an extended period; 3.3% (n = 1) used on a regular (weekly) basis  Willing to attempt to quit | |
| Interventions | **EC: Refillable**  Single-arm study. Print materials to help quit smoking. Provided booklet with instructions on how to use, store and handle EC; copies of device user manuals. Given Innokin Endura T18® vaporiser kit, Innokin Endura T22® vaporiser kit, 4 spare coils, 1 wall charger, 10 x 10 mL bottles of Nicophar® 12 mg nicotine e-liquid. Supplies to last 12 weeks | |
| Outcomes | Weeks 1, 4, 8, 12, 24; self-report and semi-structured interviews  Cessation: 7 days point prevalence at weeks 4, 8, 12, and 24. Continuous abstinence at weeks 12 and 24. No biochemical validation  Adverse events  Other outcomes: acceptability and use of trial products; number of quit attempts | |
| Study funding | "This work was supported by the HIV Foundation Queensland. The funder will play no role in the analysis and interpretation of results. All trial products were purchased and the suppliers have no involvement in the conduct of the trial or the interpretation or reporting of the results." | |
| Author declarations | "No other authors declare conflicts of interest. Mark Boyd has received research grant funding (paid to the institution) from AbbVie, Gilead and Merck and received honoraria for participation in HIV Advisory Boards and for the preparation and delivery of educational materials from AbbVie, Boehringer-Ingelheim, Bristol Myers Squibb, Gilead, Janssen-Cilag, Merck and ViiV Healthcare." | |
| Notes | Additional data provided from authors. New for 2020 update, relabelled from Bell 2017 to Edwards 2023 at 2023 update. | |
| ***Risk of bias*** | | |
| **Bias** | **Authors' judgement** | **Support for judgement** |
| Random sequence generation (selection bias) | High risk | Uncontrolled study |
| Allocation concealment (selection bias) | High risk | Uncontrolled study |
| Incomplete outcome data (attrition bias) All outcomes | Low risk | Quote: “At week 24, 26 of the 30 participants who enrolled in the study were followed up.” (confirmed by authors) |
| Selective reporting (reporting bias) | Low risk | Study not published at time of data extraction, but study protocol published |

Eisenberg 2020

| ***Study characteristics*** | | |
| Methods | Design: 3-arm RCT  Recruitment: community  Setting: Canada  Study start date: November 2016. Study end date: September 2019 | |
| Participants | Total N: 376; nicotine e-cigarettes = 128; non-nicotine e-cigarettes = 127; counselling (control) = 121  47% female; mean age 52.66; mean cpd 21; mean FTND 6 (SD 2)  Motivated to quit: yes  Inclusion criteria: active smoker, ≥ 10 CPD for past year; ≥ 18 years; motivated to quit according to the Motivation To Stop Scale (MTSS) (level 5 or higher); provide informed consent in English or French; available for follow-up (1 year)  Exclusion criteria: medical condition with a prognosis < 1 year; current or recent cancer (≤ 1 yr in remission); pregnancy/breastfeeding; current/ recent use of any pharmacotherapy or behavioural therapy for smoking cessation (e.g. NRT, bupropion, varenicline, or counselling); any EC use (nicotine/non-nicotine) in past 60 days, or ever use of any EC ≥ 7 days consecutively; psychosis, schizophrenia, or bipolar disorder; ≤ 1 month following myocardial infarction, life-threatening arrhythmia, severe or worsening angina pectoris, or cerebral vascular accident; illegal drug use past yr (excluding marijuana); planned use of tobacco products other than conventional cigarettes (e.g. cigarillos, cigars, snuff, shisha, etc.) or marijuana during the study period | |
| Interventions | **EC: Cig-a-like**  **Nicotine e-cigarettes plus counselling:**  12 weeks of e-cigarettes. Rechargeable base with prefilled, disposable, tobacco-flavoured liquid cartridges (15or 0 mg nicotine/mL), which were produced specifically for use in clinical studies (purchased from NJOY Inc, Scottsdale, Arizona). 21 cartridges at baseline with additional cartridges supplied as needed. Nicotine and non-nicotine e-cigarettes were identical in appearance. Instructed to be used as desired. No schedule for e-cigarette tapering, but participants were aware that they would return their e-cigarettes after 12 weeks.  Participants received individual smoking cessation and relapse prevention counselling (minimum 30 minutes at baseline, 10 minutes during telephone follow-ups, and 15 to 20 minutes at clinic visits). Individualized quit plans.  **Non-nicotine e-cigarettes plus counselling:**  As above with 0 mg nicotine/mL in liquid cartridge  **Counselling (control):**  Participants received individual smoking cessation and relapse prevention counselling (minimum 30 minutes at baseline, 10 minutes during telephone follow-up, and 15 to 20 minutes at clinic visits). Individualized quit plans. | |
| Outcomes | Follow-up was conducted by telephone at weeks 1, 2, 8, and 18, and at clinic visits at weeks 4, 12, 24, and 52.  Self-reported smoking (7-day recall), adherence, and adverse events (AEs) were assessed during follow-up contacts  Biochemically-validated 7-day point prevalence smoking abstinence at 4, 12, and 24 weeks, defined as self-reported abstinence in the past 7 days with exhaled carbon monoxide < 11 ppm  At baseline: cpd; FTND; Glover-Nilsson Smoking Behavioral Questionnaire (to assess behavioural dependence on smoking); and Beck Depression Inventory II (BDI-II; to assess depressive symptoms) | |
| Study funding | This trial was funded by the Canadian Institutes of Health Research (CIHR; funding reference No. 133727 and 155969). Both nicotine e-cigarettes and non-nicotine e-cigarettes were purchased from NJOY Inc (Scottsdale, Arizona) | |
| Author declarations | Dr Eisenberg reported receiving educational grants from Pfizer Inc for providing continuing medical education in cardiology. Dr Wilderman reported receiving financial compensation from Pfizer Inc for his involvement in a smoking cessation study using varenicline. Dr Filion reported receiving salary support from the Fonds de Recherche du Quebec, a William Dawson Scholar award from McGill University, and personal fees from Institut National D’excellence en Santé et Services Sociaux. No other disclosures were reported. | |
| Notes | New cessation and adverse event data for 2021 update. Previously listed as NCT02417467 (included with SAE data only) | |
| ***Risk of bias*** | | |
| **Bias** | **Authors' judgement** | **Support for judgement** |
| Random sequence generation (selection bias) | Low risk | Eligible participants were randomized via an online central randomization system. The system used a computer-generated randomization list containing permuted blocks of 6 and 9, stratified by centre. |
| Allocation concealment (selection bias) | Low risk | Participants, investigators, and study personnel were blinded to nicotine content in the e-cigarette groups. Quote: "Eligible participants were randomized via an online central randomization system. The system used a computer-generated randomization list containing permuted blocks of 6 and 9, stratified by center." |
| Blinding of participants and personnel (performance bias) All outcomes | Low risk | Participants, investigators, and study personnel were blinded to nicotine content in the e-cigarette groups. |
| Blinding of outcome assessment (detection bias) All outcomes | Low risk | Participants, investigators, and study personnel were blinded to nicotine content in the e-cigarette groups. |
| Incomplete outcome data (attrition bias) All outcomes | Low risk | Low numbers lost to follow-up, treated as ITT |
| Selective reporting (reporting bias) | Low risk | Due to a prolonged and unforeseen delay in e-cigarette manufacturing, enrolment was paused on 27 September 2019, and then terminated on 14 November 2019. Given reduced power, the timing of the primary endpoint was changed from 52 weeks to 12 weeks on 04 December 2019. No 12-month follow-up but this was for manufacturing reasons and was reported |

Eisenhofer 2015

| ***Study characteristics*** | | |
| Methods | Design: RCT  Setting: USA | |
| Participants | 11  EC = 4; NRT = 7  Veterans who meet DSM criteria for tobacco use disorder  18% female; mean age 52.6; mean cpd 26.4; mean FTND 7.5; 64% African American | |
| Interventions | EC type: cartridge  Arm 1: Electronic cigarettes 16 mg cartridge. Arm 2: NRT  Participants attended thrice-weekly visits during the first 2 weeks (week 1-“baseline” with participants smoking ad libitum) and attended 5 visits during the third week (week 3-“efficacy” with participants smoking as little as possible while using NRT or E-cigs) | |
| Outcomes | Baseline, 3 visits during week 1 and week 2, 5 visits during week 3.  Self-reports of cigarettes smoked in last 24 hours, confirmed by breath CO levels and salivary cotinine | |
| Study funding | This work was conducted at and supported by resources at the MEDVAMC, including a MEDVAMC Research Enhancement Seed Grant. | |
| Author declarations | NS | |
| Notes | Study information extracted from conference abstract only | |
| ***Risk of bias*** | | |
| **Bias** | **Authors' judgement** | **Support for judgement** |
| Random sequence generation (selection bias) | Unclear risk | Quote: “Veterans were randomized to either NRT (16 mg patch; N = 7) or E-cigs (16 mgcartridge; N = 4).” No further information provided |
| Allocation concealment (selection bias) | Unclear risk | No information provided |
| Blinding of participants and personnel (performance bias) All outcomes | Low risk | No blinding, but active interventions provided to both arms |
| Blinding of outcome assessment (detection bias) All outcomes | Low risk | Quote: "CO levels and salivary cotinine were recorded during each visit.' 'Self-reports of cigarettes smoked in last 24 h, and this was confirmed by significant reductions of breath CO levels by NRT (t = 3.7, P = 0.01) and E-cigs (t = 3.9, P = .03)." |
| Incomplete outcome data (attrition bias) All outcomes | Unclear risk | No information provided |
| Selective reporting (reporting bias) | Unclear risk | No protocol or clinical trial record available to determine whether all prespecified outcomes were reported |

Elling 2023

| ***Study characteristics*** | | |
| Methods | Design: RCT  Setting: Netherlands, online  Recruitment  Study start date: recruited March 2020 to July 2020 | |
| Participants | N = 331  Intervention = 157; control = 174  % female: 59.8%; mean age: 49.0 (SD 13.2); mean CPD: 12.6 (cigarettes per week 83.3/7); mean FTND: 3.8 (2.6) Intervention 4.1 (2.6), Control 3.5 (2.5)  Inclusion criteria were that participants are at least 18 years old, have sufficient command of the Dutch language, have necessary internet literacy to use the intervention, have smoked tobacco in the past 7 days, and are motivated to quit tobacco smoking within 5 years.  EC use at baseline: overall 33/331 (10%). Intervention 17 (10.8); control 16 (9.2)  Motivated to quit: yes. Within next 5 years | |
| Interventions | Digital computer-tailored smoking cessation intervention  Arm 1: intervention condition  Participants in the intervention condition will receive tailored information on e-cigarettes based on 5 items. The computer-tailored intervention will be based on the I-Change model.  Arm 2: control condition  The control condition will not receive tailored information about e-cigarettes. | |
| Outcomes | Baseline, 6 months  Number of tobacco cigarettes smoked in the past 7 days; average number of tobacco cigarettes smoked per day; 7-day point prevalence tobacco abstinence; 7-day point prevalence e-cigarette abstinence  Smoking cessation methods; determinants of decision-making; process evaluation | |
| Study funding | National Institute for Public Health and the Environment (Rijksinstituut voor Volksgezondheid en Milieu, RIVM) | |
| Author declarations | No conflicts of interest declared | |
| ***Risk of bias*** | | |
| **Bias** | **Authors' judgement** | **Support for judgement** |
| Random sequence generation (selection bias) | Unclear risk | Stated randomized, but the process was not described in the paper, protocol, or record |
| Allocation concealment (selection bias) | Unclear risk | Not reported |
| Blinding of participants and personnel (performance bias) All outcomes | Unclear risk | Online, so participants would be unlikely to be aware of other participants |
| Blinding of outcome assessment (detection bias) All outcomes | High risk | Self-reported abstinence |
| Incomplete outcome data (attrition bias) All outcomes | Low risk | 139/157 intervention  138/174 control |
| Selective reporting (reporting bias) | Low risk | Stated investigating the hypothesis of whether intervention arm smokeless at 6 months |

Ely 2013

| ***Study characteristics*** | | |
| Methods | Design: prospective cohort  Recruitment: letter sent to family practice patients who currently smoked  Setting: single family practice, Colorado, USA  Study start date: 14 April 2013; Study end date: not specified | |
| Participants | Letters sent to 640 patients, 48 chose to participate and 44 completed the programme, 4 were lost to follow-up  Inclusion criteria:   - Want to quit or switch from tobacco cigarettes to ECs   Exclusion criteria:   - None reported   Of the 44 participants, 66% women, all non-Hispanic/white, aged 20 to 75 (30% were age 51 to 60), 57% had a high school education or less  Motivated to quit: want to quit or switch from tobacco cigarettes to ECs  E-cigarette use at baseline: not specified | |
| Interventions | **EC: Cig-a-like**  The 6-month smoking cessation programme was based on the '5 A's' model and transtheoretical model. Options for treatment were discussed with each participant at the start of the programme. All used an EC, with 16 using bupropion and 2 using varenicline as well.  Participants were provided with written information on “blu cig” and “smoke tip” ECs, about cost, availability, nicotine dosage options. | |
| Outcomes | Phone follow-ups at 2 weeks, 1 month, 3 months, and 6 months  At completion of programme (using ITT)  Abstinence from smoking and EC use  Abstinence from smoking but not EC use  ≥ 50% reduction of baseline cigarette consumption (still using ECs) | |
| Study funding | Not specified | |
| Author declarations | Not specified | |
| ***Risk of bias*** | | |
| **Bias** | **Authors' judgement** | **Support for judgement** |
| Random sequence generation (selection bias) | High risk | Prospective cohort |
| Allocation concealment (selection bias) | High risk | Not randomized |
| Incomplete outcome data (attrition bias) All outcomes | Low risk | 4/48 lost to follow-up |
| Selective reporting (reporting bias) | Unclear risk | Unable to determine prespecified outcomes |
| Other bias | Unclear risk | No definition of abstinence provided  Not clear if 'completed programme' was at 6 months |

Felicione 2019

| ***Study characteristics*** | | |
| Methods | Design: double-blind RCT  Recruitment: people who smoke were recruited from an outpatient opioid-maintenance clinic in West Virginia, USA  Setting: outpatient opioid-maintenance clinic in West Virginia, USA  Study start date/Study end date: not reported | |
| Participants | Total N: 25; N per arm: placebo (non-nicotine): 11; active (18 mg/mL nicotine): 14  Inclusion criteria:   - ≥ 18 years of age - Report smoking ≥ 10 cpd for ≥ 1 year - Report a current interest in quitting smoking   Exclusion criteria:   - Reported regular use of any nicotine/tobacco product other than cigarettes, including EC, or were already engaged in attempts to quit smoking   Inclusion based on specific population characteristics: people who smoke who were currently receiving a buprenorphine/naloxone combination in sublingual form, and had maintained sobriety from opioids and all other illicit substances for at least 90 consecutive days as verified via urinalysis  73.0% women; mean age 32.5; mean cpd 22; mean FTND 5.8  Motivated to quit: Quit ladder score (range 1 to 10): 5.6 average | |
| Interventions | **EC: Refillable**  Compared **nicotine** (18 mg/mL) to **non-nicotine EC**  Second-generation EC consisted of the eGo-T battery (900mAh, 3.3 V constant output) (Joyetech; Irvine, CA) and the Kanger mini Protank-II, 1.5 ml Pyrex glass tank with a drip tip and atomizer head coils (KangerTech; China), choice between tobacco (n = 15) and menthol (n = 10) flavoured liquid (2-week supply). Participants were then trained in EC device operation, including assembly, liquid filling, manual battery operation, and cleaning/storage. Practised puffing on EC in the presence of a team member, and asked questions if needed. Participants instructed to use their ECIG ad libitum every day for 2 weeks. | |
| Outcomes | Baseline (day 1), 14 days, 28 days for clinic measures. Data also collected via text messages over 2-week intervention period  Withdrawal/side effects: every evening during the 2-week intervention period, participants rated a variety of effects possibly experienced as a result of nicotine/tobacco withdrawal and/or use of the ECIG: nausea, dizziness, throat irritation/soreness, cough, dry mouth, headache, shortness of breath, irritability/frustration/anger, craving/urge to smoke, and other. Each item was rated on a continuous scale that ranged from 0 (not at all) to 100 (extremely).  Expired air CO  Other outcomes: self-reported cigarette and EC use; readiness to quit at day 1, 14, and 28 | |
| Study funding | Not reported | |
| Author declarations | Not reported | |
| Notes | New for 2020 update | |
| ***Risk of bias*** | | |
| **Bias** | **Authors' judgement** | **Support for judgement** |
| Random sequence generation (selection bias) | Unclear risk | Quote: “Using a mixed factorial, simple randomization, double-blind study design, participants were assigned to one of two ECIG conditions…” (No further details given) |
| Allocation concealment (selection bias) | Unclear risk | No details on allocation given |
| Blinding of participants and personnel (performance bias) All outcomes | Unclear risk | Quote: “double-blind study design”, no further detail given |
| Blinding of outcome assessment (detection bias) All outcomes | Unclear risk | Quote: “double-blind study design”, no further details given |
| Incomplete outcome data (attrition bias) All outcomes | Low risk | Quote: “…80.6% completed the two-week intervention (n = 14 active; n = 11 placebo), and 70.9% also completed the follow-up session (n = 13 active; n = 9 placebo).”  Active follow-up completion rate: 13/14 = 93%; placebo follow-up completion rate: 9/11= 82%  N.B. 6 participants were disqualified post-randomization:  Quote: “Of those individuals who were screened for the study, 93.9% were enrolled (n = 18 active; n = 13 placebo); two individuals who were ineligible provided an expired air CO level < 10 ppm. Six of the enrolled participants (n = 4 active and n = 2 placebo; n = 5 tobacco flavour and n = 1 menthol flavour) were disqualified for responding to 7 or fewer days of text messages.” |
| Selective reporting (reporting bias) | Unclear risk | All measures listed were reported: self-reported cigarette use, text message-based cigarette use, e-cig use, expired air CO, readiness to quit ladder, withdrawal/side effects  No study protocol or clinical trial record available to confirm all intended outcome measures were reported. |

George 2019

| ***Study characteristics*** | | |
| Methods | Design: prospective, randomized controlled trial with a parallel, non-randomized preference cohort  Recruitment: participants were recruited from local advertisements, smoking cessation databases, and visits to local businesses, as well as via the Scottish Primary Care Research Network  Setting: single tertiary research centre, UK  Study start date: August 2016; Study end date: July 2018 | |
| Participants | Total N: 114 in “final evaluable dataset” (145 recruited into the trial)  N per arm: tobacco cigarettes (TC): 40; EC nicotine (16 mg): 37; EC nicotine-free: 37  Inclusion criteria: ≥ 18 years of age who had smoked ≥ 15 cigarettes/day for at least 2 years; free from established CV disease, diabetes, and chronic kidney disease (and not on medication for those conditions); willing to stop tobacco cigarettes for period of study if required and not to use EC if required.  Exclusion criteria: pregnant/breastfeeding; not abstaining from sex or using effective contraception; medication for CVD; history of CVD (excluding hypertension), diabetes, active malignancy or chronic renal disease; nut allergy; participation in another clinical trial (other than observational trials and registries) with an investigational product and/or intervention within 30 days before visit 1.  65.4% women; mean age 46.9; mean cpd 18.7  Motivated to quit: TC group: no; EC nicotine (16 mg): yes; EC nicotine-free: yes | |
| Interventions | **EC: Cig-a-like**  **EC nicotine** (16 mg) arm: EC containing 16 mg nicotine (Vapourlites Starter Kit with XR5 16 mg nicotine cartomizer; Vapourlites, Peterlee, United Kingdom)  **EC nicotine-free** arm: nicotine-free EC plus nicotine flavouring (Vapourlites Starter Kit with 0 mg nicotine cartomizer)  **(non-randomized) TC arm:** continued their usual daily smoking habits and did not use EC for the 4-week period of the trial | |
| Outcomes | Week 4  Adverse events and biomarkers: BP, heart rate, adverse events  Other outcomes measured: endothelial function, oxidised low-density lipoprotein, high-sensitivity C-reactive protein, tissue plasminogen activator, and platelet activation inhibitor-1 | |
| Study funding | "The VESUVIUS (Vascular Effects of Regular Cigarettes Versus Electronic Cigarette Use) trial was funded by the British Heart Foundation (grant PG/15/64/31681); and supported by Immunoassay Biomarker Core Laboratory, University of Dundee, the Tayside Medical Sciences Centre, and the NHS Tayside Smoking Cessation Service. The funder had no role in the study design, data collection, data analysis, data interpretation, writing of the report, or in the decision to submit for publication." | |
| Author declarations | "Dr. Donnan has received research grants from AbbVie, Shire, and Gilead Sciences. All other authors have reported that they have no relationships relevant to the contents of this paper to disclose." | |
| Notes | New for 2020 update | |
| ***Risk of bias*** | | |
| **Bias** | **Authors' judgement** | **Support for judgement** |
| Random sequence generation (selection bias) | Low risk | Consented participants who were willing to quit smoking were randomized to one of the EC arms in a 1:1 fashion using a centrally controlled web-based good clinical practices– compliant randomization system to either: 1) EC containing 16 mg nicotine; or 2) nicotine-free EC plus nicotine flavouring because it was considered by the institutional ethics committee as ethically unacceptable to randomize those who were willing to quit smoking into a smoking arm. Those unwilling to consider quitting smoking continued in the parallel preference TC cohort. |
| Allocation concealment (selection bias) | Low risk | Central randomization |
| Blinding of participants and personnel (performance bias) All outcomes | Unclear risk | Not specified |
| Blinding of outcome assessment (detection bias) All outcomes | High risk | Not blinded and AE/SAE data are self-report only. For other outcomes, low risk as objectively measured:  Quote: “Patients fasted overnight and measurements were conducted at baseline and 1 month according to the International Brachial Artery Reactivity Task Force guidelines (19) by a single operator (M.H.) blinded to study allocation at a single site.”  “Pulse wave velocity and augmentation index were measured at baseline and 1 month by a single operator (M.H.) blinded to study allocation.” |
| Incomplete outcome data (attrition bias) All outcomes | Unclear risk | Number randomized not provided per group  Quote: “A total of 145 patients were recruited into the trial. A final number of 114 patients (40 TC, 37 EC-nicotine, 37 EC-nicotine-free) completed both visits.” |
| Selective reporting (reporting bias) | Low risk | Clinical trial record lists: change in FMD; change in oxidized LDL; change in PAI-1; change in hs-CRP; change in pulse wave velocity; change in tissue plasminogen activator; change in augmentation index @ 75 bpm  All reported in the paper |

Goniewicz 2017

| ***Study characteristics*** | | |
| Methods | Design: longitudinal within-participant, observational  Recruitment: advertisements in the media, the internet, posted advertisements in clinics and offices, and by word of mouth  Setting: University, Poland  Study start date: March 2011; Study end date: June 2011 | |
| Participants | Total N: 22 started out and 2 dropped out in the first week due to an adverse event (nausea) and inability to commit to clinic visits. This resulted in an analytic sample of 20.  Inclusion criteria:   - 18 years or older, current daily cigarette smokers (> 5 cpd within the last 12 months) - May have had interest in quitting smoking, in good health (at the clinic screening visit) - Able to communicate in Polish - Able to use an e-cigarette safely   Exclusion criteria:   - Diagnosed as having asthma, COPD, hypertension, inhaled allergies, chronic heart disease, or cancer - Taking a cardiac medication - Pregnant   60% women; mean age 31; mean cpd 16; mean FTND 3.9  Motivated to quit: at the time of screening, 95% of participants (n = 19) reported planning to quit smoking, with 80% (n = 16) reporting that they have made at least 1 quit attempt prior to involvement in the study  E cigarette use at baseline: not reported | |
| Interventions | **EC: Cig-a-like**  Pen-style M201 e-cigarettes for 2 weeks, with an automatically-operated battery with an output power of 4.6 Volts (280 mAh) and the heating element resistance of 3.6 to 3.8 Ohms. At baseline, provided with EC (M201 Mild, Poland) with 20 tobacco-flavoured cartridges a week containing 11.0 ± 1.5 mg of nicotine in a mixture of propylene glycol and vegetable glycerin (50:50). Encouraged to substitute their regular cigarettes with the e-cigarettes for 2 weeks and refrain from smoking | |
| Outcomes | Day 7, Day 14  Adverse events and biomarkers:   - Biomarkers were metabolites of 13 major carcinogens and toxicants in cigarette smoke: 1 tobacco-specific nitrosamine (NNK), eight volatile organic compounds (1.3-butadiene, crotonaldehyde, acrolein, benzene, acrylamide, acrylonitrile, ethylene oxide, and propylene oxide), and 4 polycyclic aromatic hydrocarbons (naphthalene, fluorene, phenanthrene, and pyrene) - Questionnaire on ‘health’: at each visit, participants were asked, “In the last week, have you experienced any of the following symptoms?”, while providing a response of “never,” “rarely,” or “often” to the following list of health effects: daytime cough, difficulty concentrating, difficulty breathing during sleep, difficulty sleeping, dizziness, headache, irritability, nausea, nighttime cough, chest pain, phlegm, shortness of breath, tightness in chest, visual disturbances, and wheezing. Responses of “rarely” or “often” were combined to indicate presence of an adverse health effect. - Expired CO   Other outcomes measured:   - 7 nicotine metabolites (3-hydroxycotinine, cotinine, cotinine N-oxide, nicotine N-oxide, norcotinine, nornicotine, nicotine) - Revised Minnesota Nicotine Withdrawal Scale (MNWS-R) administered to measure ‘withdrawal symptoms’ (0-5 rating scale) | |
| Study funding | “This work was supported by the Ministry of Science and Higher Education of Poland (grant number N N404 025638). Instrumentation and analytical chemistry at UCSF was supported by the National Institutes of Health, P30 DA012393 and S10 RR026437. The study sponsor had no involvement in the study design, collection, analysis, and interpretation of data, the writing of the manuscript or the decision to submit the manuscript for publication.” | |
| Author declarations | "MLG was a faculty member of the Medical University of Silesia, Poland during the study. He received a research grant from Pfizer, a pharmaceutical company that markets smoking cessation medications. MLG and NLB have been consultants to pharmaceutical companies that market smoking cessation medications. NLB has been an expert witness in litigation against tobacco companies. The other authors declare no potential conflicts of interest." | |
| Notes | New for 2020 update | |
| ***Risk of bias*** | | |
| **Bias** | **Authors' judgement** | **Support for judgement** |
| Random sequence generation (selection bias) | High risk | Not randomized |
| Allocation concealment (selection bias) | High risk | Not randomized |
| Incomplete outcome data (attrition bias) All outcomes | Low risk | 2 dropouts – 1 for nausea, 1 could not complete clinic visits. Analysis based on 20 completers |
| Selective reporting (reporting bias) | Low risk | All outcomes reported |

Hajek 2015a

| ***Study characteristics*** | | |
| Methods | Design: prospective cohort, intervention provided  Recruitment: people who smoke attending stop-smoking service  Study start date: March 2014; study end date: March 2015  Setting: stop-smoking service, London, UK | |
| Participants | Total N: 100 (69 of whom accepted offer of EC)  Inclusion criteria:   - All people who smoked joining stop-smoking service   38% women (those who accepted), 55% women (those who declined), mean age 41, mean cpd 14, all motivated to quit. EC use at baseline not specified but some who declined EC offer had used EC in the past  Motivated to quit: yes  E-cigarette use at baseline: not specified | |
| Interventions | **EC: Cig-a-like and refillable**  EC: offered to all people who smoke joining service; offered choice of ‘cig-a-like’ (Gamucci, 1.6% or 2.2% nicotine per mL) product or tank model (EVOD, 1.8%; later replaced with Aspire product due to leakage issues). 69% of those offered received an EC on TQD  Medication: Offered stop-smoking medications including NRT and varenicline as in standard protocol. Of EC users, 33% opted to also use NRT, 29% varenicline, 38% nothing  Support: weekly, as in standard protocol | |
| Outcomes | Adverse events collected throughout, method for collection unclear  Also collected: 4-week biochemically validated abstinence, participant feedback, cost | |
| Study funding | "The pilot study was sponsored by City of London Corporation." | |
| Author declarations | "Peter Hajek received research funds from and provided consultancy to manufacturers of smoking cessation medications. The remaining authors have no conflicts of interest to declare." | |
| ***Risk of bias*** | | |
| **Bias** | **Authors' judgement** | **Support for judgement** |
| Random sequence generation (selection bias) | High risk | Not randomized |
| Allocation concealment (selection bias) | High risk | Not randomized |
| Incomplete outcome data (attrition bias) All outcomes | Unclear risk | 26% lost in EC group; dropout rate in EC decliners not reported. Reasons for dropout not stated |
| Selective reporting (reporting bias) | Unclear risk | Unclear which outcomes authors set out to collect, no protocol available |

Hajek 2019

| ***Study characteristics*** | | |
| Methods | Design: multicentre, pragmatic randomized controlled trial to examine the efficacy of e-cigarettes compared with nicotine replacement therapy  Recruitment: participants attending UK stop-smoking service and via social media  Setting: UK National Health Service stop-smoking services  Study start date: 1 April 2015; study end date: 31 March 2018 | |
| Participants | Total N: 886  N per arm: EC: 439; NRT: 447  Inclusion criteria:   - Adults who smoke (aged 18 years or over) with no strong preference to use or not to use nicotine replacement or e-cigarettes, and were currently not using either type of product - Able to read/write/understand English   Exclusion criteria:   - Pregnant or breastfeeding - Strong preference to use or not use NRT or EC, currently not using either type of product   48% women; median age 41; median cpd 15 ; mean FTND 4.6; 41.5% reported past use of ECs  Motivated to quit: not reported | |
| Interventions | **EC: Refillable**  **NRT:** Informed of range of NRT products (patch, gum, lozenge, nasal spray, inhalator, mouth spray, mouth strip, and microtabs) and selected preferred product, encouraged to use combination. Participants free to switch products. Supplies provided for up to 3 months  **EC:** Starter pack (1 Kit, Aspire UK) provided along with 30 mL bottle of Tobacco Royale flavour e-liquid, concentration 18 mg/mL. Participants showed how to use and asked to purchase future e-liquid online or from local vape shops and to buy different EC device if the 1 provided did not meet their needs. Encouraged to experiment with e-liquids of different strengths and flavours. If unable to obtain own supply, provided with further 10-mL bottle (not proactively offered). Oral and written info on how to operate EC  Both arms received multi-session behavioural support as per UK stop-smoking service practice (one-to-one sessions weekly with local clinicians, exhaled CO monitored for at least 4 weeks post-TQD); signed behavioural contract not to use other therapy for at least 4 weeks | |
| Outcomes | Weeks 4, 26, and 52  Cessation: sustained and biochemically validated CO < 8 ppm  Adverse events and biomarkers: “adverse reactions”: presence or absence of nausea, sleep disturbance and throat and mouth irritation, and respiratory symptoms (presence or absence of shortness of breath, wheezing, coughing, and phlegm), death  Other outcomes measured:   - Use and ratings of trial products - Rating of withdrawal symptoms (weeks 1 to 6) - Reduction of cigarette consumption - Cost-effectiveness | |
| Study funding | “Supported by the National Institute for Health Research (NIHR) Health Technology Assessment Programme (project number, 12/167/135) and by a grant (A16893) from the Cancer Research UK Prevention Trials Unit.” | |
| Author declarations | From ICJME disclosure forms: “Miss Natalie Bisal has nothing to disclose. Dr. Dawkins reports personal fees from Johnson & Johnson, outside the submitted work; Dr. Goniewicz reports personal fees from Johnson and Johnson, outside the submitted work; Dr. Hajek reports grants and personal fees from Pfizer, outside the submitted work; Ms. Li reports grants from NCCHTA, during the conduct of the study; Dr. McRobbie reports grants from NIHR HTA programme, during the conduct of the study; personal fees from Pfizer, personal fees from Johnson & Johnson, outside the submitted work; Dr. Myers Smith has nothing to disclose. Dr. Parrott has nothing to disclose. Dr. Pesola has nothing to disclose. Mrs Anna Phillips-Waller has nothing to disclose. Dr. Przulj reports grants from Pfizer, outside the submitted work; Dr. Ross has nothing to disclose. Dr. Sasieni has nothing to disclose. Ms. Wu has nothing to disclose." | |
| Notes | New for 2020 update, listed as ongoing study ISRCTN60477608 in 2016 review update  Note higher use of allocated product at 12 m in intervention group compared to control group: “Among participants with 1-year abstinence, 80% (63 of 79) were using e-cigarettes at 52 weeks in the e-cigarette group and 9% (4 of 44) were using nicotine replacement in the nicotine-replacement group.” | |
| ***Risk of bias*** | | |
| **Bias** | **Authors' judgement** | **Support for judgement** |
| Random sequence generation (selection bias) | Low risk | Quote: “Randomization took place on the quit date to limit differential dropout. Randomization sequences (1:1 ratio in permuted blocks of 20, stratified according to trial site) were generated with the use of a pseudorandom number generator in Stata software and were embedded into an application that only revealed the next treatment assignment once a participant had been entered into the database.” |
| Allocation concealment (selection bias) | Low risk | Refer to 'Random sequence generation' |
| Blinding of participants and personnel (performance bias) All outcomes | Low risk | Not blinded, but as both arms contained active interventions, performance bias judged unlikely |
| Blinding of outcome assessment (detection bias) All outcomes | Low risk | Biochemical validation used |
| Incomplete outcome data (attrition bias) All outcomes | Low risk | At 12 months:  EC arm: 356/439  NRT arm: 342/447 |
| Selective reporting (reporting bias) | Low risk | All prespecified outcomes reported |

Hajek 2022

| ***Study characteristics*** | | |
| Methods | Design: RCT multicentre  Participants: pregnant smokers (12 to 24 weeks gestation) who smoke daily and are interested in stopping smoking  Setting: maternity services across the UK. 23 hospital sites across England and one National Health Service Stop Smoking Service in Scotland  Recruitment: Recruitment was managed by research midwives in England and by the Stop Smoking Service in Scotland. Participants were identified from patient records and sent study information and invitation letters or invited via telephone, email or text; approached in person when attending antenatal hospital appointments; referred by community midwives or stop-smoking advisors; or self-referred via posters advertising the study at the sites’ antenatal clinics.  Study start date: 1 May 2017. Study end date: 26 November 2020 | |
| Participants | Total N: 1140  EC arm: 571  NRT arm: (nicotine patches) 569  Inclusion criteria:  12 to 24 weeks pregnant, daily smoker, wants help with stopping smoking. Willing to be randomized to use either NRT or EC and agreeing to use only the allocated stop-smoking product for at least the first 4 weeks of their quit attempt  Exclusion criteria:  Allergy to nicotine skin patches. Current daily use of NRT or e-cigarettes, and serious medical problems or high-risk pregnancy  Inclusion based on specific population characteristics: pregnant women  Female 100%; mean age 27; mean CPD 10  E-cigarette use at baseline: no  Motivated to quit: yes | |
| Interventions | EC: **Refillable**  Arm 1: EC  Participants were sent an EU Tobacco Product Directive-compliant refillable e-cigarette starter kit (One Kit by the UK E-cig Store), together with two 10 mL bottles of tobacco-flavoured e-cigarette liquid (1.8% nicotine; 70% propylene glycol and 30% vegetable glycerol), a pack of 5 replacement coils, and an instruction leaflet (Supplementary Data, Appendix 5). Further supplies of e-cigarette liquid were posted on request for up to 8 weeks. A lower-strength e-cigarette liquid (1.1%) and e-cigarette liquid with fruit flavour were available as alternatives. Participants were encouraged to source e-cigarette liquids of the strength and flavour they liked, as well as different e-cigarette devices, and arrange their own supplies after 8 weeks if needed. The cost of the kit provided by the study was GBP 22.75 and the cost of e-cigarette liquid was up to GBP 24 for an 8-week supply.  Products used during the initial 4 weeks (n = 344) # \* N (%): refillable e-cigarettes 324 (94.2%); cig-a-like 1 (0.3%); cartridge/pod 1 (0.3%); information missing 18 (5.2%)  Nicotine strength N (%): 0 mg/mL 7 (2.0); 1 to 10 mg/mL 47 (13.7); 11 to 20 mg/mL 199 (57.9); information missing 91 (26.5)  Arm 2: NRT - Nicotine patches  Participants were sent an initial 2-week supply of Nicorette Invisi 15 mg 16 h nicotine patches with manufacturer instruction leaflets and instructed to apply patches every day upon waking, and remove them before bedtime. Further supplies were posted on request for up to 8 weeks. A lower strength patch (10 mg 16 h) was available as an alternative. Participants were encouraged to access further supplies themselves via their general practitioner or local Stop Smoking Service. This could be patches and/or other NRT products such as nicotine chewing gum, inhalator or mouth spray, to use in addition to the patch alone if needed. In the United Kingdom, pregnant women who smoke receive NRT free of charge.  Behavioural support was given that accompanied both study arms.  Participants received 6 phone calls from stop-smoking advisors who followed the practice of the UK Stop Smoking Service 61. | |
| Outcomes | Baseline, weeks 1 to 4 after target quit date (TQD) (phone call), end of pregnancy (EOP) at least 6 months (saliva and CO), 3 months post-partum (phone call)  Cessation: saliva samples and carbon monoxide readings collected  AEs and SAEs  Continued use of study product  Flavours  EC nicotine strength | |
| Study funding | The study was funded by the National Institute of Health Research, Health Technology Programme (ref. no. 15/57/85). The funder had no role in the design and conduct of the study; collection, management, analysis, and interpretation of the data; preparation, review, or approval of the manuscript; or the decision to submit the manuscript for publication. For part of the trial, F.P. was supported by Cancer Research UK (grant no. C8162/A25356). | |
| Author declarations | P.H. provided consultancy to and received research funding from Pfizer. D.P. received research funding from Pfizer. H.M. has received honoraria for speaking at smoking cessation educational events and sitting on an advisory board organized by Pfizer. All other authors have no competing interests. | |
| Notes | New to 2022 update | |
| ***Risk of bias*** | | |
| **Bias** | **Authors' judgement** | **Support for judgement** |
| Random sequence generation (selection bias) | Low risk | Quote: "An independent statistician developed the randomization sequence using permuted block randomization with a block size of at least 6 and a maximum of 12". |
| Allocation concealment (selection bias) | Low risk | Quote: "The randomization list was accessible only to the independent statistician, on a secure server. Researchers conducting randomization used the database application to inform the participants of their study arm allocation. Researchers conducting follow-up calls were blind to treatment allocation until the follow-up contact was made". |
| Blinding of participants and personnel (performance bias) All outcomes | Low risk | Participants received equally intensive interventions. |
| Blinding of outcome assessment (detection bias) All outcomes | Low risk | Quote: "Researchers conducting follow-up calls were blind to treatment allocation until the follow-up contact was made. Once contact was made and the trial application was opened, condition-specific questions were visible on the computer screen. The trial statistician was blind to participant allocation until the analysis of the primary and secondary outcomes was complete. This was achieved by extracting and importing into Stata only the baseline characteristics, study arm and smoking status variables in the first stage of the analysis. Variables coding treatment adherence and product use were extracted only after the primary and secondary outcome analyses were completed". |
| Incomplete outcome data (attrition bias) All outcomes | Low risk | EC arm 515/571; NRT arm 495/569 |
| Selective reporting (reporting bias) | Low risk | Analysis prespecified |

Halpern 2018

| ***Study characteristics*** | | |
| Methods | Design: randomized clinical trial  Recruitment: eligible participants were employees and their spouses at 54 companies that used Vitality wellness programmes.  Setting: online resources via workplace setting (54 companies), USA  Study start date: first phase of recruitment October 2014, second phase November 2015 (to meet recruitment target); study end date: 20 April 2017 | |
| Participants | Total N: 6006  N per arm: usual care: 813; free e-cigarettes: 1199; free cessation aids: 1588; reward incentives plus free cessation aids: 1198; redeemable deposit plus free cessation aids: 1208  Inclusion criteria:   - At least 18 years old - Reported current smoking on a health risk assessment within the previous year - Employees and their spouses that used Vitality wellness programmes   Exclusion criteria:   - Participants who express wanting to opt out of this programme   51.1% women; median age 44; median cpd 10  E-cig use at baseline: 10.7% current use; 23.1% past but not current use; 39.7% never used ECs  Motivated to quit: unselected sample (total sample): 9.2% no plan to quit; 61.6% want to quit later; 27.7% want to quit/need help | |
| Interventions | **EC: Cig-a-like**  a) **Usual care:**  Standardized Vitality programme aimed at promoting tobacco cessation. This programme includes existing employee benefits for quitting and the use of text/email messages to encourage tobacco cessation.  b) as (a), plus **free EC**:  Free NJOY e-cigarettes (including battery sticks, a USB charger, and up to 20 chambers with 1.0% to 1.5% nicotine per week in participants’ chosen flavours). Use of all products was free until 6 months after the quit date.  c) as (b) plus access to free NRT, bupropion or varenicline  d) as (c) plus incentives across 6 m for testing negative for tobacco use  e) as (c) plus provide money at start and lose money from this fund if they do not test negative across 6 m | |
| Outcomes | Months 1, 3, 6, and 12  Cessation: sustained smoking abstinence for 6 months, biochemical validation (urine cotinine, anabasine and blood carboxyhaemoglobin)  Other outcomes measured: costs | |
| Study funding | "Supported by a grant from the Vitality Institute to the University of Pennsylvania Center for Health Incentives and Behavioral Economics." | |
| Author declarations | "Disclosure forms provided by the authors are available with the full text of this article at NEJM.org. Check these and: Dr. Troxel reports others from VAL Health, outside the submitted work. Dr. Volpp reports grants and personal fees from CVS Health, personal fees from VAL Health, grants from Humana, grants from Merck, grants from Weight Watchers, grants from Hawaii Medical Services Association, grants from Oscar Health Insurance, outside the submitted work. All of the other authors state that they have nothing to disclose." | |
| Notes | New for 2020 update. Study listed as ongoing study NCT02328794 in 2016 review update  Only arms (a) and (b) included in our analyses | |
| ***Risk of bias*** | | |
| **Bias** | **Authors' judgement** | **Support for judgement** |
| Random sequence generation (selection bias) | Unclear risk | Not specified |
| Allocation concealment (selection bias) | Unclear risk | Not specified |
| Blinding of participants and personnel (performance bias) All outcomes | High risk | Not blinded and different amounts of support given to each group |
| Blinding of outcome assessment (detection bias) All outcomes | Low risk | Biochemical validation |
| Incomplete outcome data (attrition bias) All outcomes | High risk | At 12 months, very low numbers completed biochemical validation. Submitted a sample n = CG: 1, free e-cigs; 4, free cessation: 5, rewards: 14, deposits: 16 |
| Selective reporting (reporting bias) | Low risk | Expected outcomes reported and checked with trial registration |

Hatsukami 2020

| ***Study characteristics*** | | |
| Methods | Design: randomized trial  Recruitment: media advertisements  Setting: clinic visits in community, USA  Study start date: 25 November 2014; study end date: 2 December 2018 | |
| Participants | Total N: 264  N per arm: usual brand: 36; AD-E: 76; CS-E: 76; CS-NRT: 76  Inclusion criteria: ≥ 18 years of age; smoking ≥ 5 cpd for the past year with a breath CO ≥ 10 ppm or NicAlert test = level 6 if CO < 10 ppm; in stable physical and mental health.  Exclusion criteria: serious quit attempt in the past 3 months; recent (< 3 months) alcohol or drug abuse problems; regular use of other nicotine or tobacco products (e.g. > 9 days per month to minimize confounding effects of these products on biomarker outcomes); planning to quit smoking in the next 3 months; chronic conditions affecting results of biomarker analyses (e.g. liver disease); using NRT or other cessation medications; pregnancy/breastfeeding  49% women; mean age 45.2; mean cpd 15.2; mean FTND 3.4  E-cigarette use at baseline: not reported  Motivated to quit: initially uninterested | |
| Interventions | **EC: Cig-a-like**, but the only cig-a-like product with high nicotine content  **Usual brand** arm: purchased their own usual brand of cigarettes; at end of clinical trial phase (week 8), offered ECs or NRT for up to 8 weeks, with a choice of product and no specific instructions for use  **EC AD-E** arm: use EC whenever you like instead of a cigarette; can smoke as many or as few cigarettes as you want  **EC CS-E** arm: complete substitution with e-cigarettes (i.e. “you will stop smoking cigarettes and use only e-cigarettes”)  The primary e-cigarette product was Vuse Solo (4.8% nicotine, manufactured by RJ Reynolds, Inc). Initially, a choice of Blu cigarettes (cartridge-based system, marketed previously by Lorillard) and Fin (prefilled tanks system, manufactured by Fin Branding Group) was offered; but because Vuse attained the highest market share during the early phase of the study, they switched exclusively to Vuse. Participants could choose 1 of 4 flavours: tobacco, mint, menthol, and berry. Participants were provided 7 cartridges a week with the option of returning to the clinic before their next visit to obtain additional cartridges if needed. All products provided free to the participants. All unused products and used EC cartridges were collected at each visit.  **CS-NRT** arm: complete substitution with 4 mg nicotine gum or lozenge, with the participant choosing what product they would like to use (i.e. “you will stop smoking cigarettes and use only nicotine gum or lozenge”). The 4 mg was down-titrated to 2 mg if adverse side effects were experienced. Nicotine gum came in mint, cinnamon, and fruit flavours, while the nicotine lozenge was mint or cherry flavours. All these products were provided free to the participants and unused products were collected at each visit.  Behavioural support: **CS-E arm** and **CS-NRT arm**: received brief counselling on how to avoid smoking cigarettes | |
| Outcomes | 2-week baseline period (weeks -1 and 0)  Week 1, 2, 3, 4, 6, and 8  Adverse events and biomarkers:   - Urinary total nicotine equivalents (total nicotine + total cotinine + total 3′-hydroxycotinine; TNE) - Exhaled CO - Urinary 4-(methylnitrosamino)-1-(3-pyridyl)-1-butanol and its glucuronides (total NNAL, biomarker for NNK) - Urinary phenanthrene tetraol (PheT, an indicator of carcinogenic polycyclic aromatic hydrocarbons) - Urinary metabolites of VOCs (mercapturic acids)—2-cyanoethylmercapturic acid (CEMA, biomarker for acrylonitrile), 3-hydroxypropylmercapturic acid (3-HPMA, biomarker for acrolein), 3-hydroxy-1-methylpropylmercapturic acid (HMPMA, biomarker for crotonaldehyde/methylvinyl ketone), 2-hydroxypropylmercapturic acid (2-HPMA, biomarker for propylene oxide), and N-acetyl-S-(carbamoylethyl)-L-cysteine(AAMA, biomarker for acrylamide) - A safety check for adverse events was conducted at week 20 follow-up - Blood pressure, heart rate, and oxygen saturation   Other outcomes measured:   - Cessation (< 6 months) | |
| Study funding | "supported by grants U19CA157345 from the National Cancer Institute (DKH/PS), UL1 TR000062 and UL1 TR002494 from the National Center for Advancing Translational Science of the National Institutes of Health, and T32 DA007097 from the National Institute of Drug Abuse (EM). The content is solely the responsibility of the authors and does not necessarily represent the official views of the funding agencies". | |
| Author declarations | "RJC is a member of the FDA Tobacco Products Scientific Advisory Committee. PGS serves or has served as an expert witness in tobacco company litigation on behalf of plaintiffs". | |
| Notes | New for 2020 update. AD-E arm not included in this review  Additional data provided from authors | |
| ***Risk of bias*** | | |
| **Bias** | **Authors' judgement** | **Support for judgement** |
| Random sequence generation (selection bias) | Unclear risk | Not specified |
| Allocation concealment (selection bias) | Unclear risk | Not specified |
| Blinding of participants and personnel (performance bias) All outcomes | Unclear risk | Not blinded and some interventions contained different levels of support |
| Blinding of outcome assessment (detection bias) All outcomes | Low risk | Not blinded but all relevant outcomes for our analyses were objective |
| Incomplete outcome data (attrition bias) All outcomes | Low risk | Quote: “There was a significant difference in dropout rates across groups following study entry (P = 0.04), with the highest dropout rates observed in the complete substitution groups, particularly in the NRT group…”  AD-E: week 1 = 73/76; week 2 = 73/76; week 4 = 69/76; week 6 = 66/76; week 8 = 65/76 = 85%  CS-E: week 1 =69/76; week 2 = 67/76; week 4 = 66/76; week 6 = 61/76; week 8 = 58/76 = 69.7%  CS-NRT: week 1 =72/76; week 2 = 65/76; week 4 = 60/76; week 6 = 57/76; week 8 = 53/76 = 69.7%  UB: week 1 = 35/36; week 2 = 35/36; week 4 = 33/36; week 6 = 33/36; week 8 = 32/36 = 88.8% |
| Selective reporting (reporting bias) | Low risk | Table in the supplementary section notes that heart rate, blood pressure, and oxygen levels were measured, but findings not reported in paper; however, provided by authors upon request |

Hickling 2019

| ***Study characteristics*** | | |
| Methods | Design: single-group assignment – pre-test post-test pilot study  Recruitment: Participants were referred from community mental health teams within the South London and Maudsley NHS Foundation Trust.  Setting: healthcare setting, UK  Study start date: 24 September 2014; Study end date: 2 May 2017 | |
| Participants | Total N: 50  Inclusion criteria: 18 to 70 years; daily smoker (unwilling to quit soon); exhaled CO ≥ 5 ppm; established clinical diagnosis of schizophreniform, schizophrenia, schizoaffective disorder or bipolar disorder, or attending an early detection service in a high-risk state  Exclusion criteria: use of EC ≥ 2 occasions in past 30 days; intention to quit smoking in the next 30 days; medication use that may reduce smoking (including, bupropion, nicotine replacement therapies, acamprosate, varenicline, baclofen, clonidine, naltrexone, buprenorphine, nortriptyline, disulfiram and anti-seizure medications); hospitalisation/change in dose of psychotropic medication(s) in the last 30 days; unstable physical health in the past 3 months; previous serious stomach ulcer and/or phaeochromocytoma; severe heartburn, stroke, unstable kidney/liver disease, an uncontrolled overactive thyroid gland past 3 months; meet the Diagnostic and Statistical Manual of Mental Disorders, Fourth Edition (DSM-IV) criteria for illicit/alcohol drug dependency; contraindications to nicotine; asthma; suicidal ideation/suicide attempt in the past month; pregnancy  Inclusion based on specific population characteristics: people who smoke tobacco with a psychotic disorder (established clinical diagnosis of schizophreniform, schizophrenia, schizoaffective disorder or bipolar disorder, or attending an early detection service in a high-risk state)  24% women; mean age 38.96; mean cpd 17.94; mean FTND not reported  Motivated to quit: “unwilling to quit soon”  E-cigarette use at baseline: must not have used e-cigarettes on more than 2 occasions in the past 30 days | |
| Interventions | **EC: Cig-a-like**  Participants provided with free tobacco-flavoured NJOY traditional bold disposable e-cigarette (4.5% nicotine) in an "amount equivalent to 150% of their daily tobacco use (as recommended by the manufacturer)" for 6 weeks. Participants were instructed in the use of EC; not required to stop smoking tobacco, but were encouraged to replace it with EC as much as possible. Followed up at 4 weeks and encouraged to continue EC use, informed about EC types and where these could be purchased | |
| Outcomes | Weeks 1, 2, 3, 4, 5, 6, 7, 8, 9, 10, 24  Self-reported and biochemical validation  Cessation: tobacco use, as measured by the Time Line Follow Back. Tobacco cigarette use was also indexed weekly by measuring exhaled CO levels with a Smokerlyzer ED50 CO meter (Bedfont Instruments, UK)  Adverse events and biomarkers:   - Side effects associated with e-cigarette use – reported weekly - Respiratory symptoms: lung capacity (measured by Wright’s Mini Peak-flow Meter (Clement Clarke International Ltd., UK) at baseline, weeks 6, 10, and 24; peak flow was obtained 3 times at each assessment - Heart rate and blood pressure - Occurrence of (serious) adverse events was assessed on a weekly basis.   In a subsample of participants (N = 8), 3-hydroxypropylmercapturic acid (3-HPMA, a measure of the toxicant acrolein) and formic acid were measured at baseline and week 6. These participants were chosen as their tobacco intake had decreased by more than 50% in this period. The measurement of 3-HPMA and formic acid was also performed by validated LC-MS/MS assays.  Other outcomes measured:   - Urinary cotinine - Weight - Motivation to Stop Scale (MTSS) - Smoking Consequences Questionnaire-Adult (SCQ-A) - Positive and Negative Syndrome Scale (PANSS) - Calgary Depression Scale for Schizophrenia (CDSS) | |
| Study funding | "This work was funded by the Maudsley Charity (grant number 715); and supported by the National Institute for Health Research (NIHR) Biomedical Research Centre at South London and Maudsley NHS Foundation Trust and King’s College London." | |
| Author declarations | "R.P-I. has received honoraria and speaker support from Lundbeck. L.D. has provided consultancy for the pharmaceutical industry (Johnson & Johnson 2015, 2017) and acted as an expert witness for an e-cigarette patent infringement case (Porzio, Bromberg & Newman Attorneys at Law, 2015). Between 2011 and 2013, she conducted research for several independent electronic cigarette companies (Totally Wicked, SKYCIGS and E-Lites) for which the University of East London received funds. The e-cigarette companies involved had no input into the design, conduct or write up of these projects and she has not received any funds from e-cigarette companies in the last 4 years. She has no links with, and has not received any funds from, the tobacco industry, although two e-cigarette companies that she worked with in 2013 were subsequently acquired by the tobacco industry (SKYCIGs and E-Lites). L.H., T.R., K-V.S., J.M., A.M. and P.M. have no conflicts of interest." | |
| Notes | Study listed as ongoing study NCT02212041 in the 2016 review update  Additional data provided from authors | |
| ***Risk of bias*** | | |
| **Bias** | **Authors' judgement** | **Support for judgement** |
| Random sequence generation (selection bias) | High risk | Uncontrolled study |
| Allocation concealment (selection bias) | High risk | Uncontrolled study |
| Incomplete outcome data (attrition bias) All outcomes | Low risk | Follow-up: week 6: 46/50; week 10: 42/50; week 24: 40/50 |
| Selective reporting (reporting bias) | Low risk | Reported all outcomes listed on clinical trials.gov except NNAL. Authors confirmed that they had intended to test for NNAL but had major issues with the assays. |

Higgins 2024

| ***Study characteristics*** | | |
| Methods | RCT  3 NCT trials data pooled: NCT04092387; NCT04090879; NCT04092101  NCT04092387. Low nicotine content cigarettes in vulnerable populations: women of reproductive age.  NCT04090879. Low nicotine content cigarettes in vulnerable populations: affective disorders.  NCT04092101 . Low nicotine content cigarettes in vulnerable populations: opioid use disorder.  All: Start Sept 2019. Estimated completion Jan 2024.  Setting: Johns Hopkins University, the University of Vermont, and Brown University, USA  Recruitment: Participants were recruited through newspaper and online advertisements and word of mouth.  Inclusion based on specific population characteristic: At risk populations (affective disorders; opioid use disorder; reproductive age females with lower educational levels).  EC use at baseline. Used EC in past 30 days = 42 (12.9 SD)  Motivated to quit: not planning to quit in the next 30 days | |
| Participants | Total N: 326 (3 trials)  Normal nicotine content tobacco cigarette only = 83 (70 at week 8; 67 at week 16)  VLNC = 85 (66 at week 8; 66 at week 16)  VLNC + EC tobacco flavour (TF) =74 (60 at week 8; 57 at week 16)  VLNC + EC preferred flavour (PF) = 84 (72 at week 8; 70 at week 16)  74.5% female (one of the three studies only recruited females). Mean age 40.09 (10.79 SD). Mean CPD 17.4 (8.87 SD). Mean FTND 5.09 (2.21 DS). Used EC in past 30 days = 42 (12.9 SD).  NCT04092387: Estimated enrolment 246. Inclusion criteria: Female; 21 to 44 years old, maximum educational attainment of graduating high school.  NCT04090879:Estimated enrolment 232 participants. Inclusion criteria: current diagnosis of an affective disorder.  NCT04092101: Estimated enrolment: 310. Inclusion criteria: Maintained on opioid medication methadone or buprenorphine treatment (methadone or buprenorphine treatment).  Shared inclusion criteria: 21 years and over (US legal age for purchasing cigarettes), 5 or more CPD in the past year, CO 8 ppm or higher, no psychiatric conditions with potential to interfere with study completion, sufficient literacy, no adverse health changes in the past 90 days, no use of EC daily or other tobacco products besides commercial cigarettes on 10 or more days in the past 30 days, and access to a computer or telephone for remote assessments (smart phone provided if necessary).  Shared exclusion criteria: prior regular use of VLNC cigarettes; plans to quit smoking in the next 30 days; a quit attempt in the past 30 days resulting in more than 3 days of abstinence; use of smoking cessation medications in the past 30 days; exclusive use of roll-your-own cigarettes; positive test result for drugs other than cannabis; binge drinking on 10 or more days in the past 30 days; abnormal vital sign measurements; positive COVID-19 symptoms or test result; recent suicidal ideation or suicide attempt; participation in another research study in the past 30 days; and cohabitation with another participant in the current study.Pregnant or breastfeeding, | |
| Interventions | 1) Normal nicotine content tobacco cigarette only (15.8 mg nicotine/g tobacco)  2) VLNC tobacco cigarette only (0.4 mg nicotine/g tobacco)  3) VLNC tobacco cigarette + EC tobacco flavour  4) VLNC tobacco cigarette + EC preferred flavour  Participants will be asked to use only their assigned study products for 16 weeks.  EC: (JUUL; Juul Labs) with pods containing 5% nicotine by weight. ECs were purchased from JUUL Labs. Those assigned to e-cigarettes participated in an orientation session and received a manual on device operation and storage. Participants received a supply of e-liquid pods sufficient to substitute for baseline smoking (1-pod/pack of cigarettes). EC flavours: tobacco flavour or preferred flavours. Preferred flavours arm: 8 flavors (classic tobacco, Virginia tobacco, crème brûlée, cucumber, fruit medley, mango, menthol, and mint) from which participants selected 3 preferred flavors. Participants selected 3 preferred flavors. Preferred flavors could be changed once during the study.  VLNC tobacco cigarette. During the first baseline visit, participants received free usual-brand cigarettes for use during the subsequent week to establish baseline CPD. The supply was 150% of self-reported CPD to accommodate increases. Participants received the first supply of study cigarettes at the second baseline visit. Participants received twice the number of cigarettes used during baseline to accommodate smoking increases or missed visits.  Normal nicotine content tobacco cigarette (15.8 mg nicotine/g tobacco). The National Institute on Drug Abuse provided study cigarettes with nicotine content averaged across menthol and non menthol cigarettes. Cigarettes were identical in appearance. Assignment to menthol or non menthol cigarettes was based on participant preference. During the first baseline visit, participants received free usual-brand cigarettes for use during the subsequent week to establish baseline CPD. The supply was 150% of self-reported CPD to accommodate increases. Participants received the first supply of study cigarettes at the second baseline visit. | |
| Outcomes | Baseline, weekly for 16 weeks.  AEs & SAEs. CPD. CO, NNAL, anatabine, biomarkers of tobacco toxicant exposure, brain function and structure, and airway inflammation (fractional nitric oxide concentration in exhaled breath (FeNO)). Urine at week 8 and 16 for: Urine specimens analyzed for cotinine (nicotine metabolite), anatabine (tobaccoalkaloid), and total 4-(methylnitrosamino)-1-(3-pyridyl)-1-butanol (NNAL, a tobacco-specific N-nitroasmine and carcinogen). Heart rate, BP. | |
| Study funding | This project was supported by Tobacco Centers of Regulatory Science grant U54DA036114 from the NIDA/NIH and FDA (Dr Higgins), Centers of Biomedical Research Excellence grant P30GM149331 from the National Institute of General Medical Sciences (Dr Higgins), and Institutional Training Award T32DA007242 from NIDA/NIH (DrsHeilandHiggins). | |
| Author declarations | Dr Tidey reported receiving a grant from the National Institute on Drug Abuseof theNationalInstitutes of Health (NIDA/NIH) during the conduct of the study and grants from the NIH outside the submitted work. Dr Klemperer reported receiving a grant from the NIH/NIDA and US Food and Drug Administration (FDA) during the conduct of the study. No other disclosures were reported. | |
| Notes | Higgins new to 2025 update. NCT records new to 2022 update as ongoing studies. | |
| ***Risk of bias*** | | |
| **Bias** | **Authors' judgement** | **Support for judgement** |
| Random sequence generation (selection bias) | Unclear risk | “Participants were allocated equally across 1 of 4 experimental conditions using block randomization, stratified by study site and menthol cigarette preference:” |
| Allocation concealment (selection bias) | Low risk | Protocol: “The Administrative Core will maintain the randomization schedule and the link between the alphabetic code and treatment assignment securely. A second sealed copy will be secured in a separate building to protect against loss related to fire or other unforeseen events. The University of Vermont will be responsible for removing all identifying information from cigarettes received from the Research Triangle Institute (RTI), labeling each carton with a blind code, assigning product using this blind code based on the randomization schedule being provided by the UVM Biostatistics Core, and shipping cigarettes and e-cigarettes to each site as needed based on recruitment.” (Quote from protocol)  From paper:“Cigarettes and e-cigarettes were studied under double-blind and open-label conditions, respectively. |
| Blinding of participants and personnel (performance bias) All outcomes | Low risk | Arms were equally intensive.  Cigarettes and e-cigarettes were studied under double-blind and open-label conditions, respectively.  “The nicotine doses will be identified by letter code and only Administrative Core personnel with no participant contact will have the link between thestatistician’s letter code and dose assignments. There will be no blinding of e-cigarette conditions.” |
| Blinding of outcome assessment (detection bias) All outcomes | Low risk | Measured (CO), Urine analysis for NNAL and anatabine |
| Incomplete outcome data (attrition bias) All outcomes | Low risk | At 16 weeks: NNC = 67/83; VLNC = 66/85; VLNC + EC (TF) = 57/74; VLNC + EC (PF) = 70/84 |
| Selective reporting (reporting bias) | Low risk | Reported CPD, cessation (to 16 weeks), SAE, AE, CO, NNAL, anatabine. Authors are working on a publication that will include the other measures ( heart rate and BP measures) |
| Other bias | Unclear risk | 3 different populations |

Hoeppner 2024

| ***Study characteristics*** | | |
| Methods | EC only arm (group) of randomised pilot trial  Setting: USA. Recruitment: phone screening. | |
| Participants | 29 in EC only group (no EMA). (30 in EC + EMA group. Total = 59)  15 in EC group at 3 month FU.  39% female; average age 44.1 (SD 12.6); CPD = 13 (SD 8.4)  For EC group only: FTND: 4.2 (2.4 SD)  Inclusion criteria: 18+; CC daily use for at least last 3 months; not using quit aids; interested to try EC; no experience of ECs.  Exclusion: pregnant, planning to become pregnant, or breast feeding.  EC use at baseline: no.  Motivation to quit CC (EC group): 7.9 (2.2 SD)  The sample was socio-economically disadvantaged. | |
| Interventions | EC: 2nd generation e-cigarette starter kit with the flavor of their choice (i.e., Classic Tobacco, Magnificent Menthol, or Variety Pack  Participants were compensated for their participation, with escalating compensation per lab visit with a total possible compensation of $120 for |EC only (non-EMA) group.  [The other arm not included here looked at role of NMA + ECs] | |
| Outcomes | CO. CC abstinence to 3 mths.  1-week, 1-month, and 3-month post EC initiation. | |
| Study funding | This research was supported by the National Institute of Health under grant award T32DA019426 (PI: Jacob Tebes) that covered part of Dr. Melissa Schick's effort, and grant award K99AA029154 (PI: Lourah Kelly) that covered part of Dr. Lourah Kelly's effort. The content is solely the responsibility of the authors and does not necessarily represent the official views of the NIH. The NIH had no role in study design; in the collection, analysis and interpretation of data; in the writing of the report; and in the decision to submit the article for publication. | |
| Author declarations | None | |
| Notes | New to 2025 update. | |
| ***Risk of bias*** | | |
| **Bias** | **Authors' judgement** | **Support for judgement** |
| Random sequence generation (selection bias) | High risk | Only used data from one arm so study treated as non-randomised |
| Blinding of outcome assessment (detection bias) All outcomes | Low risk | Expired breath carbon monoxide was assessed with a Bedfont piCO+ Smokerlyzer. Cotinine was assessed via saliva samples collected with cotton dental rolls (SalivaBio OralSwabs) placed inside the cheek. |
| Incomplete outcome data (attrition bias) All outcomes | Low risk | Non-EMA 15/29 (51.7%) at 3 months. |
| Selective reporting (reporting bias) | Unclear risk | The protocol /clinical record number is not included in the paper |
| Other bias | Low risk | None for the EC only arm. |

Holliday 2019

| ***Study characteristics*** | | |
| Methods | Design: pilot RCT  Recruitment: recruited via the Newcastle Dental Hospital and by primary care practitioners working in the north-east England region  Setting: dental clinical research facility (DCRF), located in the Newcastle Dental Hospital, Newcastle upon Tyne, UK  Study start date: 20 September 2016; study end date: 31 July 2018 | |
| Participants | Total N: 80  N per arm: Intervention group: 40; control group: 40  Inclusion criteria:   - Aged over 18 years old; smoker (≥ 10 cigarettes/day) - Willing and able to come to the DCRF for the required study visits - Having a minimum of 16 natural teeth (excluding third molars) - Being diagnosed with periodontitis   Exclusion criteria:   - Having used an e-cigarette for more than 2 days in the last 30 days - Infectious or systemic diseases that may be unduly affected by participation in this study - Haemodynamically unstable - Patients taking the medication adenosine (due to drug interaction risk) - Lack of capacity to be able to consent to the research project or inability to follow study instructions, or both - Participation in a dental research study within the previous 20 days - Pregnant by medical history, or nursing - Received any non-surgical periodontal therapy other than a routine scale and polish in the last 6 months - Currently undergoing or requiring extensive dental, orthodontic or implant treatment, or treatment for peri-implantitis   Inclusion based on specific population characteristics: periodontitis  52.5% women; mean age 44.36; mean cpd 17.4; mean FTND 5  Motivated to quit: not selected on motivation and not reported  E-cigarette use at baseline: not currently using an e-cigarette, or not having used 1 for more than 2 days in the last 30 days | |
| Interventions | **EC: Refillable**  All participants given standard stop-smoking advice (10 to 15 minutes in duration) and offer of referral to stop-smoking services  **Intervention**: given EC starter kit (Vype eTank clearomizer) and brief training on its use by a dentist. Provided with an approximately 2-week supply of e-liquid (20 ml) with a choice of flavour (Blended Tobacco, Crisp Mint, Dark Cherry and Vpure (flavourless)) and nicotine strength (0 mg/mL, 6 mg/mL, 12 mg/mL, 18 mg/mL) and information on where to buy more. EC intervention delivered directly following the standard stop-smoking advice and was expected to be 10 to 15 minutes in duration.  **Control group**: no further intervention | |
| Outcomes | Months 1 and 6; self-report and biochemical validation of smoking status  Cessation: rates of continuous eCO-verified smoking abstinence at 6 months were calculated following the Russell Standard (RS6)  Adverse events and biomarkers: expired air CO, adverse events monitored at each study visit  Other outcomes measured:   - Feasibility outcomes - Oral health outcomes - Smoking behaviour outcomes comprised: self-reported tobacco and e-cigarette use, eCO, e-salivary cotinine, salivary anabasine, FTND and Mood and Physical Symptoms Scale | |
| Study funding | "Richard Holliday is funded by a National Institute for Health Research Doctoral Research Fellowship (DRF-2015-08-077). This paper presents independent research funded by the National Institute for Health Research (NIHR). The views expressed are those of the authors and not necessarily those of the NHS, the NIHR or the Department of Health and Social Care." | |
| Author declarations | "The authors declare that they have no competing interests." | |
| Notes | New for 2020 update | |
| ***Risk of bias*** | | |
| **Bias** | **Authors' judgement** | **Support for judgement** |
| Random sequence generation (selection bias) | Low risk | Randomization was performed using a secure password-protected web-based system. |
| Allocation concealment (selection bias) | Low risk | Quote: "The randomisation allocation schedule will be generated by a statistician with no other involvement in the study to achieve concealment of allocation." |
| Blinding of participants and personnel (performance bias) All outcomes | High risk | Nature of study precluded blinding; different levels of support across intervention arms |
| Blinding of outcome assessment (detection bias) All outcomes | Low risk | Biochemical validation |
| Incomplete outcome data (attrition bias) All outcomes | Low risk | Attrition < 50% |
| Selective reporting (reporting bias) | Low risk | All prespecified outcomes were reported. |

Humair 2014

| ***Study characteristics*** | | |
| Methods | Design: prospective cohort  Recruitment: people attending an outpatient clinic  Setting: university hospital outpatient clinic, Switzerland  Study start date/end date: not specified | |
| Participants | Total N: 17  Inclusion criteria:   - Wish to reduce tobacco use or had failed to stop smoking using varenicline, bupropion or NRT in the past   Inclusion based on specific population characteristics: no  Mean 23 cpd, 82% had a psychiatric illness  Motivated to quit: yes  E-cigarette use at baseline: not specified | |
| Interventions | **EC: Cig-a-like**  Offered an EC with nicotine  59% also reported using NRT or varenicline in addition to EC. | |
| Outcomes | Smoking cessation and reduction by at least 30% at 12 months (self-report)  Adverse events  No significant side effects | |
| Study funding | Not specified | |
| Author declarations | Not specified | |
| Notes | Abstract only, hence little detail available  Not clear if EC was provided by clinic or if participants had to buy their own | |
| ***Risk of bias*** | | |
| **Bias** | **Authors' judgement** | **Support for judgement** |
| Random sequence generation (selection bias) | High risk | Prospective cohort |
| Allocation concealment (selection bias) | High risk | Not randomized |
| Incomplete outcome data (attrition bias) All outcomes | Unclear risk | Numbers lost to follow-up not reported |
| Selective reporting (reporting bias) | Unclear risk | Unable to determine prespecified outcomes |

Ikonomidis 2018

| ***Study characteristics*** | | |
| Methods | Design: (acute phase) randomized, cross-over assignment (outcomes measured within hours of the intervention and hence do not meet the criteria of 1 week or more); chronic phase: non-randomized, single-group assignment  Recruitment: hospital smoking cessation unit  Setting: hospital smoking-cessation unit, Greece  Study start date: 31 January 2017; study end date: estimated completion date: December 2021 | |
| Participants | Total N: 90  Inclusion criteria:   - Active conventional cigarette smoker - Adults 18 to 60 years   Exclusion criteria:   - Health condition adversely affected by smoking, history or presence of cardiovascular disease   Inclusion based on specific population characteristics: no  54% women; mean age 50.2; mean cpd 23.4; mean FTND: not reported  Motivated to quit: yes – recruited from smoking cessation unit  E-cigarette use at baseline: not reported | |
| Interventions | **EC: not clear**  E-cigarette details: in the chronic phase, all 70 participants were instructed to replace their conventional cigarettes (con-cig) with an e-cig containing nicotine (12 mg/dL (e-cig fluid with nicotine concentration of 12 mg/mL (propylene glycol 74.3%, glycerin 20%, flavouring 4.5%, nicotine 1.2%))) for 1 month. | |
| Outcomes | 1 month; self-report and objective measures  Cessation: self-report cessation at 1 month. CO measured at 1 month. Cessation data not used as < 6 months  Adverse events and biomarkers:   - Exhaled CO concentration - Heart rate; blood pressure   Other outcomes measured:   - Oxidative stress as assessed by malondialdehyde (MDA) plasma concentrations - Aortic stiffness as assessed by pulse wave velocity (PWV) and augmentation index (AIX75) | |
| Study funding | This study was supported by a grant from the Hellenic Cardiology Society and Hellenic Society of Lipidiology and Atherosclerosis. | |
| Author declarations | None | |
| Notes | New for 2020 update. Acute phase of trial not relevant for the review as very short-term outcomes. | |
| ***Risk of bias*** | | |
| **Bias** | **Authors' judgement** | **Support for judgement** |
| Random sequence generation (selection bias) | Unclear risk | Not specified |
| Allocation concealment (selection bias) | Unclear risk | Not specified |
| Blinding of participants and personnel (performance bias) All outcomes | High risk | Not blinded and differential levels of support given |
| Blinding of outcome assessment (detection bias) All outcomes | Unclear risk | Objective measures used for all outcomes reported |
| Incomplete outcome data (attrition bias) All outcomes | Low risk | 70 participants and 20 controls recruited – no dropout |
| Selective reporting (reporting bias) | Unclear risk | NCT record stated that chronic endothelial integrity, platelet aggregation and high-shear stress-dependent platelet function would be assessed but these outcomes were not reported in this research letter. |
| Other bias | Unclear risk | Few details – written as commentary. Trial registration suggests this is an ongoing study. |

Ikonomidis 2020a

| ***Study characteristics*** | | |
| Methods | RCT  Recruitment: smoking cessation clinic of second cardiology department of National and Kapodistrian University of Athens, Attikon General Hospital  Setting: hospital smoking-cessation unit, Greece  Study start date: NS | |
| Participants | N = 40; Arm 1 e-cigarette n = 20; Arm 2 conventional tobacco cigarette n = 20  80% female; mean age 44.8 (SD 11.3); mean cpd: 25.8 (C-cig 25.5 (SD 9.3), E-cig: 26.2 (SD 9.1))  Inclusion criteria: smokers without cardiovascular disease, who used to smoke 25.8 ± 9.2 conventional cigarettes per day of their choice  Exclusion criteria: abnormal renal function; hepatic failure (bilirubin > 2 mg/dL); active malignancy; people treated with drugs that affect platelet function; history of coronary artery disease or peripheral artery disease; history of cardiomyopathy; thrombocytopenia (PLTs < 100 × 109 /L); anaemia (HCT < 28%); alcohol or drug abuse; age < 21 years; pregnancy; risk factors for cardiovascular disease | |
| Interventions | **EC: Refillable**  E-cig: second-generation e-cig device and popular in Greek Market e-liquid (NOBACCO eGo Epsilon BDC 1100, eGo battery, 1100 mAh, operating at 3.9 V - propylene glycol 74.3%, glycerin 20%, flavouring 4.5%, nicotine 1.2%/12 mg/mL) | |
| Outcomes | Baseline, 4 months: exhaled CO concentration; blood pressure  Also, cpd; platelet function by Platelet Function Analyzer PFA-100 and Light Transmission Aggregometry; pulse wave velocity; plasma malondialdehyde levels as oxidative stress index | |
| Study funding | "There was no funding for this study". | |
| Author declarations | "The authors declare that they have no known competing financial interests or personal relationships that could have appeared to influence the work reported in this paper." | |
| ***Risk of bias*** | | |
| **Bias** | **Authors' judgement** | **Support for judgement** |
| Random sequence generation (selection bias) | Low risk | Table of random numbers as reproduced from the online randomization software www.graphpad.com/quickcalcs/index |
| Allocation concealment (selection bias) | Unclear risk | Not specified |
| Blinding of participants and personnel (performance bias) All outcomes | Unclear risk | Not possible |
| Blinding of outcome assessment (detection bias) All outcomes | Low risk | Only CO outcomes used here, which were objectively measured |
| Incomplete outcome data (attrition bias) All outcomes | Low risk | All followed up (confirmed via contact with authors) |
| Selective reporting (reporting bias) | Unclear risk | No protocol or clinical trial record available to confirm whether all prespecified criteria were reported |

Ikonomidis 2020b

| ***Study characteristics*** | | |
| Methods | Design: RCT  Setting: Smoking Cessation Clinic of Second Cardiology Department of National and Kapodistrian University of Athens, Attikon General Hospital, Greece | |
| Participants | 40  Inclusion criteria: current smokers without cardiovascular disease | |
| Interventions | EC type: NS  Conventional cigarette (conv-cig) or an electronic cigarette (e-cig) with nicotine concentration 12 mg/dL for 1 month | |
| Outcomes | a) Perfused boundary region (PBR) of the sublingual arterial micro vessels (range 5 to 25 micrometres), a marker inversely related with glycocalyx thickness  b) Pulse wave velocity (PWV), central systolic blood pressure (cSBP), and augmentation index (AIX)  c) Platelet function by 2 different methods, namely the novel Platelet Function Analyzer PFA-100 and the traditional Light Transmission Aggregometry (LTA)  d) Exhaled CO level (ppm) as a smoking status marker  e) Plasma malondialdehyde (MDA) levels, as an oxidative stress burden index | |
| Study funding | Funding acknowledgement: type of funding source: none | |
| Author declarations | NS | |
| Notes | Information extracted from a conference abstract | |
| ***Risk of bias*** | | |
| **Bias** | **Authors' judgement** | **Support for judgement** |
| Random sequence generation (selection bias) | Unclear risk | No detail given  Quote: "40 current smokers (mean age 48 years±5) without cardiovascular disease were randomized to smoke either a conventional cigarette (conv-cig) or an electronic cigarette (e-cig) (electronic cigarette fluid with nicotine concentration of 12 mg/dL) for one month." |
| Allocation concealment (selection bias) | Unclear risk | NS |
| Blinding of participants and personnel (performance bias) All outcomes | High risk | No blinding |
| Blinding of outcome assessment (detection bias) All outcomes | Unclear risk | NS |
| Incomplete outcome data (attrition bias) All outcomes | Unclear risk | NS |
| Selective reporting (reporting bias) | High risk | Results reported in summary form, not fully, with only some significant changes reported. Information taken from a conference abstract only |

Ikonomidis 2024

| ***Study characteristics*** | | |
| Methods | RCT  Setting: smoking cessation clinic, Greece. National & Kapodistrian University of Athens, Attikon University Hospital, 2nd Cardiology Department, Athens, Greece  Recruitment: subjects attending smoking cessation clinic | |
| Participants | N= 100  EC arm = 25; Heat not burn cigarette arm = 25; Control arm = 50  % female: EC = 51%; Heat not burn cigarette = 55%; CC = 53%.  Age: EC: 45 ± 8 years; Heat not burn cigarette 46 ± 14 years; CC 48 ± 5 years.  Mean CPD: EC: used 27 ± 10 cigarettes/day, 28 ± 10 pack-years; Heat not burn cigarette: 26 ± 8 cigarettes/day, 30 ± 8 pack-years; CC: Used 27 ± 9 cigarettes/day, 29 ± 9pack-years.  Inclusion: CC user.  Motivated to quit: yes | |
| Interventions | EC; Heat not burn cigarette; Control arm | |
| Outcomes | Baseline, 1 month.  CO.  Smoking status was verified by measuring the concentration of exhaled carbon monoxide [(e CO) (ppm), Bedfont Scientific, Maidstone, Kent UK]  Endothelial glycocalyx thickness measured by Perfused Boundary Region (PBR) | |
| Study funding | Reported as: 'None'. | |
| Author declarations | None | |
| Notes | New to 2025 update | |
| ***Risk of bias*** | | |
| **Bias** | **Authors' judgement** | **Support for judgement** |
| Random sequence generation (selection bias) | Unclear risk | State that study is randomised but no detail is provided. |
| Allocation concealment (selection bias) | Unclear risk | Not reported. |
| Blinding of participants and personnel (performance bias) All outcomes | Low risk | EC & heat not burn cigarettes equally intensive. |
| Blinding of outcome assessment (detection bias) All outcomes | Low risk | Concentration of exhaled carbon monoxide [(e CO) (ppm), was measured by Bedfont Scientific, Maidstone, Kent UK]. |
| Incomplete outcome data (attrition bias) All outcomes | Unclear risk | Not reported. |
| Selective reporting (reporting bias) | Unclear risk | Reported as stated in abstract. No clinical record or protocol. |

Ioakeimidis 2018

| ***Study characteristics*** | | |
| Methods | Design: randomized controlled trial  Recruitment: not specified  Setting: hospital, Greece  Study start date/Study end date: not specified | |
| Participants | Total N: 54  N per arm: arm 1: 27; arm 2: 27  Inclusion criteria: ≥ 10 cpd; motivation to quit; hospitalized with acute coronary syndrome (ACS); ≥ 18 years  Exclusion criteria: prior EC use; history of neuropsychiatric disorders; prior varenicline use or use of smoking cessation pharmacotherapy at time of ACS; cardiogenic shock or renal impairment; hepatic impairment prior to ACS; excessive alcohol use or current use of marijuana or non-cigarette tobacco products  Inclusion based on specific population characteristics: People who have experienced acute coronary syndrome  65% women; mean age 52; mean cpd 21; mean FTND 5.6  Motivated to quit: yes  E-cigarette use at baseline: no prior EC use | |
| Interventions | **EC:** information on whether cig-a-like or refillable not provided  Both arms given "low intensity counselling"  **Intervention 1:** 12-week use of EC 12 mg/mL nicotine  **Intervention 2:** 12-week varenicline | |
| Outcomes | Weeks: 4, 12, 24  Cessation: 7-day PP at 24 weeks, self-report  Adverse events and biomarkers: unclear how these were reported. Abstract says no SAEs, poster implies this may have just been CV or neuropsychiatric SAEs. Abstract says nothing about AEs but nausea and sleeping disorders given in table in poster. Implies (S)AEs collected during treatment period only  Other outcomes measured: not specified | |
| Study funding | Not reported | |
| Author declarations | Not reported | |
| Notes | New for 2020 update. Abstract and poster only; limited data available | |
| ***Risk of bias*** | | |
| **Bias** | **Authors' judgement** | **Support for judgement** |
| Random sequence generation (selection bias) | Unclear risk | Not specified |
| Allocation concealment (selection bias) | Unclear risk | Not specified |
| Blinding of participants and personnel (performance bias) All outcomes | Low risk | Not specified but equal amounts of contact and support between arms so performance bias judged unlikely |
| Blinding of outcome assessment (detection bias) All outcomes | Low risk | Self-report only but equal amounts of contact between arms; no reason to suspect differential misreport |
| Incomplete outcome data (attrition bias) All outcomes | Unclear risk | Not specified |
| Selective reporting (reporting bias) | Unclear risk | Abstract/poster only so not able to judge |
| Other bias | High risk | Abstract and poster only. Two different figures presented for quit rate in EC arm (no difference in those presented in varenicline arm) between abstract and poster. Poster percentage aligns with figure, so using that (16.5%) as opposed to abstract figure (32.5%). Contacted authors but no reply. Calculated n quit based on percentages but unclear what denominators were; EC calculates back to whole number for EC but not for varenicline. |

Kale 2025

| ***Study characteristics*** | | |
| Methods | RCT  Setting: hospitals, UK  West Park Hospital; Bradford District Care NHS Foundation Trust; Sheffield Clinical Commissioning Group | |
| Participants | N= 43 [The target was 72]  EC kit arm = 21; Control (usual care) = 22  39.5% female; mean age 45.2 (SD=12.7); mean CPD 19.6 (10.9). Strength of urges to smoke = 3.4 (mean), (1.0 SD). Past year EC use for smoking cessation = 27.9% (n=12). Past yr quit attempt = 37.2% (n=16)  Inclusion criteria: receiving treatment for a mental illness; > 18 years; smoker; willing to attempt to quit  Exclusion criteria: inpatient admission in the last 3 months according to their health care record; smokers using EC; participating in other smoking cessation trials; being treated for comorbid drug or alcohol problems; Alzheimer’s disease or dementia; pregnancy or breastfeeding.  Specific population: All participants had a diagnosed mental health condition and were receiving treatment in primary or secondary care  Motivated to quit: willing to quit (Motivated to quit smoking 5.2 mean (1.7 SD) (motivation score 1-7, higher score = higher motivation))  EC use at baseline: | |
| Interventions | EC: Aspire PockeX electronic cigarette  EC starter kit : third- generation e-cigarette (Aspire PocketX) with a four-week supply of a choice of: a) nicotine strength e-liquid (3 options: 6mg/ml, 10mg/ml, 18mg/ml) and b) flavours (tobacco, fruit, menthol) as an adjunct to usual care. Verbal explanation and demonstration, and information leaflet on how to use the EC.  Control arm: usual care.  Usual care arm. Minimum standard this would involve evidence-based Very Brief Advice to stop smoking, comprising the three As (Ask and record smoking status; Advice on the best way of quitting and; Act on patient response to build confidence) and referral to in-house or external specialist stop smoking services.”Encouraged to condider quitting, set a quit date within a week after randomisation, or reduce cigarette consumption. Possibly offered NRT as part of usual care.  Both arms: “To support engagement, all participants (both in intervention and control group) received a £10 love2shop voucher for completing the baseline assessment and a £10 love2shop voucher for completing the 1-month follow-up assessment.” | |
| Outcomes | Baseline, 2 to 4 weeks. Baseline and 4 weeks reported in Kale 2025.  Primary outcome measure:   1. Feasibility and acceptability outcomes: consenting rate and recruitment frequency. 2. Clinical (smoking-related) outcomes: continuous abstinence assessed at 1 month will be defined as not having smoked in the 2 weeks prior to follow-up, verified by a CO reading below 10 ppm   Secondary outcome measures:   1. Feasibility and acceptability outcomes. Characteristics of ‘usual care’ in different locations will also be noted, recording 2 interactions of patients with CMHTs or GPs at each site at baseline and using short pro-forma with control group participants at 1-month follow-up. 2. Clinical (smoking-related) outcomes: Self-reported abstinence 2 to 4 weeks from enrolment or target quit date (whichever is later) will be recorded at 1-month follow-up. The change in cigarette consumption (and reduction in exhaled breath CO reading) from baseline to 1-month follow-up will be calculated in both intervention and control group participants. 3. Clinical (mental health-related) outcomes 4. Cost-effectiveness 5. SAE and AE   Number of AEs and SAEs provided by author | |
| Study funding | "This work was supported by Yorkshire Cancer Research (Y433). DK receives salary support from Cancer Research UK (PRCRPG-Nov21\100002). For the purpose of Open Access, the author has applied a CC BY public copyright licence to any Author Accepted Manuscript version arising from this submission. Funders were not involved in the collection, management, analysis, and interpretation of data; writing of the report; or in the decision to submit the report for publication." | |
| Author declarations | "LS has received honoraria for talks, unrestricted research grants and travel expenses to attend meetings and workshops from manufactures of smoking cessation medications (Pfizer; J&J) and has acted as paid reviewer for grant awarding bodies and as a paid consultant for health care companies. All authors declare no financial links with tobacco companies, e-cigarette manufacturers, or their representatives." | |
| Notes | New to 2025 update. Ongoing study added to 2022 update | |
| ***Risk of bias*** | | |
| **Bias** | **Authors' judgement** | **Support for judgement** |
| Random sequence generation (selection bias) | Low risk | “intervention allocation was determined by computer block-randomisation” |
| Allocation concealment (selection bias) | Low risk | “Randomisation occurred after consent to take part in the study had been obtained via opening of consecutively numbered opaque envelopes containing information about allocation.”  “Allocation was concealed until after completion of the baseline questionnaire.” |
| Blinding of participants and personnel (performance bias) All outcomes | High risk | Interventions were not equally intensive . “Participants and researchers and clinical staff administering the intervention could not be blinded due to the nature of the intervention and study design. As follow-up questionnaires differed for intervention and control groups, outcome assessment was only blinded to researchers for questionnaires self-completed online rather than over the phone. However, the study’s statistician was blinded to participants’ allocation.” Some blinding |
| Blinding of outcome assessment (detection bias) All outcomes | Low risk | AEs & SAEs are used in this review, self-reported is usual for these measures. Other data collected by questionnaire - high. |
| Incomplete outcome data (attrition bias) All outcomes | Low risk | At 4 weeks, both <50%: EC = 15/21; Control = 12/22 |
| Selective reporting (reporting bias) | Low risk | Outcomes reported. |

Kanobe 2022\*

| ***Study characteristics*** | | |
| Methods | Design: 2-centre, randomized, controlled, switching, open-label, parallel-group, clinical confinement study  Higher versus lower nicotine study  Setting: USA. Two clinical research sites (Overland Park, KS and Minneapolis, MN), contracted by Altasciences Clinical Research as the contract research organization (CRO)  Recruitment: potential participants completed a pre-screening telephone interview and also attended a screening visit at the clinical research site within 30 days before study entry.  Study start date: April 2017. Study end date: September 2017 | |
| Participants | 125 randomized:  Kanobe 2022 reports EC Vuse Solo arm  EC (Vuse Solo) group: 35  Female 42.9% female; mean age 41.2 years; mean CPD: 18.3 (SD 10.9); mean FTND: 6.1 (SD 1.6)  Kanobe 2023 reports on Vuse Vibe and Vuse Ciro arms:  37 Vuse Vibe group: 37  37 Vuse Ciro group: 37  35.6% (32) female; mean age 40  [Abstinence arm: 16 (this arm is ignored)]  Inclusion criteria: M or F; 21 to 64 yrs; smoker using non-menthol tobacco cigarettes (at least 10 cpd); not planning to quit  Exclusion criteria: medical conditions; weight of ≤ 110 pounds; planning to quit within 30 days  EC use at baseline: no  Motivated to quit: no | |
| Interventions | EC: Vuse Solo Original ENDS product referred to as Vuse Solo (G2) or Vuse Solo. Pod. Rechargeable Vuse Solo power unit (battery capacity: ≥ 270 mAh; wattage: 3.00 W) and a closed cartridge containing 0.5 mL of original (tobacco-flavoured) e-liquid, a mix of propylene glycol (PG), glycerin (PG:glycerin ratio of 21:79), water, and flavouring ingredients. The e-liquid included nicotine at 4.8% nicotine by weight (57.4 mg/mL) and contained nicotine salts.  EC: Vuse ciro 1.5% nicotine EC. pre-filled, closed ENDS products (RJR Vapor Company, Winston-Salem, NC, USA. Original, tobacco-flavored, nicotine containing e-liquids. The Vuse Ciro product was originally marketed under the brand name “Vuse Solo+”. The product was subsequently renamed to “Vuse Ciro”. Tobacco flavour.  EC: Vuse vibe, 3% nicotine EC. The Vuse Vibe power unit is powered by a ≥ 550 -mAh rechargeable battery and is paired with a cartridge containing 1.9 mL of e-liquid with 3% nicotine content by weight (36 mg/mL) and a propylene glycol-to-glycerin ratio (PG/VG) of 20/80. The maximum puff duration for Vuse Vibe is 6 s. Tobacco flavour | |
| Outcomes | Day 1, 5, 7 (urine)  AEs, SAEs monitored throughout study from day 1 to day 8 (discharge)  Most outcomes were measured at day 5 and not eligible for extraction. | |
| Study funding | “This study was funded by RAI Services Company.“  Tobacco industry funded. Parent company is Reynolds American. Reynolds American manufacture and market a variety of tobacco products, including cigarettes (Newport, Camel, Pall Mall, Kent, Doral, Misty, Capri, and Natural American Spirit brands), electronic cigarettes (Vuse brand), and moist snuff (Grizzly and Kodiak brands).  Kanobe 2023: This research was funded by RAI Services Company, a wholly owned subsidiary of Reynolds American Inc., which is an indirect, wholly owned subsidiary of British American Tobacco plc. | |
| Author declarations | M.K., P.C., J.D., P.M., E.R., J.W.C., and E.S. are full-time employees of RAI Services Company, and B.G.B., B.A.J., G.L.P., and P.N. are former full-time employees of RAI Services Company. RAI Services Company and R.J. Reynolds Tobacco Company are wholly owned subsidiaries of Reynolds American Inc., which is a wholly owned subsidiary of British American Tobacco plc. G.L.P. is an independent consultant. B.N. is a full-time employee of JTI Leaf Services (US) LLC.  Kanobe 2023: M.N.K., P.M., P.C. and J.W.C. are full-time employees of RAI Services Company, E.K.R. is a full-time employee of BAT (Investments) Limited, and B.G.B., G.L.P. and P.R.N. are former full-time employees of RAI Services Company. G.L.P. works as an independent scientific consultant. RAI Services Company is a wholly owned subsidiary of Reynolds American Inc., which is an indirect, wholly owned subsidiary of British American Tobacco plc | |
| Notes | Participants were compensated for their time and participation. For the purposes of our analyses, this study is treated as a single-arm study and reported narratively. | |
| ***Risk of bias*** | | |
| **Bias** | **Authors' judgement** | **Support for judgement** |
| Random sequence generation (selection bias) | Low risk | A computer software program was used by the statistician at the CRO to generate the randomization sequences. |
| Allocation concealment (selection bias) | Low risk | A computer software program was used by the statistician at the CRO to generate the randomization sequences. |
| Blinding of participants and personnel (performance bias) All outcomes | High risk | “The study was unblinded by necessity due to the very different visual appearances of the study products” |
| Blinding of outcome assessment (detection bias) All outcomes | Low risk | Relevant outcomes are biomarkers, which are objectively measured |
| Incomplete outcome data (attrition bias) All outcomes | Low risk | Vuse solo group 35/35; Vuse Vibe group 33/37; Vuse Ciro group 35/37. |
| Selective reporting (reporting bias) | High risk | The NCT record stated that some biomarkers would be measured at 5 days. Extra biomarkers have been reported at 7 days follow-up. |

Katz 2025

| ***Study characteristics*** | | |
| Methods | Design: Randomized, cross-over, open-label. Two consecutive randomly ordered 2-week phases  Setting: Greater Burlington, VT, USA. University of Vermont, USA | |
| Participants | N=21  Age 'at least 40 years old’.  Inclusion: smoke (≥5 cigarettes/day for ≥1 year); diagnosed with COPD, chronic bronchitis, emphysema, or asthma-COPD overlap syndrome); no intention to quit smoking within the next month.  Exclusion criteria: patients who are medically unstable (unstable symptoms, changes in medications or hospitalizations within last 3 months); inability to conduct in-home measurements.  Not motivated to quit smoking.  Inclusion based on specific population characteristics: diagnosed with COPD | |
| Interventions | From paper.  Cigarette phase, usual brand CCs  Nicotine-containing EC phase. EC: 3% and/or 5% nicotine tobacco-flavored JUUL available. During the EC phase, participants earned monetary incentives for CO readings ≤6ppm to promote cigarette abstinence.  From NCT record:  EC: JUUL/Vuse Alto and pods  1) Experimental: e-cigarette. Participants in this arm will smoke EC for 2 weeks. EC (either JUUL or Vuse Alto) and pods (JUUL: Virginia tobacco flavour at 3% or 5% nicotine concentration; Vuse Alto: golden tobacco flavour at 1.8%, 2.4%, or 5% nicotine concentration) will be provided. In the EC arm, availability of EC and altering the availability of financial incentives for abstaining from combustible cigarettes will be investigated.  2) No intervention: CC. Participants in this arm will smoke their usual brand of combustible cigarettes for 2 weeks. | |
| Outcomes | Baseline, 2 weeks.  CO  Pulmonary (spirometry, oscillometry, COPD Assessment Test [CAT], Saint George’s Respiratory Questionnaire for COPD [SGRQ-C]) and cardiac (heart rate, blood pressure) assessments were completed at baseline and after each phase.  From NCT record. Baseline, 2 weeks, 4 weeks  Baseline and change from baseline: FEV1/FVC; lung reactance; oxygen saturation (SpO2); exhaled nitric oxide (FeNO); COPD; blood pressure; heart rate; tobacco use; Fagerstrom Test of Nicotine Dependence (FTND); Wisconsin Inventory of Smoking Dependence Motives-Brief (WISDM-Brief); Minnesota Tobacco Withdrawal Scale (MNWS); Questionnaire on Smoking Urges-Brief (QSU-Brief); health changes | |
| Study funding | Tobacco Centers of Regulatory Science (TCORS) Award U54DA036114 from the National Institute on Drug Abuse (NIDA) and Food and Drug Administration (FDA). Content is solely the authors’ responsibility and does not necessarily represent the official views of these institutions | |
| Author declarations | No conflicts for any of the authors (email correspondence). | |
| Notes | Ongoing study moved to included study in 2025 update. Contact information: Brian R Katz, Katz, Brian brkatz@UTMB.EDU Diann Gaalema Diann.Gaalema@uvm.edu. | |
| ***Risk of bias*** | | |
| **Bias** | **Authors' judgement** | **Support for judgement** |
| Random sequence generation (selection bias) | Unclear risk | Conference abstract, minimal detail |
| Allocation concealment (selection bias) | Unclear risk | Conference abstract, minimal detail |
| Blinding of participants and personnel (performance bias) All outcomes | Unclear risk | Conference abstract, minimal detail |
| Blinding of outcome assessment (detection bias) All outcomes | Low risk | All outcomes measured objectively |
| Incomplete outcome data (attrition bias) All outcomes | Unclear risk | Conference abstract, minimal detail |
| Selective reporting (reporting bias) | Unclear risk | Only conference abstract published so far |

Kerr 2020

| ***Study characteristics*** | | |
| Methods | Design: RCT. Pragmatic, 2-armed, single-centre, randomized controlled pilot study  Recruitment: community. Recruitment from NHSGGC Smokefree Services. GP surgeries. Secondary care QEUH, GRI, New Victoria Hospital and Glasgow Dental School. Advertized on NHSGGC staff payslips, local newspapers, community magazines, and on Gumtree. The British Heart Foundation, University of Glasgow and NHSGGC released a joint press release, which generated media coverage in 4 newspapers. Social media. Public engagement “pop up stands” were held collaboratively with smoke-free services and the VAPOUR study team in the entrance foyer of the QEUH and at the Celtic Football Club Healthy Hoops Event.  Setting: Scotland, UK  Study start date: 1 December 2015. Study end date: 13 July 2018 | |
| Participants | Total N: 55  EC arm 28. NRT arm 27  Inclusion criteria:  Aged 18 to 65 yrs; habitual tobacco smokers (smoking on average 1 to 15 tobacco cigarettes pd > 6 months); willing to quit tobacco smoking; with either the use of nicotine replacement patches or an EC with nicotine-containing e-liquid, in addition to engaging with NHSGGC Community Smokefree Service's 12-wk behavioural support programme. No established history of cardiovascular disease  Exclusion criteria:  Pregnant or breastfeeding; had used an EC or nicotine replacement patch in the last 3 months; were allergic to the active substances in either of the nicotine replacement products; history of illicit drug use, major depressive illness or other psychiatric conditions, peripheral arterial occlusive disease (PAOD), COPD, renal impairment (eGFR < 45 mL/min), uncontrolled hypertension (BP ≥ 165/95 mmHg), or CVD.  Female 43.7%; mean age 44.2; mean CPD 15; mean FTND 7  E-cigarette use at baseline: not reported  Motivated to quit: participants were willing to quit | |
| Interventions | EC: **Refillable**  EC arm  E-cigarette starter pack contained two commercially available second-generation e-cigarette devices with charging devices, 11 replacement atomisers and 12 x 10 bottles of nicotine containing e-liquid, tobacco-flavoured e-liquid, 18 mg/mL nicotine. Each e-cigarette device consisted of a 1300 mAh variable voltage rechargeable battery, a tank and an atomiser, and the charging device comprised of USB e-cigarette charger and USB mains adaptor (SmokeMax; Groove Trading Ltd, 194 Glasgow UK), and written instructions.  At baseline, oral and written information was given on how to operate the e-cigarette, and for the duration of the study, participants were asked only to use the study e-cigarette and e-liquid they were provided with.  NRT arm: NRT nicotine replacement patches, 12-week reducing nicotine regimen (21 mg, 14 mg, 7 mg) of Nicotinell® Patches  Weekly supply of nicotine replacement patches. If required, participants were also permitted to use additional other licensed nicotine replacement products (gum, lozenges, nasal spray, inhalers, and micro-tabs), in combination with the nicotine replacement patches.  Both arms: all participants received 12 weeks of behavioural support provided by NHSGGC Smokefree Community Services. Following the baseline visit, participants were asked to define their “quit date”. | |
| Outcomes | Baseline and 12 weeks  CO confirmed smoking cessation  Secondary outcomes: cardiovascular function (heart rate SBP, DBP), lung function, weight | |
| Study funding | Grant from British Heart Foundation (Centre of Research Excellence Award, reference number RE/13/5/30177) | |
| Author declarations | There were no conflicts of interest with the tobacco industry. | |
| Notes | New to 2022 update | |
| ***Risk of bias*** | | |
| **Bias** | **Authors' judgement** | **Support for judgement** |
| Random sequence generation (selection bias) | Low risk | Quote: "Using a secure website (Sealed Envelope Ltd, 2016), participants were randomised in a 1:1 fashion, to either the e-cigarettes combined with behavioural support or nicotine replacement patch group combined with behavioural support. Using a permuted block design with a computer random number generator, block randomisation with block sizes of 4, 6 and 8 was used to reduce bias and achieve balance in the allocation of participants to treatment arms". |
| Allocation concealment (selection bias) | Low risk | Quote: “Secure website (Sealed Envelope Ltd, 2016) participants randomised in a 1:1 fashion, ……Using a permuted block design with a computer random number generator, etc.” |
| Blinding of participants and personnel (performance bias) All outcomes | Low risk | Not blinded, but as both arms contained active interventions, performance bias judged unlikely |
| Blinding of outcome assessment (detection bias) All outcomes | Low risk | BP, heart rate, and oxygen saturation measured. Abstinence at 12 weeks, self-report was CO validated. |
| Incomplete outcome data (attrition bias) All outcomes | Low risk | 31/55 (56.3%)  EC arm 18/28; NRT arm 14/27 |
| Selective reporting (reporting bias) | Low risk | Reported outcomes ‘per protocol’ |

Kimber 2021

| ***Study characteristics*** | | |
| Methods | Design: RCT  Recruitment: participants were screened via phone interviews  Setting: East London, UK  Study start date: participants recruited between December 2015 and December 2016 | |
| Participants | Total: N = 50 people who smoked combustible cigarettes and were e-cigarette naive. Initial sample (N = 70) attended first session; all analyses were conducted on the N = 50 who returned for their 2nd and 3rd session.  Cig-a-likes: N = 11  Tank18: N = 20  Tank6: N = 19  Inclusion criteria:  Smoke daily ≥ 5 cigarettes, have smoked for ≥ 1 year, not currently using an EC, willing to abstain 1 hr before the start of the session and willing to make a quit attempt  Exclusion criteria:  < 18 years, not fluent in English, pregnancy or breastfeeding, or a known neurobiological or heart condition  64% women; mean age 29.5 (SD 9.31); mean CPD 13.09 (SD 6.66), mean FTND 4.14 (SD 2.45)  Motivated to quit: "Willing to make a quit attempt"  E-cigarette use at baseline: no | |
| Interventions | EC: **cartridge and refillable**  Arm 1: cig-a-like (18 mg/mL)  The ‘Blu’ (n = 13) and ‘TECC Go e-cigarette’ models (n = 11) were used for the Cig-a-like condition, due to issues of leakages with the latter. Non-adjustable battery power output and could be recharged, a supply of spare disposable cartridges were provided.  Arm 2: a tank model containing 18 mg/mL (Tank18)  Arm 3: a tank model containing 6 mg/mL (Tank6)  For both conditions, Tank18 and Tank6 the ‘*Totally Wicked mini curve’* was mounted with a 2 mL capacity tank which housed a standard atomiser of 1.5 ohm resistance. E-liquid ingredients composition and flavours were kept consistent across all conditions using the same ratio of propylene glycol and vegetable glycerin (PG/VG: 50/50) and tobacco flavour.  Intervention. All 3 group participants vaped 20 min ad libitum in 3 separate sessions (baseline, 1 and 2 weeks post-baseline). Ahead of their baseline session, participants were instructed to abstain from smoking for an hour. Rated their craving and withdrawal symptoms (at the beginning and end of the session), before receiving instructions on how to use their EC and to vape ad libitum for 20 mins  Positive and adverse effects were measured at the end of the last puff. All vaping sessions were video-recorded.  At the end of each session, participants were given the EC and were instructed to keep a record of the number of cigarettes smoked at the end of each day until their next and subsequent sessions. Each participant was provided with a weekly supply of either, 60–80 mL of e-liquid in refill bottles for those in the tank conditions, or 15 cartridges for those in the cig-a-like condition at the end of each testing session. The session was repeated the following week, then one week later. Participants were asked to keep the device and encouraged to try and replace as many tobacco cigarettes as they could with the use of their EC. | |
| Outcomes | Baseline, week 1, week 2  CO was measured at baseline, wk 1 and wk 2. Self-reported CPD. Adverse events. Puff duration, puff number, inter-puff intervals (IPI). Cigarette dependence, craving, withdrawal, and subjective effects | |
| Study funding | This work was funded by the University of East London through a PhD studentship award. The funder had no role in the study design, collection, analysis or interpretation of data, writing the manuscript and the decision to submit the manuscript for publication. | |
| Author declarations | CK and KS have no conflicts of interest to declare. LD has provided consultancy for the pharmaceutical industry relating to the development of smoking cessation products. | |
| Notes | New to 2022 update | |
| ***Risk of bias*** | | |
| **Bias** | **Authors' judgement** | **Support for judgement** |
| Random sequence generation (selection bias) | Low risk | Participants were randomly allocated (using SPSS). |
| Allocation concealment (selection bias) | Unclear risk | No detail |
| Blinding of participants and personnel (performance bias) All outcomes | Low risk | Intervention arms received equally intensive interventions. |
| Blinding of outcome assessment (detection bias) All outcomes | Low risk | CO measured |
| Incomplete outcome data (attrition bias) All outcomes | High risk | There was a > 20% difference in FU between arms at 2 weeks. Overall, 50/70 = 71.43%. Cig-a-like 11/24 = 45%. Tank6 19/23 = 82.61%. Tank18 20/23 = 86.96% |
| Selective reporting (reporting bias) | High risk | Reported on AEs but excluded those who had reported AEs (as they did not complete all sessions (due to AEs) |

Klonizakis 2022

| ***Study characteristics*** | | |
| Methods | Design: RCT. Pragmatic, 3-group, randomized, assessor-blinded, single-centre trial  Setting: Centre for Sport and Exercise Science (CSES) of Sheffield Hallam University, UK  Recruitment: from the community in the wider Sheffield area via: i) low-cost newspaper and post-office advertisement, ii) posters in local pharmacies, libraries, mosques, churches, and clubs, iii) social media or search engine advertisement (Facebook, Google ads) iv) notices in newsletters or participation in outreach events of community organizations (such as Sheffield U3A and AGE UK), iv) a study website, and v) out-reach events in local ethnic community centres or places of worship  Study start April 2017. Study end December 2020 | |
| Participants | Actual enrolment: 248 participants  Nicotine EC arm = 84  Non-nicotine arm = EC 82  NRT arm = 82  % female: nicotine EC = 55%; non-nicotine EC = 50%; NRT = 44%  Mean age 44  Mean CPD: nicotine EC = 18 (SD7); non-nicotine EC = 16 (SD7); NRT = 18 (SD7)  Mean FTND: 6 (SD2)  Inclusion criteria: Age > 18 years of either sex; People who smoke (at least 10 cpd for the past year); Willing (by declaration) to attempt to quit smoking by using the NHS services or e-cigarettes.  Exclusion criteria: Inability to walk; Recent (within 6 months) cardiovascular disease event (e.g. stroke, myocardial infarction) or cardiac surgery; Insulin-controlled diabetes mellitus or with co-existing skin conditions, leg ulcers, vasculitis or deep venous occlusion (as these may affect their cardiovascular function); Pregnancy; Requiring major surgery during the course of the study; Contra-indications/unsuitability for NRT; Current daily use of e-cigarettes; Currently undertaking a cessation attempt supported by a smoking cessation clinic.  Motivated to quit: yes | |
| Interventions | - a) Complimentary **e-cigarette equipment** and refills (Tornado V5, Joyetech, Shenzhen, China, Totally Wicked, Blackburn, UK) at allocation stage, together with instructions on the correct usage of e-cigarettes. They will also receive behavioural support for a 3-month period. The nicotine strength of Group A cartridges will be up to 18 mg/mL nicotine strength. Participants, on average, received 20 bottles of 10 mL during the intervention period. Participants could choose ice menthol or tobacco flavour. - b) As a), but with **nicotine-free liquid** - c) Referral to **NHS smoking cessation clinics** and will receive NRT in conjunction with behavioural support. Received money or shopping vouchers (depending on personal preference) as reimbursement for NRT prescription charges   All groups received the same level and type of behavioural support as currently offered as standard by stop-smoking services, in the form of regular face-to-face or telephone appointments as per relevant guidelines, e.g. minimum of 6 support sessions within the 3-month period. | |
| Outcomes | Follow-up: within 3 days of “quit date”, 3 and 6 months past quit date  Outcome measures:   - Macrovascular function (FMD assessment) - Microvascular function - Smoking status at 3 and 6 months, self-reported and biochemically validated by exhaled air measurement of < 10 ppm CO - Change in CVD risk using Q-risk assessment - Health economic effects using EQ-5D-L - Total cholesterol and high density lipoprotein via fingerprick blood sample - Participant experiences' assessment | |
| Study funding | The trial was funded by Heart Research UK under a Translational Research Grant (RG2658). The funder had no role in the study’s design, conduct, and reporting. | |
| Author declarations | The authors declare that they have no competing interests. | |
| Notes | New to 2023 (previously ongoing) | |
| ***Risk of bias*** | | |
| **Bias** | **Authors' judgement** | **Support for judgement** |
| Random sequence generation (selection bias) | Low risk | “Randomized remotely into three groups by an independent statistician using a computer-generated (nQuery Advisor 6.0, Statistical Solutions, Ireland) block randomization stratifed by gender and “pack-years” (number of packs (20 cigarettes per pack) per day times number of years smoked)  Unique trial number for each participant for the study duration” |
| Allocation concealment (selection bias) | Low risk | “Outcome assessors were blinded to group allocation and participants were reminded regularly not to share their group allocation with assessors or those providing behavioural support. The study statistician/health economist was blinded to group allocation.” |
| Blinding of participants and personnel (performance bias) All outcomes | Low risk | Interventions equally intensive |
| Blinding of outcome assessment (detection bias) All outcomes | Low risk | Outcome assessors were blinded to group allocation and participants were reminded regularly not to share their group allocation with assessors or those providing behavioural support. The study statistician/health economist was blinded to group allocation. Those delivering the intervention were only blinded in relation to which e-cigarette group the participants belonged to as the NRT group was receiving support through the stop-smoking service.  All measures biochemically validated or measured |
| Incomplete outcome data (attrition bias) All outcomes | Low risk | Nic EC 66/84 at 6 months  Non-Nic EC 64/82  NRT 72/82 |
| Selective reporting (reporting bias) | Unclear risk | Not all secondary outcomes reported in this paper, but more publications likely to follow |

Kouroutzoglou 2024

| ***Study characteristics*** | | |
| Methods | RCT  Setting: Spiliopouleio Hospital , Cardiology Department Athens, Greece  Recruitment: Visiting Cardiology Unit Jan 2016 to Nov 2022 | |
| Participants | N=57  Aged 41 ± 8years; BMI:33.8±2.4Kg/m2  Inclusion: person with obesity; CC user. | |
| Interventions | Electronic cigarette (EC).  NRT (nicotine patch)  NRT + bupropion (NRT+bupr) | |
| Outcomes | Baseline, 3mths (end of treatment), 6 months  Abstinence at 6 mths. EC use at FU. Blood pressure. Weight. | |
| Study funding | “Funding Acknowledgements: None.” | |
| Author declarations | Not reported. | |
| Notes | New to 2025 update. | |
| ***Risk of bias*** | | |
| **Bias** | **Authors' judgement** | **Support for judgement** |
| Random sequence generation (selection bias) | Unclear risk | ‘Randomised’ no detail provided. |
| Allocation concealment (selection bias) | Unclear risk | Not reported. |
| Blinding of participants and personnel (performance bias) All outcomes | Low risk | Equally intensive |
| Blinding of outcome assessment (detection bias) All outcomes | Unclear risk | Not reported. |
| Incomplete outcome data (attrition bias) All outcomes | Unclear risk | Not reported. |
| Selective reporting (reporting bias) | Unclear risk | No clinical record or full publication. Numbers at FU not reported. |

Kumral 2016

| ***Study characteristics*** | | |
| Methods | Design: prospective randomized clinical trial  Recruitment: all patients admitted to a smoking cessation clinic at the Department of Otorhinolaryngology-Head and Neck Surgery, Okmeydanı Training and Research hospital  Setting: smoking cessation clinic, Turkey  Study start date: March 2013; Study end date: November 2013 | |
| Participants | Total N: 98 but analysis excluded 16 from intervention and 10 from control who did not stop smoking; thus 72 analyzed  N per arm: EC: 58 (42 analyzed); non-EC 40 (30 analyzed)  Inclusion criteria:   - Smoked at least 1 pack of cigarettes a day for at least 5 years   Exclusion criteria:   - History of allergic rhinitis, chronic sinusitis, vasomotor rhinitis, asthma, malignancy, or surgery in upper respiratory tract - Age under 18 years - Use of psychoactive drugs   Inclusion based on specific population characteristics: no  44% women; mean age 36; mean cpd and mean FTND not specified  Motivated to quit: “All patients were willing to quit smoking”.  E-cigarette use at baseline: not specified | |
| Interventions | **EC: Unclear**  **EC arm**: “used EC to quit smoking” – allowed to select brand and flavour, used “medium density” liquid (11 to 12 mg/mL) (no further detail given)  **Non-EC arm**: received cognitive behavioural therapy (no further detail given) | |
| Outcomes | 3 months  Sinonasal outcome test (SNOT-22) via self-administered questionnaire, to evaluate changes in subjective symptoms. Saccharin transit test to evaluate nasal mucociliary clearance (MCC) function, which authors stated is “an important defence mechanism” | |
| Study funding | Not specified | |
| Author declarations | Not specified | |
| ***Risk of bias*** | | |
| **Bias** | **Authors' judgement** | **Support for judgement** |
| Random sequence generation (selection bias) | Unclear risk | Quote: “Patients participating in the study were randomly divided into two groups; EC smokers (group 1) and non-EC smokers (group 2).”  No further detail provided |
| Allocation concealment (selection bias) | Unclear risk | No information given |
| Blinding of participants and personnel (performance bias) All outcomes | High risk | Participants were not blinded. The trial is described as single-blinded and outcome assessors were blinded. No placebo used |
| Blinding of outcome assessment (detection bias) All outcomes | High risk | Self-reported outcome data, participants not blinded and unequal amounts of support between arms |
| Incomplete outcome data (attrition bias) All outcomes | Unclear risk | Dropout rate not clear. Only analyzed people who quit |
| Selective reporting (reporting bias) | Low risk | All expected outcomes reported |

Lee 2018

| ***Study characteristics*** | | |
| Methods | Randomized, parallel-assignment, double-blind pilot trial  Setting: San Francisco Veterans Affairs Medical Center (SFVAMC), USA  Recruitment: veterans awaiting surgery  Recruitment: from VA hospital presenting for surgery  Study start date: August 2015; study end date: May 2016 | |
| Participants | Total N: 30  N per arm: NRT: 10; END: 20  Inclusion criteria: presented to the anaesthesia preoperative clinic for elective surgery 3 or more days before surgery; currently smoked ≥ 2 CPD, having smoked at least once in the last 7 days  Exclusion criteria: exclusively used other forms of tobacco or marijuana only; pregnancy/breastfeeding; unstable cardiac condition; currently using smoking cessation pharmacotherapy; already enrolled in a smoking cessation trial; using EC on a daily basis  Inclusion based on specific population characteristics: patients awaiting elective surgery  10% women; mean age 54; mean cpd 14; mean FTND 3.3  Motivated to quit: not specified  E-cigarette use at baseline: not specified but excluded daily users | |
| Interventions | **EC: Cig-a-like**  Both groups received: i) referral to the California Smokers' Helpline, ii) brief advice lasting less than 2 minutes, iii) a brochure from the ASA about quitting smoking before surgery.  **EC arm**: 6-week supply of NJOY e-cigarettes (disposable, first generation). Instructed to use Bold (4.5%) ad lib for 3 weeks, then Gold (2.4%) ad lib for 2 weeks and then study (0%) ad lib for final week. Number of ECs issued corresponded to baseline cpd, assuming 1 EC = 10 cigarettes. Asked to refrain from the use of all study products at the end of 6 weeks  **NRT arm**: 5-week Nicoderm CQ patches, 1 week placebo patches. Dose based on cpd at baseline: ≥ 10 cpd, 21 mg/day for 3 weeks, 14 mg/day for 1 week, 7 mg/day for 1 week, 0 mg/day for 1 week. < 10 cpd at baseline: 14 mg/day for 3 weeks, 7 mg/day for 2 weeks, 0 mg/day for 1 week | |
| Outcomes | 30 days (phone), 8 weeks (in person), 6 months (phone)  Cessation: 7-day PP at 30 days (not validated), 8 weeks (CO-validated), 6 months (not validated). Smoking cessation for at least 48 hours on day of surgery (CO-validated)  Adverse events and biomarkers:   - Adverse events, side effects, and surgical complications by self-report at 30 days, 8 weeks - At 8 weeks exhaled CO, FEV1, and FVC   Other outcomes measured:   - Attitudes and usage - Salivary cotinine - Smoking reduction | |
| Study funding | “This work was funded by internal UCSF Department of Anesthesia and Perioperative Care funds (San Francisco, California, United States of America) and the UCSF Resource Allocation Program grant, administered by the Helen Diller Family Comprehensive Cancer Center developmental funds from the National Cancer Institute Cancer Center Support Grant (P30 CA 82103-16). E-cigarettes were purchased from NJOY using these funds. NJOY had no involvement in the design, execution, or analysis of the study. The funders had no role in study design, data collection and analysis, decision to publish, or preparation of the manuscript.” | |
| Author declarations | “The authors declare there are no competing interests”. | |
| Notes | 3 NRT participants used EC, 2 EC participants used nicotine patch  Study listed as ongoing study NCT02482233 in the 2016 review update | |
| ***Risk of bias*** | | |
| **Bias** | **Authors' judgement** | **Support for judgement** |
| Random sequence generation (selection bias) | Low risk | Quote: “Randomization was computer-generated, with randomly permuted block sizes of 3 or 6, in a 2:1 ratio using the ralloc program”. |
| Allocation concealment (selection bias) | Low risk | Quote: “Allocation was concealed by consecutively numbered, sealed, opaque envelopes”. |
| Blinding of participants and personnel (performance bias) All outcomes | Low risk | Not blinded but both interventions active with equal amounts of support so performance bias judged unlikely |
| Blinding of outcome assessment (detection bias) All outcomes | Low risk | Self-report only at 6 months and participants not blinded to condition, but similar level of support given to both groups so differential misreport judged unlikely |
| Incomplete outcome data (attrition bias) All outcomes | Low risk | 1 NRT and 1 ENDs loss to follow-up at 6 months |
| Selective reporting (reporting bias) | Low risk | All expected outcomes reported |

Lee 2019

| ***Study characteristics*** | | |
| Methods | Design: randomized controlled trial  Recruitment: recruited from motor company  Setting: motor company, medical office in Korea  Study start date: 5 January 2012; study end date: 31 August 2012 | |
| Participants | Total N: 150  N per arm: EC: 75; NRT: 75  Inclusion criteria: Male; At least 10 cpd in previous year; Smoked for at least 3 years; Motivate to stop smoking entirely or reduce consumption.  Exclusion criteria: Past history of serious clinical disease; Attempted to stop smoking in past 12 months by using NRTs.  Inclusion based on specific population characteristics: no  0% women; mean age 42.3; mean cpd: not reported, 1.01 packs per day; mean FTND 4.05  Motivated to quit: yes, or to reduce  E-cigarette use at baseline: not specified | |
| Interventions | **EC: Refillable**  Both arms received 50 minute education sessions on smoking cessation and the use of smoking-cessation aids, instructed to visit the medical office each month for evaluation and counselling by a health practitioner who was unaffiliated with the study.  **Arm 1:** Participants supplied with eGo-CTM EC (nicotine 0.01 mg/mL) from Ovale in 12-wk supply  **Arm 2:** 2 mg nicotine gum in 12-wk supply | |
| Outcomes | 12, 24 weeks (in person)  Cessation: continuous abstinence from 9 to 24 weeks, exhaled CO < 10 ppm, negative urine cotinine  Adverse events and biomarkers: yes, but just note ‘adverse events’  Other outcomes measured: 7-day PPA, cigarette reduction | |
| Study funding | “none” | |
| Author declarations | “none declared” | |
| Notes | Study listed as ongoing study KCT0001277 in the 2016 review update | |
| ***Risk of bias*** | | |
| **Bias** | **Authors' judgement** | **Support for judgement** |
| Random sequence generation (selection bias) | Low risk | Quote: “computer-generated randomization sequence with a block size of 2” |
| Allocation concealment (selection bias) | Low risk | Quote: “The enrolment and assignment of all subjects were performed by a clinical research coordinator not involved in the study”. |
| Blinding of participants and personnel (performance bias) All outcomes | Low risk | Not blinded but both interventions active with equal amounts of support, so performance bias judged unlikely |
| Blinding of outcome assessment (detection bias) All outcomes | Low risk | Participants not blinded but results biochemically validated |
| Incomplete outcome data (attrition bias) All outcomes | Low risk | 61/75 NRT and 71/75 EC FU at 24 weeks |
| Selective reporting (reporting bias) | Low risk | All prespecified outcomes reported |

Lucchiari 2022

| ***Study characteristics*** | | |
| Methods | Design: randomized, parallel-assignment, double-blind trial  Recruitment: participants enrolled in lung cancer screening programme  Setting: early lung cancer detection programme (Cosmos II) at European Institute of Oncology, Italy  Study start date: September 2014; study end date: January 2016 | |
| Participants | Total N: 210  N per arm: 70 participants per arm  At 12 months: nicotine EC 60; non-nicotine EC 58; support only 60  Inclusion criteria:   - Participants are involved in the COSMOS II study - Participants are 55 years or more and have smoked at least 10 cigarettes a day for the past 10 years - Participants wish to reduce tobacco smoking (motivational score higher than 10) who are not treated at a smoking centre - Signed informed consent   Exclusion criteria:   - Symptomatic cardiovascular disease - Symptomatic severe respiratory disease - Regular psychotropic medication use - Current or past history of alcohol abuse - Use of smokeless tobacco or NRT - Participation in another anti-smoking programme in the current year   Inclusion based on specific population characteristics: 55 years of age or older  37% women; mean age 62.8; mean cpd 19.38; mean FTND 4.37  Motivated to quit: yes  E-cigarette use at baseline: excluded people who smoke who had ever regularly used e-cigarettes for more than 1 week alone or in combination with tobacco cigarettes | |
| Interventions | **EC: Cig-a-like**  Both arms received “low intensity counselling” – phone at week 1, 4, 8, and 12, approx. 10 mins each  **Nicotine EC arm**: e-cigarette kit and 12 x 10 mL liquid cartridges (8 mg/mL nicotine concentration). During the first week, participants could use the e-cigarette ad libitum. At the end of the first week, asked to use only EC for the next 11 weeks.  **Nicotine-free EC (placebo) arm**: nicotine-free EC – same as above but with nicotine-free EC | |
| Outcomes | Months 3, 6, and 12  Cessation: continuous abstinence for previous month, CO ≤ 7 ppm  Adverse events and biomarkers: FOR EC ARMS ONLY:   - Exhaled CO - Leicester Cough Questionnaire (LCQ) - Respiratory symptoms (self-report) - Side effects using checklist   Other outcomes measured:   - Motivational questionnaire - HADS - EC use | |
| Study funding | This study was supported by a grant from Fondazione Umberto Veronesi (FUV). | |
| Author declarations | The authors declared no conflicts of interest. | |
| Notes | Listed as ongoing study Lucchiari 2016 (NCT02422914) in 2016 review; new for 2020 update, relabelled from Lucchiari 2020 to Lucchiari 2022 at 2023 update. | |
| ***Risk of bias*** | | |
| **Bias** | **Authors' judgement** | **Support for judgement** |
| Random sequence generation (selection bias) | Low risk | Quote: “A randomization list using a permuted block design (40 blocks of 6 subjects randomly assigned to 1 of the 3 treatment arms) had been previously prepared by independent personnel.” |
| Allocation concealment (selection bias) | Low risk | Double-blind, active and placebo e-cigarettes labelled by independent personnel; researcher and participants blind |
| Blinding of participants and personnel (performance bias) All outcomes | Low risk | “double blind” for nicotine vs no nicotine EC but limited info given; however, as similar levels of support across arms performance bias judged unlikely |
| Blinding of outcome assessment (detection bias) All outcomes | Low risk | Biochemical validation used |
| Incomplete outcome data (attrition bias) All outcomes | Low risk | Approx. 73% followed up in each group at 6 months, very little difference between groups  At 12 months > 70% FU |
| Selective reporting (reporting bias) | High risk | Papers stated CO collected but data not presented |

Martinez 2021

| ***Study characteristics*** | | |
| Methods | Design: RCT  Recruitment: participants were recruited throughout the USA through Facebook and multimedia advertisements (newspapers, radio, TV, e-cigarette forums, and so on) for a study measuring attitudes and behaviours about cigarettes and e-cigarettes.  Setting: USA  Study start date: 31 March 2015. Study end date 30 June 2019 | |
| Participants | 2896 dual users of nicotine EC and combustible tobacco cigarettes  Assessment only n = 575; generic smoking cessation booklets n = 1154; targeted booklets n = 1167  37% female; mean age 29.9  Mean cpd: 1 to 10, 1663 (57%); 11 to 20, 972 (34%); > 20, 259 (9%). Mean FTND 3.6. E-cigarette use at baseline  Inclusion criteria: age 18 years or older, smoked 1 or more CC per week over the preceding year, used EC 1 or more times per week over the preceding month, not currently enrolled in a face-to-face smoking cessation programme, and able to speak and read English. The original inclusion criteria required daily smoking. Many dual users were skipping smoking on some days. Amended the use frequency criteria for smoking and vaping at 1 or more uses per week. The protocol was amended on 25 September 2016. 652 participants had been recruited up to that date.  Participants were not necessarily seeking treatment or motivated to quit smoking or vaping. | |
| Interventions | EC type: n/a  Assessment only (n = 575)  Generic smoking cessation self-help booklets previously shown to be efficacious in smokers (n = 1154) (an introductory *Stop smoking for good* brochure, 10 x *Stop smoking for good* didactic booklets, and 9 *How I quit smoking* pamphlets)  Booklets specifically targetting dual users (n = 1167 (*If you vape: a guide to quitting smoking*), which included an introductory *If you vape* brochure, a series of 10 x *If you vape: guide to quitting smoking* booklets, and 9 x *My story* pamphlets)  Participants assigned to the GENERIC or eTARGET groups were sent the intervention materials by post, with the option of also receiving them electronically. | |
| Outcomes | Full follow-up assessments: 6, 12, 18, and 24 months. Abbreviated assessments: 3, 9, 15, and 21 months after baseline  7-day PPA at each assessment point. Sustained abstinence: 30-day and 90-day PPA  Breath CO and saliva samples (for cotinine analysis) were collected at the 12- and 24-month follow-up points for participants who reported abstinence and resided within 100 miles of the home institution.  Cut-offs of 8 ppm for CO and 10 ng/mL for cotinine were used to determine abstinence. The disconfirmation rates from this sample were used to estimate adjusted smoking rates for the full sample. | |
| Study funding | This work was supported by the National Institute on Drug Abuse of the NIH (R01DA037961). The content is solely the responsibility of the authors and does not necessarily represent the official views of the NIH.  This work has also been supported in part by the Biostatistics and Bioinformatics Shared Resource and the Participant Research, Interventions, and Measures Resource at the H Lee Moffitt Cancer Center and Research Institute, a National Cancer Institute designated Comprehensive Cancer Center (P30CA76292) | |
| Author declarations | Quote: "THB has received research support from the US National Institutes of Health (NIH), the American Cancer Society, the Florida Department of Health, and Pfizer; has collaborated on funded research with Voxiva, Optum, and the University of East Anglia (Norwich, UK); spent sabbatical at the Trimbos Institute and Utrecht University (Utrecht, Netherlands); is on the advisory board of, and holds restricted stock in, Hava Health, which is developing a pharmaceutical grade electronic nicotine delivery system for smoking cessation; participated in a Best Brains Exchange for Health Canada, providing advice on e-cigarette policy; and consulted for the Australian Government Solicitor regarding plain tobacco packaging. UM has received research support from the NIH and the Galician Plan of Research, Innovation, and Growth (Spain); and has received funding from the Barrie Foundation to receive predoctoral training at the University of Newcastle (Callaghan, NSW, Australia). VNS has received research support from the NIH and the Florida Department of Health. SKS has received research support from the NIH, the American Cancer Society, the Florida Department of Health, and Pfizer. DJD has received research support from the NIH, the American Cancer Society, and the Florida Department of Health, and has provided paid expert testimony in litigation against tobacco companies. MMB has received funding from the NIH, the Florida Department of Health, the US Department of Veterans Affairs, the US Centers for Disease Control and Prevention, the National Science Foundation, and the US Department of Housing & Urban Development; and has received research support from Gilead Sciences, Florida Blue Foundation, Bristol Myers Squibb Foundation, Merck Foundation, Maine Cancer Foundation, and Pfizer. PTH has received research support from the NIH, US Food and Drug Administration (FDA), and Virginia Foundation for Healthy Youth. TE conducts research supported by the National Institute on Drug Abuse of the NIH and the Center for Tobacco Products of the FDA; is a paid consultant in litigation against the tobacco industry and the electronic cigarette industry; is named on one patent for a device that measures the puffing behaviour of electronic cigarette users and on another patent for a smartphone app that determines electronic cigarette device and liquid characteristics; owns shares in a variety of mutual funds, the exact stock makeup of which he has no control, and owns shares in three publicly traded companies, none of which are in any way related to the tobacco industry, the electronic cigarette industry, or any other aspect of this work; and has served as a special government employee of the US Government in the context of his service on the FDA’s Tobacco Products Scientific Advisory Committee and the Department of Health and Human Services Secretary’s Advisory Committee on Human Research Protection. CRB has received research support from the New Zealand Ministry of Health, the Health Research Council of New Zealand, CureKids Foundation, Heart Foundation, Health Promotion Agency, and Auckland Council and Sanitarium; collaborates on funded research with Newcastle University (Australia) through a grant from the Australian National Health and Medical Research Council, with Zhejiang University (Hangzhou, China) and Kunming University (Yunnan, China) on an Education New Zealand Tripartite grant, and with the University of Malaya (Kuala Lumpur, Malaysia) on a University of Malaya Grand Challenges grant; received funding from Pfizer Australasia for a survey of the impact of COVID-19 on health workers in low-income and middle-income countries and from Johnson & Johnson Japan for consultancy on smoking cessation medication; and was a consultant to Moffit Cancer Center on this study through an NIH grant. LRM and KOB declare no competing interests. The employees of Moffitt Cancer Center—UM, VNS, SKS, DJD, LRM, KOB, MMB, and THB—are eligible for sharing of any revenue that might be generated by products developed during their employment, including the intervention used in this study." | |
| Notes | Quote: "Participants were compensated US$10–20 for the first eight assessments and $40 for the final one, and they were eligible for $40–60 bonuses for completing at least seven assessments. Participants returning assessments within 1 week were sent inexpensive appreciation gifts."  Appendix: "Participants were not aware in advance of the interview that they would be asked for biosamples, and new informed consent was obtained at that time. Participants received $20 for completing a biochemical verification interview and $15 for providing biosamples." | |
| ***Risk of bias*** | | |
| **Bias** | **Authors' judgement** | **Support for judgement** |
| Random sequence generation (selection bias) | Low risk | Quote: "We used computer generated randomisation with balanced permuted blocks (block size 10, with 2-4-4 ratio) to allocate participants to assessment only (ASSESS group), generic smoking cessation self-help booklets (GENERIC group), or booklets targeting dual users (eTARGET group)." |
| Allocation concealment (selection bias) | Low risk | Computer-generated (see above) |
| Blinding of participants and personnel (performance bias) All outcomes | Low risk | Tailored versus generic booklet judged low risk as similar intensity; this is the comparison used in the meta-analysis.  Tailored versus no support would be high risk due to differential levels of support provided and no blinding. |
| Blinding of outcome assessment (detection bias) All outcomes | Low risk | As above, tailored versus generic similar intensity so judged to be low risk of differential misreport (self-reported cessation only) |
| Incomplete outcome data (attrition bias) All outcomes | Low risk | All > 50% at 24 months  ASSESS: 361/575\*100 = 62.8%  GENERIC: 619/1154\*100 = 53.6%  eTARGET: 642/1167\*100 = 55% |
| Selective reporting (reporting bias) | Low risk | All prespecified outcomes reported in the trial register reported in the publication |

Martner 2019

| ***Study characteristics*** | | |
| Methods | Design: a non-concurrent multiple baseline across participants design. Three phases were included: baseline, EC, and EC + CM. Half the participants received the EC phase following baseline; the other half received EC + CM following baseline.  Recruitment: community  Setting: set-up meetings occurred at the University of Florida Behavioral Health and Technology Research Clinic, USA  Study start date/study end date: not specified | |
| Participants | Total N: 12  Inclusion criteria: 18 to 65 years old; smoked ≥ 2 years; smoked ≥ 8 cpd on average; smoked in the past 24 hours; expressed a desire to quit smoking; reliable access to the internet and a computer or smartphone; breath CO ≥ 10 ppm at set-up  Exclusion criteria: current or previous medical condition that would pose an increased risk to participation; use of benzodiazepines, cocaine, or opiates in the previous 6 months; smoking marijuana more than twice a month; exposed to elevated CO levels (e.g. spouse smokes in house); pregnant or expected to become pregnant in the next 6 months  58.3% women; mean age 37.5; mean cpd 16.25; mean FTND 5  Motivated to quit: expressed a desire to quit smoking  E-cigarette use at baseline: 3 participants never tried an EC prior to the study; 2 owned an EC but quit using it more than a month prior to the study; the remaining 7 had tried an EC more than a year prior to the study but never owned one | |
| Interventions | **EC: Refillable**  All participants provided with smokio electronic cigarettes (second-generation ECs) and V2 e-liquid with a concentration of 24 mg/mL (2.4%) of nicotine. Researchers provided participants with a copy of the National Cancer Institute’s brochure *Clearing the Air* (smokefree. gov). Then researchers and participants read through a manual that described the study procedures, and showed participants how to use the software to measure CO and how to use the EC.  Participants initially received EC without contingency for a period of 14 days following the quit attempt. If participants failed to reduce CO levels during this phase, they received contingency management in addition to EC. | |
| Outcomes | 4 weeks  Adverse events and biomarkers: adverse events collected in 4-day smoking behaviour questionnaires; eCO  Other outcomes measured: acceptability and use of EC; overall experience of study | |
| Study funding | "The study was supported in part by crowd-sourced funding enabled by Experiment.com. Preparation of this paper was supported in part by Grant P30DA029926." | |
| Author declarations | "The authors declare no conflicts of interest." | |
| Notes | N of 1 (within-participants randomized design, not between groups). New for 2020 update | |
| ***Risk of bias*** | | |
| **Bias** | **Authors' judgement** | **Support for judgement** |
| Random sequence generation (selection bias) | High risk | Not randomized |
| Allocation concealment (selection bias) | High risk | Not randomized |
| Incomplete outcome data (attrition bias) All outcomes | Unclear risk | No details provided. |
| Selective reporting (reporting bias) | Unclear risk | AEs measured in behavioural change questionnaire but not reported |

McRobbie 2015

| ***Study characteristics*** | | |
| Methods | Design: prospective cohort  Recruitment: advertisements in free London newspapers  Setting: smokers' clinic, East London, UK  Study start date: February 2013; study end date: September 2013 | |
| Participants | Total N: 40  Inclusion criteria: people who smoke daily who want to quit; aged 18 and older.  Exclusion criteria: pregnant and breastfeeding women; current serious medical illness; EC use for more than 1 week in the pastc  45% women, mean age 47 (SD 12), mean cpd 19 (SD 10), mean FTND 5.2 (SD 2.8), 65% in full-time employment  Motivated to quit: yes  E-cigarette use at baseline: excluded those who had used EC for more than 1 week in the past | |
| Interventions | **EC: Cig-a-like**  Participants attended a baseline session 1 week prior to their TQD. On the TQD, participants were provided with an EC (Green Smoke, 1st generation device, 2.4% nicotine cartridges). 2 cartridges a day were supplied initially, with the supply adjusted to actual use later. Attended 4 weekly follow-up sessions and received standard behavioural support. | |
| Outcomes | Cigarette consumption and CO readings collected at each session. Urine sample for cotinine and 3-HPMA analysis collected at baseline and 4 weeks post-TQD.  Change in urinary 3-HPMA (ng/mg creatinine) at 4 weeks  Change in urinary cotinine (ng/mg creatinine) at 4 weeks  Change in CO at 4 weeks | |
| Study funding | "This study was funded by a grant given to P. Hajek, H. McRobbie, and M.L.Goniewicz from the UK Medicines and Healthcare Products Regulatory Agency (MHRA). The costs of publication of this article were defrayed in part by the payment of page charges. This article must therefore be hereby marked advertisement in accordance with 18 U.S.C. Section 1734 solely to indicate this fact." | |
| Author declarations | "H. McRobbie is Clinical Director at The Dragon Institute; reports receiving commercial research grant from Pfizer; and has received speakers bureau honoraria from Johnson&Johnson and Pfizer. M.L. Goniewicz reports receiving a commercial research grant from Pfizer. P. Hajek has received speakers bureau honoraria from and is a consultant/advisory board member for the manufacturers of stop-smoking medications. No potential conflicts of interest were disclosed by the other authors." | |
| ***Risk of bias*** | | |
| **Bias** | **Authors' judgement** | **Support for judgement** |
| Random sequence generation (selection bias) | High risk | Prospective cohort |
| Allocation concealment (selection bias) | High risk | Not randomized |
| Incomplete outcome data (attrition bias) All outcomes | Low risk | 7/40 participants were lost to follow-up. |
| Selective reporting (reporting bias) | Low risk | All predefined outcomes reported |

Meier 2017

| ***Study characteristics*** | | |
| Methods | Design: randomized, cross-over trial (e-cig vs placebo)  Recruitment: via local media outlets  Setting: community, USA  Study start date/study end date: not specified | |
| Participants | Total N: 24  Inclusion criteria:   - ≥ 18+ years - People who smoke daily (≥ 10 cpd) - Not interested in quitting in the next 30 days - English-speaking - Interested in using EC   Exclusion criteria:   - Using cessation meds - Use of ECs in last 6 m - Exhaled CO < 6 ppm - History of CV trauma or uncontrolled hypertension - Pregnant   Inclusion based on specific population characteristics: no  25% women; mean age 48.5; mean cpd 16.3; FTND not reported  Motivated to quit: no (eligibility criteria was to not want to quit in next 30 days)  E-cigarette use at baseline: 8/24 (33%) had previously tried an EC, average 9.4 months since last use, average length of use 3.6 days | |
| Interventions | **EC: Cig-a-like**   Smoked “as usual” for 1 week followed by 2 weeks of either placebo or active 1st generation EC BluCig starter kit with up to 7 cartridges (prefilled, with either active 16 mg or 0 mg nicotine solution)  Participants were instructed “this e-cig may or may not contain nicotine; we ask that you try it at least once, but use it however you like; smoke regular cigarettes as you wish.” Shown how to charge the device and sampled the product during the visit. Provided a handout on how to use the product (e.g. switching cartridges) and general information about ECs | |
| Outcomes | 1 week in each condition, in person  Adverse events and biomarkers:   - Adverse events, not clear how collected - Exhaled CO   Other outcomes measured:   - Vaping - Regular smoking - Perceived reward from ECs - Intentions/confidence to quit - Cotinine - Withdrawal symptoms | |
| Study funding | “…supported by grants P01 CA138389, P30 CA138313 (Hollings Cancer Center Support Grant) from the National Cancer Institute of the National Institutes of Health and UL1 TR000062 from the National Center for Advancing Translational Science of the National Institutes of Health. BWH was supported by K12DA031794”. | |
| Author declarations | “KMC has received grant funding from Pfizer, Inc., to study the impact of a hospital-based tobacco cessation intervention. He also receives funding as an expert witness in litigation filed against the tobacco industry. We have no other declarations of interests to declare”. | |
| Notes | New for 2020 update | |
| ***Risk of bias*** | | |
| **Bias** | **Authors' judgement** | **Support for judgement** |
| Random sequence generation (selection bias) | Unclear risk | Quote: “Participants were randomized to receive either an active or placebo EC first”; no further information provided |
| Allocation concealment (selection bias) | Unclear risk | Refer to 'random sequence generation'. |
| Blinding of participants and personnel (performance bias) All outcomes | Low risk | Quote: “Participants and research staff conducting sessions were blinded to dose. All cartridges were pre-loaded by the manufacturer. Labeling was removed by a research team member not involved in participant contact to mask placebo versus active ECs. We restricted flavour options to regular tobacco flavour or menthol to most closely match usual cigarette brand flavour profile and reduce unwanted variance in product”. |
| Blinding of outcome assessment (detection bias) All outcomes | Low risk | Quote: “Participants and research staff conducting sessions were blinded to dose. All cartridges were pre-loaded by the manufacturer. Labeling was removed by a research team member not involved in participant contact to mask placebo versus active ECs. We restricted flavour options to regular tobacco flavour or menthol to most closely match usual cigarette brand flavour profile and reduce unwanted variance in product”. |
| Incomplete outcome data (attrition bias) All outcomes | Unclear risk | Not specified |
| Selective reporting (reporting bias) | Low risk | All expected outcomes reported |

Morphett 2022a

| ***Study characteristics*** | | |
| Methods | Design: RCT  Setting: Australia  Recruitment: commercial market research panel and supplemental recruitment by study researchers. An invitation to participate was available to 46 857 I-view panel members, including those with no smoking status recorded. In addition, advertisements to the general public were used to recruit additional participants.  Study start date: 2014 | |
| Participants | Total: N 1712 (table 1 full sample 1562)  Arm A [Standard cessation advice & NRT (NRT short-term)] flow diagram 368 (324 received intervention)  Arm B [Quit or substitute advice and NRT: advice to use NRT as a longer-term substitute for smoking if required to maintain smoking cessation] flow diagram 671 (620 received intervention)  Arm C [Quit or substitute advice and NRT and/or e-cigarettes] flow diagram 673 (619 received intervention)  Inclusion criteria:  Current daily smoking (at least 6 cpd). Agree to try samples of nicotine products. 18+ years  Exclusion criteria:  Currently being treated for serious cardiovascular disease, cancer, taking regular medication for mental health condition, uncontrolled high blood pressure, stomach ulcer, kidney or liver disease, overactive thyroid or adrenal gland cancer. Use insulin for diabetes. Asthma or chronic throat disease. Pregnant or planning to become pregnant/breastfeeding  Female 64%; mean age 46.7 (SD 12.3); mean CPD 18.2 (SD8.7)  E-cigarette use at baseline: 28% had previously tried EC  Motivated to quit: yes; 58% want to quit a lot | |
| Interventions | EC type: **cartridge**  Arm C only: disposable e-cigarette available in two strengths (free-base nicotine 3.0% and 4.5%). A rechargeable version of the same brand with replaceable cartridges (3.0% v/v and 4.5% v/v) was substituted when the disposables were discontinued by the manufacturer (September 2014). The e-cigarettes contained only nicotine, vegetable glycerin, and water, and were unflavoured.  a) Arm A (usual care smoking cessation practice in Australia) comprising quit with NRT. Factsheet explaining the relative harm of NRT compared to smoking, free sample of NRT, participant chooses preferences, has the intervention free for 3 weeks then offered at a subsidized rate for further 6 months. The NRT products included nicotine gum, lozenges, inhalator, and mouth spray. Lozenges and gum were offered at two strengths.  b) Arm B as (a), but with additional information provided: advice to quit or substitute with NRT  c) Arm C as (a), but additional information on electronic cigarettes and emphasis on cessation, and may select electronic cigarettes as well as NRT | |
| Outcomes | Baseline, 7 months and 12 months, self-report   - Continuous abstinence - Self-reported 7-day point prevalence abstinence - NRT and EC use - Interest in quitting smoking and in quitting NRT - Cigarette consumption - Product orders and use - Quit attempts - AEs | |
| Study funding | Funding was from the National Health and Medical Research Council, Australia (#GNT1020123). The e-cigarettes supplied in this trial were Vype brand, and were purchased from the manufacturer Nicoventures Trading Ltd., a UK-based company that was a division of British American Tobacco. The other nicotine products were purchased in Australia from various distributors. Participant recruitment and survey data collection was managed by I-View Social Research. None of these entities had any role in study design, data collection, data analysis, data interpretation, or writing of this paper. | |
| Author declarations | No authors have received financial support for the submitted work from any companies with a financial interest in the products under investigation. C.B. has undertaken consultancy for J&J Japan, a manufacturer of nicotine replacement therapy. N.W. and C.B. have completed a smoking cessation trial in which cytisine was supplied by Achieve Life Sciences, and a smoking cessation trial in which varenicline and matching placebo were supplied by Pfizer under their investigator-initiated research programme. N.W. and C.B. have previously undertaken two trials of e-cigarettes for smoking cessation (with e-cigarettes purchased from a NZ e-cigarette online retailer [NZVAPOR, https://www.nzvapor.com/], e-liquid for one trial purchased from NicoPharm, Australia and nicotine patches supplied by the NZ government via their contract with Novartis [Sydney, Australia]). Neither NZVAPOR nor NicoPharm have links with the tobacco industry. None of the authors' spouses, partners, or children have financial relationships that may be relevant to the submitted work. All authors have no non-financial interests that may be relevant to the submitted work. | |
| Notes | New to 2022 update | |
| ***Risk of bias*** | | |
| **Bias** | **Authors' judgement** | **Support for judgement** |
| Random sequence generation (selection bias) | Low risk | Quote: "After completing the baseline survey, participants were block randomized in a 1:2:2 ratio to one of the three conditions by a computer generated random number sequence". |
| Allocation concealment (selection bias) | Low risk | Quote: "Participants and researchers were not blind to the allocated treatment; however, participants were not advised that there were different treatment conditions". |
| Blinding of participants and personnel (performance bias) All outcomes | Low risk | Quote: "Participants and researchers were not blind to the allocated treatment; however, participants were not advised that there were different treatment conditions". |
| Blinding of outcome assessment (detection bias) All outcomes | High risk | Self-report |
| Incomplete outcome data (attrition bias) All outcomes | Low risk | Quote: "72.5% of participants who received the allocated intervention in Condition A completed the 7-month survey, compared with 74.4% of Condition B and 72.9% of Condition C".  At 12 months: arm A 233/324; arm B 457/671; arm C 448/673 |
| Selective reporting (reporting bias) | Low risk | Specified outcomes reported |

Morphett 2022b

| ***Study characteristics*** | | |
| Methods | Design: RCT, pragmatic, randomized, partial cross-over  Setting: Australia  Recruitment: not stated  Study start date: recruited in 2018-2019 | |
| Participants | Total: N 355  Arm A: 181  Arm B: 174  Inclusion criteria:  Diagnosed with/treatment for HIV or hepatitis C (HCV) or receiving opioid substitution therapy (OST). Diagnosed with or receiving treatment for priority health conditions in the past 12 months. Referral to Quitline counselling and smoking cessation support programme (standard care) but has not begun quit attempt. 18+ years; currently smoke 10+ cigarettes per day; willing to make a quit attempt  Exclusion criteria:  Already started quit attempt (i.e. post-quit day) or enrolled in another smoking cessation clinical trial or using varenicline or bupropion or used a nicotine vaporizer product in the last 30 days. Health reason (e.g. CVD, terminal illness, recent hospitalization for mental health reason, pregnancy) | |
| Interventions | EC type: **refillable**  **Arm 1)** Referral to Quitline telephone smoking cessation counselling + **Nicotine patches** (15 mg/16-hr) delivered at baseline + **refillable nicotine vaporizer device** (2 x kits) + nicotine vaporising liquid (in high and low strength - high strength: nicotine 1.8%; low strength: nicotine 0.6%). 1 patch to be applied daily to skin for up to 84 days. The vaporizer with nicotine liquid is to be used as needed, up to 3.5 mL per day to treat withdrawal symptoms for up to 2 years (concurrently with patches for the first 84 days) to assist smoking cessation and relapse prevention. Participants start on high-strength nicotine liquid and may decrease their dose to low strength to assist with dose reduction prior to stopping use of the vaporizer.  **Arm 2)** Referral to Quitline telephone smoking cessation counselling + **Nicotine patches** (15 mg/16-hr) + participant’s choice of either **nicotine gum or nicotine lozenges** (up to 800 x 4 mg pieces to be used up to 8 per day) delivered at baseline. Between 6 and 9 months post-baseline - participants in Arm 2 who are smoking (either failed to quit or relapsed) will be offered: refillable nicotine vaporizer (2 x kits) + nicotine vaporizing liquid (in high and low strength - high strength: nicotine 1.8%; low strength: nicotine 0.6%) to make a second quit attempt. Participants will have until 2 years from baseline to use the vaporizer for smoking cessation and relapse prevention.  Arm B participants who were smoking at 6 months were offered the NVP intervention (NVPs as second-line therapy). Switched over to EC intervention, called Arm C | |
| Outcomes | Baseline 6 months, 12 months, 24 months  Primary outcomes:  Continuous abstinence from smoking from weeks 12 to 26 assessed at 26 weeks from baseline by self-report (bio-confirmed)  Secondary outcomes:  Continuous abstinence from smoking  AEs at 12 weeks and 26 weeks  Abstinence is assessed through study-specific survey questions in Module CS. Combustible Smoking Questions – urine specimens will be batch-tested for anabasine and cotinine at 6-, 12-, and 21-month time points. | |
| Study funding | National Health and Medical Research Council | |
| Author declarations | Not reported | |
| Notes | Abstract only  New to 2022 update | |
| ***Risk of bias*** | | |
| **Bias** | **Authors' judgement** | **Support for judgement** |
| Random sequence generation (selection bias) | Unclear risk | No detail |
| Allocation concealment (selection bias) | Unclear risk | No detail |
| Blinding of participants and personnel (performance bias) All outcomes | Low risk | Intervention arms both received interventions. |
| Blinding of outcome assessment (detection bias) All outcomes | Low risk | Participants that self-report abstinence from smoking will be asked for a urine specimen for bio-confirmation. |
| Incomplete outcome data (attrition bias) All outcomes | Unclear risk | No detail in conference abstract |
| Selective reporting (reporting bias) | Unclear risk | Conference abstract. Outcomes may be reported more fully at a later date. |

Morris 2022\*

| ***Study characteristics*** | | |
| Methods | Design: randomized, cross-over, open-label, 2-part study  Recruitment: study participants were recruited from areas surrounding the study sites (Celerion, Lincoln, NE; Frontage, Secaucus, NJ) using standard advertising methods and were compensated for their participation in the study.  Setting: 2 clinical research centres USA (Celerion, Lincoln, NE; Frontage, Secaucus, NJ). Participants were confined to the respective clinics for the full duration of the study.  Study start date: November 2019. Study end date: January 2020 | |
| Participants | Total N: 79 (single-arm)  Inclusion criteria:  Smoking an average of > 10 manufactured combustible cigarettes pd for at least 12 months  Exclusion criteria:  Relevant illness history; presence of clinically significant mental or physical health conditions; high blood pressure; acute illnesses (e.g. upper respiratory infection, viral infection); relevant medication use; use of prescription smoking cessation treatments, anti-diabetic or insulin drugs or medications known to interact with Cytochrome P450 2A6; body mass index (BMI) > 40 kg/m2 or < 18 kg/m2; allergy to propylene glycol or glycerin; planning to quit smoking during the study; pregnancy/breastfeeding; urine screen for alcohol or drugs of use  Participants were between 21 and 65 years. CPD 'at least 10' cpd. Percentage women not reported  Motivated to quit: no  E-cigarette use at baseline: no | |
| Interventions | EC: **Pod**  Myblu™ two-piece closed system comprised of a rechargeable 350 mAh battery and disposable pod containing an e-liquid  Sixteen commercial disposable liquid pod variants; different flavours; 5 different strengths (12, 24, 25, 36, 40 mg/mL); 2 forms of nicotine: nicotine salt or free-base nicotine  The e-liquid mixtures consisted of VG, PG, nicotine, and a proprietary blend of favours; pods contained 1.5 mL of e-liquid, equating to approximately 200 puffs under standardized machine puffing conditions.  The ENDS were charged and assembled for the participants and product information sheets provided. On each study day, fresh pods and a fully charged device were provided. All participants received training from clinic staff on how to operate their ENDS and to ensure compliance in the clinic; all participants used their products under the supervision of suitably qualified staff. | |
| Outcomes | Baseline (-2 day), day 9, day 14  15 biomarkers of exposure (BoE) to selected harmful and potentially harmful constituents (HPHCs) associated with tobacco smoking  AEs | |
| Study funding | This work was funded entirely by Fontem US LLC, a subsidiary of Imperial Brands PLC. | |
| Author declarations | This work was funded by Fontem US LLC, a subsidiary of Imperial Brands PLC, and manufacturers of the myblu™ products used in this study. This work was done by Imperial Brands PLC on behalf of Fontem US LLC as a service provider. Work was contracted to Celerion, who conducted the study and analysed the data. At the time of the study and/or writing, PM, SM, FC, TV, XC, MS, JT, NC and GOC were employees of Imperial Brands PLC. | |
| Notes | Part 1 study data used only  New to 2022 update | |
| ***Risk of bias*** | | |
| **Bias** | **Authors' judgement** | **Support for judgement** |
| Random sequence generation (selection bias) | High risk | Study randomized, though for our analyses we treat as single-arm. Quote from protocol: “Subjects who complete the study screening assessments and meet all the eligibility criteria and are randomized will be assigned a unique randomization identification number on day 1 for part 1 and on day 10 for part 2, and will receive study products according to the randomization scheme generated by Celerion”. |
| Allocation concealment (selection bias) | Unclear risk | No detail |
| Incomplete outcome data (attrition bias) All outcomes | Low risk | Quote: “Seventy-two subjects completed the study and met the conditions for inclusion in the data analysis (out of a total of 79 recruited).” |
| Selective reporting (reporting bias) | High risk | FEV1 and FVC clinical trial registry outcomes not included in paper |

Myers-Smith 2022

| ***Study characteristics*** | | |
| Methods | Design: RCT  Recruitment: Clients of Queen Mary University of London's community stop-smoking service who did not manage to stop smoking with routine treatment were invited to take part. Also recruited eligible smokers seeking help with quitting via social media.  Setting: Queen Mary University of London, community stop-smoking service  Study start date: April 2017 to August 2018 | |
| Participants | Total: N = 135  N per arm: E-cigarette 68; NRT 67  Inclusion criteria:  History of failed quit attempts using stop-smoking medications and/or stop-smoking services. Willing to use their allocated harm-reduction strategy for at least 4 weeks. 18+ years  Exclusion criteria:  Currently using EC or any stop-smoking products. Strong preference to use or not to use NRT or EC. Pregnancy or breastfeeding  Women: 49%; mean age 40; median CPD 15 (IQR 10); median FTND: 5 EC arm, 4 NRT arm; motivated to quit  E-cigarette use at baseline: percentage tried EC earlier: 31% EC arm, 49% NRT arm | |
| Interventions | EC: **Refillable**  EC Arm. EC starter pack and instructions to purchase further e-liquids of flavour and strength of their choice (voucher for up to £40). Participants paid for further supplies themselves. They were encouraged to try e-liquids of different strengths and flavours if the initial purchase did not meet their needs. Up to 8 weeks supply. Minimal behaviour support  NRT Arm: NRT of choice. The choice of products included nicotine patch, chewing gum, nasal spray, microtab, inhalator, and mouth spray. Up to 8 weeks supply. At the baseline visit, participants selected an NRT product or product combination.  Minimal behavioural support  Both groups: 2 face-to-face sessions (baseline and week 1). Baseline: Participants selected products of their choice and received instructions on how to obtain them. Week 1: Smokers bring allocated product to the session, receive advice on use, test and start product use. Commitment to not using unallocated products for the next 4 weeks. Those wishing to stop smoking altogether were asked to set a target quit date (TQD). Participants received phone calls 1 and 4 weeks later to monitor product use and smoking status and to provide brief support. | |
| Outcomes | Baseline (week 0), week 1, week 4, week 24  Primary outcome measure:  Cigarette consumption per day, assessed by self-report in the follow-up survey created for the purpose of the study at 1, 4, and 24 weeks post-quit date/preparation date. Those who report ≥ 50% smoking reduction will be validated with a CO reading in the clinic.  Secondary outcome measures:  1. Use of allocated harm reduction strategies. 2. Strategy ratings. 3. Changes in smoking behaviour. 4. Proportion of people still using allocated strategy at 6 months. All measured by the follow-up survey created for the purpose of the study at 1, 4, and 24 weeks post-quit date/preparation date. AEs | |
| Study funding | The study was funded by a Tobacco Advisory Group project grant, Cancer Research UK (C6815/A20503). | |
| Author declarations | PH and HM have received research funding from and provided consultancy to Pfizer, a manufacturer of stop-smoking medications. DP has received research funding from Pfizer. All other authors had no conflicts to declare. | |
| Notes | Participants invited for CO readings at 4 weeks and 6 months received £10 in compensation for their time and travel at both visits.  New to 2022 update | |
| ***Risk of bias*** | | |
| **Bias** | **Authors' judgement** | **Support for judgement** |
| Random sequence generation (selection bias) | Low risk | Quote: “Randomisation sequences (1:1 ratio in permuted blocks of 20) were produced by an independent statistician using computer generated randomisation codes.“ |
| Allocation concealment (selection bias) | Low risk | Quote: “Codes were sealed in opaque envelopes and marked with a unique randomisation number. Study staff allocated randomisation numbers sequentially. Staff opened the next envelope and entered the allocation onto the clinical record form (CRF) and randomisation log.” |
| Blinding of participants and personnel (performance bias) All outcomes | Low risk | Quote: “Data analysis was completed blind by an independent statistician.”  Both arms active intervention |
| Blinding of outcome assessment (detection bias) All outcomes | Low risk | Quote: “Data analysis was completed blind by an independent statistician.” |
| Incomplete outcome data (attrition bias) All outcomes | Low risk | Quote: “Follow-up rates were 85% and 88% at 4 weeks and 88% and 70% at 6 months in the EC and NRT group, respectively.” |
| Selective reporting (reporting bias) | Low risk | Outcomes registered were all reported. |

NCT02648178

| ***Study characteristics*** | | |
| Methods | Setting: medical centre, USA  Recruitment: people with cancer  Design: non-randomized, single-group assignment trial  Recruitment: clinical settings, including outpatient clinics and the infusion suite  Study start date: June 2016; study end date: May 2018 | |
| Participants | Total N: 19  Inclusion criteria: histological or cytological diagnosis of aerodigestive tract cancers or bladder cancer within the past 5 years (≥ 1 tobacco-related malignancy is allowed); AJCC stages I-IV; daily smoking (≥ 10 CPD for 10 years) and breath CO2 ≥ 8 ppm; does not wish to quit smoking now (anyone wishing to quit smoking will be referred for smoking cessation counselling through the WRJ VAMC or DHMC programme); may be receiving anti-cancer agents; age ≥ 18+ years  Exclusion criteria: cancer surgery or radiation planned in the next 9 weeks; actively trying to quit smoking, or planning to in the next 30 days; any EC use in the past 30 days; pregnant or trying to get pregnant  Inclusion based on specific population characteristics: patients with stage I-IV aerodigestive tract cancers or bladder cancer who smoke daily  42.1% women; mean age: not reported -categories 18 to 65 years: N = 9, > 65 years: N = 10; cpd and FTND: not reported  Motivated to quit: no (inclusion criterion)  E-cigarette use at baseline: not specified but EC use within 30 days is an exclusion criterion | |
| Interventions | **EC: Cig-a-like and refillable**  Instructed on the use of EC, and given a supply that is "approximately equivalent to their current nicotine intake". Given Halo Triton EC (leak-proof refillable tank system) or Halo G6 leak-proof prefilled cartomizers. Began participants with 18 mg/mL and moved nicotine content up or down based on participant preference. Choice of flavours, provided for 9 weeks | |
| Outcomes | Weeks 3, 6, 9, 12. Self-report at clinic visits  Adverse events and biomarkers:   - Adverse events assessed with a checklist for commonly occurring side effects from e-cigarettes and nicotine products - Exhaled carbon dioxide - Expired carbon monoxide - Urine propylene glycol - Urine 4- (methylnitrosamino)-1-(-3pyridyl)-1butanol (NNAL) 40 and 1- hydroxy naphthalene (1-HOP)   Other outcomes measured:   - Timeline Follow-Back Questionnaire (TLFB) - EC appeal assessed with attitudinal ratings, on a 5-point Likert-type scale - e-cigarette ease of use, satisfaction, and enjoyment, and willingness to continue to purchase e-cigarettes in the future - Change in daily cigarette smoking given 10 or more E-cig sessions - Average number of E-cigs used per day - The co-ordinators will conduct and audiorecord a 10- to 15-minute qualitative interview at 9 weeks soliciting perceptions about e-cigarettes to be transcribed and analyzed for common themes that could be useful in developing the larger intervention. - Urine nicotine and cotinine | |
| Study funding | Sponsors Dartmouth-Hitchcock Medical Center | |
| Author declarations | Not reported – data extracted from clinical trial registry record | |
| Notes | Study listed as ongoing study in the 2016 review update | |
| ***Risk of bias*** | | |
| **Bias** | **Authors' judgement** | **Support for judgement** |
| Random sequence generation (selection bias) | High risk | Not randomized, single-group assignment |
| Allocation concealment (selection bias) | High risk | Not randomized, single-group assignment |
| Incomplete outcome data (attrition bias) All outcomes | Low risk | 19 enrolled; 10 participants followed up at 12 weeks |
| Selective reporting (reporting bias) | Unclear risk | The following measures were not reported: exhaled carbon dioxide; urine propylene glycol; urine nicotine, cotinine, NNAL and 1- hydroxy naphthalene (1-HOP), and Timeline Follow-Back Questionnaire (TLFB). Data at 6, 12 months also not reported |

NCT02918630

| ***Study characteristics*** | | |
| Methods | Design: RCT  Recruitment: clinics  Setting: SMI clinics, USA  Study start date: October 2016; study end date: August 2017 | |
| Participants | Total N: 7  N per arm: NRT: 4; EC + NRT 3  Inclusion criteria: diagnosed with schizophrenia (or other SMI, not clear); be in stable medical condition (DSM-V); report smoking ≥ 10 tobacco cigarettes/day; breath CO ≥ 10 ppm; report wanting to reduce their cigarette smoking; stable living situation  Exclusion criteria: pregnant or breastfeeding; report wanting to quit smoking in the immediate future; test positive for illicit drugs except THC; any illness, medical condition, or use of medications, which in the opinion of the study physicians would preclude safe or successful completion of the study, or both  Inclusion based on specific population characteristics: yes - SMI (schizophrenia and schizoaffective disorder, bipolar disorder, or PTSD)  43% women; mean age 48.3; mean cpd: NR; mean FTND: NR  Motivated to quit: Wanted to quit or reduce their cigarette smoking but did not want to quit in the immediate future (this was an exclusion criterion). NB – trial registry stated wanted to reduce and protocol stated wanted to quit or reduce as inclusion criteria.  E-cigarette use at baseline: not specified | |
| Interventions | **EC: Refillable**  Both arms received a nicotine patch 21 mg for 4 weeks.  **EC + NRT**: 4 weeks: 1) a 3.3 V, 1000 mAh battery; and 2) a 1.5 Ohm, dual-coil cartomizer (SmokTech; Shenzhen, China). Nicotine concentrations 36 mg/mL. Verbal and written instructions on how to use and maintain the e-cigarettes at week 1 visit. Nicotine patch (21 mg)  **NRT arm:** NRT only (nicotine patch, 21 mg) | |
| Outcomes | 5 weeks  Cessation: n/a but “change in smoking”  Adverse events and biomarkers:  Breath CO, COPD-related symptoms, EC side effects (e-cig side effects questionnaire), AEs, SAEs  Other outcomes measured:  Urinary cotinine, cpd, tobacco dependence, craving, withdrawal symptoms, desire to quit, confidence to quit, EC dependence, EC use, satisfaction with EC, nicotine dependence, schizophrenia symptoms (brief psychiatric rating scale), cognitive domains associated with schizophrenia (MATRICS consensus cognitive battery), changes in positive symptoms of schizophrenia (scale for the assessment of positive symptoms), changes in negative schizophrenia symptoms (scale for the assessment of negative symptoms), suicide ideation (Columbia Suicide Severity Rating Scale) | |
| Study funding | Sponsors: The University of Texas Health Science Center, Houston | |
| Author declarations | Not reported | |
| Notes | New for 2020 update. Information from clinical.trials.gov registry and unpublished protocol; discrepancies between the two in terms of trial methods. Feasibility for future NIH grant application. Intended to recruit 20 participants, but only 7 started and completed. | |
| ***Risk of bias*** | | |
| **Bias** | **Authors' judgement** | **Support for judgement** |
| Random sequence generation (selection bias) | Unclear risk | Not reported |
| Allocation concealment (selection bias) | Unclear risk | Not reported |
| Blinding of participants and personnel (performance bias) All outcomes | Unclear risk | “double-blind” but “open-label” elsewhere, no further info given |
| Blinding of outcome assessment (detection bias) All outcomes | Unclear risk | Not reported |
| Incomplete outcome data (attrition bias) All outcomes | Unclear risk | Not reported |
| Selective reporting (reporting bias) | High risk | Schizophrenia and COPD outcomes not reported |
| Other bias | Unclear risk | Some discrepancies between clinical trials record and protocol linked to from record, including when NRT started and inclusion criteria (just schizophrenia or all SMI). Target sample size was 20 but only 7 people recruited |

NCT03113136

| ***Study characteristics*** | | |
| Methods | RCT, randomized interventional clinical trial  Setting: Ohio State University Comprehensive Cancer Center, USA  Study start date: June 2017. Study completion: January 2022 | |
| Participants | Total N: 372  Usual brand CC = 105; Low wattage EC = 133; High wattage EC = 134  62.6% female; mean age 40.33 (11.04 SD); mean CPD 17.28 (8.93 SD)  Inclusion criteria: no quit attempt in the prior 3 months and no plan to quit in the next 3 months; smoked ≥ 5 cigarettes per day for the past year; minimal interest in switching to an alternative product; never purchased or regularly used a tank system, mechanical mod, or advanced personal vaporizer EC, though previous use of cig-a-like devices will be allowed  Exclusion criteria: unstable or significant medical condition such as respiratory, kidney, or liver disease; unstable or significant psychiatric conditions; history of cardiac event or distress within the past 3 months; and pregnant/breastfeeding; < 18 years.  Motivated to quit: no  EC use at baseline: no | |
| Interventions | 1. Low-wattage e-cigarette device, provided for 12 weeks. Instructed to vape ad libitum for 12 weeks. Assessed at 6 months and 12 months for continued use of device.  2. High-wattage e-cigarette device, provided for 12 weeks. Instructed to vape ad libitum for 12 weeks. Assessed at 6 months and 12 months for continued use of device.  3. Usual brand cigarette, provided free for 12 weeks, instructed to smoke ad libitum for the duration of the study. | |
| Outcomes | Baseline, week 1, week 4, week 8, week 12, week 26, and week 52  Complete change from conventional cigarettes.  Exhaled carbon monoxide of ≤ 10 ppm.  AEs, SAEs.  Oral mucosa samples collected 0, 4, and 12 weeks. Damage was quantified by q-PADDA  EC dependence; EC preference; biomarkers of exposure NNAL; NNN; TNE; nicotine metabolite ratio; nickel and other relevant metals; cadmium and other relevant metals; lead and other relevant metals; 8-iso-PGF2a; PGEM; q-PADDA | |
| Study funding | NIH/NCI (R01CA204891, Wagener; R01CA242168, Queimado. | |
| Author declarations | Not reported | |
| Notes | Moved to new study in 2025 update. Added as ongoing study in 2022 update | |
| ***Risk of bias*** | | |
| **Bias** | **Authors' judgement** | **Support for judgement** |
| Random sequence generation (selection bias) | Low risk | “Randomization will occur using a stratified block-randomization procedure with small, random-sized blocks." |
| Allocation concealment (selection bias) | Unclear risk | No detail |
| Blinding of participants and personnel (performance bias) All outcomes | Low risk | 2 EC arms equally intensive. |
| Blinding of outcome assessment (detection bias) All outcomes | Low risk | Biochemically validated complete switch from CC to EC. |
| Incomplete outcome data (attrition bias) All outcomes | High risk | High at 12 months: UC = 78/105 = 74.3%; Low wattage EC = 67/133 =50.4%; High wattage EC = 65/134 = 47.0%. [Low at week 26: CC 77/105 = 73%; Low wattage EC: 69/133 = 52%; High wattage EC: 73/134 = 54.5%.] |
| Selective reporting (reporting bias) | Unclear risk | Some data available on NCT record / abstract other outcome data may be reported later. |

Nides 2014\*

| ***Study characteristics*** | | |
| Methods | Design: open-label, non-comparative study  Recruitment: study site database and community advertisements  Setting: Clinical Trials Unit, USA  Study start date: April 2013; study end date: 10 July 2013 | |
| Participants | Total N: 29  Inclusion criteria: age 18 to 65 years; good health; BMI 18 to 35; smoking 10+ cpd; CO > 10 ppm  Exclusion criteria: pregnancy or breastfeeding; other drug dependency; use of any psychiatric or opioid medications; EC within the previous 14 days; use of NRT in last 30 days' want to reduce or quit smoking within the next 30 days  Exclusion criterion: EC within the previous 14 days; use of NRT in last 30 days  44% women; mean age 43; mean cpd 20.1; mean FTND 4.5  Motivated to quit: no  E-cigarette use at baseline | |
| Interventions | **EC: Cig-a-like**  Participants attended 3 clinic visits at 1-week intervals  Visit 1: baseline  Visit 2: Provided with 1st generation type - 'NJOY® King Bold' (NJOY, Inc. Scottsdale, AZ), with 26 mg nicotine. Used ad libitum for 20 minutes in the clinic, then ad libitum use over the next week. Recorded use of regular cigarettes and puffs on EC  Visit 3: Participants abstained from all sources of nicotine for 12 hours prior to visit. | |
| Outcomes | Adverse events | |
| Study funding | Funding for this study was provided by NJOY, Inc., Scottsdale, AZ. | |
| Author declarations | Dr Nides has received compensation from NJOY, Inc. and GlaxoSmithKline. Dr Leischow has received compensation from GlaxoSmithKline, Pfizer, and Cypress Bioscience. Mr Simmons and Ms Bhatter have no conflict of interest to report. | |
| ***Risk of bias*** | | |
| **Bias** | **Authors' judgement** | **Support for judgement** |
| Random sequence generation (selection bias) | High risk | Prospective cohort |
| Allocation concealment (selection bias) | High risk | Not randomized |
| Incomplete outcome data (attrition bias) All outcomes | Low risk | 2 participants dropped out between visits 1 and 2. |
| Selective reporting (reporting bias) | Low risk | Planned comparisons reported |

Okuyemi 2022

| ***Study characteristics*** | | |
| Methods | Design: RCT  Recruitment: The study identified African-American current cigarette smokers using the electronic health record in the University of Minnesota Fairview Health system. The authors sent recruitment letters that provided a brief description of the study and invited recipients who were interested in the study to contact study staff. Participants were also invited to refer contacts outside their family to the study.  Setting: Study visits were conducted in the Delaware Clinical Research Unit (DCRU) through the Clinical and Translational Science Institute (CTSI) at the University of Minnesota, USA.  Study start date: June 2018. Study end date September 2019 (NCT record: Start date: 15 November 2016. End date: 8 March 2019) | |
| Participants | Total N: 234  Nicotine EC arm: 118  Non-nicotine EC arm: 116  Inclusion criteria:  1) Self-identification as African-American or black, 2) smoked ≥ 5 cigarettes daily for the past year, smoking status confirmed by expired CO ≥ 5 ppm or positive NicAlert screen, 3) willingness to use EC, 4) 18 to 79 years  Exclusion criteria:  1) Recent unstable or untreated psychiatric diagnosis including substance abuse (DSM-IV criteria), 2) EC use in the past 30 days, 3) planning to quit smoking in the next 30 days, 4) pregnancy or nursing, 5) CO < 5 ppm and no cotinine detected in the urine  Female: 43.9%; mean age 50.8 (SD 11.2); mean CPD 11.5 (SD 6.0)  Motivated to quit: no  E-cigarette use at baseline: no | |
| Interventions | EC: **Refillable**  EC with 24 mg of nicotine added. Nicotine EC rechargeable Halo G6 brand 2.4% nicotine (24 mg, equivalent to the nicotine content of combustible cigarettes)  EC - No-nicotine EC rechargeable Halo G6 brand 0% nicotine (0 mg)  For both groups:  A free Halo G6 brand rechargeable EC starter kit with the accessories including the charger, batteries, and a 2-week supply of liquid cartridges. The Halo G6 device was 3.3 to 4.2 (average 3.7) volts. The prefilled cartomizers coil resistance was 2.2 to 2.8 ohms. At the week-2 visit, participants received an additional 4-week supply of cartridges.  Participants were given oral and written instructions about how to use the products.  Ad lib for 6 weeks. All participants were provided with EC by the study; menthol and non-menthol flavoured EC cartridges were available. Participants could purchase their own if needed after 6 weeks.  Participants were compensated for their time and transportation: USD 40 at baseline, USD 40 at week 2, USD 50 at week 6, and USD 20 at week 12, for a maximum of USD 150 over 12 weeks. | |
| Outcomes | Baseline, 2, 6, and 12 weeks (all visits were in-person except week 12, which was a telephone survey)  Biomarkers at baseline and 6 weeks. Urinary biomarkers (NNAL, NNK) and total nicotine equivalents (TNE, total nicotine + total cotinine + total 3-hydroxycotinine + nicotine N-oxide). Expired carbon monoxide (CO)  Combustible cigarettes self-reported number of cigarettes smoked per day (and baseline). EC use (Penn State EC Dependence Index)  EC dependence (10-item Penn State EC Dependence Index)  Nicotine withdrawal symptoms (baseline, week 2, 6, 12) (modified Minnesota Nicotine Withdrawal (MNWS))  Data collection was self-administered and collected on electronic tablets. | |
| Study funding | ClearWay Minnesota Grant Award #RC-2014-0009 | |
| Author declarations | The authors declared that they had no competing interests. | |
| Notes | New to 2022 update | |
| ***Risk of bias*** | | |
| **Bias** | **Authors' judgement** | **Support for judgement** |
| Random sequence generation (selection bias) | Unclear risk | Quote: "To ensure balance in the number of intervention assignments between the study groups, randomization was blocked (block size unknown to staff or investigators) by nicotine versus no nicotine e-cigarettes." No other information given |
| Allocation concealment (selection bias) | Unclear risk | No information given |
| Blinding of participants and personnel (performance bias) All outcomes | Low risk | Single-blind. Interventions equally intensive |
| Blinding of outcome assessment (detection bias) All outcomes | Low risk | Biomarkers measured |
| Incomplete outcome data (attrition bias) All outcomes | Low risk | Nicotine EC arm: 109/118  Non-nicotine EC arm: 106/116 |
| Selective reporting (reporting bias) | Low risk | Reported outcomes in NCT record |

Oncken 2015

| ***Study characteristics*** | | |
| Methods | Design: randomized, cross-over study  Recruitment: newspaper advertisements, radio announcements, and from local general medicine practices  Setting: lab-based study, Connecticut, USA  Study start date: October 2012; study end date: June 2015 | |
| Participants | Total N: 27  Inclusion criteria: non-treatment-seeking people who smoke who were willing to try EC for 2 weeks and abstain from conventional cigarette smoking; 18 to 55 years of age who smoked at least 10 cpd  Exclusion criteria: pregnant; previous myocardial infarction or stroke; uncontrolled hypertension (blood pressure (BP) > 160/100); insulin-dependent diabetes; COPD or current asthma; known allergy to propylene glycol.  45% women; mean age 42; 70% white; 15% Hispanic, 15% black; mean cpd 16; 45% had tried EC at baseline, 50% smoked menthol cigarettes  Motivated to quit: no  E-cigarette use at baseline: not specified | |
| Interventions | **EC: Cig-a-like**  Prescribed Joye eGo-C (www.joyetech.com) and e-Juice (18 mg/mL nicotine) procured from American eLiquid (www.americanliquid.com) Cross-over study between menthol-flavoured and non-menthol tobacco-flavoured EC. Requested not to smoke their regular cigarettes during study period, but most (60%) reported intermittently smoking cigarettes during study | |
| Outcomes | Follow-up at 1 week and 2 weeks  BP, heart rate, body plethysmography, static lung volumes and airways resistance (Raw), and specific conductance (sGaw) – taken at lab visits after abstaining from EC for at least 2 hrs, then taken again after inhaling EC and repeated 5 mins later  Adverse events also reported but method for measuring not stated  Also measured nicotine concentrations, rates of cigarette and EC use | |
| Study funding | This project was supported by Academic Enhancement funds from the Department of Medicine at the University of Connecticut Health Center (to CO) and the Clinical Research Center at the University of Connecticut Health Center. | |
| Author declarations | CO is currently receiving study medication (nicotine inhaler and placebo) from Pfizer pharmaceuticals for an NIH-funded nicotine inhaler for smoking cessation during pregnancy. | |
| ***Risk of bias*** | | |
| **Bias** | **Authors' judgement** | **Support for judgement** |
| Random sequence generation (selection bias) | Unclear risk | Method not stated  Quote: "Subjects were then randomly assigned to use the menthol or plain e-cigarette cartridge for one week, switching to the other cartridge for the second week". |
| Allocation concealment (selection bias) | Unclear risk | No detail given |
| Blinding of participants and personnel (performance bias) All outcomes | Low risk | No detail given on blinding but equal levels of support between arms, so performance bias judged unlikely |
| Blinding of outcome assessment (detection bias) All outcomes | Low risk | Some subjective outcomes but equal levels of support between arms, so differential misreport judged unlikely |
| Incomplete outcome data (attrition bias) All outcomes | Low risk | 20/27 followed up |
| Selective reporting (reporting bias) | Unclear risk | Unable to determine prespecified outcomes |

Ozga-Hess 2019

| ***Study characteristics*** | | |
| Methods | Design: RCT  Recruitment: Cigarette smokers were recruited from the community via fliers, online postings, and word of mouth.  Setting: Morgantown, West Virginia, USA  Study start date: not reported. Study end date: not reported | |
| Participants | Total N: 60; E-cigarette plus own brand = 30. Own brand cigarette (control) = 30  38.3% female; mean age completers 35.1 (SD 11) (N = 34), non-completers 36.8 (SD 12.9) (N = 26); mean cpd completers 16.7 (SD 4.9), non-completers 19.6 (SD 6.1); mean FTND completers 5.3 (SD 1.8), non-completers 5.9 (SD 1.9)  Inclusion criteria: 18 to 60 years; smoking ≥ 10 cigarettes per day for ≥ 1 year; exhaled air carbon monoxide (CO) level of ≥ 10 ppm (Micro+™ basic monitor; CoVita; Haddonfield, NJ); contemplation or Preparation Stage of Change (indicating interest in a quit attempt within the next 1 to 6 months)  Exclusion criteria: reported chronic health or psychiatric conditions; past month use of marijuana ≥ 5 days; past month use of any other illicit drugs, or regular use of ECIGs or other tobacco products (i.e. ≥ 1 day per week); individuals in the Precontemplation (no interest in quitting) or Action (actively trying to quit) Stage of Change; pregnancy/breastfeeding | |
| Interventions | **EC: Refillable**  **E-cigarette** (18 mg/mL) plus own brand cigarette. Kanger mini Protank-II, which is a 1.5 mL Pyrex glass tank with a drip tip and atomizer head coils (KangerTech; China), and a 3.3 V constant output, 900 mAh, eGo-T battery (Joyetech; Irvine, CA). The liquid (The Vapor Room, Sky Vapors LLC, Frostburg, MD) was labelled as 70% propylene glycol and 30% vegetable glycerin, with a nicotine concentration requested of 18 mg/mL. Participants could choose tobacco, menthol or wild berry flavour and could switch between sessions. Ad libitum use for 4 weeks  **Own brand cigarette** ad libitum use for 4 weeks | |
| Outcomes | Assessments days 8, 15, 22, and 29. FU 1 month post intervention.  Daily for salivary cotinine samples. Daily self-monitoring device to log e-cigarette and cigarette use. Collected used cigarette filters  Weekly CO breath test  Attended the laboratory weekly for assessments (days 8, 15, 22, and 29). Then completed a follow-up visit 1-month post-intervention  Self-reported withdrawal symptoms  Reported experience of specific symptoms rated using a visual analogue scale with a range from 0 (not at all) to 100 (extremely). e.g. craving, irritability, dry mouth, throat irritation, and cough | |
| Study funding | Financial support provided to MDB and GAD by WVU Senate Grant for Research, and to GAD, MDB, and NAT by Cooperative Agreement Number 1-U48-DP-005004 from the Centers for Disease Control and Prevention (CDC) to the West Virginia Prevention Research Center. Support provided to NJF and JEOH by the National Institute of General Medical Sciences (NIGMS T32 GM081741). Additional support provided by WV Tobacco Cessation QuitLine | |
| Author declarations | Author SGF has consulted for various pharmaceutical companies on matters relating to smoking cessation. All other authors declared that they had no conflicts of interest. | |
| ***Risk of bias*** | | |
| **Bias** | **Authors' judgement** | **Support for judgement** |
| Random sequence generation (selection bias) | Unclear risk | Quote: "Using a simple randomized design"  Comment: not adequately explained |
| Allocation concealment (selection bias) | Unclear risk | Not adequately described in the paper |
| Blinding of participants and personnel (performance bias) All outcomes | High risk | Participants and investigators were not blind. |
| Blinding of outcome assessment (detection bias) All outcomes | Low risk | Biochemical validation |
| Incomplete outcome data (attrition bias) All outcomes | High risk | 40% retention, but no difference between groups |
| Selective reporting (reporting bias) | Low risk | All outcomes reported |

Pacifici 2015

| ***Study characteristics*** | | |
| Methods | Design: uncontrolled pre-post pilot study  Recruitment: word of mouth  Setting: hospital-based smoking cessation clinic, Italy  Study start date/end date: not specified | |
| Participants | Total N: 34  Inclusion criteria: adults who smoke, unwilling to quit smoking tobacco cigarettes and who have never tried a quit-smoking protocol or have refused any smoking cessation treatment, or both  Exclusion criteria: none stated  Inclusion based on specific population characteristics: no  47.1% women, mean age 40.6, mean cpd 21.5  No EC use at baseline, not motivated to quit | |
| Interventions | **EC: Refillable**  Participants were given commercially available EC (AVATAR device, Battery 550 mAh/3.9 V, W: 7.8, cartomizer with 2, 2 ohm resistance, tank capacity 1.5 mL, temperature of the aerosol: 55/65 degrees), 2 different chargers for each EC and PUFFIT e-liquids with nicotine content matching the individual nicotine daily intake and tobacco and/or other flavours freely chosen by each participant.  W1: nicotine-free e-liquid  W2&3: Own EC with personal nicotine dosage, encouraged to use as substitute for traditional cigarettes  W4: Encouraged to forego all traditional cigarettes  Throughout: assistance at any time of day from centre staff with any EC-related problem, plus follow-up group sessions and smartphone messaging application  Behavioural support:  Multi-component medically assisted training programme with monitoring of nicotine intake as a biomarker of correct EC use, including information about general working principles, safety and risks of EC, together with medically assisted face-to-face training on how to correctly use the device to absorb nicotine vapour | |
| Outcomes | Follow-up at 1, 4, and 8 m  Cessation (measure not defined)  Adverse events  Exhaled CO, COT, 3-HCOT concentration  cpd | |
| Study funding | The authors thank Renata Solimini, Adele Minutillo, Emilia Marchei and Maria Concetta Rotolo for their technical assistance. This work was supported by the Department of Therapeutic Research and Medicines Evaluation Istituto Superiore di Sanità, Roma, Italy. | |
| Author declarations | The authors declared no conflict of interest. | |
| ***Risk of bias*** | | |
| **Bias** | **Authors' judgement** | **Support for judgement** |
| Random sequence generation (selection bias) | High risk | Not controlled |
| Allocation concealment (selection bias) | High risk | Not controlled |
| Incomplete outcome data (attrition bias) All outcomes | Low risk | All participants followed up |
| Selective reporting (reporting bias) | High risk | AEs measured but not reported |

Pericot-Valverde 2025

| ***Study characteristics*** | | |
| Methods | Single arm pilot study  Setting: Patient clinic providing maintenance treatment for OUD, Prisma Health Campus in Greenville, South Carolina, USA.  Recruitment: Recruited via medical staff referral, word of mouth, and flyers posted in the Outpatient addiction clinic, approached before or after their routine clinical visits with their providers while waiting in designated waiting areas. | |
| Participants | N= 30  50% female; mean age 44.5 (SD 10.4); mean CPD 16.2 (SD 8.3); mean FTND 5.4 (SD2.5).  Inclusion criteria: 1) age 21 or older, (2) being a current CC smoker, (3) smoking ≥5 CPD for ≥1 year, (4) OUD diagnosis and currently receiving buprenorphine, (5) interested in switching to EC, (6) plan to remain on buprenorphine for the duration of the study, and (7) have a smartphone with internet access.  Exclusion criteria: (1) used other tobacco or nicotine products, including EC, on ≥9 days in the past 30 days; (2) had used smoking cessation medication in the prior 30 days; (3) were pregnant or breastfeeding; (4) had a current medical or psychiatric condition that would contraindicate participation; or (5) were unable to speak/read English.  Inclusion based on specific populationcharacteristic: CC smokers with opioid use disorder (OUD) on buprenorphine treatment.  EC use at baseline: Not specified. Inclusion criteria 'interested in switching to EC' indicates not using EC at baseline. Exclusion using ECs for ≥9 daysdays in past 30 days  Motivated to quit: Not specified. Exclusion criteria 'used quit smoking medication in past 30 days' indicates not motivated to quit. | |
| Interventions | Single EC arm  EC plus 4-week supply of e-liquid liquid equivalent to 150% of their daily smoking rate. This amount was calculated based on self-reported daily smoking rate at baseline  RPM 4 EC device (SMOK, China), 5 mL refillable pod (dimensions 103 × 25 × 27.9 mm, 102 g, 1650 mAH, 3.3–4.2 V) and adjustable power settings ranging from 5 to 60 W. The EC was supplied with both 0.23 and 0.4 Ω coils, enabling participants to choose their preferred option. Participants were provided with additional pods and coils at no cost in the event of a malfunction. The EC liquid used was a freebase nicotine so lution (6 mg/mL) containing 70% propylene glycol and 30% vegetable glycerin.  Option to select EC liquid flavour: sweet-cooling flavor; sweet non-cooling flavor tobacco flavor; and menthol-flavored.  Brief training on use of the study EC, how to complete the daily diary surveys, and participate in the brief weekly video calls. | |
| Outcomes | Baseline, 1 week, 4 weeks, 8 weeks  CO  EC liquid flavour choice. | |
| Study funding | This project was supported in part by the Clemson Faculty SUCCEEDS (CU SUCCEEDS) funding program, the Creative Inquiry (CI) program at Clemson University, and the Prisma Health Addiction Medicine Center | |
| Author declarations | None declared. | |
| ***Risk of bias*** | | |
| **Bias** | **Authors' judgement** | **Support for judgement** |
| Random sequence generation (selection bias) | High risk | Single arm, not randomised |
| Blinding of outcome assessment (detection bias) All outcomes | Low risk | Biological samples included saliva-based toxicology screens and CO samples (iCO Smokelyzer, Bedfont Scientific Ltd), which were collected at baseline, during each remote weekly visit, and at the in-person visits for weeks 4 and 8). |
| Incomplete outcome data (attrition bias) All outcomes | Low risk | “On average 74.1% (2.9/4) of remote weekly visits were completed, and 80% (24/30) and 76.6% (23/30) of participants completed the week 4 and week 8 visits, respectively. Most of the CO (76.2% or 160/210) and drug screen (78.6% or 165/210) assessments were completed, with 23.3% (7/30) of participants lost to follow-up at week 8. On average, 17.4 (SD = 8.8) or 61.4% (SD = 31.4) of daily diary assessments were completed with 70% (21/30) responding to at least half and 40% (12/30) to ≥75%.” |
| Selective reporting (reporting bias) | Unclear risk | NCT record does not include CO.  CO measurement was ad-hoc secondary analysis. |

Piper 2025

| ***Study characteristics*** | | |
| Methods | Design: RCT  Setting: USA. University of Wisconsin Center for Tobacco Research and Intervention  Recruitment: participants from the greater Madison and Milwaukee, Wisconsin areas will be recruited via media recruitment methods (i.e. television, newspaper, and earned media) that have recruited thousands of smokers. Investigators will also use Internet/Facebook advertisements.  Study start date September 2019. Study end date May 2023 | |
| Participants | Randomised 209 (160 completed both switch weeks)  Number analyzed:  Juul EC + active and placebo patch 54  VLNC + active and placebo patch 53  No product + active and placebo patch 53  60% female; 21.9% African-American; 51.9 (SD 12.1) age; 16.8 (SD 9.3) CPD   Inclusion criteria: Smoking > 4 cigarettes/day for the previous 6 months, (CO) > 6 ppm; No plans to quit smoking in the next 30 days; Not currently taking smoking cessation medication; Willing and medically able to use nicotine patches;21+ years.  Exclusion criteria: Currently in treatment for psychosis or bipolar disorder; EC use within the last month; Pregnant or breastfeeding.  Motivated to quit: no | |
| Interventions | EC: pod  Juul Electronic cigarette. The Juul e-cigarette pods contain 0.7 mL nicotine by volume/5% nicotine by weight. Tobacco or menthol flavour  Very Low Nicotine Cigarettes: These very low nicotine cigarettes (VLNCs) consist of reduced nicotine cigarettes containing 0.03 mg of nicotine; these VLNCs were obtained from the National Institute on Drug Abuse (NIDA's) Drug Supply Program (NOT-DA-14-004).  Active nicotine patches, with dosing based on the package insert (> 10 cigs/day = 21 mg patch and < 11 cigs/day = 14 mg patches)  Placebo patch containing no nicotine  Arm 1: Active comparator: Juul + active patch in wk 1 and placebo patch in wk 2  Participants will be given Juul e-cigarettes for 4 weeks; in switch week 1, participants will also use active nicotine patches; in switch week 2, participants will use placebo patches.  Arm 2: Active comparator: Juul + placebo patch in wk 1 and active patch in wk 2  Participants will be given Juul e-cigarettes for 4 weeks; in switch week 1, participants will also use placebo patches; in switch week 2, participants will use active nicotine patches.  Arm 3: Active comparator: VLNC + active patch in wk 1 and placebo patch in wk 2  Participants will be given very low nicotine cigarettes (VLNCs) for 4 weeks; in switch week 1, participants will also use active nicotine patches; in switch week 2, participants will use placebo patches.  Arm 4: Active comparator: VLNC + placebo patch in wk 1 and active patch in wk 2  Participants will be given very low nicotine cigarettes (VLNCs) for 4 weeks; in switch week 1, participants will also use placebo patches; in switch week 2, participants will use active nicotine patches.  Arm 5: No product + active patch in wk 1 and placebo patch in wk 2  Participants will be given no alternative nicotine delivery products, but in switch week 1, participants will use active nicotine patches; in switch week 2, participants will use placebo patches.  Arm 6: No product + placebo patch in wk 1 and active patch in wk 2  Participants will be given no alternative nicotine delivery products for 2 weeks, but in switch week 1, participants will use placebo patches; in switch week 2, participants will use active nicotine patches. | |
| Outcomes | Weeks 1 to 4  Weeks 1 through 4, participants will use a smartphone to record, in the moment, each time they use their own cigarettes or any alternative product.  Primary outcome: number of conventional cigarettes smoked during each switch week  Secondary outcome: number of VLNCs or Juul pods used during each switch week  SAE, AE | |
| Study funding | Sponsors and Collaborators University of Wisconsin, Madison  National Cancer Institute (NCI) | |
| Author declarations | Not available; abstract and trial registry only | |
| Notes | New to 2023 (previously ongoing) as adverse event data now included in trial registry. However, distribution of AEs across randomised groups is unclear and so data is thus far only included in narrative synthesis and Supplementary Materials. When further information is available, these will be moved to meta-analyses where appropriate. | |
| ***Risk of bias*** | | |
| **Bias** | **Authors' judgement** | **Support for judgement** |
| Random sequence generation (selection bias) | Low risk | Electronic randomization using trial database |
| Allocation concealment (selection bias) | Unclear risk | Not described |
| Blinding of participants and personnel (performance bias) All outcomes | High risk | No placebo e-cigarette |
| Blinding of outcome assessment (detection bias) All outcomes | High risk | Different intensities of interventions. AEs self-reported |
| Incomplete outcome data (attrition bias) All outcomes | Low risk | 160/209, 76.5% |
| Selective reporting (reporting bias) | Low risk | Results posted on NCT record |

Polosa 2011\*

| ***Study characteristics*** | | |
| Methods | Design: prospective cohort  Recruitment: advertisements in local hospital in Catania, Italy  Setting: not specified  Study start date: February 2010; study end date: June 2010 | |
| Participants | Total N: 40, hospital staff  Inclusion criteria: healthy people who smoke; 18 to 60 years; smoking ≥ 15 cpd for ≥ past 10 years, and not wanting to quit smoking at any time in the next 30 days  Exclusion criteria: history of alcohol and illicit drug use; psychiatric illness; recent myocardial infarction; angina pectoris; high blood pressure (BP > 140 mmHg systolic or 90 mmHg diastolic, or both); diabetes mellitus; severe allergies; poorly-controlled asthma or other airways diseases  35% women, mean age 42.9 (SD 8.8), median cpd 25 (IQR 20 to 30), median FTND 6.0 (IQR 6 to 8)  Motivated to quit: no  E-cigarette use at baseline: not specified | |
| Interventions | **EC: Cig-a-like**  Seen at baseline, given EC ('Categoria' brand) with an initial 4-week supply of 7.4 mg nicotine cartridges. Instructed to use ad libitum up to 4 cartridges per day. EC cartridges supplied at months 1, 2, and 3  No instruction on cessation or reduction was provided. | |
| Outcomes | Follow-up at 1, 2, 3, 6, 18, and 24 months when cigarette consumption, CO, and AEs were measured, incl. 30-day PP CO-validated abstinence at 6 months and CO-validated abstinence at 18 and 24 months (not otherwise defined)  Adverse events | |
| Study funding | "We wish to thank Arbi Group Srl (Milano, Italy) for the free supplies of ‘Categoria’ e-Cigarette kits and nicotine cartridges as well as their support. We would also like to thank the study participants for all their time and effort and LIAF (Lega Italiana AntiFumo) for the collaboration." | |
| Author declarations | "None of the authors have any competing interests to declare, but RP has received lecture fees from Pfizer and, from Feb 2011, he has been serving as a consultant for Arbi Group Srl.Arbi Group Srl (Milano, Italy), the manufacturer of the e-Cigarette supplied the product, and unrestricted technical and customer support. They were not involved in the study design, running of the study or analysis and presentation of the data." | |
| Notes | Smoking cessation services provided to those who spontaneously asked for assistance with quitting. These participants were excluded from the study protocol. | |
| ***Risk of bias*** | | |
| **Bias** | **Authors' judgement** | **Support for judgement** |
| Random sequence generation (selection bias) | High risk | Prospective cohort |
| Allocation concealment (selection bias) | High risk | Not randomized |
| Incomplete outcome data (attrition bias) All outcomes | Low risk | 13/40 were lost to follow-up, but used ITT analysis |
| Selective reporting (reporting bias) | Unclear risk | Unable to determine prespecified outcomes |

Polosa 2014b\*

| ***Study characteristics*** | | |
| Methods | Design: prospective cohort study  Recruitment: volunteers, leaflets, cessation service kiosk in hospital  Setting: smoking cessation clinic, Italy  Study start date: January 2013; study end date: November 2013 | |
| Participants | Total N: 50  Inclusion criteria: healthy people who smoke; 18 to 60 years; smoking ≥ 15 conventional cpd ≥ 10 years; unwilling to quit  Exclusion criteria: none stated  40% women; mean age 41; mean cpd 25; mean FTND 6.0  No EC use at baseline, not motivated to quit | |
| Interventions | **EC: Refillable**  2nd generation devices (personal vaporisers - PVs): EGO/CE4 model, filled with tobacco aroma e-liquid containing 9 mg/mL nicotine; instructed to use the study products ad libitum (up to a maximum of 5 ml/day; i.e. half vial)  Behavioural support:  Participants were instructed how to charge, fill, activate and use the EC. Key troubleshooting was addressed and phone numbers were supplied for assistance. “No emphasis on encouragement, motivation and reward for the smoking cessation-related efforts were provided during the study.” | |
| Outcomes | 4, 8, 12, and 24 weeks  30-day PP verified by CO ≤ 10 ppm  Adverse events  Cpd, exhaled CO, reduction rates, product usage, and opinions of the EC products | |
| Study funding | "The authors wish to thank FlavourArt (Oleggio, NO, Italy; www.flavourart.it). Authors wish to thank LIAF, Lega Italiana Anti Fumo (Italian acronym for Italian Anti Smoking League) for supporting this research". | |
| Author declarations | "RP has received lecture fees and research funding from Pfizer and GlaxoSmithKline, manufacturers of stop-smoking medications. He has also served as a consultant for Pfizer and Arbi Group Srl, an Italian distributor of e-Cigarettes. RP is currently scientific advisor for LIAF, Lega Italiana Anti Fumo (Italian acronym for Italian Anti Smoking League). PC, MM, JBM, and CR have no relevant competing interest to declare in relation to this work". | |
| ***Risk of bias*** | | |
| **Bias** | **Authors' judgement** | **Support for judgement** |
| Random sequence generation (selection bias) | High risk | Not controlled |
| Allocation concealment (selection bias) | High risk | Not controlled |
| Incomplete outcome data (attrition bias) All outcomes | Low risk | 76% followed up, ITT analysis used, no significant differences in baseline characteristics between completers and those lost to follow-up |
| Selective reporting (reporting bias) | Unclear risk | Unable to determine prespecified outcomes |

Polosa 2015\*

| ***Study characteristics*** | | |
| Methods | Design: prospective cohort  Recruitment: professional retail staff in participating vape shops  Setting: 7 vape shops in Catania province, Italy  Study start date/end date: not specified | |
| Participants | Total N: 71  Inclusion criteria: adults who smoke (≥ 18 years); making first purchase at participating vape shop (definition of smoker not stated)  Exclusion criteria: none stated  38% women, mean age 41.7, mean cpd 24.9, mean FTND 5  No EC use at baseline | |
| Interventions | **EC: Refillable**  Instructed how to charge, fill, activate and use EC; key troubleshooting advice provided; phone number available for technical support; “Encouraged to use these products in anticipation of reducing the number of cig/day smoked” | |
| Outcomes | 6 and 12 m follow-up  30-day PPA via self-report  Details of product purchase  Sustained 50% and 80% reduction in cpd from baseline | |
| Study funding | Authors wish to thank the local participating Vape Shops and LIAF, Lega Italiana Anti Fumo (Italian acronym for the Italian Anti-Smoking League) for supporting this research. | |
| Author declarations | Riccardo Polosa has received lecture fees and research funding from Pfizer and GlaxoSmithKline, manufacturers of stop-smoking medications. He has also served as a consultant for Pfizer and Arbi Group Srl, an Italian distributor of e-Cigarettes. Riccardo Polosa is currently scientific advisor for LIAF, Lega Italiana Anti Fumo (Italian acronym for Italian Anti-Smoking League). Jacques Le-Houezec is a consultant for Johnson & Johnson France, a manufacturer of nicotine replacement therapy, and was reimbursed for travel and accommodation to present at a conference in Shenzhen (China) organized by the e-cig manufacturer association (CECMOL). Pasquale Caponnetto and Fabio Cibella have no relevant conflict of interest to declare in relation to this work. | |
| ***Risk of bias*** | | |
| **Bias** | **Authors' judgement** | **Support for judgement** |
| Random sequence generation (selection bias) | High risk | Not controlled |
| Allocation concealment (selection bias) | High risk | Not controlled |
| Incomplete outcome data (attrition bias) All outcomes | Low risk | 69% follow-up at 12 m. Participants lost to follow-up considered as continuing smokers |
| Selective reporting (reporting bias) | Unclear risk | Unable to determine prespecified outcomes |

Pope 2024

| ***Study characteristics*** | | |
| Methods | RCT  Two-arm pragmatic, multicentre, parallel-group, individually randomised controlled trial carried out at 6 UK NHS Emergency Departments  Study start date January 2022 | |
| Participants | People attending the Emergency Department who smoke  Total N: 972.  Intervention: 484; control: 488  Female 38%; mean age 40.5 (SD 13.65); median CPD 15 (IQR 10 to 20). Mean FTND 4.89 (SD 2.31). Motivation to quit score, mean 4.13 (SD 1.60).  Inclusion: adults (aged 18 years or older) who reported smoking tobacco daily, attending the ED for medical treatment.  Exclusion: expired carbon monoxide (CO) of < 8 per million (ppm), required immediate medical treatment, were in police custody, had a known allergy to nicotine, were current dual users (defined as daily e-cigarette use). | |
| Interventions | EC intervention:  1) EC starter kit (DotPro, manufactured by Liberty Flights, an independent e-cigarette manufacturer not funded by the tobacco industry) ‘pod’ device. The kit included 11 pods (3 tobacco flavoured, 4 berry flavoured and 4 menthol flavoured) of 20mg/mL nicotine strength. Training in use of EC.  2) Brief smoking cessation advice.  3) Referral to stop smoking services. FU consisted of telephone call offering support &, if taken up, offer of free NRT.  Control:  Treatment-as-usual. Signposting to NHS smoking cessation services through provision of written information about local services. | |
| Outcomes | Baseline, 1, 3, and 6 months  Continuous smoking abstinence, 6 months after randomization, CO confirmed  Smoking status 1, 3, and 6 months after randomization. Abstinence prevalence, CO validated (≥ 8 ppm)  Number of cpd; number of times using an EC per day; self-reported dry cough; mouth or throat irritation; use of GP services; use of smoking cessation services. Quality of Life questionnaire. Adverse events (6 months). | |
| Study funding | National Institute of Health Research, Health Technology Assessment (129438) | |
| Author declarations | None declared | |
| Notes | Data new to 2024 update | |
| ***Risk of bias*** | | |
| **Bias** | **Authors' judgement** | **Support for judgement** |
| Random sequence generation (selection bias) | Low risk | Computer-generated randomization employed varying block sizes and stratified by the recruitment sites, which allowed for concealment of allocation |
| Allocation concealment (selection bias) | Low risk | Computer-generated randomization allowed for concealment of allocation |
| Blinding of participants and personnel (performance bias) All outcomes | High risk | Due to the participatory nature of the intervention, it was not feasible to blind participants or those delivering the intervention to group allocation  Interventions not equally intensive |
| Blinding of outcome assessment (detection bias) All outcomes | Low risk | CO measured |
| Incomplete outcome data (attrition bias) All outcomes | Low risk | EC 351/484 = 72.5%  Control 317/488 = 65% |
| Selective reporting (reporting bias) | Low risk | Reported as pre-specified. Cessation and AEs |

Pratt 2016

| ***Study characteristics*** | | |
| Methods | Design: 0bservational study – uncontrolled experimental study  Recruitment: community mental health centre through self-referral and clinician referrals  Setting: community mental health centre (USA)  Study start date: October 2013; study end date: June 2014 | |
| Participants | Total N: 19 (21 originally recruited, however 2 participants did not return for any weekly visits so 19 analyzed)  Inclusion criteria:   - Age ≥ 18 years - Primary DSM-IV axis I diagnosis, based on chart review and confirmation by the community mental health centre team psychiatrist, of schizophrenia, schizoaffective disorder, or bipolar disorder - SMI defined by at least moderate impairment in multiple domains of life functioning due to mental illness - Smoking at least 10 cigarettes per day - History of failed treatment-facilitated quit attempts - Voluntary informed consent for participation   Exclusion criteria:   - Current use of e-cigarettes - Medical instability - Primary diagnosis of dementia or significant cognitive impairment defined as a Mini-Mental Status Examination (MMSE) score < 24   Inclusion based on specific population characteristics: psychiatrically stable, in-treatment, people who smoke, with a schizophrenia spectrum disorder or bipolar disorder  68% women; mean age 42; mean cpd: only cigarettes per week reported: 192 (SD = 159.3). This would be an average of 27 cpd; mean FTND 5.5  Motivated to quit: “None of the participants was actively engaged in a quit attempt during the study”.  E-cigarette use at baseline: E-cig use was an exclusion criterion. | |
| Interventions | **EC: Cig-a-like**  E-cigarette details: (NJOY brand) based on each participant's level of use of combustible tobacco. Each e-cigarette cartridge was approximately equivalent to 2 packs of combustible cigarettes. Trained research interviewers instructed participants on the proper use of e-cigarettes. | |
| Outcomes | Week 1, 2, 3, 4  Adverse events and biomarkers:   - Breath CO level - Possible side effects   Other outcomes measured:   - Use of tobacco products - Fagerström nicotine dependence scores - Appeal of EC - Level of enjoyment of EC - Satisfaction with EC compared with usual combustible tobacco - Willingness to purchase EC | |
| Study funding | “Financial support to purchase the e-cigarettes and pay small stipends to the participants in this unfunded pilot study came from Dr. Mary Brunette's discretionary reserve account.” | |
| Author declarations | “All authors declare that they have no conflicts of interest”. | |
| ***Risk of bias*** | | |
| **Bias** | **Authors' judgement** | **Support for judgement** |
| Random sequence generation (selection bias) | High risk | Not randomized |
| Allocation concealment (selection bias) | High risk | Not randomized |
| Incomplete outcome data (attrition bias) All outcomes | Low risk | 2 dropouts (9.5%) failed to return to clinic. Analysis based on 19 participants |
| Selective reporting (reporting bias) | Low risk | All expected outcomes reported |

Pratt 2022

| ***Study characteristics*** | | |
| Methods | Design: RCT  Recruitment: clinician referrals, posters/brochures and mailings. After eligibility confirmation, potential participants were invited for an informational meeting, and interested individuals returned to review the consent form and provide written informed consent.  Setting: 2 urban mental health agencies (Kentucky and Massachusetts) serving primarily Medicaid beneficiaries with SMI; USA  Study start date: 1 March 2017. Study end date: 31 January 2021 | |
| Participants | Total: N = 240  EC = 120; Assessment only = 120  Inclusion criteria:1) Diagnosis of schizophrenia, schizoaffective disorder, or bipolar disorder, 2) enrolled in services at the research site for a minimum of 3 months, 3) regular smoker (approx. 10 cigarettes for the past 5 years) with a history of at least 1 quit attempt, 4) 18+ years  Exclusion criteria:1) Regular use of EC in the past month, 2) current interest/plan to quit smoking, 3) regular use of NRT or bupropion or varenicline to quit smoking, 4) use of emergency room or hospitalization for psychiatric reasons in the past 30 days, 5) pregnancy, 6) psychiatric instability (hospitalization in the past month), 7) active substance use disorder  Female 47.9%; mean age 45.9 (SD 11.9); mean CPD 18.7; mean FTND 6.9 (SD 1.5)  E-cigarette use at baseline: no  Motivated to quit: no  Inclusion based on specific population characteristics: people living with SMI | |
| Interventions | EC: **Cartridge**  Arm 1: EC  The Study Coordinator provided participants with a 2-week supply of e-cigarettes (EC) and instructions on their safe use. Per product packaging, each disposable EC provided up to 300 puffs, roughly the equivalent of 20 cigarettes. Participants were given the opportunity to practice using EC before leaving the appointment to ensure proper use. The Study Co-ordinator also provided brief information on safety (e.g. keeping EC out of the reach of children) and they gave participants additional 2-week supplies at 2, 4, and 6 weeks.  The EC arm was provided with 8 weeks of free ECs based on self-report of regular tobacco use. Participants assigned in this arm were asked to switch combustible tobacco with ECs. The appeal of EC and health impacts were measured, but the authors were not targeting quitting combustible tobacco or reducing craving.  Arm 2: Assessment only (no intervention)  EC was given at final FU visit. Following randomization, Study Co-ordinators provided participants with appointments for follow-up study visits, asked them to refrain from using ECs, and reminded participants that they would receive a 4-week supply of ECs at the final follow-up visit. | |
| Outcomes | Baseline, 2, 4, 6, 8, 13, and 26 weeks  Breath CO was measured by the blinded Research Interviewers at each visit using the Smokerlyzer Breath Carbon Monoxide Monitor (Bedfont Scientific) as a biologic measure of toxin exposure.  CO, CPD, nicotine dependence, EC use (EC count), NNAL  AEs: Cough, itchy throat, bad throat  Stated that the following would be collected in the NCT record, but not yet reported: change in cancer-related toxin, 4-(Methylnitrosamino)-1-(3-pyridyl)-1-butanol (time frame: baseline, 4 weeks, 8 weeks, 13 weeks, 26 weeks) | |
| Study funding | The study was funded by the National Institute on Drug Abuse (NIDA, 1R01DA041416) in the United States. | |
| Author declarations | None declared | |
| Notes | New to 2022 update | |
| ***Risk of bias*** | | |
| **Bias** | **Authors' judgement** | **Support for judgement** |
| Random sequence generation (selection bias) | Low risk | Quote: “Unblinded Study Coordinator randomly assigned participants within site using an automated program that stratified by diagnosis (schizophrenia vs. bipolar disorder) and amount of daily smoking (> 20 vs. ≤ 20 cigarettes), in blocks of four to assure balance between arms (1:1 ratio).” |
| Allocation concealment (selection bias) | Low risk | As above, automated programme |
| Blinding of participants and personnel (performance bias) All outcomes | High risk | Interventions of different intensity. EC vs assessment only (control) |
| Blinding of outcome assessment (detection bias) All outcomes | Low risk | Quote: "Blinded Research Interviewers” |
| Incomplete outcome data (attrition bias) All outcomes | Low risk | 240 randomized participants. Amongst those participants, 210 (87.5%) were assessed at 8 weeks, and 214 (89.2%) were assessed at 26 weeks. |
| Selective reporting (reporting bias) | Unclear risk | Some outcomes not reported here |

Price 2022

| ***Study characteristics*** | | |
| Methods | Design: single-arm intervention  All CC users offered smoking cessation support and free EC  Setting: pharmacy-supported e-cigarette smoking cessation intervention delivered by 6 pharmacies (funded by the local authority) Greater Manchester, Northwest England, UK. Socially deprived area  Recruitment: advertising conducted through newspapers and in pharmacies, press release, workplace marketing and posters in social housing facilities and by word of mouth. Attendance was incentivized by free equipment and refills. Pharmacies were chosen that had existing skills in delivering smoking cessation services.  Study dates: smoking cessation scheme delivered January to June 2019 | |
| Participants | Total N: 871 (single arm)  Inclusion criteria: combustible cigarette smoker  Exclusion criteria: not stated  57% female  Age 18 to 21 (9.3%); 25 to 34 (21.7%); 35 to 44 (17.3%); 45 to 54 (24.8%); 55 to 64 (19%); 65+ (7.9%)  Category of smoking: 197/860 lowest (1 to 10); 240/860 medium (1 to 19); 396/860 highest (20+)  Motivated to quit: self-presented assumed interested in quitting/interested in free EC | |
| Interventions | EC intervention  EC, charger, and fluids. All equipment was sourced from a partner e-cigarette supplier and provided by pharmacies. EC included: bigger Arc Slim variety, which had longer-lasting batteries and adjustable strength  Pharmacies gave practical advice and support on smoking cessation and e-cigarette use, which was available throughout the 3-month programme.  The pharmacy consultations were seen as an opportunity to give tailored advice to service users and support and encourage them during the quitting process. The use of a carbon monoxide monitor at the pharmacies was described as a useful way of motivating service users to quit. | |
| Outcomes | Baseline, 2 weeks and 4 weeks (endpoint), 3 months, 12 months  Self-report quit at 12 months. CO confirmed quit 4 weeks.  Participants asked to report back to the pharmacy after 4 weeks, 3 months, and 12 months. If happy to be contacted at 3 months service users gave their phone number. After 12 months, the borough council sent text messages to all service users with a valid phone number on record and who had provided consent to be contacted. At 12 months, 355 texts successfully delivered and 77 users returned surveys. | |
| Study funding | Funding for the project was provided by Trafford Metropolitan Borough Council. | |
| Author declarations | The authors stated that they had no competing interests to declare. | |
| ***Risk of bias*** | | |
| **Bias** | **Authors' judgement** | **Support for judgement** |
| Random sequence generation (selection bias) | High risk | Not randomized |
| Allocation concealment (selection bias) | High risk | Uncontrolled |
| Incomplete outcome data (attrition bias) All outcomes | High risk | 77/871 at 12-month follow-up |
| Selective reporting (reporting bias) | Unclear risk | No protocol/clinical record |

Pulvers 2018

| ***Study characteristics*** | | |
| Methods | Design: observational, uncontrolled, experimental study  Recruitment: community  Setting: visits took place in university labs, USA  Study start date: January 2015; Study end date: April 2015 | |
| Participants | Total N: 40  Inclusion criteria: ≥ 18 years; cigarette smoking ≥ 4 days of the past 30 days for at least 1 year; never using EC regularly (less than 25 lifetime uses); not having used EC on ≥ 3 of the past 30 days; willing to switch from smoking regular cigarettes to ECs; fluency in English; regular access to a telephone and transportation to attend appointments; willing to abstain from using marijuana during the study  Exclusion criteria: any use of other tobacco products (OTPs) including smokeless tobacco, cigarillos, pipes, cigars, hand-rolled cigarettes, and hookah in the past 30 days; currently in a smoking cessation programme or another clinical trial; past 30-day use of NRT or medication which aids smoking cessation including bupropion, clonidine, nortriptyline, or varenicline; uncontrolled asthma, severe allergies, or diabetes mellitus; taking prescription medication for emotional distress, depression, or other psychological problems; current dependence on a substance other than nicotine; presence of any cardiovascular or pulmonary illnesses in the past 6 months; pregnancy  Inclusion based on specific population characteristics: no  27% women; mean age 30.08; mean cpd 8.76; FTND not reported  Motivated to quit: over half either did not intend to quit at all or did not intend to quit in the next 6 months 22/40 (55%).  E-cigarette use at baseline: inclusion criteria included the following:   - Never using EC regularly (less than 25 lifetime uses) - Not having used EC for more than 3 of the past 30 days | |
| Interventions | **EC: Refillable**  2nd generation EC starter kit with 2 e-Go C batteries (3.7 volts/650 MaH), a USB connection cord, an AC adapter, and a carrying case, and a supply of Saturn V4i atomizers (2.4 ohms) filled with liquid in their preferred flavour (28 atomizers total; 2/day). Provided 24 mg/mL dosage vegetable glycerin liquid in a tester sample to all participants. Those who reported the 24 mg was too strong were provided 12 mg/mL dosage liquid. The first session included brief education, training, action planning for making a complete switch to EC. A referral to the California Smokers’ Helpline was made at the final visit (week 4). | |
| Outcomes | 3 lab visits (baseline, week 2, and week 4) and 2 phone visits (week 1 and week 3). Biological samples were taken at all 3 in-person visits (baseline, week 2, and week 4). However, due to budgetary restrictions, only the baseline and week 4 biological data were analyzed.  Adverse events and biomarkers:   - Biochemical measures only: breath samples were taken with a Micro + (Bedfont, Haddonfield, NJ) to measure CO - Urine samples taken to test for change in tobacco toxicant exposure by the following measures:   - Concentrations of NNAL measured by liquid chromatography–tandem mass spectrometry (LC–MS/MS)   - Metabolites of a panel of potentially toxic VOCs, including benzene (PMA), ethylene oxide (HEMA), N-nitrosodimethylamine (MMA), acrylonitrile (CNEMA), acrolein(3-HPMA), propylene oxide (2-HPMA), acrylamide (AAMA), and crotonaldehyde (HPMMA) measured by LC–MS/MS, 2   Other outcomes measured:  Cotinine, change in tobacco consumption (cpd using TLFB interview), change in frequency of EC use, change in nicotine dependence and attitudes/behaviour, change in 30-day nicotine exposure | |
| Study funding | “This study was funded by the University of Minnesota (JSA), P30 DA012393 (NLB), P50 CA180890 (NLB), and California State University San Marcos (KP).” | |
| Author declarations | “Benowitz is a consultant to pharmaceutical companies that market smoking cessation medications and has been an expert witness in litigation against tobacco companies. The other authors have no conflicts of interest.” | |
| Notes | New for 2020 update | |
| ***Risk of bias*** | | |
| **Bias** | **Authors' judgement** | **Support for judgement** |
| Random sequence generation (selection bias) | High risk | Not randomized |
| Allocation concealment (selection bias) | High risk | Not randomized |
| Incomplete outcome data (attrition bias) All outcomes | Low risk | 37/40 provided follow-up data. |
| Selective reporting (reporting bias) | Low risk | All outcomes reported |

Pulvers 2020

| ***Study characteristics*** | | |
| Methods | Design: RCT, unblinded, 2:1 ratio  Recruitment: Participants were recruited from the San Diego, California, and Kansas City, Missouri and Kansas, metropolitan areas.  Setting: USA  Study start date: May 2018. Study end date: May 2019 | |
| Participants | Total N = 186; Electronic cigarettes = 125. Own brand cigarette = 61  40.3% female; mean age 43.3 (SD 12.5); mean cpd 12.1 (SD 7.2). E-cigarettes use at baseline: 0.05 (0.3%)  Inclusion criteria: > 21 years of age; smoked cigarettes on > 25 of past 30 days; smoked > 5 cpd on days smoked; smoked cigarettes > 6 months; carbon monoxide > 5 PPM at baseline; systolic BP of < 160 mmHg and diastolic BP of < 105 mmHg at baseline; Hispanic/Latino or African-American/black; fluent in English or Spanish; willing to switch from smoking cigarettes to ECs for 6 weeks; regular access to telephone; transportation to attend appointments (KC Only).  Exclusion criteria: primary use of other tobacco products or equal use of cigarettes and other tobacco products; EC use on > 4 of the past 30 days; currently in a smoking cessation programme or another clinical trial; use of NRT or medication which aids smoking cessation in the past 30 days; hospitalization for a psychiatric issue in the past 30 days; heart-related event in the past 30 days (e.g. heart attack, stroke, severe angina (i.e. chest pain), ischaemic heart disease, and vascular disease); uncontrolled blood pressure; planning to move out of study centres (San Diego or Kansas City) in the next 6 weeks; another person in the household enrolled in the study; pregnancy/breastfeeding; unstable mental status or health status | |
| Interventions | **EC: pod**  **Electronic-cigarettes**: JUUL (5% nicotine); choice of flavours (Menthol, Mango, Cool Mint, Virginia Tobacco); given 1 pod per pack of cigarettes; given a 2-week supply at baseline and then a further 4-week supply at week-2 visit. At each follow-up appointment (week 1, telephone call; week 2, in-person visit; and week 4, telephone call), barriers and benefits of switching to e-cigarettes were discussed and action planning for exclusive switching was revisited. Compensated on a schedule of USD 20 at baseline, USD 40 at week 2 and USD 60 at week 6  **Own brand cigarettes**: compensated on a schedule of USD 20 at baseline, USD 40 at week 2, and USD 60 at week 6 | |
| Outcomes | Baseline, week 2 and week 6. Telephone survey at 6 months  Change in past 7-day combustible cigarette use measured by 7-day timeline follow-back interview  30-day point prevalence at 6 months (EC group only)   - Reduction in toxicant exposure, as measured by NNAL excretion - Cotinine - CO   Lung function; pulmonary function test of small airway disease that is most sensitive to the effects of cigarette smoking; mean mid-expiratory phase of forced expiratory (FEF 25% to 75%); respiratory symptoms as measured with the American Thoracic Society Questionnaire (scores range from 0 to 32, with higher scores indicating greater respiratory symptoms)  Blood pressure  Adverse events: respiratory symptoms | |
| Study funding | Drs Pulvers and Nollen and Ms Rice were supported by grant No. 5SC3GM122628 from the National Institutes of Health (NIH). Drs Schmid and Ahluwalia were supported in part by grant No. P20GM130414, from the NIH-funded Center of Biomedical Research Excellence (COBRE). Dr Schmid was partially supported by Institutional Development Award No. U54GM115677 from the National Institute of General Medical Sciences of the NIH, which funds Advance Clinical and Translational Research (Advance-CTR). | |
| Author declarations | Dr Schmid reported serving as a consultant for legal firms representing Eli Lilly, Boehringer-Ingelheim, and Gilead outside the submitted work. Dr Benowitz reported receiving personal fees from Pfizer and Achieve Life Sciences and serving as a consultant to pharmaceutical companies that market smoking cessation medications and as an expert witness in litigation against tobacco companies outside the submitted work. Dr Ahluwalia reported receiving personal fees from Lucy Goods outside the submitted work. No other disclosures were reported. | |
| Notes | Additional data provided by the authors | |
| ***Risk of bias*** | | |
| **Bias** | **Authors' judgement** | **Support for judgement** |
| Random sequence generation (selection bias) | Low risk | Randomization sequence was generated with an Excel (Microsoft) random number formula applied to each site (2:1 ratio). |
| Allocation concealment (selection bias) | Low risk | Allocation was placed into sealed individual envelopes labelled with participant identification numbers for each site, retrieved from a locked cabinet monitored by the project manager, and opened individually following consent of each participant. |
| Blinding of participants and personnel (performance bias) All outcomes | High risk | Could not be blinded |
| Blinding of outcome assessment (detection bias) All outcomes | Low risk | Carbon monoxide validation |
| Incomplete outcome data (attrition bias) All outcomes | Low risk | E-cig: 115/126  Own brand: 54/61 |
| Selective reporting (reporting bias) | Low risk | Per-protocol reporting |

Rabenstein 2024

| ***Study characteristics*** | | |
| Methods | Prospective, open-label, non-randomised design  Setting: Department for Psychiatry and Institute and Outpatient Clinic for Occupational, Social and Environmental Medicine at the Ludwig Maximilian University of Munich, Germany  Study start date Oct 2015 - Study end date July 2018.  Recruitment: “subjects were recruited in a real-life setting”  EC group: selected EC shops in Munich. Potential participants were contacted by members of the study coordination team. Flyers .  Control group: via regular smoking cessation course of the tobacco ambulance of Department for Psychiatry at the Ludwig Maximilian University of Munich, Germany by informing the participants of the option to participate in the control group. | |
| Participants | N=80  EC =60. CBT supervised smoking cessation = 20  Mean age: EC: 43.7 (SD 12.7); Control 48.4 (SD 11.9). Mean CPD: EC group 19.7 (10.7); Control 17.5 (7.0). Mean FTND: EC 3.5 (1.9); Control 4.1 (1.5).  Inclusion: 18 years of age and legally competent to give consent. They had to consume cigarettes for a minimum of 5 years, with a daily consumption of 10 cigarettes. EC group: intention to completely switch to EC was required at the time of recruitment. Control group: participation in the “Rauchfrei” smoking cessation programme was required.  Exclusion: Pregnancy/breasfeeding. A smoking cessation attempt within 3 months. Relevant allergies, acute psychiatric disorders, ongoing drug, alcohol, or illegal substance abuse, a malignant disease within the last 5 years, severe cardiovascular or pulmonary comorbidities and severe active infectious diseases.  Motivated to quit - yes. “All participants expressed a desire to quit smoking if possible.” | |
| Interventions | EC group: participants planned to completely switch from conventional tobacco cigarettes to e-cigarettes. The participants were free to choose any model, liquid type, and strength of their liking. EC and liquids had to be bought by the participants.  Control group cognitive-behavioural smoking cessation programme + NRT. Use of more than one type of NRT was encouraged. Participants offered a professional introduction to nicotine replacement products, and the possibility of using a combination of multiple nicotine replacement products. NRT was paid for by the participants. Participated in the “Rauchfrei” smoking cessation programme at Department for Psychiatry at the Ludwig Maximilian University of Munich, Germany. The “Rauchfrei Programme (IFT)” is an intensive version of the cognitive behavioural group programme for smoking cessation developed in 2009 widely implemented progremme in Germany. Contains elements of multicomponent treatment and modern psychotherapy, such as dialectical behaviour therapy and acceptance/commitment therapy, as well as psycho-education.  Both groups: 50 euros for complete participation, with attendance of all follow-up appointments as a precondition. | |
| Outcomes | Trial visits at baseline, 3 mths. Questionnaire at baseline, 1, 2, 3 mths.  AE. Lung function (reference not cited correctly, contacted authors no response). | |
| Study funding | There were no funding sources for this study. | |
| Author declarations | D.N. and T.R. are members of the Pfizer Advisory Board on smoking cessation issues. | |
| Notes | New to 2025 update. | |
| ***Risk of bias*** | | |
| **Bias** | **Authors' judgement** | **Support for judgement** |
| Random sequence generation (selection bias) | High risk | Not randomised. 2 groups recruited from different places. “The study has some limitations. The design could not fully exclude the possibility of a selection bias of recruitment into the two groups or regarding the comparability between these groups” |
| Blinding of outcome assessment (detection bias) All outcomes | Unclear risk | AE data not fully reported |
| Incomplete outcome data (attrition bias) All outcomes | Unclear risk | No information provided. |
| Selective reporting (reporting bias) | High risk | Reference for lung function not cited correctly ’20. Anonymized’. |

Rose 2023\*

| ***Study characteristics*** | | |
| Methods | Design: randomized 2 × 2 factorial design; 'switching study'  Design changed mid-study to: nicotine EC + nicotine skin patch vs no nicotine EC + nicotine skin patch  Recruitment: "Participants were recruited from North Carolina"  Setting: North Carolina, USA | |
| Participants | Total N: 94  Placebo pod/Placebo patch arm n = 13; Nicotine pod/Placebo patch arm n = 9; Placebo pod/Nicotine patch arm n = 25; Nicotine pod/Nicotine patch arm n = 29  Study changed to 2 arms: Nicotine EC + nicotine skin patch vs No Nicotine EC + nicotine skin patch  Inclusion criteria: having smoked for the past year; currently smoking at least 10 cigarettes/day; age of 21 to 65 years; baseline expired air carbon monoxide (CO) reading of at least 10 ppm, body weight  Exclusion criteria: a number of major medical conditions; illicit drug use or alcohol abuse/dependence; pregnancy; prior adverse reactions to nicotine patch; use of smoking cessation medications within 30 days; use of tobacco products other than cigarettes within 7 days; or anyone seeking treatment for nicotine dependence  40.4% women; mean age 47.2 (SD 10.3); mean cpd 18.5 (SD 7.3); mean FTND 5.9 (SD 2); mean CO 28.9 ppm (SD14.2)  E-cigarette use at baseline: exclusion criteria use of tobacco products other than cigarettes within 7 days  Motivated to quit: not reported | |
| Interventions | At start of study 4 groups: ENDS (with vs without nicotine); skin patch (with vs without nicotine). Later in the study, all participants were provided with nicotine skin patches and randomized to ENDS with vs without nicotine.  Arms after study change (all participants were provided with nicotine skin patch)  Arm 1: Nicotine EC + nicotine skin patch  JUUL e-cigarette, a breath-actuated, rechargeable closed e-cigarette system. Each pod was pre-filled with 0.7 mL of e-liquid, comprising glycerol, propylene glycol, benzoic acid, flavour, 5% nicotine by weight (59 mg/mL).  Nicotine skin patch: Nicoderm (GlaxoSmithKline, Philadelphia, PA), delivering 21 mg/24 h nicotine purchased from Rejuvenation Labs (Salt Lake City, UT).  Arm 2: No Nicotine EC + nicotine skin patch  JUUL e-cigarette, a breath-actuated, rechargeable closed e-cigarette system. Each pod was pre-filled with 0.7 mL of e-liquid, comprising glycerol, propylene glycol, benzoic acid, flavour, 0% nicotine (placebo).  Nicotine skin patch: Nicoderm (GlaxoSmithKline, Philadelphia, PA), delivering 21 mg/24 h nicotine purchased from Rejuvenation Labs (Salt Lake City, UT).  ECs were offered in 2 flavours, “Cool Mint” and "Virginia Tobacco". | |
| Outcomes | Baseline 2, 4, 6, and 8 weeks  CO measured (in the laboratory, or, after the study went remote, by shipping Bedfont coVita CO breathalyzers to participants with direct observation of CO breath testing through remote televisit (via secure Zoom Videoconference Platform)).  Cessation to 8 weeks  6 SAEs reported, all determined by medical review to be unrelated to treatment. | |
| Study funding | This study was supported by grant 5P50DA027840 from the National Institute on Drug Abuse (NIDA). | |
| Author declarations | JER discloses research support from Foundation for a Smoke-Free World, Philip Morris International, Altria, Embera Neuro Therapeutics, Inc., Otsuka Pharmaceutical, JUUL Labs, consulting with Revive pharmaceuticals, and consulting and patent purchase agreements with Philip Morris International. JMD discloses fnancial support from Predictably Human Inc., including funding for research, consulting fees, and equity. Predictably Human Inc. is focused on development of a prescription treatment for smoking and e-cigarette use. | |
| Notes | Study renamed from NCT03492463 to Rose 2023 in 2024 update | |
| ***Risk of bias*** | | |
| **Bias** | **Authors' judgement** | **Support for judgement** |
| Random sequence generation (selection bias) | Unclear risk | No detail about randomization process: “participants randomly assigned to receive nicotine vs. placebo pods to use in their ENDS devices, and nicotine vs. placebo skin patches” |
| Allocation concealment (selection bias) | Unclear risk | As above |
| Blinding of participants and personnel (performance bias) All outcomes | Low risk | Equally intensive |
| Blinding of outcome assessment (detection bias) All outcomes | Low risk | CO measured |
| Incomplete outcome data (attrition bias) All outcomes | Low risk | 62/76 at 8 week FU |
| Selective reporting (reporting bias) | Low risk | All expected outcomes are reported |
| Other bias | Low risk | Study design changed midway due to COVID-19 and recruitment issues prioritised two arms, therefore participants were not evenly split between the 2x2 design |

Russell 2021\*

| ***Study characteristics*** | | |
| Methods | Design: RCT  Setting: London, UK | |
| Participants | 426, 53% M  NRT = 141; Myblu plus NSPs group = 145; Myblu plus FBNPs group = 140  Inclusion criteria: established daily cigarette smokers aged 18 years and older were recruited in London, UK | |
| Interventions | EC type: pod  3 arms: NRT; mybluTM containing nicotine salt e-liquid pods (NSPs); myblu plus freebase nicotine e-liquid pods (FBNPs)  NRT: over-the-counter nicotine replacement therapies (NRTs). Free for 3 months  Myblu plus NSPs group: a closed system pod e-vapour product (mybluTM) containing nicotine salt e-liquid pods (NSPs). Free for 3 months  Myblu plus FBNPs group: a closed system pod e-vapour product (mybluTM) containing freebase nicotine e-liquid pods (FBNPs). Free for 3 months  Participants of both myblu arms were given a primary device, a backup device, and reimbursement for retail purchases of up to 12 e-liquid pods (6 packs of x2 pods) per month for 3 months. Participants were encouraged to use their assigned e-vapour product and to choose and change flavours and nicotine concentrations of their assigned e-liquid pods as they wished. | |
| Outcomes | Online surveys, administered at study enrolment and then at 1, 2, 3, and 6-months post-enrolment, assessed self-reported past 30-day consumption of conventional cigarettes and use of NRTs and assigned e-vapour products.  Self-reported 6-month past 30-day cigarette abstinence rate  Reduction in smoking | |
| Study funding | E-cigarette/alternative nicotine products industry | |
| Author declarations | NS | |
| Notes | Conference abstract | |
| ***Risk of bias*** | | |
| **Bias** | **Authors' judgement** | **Support for judgement** |
| Random sequence generation (selection bias) | Unclear risk | No information provided |
| Allocation concealment (selection bias) | Unclear risk | No information provided |
| Blinding of participants and personnel (performance bias) All outcomes | Low risk | Unclear if participants were blinded (conference abstract only) but all participants received active interventions, so performance bias judged unlikely |
| Blinding of outcome assessment (detection bias) All outcomes | Low risk | Self-report only but all participants received active interventions, so differential misreport judged unlikely |
| Incomplete outcome data (attrition bias) All outcomes | Low risk | The 6-month retention rate was 85.8% in the NRT group, 85.5% in the myblu plus NSPs group, and 73.6% in the myblu plus FBNPs group. |
| Selective reporting (reporting bias) | Unclear risk | No protocol or clinical trial record available to determine whether all prespecified outcomes were reported |

Scheibein 2020

| ***Study characteristics*** | | |
| Methods | Design: non-randomized, single-arm  Recruitment: from supported temporary accommodation service; project workers and support staff identified potential study participants who smoked and wished to quit  Setting: Dublin Simon Community, Ireland  Study start date: recruitment February 2019 (overall trial start date March 2018). Study end date: June 2019 | |
| Participants | Total N: 23 but only report baseline for the 9 that completed the study. % female 8.7% (2/23) at baseline, (22.2% 2/9) completed and reported; mean age 43.89 (SD 7.36); mean cpd 25.22 (SD 7.77); mean FTND 7.89 (SD 1.2); mean CO 21.89 (SD 14.4 corresp)  E-cigarettes use at baseline: no  Motivated to quit: yes  Inclusion criteria: > 5 CO ppm (carbon monoxide); active smoking status; expressed intention to quit using ENDS-device  Exclusion criteria: self-reported pregnancy; exhibition of florid psychotic or substance-use-related symptoms which could have affected ability to consent | |
| Interventions | **EC: Refillable**  Electronic-cigarette: Endura T22e Electronic Nicotine Delivery System and 2 10 mL bottles of fluid strengths (0, 6, 11, 18, and 20 mg/mL) and flavours ('Purple Berry', 'Ice Menthol', 'Regular Blend', and 'American Tobacco') | |
| Outcomes | Baseline (‘week 1’), week 4, week 8, week 12: CO, adverse events  Also number of cigarettes smoked; Fagerström Test Scores | |
| Study funding | This study was completed as part of a Tobacco Harm Reduction Scholarship funded by Knowledge Action Change. | |
| Author declarations | FS was a recipient of a Tobacco Harm Reduction Scholarship provided by Knowledge Action Change. He is currently the recipient of an Enhanced Scholarship from the same organization. AM and KM acted as mentors for both the Tobacco Harm Reduction Scholarship and Enhanced Scholarship.  AM is an associate of New Nicotine Alliance.  KM is a recipient of a grant from the Foundation for a Smoke Free World.  JW declares no interests.  WR declares no interests. | |
| ***Risk of bias*** | | |
| **Bias** | **Authors' judgement** | **Support for judgement** |
| Random sequence generation (selection bias) | High risk | Only 1 arm |
| Allocation concealment (selection bias) | High risk | Not randomized |
| Blinding of participants and personnel (performance bias) All outcomes | High risk | Not randomized |
| Blinding of outcome assessment (detection bias) All outcomes | High risk | Self-reported adverse events |
| Incomplete outcome data (attrition bias) All outcomes | High risk | 9/23 completed. Reason was many people moved away so not linked to unacceptability of the study. Incomplete paperwork to enable it to be followed |
| Selective reporting (reporting bias) | Unclear risk | Protocol published afterwards |

Sifat 2024

| ***Study characteristics*** | | |
| Methods | RCT  Setting: Homeless Alliance day shelter in Oklahoma City, OK, USA  Recruitment: face to face. Participants were recruited by study staff at the Homeless Alliance day shelter in open space area.  Study start: 14 Sept 2022. Study completion: 1 March 2023 | |
| Participants | Total 60  EC switching = 30  EC switching + financial incentives = 30  25% female ( EC=23.3; EC + FI = 26.7%). Mean age: 48.77 (SD 10.98). Mean CPD: 16.00 (SD 11.53)  76.7% currently experiencing homelessness. (EC group 80.0%; EC + FI = 73.3%.)  Inclusion criteria: ≥21 years of age; Expired CO level ≥8 ppm suggestive of current smoking; reported smoking ≥5 cigarettes per day; willing and able to attend study visits 9 weeks; interested in switching from CCs to ECs; willing to abstain from smoking cannabis for the duration of the study,  Willing to switch from CCs to ECs.  EC use at enrollment: At baseline, 35% (n=21) of participants reported any e-cigarette use in the past 30 days | |
| Interventions | EC vs EC + financial incentives  Study EC: Vuse pod-based e-cigarette device in their preferred flavor (Menthol or Golden Tobacco, 5 % nicotine concentration).  Both groups had the same number of visits and study assessments  1) EC switching (device + pods). At baseline offered 3 refill pods. Encouraged to practice in the week before switching. Participants were advised to completely switch from CCs to the e-cigarette device one week after the baseline visit. On the switch date received refill pods based on their pre-switch CC smoking level (i.e., 1 pod per pack of cigarettes smoked per week), and the pods were replenished at each weekly visit through 4-weeks post-switch (intervention phase). Although participants in the EC group did not earn incentives for CC abstinence, they were yoked with a participant in the EC+FI group (described below), and they earned the same weekly incentive amount as their counterpart earned for CC abstinence credited to a reloadable study credit card.  2) EC switching (device + pods) + financial incentives for combustible cigarette cessation (EC+FI). EC group participants were asked to completely switch from CC to EC. Participants received an EC device + pods and the opportunity to earn financial incentives for biochemically-verified cigarette smoking abstinence, credited to a gift card with biochemical evidence of smoking abstinence during the first 4 weeks following a scheduled quit attempt. Participants could earn $20-$40 incentives at each visit if self-reported CC abstinence over the past 24 hours and had a CO level of ≤8 parts per million (ppm). Participants could earn up to $150 total over 5 visits. Participants who reported CC use over the past 7 days and/or had a CO level >6 ppm did not earn incentives that week but could earn incentives at the next visit with the incentive value reset to $20. | |
| Outcomes | Baseline, 4 weeks, 8 weeks.  Expired CO at 4 weeks and 8 weeks  [CC abstinence to 8 weeks as < 6 mths not included in this review.] | |
| Study funding | This research was supported by Oklahoma Tobacco Settlement Endowment Trust (TSET) grant R23- 02 and Grants P30CA225520 (Stephenson Cancer Center) and P30CA056036 (Sidney Kimmel Cancer Center). J. S. Ahluwalia was funded in part by P20GM130414, a NIH funded Center of Biomedical Research Excellence (COBRE). Manuscript preparation was additionally supported by the National Institute on Minority Health and Health Disparities grant K01MD015295 to A. C. Alexander. | |
| Author declarations | Munjireen S. Sifat, Adam C. Alexander, Michael S. Businelle, Summer G. Frank-Pearce, Laili Kharazi Boozary, and Theodore L. Wagener have nothing to disclose.  Dr. Kendzor is a member of the Scientific Advisory Board of Qnovia. Inc., which is a drug development company focused on inhaled therapies including prescription inhaled nicotine replacement therapy for smok ing cessation (not used or evaluated in the current study).  Dr. Ahluwalia also serves as a consultant and has equity in a start-up company, Qnovia. Qnovia is due to begin Phase I clinical trials to test a prescription nicotine replacement therapy through CEDR at the FDA. Dr. Ahluwalia received sponsored funds for travel expenses as a speaker for annual GTNF conferences from 2021 to 2024; as a speaker for the 2022 and 2024 Tobacco Science Research Conference; and, for the 2021, 2022, and 2023 Food and Drug Law Institute conferences. | |
| Notes | New to 2025 update. | |
| ***Risk of bias*** | | |
| **Bias** | **Authors' judgement** | **Support for judgement** |
| Random sequence generation (selection bias) | Unclear risk | No detail provided. “A total of 60 adults were randomly assigned to one of two e-cigarette switching interventions” |
| Allocation concealment (selection bias) | Unclear risk | No detail provided. No detail of whether it was concealed or not “The staff provided information about the study, answered questions, and screened/enrolled interested individuals in an adjacent private office space.” |
| Blinding of participants and personnel (performance bias) All outcomes | Low risk | Equally intensive. Both arms received ECs. EC arm was yoked to EC + financial incentives. |
| Blinding of outcome assessment (detection bias) All outcomes | Low risk | “Expired CO was assessed (via Vitalograph CO ecolyzer, Vitalograph Inc.) weekly from baseline (pre-switch) through 4 weeks post-switch, and at 8-weeks post- switch.” |
| Incomplete outcome data (attrition bias) All outcomes | High risk | At 8 weeks: EC 14/30 = 46.7%; EC + FI 13/30 = 43.3%. |
| Selective reporting (reporting bias) | Low risk | All reported, NCT record |

Skelton 2022

| ***Study characteristics*** | | |
| Methods | Design: RCT  Recruitment: clients of AOD (alcohol or drug) centre. Informed of study and given PIS. Interested clients were telephoned by RA.  Setting: 2 AOD clinical programmes, an opiate agonist treatment (i.e. methadone or buprenorphine) programme and cannabis clinic (behavioural treatment for cannabis misuse and cannabis use disorder) located within one local health district service in New South Wales, Australia  Study start date: April 2018. Study end date: July 2019 | |
| Participants | Total N: 66 (67 in flow diagram)  EC abrupt CC cessation = 30 (flow diagram 32)  EC gradual CC cessation = 30 (flow diagram 35)  Inclusion criteria: 1) Client of participating HNELHD Alcohol Or Drug (AOD) programme, 2) 18+ years, 3) daily tobacco smoker, 4) interested in making a serious quit attempt in the next 30 days, 5) has not used an END containing nicotine in past month  Exclusion criteria: pregnancy or breastfeeding  Female 26.9%; mean age 42.3 (SD 8); mean CPD 22 (SD 14.2)  Motivated to quit: yes  E-cigarette use at baseline: no | |
| Interventions | EC: **Refillable**  For both arms:  All study participants received T22 and T18 starter kits (both Innokin Endura®). The T22 kit had a 1.5 O atomizer, 2000 mAh battery, and 4.1 mL tank. The T18 kit had a 1.5 O atomizer, 1000 mAh battery, and 2.5 mL tank. Both kits included an additional atomizer and micro USB cable. An additional 5 atomizers were provided with the starter kits as replacements.  A prescription for 12 mg/10 mL nicotine e-liquid was provided for all study participants. Participants received a total of 24 bottles (8 bottles per month). At weeks 3 and 7, participants were provided with their next supply of e-liquid nicotine by either post or face-to-face at the AOD programme for which they were recruited.  Training day to learn how to use the VNP devices  Arm 1: EC abrupt CC cessation  Arm 2: EC gradual CC cessation  At their training day, participants were provided with a personalized gradual cessation schedule based on the baseline number of cigarettes smoked per day recorded in the baseline survey. Participants were told their quit date was 4 weeks hence. Participants were instructed to reduce the number of cigarettes smoked by 25% at week 1, 50% at week 2, 75% at week 3, and 100% at week 4.  Safety was monitored: All participants were briefly contacted to complete safety check-ins by telephone at weeks 1, 3, 5, 7, and 10 following their training day. | |
| Outcomes | Baseline. Training day. 6 weeks post-training day. 12 weeks post-training day  Continuous abstinence  Seven-day point prevalence abstinence, biochemically verified by CO breath test: ≤ 8 ppm  Feasibility outcomes – acceptability, quit type preference, adherence, CPD  Safety: not reported | |
| Study funding | We acknowledge HMRI and Hunter New England Local Health District for the present funding, as well as the participating trial sites staff who notified present clients about the study. | |
| Author declarations | The authors declared that they had no known competing financial interests or personal relationships that could have appeared to influence the work reported in this paper. | |
| Notes | Safety data not reported. No outcomes to extract  New to 2022 update | |
| ***Risk of bias*** | | |
| **Bias** | **Authors' judgement** | **Support for judgement** |
| Random sequence generation (selection bias) | Low risk | Quote: “The randomisation sequence (1:1 in blocks of 4 or 6, stratified by AOD program) was generated by the study statistician using SAS software”. |
| Allocation concealment (selection bias) | Low risk | Quote: "generated by the study statistician using SAS software and were uploaded into REDcap" |
| Blinding of participants and personnel (performance bias) All outcomes | Low risk | Both arms received the intervention. |
| Blinding of outcome assessment (detection bias) All outcomes | Low risk | Quote: “investigators will be blinded for outcome assessments”. |
| Incomplete outcome data (attrition bias) All outcomes | Low risk | Arm 1: 25/31. Arm 2: 27/35 |
| Selective reporting (reporting bias) | High risk | Not all protocol-defined outcomes reported |

Smith 2020

| ***Study characteristics*** | | |
| Methods | Design: double-blind randomized controlled trial  Recruitment: recruited from the local area via advertising on Craigs List social media  Setting: laboratory and electronic diaries, USA  Study start date/Study end date: not specified | |
| Participants | Total N: 30  N per arm: PG/VG ratio 70/30 = NR; PG/VG ratio 50/50 = NR; PG/VG ratio 0/100 = NR  Inclusion criteria: adults aged ≥ 18 years who have been smoking at least 5 cigarettes daily for the past year (expired CO > 8); usual brand is non-menthol; use of ENDS on 5 or fewer lifetime occasions; regular use of email or smartphone ownership with capacity to receive SMS text and internet access (necessary for electronic diaries)  Exclusion criteria: unwilling to use ENDS/EC as part of the trial; use of smokeless, hookah, or tobacco products other than cigarettes ≥ 10 days in the past 30 days; pregnancy/breastfeeding; recent history of cardiovascular distress in the last 3 months (arrhythmia, heart attack, stroke, uncontrolled hypertension); current use of cessation medications; another household member currently enrolled in the study.  30% women; mean age 43.7; mean cpd 18.5; mean FTND 5.4  Motivated to quit: not specified  E-cigarette use at baseline: participants had used an e-cigarette an average of 1.6 times in their life, and no one reported use in the last 30 days. | |
| Interventions | **EC: Cig-a-like**  EC provided for 1 week. All aspects of the ENDS device and e-liquid were held constant between groups with the exception of PG/VG ratio:  **PG/VG ratio 70/30; PG/VG ratio 50/50; PG/VG ratio 0/100.** Ego-T 1100 mAh battery and disposable cartomizers (510 Smoketech, 1.5-Ω dual coil). E-liquid was tobacco-flavoured (Classic Tobacco, American E-liquid) and contained 18 mg/mL of nicotine. | |
| Outcomes | 1 week; 2 lab visits pre and post and participant diaries  Adverse events and biomarkers: participants provided a CO sample at each visit  Other outcomes measured: cpd, ENDS puffs | |
| Study funding | Funding for this project was provided by pilot funding from the National Cancer Institute (P01CA200512 to K.M.C.). Salary support provided by the National Institute on Drug Abuse (K12DA031794 to T.T.S., K23DA041616 to B.W.H.) | |
| Author declarations | M.J.C. has received consulting honoraria from Pfizer. K.M.C. has received payment as a consultant to Pfizer, Inc., for service on an external advisory panel to assess ways to improve smoking cessation delivery in healthcare settings. He also has served as paid expert witness in litigation filed against the tobacco industry. | |
| Notes | Additional data provided from authors. New for 2020 update | |
| ***Risk of bias*** | | |
| **Bias** | **Authors' judgement** | **Support for judgement** |
| Random sequence generation (selection bias) | Unclear risk | Quote: “At the conclusion of the lab visit, participants were randomized and assigned to take home one of the three e-liquids to use at home for a 1-week sampling period (10 participants/ratio).”  Quote: “Participants were randomly assigned to receive one e-liquid to take home for 1 week.” (no further detail given). |
| Allocation concealment (selection bias) | Unclear risk | Not specified |
| Blinding of participants and personnel (performance bias) All outcomes | Low risk | Quote: “PG/VG ratio was blinded from participant and staff members who conducted experimental sessions.” |
| Blinding of outcome assessment (detection bias) All outcomes | Low risk | Biochemical validation |
| Incomplete outcome data (attrition bias) All outcomes | Low risk | Number of participants at follow-up not reported, but this may be due to the 1-week follow-up, and it seems that all participants (excluding 1 participant who was not randomized) were followed up. |
| Selective reporting (reporting bias) | Unclear risk | No protocol. Few details for CO measurements, just percentage change for each group, but mean CO data provided by author on request |

Smith 2025

| ***Study characteristics*** | | |
| Methods | Design: Randomized parallel assignment  Setting: USA | |
| Participants | N = 30  EC = 20, NRT = 10  56.7% female. Mean age 50.4 (11.9 SD). Mean CPD: EC arm 21 (6 SD); NRT arm 22 (9 SD).  Inclusion criteria: at least 21 years old; smoking at least 5 cigarettes per day for 1 year; a quit attempt using an FDA-approved pharmacotherapy in the past year that resulted in abstinence of at least 24 hours; intention to quit smoking within the next month  Exclusion criteria: use of non-cigarette tobacco products (including EC) in the past 30 days; current use of pharmacotherapy for smoking cessation; pregnant, breastfeeding, or trying to become pregnant.  EC use at baseline: no  Motivated to quit: yes | |
| Interventions | EC vs NRT (nicotine patch/nicotine lozenge)  EC arm: 5-week supply of EC. 2 NJOY Ace e-cigarettes and 36 NJOY Ace pods with 5% nicotine. Participants were provided with classic tobacco flavor or menthol flavor pods, matched for their usual brand cigarette flavor.  NRT arm: 5-week supply of combination NRT (transdermal nicotine patch and short-acting nicotine lozenge)(one 14 mg transdermal and 10 4 mg lozenges per day). Only included one dose of patch and lozenge as participants were not going to be seen between the first and last visits, and the lozenge could easily be titrated by the participants as needed but the nicotine patch could not, so a low dose was chosen. Participants in the NRT group were told to use one nicotine patch per day and at least 9 lozenges per day, consistent with standard treatment recommendations. Participants were told that if they ran out of products, they could buy more at a local store.  Both groups:  Participants selected a Target Switch on which they would stop smoking cigarettes and switch entirely to the study product (EC or NRT)  Participants were told to sample their assigned product or medication ad libitum in advance of their chosen switch or quit date, and to stop smoking completely on that date and use only their assigned product or medication.  Participants had a brief behavioral counseling session with study staff in preparation for their switch or quit date. | |
| Outcomes | Baseline, weeks 1, 2, 3 & 4. Baseline to day 35 (5 weeks)  Study assessments were completed remotely each week, and participants returned to the lab 4 weeks after their switch or quit date to provide a breath carbon monoxide sample  Percentage of participants who report daily use of e-cigarettes or NRT and no cigarette use (time frame: day 0 through day 35), daily use of e-cigarette or NRT instead of cigarettes  Percentage of participants who have biochemically confirmed abstinence from cigarette smoking (time frame: day 0 through day 35)  CO measured for all participants. Self-reported respiratory symptoms.  AEs, SAEs (reported in NCT record). | |
| Study funding | Funding for this pilot project was provided by an Institutional Research Grant from the American Cancer Society to the Hollings Cancer Center awarded internally to TS (IRG-19-137-20). | |
| Author declarations | Drs. Cummings and Toll both report testifying as expert witnesses on behalf of plaintiffs who have filed litigation against cigarette manufacturers. Dr. Toll also reports testifying as an expert witness on behalf of plaintiffs who have filed litigation against Juul Labs. MJC serves as a paid expert consultant in litigation involving trade disputes involving e-cigarette manufacturers. All other authors report no conflicts of interest. | |
| Notes | Smith 2025 moved new to 2025 update. NCT05525078 added as ongoing to 2023 update | |
| ***Risk of bias*** | | |
| **Bias** | **Authors' judgement** | **Support for judgement** |
| Random sequence generation (selection bias) | Unclear risk | “Eligible participants completed additional baseline measures and were randomized in a 2:1 fashion.” No further detail about randomisation process. |
| Allocation concealment (selection bias) | Unclear risk | No information provided |
| Blinding of participants and personnel (performance bias) All outcomes | Low risk | Randomised groups are not blinded but of equal intensity. |
| Blinding of outcome assessment (detection bias) All outcomes | Low risk | CO measured. (Respiratory symptoms self-reported) |
| Incomplete outcome data (attrition bias) All outcomes | Low risk | EC 18/20; NRT 10/10 |
| Selective reporting (reporting bias) | Low risk | AEs & SAEs reported in NCT record, not in paper. CO reported in paper, not in NCT record. Data is reported. |

Stein 2016

| ***Study characteristics*** | | |
| Methods | Design: non-controlled, open-label, experimental study  Recruitment: a flyer posted at a large methadone maintenance treatment programme  Setting: methadone maintenance treatment programme, USA  Study start date: April 2015; study end date: not specified | |
| Participants | Total N: 12  Inclusion criteria: current moderate or heavy cigarette use (10+ cpd for at least 12 months prior to enrolment); current MMT for at least 3 months; ready to make a smoking quit attempt in the next 14 days; plan to remain on MMT for at least 12 weeks  Exclusion criteria: used EC on ≥ 2 of the past 30 days; currently used medications that may reduce smoking (bupropion, varenicline, NRT); had unstable medical or psychiatric conditions (past month suicidal ideation or past year suicide attempt, hospitalization for myocardial infarction or stroke in the prior 3 months); had regular use of marijuana (self-report or positive urine drug test)  Inclusion based on specific population characteristics: people receiving MMT for opoid use disorder  50% women; mean age 45.9; mean cpd 17.8; mean FTND: not reported  Motivated to quit: yes  E-cigarette use at baseline: had not used e-cigarettes for more than 2 of the past 30 days | |
| Interventions | **EC: Cig-a-like**  2-week supply of NJOY e-cigarettes at week 1 (quit day), consisting of 5 packs of NJOY e-cigarettes (15 in total). Participants could request an additional 5 packs (20 in total) for the following 2-week study period, if they ran out before a study visit. Participants instructed to use EC exclusively for a total of 6 weeks (end of treatment). They were referred to the state telephone QuitLine for supportive counselling at the quit-day visit (week 1) | |
| Outcomes | Participants quit and received e-cigs at week 1. Assessments were carried out at week 3, 5, 7, and 9.  Adverse events and biomarkers:   - “Side effects” of e-cigarettes were recorded. Side effects were rated none, slight, mild, moderate, and severe at every assessment visit. An adverse effect possibly related to e-cigarette use was defined as positive if the value at baseline was either none or slight AND the value at any of 3, 5, or 7 weeks was mild or more severe.   Other outcomes measured:   - Reduction in the average cpd - E-cig adherence - Nicotine withdrawal | |
| Study funding | “MDS is a recipient of National Institute on Drug Abuse Award K24 DA000512. This award funded the project described here.” | |
| Author declarations | “None declared” | |
| ***Risk of bias*** | | |
| **Bias** | **Authors' judgement** | **Support for judgement** |
| Random sequence generation (selection bias) | High risk | No randomization |
| Allocation concealment (selection bias) | High risk | No randomization |
| Incomplete outcome data (attrition bias) All outcomes | Low risk | Quote: “One individual dropped out after week 3 and did not return; another completed all follow-up assessments except week 7.” |
| Selective reporting (reporting bias) | Low risk | All expected outcomes reported |

Strasser 2016

| ***Study characteristics*** | | |
| Methods | Design: randomized, factorial trial (participants were randomized to one of the 5 brands of e-cigarettes – although only 4 brands analyzed)  Recruitment: media ads  Setting: recruitment from the community; study took place at university, USA  Study start date/study end date: not specified | |
| Participants | Total N: analysis based on 24 (28 originally recruited, but the first 4 participants enrolled experienced malfunctioning NJOY e-cigs and withdrew – the project was removed from the market before the 5th participant was randomized).  N per arm: Blu: 6; Green Smoke: 6; V2: 6; White Cloud: 6  Inclusion criteria: age 18 to 65 and self-reported smoking at least 10 cigarettes per day  Exclusion criteria: use of other tobacco or nicotine-containing products, including e-cigarettes (no more than 3 previous episodes of use and not currently using); current diagnosis or evidence of substance abuse or dependence or major depression; current or history of psychotic or bipolar disorder; history of suicide attempt; history of cancer or cardiovascular disease; uncontrolled hypertension; use of smoking cessation medications; current plans to try to quit smoking; pregnancy or lactation  Inclusion based on specific population characteristics: not applicable  29% women; mean age 43.3; mean cpd 17; mean FTND 3.7  Motivated to quit: Participants had no current plans to try to quit smoking (eligibility criterion).  E-cigarette use at baseline: no more than 3 previous episodes of use and not currently using (eligibility criterion) | |
| Interventions | **EC: Cig-a-like**  All participants received nicotine EC and were instructed to use them exclusively for 9 days.  The 5 brands selected, including brand-reported nicotine levels, were: (1) NJOY (18 mg nicotine) – this brand was discontinued and not analyzed as the e-cigs provided malfunctioned; (2) V2, 18 mg nicotine; (3) Green Smoke, 18.9 to 20.7 mg nicotine; (4) Blu, 20 to 24 mg nicotine; and (5) White Cloud, 23 to 24 mg nicotine. Each brand advertised the delivery of the same level of nicotine (appropriate for about a pack/day smoker), provided the standard tobacco flavour (no other flavours made available), and used a disposable cigarette-like device. | |
| Outcomes | Day 10 was the only testing point of interest for this review, but participants were also tested at days 1 and 5.  Adverse events and biomarkers:   - Breath CO - Direct effects of nicotine (e.g. dizziness, nausea, headache) - visual analogue scale with a single word scored from 0 (not at all) to 100 (extremely). Total scores were summed such that higher scores indicated negative responses.   Other outcomes measured:   - E-cigarette use - Direct effects of the e-cigarette (e.g. satisfying, calming, pleasant, smoke another right now) - visual analogue scale with a single word scored from 0 (not at all) to 100 (extremely). Total scores were summed such that higher scores indicated positive responses. - cotinine - Withdrawal and craving | |
| Study funding | “National Cancer Institute (NCI) of the National Institutes of Health (NIH) and FDA Center for Tobacco Products (CTP) under Award Number P50CA179546, as well as grants from the National Cancer Institute (P50 CA143187, P30 CA16520, and P30 DA12393)” | |
| Author declarations | “Dr Benowitz has served on scientific advisory boards for Pfizer and GlaxoSmithKline related to smoking cessation medications and has been an expert witness in litigation against tobacco companies. Dr Schnoll receives medication and placebo free of charge from Pfizer and has provided consultation to Pfizer and GlaxoSmithKline. These companies had no involvement in this study. Dr Strasser has received funding through the Pfizer GRAND programme, an independent peer-reviewed grant programme funded through Pfizer (2008-2011); all investigators have received funding from the United States National Institutes of Health”. | |
| ***Risk of bias*** | | |
| **Bias** | **Authors' judgement** | **Support for judgement** |
| Random sequence generation (selection bias) | Unclear risk | Although participants were randomized to different brands of EC, no description on how randomization was carried out |
| Allocation concealment (selection bias) | Unclear risk | Not specified |
| Blinding of participants and personnel (performance bias) All outcomes | Unclear risk | No description of whether groups were blind to other conditions, but given similar levels of support between arms, so performance bias judged unlikely |
| Blinding of outcome assessment (detection bias) All outcomes | Low risk | Unclear whether any blinding took place, some outcomes were measured using objective measures and there was no difference in contact between arms. |
| Incomplete outcome data (attrition bias) All outcomes | High risk | For blu, Green Smoke, and V2 groups, 83% of participants completed the 10-day study; only 33% of participants randomized to White Cloud completed the 10-day study; meaning loss to follow-up was considerably higher in this group. |
| Selective reporting (reporting bias) | Low risk | All expected outcomes reported |

Tattan-Birch 2023

| ***Study characteristics*** | | |
| Methods | Design: RCT  Recruitment: stop-smoking services in England, free access for smokers trying to quit. Services recruited participants and delivered the intervention during one-to-one in-person counselling sessions with trained stop-smoking advisors.  Setting: 6 stop-smoking services, England, UK  Study start date: April 2019. Study end date: November 2021 | |
| Participants | Total: N 92  Arm 1: E-cigarette-varenicline group 48  Arm 2: Varenicline-only group 44  Inclusion criteria: 1) aged 18+ years, 2) smoker, 3) attending SSS one-to-one specialist support in London Local Authorities, 4) firm target quit date, 5) elect to use varenicline to support quit attempt, 6) willing to try e-cigarettes  Exclusion criteria: pregnancy or breastfeeding  Female 51%; mean age 43.9 (SD 13.1)  E-cigarette use at baseline: no  Motivated to quit: yes | |
| Interventions | EC **refillable**  Arm 1: EC + varenicline  EC: a nicotine e-cigarette starter-kit, Aspire PockeX e-cigarette e-liquid to last for approximately 4 weeks, and an information booklet. Participants could choose a total of eight 10 mL e-liquid bottles (from Aspire or Totally Wicked) in any combination from a selection of 3 flavours (fruit, menthol, and tobacco) and 3 nicotine concentrations (6, 12, and 18 mg/mL). Participants were encouraged to buy further bottles from local vape shops.  Varenicline: same for both arms. Prescribed the standard 12-week course of varenicline, starting approximately 2 weeks prior to their target quit date. They were advised to take one 0.5 mg pill daily for the first 3 days, then two 0.5 mg pills daily for days 4 to 7, and finally two 1 mg pills daily for the remaining 11 weeks. As this was a pragmatic trial, participants were not asked to avoid using e-cigarettes.  Behavioural support: weekly or fortnightly support until 12 weeks after their quit date. Behavioural support aimed to minimise participants’ motivation to smoke, maximise their motivation to remain abstinent, and guide their use of pharmacotherapy.  Arm 2: Varenicline only  Same as Arm 1 for varenicline and behavioural support | |
| Outcomes | Baseline, 9 to 12 weeks  Smoking abstinence, self-reported between weeks 9 and 12 from the target quit date and validated by an expired air CO concentration of below 10 ppm at week 12  Adherence to varenicline  During each session, advisors recorded smoking status, exhaled CO, adherence, adverse events, and respiratory symptoms using existing software (QuitManager or PharmOutcomes). | |
| Study funding | This project was funded by the Global Research Awards for Nicotine Dependence (GRAND) unrestricted research grant programme supported by Pfizer. Additional funding was provided by Cancer Research UK (PRCRPG-Nov21\100002). All authors are members of the UK Centre for Tobacco and Alcohol Studies (UKCTAS), funded under the auspices of the UK Clinical Research Collaboration (MR/K023195/1). | |
| Author declarations | LS has received a research grant and honoraria for a talk and travel expenses from manufacturers of smoking cessation medications (Pfizer and Johnson & Johnson). JB has received unrestricted research funding from Pfizer to study smoking cessation. RW has received travel funds and hospitality from, and undertaken research and consultancy for, pharmaceutical companies that manufacture or research products aimed at helping smokers to stop. The other authors have no conflicts of interest to declare. None of the authors have ever received personal fees or research funding of any kind from electronic cigarette or tobacco companies. | |
| Notes | “The trial was stopped early due to COVID-19 restrictions and a varenicline recall (92/1266 participants used).”  “The evidence is tentative because our sample size was smaller than planned — caused by Coronavirus Disease 2019 (COVID-19) restrictions and a manufacturing recall. This meant our effect estimates were imprecise, and additional evidence is needed to confirm that providing e-cigarettes and varenicline together helps more people remain abstinent than varenicline alone.” | |
| ***Risk of bias*** | | |
| **Bias** | **Authors' judgement** | **Support for judgement** |
| Random sequence generation (selection bias) | Low risk | Quote: "Participants were randomised [by] 1:1 ratio in blocks of 6 or 8 participants, stratified by service, using a computer-generated random sequence with allocation concealed within opaque envelopes. Due to the nature of the intervention, participants and advisors could not be blinded to treatment assignment." |
| Allocation concealment (selection bias) | Low risk | Quote: "with allocation concealed within opaque envelopes" |
| Blinding of participants and personnel (performance bias) All outcomes | Low risk | 2 active interventions |
| Blinding of outcome assessment (detection bias) All outcomes | Low risk | CO-validated smoking abstinence at 6 months following the target quit-date was measured. Trial stopped at 12 weeks |
| Incomplete outcome data (attrition bias) All outcomes | High risk | EC + varenicline: 26/48  Varenicline only: 20/44 |
| Selective reporting (reporting bias) | Low risk | Preregistered and all expected outcomes reported (some outcomes not reported, but that was judged due to early termination) |

Tseng 2016

| ***Study characteristics*** | | |
| Methods | Design: 2-arm; double-blind, placebo-controlled RCT  Recruitment: advertisements placed in Craigslist as well as flyers distributed on the street and placed in New York City venues with details for how to contact study staff  Setting: community, USA  Study start date: July 2014 to 2015 (month unclear); study end date: not specified | |
| Participants | Total N: 99 (100 were randomized, but 1 participant randomized to the control arm was found to be ineligible between randomization and baseline)  N per arm: nicotine EC: 50; placebo EC: 49  Inclusion criteria: age 21 to 35; daily smoker; smoked ≥ 10 cigarettes a day (verified by a CO level of ≥ 8 ppm); interested in reducing cigarette consumption; able to provide consent; had a cell phone and was willing/able to receive text messages and counselling on their cell phone; willing to use an EC for 3 weeks  Exclusion criteria: pregnant and/or breastfeeding; history of asthma, other airways diseases, or heart disease; currently using smoking cessation medications (including other forms of NRT, bupropion, or varenicline), or enrolled in a smoking cessation programme or another cessation trial; use of EC in the past 14 days or any other tobacco products (pipe, cigar, cigarillos, snuff, chewing tobacco, rolling tobacco, or hookah/shisha) in the past 30 days; moderate-to-severe drug use disorder defined as a score of ≥ 5 on the Drug Abuse Screening Test-10 and/or a hazardous or active alcohol use disorder defined as at least 7 for men and at least 5 for women on the Alcohol Use Disorders Identification  Inclusion based on specific population characteristics: young adults  32.3% women; mean age 28.43; mean cpd 14.33; FTND not measured but time to first cigarette was measured categorically. The mode category was 6 to 30 mins (39/99; 41.5%). Smoking behavioural dependence scale (11 items): mode category ‘Moderate’ (51/99; 51.5%)  Motivated to quit: readiness to quit (1 to 10 scale, 1 to 8 apply to current people who smoke): 5.57 ± 1.49  E-cigarette use at baseline: no use of e-cigs in past 14 days (eligibility criterion) | |
| Interventions | **EC: Cig-a-like**  E-cigarette details:  3 weeks of disposable 4.5% nicotine NJOY, King Bold (NJOY, Inc, Scottsdale, AZ) which resemble conventional cigarettes. NJOY also manufactured the non-nicotine placebo EC. Both nicotine and placebo ECs were tobacco-flavoured. The products were purchased by the investigators and provided to the participants free of charge.  Other stop-smoking pharmacotherapies: none  Behavioural support:  Prior to receiving the ECs, participants were required to complete a 20- to 30-minute telephone counselling session with a trained tobacco cessation Counsellor. The purpose of the telephone counselling was to review current smoking patterns and offer behavioural and environmental change strategies. These included specific smoking reduction options, such as eliminating cigarettes at work and in the home, carrying only those cigarettes needed for that day, dropping cigarettes associated with less intense triggers first, avoiding smoking triggers, and other strategies to manage urges. 18 participants were asked to reduce the number of cigarettes smoked daily by at least 50% of the total number of cigarettes smoked per day at baseline. To mimic real-life EC use, minimum EC use instruction was provided. Participants were encouraged to replace cigarettes with as much or as little use of an EC as needed in order to reduce nicotine withdrawal symptoms. | |
| Outcomes | Week 1, 3  Cessation: not applicable  Adverse events and biomarkers: adverse events and symptoms related to EC use  Other outcomes measured:   - Self-reported reduction of at least 50% in the number of cpd - Percentage reduction in number of cpd - Use of ECs - Satisfaction with ECs | |
| Study funding | “This work was supported by the National Center for Advancing Translational Sciences at the National Institutes of Health (grant number UL1TR000038).” | |
| Author declarations | “None declared” | |
| Notes | Study listed as ongoing study NCT02628964 in the 2016 review update | |
| ***Risk of bias*** | | |
| **Bias** | **Authors' judgement** | **Support for judgement** |
| Random sequence generation (selection bias) | Low risk | Quote: “computer generated” |
| Allocation concealment (selection bias) | Unclear risk | Quote: “…was concealed from research assistants. Blinding of the allocation of nicotine or placebo EC was ensured by the identical appearance of the ECs”. However, not enough information given on how allocation was concealed at the point of randomization |
| Blinding of participants and personnel (performance bias) All outcomes | Low risk | Quote: “Blinding of the allocation of nicotine or placebo EC was ensured by the identical appearance of the ECs”. |
| Blinding of outcome assessment (detection bias) All outcomes | Low risk | Quote: "Blinding of the allocation of nicotine or placebo EC was ensured by the identical appearance of the ECs”. |
| Incomplete outcome data (attrition bias) All outcomes | Low risk | Nicotine EC lost to follow-up: 10/50; placebo EC lost to follow-up: 10/49 |
| Selective reporting (reporting bias) | Low risk | All expected outcomes reported |

Tuisku 2024

| ***Study characteristics*** | | |
| Methods | RCT (placebo controlled)  Setting: Northern Finland  Recruitment: Re-contacted previously recruited study participants who were still smoking and willing to quit [2003 to 2009 via local newspaper announcements.] Also recruited other participants via local newspapers announcements in the same area. | |
| Participants | N=458  By study arm: EC= 152; Vareniciline = 153; Placebo = 153  56% female. Mean age 51 (11.6 SD). Mean FTND: EC 5.6 (SD 1.7); Varenicline 5.7 (DS 1.7); Placebo 5.5 (SD 1.8).  Inclusion criteria: aged 25 to 75 years); smoking daily >10years; minimum of 10 cigarettes per day for at least the previous 5 years; exhaled CO level of 15 ppm or greater at the baseline visit; moderate to high nicotine dependence according to the Fagerström Test for Nicotine Dependence or Heaviness of Smoking Index; intent to quit smoking.  Exclusion: Pregnancy. Use of smoking cessation pharmacotherapy or ECs during the past year. Attempt of smoking cessation by using of EC during the past year. Any cancer (at least five healthy follow-up years after stopping the cancer therapy). Instable (ischemic) vascular or heart disease. Recent myocardial infarction in the past three months. High blood pressure (systolic BP> 140mmHg or diastolic 90mmHg at rest). History of major psychiatric depression or other psychiatric conditions and daily use of any psychiatric medicine. Current addiction of alcohol or misuse of substance. Inability to express himself/herself. Known allergy to any of the study medications. Severe allergy or poorly controlled asthma or other pulmonary disease. Epilepsy  EC use at baseline: No  Motivated to quit smoking: Yes  Inclusion based of specific population characteristic: Heavy smokers, 25-75 year old . Middle-aged Heavy Smokers: NCT record title: Efficacy and Safety of E-cigarettes for Smoking Cessation in Middle-aged Heavy Smokers. | |
| Interventions | 1. Nicotine EC + placebo varenicline tablets  2-mL top-fill tank and universal serial bus cable with wall adapter (output DC5V) plus 6 10-mL refills containing 18 mg/mL of nicotine. Use ECs ad libitum during the 12 weeks after the quit day they had chosen on the second week. All ECs cigarette-like flavour.  Placebo tablets: advised to take placebo tablets at 0.5mg once daily on days 1 to 3, 0.5mg twice daily on days 4 to 7, and subsequently to continue with 1mg twice daily for a total of 12 weeks.  2. Varenicline + non-nicotine ECs  Non-nicotine ECs as above, 0mg nicotine. Varenicline tablets (Champix; Pfizer). Instructions to use as above. The dosing and the duration of varenicline treatment followed the manufacturer’s recommendations.  3. Non-nicotine EC + placebo tablets  Non-nicotine ECs as above, 0mg nicotine. Placebo tablets 0.5mg. Instructions to use as above.  All arms: In addition to the group-specific treatment, all participants were offered 8 motivational interview sessions. | |
| Outcomes | Baseline, 12 weeks, 26 weeks, 52 weeks.  Cessation, CO confirmed. (7-day point prevalence verified by exhaled-CO<10ppm). We used an exhaled CO level of less than 10 parts per million as the cut off value to confirm abstinence from cigarette smoking.  AEs, SAEs. | |
| Study funding | The trial was funded by GRAND with financial support from Pfizer. The award selection process is independent of Pfizer and was supervised by an independent GRAND committee comprising international researchers in the field. Electronic cigarettes were purchased with the grant from a local commercial enterprise. Varenicline and placebo tablets were provided by Pfizer for this trial. | |
| Author declarations | Dr Tuisku received a personal grant from Lapland Central Hospital. Dr Rahkola received grants from the Finnish Medical Foundation and from the Research Foundation of the Pulmonary Diseases.No other disclosures were reported. | |
| Notes | New to 2025 update. | |
| ***Risk of bias*** | | |
| **Bias** | **Authors' judgement** | **Support for judgement** |
| Random sequence generation (selection bias) | Low risk | "Before the first participant’s enrollment, an external statistician from the Medical Informatics and Statistics Research Group in the University of Oulu generated a sequential list of randomized group assignments in a 1:1:1 ratio, with a block size of 9." |
| Allocation concealment (selection bias) | Low risk | "The participants, the 2 nurses making personal contact with them and providing the interventions, and the researchers assessing the outcomes and analyzing the data were all masked to group assignment." |
| Blinding of participants and personnel (performance bias) All outcomes | Low risk | "The participants, the 2 nurses making personal contact with them and providing the interventions, and the researchers assessing the outcomes and analyzing the data were all masked to group assignment. The varenicline and placebo tablets in this trial appeared identical. Nicotine containing EC and nicotine-free ECs had a cigarette-like flavor and an identical appearance, and EC refills were masked with identical taping." |
| Blinding of outcome assessment (detection bias) All outcomes | Low risk | “The researchers assessing the outcomes and analyzing the data were all masked to group assignment.” The Intensity of the interventions was similar across groups. |
| Incomplete outcome data (attrition bias) All outcomes | Low risk | Retention to 52 weeks: 111/152 (73%); 120/153 (78.4%); 120/153 (78.4%) |
| Selective reporting (reporting bias) | Low risk | Reported as set out in NCT record. |

Valentine 2018

| ***Study characteristics*** | | |
| Methods | Design: open-label prospective cohort study  Recruitment: recruited from within the Department of Veterans Affairs (VA) Connecticut Healthcare System by word of mouth  Setting: receiving psychiatric services from Department of Veterans Affairs healthcare system, USA  Study start date/study end date: not specified | |
| Participants | Total N: 50 (sample analyzed for primary outcomes on week 1 completers – N = 43)  Inclusion criteria: no immediate intention to stop smoking; smoking history of ≥ 5 CPD for the past year  Exclusion criteria: current untreated medical or psychiatric or substance use disorders, or both, as determined by a review of the veteran’s electronic medical record; current use of nicotine replacement or other cessation pharmacotherapies; use of e-cigarettes or smokeless tobacco products for more than 2 of the past 30 days  Inclusion based on specific population characteristics: military veteran people who smoke who had no immediate intention to stop smoking and were currently receiving psychiatric services from the Department of Veterans Affairs healthcare system  7% women; mean age 56.9; mean cpd 16.6; mean FTND 4.9  Motivated to quit: had no immediate intention to stop smoking  E-cigarette use at baseline: E-cigarettes or smokeless tobacco products may have been used for less than 2 of the past 30 days. | |
| Interventions | **EC: Refillable**  All given eVic Supreme (Joyetech), "a commercial, variable-power, tank-type device". 6.5 mL tank (Delta 23, Joyetech) and a C3 triple coil atomizer head (Joyetech) with a total resistance of 1.8 ohms. Participants could choose flavour (menthol or tobacco) and nicotine concentration (12 or 24 mg/mL).  Participants taught how to use EC, with additional materials dispensed as needed. Participants were informed that they could use the study e-cigarette or regular tobacco cigarettes, or both, ad libitum during study participation. | |
| Outcomes | Week 1, 2, 3, 4, 8 (weekly lab visits and 1-month follow-up)  Adverse events and biomarkers: Alveolar (breath) CO levels (ppm)  Other outcomes measured:   - Number of cpd - The frequency of e-cigarette use (mean days/week) - The amount of money spent on combustible cigarettes (US dollars/week) - Fagerström Test of Nicotine Dependence - Contemplation Ladder - E-cigarette questionnaire (assessed changes in perceptions about e-cigarettes (e.g. harmfulness, benefits, cost), motivations to use (or not use) them, and the reasons for e-cigarette or combustible cigarette preferences) (measured at baseline and follow-up) - Cotinine | |
| Study funding | "This research was supported by the New England Mental Illness Research, Education and Clinical Center and the U.S. Department of Veterans Affairs. Statistical analyses, biochemical assays, and analyses of e-cigarette solutions were supported by the Administrative and Laboratory cores of P50DA036151 (Yale TCORS) from the National Institutes of Health and the U.S. Food and Drug Administration Center for Tobacco Products. The content is solely the responsibility of the authors and does not necessarily represent the official views of the National Institutes of Health or of the U.S. Food and Drug Administration." | |
| Author declarations | "Ralitza Gueorguivea, PhD, discloses consulting fees for Palo Alto Health Sciences and Mathematica Policy Research and a provisional patent submission by Yale University: Chekroud, A. M., Gueorguieva, R., & Krystal, K. H. “Treatment Selection for Major Depressive Disorder” (filing date June 3, 2016, USPTO docket number Y0087.70116US00). The authors report no other financial relationships with commercial interests." | |
| Notes | New for 2020 update | |
| ***Risk of bias*** | | |
| **Bias** | **Authors' judgement** | **Support for judgement** |
| Random sequence generation (selection bias) | High risk | Uncontrolled cohort study |
| Allocation concealment (selection bias) | High risk | Uncontrolled cohort study |
| Incomplete outcome data (attrition bias) All outcomes | Unclear risk | Follow-up: 31/50 at week 8 |
| Selective reporting (reporting bias) | Unclear risk | No protocol or clinical trial record |

Van Staden 2013\*

|  |  |  |
| --- | --- | --- |
| ***Study characteristics*** | | |
| Methods | Design: single-group, within-subject design  Recruitment: participants from a military hospital in South Africa  Setting: South Africa  Study start date/end date: not specified | |
| Participants | Total N: 15, mean age 38 years, smoked 20 cpd (range 10 to 30), for an average of 17 years (range 5 to 27)  Total N: 13 completed the study (5 women)  Inclusion criteria: adults who smoke daily, ≥ 10 cpd  Exclusion criteria: history of lung disease  Inclusion based on specific population characteristics: no  Motivated to quit: not specified  E-cigarette use at baseline: not specified | |
| Interventions | **EC: Cig-a-like**  Participants were asked to use an EC only for 2 weeks (i.e. no cigarettes).  EC: 'Twisp eGo' cartridge 0.8 mL containing 0.0144 mg of nicotine | |
| Outcomes | The following measurements were taken at baseline and 2-week follow-up:   - Blood pressure and pulse - Arterial and venous COHb and blood oxygen saturation | |
| Study funding | "We are grateful for the sponsorship of the eGo e-cigarette packs by Twisp and also for the valuable advice and laboratory assistance given by Col. (Dr) J Lubbe, Chemical Pathologist, 1 Military Hospital, Pretoria with regard to the measurement of the cotinine levels. We also wish to acknowledge Professor Martin Veller for his insightful contributions during the preparation of this manuscript and also Dr Richard van Zyl-Smith for his assistance and review." | |
| Author declarations | "The sponsor of the Twisp e-cigarette had no role in the design and conduction; the collection, analysis and interpretation of the study; or in the preparation, review or approval of the manuscript." | |
| Notes | Dropouts (N = 2) were due to illness (headache and fever) and undertaking a military course associated with high stress and exposure to others' smoking, making it difficult to abstain from cigarettes.  The paper stated that the EC cartridge contained 0.8 mL of solution with 0.0144 mg of nicotine. This would be an unusually low concentration of nicotine, and we have assumed an error in units where milligrams should have been grams (0.0144 grams of nicotine would make the concentration 18 mg/mL). | |
| ***Risk of bias*** | | |
| **Bias** | **Authors' judgement** | **Support for judgement** |
| Random sequence generation (selection bias) | High risk | Prospective cohort |
| Allocation concealment (selection bias) | High risk | Not randomized |
| Incomplete outcome data (attrition bias) All outcomes | Low risk | 2/15 lost to follow-up |
| Selective reporting (reporting bias) | Unclear risk | Unable to determine prespecified outcomes |

Vickerman 2022

|  |  |  |
| --- | --- | --- |
| ***Study characteristics*** | | |
| Methods | Design: RCT  Recruitment: Participants were recruited from incoming callers to the Oklahoma Tobacco Helpline (OTH) between November 2018 and March 2020 who were smoking and had used e-cigarettes in the past 30 days. The OTH is a free, public health resource offering tobacco cessation services via phone, web, text, and/ or print materials, as well as NRT.  Setting: Seattle, Washington, USA  Study start date: May 2018; study end date: October 2020 | |
| Participants | Total: N = 110  55 in arm 1: enhanced e-cigarette coaching (EEC)  54 in arm 2: quitline treatment-as-usual (TAU)  Dual users of EC and combustible cigarettes  Inclusion criteria: 1) Requested quitline coaching services and enrolled in 5-call Oklahoma Tobacco Helpline (OKHL) programme, smoked at ≥ 1 cigarette per day (CPD), 2) using EC or planning to use EC, 3) willing to quit cigarettes, 4) consented to receive automated phone outreach via the quitline, 5) Android smartphone, regular access to email, 6) 18+ years  Exclusion criteria: 1) schizophrenia, heart attack, stroke or heart condition in past 2 weeks, 2) taking varenicline or bupropion, 3) pregnancy  Female 61.5%; mean age 40.6 (SD 13.5); mean CPD 19.2 (SD 11.3)  E-cigarette use at baseline: yes  Motivated to quit: yes | |
| Interventions | No EC intervention. Advice only  Arm 1: Enhanced e-cigarette coaching (EEC) [Quitline (NRT available) + EC advice (no EC intervention)]  The EEC protocol added 4 intervention components to standard evidence-based quitline treatment: EC education, a shared decision-making model (SDM) for quit plan development (offering selection of NRT, EC, both NRT and EC, or no nicotine replacement aid; in these conversations, coaches discussed EC as a quitting tool similar to NRT and as an alternative form of nicotine replacement), behavioural support tailored to the selected quit plan, and a requirement for coaches to assess and address EC use on every call.  Arm 2 : Quitline treatment-as-usual (TAU) (NRT available)  The TAU protocol included recommending participants stop both cigarettes and e-cigarettes on their quit date when they start NRT.  Both arms:  Standard benefits available through the OTH (2 to 8 weeks of patch, gum, and/or lozenge)  The quitline intervention focused on 5 key strategies for quitting: committing to a quit date, coping with urges, using medications effectively, disposing of tobacco paraphernalia, and utilizing social support.  The 5 coaching calls for the 2 groups were delivered over approx. 2 months, with call 1 intended to take approx. 20 to 25 mins, and later calls to take approx. 15 mins. Participants downloaded a smartphone app to complete daily questionnaires on product use for 12 weeks. Participants received USD 20 for completing the baseline survey and up to USD 110 for daily questionnaires. | |
| Outcomes | Cessation 3 months (CO confirmed)  AEs  Continued EC use | |
| Study funding | This work was supported by the National Institute on Drug Abuse (R21DA042960 to KAV) and used the InsightTM mHealth Platform and Android smartphone app, which was supported by the National Cancer Institute Cancer Center Support Grant P30CA225520 awarded to the University of Oklahoma Stephenson Cancer Center. The content is solely the responsibility of the authors and does not necessarily represent the official views of the National Institutes of Health or Optum. | |
| Author declarations | KAV, KMC, LNM, JMH, and KAW are employees of Optum, the provider of quitline services for the Oklahoma Helpline in this study. THB is on the Advisory Board of Hava Health, Inc., a start-up (with no connections to the tobacco industry) that is developing a therapeutic e-cigarette. All other authors declared no conflicts of interest. | |
| Notes | New to 2022 update | |
| ***Risk of bias*** | | |
| **Bias** | **Authors' judgement** | **Support for judgement** |
| Random sequence generation (selection bias) | Low risk | Quote: “participants were then randomized to receive quitline treatment as usual (TAU) or EEC, using blocked randomization stratified by gender (male vs female; other response options for gender were not included for the quitline at the time of this trial, but have since been added) with an allocation ratio of 1:1. The quitline participant record software accessed a randomization table to automatically assign a participant to a group after a coach clicked a randomize button". |
| Allocation concealment (selection bias) | Low risk | As above |
| Blinding of participants and personnel (performance bias) All outcomes | Unclear risk | No detail |
| Blinding of outcome assessment (detection bias) All outcomes | Low risk | CO measured |
| Incomplete outcome data (attrition bias) All outcomes | Low risk | EEC: 34/55 (46)  TAU: 32/54 (50) |
| Selective reporting (reporting bias) | Low risk | Outcomes from NCT record reported |

Vojjala 2025

|  |  |  |
| --- | --- | --- |
| ***Study characteristics*** | | |
| Methods | RCT  Setting: USA | |
| Participants | 121 participants randomised  EC intervention = 63; NRT standard of care =58  54% female; mean age 58; average 18 CPD (+/- 10)  59% had COPD, 33% had asthma, and 24% had CAD/PAD, with 16% having a combination of two or more conditions.  Motivated to quit smoking: yes. On a scale of 1–10, mean ± SD motivation to quit smoking among participants was 9 ± 2 and confidence to quit smoking was 6 ± 2.  Inclusion criteria: An ambulatory ICD-10 code for COPD in the last 12 months and a COPD Assessment Tool (CAT) score on the screening ≥ 10; aged 21 to 75; current CC user; motivated to quit smoking (at least a 5 on a 10-point Likert scale)  Exclusion criteria: A CAT score > 30 representing severe COPD; pregnancy; diagnosis of any medical condition; reporting using NRTs or e-cigarettes within the last 30 days | |
| Interventions | EC: NJOY daily e-cigarettes  Arm 1: EC + Counselling  E-cigarette (EC). NJOY daily e-cigarettes are self-contained and non-refillable. Each DAILY provides approximately 300 puffs, comparable to a full pack of cigarettes.  Behavioural: Smoking harm reduction counselling sessions  Both study arms received six counseling sessions delivered by telephone with a licensed mental health counselor trained in motivational interviewing.and smoking cessation treatment. Counselling to cover health education, social support issues, and motivational enhancement to improve self-efficacy while addressing other aspects know to contribute to smoking among people with COPD (e.g. tips on dealing with depression)  Arm 2: Nicotine Replacement Therapy (NRT)  Participants in the NRT arm will receive 21 mg nicotine patch (for those with cpd ≥ 20) or 14 mg nicotine patch (for those with cpd < 20) + 4 mg nicotine gum.  Behavioural: Smoking harm reduction counselling sessions as for Arm 1 | |
| Outcomes | Baseline, 3 months, 6 months  Primary outcome measures:  Number of participants who achieve 50% reduction in cigarettes per day (cpd)  Average change in score on the mMRC Dyspnoea Scale  Secondary outcome measures:  Change in score of COPD Assessment Test  Change in score of Clinical COPD Questionnaire  Number of participants who reported satisfaction with use of ECs  Number of participants who reported additional use of tobacco products and/or marijuana.  Abstinence reported. | |
| Study funding | Funding was received from internal funds provided to Dr. Scott Sherman by New York University (NYU) Grossman School of Medicine. The study sponsor had no role in the study design; collection, analysis, and interpretation of data; writing the report; or the decision to submit the report for publication. The NYU Langone Health IRB approved the study (#s20-00839). | |
| Author declarations | The authors have no conflicts of interest to declare. The study sponsor had no role in the study design; collection, analysis, and interpretation of data; writing the report; or the decision to submit the report for publication. | |
| Notes | NCT04465318 new to 2022 update.  Moved from ongoing to included (Vojjala 2024) in 2025 | |
| ***Risk of bias*** | | |
| **Bias** | **Authors' judgement** | **Support for judgement** |
| Random sequence generation (selection bias) | Low risk | Participants were randomized into one of two study arms, counseling with standard care (NRT) or counseling with inter vention (EC), using a minimization strategy. |
| Allocation concealment (selection bias) | High risk | Not reported for study staff “Participants were not blinded to their randomization allocation.” “open-label RCT pilot” |
| Blinding of participants and personnel (performance bias) All outcomes | Low risk | Equally intensive. |
| Blinding of outcome assessment (detection bias) All outcomes | High risk | Abstinence self-reported |
| Incomplete outcome data (attrition bias) All outcomes | Low risk | EC arm 40/63 at 6 mths = 63%; NRT arm 31/58 at 6 mths = 53.5% |
| Selective reporting (reporting bias) | Low risk | Measures in NCT record reported: COPD (CAT), CPD, Asthma severity. |

Wadia 2016

|  |  |  |
| --- | --- | --- |
| ***Study characteristics*** | | |
| Methods | Design: uncontrolled experimental study  Recruitment: dental hospital staff were recruited – not specified how  Setting: dental hospital, UK  Study start date: April 2015; study end date: December 2015 | |
| Participants | Total N: 20 (18 of the 20 attended the reassessment visit)  Inclusion criteria: 18 to 65 years; systemically healthy; smoked ≥ 10 CPD for at least 5 years; ≥ 24 natural teeth (excluding third molars) and had no probing pocket depths over 4 mm at any site; did not wish to quit  Exclusion criteria: systemic condition known to exacerbate or modulate periodontitis (for example, diabetes); antibiotics in the previous 3 months; anti-inflammatory drugs or other medication likely to affect the periodontal tissues were taken routinely; pregnancy/breastfeeding  Percentage of women in study, age, cpd and FTND: not specified  Motivated to quit: enrolled people who smoke who did not intend to quit smoking, but were prepared to attempt to substitute smoking with the use of e-cigarettes for 2 weeks  E-cigarette use at baseline: not specified | |
| Interventions | **EC: Refillable**  Participants provided with a blu PROTM e-cigarette kit (Electric Tobacconist^®^), an extra bottle of blu PRO Tobacco™ e-Liquid (Electric Tobacconist) and written instructions. The e-Liquid was Classic Tobacco-flavoured and contained 18 mg of nicotine (medium strength). The participants agreed to substitute their regular smoking habits with the use of e-cigarettes for 2 weeks. They were asked to make a note of any cigarette smoking during the 2 weeks if complete abstinence was unsuccessful. | |
| Outcomes | 2 weeks  Adverse events and biomarkers: adverse effects  Other outcomes measured:   - Cigarette use - Dental outcomes | |
| Study funding | Not specified | |
| Author declarations | Not specified | |
| ***Risk of bias*** | | |
| **Bias** | **Authors' judgement** | **Support for judgement** |
| Random sequence generation (selection bias) | High risk | No randomization |
| Allocation concealment (selection bias) | High risk | No randomization |
| Incomplete outcome data (attrition bias) All outcomes | Low risk | 2 lost to follow-up |
| Selective reporting (reporting bias) | Low risk | All expected outcomes reported |

Wagener 2023

|  |  |  |
| --- | --- | --- |
| ***Study characteristics*** | | |
| Methods | Design: RCT  Setting: Ohio State University Comprehensive Cancer Center, USA  Recruitment: recruit and enrol recent smoking cessation treatment failures from a state QL  Study start date: 15 October 2020. Primary completion date: 31 January 2023. | |
| Participants | N = 350  EC = 175  Quitline treatment as usual (NRT) = 175  Participants: Quitline recent treatment failure  % female 61.3; age 54.72; CPD 17.10 (8.18)  Predominately serves priority populations (e.g. low socioeconomic status, high levels of mental health conditions)  Inclusion criteria: smoke ≥ 5 cigarettes per day for the past year; report at least minimal interest in switching to an alternative product (> "not at all" on a Likert scale); participation in the Oklahoma Tobacco Helpline or South Carolina Tobacco Helpline within the last 4 to 7 months  Exclusion criteria: < 21 years old; report NRT use or making a quit attempt within the last 7 days; current daily use of an e-cigarette over last month; unstable or significant medical condition or psychiatric conditions; pregnant/breastfeeding | |
| Interventions | EC: JUUL pod  Experimental: E-cigarette. 5% nicotine JUUL e-cigarette (tobacco or menthol flavour). Will also receive counselling  Active comparator: Quitline Treatment-As-Usual. NRT consisting of nicotine patches and lozenges along with standard assessments. Will also receive counselling.  All participants will receive 3 calls from QL coaches and EC and NRT will be provided at no cost for 8 weeks. The final follow-up will occur 12-weeks post baseline. | |
| Outcomes | Baseline, 12 weeks  Smoking behaviour, product use patterns and continued use, and carbon monoxide, AEs, SAEs.  Self-reported 7-day point prevalence abstinence from cigarettes (time frame: daily for 12 weeks), carbon monoxide validated  At 12 weeks:  Minnesota Nicotine Withdrawal Scale  Tiffany-Drobes Questionnaire of Smoking Urges: Brief Form (QSU)  Cigarette/EC Dependency Scale  Tobacco Use History Questionnaire-Years of Smoking  Change in motivation rulers  Drug effects/liking Questionnaire  Cigarette purchase task - breakpoint  Change in motivation rulers (scale) from baseline to week 12 | |
| Study funding | Ohio State University Comprehensive Cancer Center  National Institute on Drug Abuse (NIDA) | |
| ***Risk of bias*** | | |
| **Bias** | **Authors' judgement** | **Support for judgement** |
| Random sequence generation (selection bias) | Unclear risk | NCT record and SRNT abstract - no detail provided |
| Allocation concealment (selection bias) | Unclear risk | NCT record and SRNT abstract - no detail provided |
| Blinding of participants and personnel (performance bias) All outcomes | Unclear risk | NCT record and SRNT abstract - no detail provided |
| Blinding of outcome assessment (detection bias) All outcomes | Unclear risk | NCT record and SRNT abstract - no detail provided |
| Incomplete outcome data (attrition bias) All outcomes | Unclear risk | NCT record and SRNT abstract - no detail provided |
| Selective reporting (reporting bias) | Unclear risk | NCT record and SRNT abstract - no detail provided |
| Other bias | Unclear risk | NCT record and SRNT abstract - no detail provided |

Walele 2018\*

|  |  |  |
| --- | --- | --- |
| ***Study characteristics*** | | |
| Methods | Design: RCT (short-term, Cravo 2016) followed by cohort study (Walele 2018\*) in which all participants were given nicotine EC  Recruitment: community  Setting: 2 centres in the UK (Covance Clinical Research Unit Ltd, Leeds and Simbec Research Ltd, Wales)  Study start date: December 2013; study end date: December 2016 | |
| Participants | Two study phases.  Inclusion criteria differ per study phase:  Cravo 2016 (short-term RCT), inclusion: 21 to 65 years; BMI 18 to 35 kg/m^2^; 5 to 30 CPD for ≥ 1 year (self-reported); in good health (determined by medical history, a physical examination, a 12-lead ECG, lung function tests and clinical laboratory evaluations); people who smoke (urinary cotinine ≥ 3 and exhaled CO ≥ 6 ppm)  Additional criteria for Walele 2018 (participants from Cravo 2016):  Participants assessed by PI as being compliant in Cravo 2016 (e.g. having attended outpatient visits and having been compliant with study procedures)  Participants had to be willing to use the study product as the only nicotine-containing product for the duration of the study, and, as deemed by PI, had to have no clinically significant abnormalities in 12-lead electrocardiogram, vital signs, spirometry and clinical laboratory assessments in the preceding study.  In addition, participants who were assigned to the conventional cigarette (CC arm) in Cravo 2016 had to be established people who smoke CCs, which was assessed by urinary cotinine levels (a score of 3 and above on a NicAlert™ test strip was considered positive), eCO levels (a readout > 6 ppm was considered positive) and by review of a smoking history questionnaire.  Exclusion criteria:  Cravo 2016: Use of NRT, snuff or chewing tobacco in 14 days previous, or intended to use during study; trying to stop smoking or considering quitting; clinically-significant illness or disorder, history of drug or alcohol abuse within 2 years prior to study start; woman of “childbearing potential” unwilling to use “acceptable contraceptive measure” during study.  Walele 2018 (participants from Cravo 2016): people who had taken or received any form of NRT, snuff or chewing tobacco during the previous study or intended to use it during this study; relevant illness history; history of drug or alcohol abuse; lung function test or vital signs considered unsuitable; trying to stop smoking; women who are pregnant, or unwilling to use acceptable contraceptive method for the duration of the study  **Cravo 2016**  Total N: 419 randomized, 408 analyzed (excludes 11 who were excluded prior to any product use)  N per arm: EVP: 306; control: 102  45% women; mean age 34.6; mean cpd: most 11 to 20 cpd (56% intervention, 62% control); mean FTND: most moderate (57% intervention, 54% control)  Motivated to quit: no  E-cigarette use at baseline: not excluded based on prior EC use  **Walele 2018**  Total N: 209 (147 pre-EVP group; 62 pre-CC group)  45% women; mean age 36.6; mean cpd 2.6 (data from figure): not reported; FTND: not reported; motivated to quit: as reported for Cravo 2016  E-cigarette use at baseline: not reported | |
| Interventions | **EC: Cig-a-like**  **Cravo 2016**  **EC**: EVP prototype (2.0% nicotine), developed by Fontem Ventures B.V. (Amsterdam, the Netherlands). Instructed to only use EVP for study period. It consisted of a rechargeable battery (voltage range of 3.0e4.2 V), an atomiser and a capsule (small cartridge) containing e-liquid. The capsules were replaceable and the battery and atomiser were reusable. Could choose between 2 different e-liquids, which differed solely in their flavour: a menthol-flavoured e-liquid with 2.0% nicotine (2.7 mg nicotine/capsule) and a tobacco-flavoured e-liquid with 2.0% nicotine (2.7 mg nicotine/capsule)  **Control**: used their own usual conventional cigarette brand  **Walele 2018**  E-cigarette details: commercially available Puritane™ (closed system EVP) consists of a lithium-ion rechargeable battery and a replaceable cartomizer comprising of an e-liquid reservoir pre-filled by the manufacturer, a heating element and a mouthpiece; 1.6% nicotine (16 mg/g). Available in tobacco or menthol. 2 weeks before baseline, participants had a familiarization session with Puritane™, where they could see and try the EVP. | |
| Outcomes | **Cravo 2016:** weeks 1, 2, 4, 6, 8, 10, and 12  **Walele 2018:** starting on the last day of the previous trial: months 1, 2, 3, 6, 9, 12, 15, 18, 21, and 24  Study centre visits for assessments  Adverse events and biomarkers:   - “adverse events” (coded using Medical Dictionary for Regulatory Activities version 16.1, 2013, collected via diary cards and questionnaires) - Vital signs (systolic and diastolic blood pressure, pulse rate and oral temperature) - Lung function (FEV, FEF, PEF) - Urine biomarkers (nicotine equivalents (NEQs: nicotine, cotinine, nicotine-N-glucuronide, cotinine-Nglucuronide, trans 3’-hydroxycotinine and trans 3’-hydroxycotinine glucuronide); S-PMA; 3-HPMA; PG; total NNAL (NNAL þ NNAL-glucuronide)); exhaled CO - Blood COHb   Other outcomes measured:   - Number of conventional cigarettes smoked - EVP capsules used - ECG (categorized them as normal, abnormal-not clinically significant (NCS) or abnormal-clinically significant (CS)) - MWS-R (revised Minnesota Nicotine Withdrawal Scale) - QSUBrief (Brief Questionnaire of Smoking Urges) questionnaires - Clinical chemistry (blood levels of aspartate aminotransferase (AST), alanine aminotransferase (ALT), alkaline phosphatase, gamma-glutamyl transferase (GGT), sodium, potassium, chloride, calcium, inorganic phosphate, glucose, urea nitrogen (BUN), total bilirubin, creatinine, total protein, albumin, cholesterol (HDL, LDL, and total)); clinical haematology (white blood cell count (WBC), red blood cell count (RBC), haemoglobin, haematocrit (PCV), mean cell volume (MCV), mean cell haemoglobin (MCH), mean cell haemoglobin concentration (MCHC), platelet count, differential WBC);urinalysis (pH, protein, glucose, ketones, urobilinogen, blood and specific gravity) | |
| Study funding | **Cravo 2016**  "This work was funded and supported by Fontem Ventures B.V. Imperial Brands plc is the parent company of Fontem Ventures B.V. the manufacturer of the EVP prototype used in this study".  **Walele 2018**  "This work was funded and supported by Fontem Ventures B.V. Imperial Brands Group plc is the parent company of Fontem Ventures B.V., the manufacturer of the EVP used in this study". | |
| Author declarations | **Cravo 2016**  "Dr. Cravo has nothing to disclose. Mrs Martin reports personal fees from Fontem Ventures B.V. during the conduct of the study; personal fees from tobacco and pharmaceutical industries outside the submitted work. Dr. Sharma reports others from Fontem Ventures B.V. during the conduct of the study. Dr. Bush reports others from Fontem Ventures B.V. during the conduct of the study. Mrs Savioz reports personal fees from Fontem Ventures B.V. during the conduct of the study; personal fees from tobacco and pharmaceutical industries outside the submitted work. Mr Craige has nothing to disclose. Mr Walele has nothing to disclose."  **Walele 2018** (copied from Transparency documents)  "Dr. Koch reports others from Fontem Ventures B.V., during the conduct of the study; Dr. Martin reports personal fees from Fontem Ventures B.V., during the conduct of the study; personal fees from tobacco and pharmaceutical industries, outside the submitted work; Dr. O'Connell has nothing to disclose. Dr. Bush reports others from Fontem Ventures B.V., during the conduct of the study; Dr. Savioz reports personal fees from Fontem Ventures B.V., during the conduct of the study; personal fees from tobacco and pharmaceutical industries, outside the submitted work; Dr. Walele has nothing to disclose." | |
| Notes | Sponsor: Imperial Tobacco Group PLC  Study listed as ongoing studies NCT02029196 and NCT02143310 in 2016 review update. Treated as single study in this review due to including the same participants, and no time lag between studies.  "The same subjects who participated in our previous clinical trial (ClinicalTrials.gov, #NCT02029196) conducted in the same centres, with another EVP (Cravo 2016), were invited to participate in the study by Walele 2018\*. All volunteering subjects were assigned to switch to using Puritane™, a closed system EVP, for two years, starting on the last day of the previous trial (End of Study [EoS] visit), which corresponded to the baseline visit of Walele 2018\*." | |
| ***Risk of bias*** | | |
| **Bias** | **Authors' judgement** | **Support for judgement** |
| Random sequence generation (selection bias) | Low risk | Quote: “Randomisation was performed using an Interactive Web Response System (IWRS; Almac Clinical Technologies)”. |
| Allocation concealment (selection bias) | Low risk | Quote: "Randomisation was performed using an Interactive Web Response System (IWRS; Almac Clinical Technologies)”. |
| Blinding of participants and personnel (performance bias) All outcomes | High risk | Open-label, no blinding, differential levels of support/product use so performance bias cannot be ruled out. |
| Blinding of outcome assessment (detection bias) All outcomes | High risk | Open-label, no blinding, with differential levels of support/product use and subjective outcomes |
| Incomplete outcome data (attrition bias) All outcomes | Low risk | Cravo: 286/306 int and 101/102 control completed study but all who received product included in analysis. In EVP group, 14 withdrew consent, 2 experienced AEs, 1 death, 3 “other”. CC group 1 AE  Walele 2018\*: High  209/387 enrolled for study Walele 2018\*. A total of 102 participants (48.8%; EVP: 75/145 (51%); CC: 27/61 (43.5%)) completed the study. |
| Selective reporting (reporting bias) | Low risk | Cravo 2016: low  All anticipated outcomes reported (study registered prior to study completion)  Walele 2018\*: low  All anticipated outcomes reported (study registered prior to study completion) |

Walker 2020

|  |  |  |
| --- | --- | --- |
| ***Study characteristics*** | | |
| Methods | Design: RCT  Recruitment: national media advertising  Setting: community-based, New Zealand  Study start date: recruitment between March 2016; study end date: Aug 2018 | |
| Participants | Total N: 1,124. N per arm: patches-only group: 125; patches plus nicotine e-cigarette group: 500; patches plus nicotine-free e-cigarette group: 499  Inclusion criteria: living in New Zealand; 18 years or older; smoked tobacco (amount not specified); motivated to quit in the next 2 weeks; able to provide verbal consent; prepared to use any of the trial treatments; access to a telephone  Exclusion criteria: pregnancy or breastfeeding; used an EC for smoking cessation for ≥ 1 week any time in the past year; currently using smoking cessation medication; enrolled in another cessation programme or study; history of severe allergies; poorly controlled asthma; cardiovascular event in the 2 weeks before enrolment; only 1 participant per household was permitted.  69% women; mean age 41.6; mean cpd 17.3; mean FTND 5.2  Motivated to quit: yes  E-cigarette use at baseline: not reported but use of an e-cigarette for smoking cessation for more than 1 week any time in the past year was an exclusion criterion. | |
| Interventions | **EC: Refillable**  Moderate-intensity behavioural support was available for all participants immediately after randomization, then once a week for 6 weeks. This support consisted of 10 to 15 mins of withdrawal-oriented behavioural support and advice on using their allocated treatment, delivered proactively over the phone by researchers who had received standardized training in delivery of such support. Assigned to:  1) **Nicotine patch** for 14 weeks, including 2-week prequit. 21 mg, 24-hr nicotine patch (Habitrol)  2) **Nicotine patch and nicotine-free EC** for 14 weeks. As 1, plus 14-week supply at no cost. A 2nd generation eVOD (Kangertech, Shenzhen GuangDong, China) starter kit, with a choice of 1 of 2 tobacco e-liquid flavours. Advised to start using the e-cigarette 2 weeks before their quit date, as and when necessary or desired, and in accordance with the manufacturer’s written instructions, to become familiar with its use. Participants were instructed to stop smoking from their quit date and continue with their allocated treatment for 12 weeks (ad libitum use of the e-cigarette), irrespective of any lapses to smoking.  3) **Nicotine patch and nicotine EC** for 14 weeks. As above, but 18 mg/mL nicotine | |
| Outcomes | Quit date, 1, 3, 6, and 12 months  Continuous abstinence at 6 months with CO validation  Adverse events and biomarkers: known side effects associated with e-cigarette use and nicotine patch use; SAEs  Other outcomes measured:   - Relapse - Self-reported treatment adherence - Tobacco withdrawal symptoms and urge to smoke - Urge to vape - Self-reported weight - Concomitant medication - Treatment cross-over - Use of other smoking cessation support or medication - Continued use of allocated treatment past 14 weeks - Changes in shortness of breath, cough, asthma, COPD, and mental health problems - Belief in ability to quit and remain tobacco-free - Smoking identity and views on their allocated treatment for smoking cessation and whether they would recommend it to other people who smoke who want to quit - In people still smoking at each follow-up call, outcomes were number of cigarettes smoked per day and reduction in smoking. - Participants allocated e-cigarettes were asked about their urge to vape; whether they changed devices or e-liquid, or both; whether they accessed any e-cigarette support. | |
| Study funding | Funding: Health Research Council of New Zealand. "The sponsor of the study had no role in the study design, data collection, data analysis, data interpretation, or writing of the report. The corresponding author had full access to all the data in the study and had final responsibility for the decision to submit for publication." | |
| Author declarations | NW, CB, MV, GL, ML, and VP report grants from the Health Research Council of New Zealand, during the conduct of the study. NW, CB, MV, and VP report grants from Pfizer, outside the submitted work. GL chairs the organization End Smoking New Zealand, which advocates for harm-reduction approaches to tobacco control. E-cigarettes were purchased from a New Zealand e-cigarette online retailer (NZVAPOR, https://www.nzvapor.com/), e-liquid was purchased from Nicopharm, Australia (https://www.nicopharm.com.au/), and nicotine patches were supplied by the New Zealand Government via their contract with Novartis (Sydney, Australia). NZVAPOR also provided, at no cost to participants, on-line and phone support regarding use of the e-cigarettes. Neither NZVAPOR nor Nicopharm have links with the tobacco industry. None of the above parties had any role in the design, conduct, analysis, or interpretation of the trial findings, or writing of this publication. | |
| Notes | Study listed as ongoing study NCT02521662 in the 2016 review update | |
| ***Risk of bias*** | | |
| **Bias** | **Authors' judgement** | **Support for judgement** |
| Random sequence generation (selection bias) | Low risk | Computer-generated randomization sequence |
| Allocation concealment (selection bias) | Low risk | Quote: “We ensured allocation concealment because the statistician who generated the random allocation was not the person randomising participants.” |
| Blinding of participants and personnel (performance bias) All outcomes | High risk | Quote: “Participants and researchers collecting outcome data were masked to the nicotine content of the e-liquid” but those allocated to patch only would be aware they did not have an E-cigarette.  Quote: “Third, while we attempted to minimise detection bias by masking the nicotine content of the e-liquid, we were only 30% successful, and thus some bias in favour of nicotine e-cigarettes could have occurred.” |
| Blinding of outcome assessment (detection bias) All outcomes | Low risk | Biochemical validation |
| Incomplete outcome data (attrition bias) All outcomes | Low risk | < 50% lost to follow-up, similar rates of attrition between groups (within 20%) |
| Selective reporting (reporting bias) | Unclear risk | CO-verified abstinence at 12 months stated as a secondary outcome, but data were not reported in the main text. However, stated in the appendix that too few people in each group were followed up to 12 months (36/1124), so no data were presented for this time point |

White 2022

|  |  |  |
| --- | --- | --- |
| ***Study characteristics*** | | |
| Methods | Design: RCT (2 x 2 x 2 factorial design)  Recruitment:  Setting: Winston-Salem, NC, Philadelphia, PA and the respective surrounding areas, USA  Study start date: 6 August 2018. Study end date: 13 March 2020 | |
| Participants | Total study N: 50  50 were randomized, 32 completed study  Inclusion criteria:  Self-reported smoking 5 to 50 cpd, CO and urinary cotinine confirmed  Reported vaping on at least 2 separate occasions, without an allergic or otherwise adverse experience  18+ years  Exclusion criteria:  Self-reporting frequent use of e-cigarettes (> 15 days in the past month)  Female 40%; mean age 41.6 (SD 11.7); mean CPD 22.8 (SD 10.5); mean FTDN 5.7 (SD 1.8)  E-cigarette use at baseline: 30%  Motivated to quit: NR | |
| Interventions | EC type: **refillable**  EC: Halo Triton (3.7 V battery; 650 mAh) vape pen and compatible Triton 2.4 mL refillable tanks with 2.2-2.4 ohm coils. E-liquid, sourced from Syndicate Distribution (Westchase, Florida, US) in 10 mL bottles; had a base of 70% propylene glycol and 30% vegetable glycerin  Combustible cigarettes: participants received Spectrum research cigarettes matched to their usual brand cigarette menthol preference.  All received study cigarettes and a vape pen with e-liquid to use for 12 weeks.  Study cigarettes (very low nicotine content (0.4 mg/g of tobacco))  Study cigarettes (normal nicotine content (15.8 mg/g of tobacco; double-blind))  Vape pen - E-liquids contained low nicotine (0.3% free-base nicotine) levels of nicotine.  Vape pen - E-liquids contained moderate nicotine (1.8% free-base nicotine; open-label).  Flavours: 3 tobacco flavours (robust tobacco, light tobacco, tobacco/menthol blend), mint, fruit and dessert flavour options (mixed berry, watermelon, berry, cookies and crème, chocolate, caramel, spearmint, peppermint, and menthol)  Arm 1: Normal Nicotine Content (NNC) cigarette + moderate nicotine e-liquid (1.8% free-base nicotine) + tobacco flavours  Arm 2: NNC cigarette + low nicotine e-liquid (0.3% free-base nicotine) + tobacco flavours (0.3% free-base nicotine)  Arm 3: NNC cigarette + moderate nicotine e-liquid + varied flavours  Arm 4: NNC cigarette + low nicotine e-liquid + varied flavours  Arm 5: Very Low Nicotine Content (VLNC) cigarette + moderate nicotine e-liquid + tobacco flavour  Arm 6: VLNC cigarette + low nicotine e-liquid + tobacco flavours  Arm 7: VLNC cigarette + moderate nicotine e-liquid + varied flavours  Arm 8: VLNC cigarette + low nicotine e-liquid + varied flavours  Participants could be compensated up to USD 1070 for completing all study procedures. | |
| Outcomes | Week 1, 2, 3, 4, 6, 8, 10, 12  CPD during week 12 was the primary outcome.  CO up to 12 weeks. Measure of cigarette smoke exposure using data on expired breath CO. CO (ppm) was captured using Covita Smokelyzer devices and standard exhalation procedures.  TCD  N-acetyl-S-(2-cyanoethyl) cysteine (CEMA), the mercapturic acid metabolite of acrylonitrile, which serves as a biomarker of cigarette smoke exposure  Anatabine levels from the spot urine samples to assess whether participants assigned to VLNC cigarettes adhered to only using VLNC cigarettes  Total nicotine equivalents | |
| Study funding | The research reported in this paper was funded by the National Institute on Drug Abuse and the U.S. Food and Drug Administration (FDA) Center for Tobacco Products (U54DA031659, ECD & DKH). Research reported in this publication was supported by National Institutes of Health (NIH) grant P30 CA77598 utilizing the Biostatistics and Bioinformatics Core shared resource of the Masonic Cancer Center, University of Minnesota and by the National Center for Advancing Translational Sciences of the National Institutes of Health Award Number UL1TR0002494. Author support also included NIDA U54DA036105 (COC) and NIDA R36DA054481 (CMW). The content of this article is solely the responsibility of the authors and does not necessarily represent the official views of the NIH or the FDA. | |
| Author declarations | Pre-print. NR | |
| Notes | Study recruitment ended early due to concerns about pending regulations and the availability/relevance of the study vaping products.  New to 2022 review | |
| ***Risk of bias*** | | |
| **Bias** | **Authors' judgement** | **Support for judgement** |
| Random sequence generation (selection bias) | Unclear risk | No detail on how randomization was carried out |
| Allocation concealment (selection bias) | Unclear risk | No detail |
| Blinding of participants and personnel (performance bias) All outcomes | Low risk | Cigarette nicotine content was masked to participants and researchers. |
| Blinding of outcome assessment (detection bias) All outcomes | Low risk | CO measured |
| Incomplete outcome data (attrition bias) All outcomes | High risk | > 50% dropout in 2 arms  At FU: 4/5; 2/6; 4/5; 7/10; 4/7; 5/6; 2/5; 4/6 |
| Selective reporting (reporting bias) | High risk | Outcomes not all reported |

Xu 2023\*

|  |  |  |
| --- | --- | --- |
| ***Study characteristics*** | | |
| Methods | Design: "open-label randomized trial"  Recruitment: random sampling from a specially constructed panel assembled using probabilistic address-based sampling (i.e. by inviting a stratified random sample of all US residential addresses drawn from the US Postal Service’s master file)  Setting: general population, USA  Study start date September 2018. Study end date October 2019 | |
| Participants | Total N: 837  285 – JUUL Virginia tobacco; 281 – JUUL flavour choice; 271 – quit advice group  50.2% female; mean age 45.99 (SD 11.48); mean CPD 18.76 (SD 7.86)  Not motivated to quit.  EC use at baseline: lifetime ENDS ever use 62.4%, 63.8%, 62.8%. Ever-used ENDS fairly regularly 15.6%.  Inclusion criteria: (a) age 21 to 65 years; (b) smoked ≥ 100 cigarettes in lifetime; (c) smoke cigarettes daily and smoke ≥ 10 cigarettes/day; (d) willing to attempt to substitute use of ENDS (and JUUL specifically) for some cigarette smoking (“Would you be willing to try using JUUL e-cigarettes in place of smoking at least some regular cigarettes for 6 months?”)  Exclusion criteria: (a) use of ENDS or smoking cessation medications (e.g. NRT, bupropion, varenicline), or behavioural smoking-cessation counselling in the past 30 days; (b) serious medical or psychiatric condition; (c) living with a study enrollee; (d) participation in another clinical study in the past 30 days; (e) employed by or related to an employee of Juul Labs, Inc. or Pax Labs, Inc.; or (f) pregnancy or breastfeeding | |
| Interventions | EC arms: JUUL products were provided to JUUL group participants for up to 6 months; a 12-month follow-up was completed 6 months later.  Participants in the JUUL groups initially received two JUUL devices, a Universal Serial Bus charging dock and the instructions for use included with purchase, as well as 2 to 5 packs of pods (4 pods per pack, 5.0% nicotine by weight (59 mg/mL)), based on baseline cigarette consumption. Participants could order up to 15 four-packs of pods and additional devices each month for the first 6 months.  The Virginia Tobacco group was only provided Virginia Tobacco pods.  The flavour choice group could order their preferred flavour(s) available at time of study (Virginia Tobacco, Classic Tobacco, Mint, Menthol, Mango, Creme, Fruit, and Cucumber).  Comparator arm: Quit Advice. Printed materials explaining why quitting smoking is important and behavioural advice about how to quit smoking (Centers for Disease Control and Prevention (CDC) and https://Smokefree.gov about reasons to quit smoking and means of cessation) and given contact information for smoking-cessation services. This was meant to represent basic advice to adults who smoke and were not necessarily ready to quit or seek treatment. | |
| Outcomes | Baseline, 1, 3, 6, 12 months  Cessation at 6 and 12 months  AEs and SAEs were not assessed by a medical monitor in this study and were not reported.  EC use at FU  Flavour use in past 30 days | |
| Study funding | This study was funded by Juul Labs, Inc. | |
| Author declarations | Yingying Xu was a full-time employee of Juul Labs, Inc. when the article was drafted. Nicholas I. Goldenson is a full-time employee of Juul Labs, Inc. Shivaani Prakash was a full-time employee of Juul Labs, Inc. when the article was drafted. Erik M. Augustson was a full-time employee of Juul Labs, Inc. when the article was drafted. Saul Shiffman is a senior advisor to PinneyAssociates, Inc, through which he provides consulting services on tobacco harm reduction on an exclusive basis to Juul Labs, Inc. The Centre for Substance Use Research (CSUR; https://www.csures.com) developed the study instruments and protocol. Yingying Xu, Shivaani Prakash, and Erik M. Augustson was a full-time employee of Juul Labs, Inc., during the time that this study was conducted and article was drafted. | |
| Notes | New to 2024 update | |
| ***Risk of bias*** | | |
| **Bias** | **Authors' judgement** | **Support for judgement** |
| Random sequence generation (selection bias) | Low risk | Participants were randomized in equal numbers to the 3 study groups (JUUL Virginia Tobacco vs JUUL flavour choice vs Quit Advice) via a blocked randomization scheme (within 36 strata defined by age, gender, race/ethnicity, and educational attainment, to ensure balanced allocation) using a random number generator in Statistical Package for the Social Sciences. |
| Allocation concealment (selection bias) | Low risk | Neither participants nor study staff knew the allocation in advance. |
| Blinding of participants and personnel (performance bias) All outcomes | High risk | Interventions were not equally intensive |
| Blinding of outcome assessment (detection bias) All outcomes | High risk | Self-reported |
| Incomplete outcome data (attrition bias) All outcomes | Low risk | Among randomized participants, 84.7%, 91.8%, 93.0%, and 92.2% completed the 1-, 3-, 6-, and 12-month FU. |
| Selective reporting (reporting bias) | Unclear risk | Study not pre-registered. In the main publication, the study does not break down abstinence data by flavour arm. However, the study authors provided these data to us upon request. |

Yingst 2020

|  |  |  |
| --- | --- | --- |
| ***Study characteristics*** | | |
| Methods | Design: cross-over study  Recruitment: Participants were recruited from people living with HIV/AIDS (PLWHA) (who smoked) seeking care at the Penn State Health HIV Comprehensive Care Program.  Setting: USA  Study start date: not reported | |
| Participants | Total N: 17; 41.2% female; mean age 49.1 (SD 8.8); mean cpd 16.9 (SD 7.9); mean CO 22.4 (13.1)  E-cigarettes use at baseline: not reported  Motivated to quit: no  Inclusion criteria: adult (age ≥ 18 years); smokers (≥ 10 cigarettes daily); not planning to quit smoking; documented history of a positive HIV status  Exclusion criteria: not reported | |
| Interventions | **EC: Cig-a-like; Refillable**  **Cig-a-like device** (Blu), nicotine concentration 24 mg/mL. Propylene glycol/ vegetable glycerin ratio 70/30. Nicotine delivery 4.56 ng/mL after 20 puffs in 10 minutes  **Button-operated device** (eGO), nicotine concentration 36 mg/mL. Propylene glycol/ vegetable glycerin ratio 70/30. Nicotine delivery 6.9 ng/ml after 10 puffs in 5 minutes (refillable) | |
| Outcomes | Visits: baseline, day 7, day 14, day 21  CO measured (day 0, 7, 14, 21); adverse events (nausea, dizziness)  Also: number of tobacco cigarettes smoked per day (self-report); EC puffs per day (self-report) | |
| Study funding | This study was supported by the National Institute on Drug Abuse of the National Institutes of Health under Award Number P50DA036107 and the Center for Tobacco Products of the U.S. Food and Drug Administration. JY is also funded by the Penn State Cancer Institute (PSCI) and TE is also supported by U54DA036105. The content is solely the responsibility of the authors and does not necessarily represent the official views of the National Institutes of Health or the Food and Drug Administration. | |
| Author declarations | JF has done paid consulting for pharmaceutical companies involved in producing smoking cessation medications, including GSK, Pfizer, Novartis, J&J, and Cypress Bioscience. TE is a paid consultant in litigation against the tobacco industry and the electronic cigarette industry and is named on a patent application for a device that measures the puffing behaviour of electronic cigarette users. | |
| ***Risk of bias*** | | |
| **Bias** | **Authors' judgement** | **Support for judgement** |
| Random sequence generation (selection bias) | Unclear risk | Used 2 ENDS in a random order – not enough information |
| Allocation concealment (selection bias) | Unclear risk | Not reported |
| Blinding of participants and personnel (performance bias) All outcomes | Low risk | Unable to blind, but interventions judged equally intensive |
| Blinding of outcome assessment (detection bias) All outcomes | Low risk | Objective outcome - CO monitoring (CO < 10 ppm) |
| Incomplete outcome data (attrition bias) All outcomes | Low risk | No loss to follow-up |
| Selective reporting (reporting bias) | Unclear risk | Unclear what outcomes were prespecified |

## Footnotes

ACS: acute coronary syndromes  
AE: adverse event  
AIDS: acquired immunodeficiency syndrome  
AIX: augmentation index  
AJCC: American Joint Committee on Cancer   
ALT: alanine aminotransferase  
AOD: alcohol or other drugs  
AST: aspartate aminotransferase  
BDI: Beck Depression Inventory   
BL: baseline   
BMI: body mass index  
BoE: biomarkers of exposure  
BP: blood pressure  
BUN: blood urea nitrogen  
CAR: continuous smoking abstinence rate  
CAT: COPD assessment tool

CBT: Cognitive behaviour Therapy  
CC: combustible cigarette  
CDSS: Calgary Depression Scale for Schizophrenia  
CEMA: 2-cyanoethylmercapturic acid  
CM: contingency management  
CO: carbon monoxide  
COHB: carboxyhaemoglobin  
conv-cig: conventional cigarette  
COPD: chronic obstructive pulmonary disease  
COT: cotinine  
CPD/cpd: cigarettes per day  
CRF: cardiovascular risk factors  
CRO: contract research organization  
CS: complete substitution  
CV: cardiovascular  
CVD: cardiovascular disease  
DBP: diastolic blood pressure  
DCRF: dental clinical research facility  
DHMC: Dartmouth Hitchcock Medical Center  
DSM-IV/V: Diagnostic and Statistical Manual of Mental Disorders IV/V  
ECG: electrocardiogram  
EC(IG): electronic cigarette  
eCO: expired carbon monoxide  
EEC: enhanced e-cigarette coaching   
eGO: brand of electronic cigarette  
eGFR: estimated glomerular filtration rate  
EMA: ecological momentary assessment  
ENDS: electronic nicotine delivery system  
EOP: end of pregnancy  
EoS: end of study  
EQ-5D: EuroQol-5  
EVP: electronic vaping product  
FEF: forced expiratory flow  
FEV1: forced expiratory volume  
FMD: flow-mediated dilation  
FTCD: Fagerström Test for Cigarette Dependence  
FTND: Fagerström Test for Nicotine Dependence  
FU: follow-up  
FVC: forced vital capacity  
GGT: gamma-glutamyl transferase  
GP: general practitioner doctor  
H2O: water  
HADS: Hospital Anxiety and Depression Scale  
HCT: haematocrit  
HCV: hepatitis C  
HDL(C): high-density lipoprotein  
HEETS: tobacco sticks specifically processed and manufactured for IQOS  
HIV: human immunodeficiency virus  
HPHC: harmful and potentially harmful constituents  
HR: heart rate  
HRQoL: health-related quality of life  
HS: high school  
hs-CRP: high-sensitivity C-reactive protein  
HTP: heated tobacco products  
LCQ: Leicester Cough Questionnaire  
IPI: inter-puff intervals  
IQOS: a heat-not-burn product that uses real tobacco  
IQR: interquartile range  
ITT: intention-to-treat  
LDL: low density lipoprotein  
LTA: light transmission aggregometry  
MCC: mucociliary clearance  
MCH: mean cell haemoglobin  
MCHC: mean cell haemoglobin concentration  
MDA: malondialdehyde  
MMSE: Mini Mental Status Examination  
MMT: methadone maintenance treatment  
MNWS(-R): modified Minnesota Nicotine Withdrawal  
MPS: Mood and Physical Symptoms scale  
MTSS: Motivation to Stop Scale  
MWS-R: Revised Minnesota Nicotine Withdrawal scale  
n/a: not applicable  
NCS: not clinically significant  
NCT: National Clinical Trials  
NEC: nicotine electronic cigarette  
NEQ: nicotine equivalents  
Nic: nicotine  
NNAL: carcinogen found in tobacco smoke (4‐(methylnitrosamino)‐1‐(3‐pyridyl)‐1‐butanol)  
NNC: non-nicotine cigarette  
NNK: nicotine-derived nitrosamine ketone  
NR: not reported  
NRT: nicotine replacement therapy  
NS: not stated  
NSP: nicotine salt e-liquid pods  
NVP: nicotine vaping product  
OKHL: Oklahoma Tobacco Helpline  
OST: opioid substitution therapy  
OTH: Oklahoma Tobacco Helpline  
OTP: other tobacco products  
PAI-1: plasminogen activator inhibitor type 1  
PANSS: Positive and Negative Syndrome Score  
PAOD: peripheral arterial occlusive disease  
PBR: perfused boundary region  
PCV: haematocrit  
pd: per day  
PEC: placebo electronic cigarette  
PEF: peak expiratory flow  
PG: propylene glycol  
PI: principal investigator  
PIS: patient information sheet   
PLTs: platelets  
PLWHA: people living with HIV/AIDS  
PP(A): point prevalence (abstinence)  
ppm: parts per million  
PTSD: post-traumatic stress disorder  
PV: personal vaporizers  
PWV: pulse wave velocity  
QL: quitline  
QSU(Brief): Questionnaire of Smoking Urges-Brief  
RA: research assistant  
RBC: red blood cells  
SAE: serious adverse event  
SBP: systolic blood pressure   
SCQ-A: Smoking Consequences questionnaire-Adult  
SD: standard deviation  
SDM: standardised mean  
SMI: serious mental illness  
SMS: short message service  
SNOT-22: Sino-Nasal Outcomes Test  
SRNT: Society for Research on Nicotine and Tobacco  
SSS: stop-smoking services  
TAU: treatment-as-usual  
TC: total cigarettes, tobacco cigarettes  
TCD: transcranial doppler  
THC: tetrahydrocannabinol  
TLFB: timeline follow-back  
TNE: total nicotine equivalents  
TQD: target quit date  
UC: usual care  
USB: universal serial bus  
VA: Veterans Association  
VAMC: Veterans Affairs Medical Center  
VAS: visual analogue scale  
VG: vegetable glycerine  
VLNC: very low nicotine content  
VNP: vaporized nicotine products  
VO2: oxygen consumption  
WBC: white blood cells

## References to studies

### Adriaens 2014 {published data only}

- Adriaens K, Van Gucht D, Declerck P, Baeyens F. Effectiveness of the electronic cigarette: an eight-week Flemish study with six-month follow-up on smoking reduction, craving and experienced benefits and complaints. International Journal of Environmental Research and Public Health 2014;11(11):11220-48.

### Auer 2024 {published data only}

- \*Auer R, Schoeni A, Humair J-P, Jacot-Sadowski I, Berlin I, Stuber MJ, et al. Electronic nicotine-delivery systems for smoking cessation. New England Journal of Medicine 2024;390(7):601-10. [DOI: 10.1056/NEJMoa2308815]
- Auer R, Schoeni A, Humair JP, Jacot-Sadowski I, Berlin I, Stuber M, et al. Efficacy, safety and toxicology of electronic nicotine delivery systema (ESTXENDS) as an aid for smoking cessation, a randomised controlled trial. In: SRNT 29th Annual Meeting; 2023 Mar 1-4; San Antonio (TX), USA. 2023:POS3-5.
- Baggio S, Bruggmann P, Schoeni A, Abolhassani N, Tal K, Pohle S, et al. Efficacy of e-cigarettes for smoking cessation in populations with psychiatric and/or substance use problems: a secondary analysis of a randomized controlled trial. Tobacco Prevention & Cessation 2025;11:1-10. [DOI: 10.18332/tpc/199473]
- Baggio S, Schoeni A, Abolhassani N, Tal K, Pohle S, Vetsch J, et al. Efficacy of electronic nicotine delivery systems (ENDS) for smoking cessation in populations with psychiatric and substance use problems: secondary analysis of a randomized controlled trial. In: SRNT 30th Annual Meeting, March 2024, Edinburgh UK. 2024:PPS24-1.
- Güttinger E, Mosimann A, Tal K, Schoeni A, Jacot-Sadowski I, Humair JP, et al. Prevalence of vaping-associated symptoms over six months among participants of a randomized controlled trial on smoking cessation with electronic nicotine delivery systems (ENDS). In: SRNT 29th Annual Meeting; 2023 Mar 1-4; San Antonio (TX), USA. 2023:PPS14-6.
- Jakob J, Schoeni A, Tal K, Jacot-Sadowski I, Clair C, Humair JP, et al. Effects of electronic nicotine delivery systems (ENDS) for smoking cessation on changes in weight- secondary analysis of the ESTXENDS trial. In: SRNT 29th Annual Meeting; 2023 Mar 1-4; San Antonio (TX), USA. 2023:POS3-8.
- Mosimann AF, Güttinger EM, Tal K, Baggio S, Schoeni A, Jacot-Sadowski I, et al. Change in e-liquids flavor use and nicotine concentration over 6 months in participants of a smoking cessation trial with electronic nicotine delivery systems (ENDS): the ESTxENDS Trial. In: SRNT 29th Annual Meeting; 2023 Mar 1-4; San Antonio (TX), USA. 2023:46 (PPS10-5).
- Mosimann AF, Guttinger EM, Tal K, Schoeni A, Baggio S, Sambiagio N, et al. E-liquid flavors and nicotine concentration choices over 6 months after a smoking cessation attempt with ENDS: secondary analyses of a randomized controlled trial. Tobacco Prevention & Cessation 2025;11:101693412. [DOI: 10.18332/tpc/196136]
- NCT03589989. The ESTxENDS trial - electronic nicotine delivery systems (ENDS/vaporizer/e-cigarette) as an aid for smoking cessation (ESTxENDS). clinicaltrials.gov/ct2/show/record/NCT03589989 (first received 18 July 2018).
- NCT03603340. The ESTxENDS trial - effects of using Electronic Nicotine Delivery Systems (ENDS/Vaporizer/E-cig) on depression (ESTxENDS). clinicaltrials.gov/show/NCT03603340 (first received 27 July 2018).
- NCT03603353. The ESTxENDS trial - effects of using Electronic Nicotine Delivery Systems (ENDS/Vaporizer/E-cig) on sleep quality (ESTxENDS). clinicaltrials.gov/show/NCT03603353 (first received 27 July 2018).
- NCT03612336. The ESTxENDS trial - metabolic effects of using Electronic Nicotine Delivery Systems (ENDS/Vaporizer/E-cig) (ESTxENDS). clinicaltrials.gov/show/NCT03612336 (first received 2 August 2018).
- NCT03612375. ESTxENDS trial - oxidative stress induced by Electronic Nicotine Delivery Systems (ENDS/Vaporizer/E-cig) measured in urine (ESTxENDS). clinicaltrials.gov/show/NCT03612375 (first received 2 August 2018).
- NCT03612453. ESTxENDS Trial - oxidative stress induced by Electronic Nicotine Delivery Systems (ENDS/Vaporizer/E-cig) measured in EBC (ESTxENDS). clinicaltrials.gov/show/NCT03612453 (first received 2 August 2018).
- NCT03612544. The ESTxENDS trial - toxins from using Electronic Nicotine Delivery Systems (ENDS/Vaporizer/E-cig) (ESTxENDS). clinicaltrials.gov/show/NCT03612544 (first received 2 August 2018).
- NCT03632421. The ESTxENDS trial - effects of using Electronic Nicotine Delivery Systems (ENDS/Vaporizer/E-cig) on respiratory symptoms (ESTxENDS). clinicaltrials.gov/show/NCT03632421 (first received 15 August 2018).
- NCT03938298. The ESTxENDS trial: pulmonary function substudy (PulmENDS). clinicaltrials.gov/ct2/show/nct03938298 (first received 6 May 2019).
- NCT04236791. The ESTxENDS trial - electronic nicotine delivery systems as an aid for smoking cessation - extension of follow-up. clinicaltrials.gov/show/NCT04236791 (first received 22 January 2020).
- NCT04244773. ESTxENDS trial: MN substudy - micronuclei in buccal epithelium, a surrogate measure of future cancer risk, induced by Electronic Nicotine Delivery Systems (ENDS/Vaporizer/E-cig). clinicaltrials.gov/show/NCT04244773 (first received 28 January 2020).
- NCT04617444. The ESTxENDS trial - effects of using Electronic Nicotine Delivery Systems (ENDS/Vaporizer/E-cig) on olfactory function. clinicaltrials.gov/show/NCT04617444 (first received 05 November 2020).
- Nyilas S, Bauman G, Korten I, Pusterla O, Singer F, Ith M. MRI shows lung perfusion changes after vaping and smoking. Radiology 2022;304(1):195-204. [DOI: 10.1148/radiol.211327]
- Rihs A, Schoeni A, Scharf T, Jakob J, Rodondi N, Jacot-Sadowski I, et al. Effect of e-cigarettes for smoking cessation on depressive and anxiety symptoms: secondary analyses of a randomised controlled trial. In: Society for Research on Nicotine and Tobacco (SRNT) 29th Annual Meeting 1-4 March 2023. PPS10-6. 2023:46.
- Rihs A, Schoeni A, Scharf T, Jakob J, Tal K, Jacot-Sadowski I, et al. Effect of e-cigarettes for smoking cessation on depressive and anxiety symptoms: secondary analysis of a randomized controlled trial. General Hospital Psychiatry 2025;93:67-72. [DOI: 10.1016/j.genhosppsych.2025.01.011]
- Scharf T, Rihs 1, Schoeni A, Maire M, Tal K, Jakob J, et al. Effect of electronic nicotine delivery systems for smoking cessation on sleep quality: secondary analysis of a randomised controlled trial. In: Society for Research on Nicotine and Tobacco (SRNT) 20-23 March 2024 Edinburgh UK. POS4-66. 2024.
- Schoeni A, Baggio S, Jakob J, Humair J-P, Jacot-Sadowski I, Berlin I, et al. Long-term efficacy outcomes of electronic nicotine delivery systems (ENDS) for smoking cessation: 12- and 24-months follow-up of the efficacy, safety and toxicology of ENDS (ESTxENDS) randomized controlled trial. In: Society for Research on Nicotine and Tobacco (SRNT) 20-23 March 2024 Edinburgh UK. PPS25-1. 2024.
- Schoeni A, Baggio S, Jakob J, Humair J-P, Jacot-Sadowski I, Berlin I, et al. Long term efficacy outcomes of electronic nicotine delivery systems (ENDS) for smoking cessation: 12- and 24- months follow-up of the efficacy, safety and toxicology of ENDS (ESTxENDS) randomized controlled trial. Swiss Medical Weekly 2024;154:15S-16S. [DOI: 10.57187/s.3896]

### Avila 2024 {published data only}

- \*Avila JC, Maglalang DD, Nollen N, Lee SC, Suh R, Malone M, et al. Using pod based e-cigarettes and nicotine pouches to reduce harm for adults with low socioeconomic status who smoke: a pilot randomized controlled trial. Nicotine & Tobacco Research 2024;26(9):1150-8. [DOI: 10.1093/ntr/ntae047]
- NCT05327439. Using alternative nicotine delivery systems (ANDS) to reduce harm for low SES cigarette smokers (Tri-PEC study). clinicaltrials.gov/ct2/show/NCT05327439 (first received 14 April 2022).

### Baldassarri 2018 {published data only}

- \*Baldassarri SR, Bernstein SL, Chupp GL, Slade MD, Fucito LM, Toll BA. Electronic cigarettes for adults with tobacco dependence enrolled in a tobacco treatment program: a pilot study. Addictive Behaviors 2018;80:1-5. [DOI: 10.1016/j.addbeh.2017.11.033]
- Baldassarri SR, Bernstein SL, Chupp GL, Toll BA. Electronic cigarettes for adults with tobacco dependence undergoing a tobacco treatment program: a pilot prospective study. American Journal of Respiratory and Critical Care Medicine, Meeting Abstracts 2016;193:A6511.
- NCT02498145. Short term effects of electronic cigarettes in tobacco dependent adults. clinicaltrials.gov/show/NCT02498145 (first received 13 July 2015).

### Begh 2021 {published data only (unpublished sought but not used)}

- \*Begh R, Bateman P, Williams N, Grabey J, Stevens R. Examining the effectiveness of general practitioner and nurse promotion of electronic cigarettes versus standard care for smoking reduction and abstinence in hardcore smokers with smoking-related chronic disease: a randomised controlled trial. Statistical Analysis Report, Version 2.0 20211-56.
- Albury C, Barnes R, Ferrey A, Coleman T, Gilbert H, Naughton F, et al. The old and familiar meets the new and unknown: patient and clinician perceptions on e-cigarettes for smoking reduction in UK general practice, a qualitative interview study. Addiction 2022;117(5):1427-37. [DOI: 10.1111/add.15760]
- Begh R, Coleman T, Yardley L, Barnes R, Naughton F, Gilbert H, et al. Examining the effectiveness of general practitioner and nurse promotion of electronic cigarettes versus standard care for smoking reduction and abstinence in hardcore smokers with smoking-related chronic disease: protocol for a randomised controlled trial. Trials 2019;20(1):659.
- Begh R. Training general practitioners and nurses to deliver brief advice about e-cigarettes for smoking reduction: lessons learned from a randomised controlled trial. In: SRNT E-Conference, SY3-04. 2021.
- ISRCTN59404712. GP/nurse promotion of e-cigarettes in supporting reduced smoking and cessation in smokers. www.isrctn.com/ISRCTN59404712 (first received 28 November 2017).

### Bonafont Reyes 2022 {published data only}

- \*Bonafont Reyes BV, Stevens E, Nicholson A, Lei L, Vojjala M, Sherman S. Understanding racial and ethnic differences in switching from combustible cigarettes to e-cigarettes in COPD patients. Journal of the American Geriatrics Society, Annual Scientific Meeting 2022;170(S1):S1-S345.

### Bonevski 2021 {unpublished data only}

- \*Bonevski B, Manning V, Wynne O, Gartner C, Borland R, Baker A, et al. QuitNic: a pilot randomised controlled trial comparing nicotine vaping products with nicotine replacement therapy for smoking cessation following residential detoxification. Nicotine & Tobacco Research 2021;23(3):462-70.
- ACTRN12617000849392. The QuitNic Study: a pilot study of electronic nicotine devices for smoking cessation with drug and alcohol clients. www.anzctr.org.au/Trial/Registration/TrialReview.aspx?id=372198 (first received 8 June 2017).
- Guillaumier A, Manning V, Wynne O, Gartner C, Borland R, Baker AL, et al. Electronic nicotine devices to aid smoking cessation by alcohol- and drug-dependent clients: protocol for a pilot randomised controlled trial. Trials 2018;19:415.

### Bullen 2013 {published data only}

- \*Bullen C, Howe C, Laugesen M, McRobbie H, Parag V, Williman J, et al. Electronic cigarettes for smoking cessation: a randomised controlled trial. Lancet 2013;382(9905):1629-37.
- Bullen C, Howe C, Laugesen M, McRobbie H, Parag V, Williman J, et al. Electronic cigarettes and smoking cessation: a quandary? - Authors' reply. Lancet 2014;383(9915):408-9.
- Bullen C, Howe C, Laugesen M, McRobbie H, Parag V, Williman J. Do electronic cigarettes help smokers quit? Results from a randomised controlled trial [Abstract]. In: European Respiratory Society Annual Congress, 2013 September 7-11, Barcelona, Spain. Vol. 42. 2013:215s-[P1047].
- Bullen C, Williman J, Howe C, Laugesen M, McRobbie H, Parag V, et al. Study protocol for a randomised controlled trial of electronic cigarettes versus nicotine patch for smoking cessation. BMC Public Health 2013;13:210.
- O'Brien B, Knight-West O, Walker N, Parag V, Bullen C. E-cigarettes versus NRT for smoking reduction or cessation in people with mental illness: secondary analysis of data from the ASCEND trial. Tobacco Induced Diseases 2015;13(1):5.

### Caponnetto 2013a\* {published data only}

- \*Caponnetto P, Campagna D, Cibella F, Morjaria JB, Caruso M, Russo C, et al. EffiCiency and safety of an eLectronic cigAreTte (ECLAT) as tobacco cigarettes substitute: a prospective 12-month randomized control design study. PloS One 2013;8(6):e66317.
- Campagna D, Cibella F, Caponnetto P, Amaradio MD, Caruso M, Morjaria JB, et al. Changes in breathomics from a 1-year randomized smoking cessation trial of electronic cigarettes. European Journal of Clinical Investigation 2016;46(8):698-706.
- Cibella F, Campagna D, Caponnetto P, Amaradio MD, Caruso M, Russo C, et al. Lung function and respiratory symptoms in a randomized smoking cessation trial of electronic cigarettes. Clinical Science 2016;130(21):1929-37.
- Farsalinos K, Cibella F, Caponnetto P, Campagna D, Morjaria JB, Battaglia E, et al. Effect of continuous smoking reduction and abstinence on blood pressure and heart rate in smokers switching to electronic cigarettes. Internal and Emergency Medicine 2016;11(1):85-94.
- NCT01164072. Efficacy and safety of an electronic nicotine delivery device (e-cigarette). clinicaltrials.gov/ct2/show/NCT01164072 (first received 16 July 2010).
- NCT01188239. A structured protocol to evaluate efficacy and safety of a popular electronic nicotine delivery device (e-cigarette) efficacy and safety of a popular electronic nicotine delivery device (e-cigarette). https://clinicaltrials.gov/ct2/show/NCT01188239 (first received 25 AUgust 2010).
- NCT01194583. Efficacy and safety of an electronic nicotine delivery device (e-cigarette) without nicotine cartridges. clinicaltrials.gov/ct2/show/NCT01194583 (first received 3 September 2010).
- Russo C, Cibella F, Caponnetto P, Campagna D, Maglia M, Frazzetto E, et al. Evaluation of post cessation weight gain in a 1-year randomized smoking cessation trial of electronic cigarettes. Scientific Reports 2016;6:18763.

### Caponnetto 2013b\* {published data only}

- \*Caponnetto P, Auditore R, Russo C, Cappello GC, Polosa R. Impact of an electronic cigarette on smoking reduction and cessation in schizophrenic smokers: a prospective 12-month pilot study. International Journal of Environmental Research and Public Health 2013;10(2):446-61.
- Minutolo G, Caponnetto P, Auditore R, Russo C, Polosa R. Management of smoking reduction and cessation in inpatients with schizophrenia: impact of electronic cigarettes. European Neuropsychopharmacology 2013;23:S581-2.

### Caponnetto 2021\* {published data only}

- \*Caponnetto P, DiPiazza J, Kim J, Maglia M, Polosa R. A single-arm, open-label pilot, and feasibility study of a high nicotine strength e-cigarette intervention for smoking cessation or reduction for people with schizophrenia spectrum disorders who smoke cigarettes. Nicotine & Tobacco Research 2021;23(7):1113-22. [DOI: 10.1093/ntr/ntab005]

### Caponnetto 2023\* {published data only}

- \*Caponnetto P, Campagna D, Maglia M, Benfatto F, Emma R, Caruso M, et al. Comparing the effectiveness, tolerability, and acceptability of heated tobacco products and refillable electronic cigarettes for cigarette substitution (CEASEFIRE): randomized controlled trial. Public Health Surveillance 2023;9:e42628. [DOI: 10.2196/42628]
- Caponnetto P, Caruso M, Maglia M, Emma R, Saitta D, Busa B, et al. Non-inferiority trial comparing cigarette consumption, adoption rates, acceptability, tolerability, and tobacco harm reduction potential in smokers switching to Heated Tobacco Products or electronic cigarettes: study protocol for a randomized controlled trial. Contemporary Clinical Trials Communications 2020;8:17.
- NCT03569748. Heated tobacco products vs electronic cigarettes. clinicaltrials.gov/ct2/show/NCT03569748 (first received 25 June 2021).

### Carpenter 2017 {published data only}

- \*Carpenter MJ, Heckman BW, Wahlquist AE, Wagener TL, Goniewicz ML, Gray KM, et al. A naturalistic, randomized pilot trial of e-cigarettes: uptake, exposure, and behavioral effects. Cancer Epidemiology, Biomarkers & Prevention 2017;26(12):1795-803.
- NCT02357173. A trial of e-cigarettes: natural uptake, patterns and impact of use. clinicaltrials.gov/show/NCT02357173 (first received 3 October 2014).
- Smith TT, Wahlquist AE, Heckman BW, Cummings KM, Carpenter MJ. Impact of e-cigarette sampling on cigarette dependence and reinforcement value. Nicotine & Tobacco Research 2020;22(2):297-301.

### Carpenter 2023 {published and unpublished data}

- \*Carpenter MJ, Wahlquist A, Cummings KM, Smith T. Complementary approaches to examining relationships between e-cigarettes and smoking: novel data from the US. In: SRNT 29th Annual Meeting; 2023 Mar 1-4; San Antonio (TX), USA. SYM5-1. 2023:5-6.
- Carpenter MJ, Wahlquist AE, Dahne J, Gray KM, Cummings KM, Warren G, et al. Effect of unguided e-cigarette provision on uptake, use, and smoking cessation among adults who smoke in the USA: a naturalistic, randomised, controlled clinical trial. EClinicalMedicine 2023;63:102142. [DOI: 10.1016/j.eclinm.2023.102142]
- Hunter JN, Fahey MC, Wahlquist A, Smith T, Carpenter M. E-cigarette expectations and use among non-treatment seeking veteran smokers in a small nationawide veteran population. In: SRNT 30th Annual Meeting March 2024, Edinburgh UK. POS3-122. 2024.
- NCT03453385. Clinical outcomes of a nationwide, naturalistic E-cig trial (CONNECT). clinicaltrials.gov/ct2/show/NCT03453385 (first received 5 March 2018).
- O'Neal R, Fahey MC, Wahlquist A, Smith T, Carpenter M. Demographic and smoking history correlates of anticipated reasons for ENDS use. In: SRNT 29th Annual Meeting; 2023 Mar 1-4; San Antonio (TX), USA. POS2-18. 2023:121.
- O'Neal RA, Carpenter MJ, Wahlquist AE, Leavens EL, Smith TT, Fahey MC. The prospective relationship between a-priori intentions for and patterns of e-cigarette use among adults who smoke cigarettes. Addictive Behaviors 2024;156:108067. [DOI: 10.1016/j.addbeh.2024.108067]
- Smith TT, Wahlquist AE, Wagener TL, Cummings KM, Carpenter MJ. The impact of non-tobacco e-cigarette flavoring on e-cigarette uptake, cigarette smoking reduction, and cessation: a secondary analysis of a nationwide clinical trial. Addictive Behaviors 2025;163:108240. [DOI: 10.1016/j.addbeh.2024.108240]

### Cobb 2021 {published and unpublished data}

- \*Cobb CO, Foulds J, Yen M-S, Veldheer S, Lopez AA, Yingst JM, et al. Effect of an electronic nicotine delivery system with 0, 8, or 36 mg/mL liquid nicotine versus a cigarette substitute on tobacco-related toxicant exposure: a four-arm, parallel-group, randomised, controlled trial. Lancet Respiratory Medicine 2021;9(8):840-50. [DOI: 10.1016/ S2213-2600(21)00022-9]
- Dahal S, Yingst J, Wang X, Cobb C, Hrabovsky S, Bascom R, et al. Changes in cardiopulmonary and other clinical health outcomes among adult smokers using electronic cigarettes: an exploratory analysis from a randomized controlled smoking reduction trial. In: SRNT Rapids 30th Annual Meeting, March 2024, New Orleans USA. POS5-5. 2024.
- Dahal S, Yingst J, Wang X, Cobb CO, Carrillo M, Hrabovsky S, et al. Changes in cardiovascular disease risk, lung function, and other clinical health outcomes when people who smoke use e-cigarettes to reduce cigarette smoking: an exploratory analysis from a randomized placebo-controlled trial. MedRxiv: the Preprint Server for Health Sciences 2024. [DOI: 10.1101/2024.12.14.24319048]
- Foulds J, Cobb C, Yen M-S, Veldheer S, Brosnan P, Yingst J, et al. Effect of electronic nicotine delivery systems on cigarette abstinence in smokers with no plans to quit: exploratory analysis of a randomized placebo-controlled trial. Nicotine Tobacco Research 2022;24(7):955-61. [DOI: 10.1093/ntr/ntab247]
- Lopez AA, Cobb CO, Yingst JM, Veldheer S, Hrabovsky S, Yen MS, et al. A transdisciplinary model to inform randomized clinical trial methods for electronic cigarette evaluation. BMC Public Health 2016;16(1):217. [PMCID:: PMC4778292] [PMID: 26941050]
- NCT02342795. Randomized controlled trial methods for novel tobacco products evaluation. clinicaltrials.gov/show/NCT02342795 (first received 16 December 2014).
- Veldheer S, Yingst J, Midya V, Hummer B, Lester C, Krebs N. Pulmonary and other health effects of electronic cigarette use among adult smokers participating in a randomized controlled smoking reduction trial. Addictive Behaviors 2019;91:95-101.
- Yingst J, Foulds J, Veldheer S, Cobb CO, Yen M, Hrabovsky S, et al. Measurement of electronic cigarette frequency of use among smokers participating in a randomized controlled trial. Nicotine & Tobacco Research 2020;22(5):699-704.
- Yingst J, Midya V, White A, Foulds J, Cobb CO, Veldheer S, et al. Effects of liquid nicotine concentration and flavour on the acceptability of electronic nicotine delivery systems (ENDS) among people who smoke participating in a randomised controlled trial to reduce cigarette consumption. Tobacco Control 2025;34(4):496-505. [DOI: 10.1136/tc-2023-058282]
- Yingst J, Wang X, Anderson A, Breland A, Soule E, Barnes A, et al. Evaluation of nicotine dependence among smokers using electronic cigarettes to reduce cigarette smoke. In: Society for Research on Nicotine and Tobacco, Annual Meeting, March 2022, Baltimore USA. 2022.
- Yingst J, Wang Xi, Lopez AA, Breland A, Soule E, Barnes A, et al. Randomized control trial methods workgroup of the center for the study of tobacco products, changes in nicotine dependence among smokers using electronic cigarettes to reduce cigarette smoking in a randomized controlled trial. Nicotine & Tobacco Research 2023;25(3):372-8. [DOI: 10.1093/ntr/ntac153]

### Coffey 2020 {published data only}

- Coffey M, Cooper-Ryan AM, Houston L, Thompson K, Cook PA. Using e-cigarettes for smoking cessation: evaluation of a pilot project in the North West of England. Perspectives in Public Health 2020;140(6):351-61. [DOI: 10.1177/1757913920912436]

### Czoli 2019 {published and unpublished data}

- Czoli CD, Fong GT, Goniewicz ML, Hammond D. Biomarkers of exposure among "dual users" of tobacco cigarettes and electronic cigarettes in Canada. Nicotine & Tobacco Research 2019;21(9):1259-66.

### Dawkins 2020 {published and unpublished data}

- \*Dawkins L, Bauld L, Ford A, Robson D, Hajek P, Parrott S, et al. A cluster feasibility trial to explore the uptake and use of e-cigarettes versus usual care offered to smokers attending homeless centres in Great Britain. PloS One 2020;15(10):e0240968.
- ISRCTN14140672. Exploring the use and uptake of e-cigarettes for homeless smokers. www.isrctn.com/ISRCTN14140672 (first received 7 November 2018).

### Edmiston 2022\* {published data only}

- \*Edmiston JS, Webb KM, Wang J, Oliveri D, Liang Q, Sarkar M. Biomarkers of exposure and biomarkers of potential harm in adult smokers who switch to e-vapor products relative to cigarette smoking in a 24-week, randomized, clinical trial. Nicotine & Tobacco Research 2022;24(7):1047–54. [DOI: 10.1093/ntr/ntac029]

### Edwards 2023 {published and unpublished data}

- \*Edwards S, Puljevic C, Dean JA, Gilks C, Boyd MA, Baker P, et al. Tobacco harm reduction with vaporised nicotine (THRiVe): a feasibility trial of nicotine vaping products for smoking cessation among people living with HIV. AIDS and Behavior 2023;27(2):618-27. [DOI: 10.1007/s10461-022-03797-0]
- ACTRN12616001641482. Tobacco harm reduction with vaporised nicotine (THRiVe): feasibility study. www.anzctr.org.au/Trial/Registration/TrialReview.aspx?ACTRN=12616001641482 (first received 28 November 2016).
- Bell S, Dean J, Gilks C, Boyd MA, Fitzgerald L, Mutch A, et al. Tobacco harm reduction with vaporised nicotine (THRiVe): the study protocol of an uncontrolled feasibility study of novel nicotine replacement products among people living with HIV who smoke. International Journal of Environmental Research and Public Health 2017;14(7):799.

### Eisenberg 2020 {published data only}

- \*Eisenberg MJ, Hébert-Losier M, Windle SB, Greenspoon T, Brandys T, Fülöp T. Effect of e-cigarettes plus counseling vs counseling alone on smoking cessation: a randomized clinical trial. JAMA 2020;324(18):1844-54.
- Hebert-Losier A, Filion KB, Windle SB, Eisenberg MJ. A randomized controlled trial evaluating the efficacy of e-cigarette use for smoking cessation in the general population: E3 trial design. Canadian Journal of Cardiology Open 2020;2(3):168-75.
- Lyzwinski L, Dong M, Wolfinger RD, Filion KB, Eisenberg MJ. E-cigarettes, smoking cessation, and weight change: retrospective secondary analysis of the evaluating the efficacy of e-cigarette use for smoking cessation trial. JMIR Public Health and Surveillance 2024;10:e58260. [DOI: 10.2196/58260]
- NCT02417467. Evaluating the efficacy of e-cigarette use for smoking cessation (E3) trial. clinicaltrials.gov/show/NCT02417467 (first received 1 April 2015).
- Prell C, Hebert-Losier A, Filion KB, Reynier P, Eisenberg MJ. Evaluating the impact of varying expired carbon monoxide thresholds on smoking relapse identification: insights from the E3 trial on e-cigarette efficacy for smoking cessation. BMJ Open 2023;13(13):10. [DOI: 10.1136/bmjopen-2022-071099]

### Eisenhofer 2015 {published data only}

- Eisenhofer J, Makanjuola T, Martinez V, Thompson-Lake DG, Rodgman C, DeBrule DS, et al. Efficacy of electronic cigarettes for smoking cessation in veterans. Drug and Alcohol Dependence 2015;156:e63-4.

### Elling 2023 {published data only}

- \*Elling JM, Crutzen R, Talhout R, De Vries H. Effects of providing tailored information about e-cigarettes in a digital smoking cessation intervention: randomized controlled trial. Health Education Research 2023;38(2):150-62. [DOI: 10.1093/her/cyad004]
- Elling JM, Crutzen R, Talhout R, De Vries H. Effects of providing tailored information about e-cigarettes in a web-based smoking cessation intervention: protocol for a randomised control trial. Journal of Medical Internet Research, Research Protocols 2021;10(5):e27088. [DOI: 10.2196/27088]
- NL8330. Communication about e-cigarettes in a digital smoking cessation intervention [Effect of communication about e-cigarettes in a digital smoking cessation intervention on smoking reduction, smoking cessation, and informed decision making]. trialsearch.who.int/?TrialID=NL8330 (first received 24 January 2020).

### Ely 2013 {published data only}

- Ely J. Evaluation of the use of electric cigarettes in a rural smoking cessation program. digscholarship.unco.edu/cgi/viewcontent.cgi?article=1001&context=capstones 2013 (accessed 1 November 2014).

### Felicione 2019 {published data only}

- Felicione NJ, Enlow P, Elswick D, Long D, Sullivan CR, Blank MD. A pilot investigation of the effect of electronic cigarettes on smoking behavior among opioid-dependent smokers. Addictive Behaviors 2019;91:45-50.

### George 2019 {published data only}

- \*George J, Hussain M, Vadiveloo T, Ireland S, Hopkinson P, Struthers AD, et al. Cardiovascular effects of switching from tobacco cigarettes to electronic cigarettes. Journal of the American College of Cardiology 2019;74(25):3112-20.
- NCT02878421. Vascular EffectS of regUlar cigarettes Versus electronIc cigarette USe (VESUVIUS). clinicaltrials.gov/ct2/show/NCT02878421 (first received 25 August 2016).

### Goniewicz 2017 {published data only}

- \*Goniewicz ML, Gawron M, Smith DM, Peng M, Jacob P 3rd, Benowitz NL. Exposure to nicotine and selected toxicants in cigarette smokers who switched to electronic cigarettes: a longitudinal within-subjects observational study. Nicotine & Tobacco Research 2017;19(2):160-7.

### Hajek 2015a {published data only}

- Hajek P, Corbin L, Ladmore D, Spearing E. Adding e-cigarettes to specialist stop-smoking treatment: City of London pilot project. Journal of Addiction Research & Therapy 2015;6(244):(online ahead of print). [DOI: 10.4172/2155-6105.1000244]

### Hajek 2019 {published and unpublished data}

[isrctn: ISRCTN60477608 ]

- \*Hajek P, Phillips-Waller A, Przulj D, Pesola F, Myers Smith K, Bisal N, et al. A randomized trial of e-cigarettes versus nicotine-replacement therapy. New England Journal of Medicine 2019;380(7):629-37.
- Hajek P, Phillips-Waller A, Przulj D, Pesola F, Smith KM, Bisal N, et al. E-cigarettes compared with nicotine replacement therapy within the UK Stop Smoking Services: the TEC RCT. Health Technology Assessment 2019;23(43):1-82.
- ISRCTN60477608. The efficacy of e-cigarettes compared with nicotine replacement therapy, when used within the UK stop smoking service. www.isrctn.com/ISRCTN60477608 (first received 2 April 2015).
- Li J, Hajek P, Pesola F, Wu Q, Phillips-Waller A, Przulj D, et al. Cost-effectiveness of e-cigarettes compared with nicotine replacement therapy in stop smoking services in England (TEC study): a randomized controlled trial. Addiction (Abingdon, England) 2020;115(3):507-17.

### Hajek 2022 {published and unpublished data}

- \*Hajek P, Przulj D, Pesola F, Griffiths C, Walton R, McRobbie H, et al. Electronic cigarettes versus nicotine patches for smoking cessation in pregnancy: a randomized controlled trial. Nature Medicine 2022;28:958-64. [DOI: 10.1038/s41591-022-01808-0]
- Ford A, Uny I, Lowes J, Naughton F, Cooper S, Coleman T. A qualitative study of factors influencing adherence among pregnant women taking part in a trial of e-cigarettes for smoking cessation. International Journal of Environmental Research and Public Health 2021;18(2):430.
- Hajek P, Przulj D, Pesola F, Griffiths C, Walton R, McRobbie H, et al. Author Correction: Electronic cigarettes versus nicotine patches for smoking cessation in pregnancy: a randomized controlled trial. Nature Medicine 2023;29(11):2957. [DOI: 10.1038/s41591-022-02099-1]
- ISRCTN62025374. Helping pregnant smokers quit: a multi-centre study of electronic cigarettes and nicotine patches. www.isrctn.com/ISRCTN62025374 (first received 21 March 2017). [DOI: 10.1186/ISRCTN62025374]
- Pesola F, Smith KM, Phillips-Waller A, Przulj D, Griffiths C, Walton R, et al. Safety of e-cigarettes and nicotine patches as stop-smoking aids in pregnancy: secondary analysis of the Pregnancy Trial of E-cigarettes and Patches (PREP) randomized controlled trial. Addiction (Abingdon, England) 2024;119(5):875-84. [DOI: 10.1111/add.16422]
- Przulj D, Pesola F, Smith KM, McRobbie H, Coleman T, Lewis S, et al. Helping pregnant smokers quit: a multicentre randomised controlled trial of electronic cigarettes versus nicotine replacement therapy. Health Technology Assessment 2023;27(13):1-53. [DOI: 10.3310/AGTH6901]

### Halpern 2018 {published data only}

- \*Halpern SD, Harhay MO, Saulsgiver K, Brophy C, Troxel AB, Volpp KG. A pragmatic trial of e-cigarettes, incentives, and drugs for smoking cessation. New England Journal of Medicine 2018;378(24):2302-10.
- Harhay MO, Troxel AB, Brophy C, Saulsgiver K, Volpp KG, Halpern SD. Financial incentives promote smoking cessation directly, not by increasing use of cessation aids. Annals of the American Thoracic Society 2019;16(2):280-2. [DOI: 10.1513/AnnalsATS.201808-574RL]
- NCT02328794. Randomized clinical trial to reduce harm from tobacco. clinicaltrials.gov/show/NCT02357173 (first received 3 October 2014).

### Hatsukami 2020 {published and unpublished data}

- \*Hatsukami D, Meier E, Lindgren BR, Anderson A, Reisinger S, Norton K, et al. A randomized clinical trial examining the effects of instructions for electronic cigarette use on smoking-related behaviors, and biomarkers of exposure. Nicotine & Tobacco Research 2020;22(9):1524-32. [DOI: 10.1093/ntr/ntz233]
- NCT03111537. Methods project 4: clinical trial - amended (COMET). https://clinicaltrials.gov/ct2/show/NCT03111537 (first received 21 May 2015).

### Hickling 2019 {published data only}

- \*Hickling LM, Perez-Iglesias R, McNeill A, Dawkins L, Moxham J, Ruffell T, et al. A pre-post pilot study of electronic cigarettes to reduce smoking in people with severe mental illness. Psychological Medicine 2019;49(6):1033-40.
- NCT02212041. Acceptability, patterns of use and safety of electronic cigarette in people with mental illness: a pilot study. clinicaltrials.gov/show/NCT02212041 (first received 6 August 2014).

### Higgins 2024 {published data only}

- \*Higgins ST, Sigmon SC, Tidey JW, Heil SH, Gaalema DE, Lee DC, et al. Reduced nicotine cigarettes and e-cigarettes in high-risk populations: 3 randomized clinical trials. JAMA Network Open 2024;7(9):e2431731. [DOI: 10.1001/jamanetworkopen.2024.31731]
- DeAtley T, Harrison A, Camacho B, Cassidy R, Kuo C, Higgins ST, et al. Subjective experiences, contexts, and risk perceptions of very low content cigarettes and electronic cigarettes among people with affective disorders who smoke. In: Society for Research on Nicotine and Tobacco Annual Meeting, 2022 March; Baltimore USA. 2022.
- DeAtley T, Harrison A, Cassidy R, Kuo C, Higgins ST, Tidey JW. Subjective experiences, contexts, and risk perceptions of very low nicotine content cigarettes and electronic cigarettes among people with depression and anxiety disorders who smoke. Drug and Alcohol Dependence 2023;244:109767.
- Higgins ST, Tidey J, Sigmon S, Gaalema DE, Lee D, Heil S, et al. Availability of e-cigarettes and flavoured liquids may enhance the effects of a nicotine product standard on smoking in vulnerable populations. In: SRNT 30th Annual Meeting, March 2024, Edinburgh UK. PPS7-2. 2024.
- NCT04090879. Low nicotine content cigarettes in vulnerable populations: affective disorders. clinicaltrials.gov/ct2/show/NCT04090879 (first received 16 September 2019).
- NCT04092101. Low nicotine content cigarettes in vulnerable populations: opioid use disorder. Clinicaltrials.gov/Ct2/Show/NCT04092101 (first received 17 September 2019).
- NCT04092387. Low nicotine content cigarettes in vulnerable populations: women of reproductive age. Clinicaltrials.gov/Ct2/Show/NCT04092387 (first received 17 September 2019).

### Hoeppner 2024 {published data only}

- Hoeppner BB, Eddie D, Schick M, Hoeppner SS, Kelly L, Kelly JF. Feasibility of and reactivity to ecological momentary assessment (EMA) during electronic cigarette use initiation in adults who smoke daily. European Journal of Psychiatry 2024;38(3):100247. [DOI: 10.1016/j.ejpsy.2023.100247]

### Holliday 2019 {published data only}

- \*Holliday R, Preshaw PM, Ryan V, Sniehotta FF, McDonald S, Bauld L, et al. A feasibility study with embedded pilot randomised controlled trial and process evaluation of electronic cigarettes for smoking cessation in patients with periodontitis. Pilot and Feasibility Studies 2019;5:74.
- Holliday R, McColl E, Ryan V, McDonald S, Sniehotta F, Bauld L, et al. E-cigarettes for smoking cessation in patients with periodontitis: pilot RCT. In: IADR International Association for Dental Research, Vancouver, Canada. 2019.
- ISRCTN17731903. Feasibility study of e-cigarettes in periodontitis. www.isrctn.com/ISRCTN17731903 (first received 27 September 2016).

### Humair 2014 {published data only}

- Humair J-P, Tango R. Can e-cigarette help patients to reduce or stop smoking in primary care practice? Journal of General Internal Medicine 2014;29:S480.

### Ikonomidis 2018 {published data only}

- \*Ikonomidis I, Vlastos D, Kourea K, Kostelli G, Varoudi M, Pavlidis G, et al. Electronic cigarette smoking increases arterial stiffness and oxidative stress to a lesser extent than a single conventional cigarette: an acute and chronic study. Circulation 2018;137(3):303-6.
- NCT03039920. The effects of electronic cigarette smoking on the arterial wall and endothelial glycocalyx properties of smokers. clinicaltrials.gov/ct2/show/NCT03039920 (first received 1 February 2017).

### Ikonomidis 2020a {published data only}

- Ikonomidis I, Katogiannis K, Kostelli G, Kourea K, Kyriakou E, Kypraiou A, et al. Effects of electronic cigarette on platelet and vascular function after four months of use. Food and Chemical Toxicology 2020;141:111389.

### Ikonomidis 2020b {published and unpublished data}

- Ikonomidis I, Katogiannis K, Kostelli G, Kourea K, Kyriakou E, Kypraiou A, et al. Effects of electronic cigarette on platelet and vascular function after one month of use. European Heart Journal; Conference: European Society of Cardiology Congress, ESC 2020 2020;41(Suppl 2):2359.

### Ikonomidis 2024 {published data only}

- Ikonomidis I, Katogiannis K, Kourea K, Kostelli G, Pavlidis G, Thymis J, et al. Differential effects of heat-not-burn, electronic, and conventional cigarettes on endothelial glycocalyx. European Heart Journal. Imaging Methods & Practice 2023;7(1):1. [DOI: 10.1093/ehjimp/qyad008]
- Ikonomidis I, Katogiannis K, Kourea K, Kostelli G, Pavlidis G, Thymis J, et al. Differential effects of heat-not-burn, electronic cigarettes and conventional cigarettes on endothelial glycocalyx. European Heart Journal 2024;45(Suppl 1):ehae666.3388. [DOI: 10.1093/eurheartj/ehae666.3388]

### Ioakeimidis 2018 {published data only}

- Ioakeimidis N, Vlachopoulos C, Georgakopoulos C, Abdelrasoul M, Skliros N, Katsi V, et al. Smoking cessation rates with varenicline and electronic cigarettes in relapsed smokers with a history of acute coronary syndrome. European Heart Journal 2018;39(Suppl 1):242.

### Kale 2025 {published data only}

- \*Kale D, Beard E, Marshall A-M, Pervin J, Wu Q, Ratschen E, et al. Providing an e-cigarette starter kit for smoking cessation and reduction as adjunct to usual care to smokers with a mental health condition: findings from the ESCAPE feasibility study. BMC Psychiatry 2025;25(1):13. [DOI: 10.1186/s12888-024-06387-7]
- ISRCTN17691451. ESCAPE: E-cigarettes for smoking cessation and reduction in people with mental illness [E-cigarettes for smoking cessation and reduction in people with mental illness - a randomised pilot feasibility trial]. isrctn.com/ISRCTN17691451 (first received 30 September 2021). [DOI: 10.1186/ISRCTN17691451]

### Kanobe 2022\* {published data only}

- \*Kanobe MN, Jones BA, Nelson P, Brown BG, Chen P, Makena P, et al. Part three: a randomized study to assess biomarker changes in cigarette smokers switched to Vuse Solo or abstinence. Scientific Reports 2022;12:20658. [DOI: 10.1038/s41598-022-25054-z]
- Kanobe MN, Nelson PR, Brown BG, Chen P, Patrudu M, Caraway JW, et al. Changes in biomarkers of exposure and potential harm in smokers switched to Vuse Vibe or Vuse Ciro electronic nicotine delivery systems. Toxics 2023;11(7):564. [DOI: 10.3390/toxics11070564]
- NCT03170674. CSD170501: Study to assess biomarkers of tobacco exposure in smokers during in-clinic confinement switch to an electronic cigarette. clinicaltrials.gov/ct2/show/NCT03170674 (first received 31 May 2017).

### Katz 2025 {published data only}

- \*Katz B, Medina N, O'Connor S, Coleman S, Irvin C, Kaminsky D, et al. Comparison of cardiopulmonary effects of cigarettes and e-cigarettes in individuals with chronic obstructive pulmonary disease (COPD). Drug and Alcohol Dependence 2025;267(Suppl):112271.
- NCT05610514. Pulmonary and cardiac effects of e-cigarette use in pulmonary patients who smoke cigarettes. clinicaltrials.gov/ct2/show/NCT05610514 first received 9 November 2022.

### Kerr 2020 {published and unpublished data}

- \*Kerr D. Chapter 5: A randomised controlled trial investigating the cardiovascular effects of e-cigaretttes in comparison to nicotine replacement patches. In: Studies into the Cardiovascular and Respiratory Effects of Electronic Cigarettes (PhD Thesis). Publisher not available, 27 September 2020.
- NCT03358953. The cardiovascular impacts of electronic cigarettes in comparison to the use of nicotine replacement patches (VAPOUR). clinicaltrials.gov/ct2/show/NCT03358953 (first received 2 December 2017).

### Kimber 2021 {published data only}

[doi: 10.1016/j.addbeh.2021.106909]

- Kimber CF, Soar K, Dawkins LE. Changes in puffing topography and subjective effects over a 2-week period in e-cigarette naive smokers: effects of device type and nicotine concentrations. Addictive Behaviors 2021;118:106909. [DOI: 10.1016/j.addbeh.2021.106909]

### Klonizakis 2022 {published data only}

- \*Klonizakis M, Gumber A, McIntosh E, Brose LS. Medium-and longer-term cardiovascular effects of e-cigarettes in adults making a stop-smoking attempt: a randomized controlled trial. BMC Medicine 2022;20(1):276. [DOI: 10.1186/s12916-022-02451-9]
- Jones G, McIntosh E, Brose LS, Klonizakis M. Participant experiences of a quit smoking attempt through either Nicotine Replacement Therapy (NRT) methods or the use of an e-cigarette. Journal of Addiction Medicine 2022;16(3):272-7.
- Klonizakis M, Crank H, Gumber A, Brose LS. Smokers making a quit attempt using e-cigarettes with or without nicotine or prescription nicotine replacement therapy: impact on cardiovascular function (ISME-NRT) - a study protocol. BMC Public Health 2017;17(1):293.
- Klonizakis M, Gumber A, McIntosh E, Brose LS. Short-term cardiovascular effects of e-cigarettes in adults making a stop-smoking attempt: a randomized controlled trial. Biology 2021;10(11):1208. [DOI: 10.3390/biology10111208]
- NCT03061253. E-cigarettes and cardiovascular function (ISME-NRT). clinicaltrials.gov/ct2/show/NCT03061253 (first received 23 February 2017).

### Kouroutzoglou 2024 {published data only}

- Kouroutzoglou A, Ioakeimidis N, Terentes-Printzios D, Tsabras T, Katsi V, Lazaros G, et al. Smoking cessation medicines and e-cigarettes in smokers with obesity. European Heart Journal 2024;45(Suppl 1):ehae666.2955. [DOI: 10.1093/eurheartj/ehae666.2955]

### Kumral 2016 {published data only}

- Kumral TL, Saltürk Z, Yildirim G, Uyar Y, Berkiten G, Atar Y, et al. How does electronic cigarette smoking affect sinonasal symptoms and nasal mucociliary clearance? Royal Belgian Society for Ear, Nose, Throat, Head and Neck Surgery (B-ENT) 2016;12(1):17-21.

### Lee 2018 {published data only}

- \*Lee SM, Tenney R, Wallace AW, Arjomandi M. E-cigarettes versus nicotine patches for perioperative smoking cessation: a pilot randomized trial. PeerJ 2018;6(9):e5609.
- Lee SM, Tenney R, Wallace A, Arjojmandi M. The end perioperative smoking pilot study: a randomized trial comparing e-cigarettes versus nicotine patch. Canadian Journal of Anesthesia 2017;64(1 Suppl 1):S48-9.
- Lee SM, Tenney R, Wallace A, Arjomandi M. Exploring the teachable moment: facilitators and barriers to perioperative smoking cessation, a qualitative study. Canadian Journal of Anesthesia (Conference: 2017 Annual Meeting of the Canadian Anesthesiologists' Society, Canada) 2017;61(Suppl 1):S46-7.
- NCT02482233. A pilot randomized controlled clinical trial - "Electronic nicotine delivery device (e-cigarette) for perioperative smoking cessation in veterans". clinicaltrials.gov/show/NCT02482233 (first received 17 June 2015).

### Lee 2019 {published data only}

- \*Lee SH, Ahn SH, Cheong YS. Effect of electronic cigarettes on smoking reduction and cessation in Korean male smokers: a randomized controlled study. Journal of the American Board of Family Medicine 2019;32(4):567-74.
- KCT0001277. Effect of an electronic cigarette for smoking reduction and cessation in Korean male smokers: a randomized, controlled study. https://cris.nih.go.kr/cris/search/detailSearchEn.do?seq=KCT0001277 (first received 2014).

### Lucchiari 2022 {published data only}

- \*Lucchiari C, Masiero M, Mazzocco K, Veronesi G, Maisonneuve P, Jemos C, et al. Nicotine-free e-cigarettes might promote tobacco smoking reduction better than nicotine delivery devices: results of a double-blind randomized controlled trial at 1 year. Current Oncology (Toronto, Ont.) 2022;29(11):8579-90. [DOI: 10.3390/curroncol29110676]
- Lucchiari C, Masiero M, Mazzocco K, Veronesi G, Maisonneauve P, Jemos C, et al. Benefits of e-cigarettes in smoking reduction and in pulmonary health among chronic smokers undergoing a lung cancer screening program at 6 months. Addictive Behaviours 2020;103:106222.
- Lucchiari C, Masiero M, Veronesi G, Maisonneuve P, Spina S, Jemos C, et al. Benefits of e-cigarettes among heavy smokers undergoing a lung cancer screening program: randomized controlled trial protocol. JMIR Research Protocols 2016;5(1):e21. [PMID: 26842790]
- Masiero M, Lucchiari C, Mazzocco K, Veronesi G, Maisonneuve P, Jemos C, et al. Corrigendum: E-cigarettes may support smokers with high smoking-related risk awareness to stop smoking in the short run: preliminary results by randomized controlled trial. Nicotine & Tobacco Research 2020;22(4):594-5.
- Masiero M, Lucchiari C, Mazzocco K, Veronesi G, Maisonneuve P, Jemos C, et al. E-cigarettes may support smokers with high smoking-related risk awareness to stop smoking in the short run: preliminary results by randomized controlled trial. Nicotine & Tobacco Research 2019;21(1):119-26.
- NCT02422914. Benefits of tobacco free cigarette among heavy smokers undergoing a lung cancer screening program: a randomized controlled study. clinicaltrials.gov/show/NCT02422914 (first received 14 January 2015).
- Pravettoni G, Masiero M, Lucchiari C, Maisenneuve P, Mazzocco K, Veronesi G. The role of electronic cigarettes in smoking cessation among heavy smokers undergoing a lung cancer screening program: preliminary results of a randomized controlled study. Psycho-Oncology 2016;25:72. [DOI: 10.1002/pon.4082]

### Martinez 2021 {published data only}

- \*Martinez U, Simmons VN, Sutton SK, Drobes DJ, Meltzer LR, Brandon KO, et al. Targeted smoking cessation for dual users of combustible and electronic cigarettes: a randomised controlled trial. Lancet. Public Health 2021;6(7):e500-9.
- Byrne M, Simmons V, Martinez U, Sutton S, Brandon K, Brandon T. Cost effectiveness of a smoking cessation intervention for dual users of combustible and electronic cigarettes. In: Society for Research on Nicotine and Tobacco, Annual Meeting, March 2022, Baltimore USA. 2022.
- Gore LR, Sutton SK, Martinez U, Brandon KO, Simmons VN, Brandon TH. Mediators of initial abstinence for an extended self help smoking cessation intervention with dual users of combustible and electronic cigarettes. In: Society for Research on Nicotine and Tobacco, Annual Meeting, March 2022, Baltimore USA. PS3-7. 2022.
- Martinez U, Simmons VN, Brandon KO, Quinn GP, Brandon TH. Examining smoking and vaping behaviors, expectancies, and cessation outcomes between bisexual and heterosexual individuals. Behavioral Medicine (Washington, D.C.) 2023;49(4):392-401. [DOI: 10.1080/08964289.2022.2077295]
- Meltzer LR, Simmons VN, Sutton SK, Drobes DJ, Quinn GP, Meade CD, et al. A randomized controlled trial of a smoking cessation self-help intervention for dual users of tobacco cigarettes and e-cigarettes: intervention development and research design. Contemporary Clinical Trials 2017;60:56-62.
- NCT02416011. Smoking cessation self-help for dual users of tobacco cigarettes and e-cigarettes. clinicaltrials.gov/ct2/show/NCT02416011 (first received 18 August 2020).
- Sutton SK, Brandon KO, Harrell PT, Martínez Ú, Simmons VN, Gore LR, et al. Identifying prospective subpopulations of combustible and electronic cigarette dual users in the United States via finite mixture modeling. Addiction (Abingdon, England) 2022;117(9):2493-503. [DOI: 10.1111/add.15906]

### Martner 2019 {published data only (unpublished sought but not used)}

- Martner SG, Dallery J. Technology-based contingency management and e-cigarettes during the initial weeks of a smoking quit attempt. Journal of Applied Behavior Analysis 2019;52(4):928-43.

### McRobbie 2015 {published data only}

- \*McRobbie H, Phillips A, Goniewicz ML, Smith KM, Knight-West O, Przulj D, et al. Effects of switching to electronic cigarettes with and without concurrent smoking on exposure to nicotine, carbon monoxide, and acrolein. Cancer Prevention Research (Philadelphia, Pa.) 2015;8(9):873-8.
- McRobbie H, Goniewicz M, Phillips A, Myers-Smith K, West O, Hajek P. Effects of the use of electronic cigarettes with and without concurrent smoking on acrolein delivery. In: Society for Research on Nicotine and Tobacco, 20th Annual Meeting, Seattle, Washington. POS4-33. 2014:13.
- NCT01714778. Toxins and delivery in E-cigarette users (TADEUS). clinicaltrials.gov/ct2/show/NCT01714778 (first received 26 October 2012).

### Meier 2017 {published data only}

- Meier E, Wahlquist AE, Heckman BW, Cummings KM, Froeliger B, Carpenter MJ. A pilot randomized crossover trial of electronic cigarette sampling among smokers. Nicotine & Tobacco Research 2017;19(2):176-82.

### Morphett 2022a {unpublished data only}

- \*Morphett K, Fraser D, Borland R, Hall W, Walker N, Bullen C, et al. A pragmatic randomized comparative trial of e-cigarettes and other nicotine products for quitting or long-term substitution in smokers. Nicotine & Tobacco Research 2022;24(7):1079-88. [DOI: 10.1093/ntr/ntab266]
- ACTRN12612001210864. Can using nicotine as a long-term substitute enhance smoking cessation over using it only as a cessation aid? [An open-label randomised pragmatic policy trial examining effectiveness of short-term use of Nicotine Replacement Therapy (NRT) vs short- or long-term use of NRT vs short- or long-term use of NRT or electronic nicotine delivery systems for smoking cessation in cigarette smokers]. https://www.anzctr.org.au/Trial/Registration/TrialReview.aspx?ACTRN=12612001210864 (first received 15 November 2012).
- Fraser D, Borland R, Gartner C. Protocol for a randomised pragmatic policy trial of nicotine products for quitting or long-term substitution in smokers. BMC Public Health 2015;15:1026.

### Morphett 2022b {published and unpublished data}

- \*Morphett K, Boyd M, Puljevic C, Gilks C, Bonevski B, Borland R, et al. Do nicotine vaping products increase quitting among priority population groups over standard smoking cessation therapy? A pragmatic randomised partial cross-over trial. In: Society for Research on Nicotine and Tobacco (SRNT), Annual Meeting 2022. SYM17-4. 2022.
- ACTRN12618000408280. Cessation And Relapse Prevention (CARP) trial: nicotine vaporisers compared to standard nicotine replacement therapy for smoking cessation among people with co-morbidities. www.anzctr.org.au/ACTRN12618000408280.aspx (first received 21 March 2018).

### Morris 2022\* {published data only}

- \*Morris P, McDermott S, Chapman F, Verron T, Cahours X, Stevenson M, et al. Reductions in biomarkers of exposure to selected harmful and potentially harmful constituents following exclusive and partial switching from combustible cigarettes to myblu TM electronic nicotine delivery systems (ENDS). Internal and Emergency Medicine 2022;17(2):397-410. [DOI: 10.1007/s11739-021-02813-w]
- NCT04429932. A study to evaluate nicotine uptake and biomarkers in adult smokers using mybluTM electronic cigarettes. clinicaltrials.gov/ct2/show/NCT04429932 (first received 7 June 2021).
- NCT04430634. A study to evaluate nicotine uptake and biomarkers in smokers using mybluTM electronic cigarettes [An open-label, randomized, crossover study to assess nicotine uptake, tobacco-related biomarkers of exposure, biomarkers of potential harm, and puff topography with use of mybluTM electronic cigarettes in adult smokers]. Clinicaltrials.gov/Ct2/Show/NCT04430634 (first received 7 June 2021).

### Myers-Smith 2022 {published data only}

- \*Myers Smith K, Phillips-Waller A, Pesola F, McRobbie H, Przulj D, Orzol M, et al. E-cigarettes versus nicotine replacement treatment as harm reduction interventions for smokers who find quitting difficult: randomised controlled trial. Addiction (Abingdon, England) 2022;117(1):224-33. [DOI: 10.1111/add.15628]
- ISRCTN13288677. Can electronic cigarettes and nicotine replacement treatment help reduce smoking in smokers who struggle to quit? www.isrctn.com/ISRCTN13288677 (first received 17 March 2017).

### NCT02648178 {published data only}

- NCT02648178. Evaluation of appeal and impact of e-cigarettes among chronic smokers with smoking-related cancers. clinicaltrials.gov/show/NCT02648178 (first received 5 January 2016).

### NCT02918630 {published data only}

- NCT02918630. E-cigarettes to promote smoking reduction among individuals with schizophrenia. clinicaltrials.gov/ct2/show/NCT02918630 (first received 5 July 2018).

### NCT03113136 {published data only}

- \*NCT03113136. Examination of low wattage and high wattage e-cigarettes (SWITCH). clinicaltrials.gov/ct2/show/study/NCT03113136 (first received 13 April 2017).
- Ganapathy V, Chinnaiyan M, Brobst D, Sadhasivam B, Nshimiyimana JD, Zhao Y, et al. Measuring oral DNA damage to evaluate electronic cigarette use as a tobacco harm reduction strategy. Cancer Research 2024;84(7):LB086. [DOI: 10.1158/1538-7445]

### Nides 2014\* {published data only}

- \*Nides MA, Leischow SJ, Bhatter M, Simmons M. Nicotine blood levels and short-term smoking reduction with an electronic nicotine delivery system. American Journal of Health Behavior 2014;38(2):265-74.
- NCT01898169. Evaluation of short-term safety and use patterns of an electronic nicotine delivery system. clinicaltrials.gov/ct2/show/NCT01898169 (first received 12 July 2013).

### Okuyemi 2022 {published data only}

- \*Okuyemi KS, Ojo-Fati O, Aremu TO, Friedrichsen CS, Grude L, Oyenuga M, et al. A randomized trial of nicotine vs. no-nicotine e-cigarettes among African American smokers: changes in smoking and tobacco biomarkers. Nicotine & Tobacco Research 2022;24(4):555-63. [DOI: 10.1093/ntr/ntab212]
- NCT03084315. Changes in biomarkers associated with use of electronic cigarettes [Changes in biomarkers associated with use of electronic cigarettes among African American menthol and non-menthol smokers]. clinicaltrials.gov/ct2/show/NCT03084315 (first received 20 March 2017).

### Oncken 2015 {published data only}

- \*Oncken CA, Litt MD, McLaughlin LD, Burki NA. Nicotine concentrations with electronic cigarette use: effects of sex and flavor. Nicotine & Tobacco Research 2015;17(4):473-8.
- NCT01775787. Effects of electronic cigarettes on nicotine concentrations (ECIG). clinicaltrials.gov/ct2/show/NCT01775787 (first received 25 January 2013).
- Swedeh MA, Oncken C, Burki NK. Acute effects of electronic cigarettes on airway function in human subjects. In: American Thoracic Society International Conference Abstracts. Vol. 189. 2014:A4089.

### Ozga-Hess 2019 {published data only}

- Ozga-Hess JE, Felicione NJ, Ferguson SG, Dino G, Elswick D, Whitworth C, et al. Piloting a clinical laboratory method to evaluate the influence of potential modified risk tobacco products on smokers' quit-related motivation, choice, and behaviour. Addictive Behaviors 2019;99:106105.

### Pacifici 2015 {published data only}

- Pacifici R, Pichini S, Graziano S, Pellegrini M, Massaro G, Beatrice F. Successful nicotine intake in medical assisted use of e-cigarettes: a pilot study. International Journal of Environmental Research and Public Health 2015;12(7):7638-46.

### Pericot-Valverde 2025 {published data only}

- \*Pericot-Valverde I, Heo M, Nahvi S, Barron J, Voss S, Ortiz EG, et al. Effects of e-cigarettes on combustible cigarette smoking among adults with opioid use disorder on buprenorphine: single arm ERASER pilot trial. Nicotine & Tobacco Research 2025;27(5):865-3. [DOI: 10.1093/ntr/ntae260]
- NCT06277271. E-cigarettes as a harm reduction strategy. https://clinicaltrials.gov/study/NCT06277271 (first received 19 January 2024).

### Piper 2025 {published data only}

- \*Piper ME, Schlam TR, Donny EC, Kobinsky K, Matthews J, Piasecki TM, et al. The impact of three alternate nicotine-delivery products on combusted cigarette use: a randomized controlled trial. Nicotine & Tobacco Research 2025;27(2):317-25. [DOI: 10.1093/ntr/ntae014]
- NCT04084210. Impact of alternative nicotine-delivery products on combustible cigarette use [Understanding the real-world impact of the use of three alternate nicotine-delivery products on combustible cigarette use]. clinicaltrials.gov/ct2/show/NCT04084210 (first received 10 September 2019).
- Piasecki T, Sluts WS. The effects of three alternative nicotine delivery products on own-cigarette abstinence in a randomized controlled switching trial. In: Society for Research on Nicotine and Tobacco (SRNT) 30th Annual Meeting, 20-23 March 2024, Edinburgh UK. 2024:POS5-125 Rapids.
- Piasecki TM, Slutske WS, Bolt DM, Jorenby DE, Piper ME. Effects of very low nicotine cigarettes, e-cigarettes, and nicotine patches on daily own-cigarette abstinence in a randomized controlled switching trial. Drug and Alcohol Dependence 2025;268:112576. [DOI: 10.1016/j.drugalcdep.2025.112576]
- Piper M, Schlam T, Donny E, Jorenby D. The real-world impact of three alternative nicotine delivery products on combustible cigarette use. In: Society for Research on Nicotine and Tobacco (SRNT) 29th Annual Meeting, 1-4 March. 2023:22 SYM17-2.
- Piper M. What motivates e-cigarette or very low nicotine cigarette use during a switching study? In: Society for Research on Nicotine and Tobacco (SRNT), 20-23 March 2024, Edinburgh UK. 2024:POS3-108.

### Polosa 2011\* {published data only}

- \*Polosa R, Caponnetto P, Morjaria JB, Papale G, Campagna D, Russo C. Effect of an electronic nicotine delivery device (e-cigarette) on smoking reduction and cessation: a prospective 6-month pilot study. BMC Public Health 2011;11:786.
- NCT01195597. Smoking cessation and reduction with an electronic nicotine delivery device (ENDD). clinicaltrials.gov/ct2/show/NCT01195597 (first received 6 September 2010).
- Polosa R, Morjaria JB, Caponnetto P, Campagna D, Russo C, Alamo A, et al. Effectiveness and tolerability of electronic cigarette in real-life: a 24-month prospective observational study. Internal and Emergency Medicine 2014;9(5):537-46.

### Polosa 2014b\* {published data only}

- NCT02124200. High cessation rates in smokers using personal vaporizers (VAPECIG). clinicaltrials.gov/ct2/show/NCT02124200 (first received 28 April 2014).
- Polosa R, Caponnetto P, Maglia M, Morjaria JB, Russo C. Success rates with nicotine personal vaporizers: a prospective 6-month pilot study of smokers not intending to quit. BMC Public Health 2014;14:1159.

### Polosa 2015\* {published data only}

- Polosa R, Caponnetto P, Cibella F, Le-Houezec J. Quit and smoking reduction rates in vape shop consumers: a prospective 12-month survey. International Journal of Environmental Research and Public Health 2015;12(4):3428-38.

### Pope 2024 {published data only}

[doi: ]

- \*Pope I, Clark LV, Clark A, Ward E, Belderson P, Stirling S, et al. CessatiOn of Smoking Trial in the Emergency Department (COSTED): a multicentre randomised controlled trial. Emergency Medicine Journal 2024;41(5):276-82. [DOI: 10.1136/emermed-2023-213824]
- Belderson P, Ward E, Clark L, Stirling S, Clark A, Notley C, et al. Cessation of smoking trial in the emergency department (COSTED): a multi-centre, randomised controlled trial. In: SRNT 30th Annual Meeting, 2024 March 20-23, Edinburgh, UK. 2024:POS2-167.
- Belderson P, Ward E, Pope I, Notley C. Selecting an e-cigarette for use in smoking cessation interventions and healthcare services: findings from patient and public consultation for the COSTED trial. BMJ Open 2024;13(3):1-9. [DOI: 10.1136/bmjopen-2023-078677]
- Li J, Wu Q, Parrott S, Pope I, Clark LV, Clark A, et al. Cost-utility analysis of provision of e-cigarette starter kits for smoking cessation in emergency departments: an economic evaluation of a randomized controlled trial. Addiction (Abingdon, England) 2025;120(2):368-79. [DOI: 10.1111/add.16698]
- NCT04854616. Cessation of smoking trial in the emergency department (COSTED). clinicaltrials.gov/ct2/show/NCT04854616 (date first received 22 April 2021).
- Notley C, Belderson P, Ward E, Clark LV, Clark A, Stirling S, et al. The context of the emergency department as a location for a smoking cessation intervention - process evaluation findings from the cessation of smoking trial in the emergency department trial. Nicotine & Tobacco Research 2025;27(5):909-16. [DOI: 10.1093/ntr/ntae223]
- Notley C, Clark L, Belderson P, Ward E, Clark AB, Parrott S, et al. Cessation of smoking trial in the emergency department (CoSTED): protocol for a multicentre randomised controlled trial. BMJ Open 2023;13(1):e064585.
- Pope I, Notley C, Boyle A. Results of the cessation of smoking trial in the emergency department (COSTED). Emergency Medicine Journal 2023;40(12):873-4.
- Pope I, Notley C. Cessation of smoking trial in the emergency department. In: SRNT 29th Annual Meeting; 2023 Mar 1-4; San Antonio (TX), USA. 2023:119-20 POS2-14.
- Pope I, Rashid S, Iqbal H, Belderson P, Ward E, Clark L, et al. Engagement with stop smoking services after referral or signposting: a mixed methods study. Nicotine & Tobacco Research 2025;27(2):360-3. [DOI: 10.1093/ntr/ntae159]
- Ward E, Belderson P, Clark A, Stirling S, Clark L, Pope I, et al. How do people quit smoking using e-cigarettes? A mixed-methods exploration of participant smoking pathways following receiving an opportunistic e-cigarette-based smoking cessation intervention. Addiction (Abingdon, England) 2024;119:2185-96. [DOI: 10.1111/add.16633]

### Pratt 2016 {published data only}

- Pratt SI, Sargent J, Daniels L, Santos MM, Brunette M. Appeal of electronic cigarettes in smokers with serious mental illness. Addictive Behaviors 2016;59:30-4.

### Pratt 2022 {published and unpublished data}

- \*Pratt SI, Brunette MF, Ferron JC, Santos M, Sargent J, Xie H. E-cigarette provision to promote switching in cigarette smokers with serious mental illness - a randomized trial. Nicotine & Tobacco Research 2022;6(24):1405-12. [DOI: 10.1093/ntr/ntac082]
- Ferron J. What predicts harm reduction in adults with severe mental illness provided e-cigarettes? In: Society for Research on Nicotine and Tobacco (SRNT), 20-23 March 2024, Edinburgh UK. 2024.
- NCT03050853. The appeal and impact of e-cigarettes in smokers with SMI. clinicaltrials.gov/ct2/show/NCT03050853 (first received 13 February 2017).
- Pratt S, Ferron J, Sargent J, Brunette M. Assessing the effect of e-cigarettes versus usual smoking on NNAL among chronic smokers with serious mental illness. In: Society for Research on Nicotine and Tobacco (SRNT) 29th Annual Meeting, 1-4 March 2023. Publisher not available, 2023:73 (PPS22-5).
- Santos M. Novel evaluation of the relationship between trajectories of e-cigarette initiation and cigarette reduction while switching for harm reduction. In: Society for Research on Nicotine and Tobacco (SRNT), 20-23 March 2024, Edinburgh UK. Publisher not available, 2024.
- Sargent JD, Pratt SI, Brunette MF, Ferron JC, Santos MM, Stoolmiller M. Level and timing of product substitution in a trial of e-cigarettes for smokers not interested in quitting. Tobacco Induced Diseases 2024;22:101201591. [DOI: 10.18332/tid/189220]

### Price 2022 {published data only}

- \*Price AD, Coffey M, Houston L, Cook PA. Evaluation of a pharmacy supported e-cigarette smoking cessation intervention in Northwest England. BMC Public Health 2022;22:1326. [DOI: 10.1186/s12889-022-13711-x]

### Pulvers 2018 {published data only}

- Pulvers K, Emami AS, Nollen NL, Romero DR, Strong DR, Benowitz NL, et al. Tobacco consumption and toxicant exposure of cigarette smokers using electronic cigarettes. Nicotine & Tobacco Research 2018;20(2):206-14.

### Pulvers 2020 {published and unpublished data}

- \*Pulvers K, Nollen NL, Rice M, Schmid CH, Qu K, Benowitz NL, et al. Effect of pod e-cigarettes vs cigarettes on carcinogen exposure among African American and Latinx smokers: a randomized clinical trial. JAMA Network Open 2020;3(11):e2026324.
- Arnold M, Nollen N, Mayo M, Ahluwalia J, Leavens E, Zhang G, et al. Dual use of cigarettes and e-cigarettes in African American and LatinX smokers: secondary analyses from a randomised controlled e-cigarettes switching trial. In: Society for Research on Nicotine and Tobacco (SRNT) 2021 Annual Meeting, February 24-27 2021, virtual. Publisher not available as virtual session, 2021:31 (POD4-2).
- Arnold MJ, Nollen NL, Mayo MS, Ahluwalia JS, Leavens EL, Zhang G, et al. Harm reduction associated with dual use of cigarettes and e-cigarettes in Black and Latino smokers: secondary analyses from a randomized controlled e-cigarette switching trial. Nicotine & Tobacco Research 2021;23(11):1972-6. [DOI: 10.1093/ntr/ntab069]
- Leavens E, Nollen N, Ahluwalia J, Mayo M, Rice M, Brett E, et al. Changes in dependence, withdrawal, and craving among adult smokers who switch to nicotine salt pod-based e-cigarettes. Journal of Clinical and Translational Science 2021;5(Suppl 1):85-6. [DOI: 10.1017/cts.2021.622]
- Leavens EL, Nollen N, Ahluwalia J, Mayo M, Rice M, Brett E, et al. Changes in dependence, withdrawal, and craving among adult smokers who switch to nicotine salt pod-based e-cigarettes. In: Society for Research on Nicotine and Tobacco (SRNT) 2021 Annual Meeting, February 24-27 2021, virtual. Publisher not available as virtual session, 2021:96 (POD53-1), 116 (C-6).
- Leavens EL, Nollen N, Ahluwalia JS, Mayo MS, Rice M, Brett EI, et al. Changes in dependence, withdrawal, and craving among adult smokers who switch to nicotine salt pod-based e-cigarettes. Addiction (Abingdon, England) 2021;117(1):207-15. [DOI: 10.1111/add.15597]
- Lee SC, Maglalang DD, Avila JC, Leavens EL, Nollen NL, Pulvers K, et al. Change in e-cigarette risk perception and smoking behavior of Black and Latinx individuals who smoke. Drug and Alcohol Dependence 2023;245:109824. [DOI: 10.1016/j.drugalcdep.2023.109824]
- NCT03511001. Effects among smokers who use and do not use e-cigarettes. https://clinicaltrials.gov/ct2/show/NCT03511001 (first received 10 April 2018).
- Nollen N, Lambert L, Leavens E, Ahluwalia J, Rice M, Pulvers K. Does menthol flavoring help black and LatinX adult menthol combustible cigarette smokers switch to e-cigarettes?: secondary analyses from a randomized clinical trial. In: Society for Research on Nicotine and Tobacco, Annual Meeting, March 2022, Baltimore USA. 2022.
- Nollen NL, Leavens EL, Ahluwalia JS, Rice M, Mayo MS, Pulvers K. Menthol versus non-menthol flavouring and switching to e-cigarettes in black and Latinx adult menthol combustible cigarette smokers: secondary analyses from a randomised clinical trial. Tobacco Control 2022;EPub:no pagination. [DOI: 10.1136/tobaccocontrol-2021-057180]
- Rice M, Nollen N, Woodcock A, Benowitz N, Ahluwalia J, Pulvers K. Effect of marijuana use on smokers switching to e-cigarettes in a randomised control trial. In: Society for Research on Nicotine and Tobacco (SRNT) 2021 Annual Meeting, February 24-27 2021, virtual. 2021:125 (SRNT C-44).
- Rice M, Nollen NL, Ahluwalia JS, Benowitz N, Woodcock A, Pulvers K. Effects of marijuana use on smokers switching to e-cigarettes in a randomized clinical trial. Nicotine & Tobacco Research 2022;24(7):994-1002. [DOI: 10.1093/ntr/ntac008]
- Rubenstein D, Sokolovsky A, Aston E, Nollen N, Schmid C, Pulvers K, et al. Predictors of smoking reduction among African American and LatinX smokers in a randomised controlled trial of Juul e-cigarettes. In: Society for Research on Nicotine and Tobacco (SRNT) 2021 Annual Meeting, February 24-27 2021, virtual. 2021:129 (C-56).
- Rubenstein D, Sokolovsky AW, Aston ER, Nollen NL, Schmid CH, Rice M, et al. Predictors of smoking reduction among African American and Latinx smokers in a randomized controlled trial of JUUL e-cigarettes. Addictive Behaviors 2021;122:107037. [DOI: 10.1016/j.addbeh.2021.107037]

### Rabenstein 2024 {published data only}

- Rabenstein A, Czermak L, Fischer E, Kahnert K, Pogarell O, Jorres RA, et al. Implications of switching from conventional to electronic cigarettes on quality of life and smoking behaviour: results from the EQualLife Trial. European Addiction Research 2024;30(4):207-15. [DOI: 10.1159/000536255]

### Rose 2023\* {published data only}

- \*Rose JE, Frisbee S, Campbell D, Salley A, Claerhout S, Davis JM. Smoking reduction using electronic nicotine delivery systems in combination with nicotine skin patches. Psychopharmacology 2023;240:1901-9. [DOI: 10.1007/s00213-023-06401-y]
- NCT03492463. The role of nicotine dose and route of delivery in affecting adoption of e-cigarettes and reducing exposure to toxic combustion products (ENDS-Switch). clinicaltrials.gov/ct2/show/study/NCT03492463 (first received 10 April 2018).
- Rose JE, Frisbee S, Campbell D, Salley A, Claerhout S, Davis JM. Correction to: Smoking reduction using electronic nicotine delivery systems in combination with nicotine skin patch. Psychopharmacology 2025;Advance online publication:no pagination. [PMID: 10.1007/s00213-025-06868-x]

### Russell 2021\* {published and unpublished data}

- Russell C, McKeganey N, Katsampouris E, Satchwell A, Haseen F. A randomised community-based trial of a closed-system pod e-vapour product and nicotine replacement therapy for cigarette abstinence and reduction. In: Society for Research on Nicotine and Tobacco (SRNT) 2021 Annual Meeting, February 24-27 2021, virtual. 2021:230 (PH-353).

### Scheibein 2020 {published and unpublished data}

- \*Scheibein F, McGirr K, Morrison A, Roche W, Wells JS. An exploratory non-randomized study of a 3-month electronic nicotine delivery system (ENDS) intervention with people accessing a homeless supported temporary accommodation service (STA) in Ireland. Harm Reduction Journal 2020;17(1):73.
- Scheibein F, McGirr K, Morrison A, Roche W, Wells JS. Correction to: an exploratory non-randomized study of a 3-month electronic nicotine delivery system (ENDS) intervention with people accessing a homeless supported temporary accommodation service (STA) in Ireland. Harm Reduction Journal 2021;18(1):113. [DOI: 10.1186/s12954-021-00549-6]

### Sifat 2024 {published data only}

- \*Sifat MS, Alexander AC, Businelle MS, Frank-Pearce SG, Boozary LK, Wagener TL, et al. E-Cigarette switching and financial incentives to promote combustible cigarette cessation among adults accessing shelter services: a pilot study. Drug and Alcohol Dependence Reports 2024;13:1-9. [DOI: 10.1016/j.dadr.2024.100295]

### Skelton 2022 {published data only}

- \*Skelton E, Lum A, Robinson M, Dunlop A, Guillaumier A, Baker A, et al. A pilot randomised controlled trial of abrupt versus gradual smoking cessation in combination with vaporised nicotine products for people receiving alcohol and other drug treatment. Addictive Behaviors 2022;131:107328. [DOI: 10.1016/j.addbeh.2022.107328]
- ACTRN12617000905369. Smoking reduction interventions for smoking cessation. ncbi.nlm.nih.gov/pmc/articles/PMC6953262/ (first received 9 June 2017). [DOI: 10.1002/14651858.CD013183.pub2]
- Lum A, Skelton E, Robinson M, Guillaumier A, Wynne O, Gartner C, et al. Barriers and facilitators to using vaporised nicotine products as smoking cessation aids among people receiving treatment for substance use disorder. Addictive Behaviors 2021;124:107097. [DOI: 10.1016/j.addbeh.2021.107097]

### Smith 2020 {published and unpublished data}

- Smith TT, Heckman BW, Wahlquist AE, Cummings KM, Carpenter MJ. The impact of e-liquid propylene glycol and vegetable glycerin ratio on ratings of subjective effects, reinforcement value, and use in current smokers. Nicotine & Tobacco Research 2020;22(5):791-7.

### Smith 2025 {published and unpublished data}

- \*Smith TT, Crawford A, Wahlquist AE, Cummings KM, Rojewski AM, McClure EA, et al. Pilot study to test the feasibility for a randomized controlled trial of e-cigarettes as harm reduction tools among people who smoke and previously failed to quit with pharmacotherapy. Nicotine & Tobacco Research 2025;27(3):553-7. [DOI: 10.1093/ntr/ntae212]
- NCT05525078. Quit or switch: e-cigarette study. clinicaltrials.gov/ct2/show/NCT05525078 (first received 1 September 2022).
- Smith TT, Wahlquist AE, Cummings KM, Rojewski AM, McClure EA, Toll BA, et al. Pilot investigation of e-cigarettes as a harm reduction tool among smokers who previously failed to quit with pharmacotherapy. In: SRNT 30th Annual Meeting, March 2024, Edinburgh, UK. 2024:PPS25-2A.

### Stein 2016 {published data only}

- Stein MD, Caviness C, Grimone K, Audet D, Anderson BJ, Bailey GL. An open trial of electronic cigarettes for smoking cessation among methadone-maintained smokers. Nicotine & Tobacco Research 2016;18(5):1157-62.

### Strasser 2016 {published data only}

- Strasser AA, Souprountchouk V, Kaufmann A, Blazekovic S, Leone F, Benowitz NL, et al. Nicotine replacement, topography, and smoking phenotypes of e-cigarettes. Tobacco Regulatory Science 2016;2(4):352-62.

### Tattan-Birch 2023 {published data only}

- \*Tattan-Birch H, Kock L, Brown J, Beard E, Bauld L, West R, et al. E-cigarettes to augment stop smoking in-person support and treatment with varenicline (E-ASSIST): a pragmatic randomised controlled trial. Nicotine & Tobacco Research 2023;25(3):395-403. [DOI: 10.1093/ntr/ntac149]
- ISRCTN16931827. A trial to assess the benefit of offering an e-cigarette starter kit to smokers attempting to stop smoking with varenicline. isrctn.com/ISRCTN16931827 (first received 23 August 2018).

### Tseng 2016 {published data only}

- \*Tseng TY, Ostroff JS, Campo A, Gerard M, Kirchner T, Rotrosen J, et al. A randomized trial comparing the effect of nicotine versus placebo electronic cigarettes on smoking reduction among young adult smokers. Nicotine & Tobacco Research 2016;18(10):1937-43.
- Guttentag A, Tuo-Yen T, Shelley D, Thomas K. Analyzing trajectories of acute cigarette reduction post-introduction of an e-cigarette using ecological momentary assessment data. International Journal of Environmental Research and Public Health 2022;19(12):7452. [DOI: 10.3390/ijerph19127452]
- Guttentag A. Do e-cigarettes "Work" for smokers as a step on the way to cessation? An analysis of the temporal and spatial correlates of switching in a harm reduction trial among adult smokers. Dissertation Abstracts International: Section B: The Sciences and Engineering 2021;82:8-B.
- NCT02628964. Assessing the use of electronic cigarettes (e-cigarettes) as a harm reduction strategy. clinicaltrials.gov/show/NCT02628964 (first received 28 September 2015).

### Tuisku 2024 {published data only}

- Tuisku A, Rahkola M, Nieminen P, Toljamo T. Electronic cigcarettes vs varenicline for smoking cessation in adults: a randomized clinical trial. JAMA Internal Medicine 2024;184(8):915-21. [DOI: 10.1001/jamainternmed.2024.1822]
- Tuisku A, Toljamo T, Nieminen P. Relevant data missing in electronic cigarette vs varenicline trial - reply. JAMA Internal Medicine 2024;184(12):1483.

### Valentine 2018 {published data only}

- Valentine GW, Hefner K, Jatlow PI, Rosenheck RA, Gueorguieva R, Sofuoglu M. Impact of e-cigarettes on smoking and related outcomes in veteran smokers with psychiatric comorbidity. Journal of Dual Diagnosis 2018;14(1):2-13.

### Van Staden 2013\* {published data only}

- Van Staden SR, Groenewald M, Engelbrecht R, Becker PJ, Hazelhurst LT. Carboxyhaemoglobin levels, health and lifestyle perceptions in smokers converting from tobacco cigarettes to electronic cigarettes. South African Medical Journal 2013;103(11):865-8.

### Vickerman 2022 {published data only}

- \*Vickerman KA, Carpenter KM, Miles LN, Hsu JM, Watt KA, Brandon TH, et al. A randomized pilot of a tailored smoking cessation quitline intervention for individuals who smoke and vape. Nicotine & Tobacco Research 2022;24(11):1811-20. [DOI: 10.1093/ntr/ntac129]
- NCT03575468. Enhanced e-cigarette coaching intervention for dual users of cigarettes and e-cigarettes. clinicaltrials.gov/ct2/show/NCT03575468 (first received 2 July 2018).
- Vickerman KA, Carpenter KM, Watt K, Miles L, Hsu J, Brandon T, et al. A behavioral smoking cessation intervention for Quitline callers who also use e-cigarettes. In: Society for Research on Nicotine and Tobacco (SRNT) 2021 Annual Meeting; February 24-27 2021 (virtual). 2021:50 (POD14-1).

### Vojjala 2025 {published data only}

- \*Vojjala M, Stevens ER, Nicholson A, Morgan T, Kaneria A, Xiang G, et al. Switching to e-cigarettes as harm reduction among individuals with chronic disease who currently smoke: results of a pilot randomized controlled trial. Nicotine & Tobacco Research 2025;27(1):36-45. [DOI: 10.1093/ntr/ntae158]
- NCT04465318. Electronic cigarettes as a harm reduction strategy among patients with COPD. clinicaltrials.gov/ct2/show/NCT04465318 (first received 10 July 2020).
- Stevens ER, Lei L, Cleland CM, Berger KM, Sherman SE, Vojjala M. Electronic cigarettes as a harm reduction strategy among patients with COPD: protocol for an open-label two arm randomized controlled pilot trial. Addiction Science & Clinical Practice 2022;17(1):2. [DOI: 10.1186/s13722-021-00284-0]
- Vojjala M, Stevens ER, Nicholson A, Morgan T, Kaneria A, Xiang G, et al. Switching to e-cigarettes as a harm reduction tool among individuals with chronic disease who currently smoke: results of a pilot randomised controlled trial. In: SRNT 30th Annual Meeting, 12-15 March 2025, Edinburgh, UK. 2024:POS5-147.
- Vojjala M, Wilker OG, Nicholson A, Morgan T, Rozon I, Stevens ER, et al. Electronic cigarettes and counseling as a harm reduction strategy among patients with COPD, asthma, CAD/PAD. In: SRNT 29th Annual Meeting; 2023 Mar 1-4; San Antonio (TX), USA. 2023:206 (POS4-100).

### Wadia 2016 {published data only}

- Wadia R, Booth V, Yap HF, Moyes DL. A pilot study of the gingival response when smokers switch from smoking to vaping. British Dental Journal 2016;221(11):722-6.

### Wagener 2023 {published data only}

- Wagener T, Beebe L, Businelle M, Carpenter M, Hinton A, Hart J, et al. E-cigarette versus combination nicotine replacement therapy delivered through state quitlines on smoking abstinence following a recent failed quit attempt: a randomized trial. In: Society for Research on Nicotine and Tobacco (SRNT) 29th Annual Meeting; 2023 Mar 1-4; San Antonio (TX), USA. 2023:SYM5-2.

### Walele 2018\* {published data only}

- \*Walele T, Bush J, Koch A, Savioz R, Martin C, O'Connell G. Evaluation of the safety profile of an electronic vapour product used for two years by smokers in a real-life setting. Regulatory Toxicology and Pharmacology 2018;92:226-38. [DOI: 10.1016/j.yrtph.2017.12.010]
- Cravo AS, Bush J, Sharma G, Savioz R, Martin C, Craige S, et al. A randomised, parallel group study to evaluate the safety profile of an electronic vapour product over 12 weeks. Regulatory Toxicology and Pharmacology 2016;81:S1-S14.
- NCT02029196. A randomised, parallel group, multi-centre study to evaluate the safety profile of the ITG EVP G1 product. clinicaltrials.gov/show/NCT02029196 (first received 19 December 2013).
- NCT02143310. A multi-centre study to evaluate the safety of use of electronic vapour products for two years. clinicaltrials.gov/show/NCT02143310 (first received 9 May 2014).

### Walker 2020 {published data only}

- \*Walker N, Parag V, Verbiest M, Laking G, Laugesen M, Bullen C. Nicotine patches used in combination with e-cigarettes (with and without nicotine) for smoking cessation: a pragmatic, randomised trial. Lancet Respiratory Medicine 2020;8(1):54-64. [DOI: 10.1016/S2213-2600(19)30269-3]
- NCT02521662. A randomised-controlled clinical trial to evaluate the effectiveness and safety of combining nicotine patches with e-cigarettes (with and without nicotine) plus behavioural support, on smoking abstinence. clinicaltrials.gov/show/NCT02521662 (first received 9 August 2015).
- Walker N, Verbiest M, Kurdziel T, Laking G, Laugesen M, Parag V, et al. Effectiveness and safety of nicotine patches combined with e-cigarettes (with and without nicotine) for smoking cessation: study protocol for a randomised controlled trial. BMJ Open 2019;9(2):e023659.

### White 2022 {published and unpublished data}

- \*White CM, Tessier KM, Koopmeiners JS, Denlinger-Apte RL, Cobb CO, Lane T, et al. Preliminary evidence on cigarette nicotine reduction with concurrent access to an e-cigarette: manipulating cigarette nicotine content, e-liquid nicotine content, and e-liquid flavor availability. Preventive Medicine 2022;165(Pt B):107213. [DOI: 10.1016/j.ypmed.2022.107213]
- NCT03185546. Project 2 cigarette and e-cigarette nicotine content and e-liquid flavors. clinicaltrials.gov/ct2/show/NCT03185546 (first received 14 May 2021).
- White CM, Denlinger-Apte RL, Tessier KM, Koopmeiners JS, Hatsukami DK, Strasser AA. Cigarette nicotine reduction in the presence of an alternative: investigating how cigarette nicotine content and e-liquid content affect smoking. In: Society for Research on Nicotine and Tobacco (SRNT) 2021 Annual Meeting, February 24-27 2021 (virtual). 2021:2-3 (SYM2C).

### Xu 2023\* {published data only}

- Xu Y, Goldenson NI, Prakash S, Augustson EM, Shiffman S. Randomized trial assessing the effect of the JUUL system on switching away from cigarettes and smoking reduction among U.S. adults who smoke cigarettes. Experimental and Clinical Psychopharmacology 2024;32(1):3-15. [DOI: 10.1037/pha0000698]

### Yingst 2020 {published data only}

- Yingst J, Foulds J, Zurlo J, Steinberg MB, Eissenberg T, Du P. Acceptability of electronic nicotine delivery systems (ENDS) among HIV positive smokers. AIDS Care 2020;32(10):1224-8.
