# Supplementary material for: Electronic cigarettes for smoking cessation
Source: Cochrane Database Syst Rev. 2025 Nov 10;2025(11):CD010216. doi: 10.1002/14651858.CD010216.pub10 (PMC12599494; doi:10.1002/14651858.CD010216.pub10)
Supplement: Supplementary file 3 — Supplementary material 3 Characteristics of excluded studies [file CD010216-SUP-03-characteristicsOfExcludedStudies.html]

Characteristics of excluded studies


# Supplementary material 3 to: Electronic cigarettes for smoking cessation

Lindson N, Livingstone-Banks J, Butler AR, McRobbie H, Bullen CR, Hajek P, Wu AD, Begh R, Theodoulou A, Notley C, Rigotti NA, Turner T, Fanshawe T, Hartmann-Boyce J
  
https://doi.org/10.1002/14651858.CD010216.pub10

The material in this section has been supplied by the author(s) for publication under a Licence for Publication and the author(s) are solely responsible for the material. Cochrane has reviewed this material, but Cochrane has not copyedited, formatted or proofread. Cochrane accordingly gives no representations or warranties of any kind in relation to, and accepts no liability for any reliance on or use of, such material.

Back to top

# Characteristics of excluded studies

## Table of contents

- Studies ordered by Study ID
  - Adkison 2013
  - Al-Delaimy 2015
  - Bianco 2019
  - Caponnetto 2019
  - Chaumont 2019
  - ChiCTR2100044062
  - Collins 2019
  - Cox 2019a
  - Douptcheva 2013
  - Dyer 2023
  - Farsalinos 2013a
  - Grana 2014b
  - ISRCTN54776958
  - James 2016
  - Kasza 2013
  - Kimber 2023
  - Kotlyar 2022
  - Krysinski 2021
  - Manzoli 2015
  - Miura 2015
  - NCT03249428
  - NCT03575468
  - NCT03700112
  - NCT05658471
  - Nolan 2016
  - Palamidas 2014
  - Pearson 2012
  - Song 2020
  - St.Helen 2020
  - Tucker 2018
  - Wagener 2014
  - Walele 2016a
  - Walele 2016b
- Footnotes
- References to studies

## Studies ordered by Study ID

| Study | Reason for exclusion |
| --- | --- |
| Adkison 2013 | Although this study uses a prospective cohort design, no data on EC use were collected at baseline, with EC use data only being available at follow-up |
| Al-Delaimy 2015 | Observational study with no intervention provided - included in previous versions, but excluded from 2020 |
| Bianco 2019 | Ineligible intervention |
| Caponnetto 2019 | Ineligible intervention |
| Chaumont 2019 | Ineligible intervention |
| ChiCTR2100044062 | Wrong intervention |
| Collins 2019 | Ineligible intervention |
| Cox 2019a | Short-term abstinence only (< 6 months) |
| Douptcheva 2013 | Longitudinal study, but no data are reported for smoking cessation or reduction or for adverse events |
| Dyer 2023 | Wrong outcomes |
| Farsalinos 2013a | Included people that had already stopped smoking conventional cigarettes |
| Grana 2014b | Observational study with no EC intervention provided - included in previous versions, but excluded from 2020 |
| ISRCTN54776958 | Wrong outcomes |
| James 2016 | Follow-up at 12 weeks, AE data not collected |
| Kasza 2013 | Longitudinal study, but no data were reported for smoking cessation or for adverse events |
| Kimber 2023 | No valid short-term outcomes. Study 12 weeks (less than 6 months). |
| Kotlyar 2022 | Wrong intervention |
| Krysinski 2021 | Participants will be given a choice of a range of smoke-free products including EC and heat not burn. |
| Manzoli 2015 | Observational study with no EC intervention provided - included in previous versions, but excluded from 2020 |
| Miura 2015 | Tests a device that is not an EC |
| NCT03249428 | The study no longer includes e-cigarettes |
| NCT03575468 | Ineligible intervention |
| NCT03700112 | The study is < 7 days. The timeframe of 48 days for outcomes in the NCT record refers to the entire duration of the data collection. Timing confirmed by correspondence. |
| NCT05658471 | Wrong patient population |
| Nolan 2016 | Short-term abstinence only (< 6 months) |
| Palamidas 2014 | Short-term EC use only |
| Pearson 2012 | Longitudinal study, but no data were reported for smoking cessation or reduction or for adverse events |
| Song 2020 | Ineligible patient population |
| St.Helen 2020 | Ineligible intervention |
| Tucker 2018 | Short-term abstinence only (< 6 months) |
| Wagener 2014 | EC use for up to 1 week, but did not report on any adverse events |
| Walele 2016a | RCT but follow-up too short |
| Walele 2016b | RCT but follow-up too short |

## Footnotes

AE: adverse events  
 EC: electronic cigarette  
 RCT: randomized controlled trial

## References to studies

### Adkison 2013 {published data only}

- Adkison SE, O'Connor RJ, Bansal-Travers M, Hyland A, Borland R, Yong HH, et al. Electronic nicotine delivery systems: international tobacco control four-country survey. American Journal of Preventive Medicine 2013;44(3):207-15.

### Al-Delaimy 2015 {published data only}

- \*Al-Delaimy WK, Myers MG, Leas EC, Strong DR, Hofstetter CR. E-cigarette use in the past and quitting behavior in the future: a population-based study. American Journal of Public Health 2015;105(6):1213-9.
- Donzelli A. E-cigarettes may impair ability to quit, but other explanations are possible. American Journal of Public Health 2015;105(11):e1.

### Bianco 2019 {published data only}

- Bianco CL, Pratt SI, Ferron JC, Brunette MF. Electronic cigarette use during a randomized trial of interventions for smoking cessation among Medicaid beneficiaries with mental illness. Journal of Dual Diagnosis 2019;15(3):184-91.

### Caponnetto 2019 {published data only}

- Caponnetto P, Maglia M, Polosa R. Efficacy of smoking cessation with varenicline plus counselling for e-cigarettes users (VAREVAPE): a protocol for a randomized controlled trial. Contemporary Clinical Trials Communications 2019;15:100412.

### Chaumont 2019 {published data only}

- Chaumont M, El Channan M, Bernard A, Lesage A, Deprez G, Van Muylem A, et al. Short-term high wattage e-cigarette cessation improves cardiorespiratory outcomes in regular users: a randomized crossover trial. Journal of Hypertension 2019;Conference: 29th European Meeting on Hypertension and Cardiovascular Protection, ESH 2019. Italy. 37(Suppl 1):e8-9.

### ChiCTR2100044062 {published data only}

- ChiCTR2100044062. Study on the kinetics of nicotine metabolism of cigarettes/e-cigarettes in Chinese healthy subjects and the effect of complete/partial replacement of cigarettes with e-cigarettes on human biomarkers, tolerance and satisfaction survey of e-cigaret [传统卷烟/电子烟在中国健康受试者中尼古丁代谢动力学研究及用电子烟完全/部分替代香烟后对人体生物标志物的影响、对电子烟的耐受性及满意度调查]. Chinese Clinical Trial Registry 2021.

### Collins 2019 {published data only}

- Collins SE, Nelson LA, Stanton J, Mayberry N, Ubay T, Taylor EM, et al. Harm reduction treatment for smoking (HaRT-S): findings from a single-arm pilot study with smokers experiencing chronic homelessness. Substance Abuse 2019;40(2):229-39.

### Cox 2019a {published data only}

- Cox S, Dawkins L, Doshi J, Cameron J. Effects of e-cigarettes versus nicotine replacement therapy on short-term smoking abstinence when delivered at a community pharmacy. Addictive Behaviors Reports 2019;10:100202.

### Douptcheva 2013 {published data only}

- Douptcheva N, Gmel G, Studer J, Deline S, Etter JF. Use of electronic cigarettes among young Swiss men. Journal of Epidemiology and Community Health 2013;67(12):1075-6.

### Dyer 2023 {published data only}

- Dyer ML, Khouja JN, Jackson AR, Havill MA, Dockrell MJ, Munafo MR et al. Effects of electronic cigarette e-liquid flavouring on cigarette craving. Tobacco Control 2023;e1:e3-e9. [DOI: 10.1136/tobaccocontrol-2021-056769]

### Farsalinos 2013a {published data only}

- Farsalinos KE, Romagna G, Tsiapras D, Kyrzopoulos S, Voudris V. Evaluating nicotine levels selection and patterns of electronic cigarette use in a group of "vapers" who had achieved complete substitution of smoking. Substance Abuse: Research and Treatment 2013;7:139-46.

### Grana 2014b {published data only}

- Grana RA, Popova L, Ling PM. A longitudinal analysis of electronic cigarette use and smoking cessation. JAMA Internal Medicine 2014;174(5):812-3.

### ISRCTN54776958 {published data only}

- ISRCTN. E-cigarette support for smoking cessation: identifying the effectiveness of intervention components in an online randomised optimisation experiment. ISRCTN 54776958.

### James 2016 {published data only}

- \*James SA, Meier EM, Wagener TL, Smith KM, Neas BR, Beebe LA. E-Cigarettes for immediate smoking substitution in women diagnosed with cervical dysplasia and associated disorders. International Journal of Environmental Health Research 2016;13(3):E288. [DOI: 10.3390/ijerph13030288]
- NCT01989923. Smoking cessation in women with gynecological conditions. https://clinicaltrials.gov/ct2/show/NCT01989923 (accessed 9 September 2022).

### Kasza 2013 {published data only}

- Kasza KA, Bansal-Travers M, O'Connor RJ, Compton WM, Kettermann A, Borek N, et al. Cigarette smokers' use of unconventional tobacco products and associations with quitting activity: findings from the ITC-4 U.S. cohort. Nicotine & Tobacco Research 2013;16(6):672-81.

### Kimber 2023 {published data only}

- Kimber C, Sideropoulos V, Cox S, Frings D, Naughton F, Brown J et al. E-cigarette support for smoking cessation: identifying the effectiveness of intervention components in an on-line randomized optimization experiment. Addiction (Abingdon, England) 2023;118(11):2105-17. [DOI: 10.1111/add.16294]

### Kotlyar 2022 {published data only}

- Kotlyar M, Shanley R, Dufresne SR, Corcoran GA, Hatsukami DK. Effect on tobacco use and subjective measures of including e-cigarettes in a simulated ban of menthol in combustible cigarettes. Nicotine & Tobacco Research 2022;24(9):1448-57. [DOI: 10.1093/ntr/ntac107]

### Krysinski 2021 {published data only}

- \*Krysinski A, Russo C, John S, Belsey JD, Campagna D, Caponnetto P, et al. International randomised controlled trial evaluating metabolic syndrome in type 2 diabetic cigarette smokers following switching to combustion-free nicotine delivery systems: the DIASMOKE protocol. BMJ Open 2021;11(4):e045396. [DOI: 10.1136/bmjopen-2020-045396]
- Krysinski A, Russo C, Campagna D, Di Pino A, John S, Belsey J et al. A multicenter prospective randomized controlled trial investigating the effects of combustion-free nicotine alternatives on cardiovascular risk factors and metabolic parameters in individuals with type 2 diabetes who smoke: the DiaSmokeFree study protocol. Internal and Emergency Medicine 2024;19:321-32. [DOI: 10.1007/s11739-023-03467-6]
- NCT04231838. Metabolic syndrome in diabetic smokers using cigarettes & combustion-free nicotine delivery systems (DIASMOKE). clinicaltrials.gov/ct2/show/NCT04231838 (first received 18 January 2020).

### Manzoli 2015 {published data only}

- \*Manzoli L, Flacco ME, Fiore M, La Vecchia C, Marzuillo C, Gualano MR, et al. Electronic cigarettes efficacy and safety at 12 months: cohort study. PloS One 2015;10(6):e0129443.
- Manzoli L, La Vecchia C, Flacco ME, Capasso L, Simonetti V, Boccia S, et al. Multicentric cohort study on the long-term efficacy and safety of electronic cigarettes: study design and methodology. BMC Public Health 2013;13(1):883.
- NCT01785537. The efficacy and safety of electronic cigarettes: a 5-year follow-up study. clinicaltrials.gov/ct2/show/NCT01785537 (first received 7 February 2013).

### Miura 2015 {published data only}

- Miura N, Yuki D, Minami N, Kakehi A, Futama Y. A study to investigate changes in the levels of biomarkers of exposure to selected cigarette smoke constituents in Japanese adult male smokers who switched to a non-combustion inhaler type of tobacco product. Regulatory Toxicology and Pharmacology 2015;71(3):498-506.

### NCT03249428 {published data only}

- NCT03249428. E-cigarette inner city RCT [A community-based participatory action pragmatic randomized controlled trial using electronic-cigarette for tobacco dependence in the inner city population with a holistic approach]. clinicaltrials.gov/ct2/show/NCT03249428 (first received 15 August 2017).

### NCT03575468 {published data only}

- NCT03575468. Enhanced e-cigarette coaching intervention for dual users of cigarettes and e-cigarettes. https://clinicaltrials.gov/ct2/show/NCT03575468 (first received 2 July 2018).

### NCT03700112 {published data only}

- NCT03700112. Clinical study comparing 7 ENDS products and 1 combustible cigarette using 2 delivery methods. Clinicaltrials.gov/Ct2/Show/NCT03700112 (first Received 7 December 2018)..

### NCT05658471 {published data only}

- NCT05658471. Pharmacokinetics and pharmacodynamics of nicotine with use of standardized research electronic cigarette (SREC). Clinicaltrials.gov 2022;(first received 24 August 2023).

### Nolan 2016 {published data only}

- Nolan M, Leischow S, Croghan I, Kadimpati S, Hanson A, Schroeder D, et al. Feasibility of electronic nicotine delivery systems in surgical patients. Nicotine & Tobacco Research 2016;18(8):1757-62.

### Palamidas 2014 {published data only}

- Palamidas A, Gennimata SA, Kaltsakas G, Tsikrika S, Vakali S, Gratziou C, et al. Acute effect of an e-cigarette with and without nicotine on lung function. Tobacco Induced Diseases 2014;12(Suppl 1):A34.

### Pearson 2012 {published data only}

- Pearson JL, Richardson A, Niaura RS, Vallone DM, Abrams DB. E-cigarette awareness, use, and harm perceptions in US adults. American Journal of Public Health 2012;102(9):1758-66.

### Song 2020 {published data only}

- NCT02596685. Effects of electronic cigarette use on the lungs. https://www.clinicaltrials.gov/ct2/show/NCT02596685 (accessed 8 September 2022).
- Song M-A, Reisinger SA, Freudenheim JL, Brasky TM, Mathe EA, McElroy JP, et al. Effects of electronic cigarette constituents on the human lung: a pilot clinical trial. Cancer Prevention Research (Philadelphia, Pa.) 2020;13(2):145-52.

### St.Helen 2020 {published data only}

- St Helen G, Nardone N, Addo N, Dempsey D, Havel C, Jacob P, et al. Differences in nicotine intake and effects from electronic and combustible cigarettes among dual users. Addiction (Abingdon, England) 2020;115(4):757-67.

### Tucker 2018 {published data only}

- Tucker MR, Laugesen M, Bullen C, Grace RC. Predicting short-term uptake of electronic cigarettes: effects of nicotine, subjective effects, and simulated demand. Nicotine & Tobacco Research 2018;20(10):1265-71.

### Wagener 2014 {published data only}

- Wagener TL, Meier E, Hale JJ, Oliver ER, Warner ML, Driskill LM, et al. Pilot investigation of changes in readiness and confidence to quit smoking after e-cigarette experimentation and 1 week of use. Nicotine & Tobacco Research 2014;16(1):108-14.

### Walele 2016a {published data only}

- Walele T, Sharma G, Savioz R, Martin C, Williams J. A randomised, crossover study on an electronic vapour product, a nicotine inhalator and a conventional cigarette. Part A: Pharmacokinetics. Regulatory Toxicity 2016;74:187-92.

### Walele 2016b {published data only}

- Walele T, Sharma G, Savioz R, Martin C, Williams J. A randomised, crossover study on an electronic vapour product, a nicotine inhalator and a conventional cigarette. Part B: Safety and subjective effects. Regulatory Toxicity 2016;74:193-9.
