# Supplementary material for: Electronic cigarettes for smoking cessation
Source: Cochrane Database Syst Rev. 2025 Nov 10;2025(11):CD010216. doi: 10.1002/14651858.CD010216.pub10 (PMC12599494; doi:10.1002/14651858.CD010216.pub10)
Supplement: Supplementary file 4 — Supplementary material 4 Characteristics of studies awaiting classification [file CD010216-SUP-04-characteristicsOfAwaitingStudies.html]

Characteristics of studies awaiting classification


# Supplementary material 4 to: Electronic cigarettes for smoking cessation

Lindson N, Livingstone-Banks J, Butler AR, McRobbie H, Bullen CR, Hajek P, Wu AD, Begh R, Theodoulou A, Notley C, Rigotti NA, Turner T, Fanshawe T, Hartmann-Boyce J
  
https://doi.org/10.1002/14651858.CD010216.pub10

The material in this section has been supplied by the author(s) for publication under a Licence for Publication and the author(s) are solely responsible for the material. Cochrane has reviewed this material, but Cochrane has not copyedited, formatted or proofread. Cochrane accordingly gives no representations or warranties of any kind in relation to, and accepts no liability for any reliance on or use of, such material.

Back to top

# Characteristics of studies awaiting classification

## Table of contents

- Studies ordered by Study ID
  - UMIN000051684 2023
  - UMIN000051685 2023
- Footnotes
- References to studies

## Studies ordered by Study ID

UMIN000051684 2023

| Methods | RCT  Setting: dental surgery, West Java, Indonesia |
| Participants | Target sample 34  Inclusion criteria: 18 to 65 years, smoke CC  Exclusion criteria: acute illness that required treatment (subjects who had a viral infection) within 4 weeks before the enrolment visit, having oral soft tissue disease or any type of gingival overgrowth other than those caused by plaque, suffering from periodontitis, having a history of tooth loss due to periodontitis, being treated with anticoagulant therapy in the previous 6 months, using prostheses or orthodontic appliances, history of alcoholism or drug/chemical abuse, taking any drug or substance (other than tobacco) that interferes with the cyclooxygenase pathway (e.g. anti-inflammatory drugs including aspirin and ibuprofen) in the 14 days prior to the enrolment visit, taking antibiotics in the 14 days prior to the enrolment visit, breastfeeding or pregnant women. |
| Interventions | 2 arms  EC vs control (continued CC use) |
| Outcomes | Investigating effect of switching to vaping from tobacco cigarettes on gingival health |
| Notes | New to 2024  Contact: Indra Mustika, Universitas Padjadjaran, West Java Indonesia. Email: indra.mustika@unpad.ac.id |

UMIN000051685 2023

| Methods | RCT, 2-arm, non-blinded  Setting: dental surgery, West Java, Indonesia |
| Participants | Target sample 34  Inclusion criteria: 18 to 65, current CC use  Exclusion criteria: having an acute illness that required treatment (subjects who had a viral infection) within 4 weeks before the enrolment visit, having oral soft tissue disease or any type of gingival overgrowth other than those caused by plaque, suffering from periodontitis, having a history of tooth loss due to periodontitis, being treated with anticoagulant therapy in the previous 6 months, using prostheses or orthodontic appliances, history of alcoholism or drug/chemical abuse, taking any drug or substance (other than tobacco) that interferes with the cyclooxygenase pathway (e.g. anti-inflammatory drugs including aspirin and ibuprofen) in the 14 days prior to the enrolment visit, taking antibiotics in the 14 days prior to the enrolment visit, breastfeeding or pregnant women |
| Interventions | Intervention: EC  Control: continued CC use |
| Outcomes | Impact of switching from tobacco smoking to vape use on the number of buccal and palatal mucosal micronucleus cells |
| Notes | New to 2024  Contact: Indra Mustika, Universitas Padjadjaran, West Java Indonesia. Email: indra.mustika@unpad.ac.id |

## Footnotes

CC: combustible cigarette  
 EC: electronic cigarette  
 RCT: randomized controlled trial

## References to studies

### UMIN000051684 2023 {published data only}

- Taoc and vaping. From record: effect of switching to vaping from tobacco cigarettes on gingival health. UMIN000051684.

### UMIN000051685 2023 {published data only}

- MNC From record: Evaluating the impact of switching from tobacco smoking to vape use on the number of buccal and palatal mucosal micronucleus cells. UMIN000051685 2023.
