# Supplementary material for: Electronic cigarettes for smoking cessation
Source: Cochrane Database Syst Rev. 2025 Nov 10;2025(11):CD010216. doi: 10.1002/14651858.CD010216.pub10 (PMC12599494; doi:10.1002/14651858.CD010216.pub10)
Supplement: Supplementary file 5 — Supplementary material 5 Characteristics of ongoing studies [file CD010216-SUP-05-characteristicsOfOngoingStudies.html]

Characteristics of ongoing studies


# Supplementary material 5 to: Electronic cigarettes for smoking cessation

Lindson N, Livingstone-Banks J, Butler AR, McRobbie H, Bullen CR, Hajek P, Wu AD, Begh R, Theodoulou A, Notley C, Rigotti NA, Turner T, Fanshawe T, Hartmann-Boyce J
  
https://doi.org/10.1002/14651858.CD010216.pub10

The material in this section has been supplied by the author(s) for publication under a Licence for Publication and the author(s) are solely responsible for the material. Cochrane has reviewed this material, but Cochrane has not copyedited, formatted or proofread. Cochrane accordingly gives no representations or warranties of any kind in relation to, and accepts no liability for any reliance on or use of, such material.

Back to top

# Characteristics of ongoing studies

## Table of contents

- Studies ordered by Study ID
  - ACTRN12619001787178
  - ACTRN12621000148875
  - ACTRN12625000179437
  - Berlin 2019
  - Cox 2022
  - El-Khoury 2021
  - Hameed 2024
  - Holliday 2022
  - Howard 2022
  - ISRCTN14068059
  - ISRCTN61193406
  - ISRCTN82413824
  - Lin 2024
  - Malik 2023
  - Murray 2020
  - NCT01842828
  - NCT02398487
  - NCT02590393
  - NCT03277495
  - NCT03625986
  - NCT03862924
  - NCT03962660
  - NCT04003805
  - NCT04058717
  - NCT04063267
  - NCT04218708
  - NCT04238832
  - NCT04452175
  - NCT04521647
  - NCT04649645
  - NCT04708106
  - NCT04709471
  - NCT04725656
  - NCT04946825
  - NCT05023096
  - NCT05144542
  - NCT05199480
  - NCT05205811
  - NCT05206435
  - NCT05257629
  - NCT05278065
  - NCT05510154
  - NCT05555069
  - NCT05610514
  - NCT05703672
  - NCT05815199
  - NCT05825924
  - NCT05881304
  - NCT05887947
  - NCT05960305
  - NCT06063421
  - NCT06077240
  - NCT06111053
  - NCT06118502
  - NCT06169813
  - NCT06260683
  - NCT06264154
  - NCT06372899
  - NCT06373679
  - NCT06534905
  - NCT06543407
  - NCT06554873
  - NCT06614504
  - Polosa 2024
  - Schiek 2024
  - Walker 2023
- Footnotes
- References to studies

## Studies ordered by Study ID

ACTRN12619001787178

| Study name | Project NEAT: NicotinE As Treatment for tobacco smoking following discharge from residential withdrawal services |
| Methods | RCT  Project NEAT: A randomized controlled trial to examine the efficacy of vaporised nicotine products and telephone quitline support compared with nicotine replacement therapy and telephone quitline support when used following discharge from residential withdrawal services  Setting: Australia (New South Wales, Queensland, Victoria)  Recruitment 4 hospital sites: Belmont Hospital, Belmont; St Vincent's Hospital, Darlinghurst; Turning Point Drug and Alcohol Centre, Richmond; Royal Brisbane & Womens Hospital, Herston |
| Participants | Target sample size: 926  Inclusion criteria:   - Aged 18 or over - Daily tobacco smoker (10 or more cigarettes) on entering withdrawal unit - Accessing treatment from participating services - Want to quit smoking in the next 30 days - Has capacity to consent and able to understand the participant materials and follow the study instructions and procedure (e.g. sufficient English language ability and not too unwell as judged by medical staff)   Exclusion criteria:   - Pregnant or breastfeeding - Enrolled in another study - Scheduled to be transferred to a long-term residential rehabilitation service following discharge from the withdrawal unit - Used VNP (containing nicotine) in the last 30 days - Currently engaged in Quitline’s call-back services - No ready access to a phone |
| Interventions | Condition 1: vaporised nicotine products and Quitline  Condition 2: current best practice treatment for tobacco smoking (combination nicotine replacement therapy and Quitline) |
| Outcomes | 9 months after inpatient withdrawal unit discharge:   - Self-reported 7 months continuous abstinence from tobacco smoking - Biochemically verified 7-month continuous abstinence from tobacco smoking   3 and 9 months after inpatient withdrawal unit discharge:   - 30-day point prevalence abstinence - 7-day point prevalence abstinence - Abstinence from all nicotine/tobacco products |
| Starting date | Anticipated enrolment: 19 December 2019. Anticipated date last data collection: 19 September 2022 |
| Contact information | Prof Billie Bonevski, Billie.Bonevski@newcastle.edu.au |
| Notes | Funding: National Health and Medical Research Council (grant number: G1800272), Canberra ACT 2601 |

ACTRN12621000148875

| Study name | HARMONY: HARM reduction for Opiates, Nicotine and You |
| Methods | Design: randomized, single-blinded, parallel-group trial |
| Participants | Inclusion criteria: written, informed consent; 18 to 65 years; accessing opioid agonist treatment from a participating service; current daily tobacco smokers on self-report; want to quit or cut down their tobacco smoking; willing and able to comply with requirements of study (including having access to a phone)  Exclusion criteria: breastfeeding or pregnant; severe medical disorder assessed by study medical officer (such as, but not limited to, unstable cardiovascular/peripheral vascular disease, poorly controlled hypertension); severe and unstable psychiatric disorder assessed by study medical officer (such as, but not limited to, acute psychosis, severe anxiety and/or mood disorder, intent to harm self or others); current enrolment in a clinical trial involving any investigational drug; gular use (more than one day per week) of VNP or EC containing nicotine in the last 30 days; not available for FU  Motivated to quit |
| Interventions | Comparison of a 12-week course of liquid nicotine delivered via EC or Vaporised Nicotine Product (VNP) to best practice Nicotine Replacement Therapy (NRT)  Condition 1: EC (VNP) (Innokin Endura T18‐II starter kit) • Device loaded with one bottle of 12 mg/mL e-liquid • An additional seven (7) bottles of 12 mg/mL e-liquid • A brief information session on how to use the VNP • A VNP information pack including safe storage of e-liquid nicotine and disposal • 1-week supply of nicotine patches • Training on the use of NRT patches • Where possible, ensure that participant uses the VNP before leaving and leaves wearing an NRT patch. Adherence will be measured via questionnaires. In addition to the VNP, liquid nicotine and NRT patches, participants will be shown the New Zealand website vapingfacts.health.nz/ and encouraged to visit the site as an online resource throughout the trial. Participants will also receive training in the forms of brief videos, information pamphlets, user manuals and interactive discussions with research staff. |
| Outcomes | Baseline, 12, 24 weeks  Primary outcome: self-reported 7-day PPA from tobacco smoking assessed in the following dichotomous question “In the last 7 days, have you smoked a cigarette, even a puff?" (Week 12)  Secondary outcome: a cost consequence study setting out detailed comparative costs of treatments - from the perspective of healthcare provider and primary and secondary outcomes of the VNP and NRT  Adverse events recorded  Biochemically verified PPA: this will be measured via a CO monitor breath test for participants who self-report 7-day PPA at end of treatment (Week 12)  Changes in nicotine craving and withdrawal symptoms |
| Starting date | Registered Feb 2021. Record updated Jan 2024. |
| Contact information | Adrian Dunlop, adrian.dunlop@health.nsw.gov.au |

ACTRN12625000179437

| Study name | Puff vs Pill: break the Habit Study: effect of Nicotine Vaping Products vs Varenicline on Smoking Cessation Among People Experiencing Social Disadvantage |
| Methods | RCT |
| Participants | Target N = 872  Inclusion: Aged 18 years or older; Receiving a government pension, support, or allowance (proxy for low-SES)\*; Person who currently smokes tobacco daily\*\* and wanting to quit tobacco smoking; Willing to use varenicline or NVPs in next quit attempt; Willing to make a quit attempt on designated quit day (~8-14 days post-randomisation);  Exclusion: Pregnant / breast-feeding; participation in another quit smoking program or study, or previously enrolled in this study; Current use of any quit smoking medications or products (i.e., NRT, bupropion [Zyban], varenicline [Varenapix], cytisine, NVPs/ e-cigarettes containing nicotine, nicotine inhalers or any other quit smoking medications or products); Allergies or hypersensitivity to either varenicline or nicotine-containing e-liquids, or any excipients; End stage or severe renal diseased; Deemed medically unfit.  Population: low SES  EC use at baseline: no  Willing to quit CC: yes |
| Interventions | Arm 1. EC / Nicotine vaping products (NVPs) in the form of two “pod” devices either prefilled [Alt] and refillable [Rift] with nicotine salt e-liquids containing nicotine (4%;40mg/ml) in tobacco or mint flavours for 12 weeks. For each device a USB charger will be provided, as well as a shared wall adaptor. Participants will be provided with detailed instructions on how to use the vaping products, along with a wallet card and fridge magnet with product use and safety information for quick access. Participants are advised to use the study product ad libitum throughout the day to either mitigate or satiate the urge to smoke tobacco products. They are encouraged to stop using the devices once they no longer feel the urge to smoke.    The alt. device has replaceable pre-filled 2ml pods that contain 40mg/ml nicotine salt e-liquid in either tobacco or mint flavour. The Rift device has a refillable 2ml pod, and is supplied alongside 40mg/ml nicotine salt e-liquid (provided in 30ml bottles) in tobacco and mint flavours.  Arm 2. Varenicline |
| Outcomes | Baseline, 12 months  Carbon monoxide (CO) verified 6-month continuous abstinence at 12-month follow-up. [Only participants self-reporting continuous abstinence from smoking tobacco at final follow-up will be biochemically verified]. Bedfont iCO Smokerlyzer®, or administered by a trained researcher using a hand-held Micro+™ Smokerlyzer® with a disposable, one-use mouthpiece. CO level of less-than-or-equal-to 5 parts per million will be considered abstinent.  Change in number of cigarettes smoked from baseline to final follow up at 12 months  Self-reported AEs and SAEs.  Use of varenicline or NVP at final follow up  Change in self-reported respiratory symptoms.[ Modified Medical Research Council (MRC) dyspnoea scale  Participants’ treatment adherence and compliance.  Self-reported: 7-day point prevalence abstinence; and continuous abstinence for 6 months  Change in self-reported respiratory symptoms |
| Starting date | Registered 14 February 2025. |
| Contact information | Ryan Courtney, National Drug and Alcohol Research Centre, The University of New South Wales, Sydney NSW 2052 Australia. r.courtney@unsw.edu.au |
| Notes | Added as ongoing study 2025. |

Berlin 2019

| Study name | Randomized, placebo-controlled, double-blind, double-dummy, multicentre trial comparing electronic cigarettes with nicotine to varenicline and to electronic cigarettes without nicotine: the ECSMOKE trial protocol |
| Methods | 3-arm, randomized, placebo-controlled, multicentre, double-blind, double-dummy, parallel-group phase III type trial  Setting: smoking cessation clinics of both academic and community hospitals  Recruitment is either local (a) directly by the centres or centralized (b) using a web page and a centralized study-specific phone number and email address.   - People who smoke, intending to quit smoking, are recruited by advertisement in pharmacies, physicians’ offices situated in the catchment area of each investigator’s centre, by local newspapers and in public places of the centres’ healthcare facilities. - Candidates to participate can register by the study’s website, unique email address, and phone number. Registration is followed by a phone screening before dispatching to the study centres. Only 1 person by household will be recruited. |
| Participants | Estimated enrolment: 650 participants  Inclusion criteria: people who smoke, ≥ 10 CPD (factory-made or roll-your-own) in the past year; aged 18 to 70; motivated to quit, defined as a score > 5 on a visual rating scale ranging from 0 (not motivated at all) to 10 (extremely motivated); informed consent; understanding and speaking French; women of childbearing age can be included if they use an effective contraceptive method: either hormonal contraception or an intrauterine device started at least 1 month before the first research visit; individual affiliated to a health insurance system; previous failure of NRT for smoking cessation  Exclusion criteria: any unstable disease condition within the last 3 months defined by the investigator as major change in symptoms or treatments, such as recent myocardial infarction, unstable or worsening angina, severe cardiac arrhythmia, unstable or uncontrolled arterial hypertension, recent stroke, cerebrovascular disease, obliterative peripheral arterial disease, cardiac insufficiency, diabetes, hyperthyroidism, pheochromocytoma, severe hepatic insufficiency, history of seizures, severe depression, COPD; any life-threatening condition with life expectancy of < 3 months; alcohol use disorder defined as a score ≥ 10 on the Alcohol Use Disorders Identification Test (AUDIT)-C questionnaire; abuse of or dependence on illegal drugs in the last 6 months, revealed by medical history; regular use of tobacco products other than cigarettes; current or previous (last 6 months) use of EC; pregnancy/breastfeeding; protected adults; current or past 3 months participation in another interventional research; current or past 3 months use of smoking cessation medication such as varenicline, bupropion, NRTs; known lactose intolerance (placebo tablets contain lactose); hypersensitivity to the active substance or to any of the excipients; known severe renal failure |
| Interventions | A) **EC without nicotine** (ECwoN) plus placebo tablets of varenicline (0.50 mg) administered by oral route: placebo condition  B) **EC with nicotine** (ECwN) plus placebo tablets of varenicline: ECwN condition.  C) Reference: ECwoN plus 0.5 mg varenicline tablets: **varenicline condition**. Varenicline administered according to the marketing authorization  E-cigarette details:   - EC device Mini iStick kit (20 W) Eleaf, clearomzser: GS Air M with resistance of 1.5 ohm. To keep the blinding, the clearomizer’s Pyrex window is of grey colour not allowing to distinguish the colouration of the e-liquid containing nicotine. Liquid for EC is manufactured by GAIATREND SARL (www.gaiatrend.fr/fr/). - All participants will be delivered a short manual and a video specifically developed for this study explaining the use of EC. At each visit, participants receive verbal counselling about the use of the EC device and answers to their questions about handling the EC device.   Behavioural support:   - Brief behavioural smoking cessation counselling for all participants is administered at all visits by the investigators specialized in smoking cessation. It is based on the national guidelines for smoking cessation.   Treatment duration: 1 week + 3 months |
| Outcomes | Week 2, 4, 8, 10, 12, 24 after target quit day  Primary outcome:   - Continuous smoking abstinence rate (CAR) (abstinence from conventional/combustible cigarettes) during the last 4 weeks (weeks 9 to 12) of the treatment period of 3 months   Secondary outcomes:   - Safety profile - PPA rate - CAR confirmed by urinary anabasine concentration - Changes in cpd consumption - Craving for tobacco and withdrawal symptoms with respect to baseline |
| Starting date | 17 October 2018. Trial suspended (March 2025) due to unavailabilty of varenicline. Due to re-start as soon as varenicline is available. |
| Contact information | Ivan Berlin, ivan.berlin@aphp.fr |

Cox 2022

| Study name | E-cigarettes vs usual care for smoking cessation when offered at homeless centres (SCeTCH) |
| Methods | RCT. A multicentre, cluster-randomized controlled trial  Setting: 32 centres across 6 areas in Great Britain: Scotland; Wales; London; South-East England; South-West England and East England |
| Participants | Enrolment: 477.  Inclusion criteria: currently accessing homeless centre services and actively engaging with the service; > 18 years; self-reported daily smoking, then biochemically verified  Exclusion criteria: never- and ex-smokers; currently using a smoking cessation aid; unable to provide written consent; not known to centre staff; allergic to any of the e-liquid ingredients (EC arm only); pregnant |
| Interventions | EC: refillable  Cluster rather than individual randomization will be used.  Arm 1: Planned intervention  Delivery of the EC intervention will be as per our feasibility study. Centre staff will provide EC arm participants with a tank-style refillable EC starter kit (e.g. the PockeX as used in our feasibility study or similar model determined via our PPI work), a choice of nicotine strength e-liquids (12 mg/mL and 18 mg/mL) and flavours (tobacco, menthol or fruit) and an EC fact-sheet. E-liquids (5 x 10 mL bottles per week) will be supplied for 4 weeks at weekly intervals by centre staff.     Arm 2: Control/comparator group  Control arm will be usual care (UC). This will include very brief advice (VBA) to quit (in the form of an ‘NHS choices’ leaflet adapted for this population as used in our feasibility study) and signposting to the local SSS.    All participants (intervention and control) will be offered a GBP 15 Love2Shop gift card (which cannot be used for tobacco or alcohol purchases) for each follow-up appointment attended. |
| Outcomes | Baseline, 4, 12, and 24 weeks  Primary outcome measure  Current primary outcome measure as of 27/07/2022:  Sustained CO-validated smoking cessation at 24 weeks using the Russell Standard for cessation trials and intention-to-treat analysis (i.e. no more than 5 cigarettes since 2 weeks from baseline, validated by expired CO < 8 ppm  Secondary outcome measures  Current secondary outcome measure as of 27/07/2022:   1. Smoking reduction at 24 weeks 2. 7-day point prevalence quit rates at 4, 12, and 24 weeks self-reported and validated by expired CO < 8 ppm 3. Changes in the frequency of risky smoking practices (e.g. sharing cigarettes, smoking discarded cigarettes) 4. Cost-effectiveness of the intervention measured using a service use questionnaire and the EQ-5D-5L (4, 12, 24 weeks) 5. Fidelity of intervention implementation 6. Mechanisms of change measured quantitatively via questions (e.g. attitudes and perceptions of e-cigs) 7. Contextual influences and sustainability telephone interviews |
| Starting date | Start date 23 April 2021. Estimated study end date: 31.01.2025. |
| Contact information | Lynne Dawkins, dawkinl3@lsbu.ac.uk |
| Notes | New to 2022 update |

El-Khoury 2021

| Study name | Preference-based tools for smoking cessation among disadvantaged smokers, a pragmatic randomised controlled trial (STOP) |
| Methods | RCT  France |
| Participants | Actual enrollment: 167. Estimated enrolment: 528.  Inclusion criteria: daily smokers (≥ 5 cigarettes/day); low socioeconomic position; available for at least 4 appointments over a 6-month period; affiliation to or benefiting from social security or state medical support  Exclusion criteria: individuals who do not speak French; major citizens protected by law, adults unable to express their consent; pregnant women; regular smokers who vape daily (at least once a day) |
| Interventions | EC: type not stated  Arm 1: The STOP intervention  Assisting smokers with low socioeconomic position in their smoking cessation attempt. Routine care and adapted advice supplemented with a free delivery of any or several type(s) of nicotine replacement therapy (NRT) (patches, inhalers, gum, tablets, etc.) and/or an e-cigarette + e-liquid, based on the smokers' preference and choice  Arm 2: Standard care  Participants randomised to the standard care group will be given standard care in assisting their smoking cessation attempt, but without free delivery of NRT or e-cigarettes.  Standard care includes motivational interviewing, advice to quit, and prescription for NRTs. |
| Outcomes | Smoking abstinence at 6 months after inclusion  Total number of days of abstinence at 6 months  Smoking abstinence at 1 and 3 months after inclusion  Number of relapses; CPD; proportion of participants who have significantly reduced daily smoking |
| Starting date | Start date: 26 February 2021. Study completion date: 30 August 2024 (final data collection date for primary outcome measure). |
| Contact information | Fabienne El-Khoury, Institut National de la Santé Et de la Recherche Médicale, France |
| Notes | New to 2022 update |

Hameed 2024

| Study name | Clinical study protocol on electronic cigarettes and nicotine pouches for smoking cessation in Pakistan: a randomized controlled trial |
| Methods | RCT  Setting: Pakistan  Recruitment centres in 2 metropolitan districts: Islamabad and Rawalpindi |
| Participants | Estimated enrolment: 600  EC + counselling: estimated 200  Nicotine pouches + counselling: estimated 200  Counselling: estimated 200  Inclusion criteria: > 18; > 10 CPD; CC use for > 1 year; willing to stop CC use; sign a written consent form; 1 applicant per household; phone  Exclusion criteria: pregnant; childbearing mothers; using other nicotine- and non-nicotine-based cessation therapies; chest pain, or another cardiovascular event or procedure (e.g. heart attack, stroke, insertion of stent, bypass surgery)  Motivated to quit |
| Interventions | Provision of EC or nicotine pouches for 48 weeks  3 groups:   1. EC and liquid + 4 basic counselling sessions every 12 weeks over 48 weeks 2. Nicotine pouches + 4 basic counselling sessions every 12 weeks over 48 weeks 3. Counselling only: 4 basic counselling sessions every 12 weeks over 48 weeks |
| Outcomes | Baseline, 12, 24, 36, 48 weeks. 60 weeks FU.  AEs  CPD  Self-reported point-prevalence abstinence from CC in the previous week with biochemical validation will be used (exhaled carbon monoxide less than 10 parts per million (PPM))  7-day point-prevalence abstinence from CC (at all subsequent check-ups) (biochemically validated at weeks 12, 24, 36, 48, and 60)  Harm-reduction effect of e-cigarettes and nicotine pouches |
| Starting date | Estimated starting date: December 2023  Estimated completion date: April 2025 |
| Contact information | Abdul Hameed, +923315813713, hameedleghari@gmail.com  Daud Malik, +923028560310, daud31us@yahoo.com |
| Notes | New to 2024  Funding: Foundation for a Smoke Free World INC. Funded by Philip Morris International.  ClinicalTrials.gov NCT05715164 |

Holliday 2022

| Study name | ENHANCE-D trial: Enhancing dental health advice |
| Methods | Design: RCT  A pragmatic, multicentre, definitive, open-label, 3-arm, parallel-group, individually randomized, controlled, superiority trial, comparing the clinical- and cost-effectiveness, and safety of enhanced smoking cessation interventions to usual care, and each other (with an internal pilot)  Setting: 56 NHS primary dental care setting in England and Scotland. Newcastle Clinical Trials Unit, UK |
| Participants | Estimated enrolment: 1460 participants, 455 periodontitis subgroup  Adult regular tobacco smokers attending an NHS dental setting. Dental patients with or without gum disease  Inclusion criteria: a basic periodontal examination completed within the last 3 months; ≥ 18 years; current smoker  Periodontitis subgroup: minimum of 16 natural teeth; diagnosis of periodontitis stage II (or greater)  Exclusion criteria: pregnant or currently breastfeeding; enrolled in another interventional research trial; used quit-smoking aid or reduce/quit alcohol; phaeocromocytoma, uncontrolled hyperthyroidism, extensive dermatitis/skin disorder; hypersensitivity to nicotine or any component of the study products; taking: clozapine, olanzapine, theophylline or aminophylline |
| Interventions | EC: EC starter kit. Stainless Steel Aspire PockeX e-cigarette, coil replacement pack, 3-pin plug, 10 x Halo standard 10ml e-liquids (4 flavour options available)  Condition: smoking cessation in dental patients with or without gum disease  Arm 1: E-cigarette (EC) starter kit with single-visit behavioural support (same behavioural intervention as the NRT group). Participants will be expected to source their own supply of e-liquid after the initial supply and advice will be given as to where to source suitable MHRA registered products. Duration will vary depending on use of EC.  Arm 2. Nicotine Replacement Therapy (NRT): standard 12-week course of combination NRT with single-visit behavioural support including the offer of NRT. 12-week course of combination NRT (patch plus faster-acting form such as chewing gum or lozenge), in line with current recommendations. Duration will be 12 weeks if a participant wants to continue NRT after initial 4-week supply. Nicotine transdermal patches. Option 1: NiQuitin 7 mg, 14 mg, 21 mg (24-hour patch). Option 2: Nicorette invisi 10 mg, 15 mg, 25 mg (16-hour patch) Nicorette gum 2 mg, 4 mg. Nicorette lozenge 2 mg, 4 mg  Arm 3. VBA: usual care (control) 1. VBA is usual care for smokers in dental settings usually following the 3As: Ask, Advise, Act technique. This will signpost participants to a GP, pharmacy or stop-smoking service (SSS). 2. Participants in the control group will be free to use NRT or ECs as they wish, but these will not be provided by the dental professional. 3. Conducted at baseline visit, only a 5-minute intervention  All patients will be followed up for up to 12 months from baseline. |
| Outcomes | 6 months for periodontal health parameters and 12 months for smoking outcomes  To compare smoking abstinence at 6 months of NRT and EC to usual care and to each other (all participants). Biochemically verified smoking abstinence at 6 months, carbon monoxide monitor.  To compare the periodontal health at 6 months of NRT and EC interventions to usual care and to each other, for those with periodontitis at baseline. Percentage of periodontal sites at 6 months with PPD (Pocket Probing Depths) ≥ 5 mm  AEs; oral health, and oral health QoL; to evaluate nicotine dependence, urges to smoke, withdrawal symptoms, and longer-term smoking abstinence (12 months); cost benefit. SES inequalities  Expired air carbon monoxide (eCO). Continuous biochemically verified smoking abstinence at 12 months. Fagerstrom Test for Nicotine Dependence (FTND). Cigarette withdrawal symptoms are measured using Mood and Physical Symptoms Scale (MPSS). Oral Health Quality of Life Assessment (OHQoL-UK). Oral health is measured using number of teeth at baseline and 6 months. Health economic evaluation. |
| Starting date | Protocol 2022  Study start date February 2022. Estimated completion date March 2025. |
| Contact information | Dr Richard Holliday richard.holliday@newcastle.ac.uk  Professor Elaine McColl, Newcastle University |
| Notes | New to 2023 update |

Howard 2022

| Study name | Vaporized nicotine products (VNP) versus nicotine replacement therapy for tobacco smoking cessation among low-socioeconomic status smokers: a randomised controlled trial |
| Methods | Design: RCT  Recruitment: study advertisements across online and social media platforms such as Facebook advertisements. Participants in a recently completed clinical trial comparing cytisine versus varenicline for smoking cessation who consented to being contacted about future research and were receiving a government pension were also invited to take part.  Setting: National Drug and Alcohol Research Centre at the University of New South Wales, Sydney, Australia |
| Participants | Target N: 1058  Inclusion criteria: participants can be included if they meet the following criteria:   - Willing to allow the research team and study clinician to access their data for quality assurance and to maintain the integrity of the trial - 18 years of age or older - Receiving a government pension or allowance (proxy for low SES) - Are a current daily smoker - Interested in quitting smoking and using the study products and willing to make a quit attempt in the next 2 weeks - Have a mobile phone that can receive text messages - Agree to use the allocated study product and refrain from using another quit-smoking medication whilst using the study products - Willing to receive daily quit-support text messages during the treatment period (with the option to opt out during the study) |
| Interventions | Vaporized nicotine product (VNP) devices (1 tank device and 1 pod device) for 8 weeks plus 5-week Text Message behavioural quit Support (TMS) with the option to opt out at any stage if desired. Participants will receive a mix of quit-smoking support text messages with content including information on how to use the study products; coping with nicotine withdrawal symptoms; study progress updates; and motivational ‘feel good’ messages. A mix of text, emojis and links to resources such as videos, websites and Graphics Interchange Format (GIF) images, will be used throughout the TMS programme to promote engagement with the programme. Each device will be charged using the provided USB charger and wall adaptor. A replacement battery and replacement coils (5 pieces per pack) will also be provided. The VNP tank device used is the Innokin Endura T18 Personal Vaporizer, which has a refillable 2.5 mL tank for the e‐liquid (18 mg/mL nicotine). 3 e-liquid flavours will be provided: tobacco, menthol and a fruit flavour. The study will have 3 e-liquid suppliers to guarantee ongoing supply throughout the study: Lumo Liquid in 10 mL bottles; VAPO e-liquid in 30 mL bottles; and DashVapes e-liquid in 30 mL bottles. All e-liquids are 18 mg/mL in strength. Lumo Liquid ingredients are as follows (w/w): tobacco flavouring (1.19%), nicotine (1.60%), vegetable glycerine (24.56%), propylene glycol (73.24%); menthol flavouring (4.83%), nicotine (1.60%), vegetable glycerine (22.99%), propylene glycol (71.18%); strawberry flavouring (0.63%), nicotine (1.60%), vegetable glycerine (33.00%), propylene glycol (71.00%). VAPO e-liquid additional flavour ingredients are as follows (w/w): tobacco flavouring (25.88%), nicotine (17.25%), vegetable glycerine (36.53%), propylene glycol (20.34). |
| Outcomes | Primary outcome:  CO-verified 6-month continuous abstinence at 7-month follow-up. Continuous 6-month abstinence will be defined as having remained quit for 6 months (having smoked no more than 5 cigarettes in that time), and a CO level of ≤ 5 ppm. Depending on the participant's indicated preference, the CO breath test will be self-administered using a hand-held iCO™ Smokerlyzer® (using provided instructions), or administered by a trained researcher using a hand-held iCO™ Micro+™ Smokerlyzer® with a disposable, one-use mouthpiece. Both devices are non‐invasive and require the participant to blow air into the device for 15 seconds to measure their CO level. An exhaled CO level of ≤ 5 ppm will be considered abstinent.  The final follow-up interview will occur 7 months after the baseline interview completion date.  Secondary outcome:  Change in financial stress (assessed using Index of Financial Stress) |
| Starting date | Start date: 30 March 2021. Date last data collection 8 Dec 2022. |
| Contact information | Dr Ryan Courtney, National Drug and Alcohol Research Centre The University of New South Wales Sydney NSW 2052 Australia, r.courtney@unsw.edu.au |

ISRCTN14068059

| Study name | E-cigarettes for smoking cessation and reduction in people with a mental illness, ESCAPE |
| Methods | RCT, multicentre  Setting: GP practice, hospital, England, UK  Organisation: University of York  Tees, Esk and Wear Valleys NHS Foundation Trust; Bradford District Care NHS Foundation Trust; Sheffield Clinical Commissioning Group Hq; Oxford NHS Foundation Trust; Greater Manchester Mental Health NHS Foundation Trust; South West Yorkshire Partnership NHS Foundation Trust; Nottinghamshire Healthcare NHS Foundation Trust; Lancashire and South Cumbria NHS Foundation Trust; Norfolk and Suffolk NHS Foundation Trust; CRN North East and North Cumbria; CRN East of England; The Burns Practice Bennetthorpe; Conisborough Medical Practice Conisbrough, Doncaster; Woodstock Bower Surgery, Rotherham |
| Participants | Target N = 616  Mental Health Trusts and GP practices mainly in Yorkshire (UK)  Inclusion: adults (aged over 18 years) receiving treatment for a mental illness in primary or secondary care, who smoke regularly, willing to quit or reduce cigarette smoking  Exclusion: inpatient admission in the last 3 months; currently using EC regularly (at least weekly); participating in other smoking cessation trials; receiving treatment for drug or alcohol use; Alzheimer’s disease or dementia; pregnant or breastfeeding |
| Interventions | Group 1 EC: e-cigarette and e-liquid to use for 4 weeks in addition to the usual care they are receiving. EC starter kit, a 20 mg/mL strength DOTPRO e-cigarette starter kit (https://www.liberty-flights.co.uk/DOT-PRO/DOT-PRO-Vape-Kit/) will be offered in a choice of flavours. The starter kit containing a pod-based e-cigarette, a 4-week supply of refill pods and an information leaflet. Brief face-to-face consultation with a clinician, who will explain how to use the e-cigarette and provide information to enable participants to make positive changes to their smoking behaviour. Encouraged to set a quit date. Participants will be provided with an e-liquid supply for 4 weeks.  Group 2, usual care: participants will receive care as usual but will receive an e-cigarette and some e-liquid at the end of the study at the 6-month follow-up. |
| Outcomes | Baseline, 1, 6 months  Questionnaire; CO monitor  Primary outcome measure   1. Self-reported 7-day point prevalence abstinence measured using a questionnaire (question written in-house) at 6 months 2. Co-verified quit (main outcome) measured using a CO monitor (smokylizer) at 6 months   Secondary outcome measures   1. General smoking-related characteristics, abstinence, quit attempts and methods (including e-cigarettes), measured using a questionnaire (questions written in-house) at baseline, 1 month and 6 months 2. Nicotine dependence measured using the Fagerstrom Test for Nicotine Dependence at baseline, 1 month and 6 months 3. Strength of urges to smoke measured using the SUTS questionnaire at baseline, 1 month and 6 months 4. Motivation to quit measured using the Motivation To Stop Scale (MTSS) at baseline, 1 month and 6 months 5. Mental wellbeing measured using PHQ-9 and GAD-7 at baseline and 6 months 6. Alcohol use measured using AUDIT-C at baseline and 6 months 7. Health-related quality of life measured using EQ-5D-5L at baseline and 6 months 8. Attrition measured using a questionnaire (questions written in-house) at 1 month and 6 months 9. Adherence rate measured using a questionnaire (questions written in-house) at 1-month follow-up 10. Cost-effectiveness measured using a questionnaire (questions written in-house) at baseline, 1 month and 6 months 11. Adverse events measured using a questionnaire (questions written in-house) at 1 month and 6 months |
| Starting date | Study start date: March 2023  Estimated study end date: April 2025 |
| Contact information | Dr Anna-Marie Marshall, a.marshall@york.ac.uk. Lion Shahab, Lion.shahab@ucl.ac.uk |
| Notes | New to 2024  Funding: Yorkshire Cancer Research (UK) |

ISRCTN61193406

| Study name | Do e-cigarettes help smokers quit when not accompanied by intensive behavioural support? A multi-center randomized controlled trial |
| Methods | RCT  Setting: UK  Multicentre. Participants will be recruited mainly from hospitals and GP practices across the UK by the Clinical Research Network. The study is being organized by Queen Mary University of London (QMUL).  Researchers from QMUL will provide the study treatment and conduct follow-up calls. |
| Participants | 1170 people who smoke tobacco cigarettes  Inclusion criteria:   - Adult daily smokers who are motivated to stop smoking - Must own a mobile phone and be willing to try either an online or texting treatment package, or both, or an e-cigarette with or without telephone support - Be happy to receive follow-up calls - Be able to read/write/understand English   Exclusion criteria:   - Women who are pregnant - Currently using an e-cigarette |
| Interventions | 1. Control: NHS Quit Now programme (QN) 2. E-cigarette starter pack with no ongoing support (EC) 3. EC starter pack with helpline support (EC+)   The study will aim to use a refillable EC that is similar to the type used in a previous EC trial (One Kit - Innokin, UK Ecig Store), and one that is compliant with UK regulations, and not produced by a tobacco company. |
| Outcomes | Follow-up at 4 weeks, 6 months and 12 months. CO at 6 and 12 months  Primary outcome measure:  Sustained smoking cessation at 6 months post-TQD. This is measured by asking participants if they have smoked since their TQD at the 6-month follow-up. To be counted as a 'quitter', participants must report smoking no more than 5 cigarettes since 2 weeks post-TQD with no smoking in the previous week, validated by carbon monoxide (CO) reading of < 8 ppm. Participants lost to follow-up will be counted as smokers.  Secondary outcome measures:   - Validated sustained abstinence rates measured by asking smoking status and taking a carbon-monoxide reading at 12 months post-TQD - Validated sustained abstinence rates between 6 and 12 months, measured by asking smoking status and taking a carbon-monoxide reading at 6 and 12 months - Self-reported 7-day point-prevalence abstinence, measured by asking smoking status in last 7 days at 4 weeks, 6 months and 12 months post-TQD - Cigarette consumption in non-abstainers by vaping status, measured by questionnaire at four weeks, 6 and 12 months - Frequency and severity of urges to smoke and withdrawal symptoms, measured by questionnaire at 4 weeks post-TQD - Weight, measured by asking weight at 4 weeks, 6 months and 12 months post-TQD - Respiratory symptoms, measured by questionnaire, at 4 weeks, 6 months and 12 months post-TQD - Treatment adherence and ratings, measured by questionnaire at 4 weeks (and 6 and 12 months for EC arms) - Adverse reactions to EC, measured by questionnaire at 4 weeks, 6 and 12 months post-TQD - Cost-effectiveness of the interventions, measured by questionnaires at baseline, 6 and 12 months - Smokers' and healthcare professionals' views and opinions of the helpline, measured by one-off qualitative interviews separate to the main trial |
| Starting date | Overall trial start date: 1 September 2020  Estimated completion date January 2025 |
| Contact information | Dr Katie Myers Smith, katie.smith@qmul.ac.uk |

ISRCTN82413824

| Study name | Effectiveness of electronic cigarettes compared with combination nicotine replacement therapy for smoking cessation in patients with chronic obstructive pulmonary disease and effect on lung health (ECAL Trial) |
| Methods | Multicentre, two-arm randomized controlled trial with embedded cost-effectiveness and cohort analyses (Prevention, Efficacy)  Setting: England, Scotland, UK  Study to look at CC abstinence, COPD and respiratory outcomes |
| Participants | Target sample size: 1250  Inclusion criteria: COPD diagnosis previously confirmed by post-bronchodilator spirometry (FEV1/FVC < 0.7), any GOLD stage; current smoker (= 5 cigarettes per day); motivated to stop smoking; aged 35 or over  Exclusion criteria: unable to perform spirometry to a satisfactory standard (e.g. due to dementia, lack of teeth, lack of coordination or not having a good oral seal); unsuitable to participate in the trial (e.g. terminal illness, unable to give informed consent); unable to participate in behaviour support calls; severe angina or unstable cardiovascular disease; end stage kidney disease/cirrhosis of the liver; taking NRT, bupropion, varenicline or ECs to stop or reduce smoking; in another trial of smoking cessation or COPD treatment/management; COPD exacerbation or inpatient hospital stay within the last 8 weeks; contraindications to spirometry within the last 12 weeks – tuberculosis infection, cardiac infarction, retinal detachment or surgery on the chest, abdomen, brain, ears or eyes |
| Interventions | EC + telephone support vs NRT + telephone support  Intervention arm: Electronic Cigarettes (EC) - At the baseline visit, participants will be given an EC starter pack and an initial supply of e-liquid (up to 20 mg nicotine/mL). Participants will be provided with instructions on how to continue sourcing further supplies themselves from reputable vendors in their preferred nicotine strength and flavours.    Comparator arm: Combination Nicotine Replacement Therapy (NRT) - Participants will receive up to a 12-week supply of a nicotine patch plus a fast-acting nicotine product to be used in combination. Participants who do not wish to use patches (e.g. due to previous experience with skin irritation) will be offered 2 types of fast-acting products.    Telephone behavioural support: All participants (intervention and comparator arm) will be advised at the baseline visit that they will receive 6 weekly behavioural support telephone calls from stop-smoking advisors which will commence within a few days of the baseline visit. During these calls, the advisor will deliver behavioural support according to the National Centre for Smoking Cessation Training Standard Programme including setting a TQD and providing further support around medication use. |
| Outcomes | Baseline, 4, 26, 52 weeks  Questionnaire, CO monitored. Study to look at CC abstinence, COPD and respiratory outcomes.  Primary outcome  Abstinence from smoking since target quit date (TQD) biochemically validated (exhaled CO < 8 ppm), defined in accordance with the Russell Standard. This will be measured with a questionnaire and exhaled carbon monoxide measurement at 52 weeks post-TQD.  Secondary outcomes   1. Abstinence from smoking for at least 26 weeks biochemically validated (exhaled CO < 8 ppm) measured using a questionnaire at 52 weeks 2. 7-day point prevalence abstinence from smoking biochemically validated (exhaled CO < 8 ppm) measured using a questionnaire at 52 weeks 3. Self-reported abstinence from smoking for at least 26 weeks measured using a questionnaire at 52 weeks 4. Self-reported 7-day point prevalence abstinence from smoking measured using a questionnaire at 4, 26, and 52 weeks 5. Reduction in cigarettes smoked (self-report of any and > 50% reduction) from baseline to 52 weeks measured using a questionnaire, confirmed by reductions in expired CO readings at 52 weeks 6. Reduction in cigarettes smoked (self-report of any and > 50% reduction) from baseline to 26/52 weeks, measured using a questionnaire 7. Continued use of the allocated product measured using a questionnaire at 4, 26 and 52 weeks 8. Withdrawal symptoms and urges to smoke (change from baseline to 1/2/3/4 week) measured using the mood and physical symptom scale (MPSS) 9. COPD Symptoms (change from baseline to 4/26/52 weeks) measured using COPD Assessment Test (CAT) and the Clinical COPD Questionnaire (CCQ) 10. Number of COPD exacerbations over the past 52 weeks (change from baseline to 52 weeks) measured using a questionnaire 11. Number of self-reported upper respiratory tract over the past 52 weeks (change from baseline to 52 weeks) measured using a questionnaire 12. Post bronchodilator spirometry (FEV1, FVC and MMEF change from baseline to 52 weeks). Forced Expiratory Volume in 1 Second (FEV1): The maximal volume of air that can be expired in the first second of a forced expiration from a position of full inspiration (measured in Litres (L) and also expressed as the % predicted for age, sex, height and race). Forced Vital Capacity (FVC): The maximal volume of air that can be expired during a forced and complete expiration from a position of full inspiration (measured in L and % predicted). Mean Mid-Expiratory Flow (MMEF): The average flow between 25% and 75% of the FVC manoeuvre (measured in L/sec and % predicted).   Health economic outcomes:   1. Health-related quality of life (EQ-5D-5L) measured using a questionnaire change from baseline to 4/26/52 weeks 2. Use of healthcare resources and costs measured using a questionnaire at 26 and 52 weeks 3. Cost-effectiveness based on cost per quitter and cost per Quality-Adjusted Life-Year (QALY) at 52 weeks, and modelled cost per QALY over a patient's lifetime |
| Starting date | Study registered: October 2023. First enrolment March 2024.  Estimated study completion: September 2025 |
| Contact information | Amanda Farley, a.c.farley@bham.ac.uk  Institute of Applied Health Research, University of Birmingham, UK |
| Notes | New to 2024 update.  Funding: National Institute for Health and Care Research |

Lin 2024

| Study name | Efficacy of Electronic Cigarettes vs Varenicline and Nicotine Chewing Gum as an Aid to Stop Smoking: A Randomized Clinical Trial  ChiCTR2100048156 (Chinese Clinical Trial Registry) |
| Methods | RCT 3 arm open label, multicentre  Setting: 7 sites in China. China-Japan Friendship Hospital (Beijing, China), Peking University Health Science Center (Beijing, China), Beijing Hospital (Beijing, China), Beijing Xiyuan Hospital (Beijing, China), Beijing Geriatric Hospital (Beijing, China), Beijing Dongzhimen Hospital (Beijing, China), and Wuhan Tongji Hospital (Wuhan, China)  Recruitment: Participants were recruited via trial sites, local newspapers, community events, websites, and referrals from other medical institutions.  Inclusion criteria: smoked at least 10 cigarettes per day for at least 5 years, had expired air carbon monoxide (CO) reading of 9 parts per million (ppm) or greater, were aged 25 to 45 years, and were motivated to stop smoking. Age group chosen: “Due to concerns about adverse events being more likely in older age groups, the sample was limited to adults aged 25 to 45 years. Caution is needed in generalizing the results to older smokers.”  Exclusion criteria: pregnancy or breastfeeding, use of stop-smoking medication during the previous 30 days, ever used ECs for 7 days or longer, history of severe psychiatric illness, unwillingness to use study products, and current diagnosis of cancer or in remission from cancer for less than 1 year. |
| Participants | Total N = 1068  EC = 409; varenicline = 409; NRT = 250  33.5% female; mean age 33.9 (SD 3.1); mean CPD 16.0 (SD5.3); mean FTND 4.1 (SD 2.1)  EC use at baseline: no  Motivation to quit: 50.4% had made previous quit attempt; 49.6% had not made previous quit attempt |
| Interventions | 1. EC arm. A cartridge-based EC product called RELX Wuxian (RELX Technology (30 mg/mL nicotine salt for 2 weeks and 50 mg/mL after that)). Leaflet with product use instructions. Supplied for 12 weeks free. Choice of 3 flavours: mung bean, watermelon, and ice cream.  Participants were instructed to use 30 mg/mL cartridges of their preferred flavour for the first 2 weeks and 50 mg/mL cartridges after that, but were asked to continue using 30 mg/mL or reverse to it if they did not like the higher strength. One cartridge was expected to last for 3 days. At the baseline session, 10 cartridges were provided, with an option to request additional supplies at 1-month and 2-month follow-ups (up to 30 cartridges altogether). Participants were instructed to start using their EC ad lib from the next day and stop smoking completely from their TQD onward.  2. Varenicline arm (0.5 mg, once a day for 3 days; 0.5 mg, twice a day for 4 days; and 1 mg, twice a day, after that). Supplied for 12 weeks free. Participants received a 12-week supply of varenicline (Chantix; Pfizer) and a leaflet with product use instructions. Participants were instructed to take varenicline, 0.5 mg, once per day for the first 3 days, followed by 0.5 mg twice a day for the next 4 days and 1 mg twice a day from day 8, as per the China Clinical Guidelines for Tobacco Cessation. The product was purchased from the manufacturers. Participants were instructed to start using varenicline from the next day and stop smoking completely from their TQD onward.  3. NRT arm: nicotine chewing gum 2 mg (for smokers of 20 cigarettes per day) or 4 mg (> 20 cigarettes per day). Supplied for 12 weeks free  Participants received a 12-week supply of nicotine chewing gum (Johnson & Johnson) product use instruction leaflet. Nicotine gum was selected as the most widely used form of NRT in China. Three boxes containing 105 pieces of the gum each were provided at each monthly contact, with an option to request additional supplies if needed. Participants who smoked up to 20 cigarettes per day received 2 mg nicotine gum, those smoking 20 or more cigarettes per day received 4 mg nicotine gum. Both strengths were provided with the fresh mint flavour. Supplies were bought from the manufacturer. Participants were instructed to use 8 to 12 pieces per day during the first 6 weeks, 4 to 8 pieces per day during weeks 7 and 8, and 2 to 4 pieces per day during the final 4 weeks, asper China Clinical Guidelines for Tobacco Cessation. Participants were instructed to use their NRT from the next day and stop smoking completely from the TQD onward.  All groups: At the 3-month visit, participants were told that they could continue to use their products as needed, but would have to purchase them themselves. A leaflet was provided with information on where the products could be bought. At the last visit, participants received a $60 shopping voucher.  All groups: accompanied by minimal behavioural support (an invitation to join a self-help internet forum). WeChat group for motivational support  All participants set up their target quit date (TQD), normally 2 weeks after the baseline visit.  All participants received a $40 shopping voucher as compensation for their time and travel. The baseline visit took approximately 30 to 45 minutes. At the last visit, participants received a $60 shopping voucher. |
| Outcomes | Baseline, 1, 2, 3, 4, 5, 6 months  CO measured at all time points  Baseline and 6 months – blood pressure (BP) and heart rate |
| Starting date | Study start date May 2021 |
| Contact information | Nicholas I. Goldenson, Juul Labs, Inc., 1000 F Street NW, Suite 800, Washington, DC 20004, United States. Email: Nicholas.Goldenson@juul.com  Funding: This study was funded by Juul Labs, Inc. |
| Notes | New to 2024 update  This study was published then withdrawn. We will monitor to see when this is re-published.  Authors stated: “109 participants, who should be in NRT group, were wrongly placed in the EC group, while another 109 participants, who should be in EC group, were wrongly placed in the NRT group*."* |

Malik 2023

| Study name | Protocol for randomized, two arm parallel, clinical trial for effectiveness of THR products in Low and Middle Income Counties |
| Methods | RCT  Recruitment: outpatient clinics and advertisements will be used for the recruitment of participants and directed to contact the trial site by phone, email, or through the study website. Randomisation: A web-based application will be used to issue a computer-generated sequence for randomization by the principal investigator.  Setting: Pakistan |
| Participants | Target N = 258  Inclusion: Adults who smoke tobacco cigarettes among the general population in Low and Middle Income Countriss and have the motivation to quit.  Exclusion: pregnant/ breastfeeding; taking any other NRT and/or enrolled in any other smoking cessation program; any contraindications to products such as cardiovascular history; and/or suffering from a major illness with a prognosis of less than 1 year. |
| Interventions | (1) E-cigarettes (18mg/ml) with individual counseling  (2) Nicotine patches (21mg) with individual counseling.  Both groups: On allocated quit day, the participants will stop smoking and use study product daily for the next twelve weeks. |
| Outcomes | Baseline, weeks 1, 2, 4, 8, 12, 18, 24, and 52.  12 week treatment.  Abstinence from CC; Use of CC; AEs; Withdrawal;  Exhaled carbon monoxide assessment will be used at the trial site to quantify biochemically validated smoking abstinence. |
| Starting date | Not stated |
| Contact information | Madeeha Malik, ceo@cyntaxhealthprojects.com |
| Notes | Added in 2025. Industry funded (Foundation for a Smoke Free World) |

Murray 2020

| Study name | Yorkshire Enhanced Stop Smoking (YESS) study: a protocol for a randomized controlled trial to evaluate the effect of adding a personalized smoking cessation intervention to a lung cancer screening programme |
| Methods | RCT  Setting: Yorkshire, UK |
| Participants | Actual recruitment: 1001 people who smoke tobacco cigarettes (target 1040)  Participants are aged 55 to 80, registered with a general practitioner (GP) in the Leeds Clinical Commissioning Group area and registered as a current or ex-smoker in primary care databases  Inclusion criteria:   - Attended a lung health check (LHC) and consent to participate in the Yorkshire Lung Screening Trial (YLST) - Have smoked within the last month - Have an exhaled carbon monoxide (CO) reading ≥ 6 ppm - Have agreed to see an SCP on the mobile unit   Exclusion criteria:   - Any individual who does not have an LDCT scan - Unable to provide informed consent |
| Interventions | Arm 1: enhanced, personalized smoking cessation (SC) support package, including CT scan images. SC support over 4 weeks comprising behavioural support, pharmacotherapy and/or a commercially available e-cigarette  Arm 2: continued standard best practice |
| Outcomes | Follow-up contact will be requested at 4 weeks, 3 months, and 12 months, with a 2-week window to accommodate participant availability.  The primary objective is to measure 7-day point prevalent carbon monoxide (CO)-validated SC after 3 months.  Secondary outcomes include CO-validated cessation at 4 weeks and 12 months, self-reported continuous cessation at 4 weeks, 3 months and 12 months, attempts to quit smoking and changes in psychological variables, including perceived risk of lung cancer, motivation to quit smoking tobacco, confidence and efficacy beliefs (self and response) at all follow-up points. |
| Starting date | January 2019 and December 2020 with follow-up data collection ending December 2021. Study completion March 2022. |
| Contact information | Professor Rachael L Murray; rachael.murray@nottingham. ac.uk |

NCT01842828

| Study name | Spain-UK-Czech E-cigarette Study (SUKCES) |
| Methods | Randomized controlled trial, open-label pilot study  Setting: smoking cessation clinics in London, Madrid and Prague  Recruitment: via smoking cessation clinics |
| Participants | 220 people who smoke, seeking help to quit  Inclusion criteria:   - 18 years or older - Want help to quit   Exclusion criteria:   - Pregnant or breastfeeding - Enrolled in other research - Currently using EC |
| Interventions | - Standard care plus **4 weeks EC supply** - **Standard care** only |
| Outcomes | - CO-validated continuous abstinence at 4 and 24 weeks post-TQD - Withdrawal symptoms at 1 and 4 weeks post-TQD - EC use - EC taste and satisfaction compared to conventional cigarettes - Adverse events |
| Starting date | December 2013 |
| Contact information | Peter Hajek, p.hajek@qmul.ac.uk |

NCT02398487

| Study name | Head-to-head comparison of personal vaporizers versus cig-a-like: prospective 6-month randomized control design study (VAPECIG 2) |
| Methods | Randomized, parallel-assignment, open-label trial  Setting: Italy, community |
| Participants | Estimated enrolment: 200  Inclusion criteria:   - (People who smoke) in good general health - Committed to follow trial procedures   Exclude if:   - Recent vaping history (stopped vaping < 3 months ago) - Use of any other form of non-combustible nicotine-containing products (chewable tobacco or nicotine replacement therapy) - Symptomatic cardiovascular disease - Clinical history of asthma and COPD - Regular psychotropic medication use - Current or past history of alcohol abuse - Use of smokeless tobacco or nicotine replacement therapy - Pregnancy or breastfeeding |
| Interventions | Comparison between 2 types of EC; 'personal vaporizers' and 'cig-a-like' |
| Outcomes | 24 weeks:   - Smoking cessation - Smoking reduction |
| Starting date | October 2014. Actual completion December 2015.  Authors unable to provide date when study will be available. |
| Contact information | Riccardo Polosa |

NCT02590393

| Study name | The role of nicotine and non-nicotine alkaloids in e-cigarette use and dependence |
| Methods | Randomized, parallel-assignment, double-blind trial  Setting: smoking research clinic, USA  Recruitment: volunteers |
| Participants | Estimated enrolment: 375  Inclusion criteria:   - Have no known serious medical conditions - Are 18 to 65 years old - Smoke an average of at least 10 cpd - Have smoked at least 1 cumulative year - Have an expired air CO reading of at least 10 ppm - Are able to read and understand English   Exclude if: multiple, related to baseline health status |
| Interventions | - Switch to standard **nicotine EC** use for 8 weeks - Switch to **ECs with same nicotine but very low non-nicotine alkaloid levels** - Switch to **ECs with very low nicotine** and non-nicotine alkaloids |
| Outcomes | Primary:   - CO levels at 8 weeks   Secondary:   - EC use - EC solution use - Cigarette use, at 8 weeks |
| Starting date | May 2016. Study completion 2022.  Emailed author Feb and March 2024 no response. |
| Contact information | Jed Rose, jed.rose@duke.edu |
| Notes | "This is not a smoking cessation study; people who smoke will not be asked to quit smoking, and e-cigarettes will not be used as a medical device or therapy." |

NCT03277495

| Study name | Predictors and consequences of combustible cigarette smokers' switch to standardized research e-cigarettes |
| Methods | RCT. Randomized, parallel assignment  Setting: USA |
| Participants | Estimated enrolment 120 participants  Nicotine EC = 60; placebo EC = 60  Inclusion criteria: ≥ 21 years; ≥ 7 cpd ≥ 1 yr; breath CO ≥ 10 ppm; interested in reducing combustible cigarette use; willing to try EC; attend in-person assessments for 5 months; English-speaking; women who are of childbearing age cannot be pregnant and must agree to use an approved form of birth control during the study.  Exclusion criteria: current use of any smoking cessation medication or participation in a smoking cessation programme or study; daily EC use; pregnancy; no 2 members of the same household may participate in this study. |
| Interventions | EC: Standardized Research E-Cigarette (SREC)  Participants will be stratified by sex and use of menthol cigarettes and randomly assigned with a 1:1 allocation ratio to one of two conditions:   1. Active comparator: nicotine SREC. The liquid in the e-cigarette refills contains nicotine and comes in the following flavours: tobacco, menthol, blueberry, and watermelon. 2. Placebo comparator: placebo SREC   The liquid in the e-cigarette refills does not contain nicotine and comes in the following flavours: tobacco, menthol, blueberry, and watermelon. |
| Outcomes | 3, 4, 5 to 13, 14, 18 weeks  Combustible cigarette use  Abstinence from combustible cigarettes (defined as no cigarette smoking in the past 7 days)  The total number of cigarettes smoked in the 7 days prior to the last assessment  CO level. BP. Heart rate. Weight. Self-report of respiratory symptoms. Fagerstrom Test for Nicotine Dependence |
| Starting date | Estimated starting date June 2022. Estimated completion date: August 2024 |
| Contact information | Kathleen Diviak, PhD 312-996-2327 kdiviak@uic.edu |
| Notes | New to 2022 update |

NCT03625986

| Study name | Does switching to nicotine containing electronic cigarettes reduce health tisk markers |
| Methods | RCT. Prospective, parallel-group, randomized, double-blind, placebo-controlled study  Setting: Penn State Milton S. Hershey Medical Center, USA |
| Participants | Estimated enrolment: 240  Inclusion criteria: age 21 to 70 years; smoke regular, filtered cigarettes or machine-rolled cigarettes with a filter ≥ 5 cpd for ≥ 12 months (CO ≥ 6 ppm at baseline visit); no serious quit attempt in prior month; willing to stop cigarette consumption and switch to an EC and to attend regular visits over a 7-week period  Exclusion criteria: unstable or significant medical condition such as COPD, kidney disease, or liver disease in the past 12 months or severe immune system disorders, uncontrolled mental illness or substance abuse or use of illicit drug/prescription, history of a seizure or seizure medication. Use of any non-cigarette nicotine delivery product in the past 7 days (including EC); use of hand-rolled, roll-your-own cigarettes; allergy to propylene glycol or vegetable glycerin; pregnancy or breastfeeding |
| Interventions | EC: Pod  The electronic cigarette (e-cig) used in this study will be the Standardized Research Electronic Cigarette (SREC). The SREC product is a pod-based device and comprises a replaceable pre-filled liquid reservoir ("pod") and a rechargeable power supply unit.  Arm 1. Experimental: Nicotine-containing electronic cigarette  The experimental group will be provided with and encouraged to use a Standardized Research Electronic Cigarette (SREC) with liquid containing 58 mg/mL nicotine for the duration of 6 weeks.  Arm 2. Placebo comparator: Non-nicotine electronic cigarette  The placebo group will be provided with and encouraged to use a Standardized Research Electronic Cigarette (SREC) with liquid containing 0 mg/mL nicotine for the duration of 6 weeks. |
| Outcomes | 3 weeks, 6 weeks, 10 weeks (phone)  3 weeks and 6 weeks after switching  NNAL, FEV1, CO, plasma cotinine concentration, Fagerstrom Test for Nicotine Dependence mean total score, cpd, abstinence from cigarettes and other tobacco (not including e-cigs) CO < 6 ppm, total score on Minnesota Nicotine Withdrawal Scale, EC use days, self-reported abstinence |
| Starting date | Actual start date 22 April 2022. Study completion date: 23 February 2024. |
| Contact information | Jessica Yingst, DrPH 7175314637, jyingst@phs.psu.edu  Nicolle Krebs, MS 7175315673, nkrebs@pennstatehealth.psu.edu |
| Notes | New to 2022 update |

NCT03862924

| Study name | Health effects of the standardized research e-cigarette in smokers with HIV (ProjectSREC) |
| Methods | RCT  12 week study. Randomization: computerized urn randomization  Setting: Brown University, USA |
| Participants | Target N = 72  HIV positive CC smokers, who are not ready or willing to quit smoking  Inclusion Criteria: diagnosed with and engaged in care for HIV (defined as at least one HIV clinic medical appointment within the past six month period. At least 18 years of age. Smoke at least 5 cigarettes per day for longer than one year. Exhaled CO greater than 5 at BL  Exclusion Criteria: intention to quit smoking in the next 30 days. Using pharmacotherapy for smoking cessation. Using electronic cigarettes more than 2 days/week. Unstable medical or psychiatric condition (defined as hospitalization). Medical contraindications to nicotine (unstable angina, uncontrolled hypertension, or recent cardiovascular event, including hospitalization). Psychotic symptoms. Substance use disorder other than nicotine dependence.Past-month suicidal ideation or past-year suicide attempt. Pregnant or nursing.  Specific population characteristic: HIV positive  Motivated to quit smoking: No.  EC use at baseline: No. |
| Interventions | 1) EC arm. Standardized Research Electronic Cigarette. 6-weeks of free EC (a standardized form developed by the NIH) and encouraged to use them whenever they would smoke a regular cigarette.  2) Control arm. Continue to smoke their usual brand of CC.  At week 6, all participants receive advice to stop smoking and referral to the RI Department of Health Quitline (a state-funded smoking cessation resource/program), if desired. |
| Outcomes | Baseline, weekly for 6 weeks, 12 weeks  Change, weekly to week 6: CC use, heart and lung function (e.g. BP).  Change from baseline to 6 weeks: CO, serum biomarkers, toxicant levels (e.g.NNAL), pulmonary function (Forced expiratory volume in 1 sec (FEV1)).  Study aims to assess: 1) the feasibility and acceptability of EC distribution in people with HIV; 2) the effect of EC use on smoking behaviors; and 3) the change in cardiopulmonary symptoms and biomarkers in smokers who transition from CC to EC use. |
| Starting date | Start date March 2022. Estimated completion date: December 2024. |
| Contact information | Patricia Cioe PhD, patricia\_cioe@brown.edu  Jasminette Dilorenzo BA, jasminette\_dilorenzo@brown.edu |
| Notes | Ongoing study added 2025 |

NCT03962660

| Study name | Harm reduction for tobacco smoking with support of tobacco-replacing electronic nicotine delivery systems (HaRTS-TRENDS) |
| Methods | Parallel, randomized controlled trial  Setting: USA  Recruitment: from prominent Housing First programmes serving chronically homeless people who are often affected by multiple psychiatric, medical and substance-use disorders. The proposed sample will be recruited from a highly vulnerable and marginalized population in a tight-knit urban community. |
| Participants | Estimated enrolment: 94  Inclusion criteria:   - Having a history of chronic homelessness according to the widely-accepted federal definition - Being a current DESC client living in 1 of DESC's participating permanent supportive housing projects - Being between 21 and 65 years of age - Being a daily smoker (> 4 cigarettes/day in the past year with a breath CO ≥ 6 ppm or salivary cotinine test at level 1 if CO < 6 ppm) - Having adequate English language skills to understand verbal information and communicate in the study   Exclusion criteria:   - Use of other tobacco products besides cigarettes ≥ 9 days in the past month - Refusal or inability to consent to participation in research - Constituting a risk to the safety and security of other clients or staff |
| Interventions | - Intervention: HaRTS-TRENDS: 4 individual sessions delivered in the context of the interventionist's pragmatic harm-reduction mind set paired with a compassionate, advocacy-oriented 'heart-set' or style. It comprises the delivery of 4 manualized components, including:   - a) participant-led tracking of preferred smoking outcomes,   - b) elicitation of participants' harm-reduction goals and their progress toward achieving them,   - c) discussion of the relative risks of various nicotine delivery systems,   - d) instruction in using ENDS. Additionally, HaRTS-TRENDS entails provision of commercially available ENDS.  - Standard care: The 4-session, individual standard care control condition entails the well-documented and evidence-based 5 As intervention (i.e. Ask about nicotine use, Assess use, Advice to quit smoking, Assist with exploring current smoking/planning smoking cessation, Arrange follow-up). Part of arranging follow-up is the recommendation to call the smoking quitline, which can supply additional counselling and nicotine replacement therapy. |
| Outcomes | Primary outcomes, measured across the 12-month follow-up:   - Biologically-verified nonsmoking (i.e. self-reported nonsmoking if corresponding CO measure is < 8) in the past 7 days - Urinary concentration of a tobacco-specific nitrosamine   Secondary outcomes, measured across the 12-month follow-up:   - Self-reported smoking intensity is the mean number of cigarettes participants report smoking per day in the 7 days prior to the assessment. - Self-reported smoking frequency is the number of days participants report smoking in the 7 days prior to the assessment - CO level - Urinary cotinine - FEV1% - 10-item Clinical COPD Questionnaire - EQ-5D-5L   Other outcomes:   - Smoking craving - Side effects of ENDS |
| Starting date | 9 May 2019 |
| Contact information | Tatiana M Ubay, tatiubay@uw.edu |

NCT04003805

| Study name | Biomarkers of exposure and effect in standardized research e-cigarette (SREC) users |
| Methods | Design: RCT  Setting: USA |
| Participants | Estimated enrolment: 125  Inclusion criteria:   - 18 to 65 smokers willing to stop smoking and completely switch to EC or medicinal nicotine - ≥ 5 cigarettes daily and not using any other nicotine or tobacco product; biochemically confirmed - Smoking daily for at least 1 year and no serious quit attempts   Exclusion criteria:   - Regular tobacco or nicotine product use other than cigarettes - Currently using NRT or other tobacco cessation products - Significant immune system disorders, respiratory diseases, kidney or liver diseases or any other medical disorders that may affect biomarker data; taking anti-inflammatory medications; unstable health conditions; unstable mental health; excessive drinking; positive toxicology screen for illicit any drugs: pregnant or breastfeeding   For a full list see NCT record. |
| Interventions | EC: Standardized Research E-cigarette (SREC)  Arm 1: Experimental: Switching from Smoking Cigarettes to E (SREC)  The device operates at a single output voltage (3.30 ± 0.05 V) and uses sealed disposable 3 mL cartridges with tobacco-flavoured e-liquid (~350 puffs/cartridge). The concentration of nicotine in e-liquid is 15 mg/mL, and the vehicle composition is 50:50 propylene glycol and glycerin. The device uses a battery that can be recharged via a micro USB port.  Arm 2: Experimental: Switching from smoking cigarettes to nicotine mini-lozenge  We will use commercially available nicotine mini-lozenges containing 2 or 4 mg nicotine/lozenge (Nicorette, manufactured by GlaxoSmithKline). Dose will be determined per instructions on the package (e.g. if smoking within 30 minutes upon awakening, then 4 mg dose will be prescribed). |
| Outcomes | 1 year  4 and 8 weeks for formaldehyde-DNA adducts and oxidative DNA adduct 8-oxo-dG in DNA  Biomarkers: TNE, NNAL, NNN, PneT, mercapturic acids HMPMA, 2-HPMA, 3-HPMA, formaldehyde-DNA adducts, oxidative DNA adduct 8-oxo-dG in DNA, NNN and nornicotine, HPB-releasing DNA adducts  cpd, product use (EC and nicotine lozenges), CC avoidance |
| Starting date | Actual start date: 11 May 2022. Estimated study completion date: January 2025 |
| Contact information | Hanna Vanderloo, RN, MSN 612.624.4983, hannav@umn.edu |
| Notes | New to 2022 update |

NCT04058717

| Study name | Low nicotine cigarettes plus electronic cigarettes |
| Methods | RCT: randomized, parallel-group assignment, 2 x 2 factorial design  Setting: USA |
| Participants | Actual enrollment: 88. Estimated enrolment 240 participants  Inclusion criteria:   - Meet lifetime diagnostic criteria for a current or lifetime unipolar or bipolar mood disorder - Smoke > 5 cigarettes per day for at least the prior 12 months - No serious cigarette smoking quit attempt or use of any FDA-approved smoking cessation medication in the prior 30 days; no plans to quit smoking within the next 3 weeks - Willing to both switch to a different type of cigarette that may contain a different amount of nicotine and to try an EC to substitute for some of their cigarettes   Exclusion criteria:   - Unstable or significant medical condition in the past 3 months - Uncontrolled mental illness or substance abuse, or inpatient treatment for these in the past 6 months or current suicide risk - Use of any non-cigarette nicotine delivery product or EC - Use illegal drugs/prescription drugs - Pregnancy or breastfeeding   For a full list see NCT record. |
| Interventions | EC: type of EC not reported  Arm 1 Experimental: NNC cigarettes + high nicotine-containing e-cigarette. Participants are provided with normal nicotine content (NNC) cigarettes (11.6 mg nicotine/cigarette) plus e-cigarette with high nicotine e-liquid.  Arm 2 Experimental: NNC cigarettes + zero nicotine containing e-cigarette. Participants are provided with normal nicotine content (NNC) cigarettes (11.6 mg nicotine/cigarette) plus e-cigarette with zero nicotine e-liquid.  Arm 3 Experimental: VLNC cigarettes + high nicotine-containing e-cigarette. Participants are provided with very low nicotine content (VLNC) cigarettes (0.2 mg nicotine/cigarette) plus e-cigarette with high nicotine e-liquid.  Arm 4 Experimental: VLNC cigarettes + zero nicotine-containing e-cigarette. Participants are provided with very low nicotine content (VLNC) cigarettes (0.2 mg nicotine/cigarette) plus e-cigarette with zero nicotine e-liquid. |
| Outcomes | 4, 8, 12 and 16 weeks  Urinary NNAL (4-(methylnitrosamino)-1-(3-pyridyl)-1-butanol)  Exhaled carbon monoxide  Kessler-6 score measure of serious psychological distress  Penn State Cigarette Dependence Index  Penn State Electronic Cigarette Dependence Index  Cigarette abstinence. No cigarette use in the past 7 days and exhaled carbon monoxide < 6 ppm |
| Starting date | Actual start date: 1 June 2021. Estimated completion date: Aug 2024. |
| Contact information | Nicolle Krebs, MS 717-531-5673, nkrebs@pennstatehealth.psu.edu  Jonathan Foulds, PhD 717-531-3504, jfoulds@psu.edu |
| Notes | New to 2022 update |

NCT04063267

| Study name | Electronic cigarettes as a harm reduction strategy in individuals with substance use disorder |
| Methods | Parallel-group, randomized trial  Recruitment/setting: Not specified |
| Participants | Actual enrollment: 48. Estimated enrolment: 240  Inclusion criteria:   - Smokes at least 10 cpd - Meet DSM-V AUD and/or OUD within the past year, interested in reducing cpd - Able to provide consent - Use a cell phone, are willing/able to receive and respond to daily text messages about their cigarette use and e-cigarette use on their cell phone - Provide 1 additional contact, and are willing to use an e-cigarette for 3 weeks   Exclusion criteria:   - Pregnant and/or breastfeeding (self-reported) - Currently using smoking cessation medications (including other forms of NRT, bupropion, or varenicline) - Enrolled in a smoking cessation programme or another cessation trial - Have used an e-cigarette in the past 14 days - Have used any other tobacco products (pipe, cigar, cigarillos, snuff, chewing tobacco, rolling tobacco, or hookah/shisha) in the past 30 days - Report having a history of asthma, other airways diseases, or heart disease |
| Interventions | E-cigarettes arm:  Participants will be encouraged to substitute e-cigarettes for combustible cigarettes in order to reduce nicotine withdrawal symptoms.  Nicotine Replacement Therapy arm:  Nicotine patches and gum to last them the first week based on their baseline recorded smoking. Participants will be advised to use both a 21 mg nicotine patch and 4 mg nicotine for cravings. |
| Outcomes | Baseline, 3 weeks.  AEs/SAEs. |
| Starting date | Start date: October 2020  Estimated completion date 30 June 2024 |
| Contact information | NYU Langone Health, Scott.Sherman@nyulangone.org |
| Notes | In SRNT abstract no eligible data. To confirm that the SRNT abstract is linked to this study. |

NCT04218708

| Study name | Electronic cigarettes as a harm reduction strategy among people living with HIV/AIDS |
| Methods | RCT  Setting: NYU Langone Health, USA |
| Participants | Actual enrollment: 64. Estimated enrolment 120. [SRNT abstract N=43]  Inclusion criteria:   - Current Combustible Cigarette (CC) smokers (more than 5 packs in a lifetime; smokes 4 or more days/week), at least 10 cigarettes per day on days they smoke CC - Motivated to quit smoking (at least a 5 on a 10-point Likert scale) - Be willing to use an e-cigarette or NRT for 12 weeks   Exclusion criteria:   - Pregnancy or breastfeeding - Stated diagnosis of any medical condition (angina/heart disease) precluding use of nicotine patch or gum, or by self-report in screening questionnaire. Reporting a history of severe or untreated cardiopulmonary disease such as asthma or emphysema - Reporting using NRTs or e-cigarettes within the last 30 days - Have untreated/are undergoing current treatment for psychiatric illness or cognitive impairment |
| Interventions | EC: Pod. NIDA Standardized Research E-cigarettes (SREC) (15 mg/mL nicotine in tobacco flavour)  Arm 1: counselling + nicotine replacement therapies NRT  A research assistant (RA) trained in motivational interviewing and qualitative methods will support the PI to deliver counselling sessions and conduct interviews. Briefly, during each visit, with help of the RA, participants will provide exhaled CO and saliva cotinine test, and complete surveys in REDCAP using a tablet, allowing programmed logic checks and skip patterns to minimize burden. The RA will also deliver brief motivational counselling tailored to the participant's readiness to quit and arm in the study (NRT). Participants will also receive their NRT to last them to the following visit based on their baseline smoking.  Arm 2: Counselling + Standardized Research E-cigarettes (SREC)  Participants in the SREC arm to practice using the SREC and RA to give them instructions to return with their SREC and used refill tanks on every visit. A research assistant (RA) trained in motivational interviewing and qualitative methods will support the PI to deliver counselling sessions and conduct interviews. Briefly, during each visit, with help of the RA, participants will provide exhaled CO and saliva cotinine test, and complete surveys in REDCAP using a tablet, allowing programmed logic checks and skip patterns to minimize burden. The RA will also deliver brief motivational counselling tailored to the participant's readiness to quit and arm in the study (SREC). Participants will also receive their SREC to last them to the following visit based on their baseline smoking. |
| Outcomes | Week 1, 2, 4, 6, 8, 12  Change in cigarettes per day (cpd). Smoking reduction will be measured by a combination of self-report, text message data and changes in CO and saliva cotinine between baseline and end of treatment.  Assessing differences in nicotine withdrawal symptoms  Assessing differences in e-cigarette dependency  Assessing differences in nicotine use  Assessing differences in use of substance use  Assessing differences in side effects associated with e-cigarette use |
| Starting date | Study start date: 17 June 2021. Estimated study completion date: October 2024. |
| Contact information | Omar El Shahawy, MD 1-646-501-2587, omar.elshahawy@nyulangone.org |
| Notes | New to 2022 update |

NCT04238832

| Study name | Impact of non-cigarette tobacco product formulation on reinforcement value and use in current smokers  Short title: Salt-based e-cigarette |
| Methods | RCT  Setting: USA, South Carolina |
| Participants | Actual enrollment: 24. (Estimated enrollment: 30)  Inclusion criteria:   - Daily cigarette smoker - Interested in using non-cigarette tobacco product - Have a smartphone that can receive text messages and has access to the internet or have an email account they check daily (necessary for daily diary completion)   Exclusion criteria:   - Additional tobacco use criteria - Additional medical criteria |
| Interventions | Salt-base nicotine  Free-base nicotine |
| Outcomes | Most preferred product (time frame: Lab visit 2, occurring approximately 1 week after the initial screening/baseline visit)  Participants complete a preference assessment in which they choose between the salt liquid, free-base liquid, or a traditional cigarette in a series of trials. The outcome of this assessment is the product chosen most often by each participant.  Cigarettes per day (time frame: Week 2 of study)  The average number of cigarettes smoked per day during the 1-week sampling period  Biomarkers (i.e. expired CO, cotinine) will corroborate self-reported indices of use. |
| Starting date | 23 June 2020. Estimated completion: August 2021 |
| Contact information | Tracy Smith, smithtra@musc.edu |

NCT04452175

| Study name | Official title: Cigarette consumption after switchinG to high or low Nicotine strENght E-cigaretteS In Smokers with schizophrenia spectrum disorders: a 12-month randomized, double-blind multicentre trial  Brief title: Cigarette consumption after switchinG to high or low nicotine strENght E-cigaretteS In Smokers with schizophrenia (GENESIS)  NB: The GENESIS protocol (NCT04452175) now incorporates SCARIS protocol, NCT01979796. Antismoking effects of electronic cigarettes in subjects with schizophrenia and their potential influence on cognitive functioning: design of a randomized trial. Smoking Cessation And Reduction In Schizophrenia (The SCARIS Study). clinicaltrials.gov/show/NCT01979796 |
| Methods | RCT  Multicentre: Italy, Russia, Ukraine, UK  Collaborators:   - Juul Labs, Inc. - St. Petersburg State Pavlov Medical University - Bashkir State Medical University - Ukrainian Institute on Public Health Policy - University of Surrey - Eclat Srl |
| Participants | Estimated enrolment: 260  Inclusion criteria:   - Adult (> 18 yrs) - Regular smoking (> 10 cigarettes a day; for at least 1 year) - Exhaled breath CO (eCO) level > 7 ppm - Not currently attempting to quit smoking or wishing to do so in the next 30 days; this will be verified at screening by the answer "NO" to the question "Do you intend to quit in the next 30 days?" - Schizophrenia spectrum disorder diagnosis (schizophrenia, delusional disorder, schizoaffective disorder, personality disorder, schizoid personality disorder, etc) by DSM-V criteria - Understand and provide informed consent - Able to comply with all study procedures   Exclusion criteria:   - Institutionalized patients - Acute decompensation of schizophrenia spectrum disorder symptoms within the past month - Change in antipsychotic treatment within the past month - No recent history of hospitalization for any serious medical condition within 3 months prior to screening, as determined by the investigator - Myocardial infarction or angina pectoris within 3 months prior to screening, as determined by the investigator - Current poorly-controlled asthma or COPD - Pregnancy, planned pregnancy, or breastfeeding. Any female participant who becomes pregnant during this study will be withdrawn. - People who have a significant history of alcoholism or drug/chemical abuse within 12 months prior to screening, as determined by the investigator - Accepting to take part in a smoking cessation programme - People who regularly use any recreational nicotine (e.g. e-cigarettes) or tobacco product (e.g. tobacco heated products, oral smokeless) other than their own cigarettes within 30 days of screening - People who have used smoking cessation therapies (e.g. varenicline, bupropion, or NRT) within 30 days of screening - People who are still participating in another clinical study (e.g. attending follow-up visits) or who have recently participated in a clinical study involving administration of an investigational drug (new chemical entity) within the past 3 months - People who have, or who have a history of, any clinically significant neurological, gastrointestinal, renal, hepatic, cardiovascular, psychiatric, respiratory, metabolic, endocrine, haematological or other major disorder that, in the opinion of the investigator or their appropriately qualified designee, would jeopardize the safety of the participant or impact on the validity of the study results |
| Interventions | - Experimental: high 5%. Intervention: JUUL e-cigarette - Active comparator: low 1.7%. Intervention: JUUL e-cigarette |
| Outcomes | Primary outcomes:   - Rates of participants with continuous smoking abstinence at 6 months; time frame: 24 weeks - Self-reported continuous smoking abstinence at 6 months from the previous visit, biochemically verified by exhaled CO measurements of ≤ 7 ppm   Secondary outcomes   - Rates of participants with continuous smoking abstinence at 12 months (time frame: 52 weeks) - Rates of participants with continuous smoking reduction at 6 months (time frame: 24 weeks) - Rates of participants with continuous smoking reduction at 12 months (time frame: 52 weeks) - Proportion of AEs (time frame: 24 weeks) - Absolute change in PANSS (time frame: 24 weeks) - Absolute change in mCEQ (time frame: 24 weeks) - Absolute change in Chester Step Test-derived values (time frame: 24 weeks) - Change in App-derived endpoints (self-rated mental health SRMH) (time frame: 24 weeks) |
| Starting date | Actual start date 30 October 2021. Estimated study completion: February 2023 (NCT record update posted on 23 May 2022) |
| Contact information | Pasquale Caponnetto, p.caponnetto@unict.it |

NCT04521647

| Study name | Effects of menthol in e-cigarettes on smoking behaviors |
| Methods | Randomized cross-over  Setting: Connecticut Mental Health Center, USA |
| Participants | Actual enrollment: 10. Estimated enrolment 85  Inclusion criteria: ≥ 21 years, use combustible cigarettes  Exclusion criteria: none |
| Interventions | EC: type not stated  Arm 1: menthol flavour. Participants will receive 5% nicotine in an EC. Participants will receive 2 nicotine concentrations via EC. Each exposure will be 10 3-sec puffs and ad libitum use.  Arm 2: tobacco flavour. Participants will receive 5% nicotine in an EC. Participants will receive 2 nicotine concentrations via EC. Each exposure will be 10 3-sec puffs and ad libitum use. |
| Outcomes | Baseline and 2, 5, 15, 30, 45, 60, 90, 120, and 180 minutes after nicotine exposure (plasma nicotine levels), 2 weeks (CO), 3 weeks, 5 weeks (BP and heart rate)  Primary outcomes: cigarette craving; plasma nicotine levels; carbon monoxide  Secondary outcomes: EC craving; irritation/harshness; liking of EC; coolness; nicotine withdrawal; stimulation; EC use; cigarette use. Other outcomes measured: heart rate, blood pressure, pulse oximetry |
| Starting date | Study start date: 1 November 2020. Completion date: 25 July 20253 |
| Contact information | Asti Jackson, PhD 4752414904, asti.jackson@yale.edu |
| Notes | New to 2022 update |

NCT04649645

| Study name | International randomized controlled trial evaluating changes in oral health in smokers after switching to combustion-free nicotine delivery systems (SMILE) |
| Methods | RCT  Setting: multicentre: Italy, Moldova, Poland, UK, and Indonesia |
| Participants | Estimated enrolment 606 participants  Inclusion criteria:   - Demonstrate understanding of the study and willingness to participate in the study by providing a signed written informed consent - Healthy, not taking regular medications for chronic medical conditions - Adults, age at least 18 years old - Presence of at least 10 natural anterior teeth in total (cuspid to cuspid, lower and upper jaw) - Presence of at least 18 'scorable' teeth with scorable facial and lingual surfaces. Teeth that are grossly carious, orthodontically banded, exhibiting general cervical abrasion and/or enamel abrasion, and third molars will not be included in the tooth count. - Willingness and ability to comply with the requirements of the study, including installing an APP on their digital device, e.g. smartphone or tablet   For Arms A and B, participants have to be:   - Regular smokers, defined as: smoked for at least 5 consecutive years prior to screening. Smoked > 10 and < 30 cigarettes per day (cpd) with an exhaled breath carbon monoxide (CO) level ≥ 7 ppm at screening - Willing to regularly use any nicotine or tobacco product other than their own conventional cigarette brand within 14 days prior to screening - Willing to change to use of study products or, if randomized to Arm A, continuing to use their own brand of conventional cigarettes for the whole duration of the study   For Arm C, participants have to be:   - Never-smokers, defined as: never smoked or who have smoked < 100 cigarettes in their lifetime and none in the 30 days prior to screening with an exhaled breath CO level < 7 ppm at screening - Willing to not smoke or use any form of tobacco or nicotine-containing products for the whole duration of the study   Exclusion criteria:   - Pregnancy - Presence of extensive crown or bridge work, dental implants, and/or rampant decay (per investigator/examiner discretion) - Significant oral soft tissue pathology or any type of gingival overgrowth, other than plaque-induced gingivitis and mild periodontitis (Stage I) - Moderate-to-severe periodontitis (Stage II, III and IV) based on 2017 World Workshop on the Classification of Periodontal and Peri-Implant Diseases and Conditions, which require: Detectable interdental Clinical Attachment Loss (CAL) ≥ 3 mm at ≥ 2 non-adjacent teeth. Buccal or oral CAL ≥ 3 mm with pocketing ≥ 5 mm detectable at ≥ 2 teeth - Removable dentures or fixed and removable orthodontic appliance (except fixed lingual wires) - Significant history of alcoholism or drug abuse (other than tobacco/nicotine) within 24 months prior to screening, as determined by the investigator - A course of treatment with any medications or substances (other than tobacco/nicotine) which: interfere with the cyclo-oxygenase pathway (e.g. anti-inflammatory drugs including aspirin and ibuprofen) within 3 days prior to each visit or are known to have antibacterial activity (e.g. antibiotics) within 7 days prior to each visit |
| Interventions | Standard arm (Arm A): own tobacco cigarette brand  Intervention arm (Arm B): combustion-free nicotine delivery system (C-F NDS)  Control arm (Arm C): no smoking or use of any nicotine/tobacco products |
| Outcomes | Oral health parameters and teeth appearance, comparing short- and long-term impact on periodontal health between smokers continuing with conventional cigarette smoking, those switching to combustion-free nicotine delivery systems (C-F NDS), and never-smokers over 18 months |
| Starting date | Not yet recruiting (last updated February 2021)  Estimated study start date: Feb 2021. Estimated primary completion date: Feb 2023. Estimated completion April 2023. |
| Contact information | Principal investigator: Antonio Pacino, DDS, Addendo srl, Catania, Italy  info@addendo.net |

NCT04708106

| Study name | Characterization of product use in smokers switching from cigarettes to a RELX electronic nicotine delivery system  Setting: USA |
| Methods | Design: RCT, multicentre, open-label, parallel-cohort study |
| Participants | Estimated 200  Inclusion criteria:   - Provides voluntary consent to participate in the study as documented on the signed informed consent form (ICF) - Is 22 to 65 years of age, inclusive, at the time of consent - Is willing to comply with the requirements of the study - Reports typically smoking 5 or more combustible cpd at screening - Has been a daily smoker for at least 12 months prior to screening. Brief periods of non-smoking (e.g. up to ~7 consecutive days due to illness, trying to quit, participation in a study where smoking was prohibited) ≥ 56 days prior to screening will be permitted at the discretion of the investigator. - Has a positive urine cotinine test (≥ 200 ng/mL) at screening and test visit 1 - Has an eCO value > 10 ppm at screening and test visit 1 - Has daily access to a cell phone for daily product use reporting - If female, meets one of the following criteria: - If of childbearing potential - agrees to use one of the accepted contraceptive regimens from at least 30 days prior to the first product use and during the study. An acceptable method of contraception includes one of the following: - Abstinence from heterosexual intercourse - Hormonal contraceptives (birth control pills, injectable/implant/insertable hormonal birth control products, transdermal patch) - Intrauterine device (with or without hormones) OR agrees to use a double barrier method (e.g. condom and spermicide) during the study - If a female of non-childbearing potential - should be surgically sterile (i.e. has undergone complete hysterectomy, bilateral oophorectomy, or tubal ligation) or in a menopausal state (at least 1 year without menses), as confirmed by follicle stimulating hormone (FSH) levels.   Exclusion criteria:   - Has a history or presence of clinically significant uncontrolled gastrointestinal, renal, hepatic, neurologic, haematologic, endocrine, oncologic, urologic, pulmonary, immunologic, psychiatric, or cardiovascular disease, or any other condition that, in the opinion of the investigator, would jeopardize the safety of the subject or impact the validity of the study results - Has a clinically significant abnormal finding on the physical examination, medical history, vital signs, electrocardiogram (ECG), or clinical laboratory results, in the opinion of the investigator - Has a positive test for human immunodeficiency virus (HIV), hepatitis B surface antigen (HBsAg), or hepatitis C virus (HCV) at screening - Has a positive COVID-19 test at screening or during the study - Has had an acute illness (e.g. upper respiratory infection, viral infection) within 14 days prior to test visit 1 - Has a fever (> 100.5°F) at screening or test visit 1 - Has a body mass index (BMI) greater than 40.0 kg/m2 or less than 18.0 kg/m2 at screening - Has a systolic blood pressure < 90 mmHg or > 150 mmHg, diastolic blood pressure < 40 mmHg or > 95 mmHg, or heart rate < 40 bpm or > 99 bpm at screening - Has a post-bronchodilator forced expiratory volume in 1 second:forced vital capacity (FEV1:FVC) ratio < 0.7 and FEV1 < 50% of predicted at screening - Has a post-bronchodilator FEV1 increase ≥ 12% and > 200 mL from pre- to post-bronchodilator at screening - Has used an ENDS product on > 7 days during each of the 3 months prior to screening or any use from screening to test visit 1 other than as may be required for this study - Reports use of a very-low-nicotine content cigarette (e.g. Moonlight, Spectrum, VLN) as usual brand - Has used nicotine-containing products other than manufactured cigarettes (e.g. ENDS products (e-cigarettes), roll-your-own cigarettes, bidis, snuff, nicotine inhaler, pipe, cigar, chewing tobacco, nicotine patch, nicotine spray, nicotine lozenge, or nicotine gum) within 14 days prior to test visit 1 - Has used any products for the purpose of smoking cessation, including, but not limited to, nicotine replacement therapies, varenicline (Chantix), or bupropion (Zyban) from 30 days prior to screening through the duration of the study - Is a self-reported puffer (i.e. draws smoke from the cigarette into the mouth and throat but does not inhale) - Is postponing a planned smoking quit attempt in order to participate in the study - Has a history of drug or alcohol abuse within 12 months prior to screening, as determined by the investigator - Is allergic to PG or glycerin - Has a positive urine drug or alcohol breath test at screening or test visit 1. At the discretion of the investigator, a subject testing positive for tetrahydrocannabinol may be permitted to participate if the subject reports use by routes other than inhalation. - If female, the subject is pregnant, breastfeeding, or intends to become pregnant from screening through the duration of the study - Has been treated for depression, diabetes, asthma, emphysema, or chronic obstructive pulmonary disease within 12 months prior to test visit 1 - Has previously been diagnosed with any form of cancer, except for basal cell or squamous epithelial carcinomas of the skin that have been resected at least 12 months prior to screening 1 - Has a planned surgery that would occur during study participation - Has participated in a previous clinical study for an investigational drug, device, biologic, or tobacco product within 30 days prior to test visit 1 - Is or has a first-degree relative (e.g. spouse, parent, sibling, or child) who is a current or former employee of a tobacco or ENDS manufacturer or is a named party or class representative in litigation with the tobacco or ENDS industry - Is or has a first-degree relative (e.g. spouse, parent, sibling, or child) who is a current employee of the clinic site - Is or has a first-degree relative (e.g. spouse, parent, sibling, or child) who is a current employee of the sponsor - Has previously taken part in (from completion of any baseline measurements), has been withdrawn from, or has completed this study - In the opinion of the investigator, the subject should not participate in this study. |
| Interventions | RELX ENDS tobacco flavour ad libitum use of the RELX ENDS tobacco flavour product  RELX ENDS menthol flavour ad libitum use of the RELX ENDS menthol product  Ad libitum use of the RELX ENDS tobacco and menthol flavour products |
| Outcomes | Primary outcomes:  Weekly RELX ENDS product use; time frame: 56 days. Self-reported number of RELX ENDS pods started each week  Daily number of cigarettes smoked; time frame: 56 days. Self-reported number of cigarettes smoked daily by study week  Number of puffs from the RELX ENDS each day; time frame: 56 days. Self-reported number of puffs from the RELX ENDS daily by study week (0, < 100, ≥ 100 per day)  Secondary outcomes:  Biomarkers of exposure measured in blood; time frame: baseline, day 28, and day 56; change in carbon monoxide concentration in the blood  Biomarkers of tobacco exposure measured in urine; time frame: baseline, day 28, and day 56; change in creatinine-adjusted NNAL, NNN, 3-HPMA, CEMA, HMPMA, S-PMA, HEMA, 1-OHP, o-toluidine, nicotine equivalents, and propylene glycol excreted in urine  Subjective effects as measured by the Penn State (Electronic) Cigarette Dependence Index (PS(E)CDI); time frame: baseline, day 14, day 28, day 42, and day 56. Change in product dependence as measured by the PSCDI/PS(E)CDI total score. Total scores may range for 0 to 20, with higher levels of dependence associated with higher scores.  Subjective effects as measured by the Cough Questionnaire; time frame: baseline, day 14, day 28, day 42, and day 56. Change in self-reported cough symptoms as measured by responses to the Cough Questionnaire  Subjective effects as measured by the Questionnaire of Smoking Urges-Brief (QSU-Brief); time frame: baseline, day 14, day 28, day 42, and day 56. Change in smoking urge as measured by the QSU-Brief factor 1 and factor 2 scores. Questionnaire responses are measured on a Likert scale range of 1 (not at all) to 7 (extremely).  Subjective effects as measured by the Minnesota Tobacco Withdrawal Scale-Revised (MTWS-R); time frame: baseline, day 14, day 28, day 42, and day 56. Change in withdrawal symptoms as measured by the MTWS-R total score, which includes the DSM-5 and craving items from the Minnesota Tobacco Withdrawal Scale. Questionnaire responses are measured on a Likert scale range of 0 (none) to 4 (severe)).  Subjective effects as measured by the Modified Product Evaluation Scale (mPES); time frame: baseline, day 14, day 28, day 42, and day 56. Change in product assessments as measured by mPES satisfaction, psychological reward, aversion, and relief subscale scores. Questionnaire responses are measured on a Likert scale range of 1 (not at all) to 7 (extremely).  Subjective effects as measured by the Future Intent to Use Questionnaire; time frame: baseline, day 14, day 28, day 42, and day 56. Change in future intent to use cigarettes and ENDS products as measured by responses to the Future Intent to Use Questionnaire Questionnaire responses are measured on a Likert scale range of 1 (extremely unlikely) to 7 (extremely likely)  Subjective Effects as measured by the Health Effects Perceptions Questionnaire; time frame: baseline and day 56. Harmful and addictiveness perceptions as measured by responses to the Health Effects Perceptions Questionnaire  Puff topography - number of puffs; time frame: baseline, day 28, and day 56. Change in the number of puffs during a 1-hour puff topography session  Puff topography - puff duration; time frame: baseline, day 28, and day 56. Change in puff duration during a 1-hour puff topography session  Puff topography - puff volume; time frame: baseline, day 28, and day 56. Change in puff volume during a 1-hour puff topography session  Puff topography - peak puff flow rate; time frame: baseline, day 28, and day 56; change in peak puff flow rate during a 1-hour puff topography session  Puff topography - average flow rate; time frame: baseline, day 28, and day 56. Change in average flow rate during a 1-hour puff topography session  Puff topography - inter-puff interval; time frame: baseline, day 28, and day 56. Change in inter-puff interval during a 1-hour puff topography session  RELX ENDS product use; time frame: day 28 and day 56; change in pod weight during a 1-hour topography session  Incidence of product-use emergent adverse events (safety and tolerability); time frame: 56 days  Incidence of product-use emergent adverse events |
| Starting date | Study start date: 15 October 2020. Estimated completion date: April 2021 |
| Contact information | Study Director: Donald Graff. Principal Investigator: Mark Adams, MD  Cheerain HK Limited. AMR, USA. Pillar Clinical Research, USA. QPS, USA. No contact details provided. |

NCT04709471

| Study name | E-cigarette nicotine study |
| Methods | Design: RCT. Parallel-group assignment  Setting: USA  Start date: 20 January 2021. Estimated completion date: September 2021 |
| Participants | Actual enrollment 77. Estimated: 75  Eligibility criteria include at least 21 years old, use e-cigarettes and tobacco cigarettes regularly, not planning to quit in the near future, and not pregnant, breastfeeding or planning to become pregnant or breastfeed in the next 2 months  Additional criteria will be evaluated to assess for eligibility. |
| Interventions | Experimental: Switch to low-nicotine e-cigarettes: switch to e-cigarettes containing 60% of baseline e-cigarette nicotine content. Device: Juul e-cigarette. Participants will switch to Juul pods containing less nicotine.  Experimental: Reduce number of e-cigarette pods: reduce e-cigarette use to 60% of baseline number of pods per week  Behavioural: Reduction: participants will reduce the number of Juul pods that they use  No Intervention: Use e-cigarettes as usual: continue using nicotine e-cigarettes as usual |
| Outcomes | Baseline, 4 week reduction period, 8 weeks.  All participants will complete a web-based follow-up survey and provide a breath CO sample 4 weeks after study completion (i.e., 8 weeks after randomization) to assess tobacco use, ENDS use, quit attempts, and behavioral economic measures.  Primary outcome measure:  Feasibility; time frame: Baseline and the 4-week reduction period. The investigators will assess compliance with study e-cigarettes and compare the percentage of non-study e-cigarette use between conditions to determine which behaviour-changing strategy is more feasible.  Combustible cigarette smoking; time frame: Baseline and the 4-week reduction period. The investigators will compare change in number of cigarettes per day between conditions.  Cigarette dependence; time frame: Baseline and the 4-week reduction period. The investigators will compare change in cigarette dependence between conditions using the PATH dependence measure.  E-cigarette dependence: time frame: Baseline and the 4-week reduction period. The investigators will compare change in e-cigarette dependence between conditions using the PATH dependence measure.  Secondary outcome measure:  Cigarette demand; time frame: Baseline and the 4-week reduction period. The investigators will compare change in cigarette demand using the Brief Assessment of Cigarette Demand task.  E-cigarette demand; time frame: Baseline and the 4-week reduction period. The investigators will compare change in e-cigarette demand using a version of the Brief Assessment of Cigarette Demand task adapted for e-cigarettes. |
| Starting date | 20 January 2021. Study completion date: June 2022. |
| Contact information | Elias M Klemperer, PhD802-656-1641, elias.klemperer@med.uvm.edu |

NCT04725656

| Study name | Concentration Impact Nicotine Salt (CINS) |
| Methods | Design: RCT |
| Participants | Estimated enrolment: 312  Inclusion criteria:   - Adult (≥ 18 years old) smokers (at least 5 TC per day for at least 12 months) - Motivated to quit smoking as evidenced by signing the informed consent form at trial enrolment specifying that a target quit date will be set - Saliva cotinine of > 50 ng/mL at screening - Willing to participate in the trial even if allocated to the control group - Ability to communicate well with the investigator and to understand and comply with the requirements of the study - Signed informed consent form   Exclusion criteria:   - Known hypersensitivity/allergy to a content of the e-liquid - Pregnancy or breastfeeding - Intention to become pregnant during the course of the study - Regular use of EC or tobacco heating systems - Use of NRT, varenicline, or bupropion in the month prior to the screening visit - Smoke tobacco combined with marijuana and do not currently want to quit marijuana use - Participation in an interventional trial within 30 days prior to the screening visit - Legal incapacity or limited legal capacity at screening - Any circumstances or conditions which, in the opinion of the investigator, may affect full participation in the study or compliance with the protocol |
| Interventions | Active comparator: Active arm, low concentration (18 mg/mL) nicotine salt e-liquids. Procedure: Smoking cessation counselling: smoking cessation counselling at baseline, week 1, week 2 and week 4  Other: Open system vape device and nicotine salt e-liquids; ad libitum use of nicotine salt e-liquids during 3 months  Active comparator: Active arm, high concentration (59 mg/mL) nicotine salt e-liquids. Procedure: Smoking cessation counselling: smoking cessation counselling at baseline, week 1, week 2 and week 4  Other: Open system vape device and nicotine salt e-liquids; ad libitum use of nicotine salt e-liquids during 3 months  Control group: Receive only smoking cessation counselling. Procedure: Smoking cessation counselling: smoking cessation counselling at baseline, week 1, week 2 and week 4 |
| Outcomes | Primary outcome:  7-day point prevalence tobacco abstinence (in terms of non-inferiority); time frame: 1 month. Defined as no smoking, i.e. "not a puff", self-reported and confirmed by exhaled carbon monoxide (< 10 ppm) and urinary anabasine levels (< 3 ng/mL) when using low vs. high nicotine salt concentration e-liquids  Volume of e-liquid used (in terms of superiority); time frame: 1 month; volume of e-liquid used when using low vs high nicotine salt concentration e-liquids  Secondary outcome:  7-day point prevalence tobacco abstinence (in terms of non-inferiority); time frame: 1 month. Defined as no smoking, i.e. "not a puff", self-reported and confirmed by exhaled carbon monoxide (< 10 ppm) and urinary anabasine levels (< 3 ng/mL) when using low vs. high nicotine salt concentration e-liquids  Volume of e-liquid used (in terms of superiority); time frame: 1 month; volume of e-liquid used when using low vs high nicotine salt concentration e-liquids  Liking/rating of trial product (active arms); time frame: 1 and 3 months. Questions regarding helpfulness in refraining from smoking, how satisfying and how good the e-cigarette tastes compared to the tobacco cigarettes, if they would recommend the assigned trial product to another smoker, and any potential practical problems they might have with the handling  Respiratory symptoms; time frame: up to 12 months; checklist with specific questions regarding shortness of breath, wheezing, cough or phlegm  Adverse events; time frame: up to 12 months; checklist with specific questions regarding presence or absence of nausea, sleep disturbance, throat/mouth irritation, or other  Total nicotine amount vaped; time frame: 1 and 3 months  Total volume of e-liquid consumed; time frame: 1 and 3 months |
| Starting date | Start date: Jan 2024. Estimated study completion date: 31 Dec 2024 |
| Contact information | Evangelia Liakoni, MD0041316325461, evangelia.liakoni@insel.ch |

NCT04946825

| Study name | Quit smoking study for people who use e-cigarettes. A randomized controlled trial of smoking cessation treatment for young adult dual users of combustible and electronic cigarettes |
| Methods | RCT. Randomized factorial assignment  Setting: community; University of Vermont, USA |
| Participants | Estimated enrolment 390  Inclusion criteria: young adult; smokes tobacco cigarettes; uses EC; interested in quitting tobacco  Exclusion criteria: pregnancy or breastfeeding; ≥ 1 contraindications for NRT |
| Interventions | EC: type not stated  NRT: patch and lozenge  A) NRT plus text messages to quit CCs only  B) NRT plus text messages to quit CCs and ECs simultaneously  C) text messages alone to quit CCs only  D) text messages alone to quit CCs and ECs simultaneously |
| Outcomes | Baseline, 3 months, 6 months  CO confirmed 7-day point-prevalence abstinence at the end of treatment (i.e. 3 months after randomization)  CO biochemically confirmed prolonged 30-day abstinence, 3-month follow-up (i.e. end of treatment) and 6-month follow-up (3 months after the end of treatment)  Self-reported abstinence, 7 days, 30 days  Attempts to quit combustible cigarettes (CC), cpd, CC dependence |
| Starting date | Study start date: 27 June 2021. Estimated study completion: January 2024. |
| Contact information | Elias Klemperer, PhD 8026561641, elias.klemperer@med.uvm.edu  Shaun Meyers, BA 8026568681, shaun.meyers@uvm.edu |
| Notes | New to 2022 update |

NCT05023096

| Study name | Potential effects of electronic nicotine delivery system flavor regulations on African American menthol smokers (RVA Flavors) |
| Methods | RCT  Virginia Commonwealth University, USA |
| Participants | Actual enrollment 71. Estimated enrolment: 210  Inclusion criteria: 21+ years; identify as Black/African-American (single or multi-race); used ≥ 5 cigarettes per day for ≥ 1 year (biochemically confirmed); regular cigarette brand menthol or mint flavoured; EC use in the past 30 days; no intent to quit smoking in the next 6 months; previous quit attempt using evidence-based method; mobile phone, willing to receive calls/text  Exclusion criteria: unwilling to use EC; report other tobacco use > 10 days in past 30 other than combustible cigarettes; unstable or significant medical condition in the past 12 months; > 15 days of marijuana or other illegal drug use in the past 30 days; pregnancy/breastfeeding |
| Interventions | EC: type not stated  Arm 1: Menthol + tobacco. Both menthol and tobacco-flavoured liquids for EC are available to choose from.  Arm 2: Tobacco - only tobacco-flavoured liquid is available for EC.  Arm 3: Unflavoured - only unflavoured liquid is available for EC.  Participants are instructed to smoke their usual brand of menthol cigarettes normally for 7 days and avoid using any other tobacco products. After this baseline week, participants are randomized to 1 of 3 EC flavour conditions; all contain 5% nicotine (menthol + tobacco, tobacco, unflavoured) with equal probability and provided with a supply of their condition-specific EC and asked to use it in place of their usual menthol cigarettes for the next 6 weeks. |
| Outcomes | Week 1, week 6  Change in: average daily cigarette use; carbon monoxide exposure; urinary NNAL; urinary propylene glycol exposure; average daily ENDS use  Willingness to substitute from cigarettes to EC (ENDS); measure of substitution for condition-specific tobacco products will be assessed using drug purchase tasks. Choices made during this task are not reinforced.  Willingness to pay for ENDS (week 6); willingness to pay for condition-specific tobacco products will be assessed using drug purchase tasks. Choices made during this task are not reinforced. |
| Starting date | Study start date: 14 April 2022. Estimated primary completion date: June 2025 |
| Contact information | Andrew J. Barnes, PhD 804-827-4361, abarnes3@vcu.edu  Caroline O. Cobb, PhD, cobbco@vcu.edu |
| Notes | New to 2022 update |

NCT05144542

| Study name | Risk and benefits of electronic cigarettes to older smokers at high risk for lung cancer |
| Methods | RCT  Setting: M. D. Anderson Cancer Center, Texas, USA |
| Participants | Estimated enrolment: 330  Inclusion criteria: meeting National Comprehensive Cancer Network (NCCN) guideline for lung cancer screening; daily or non-daily smoker; interested in trying ECs to change CC smoking behaviour; willing and able to complete two spirometry sessions  Exclusion criteria: used ECs on more than 2 days in the past 30 days; meet criteria for current major depressive disorder (MDD) or suicidality; report more than once weekly of tobacco products other than CCs during the past 30 days; ever diagnosis of lung cancer, have uncontrolled or unstable medical condition; spirometry forced expiratory volume in 1 second (FEV1) percentage reading < 50; pregnancy/breastfeeding |
| Interventions | EC: type not specified  GROUP A: Participants smoke their usual brand of cigarettes for 26 weeks. Participants use smartphone to answer questions about nicotine cravings and mood, and log daily smoking activity every day for up to 182 days. Participants complete questionnaires over 50 minutes and undergo collection of urine sample at 1, 6, 12, and 26 weeks, and collection of blood samples at 6, 12, and 26 weeks. Participants may also undergo measurement of CO levels at 1, 6, 12, and 26 weeks.  GROUP B: Participants vape EC for 26 weeks. Participants use smartphone to answer questions about nicotine cravings and mood, and log daily smoking activity every day for up to 182 days. Participants complete questionnaires over 50 mins and undergo collection of urine sample at 1, 6, 12, and 26 weeks, and collection of blood samples at 6, 12, and 26 weeks. Participants may also undergo measurement of CO levels at 1, 6, 12, and 26 weeks. |
| Outcomes | 1, 6, 12, and 26 weeks, and collection of blood samples at 6, 12, and 26 weeks  Primary outcome measure: cigarettes per day, diary data of combustible cigarette use over last 24 hours  Secondary outcome measures: high-sensitivity C-reactive protein (hs-CRP); white blood cells (WBC); 8-epi prostaglandin F2 alpha (8-epi-PGF2a). All from blood draws at weeks 0, 6, 12, and 26 |
| Starting date | Start date: 7 March 2022. Estimated completion date: 30 April 2025 |
| Contact information | Jason Robinson, PHD 713-792-0919, jdrobinson@mdanderson.org |
| Notes | New to 2022 update |

NCT05199480

| Study name | Understanding the impact of cartridge-based electronic cigarettes and generated aerosols on cardiopulmonary health |
| Methods | Two groups recruited: EC users and demographically matched non-EC users.  NCT record: 'Randomised' 'parallel assignment'.  Virginia Commonwealth University, USA |
| Participants | Actual enrolment: 64  Inclusion criteria for EC group: ≥ 21 yrs; used EC (≥ 3 times/week for ≥ 3 months)  Inclusion criteria for the group not using EC (Non-e-cigarette group: ≥ 21 yrs  Exclusion criteria: use of cigarettes for 15 days or more in the past 60 days; use of other tobacco products (cigars, hookah, smokeless) weekly or more frequently in the past 60 days; use of marijuana or any illicit or prescription drugs for non-medical use weekly or more frequently in the past 60 days; allergy to propylene glycol or vegetable glycerin; evidence of cardiovascular, pulmonary, renal, hepatic, metabolic, or cerebral diseases; disorder or use of medication that affects cardiopulmonary health; pregnancy/breastfeeding |
| Interventions | EC: commercially available cartridge-based EC device  Arm 1: E-cigarette liquid type 1 (tobacco flavour)  A commercially available cartridge-based device with tobacco-flavoured liquid. Participants will be instructed to use at least one study product daily in place of their own EC during the intervention period.  Arm 2: E-cigarettes liquid type 2 (tobacco flavour)  A commercially available cartridge-based device with tobacco-flavoured liquid. Participants will be instructed to use at least one study product daily in place of their own EC during the intervention period.  Arm 3: No e-cigarettes. No e-cigarette use |
| Outcomes | Baseline, 2 weeks  Change in peak oxygen consumption (VO2 peak)  Change in expiratory volume  Change in skeletal muscle O2 utilization  Change in maximal microvascular dilation |
| Starting date | Study start date: 10 January 2022. Study completion 30 July 2024. |
| Contact information | Paula Rodriguez Miguelez, PhD804-396-4498, prodriguezmig@vcu.edu |
| Notes | New to 2022 update |

NCT05205811

| Study name | A randomized controlled trial to determine the effects of combination zonisamide and bupropion on switching to an electronic cigarette |
| Methods | RCT  Rose Research Center, USA |
| Participants | Estimated enrolment: 180  Inclusion criteria: 21 to 65 yrs; ≥ 10 commercially available cigarettes per day, for the last 12 months (CO reading ≥ 10 ppm); interested in switching to an EC; smartphone with text message and data capabilities  Exclusion criteria: unhealthy or cannot participate in the study for any reason; PHQ-9 score greater than 9, or a score greater than 0 on item #9; plans to use an FDA-approved smoking cessation product; high blood pressure, coronary heart disease, structural cardiac disease; BMI ≤ 15.0 kg/m2 or > 40.0 kg/m2; depression, anxiety, or nicotine withdrawal within 30 days of screening, or during the study, taking antidepressants, psychoactive medications or medications that prolong QTc  For full list see NCT record |
| Interventions | EC: JUUL  Zonisamide  Bupropion  Arm 1: Combination zonisamide and bupropion with EC  After the first week of EC use (JUUL), participants will be given bupropion (150 mg each morning for days 1 to 3, then 300 mg daily) with zonisamide (100 mg daily). The combination of zonisamide and bupropion use will continue for 7 weeks of treatment, and EC use will continue until the end of the study (an additional 4 weeks). EC for ad libitum use for 2 weeks prior to complete switch day and for an additional 10 weeks  Arm 2: Bupropion with EC  After the first week of EC use (JUUL), participants will be given bupropion (150 mg each morning for days 1 to 3, then 300 mg daily) with placebo zonisamide. The combination of placebo and bupropion use will continue for 7 weeks of treatment, and e-cigarette use will continue until the end of the study (an additional 4 weeks). EC for ad libitum use for 2 weeks prior to complete switch day and for an additional 10 weeks  Arm 3: Placebo with EC  After the first week of EC use (JUUL), participants will be given placebo bupropion with placebo zonisamide. The combination of these placebos will continue for 7 weeks of treatment, and EC use will continue until the end of the study (an additional 4 weeks). EC for ad libitum use for 2 weeks prior to complete switch day and for an additional 10 weeks |
| Outcomes | Baseline, week 8, week 12, 6 months  Complete switching from combustible cigarettes to JUUL EC as measured by: exhaled carbon monoxide (CO); change in total urinary 4-(methylnitrosamino)-1-(3-pyridyl)-1-butanol (NNAL); change in self-report of daily cigarette and EC use  Seven-day point abstinence at 6 months post-switch, assessed by self-report and confirmed by exhaled CO < 5 ppm. Change in smoking withdrawal symptoms. Change in rewarding and aversive effects of smoking and EC use  AEs; SAEs |
| Starting date | Start date: 14 December 2021. Estimated completion date: 30 June 2024 |
| Contact information | Derek Mercedes 704-350-2999, derek.mercedes@roseresearchcenter.com |
| Notes | New to 2022 update |

NCT05206435

| Study name | Methadone-maintained smokers switching to e-cigarettes (SHINE) |
| Methods | RCT  Butler Hospital, Providence, Rhode Island, United States, 02906 |
| Participants | Estimated enrolment: 240  Inclusion criteria:   - Moderate to heavy cigarette smokers (10 cigarettes/day for > 1 yr; breath CO > 10 ppm) - Receiving methadone for ≥ 3 months and attend at least weekly to receive methadone dose - Interested in switching to EC or NRT   Exclusion criteria:   - Using ECs on > 2 of the past 30 days - Currently use medications that may reduce smoking (e.g. bupropion, varenicline, NRT) - Unstable psychiatric conditions - Near-daily or daily use of marijuana - Pregnancy - Cardiovascular event in the last month, daily medication for asthma or COPD |
| Interventions | EC: type not stated  Arm 1: Electronic cigarettes. Participants in this arm are randomized to receive electronic cigarettes for the 6-week study period. Electronic cigarettes are provided to replace tobacco cigarettes.  Arm 2: Nicotine lozenges. Participants in this arm are randomized to receive nicotine lozenges for the 6-week study period. Nicotine lozenges are provided to replace tobacco cigarettes. |
| Outcomes | Baseline, 6 weeks  Nicotine exposure (urine)  Lung functioning: FVC (changes in Forced Vital Capacity, spirometry); FEV1 (changes in Forced Expiratory Volume (FEV - during the first second), spirometry)  Smoking behaviour and experiences (self-report)  For complete switchers: nicotine exposure; lung functioning (FVC, FEV1, FEV1/FVC); smoking behaviour and experiences |
| Starting date | Study start date: 31 March 2022. Estimated study completion date: 30 June 2024. |
| Contact information | Michael Stein, MD 401-455-6200, michael\_stein@brown.edu  Ana Abrantes, PhD 401-455-6200, ana\_abrantes@brown.edu |
| Notes | New to 2022 update |

NCT05257629

| Study name | Aggressive smoking cessation therapy post-acute coronary syndrome (ASAP) trial |
| Methods | RCT  Setting: hospital  Jewish General Hospital, USA |
| Participants | Estimated enrolment: 798  Inclusion criteria:  Currently hospitalized (or at time of discharge) for ACS. Defined as follows: MI, defined by positive troponin T, troponin I, or CK-MB levels (as defined by institution-specific cut-offs). For definition, see NCT record. CC user; motivated to quit smoking according to the Motivation To Stop Scale (MTSS) (≥ level 5); ≥ 18 years  Exclusion criteria:  Use of any of the following in the 30 days prior to ACS admission: i. Pharmacotherapy (e.g. NRTs, bupropion, or varenicline) for smoking cessation; ii. Nicotine or non-nicotine e-cigarettes; iii. Psychotropic medications (e.g. mood stabilizers, antipsychotics, prescribed opiates and sedatives); iv. Other anti-craving medication (e.g. naltrexone, acamprosate) with the potential to alter substance-seeking behaviours  Pregnancy/breastfeeding  For a full list, see NCT record. |
| Interventions | EC: participant's choice  Arm 1: Combination therapy arm (varenicline and nicotine EC plus counselling)  Patients in the combination therapy arm will be supplied funds and instructions for the purchase of EC and cartridges/pods upon hospital discharge and at the week 4 and 12 clinic visits. As with standard NRTs such as the gum, inhaler, and lozenge, we expect smokers will self-regulate administration according to their withdrawal symptoms. Use will be monitored via self-report for telephone follow-ups. At clinic visits, patients will be asked to bring their EC, used and unused cartridges/pods, and purchasing receipts. Patients will be advised regarding the signs and symptoms of nicotine toxicity and of an allergic reaction.  Arm 2: Varenicline plus counselling  All patients will begin varenicline in-hospital upon randomization. For the first 3 days, patients will take a 0.5 mg tablet once a day. They will then take a 0.5 mg tablet twice a day for the following 4 days, and one 1 mg tablet twice a day from day 8 onward for the remainder of the 12-week treatment. Use will be monitored via self-report for telephone follow-ups and return of all unused tablets at the end of the treatment period. Should a patient experience severe side effects (such as headache, nausea, vomiting, dizziness, dyspepsia, fatigue, insomnia, abnormal dreams, constipation, or flatulence) on day 8 onward, the varenicline dose should be reduced from 1 mg twice daily to 0.5 mg twice daily prior to study medication discontinuation. |
| Outcomes | 1, 2, 8, 18, 24 weeks  Week 4, week 12, and week 52  Number of participants with: 7-day point prevalence smoking abstinence (biochemically-validated); continuous smoking abstinence; prolonged smoking abstinence; change in daily cigarette consumption; ≥ 50% reduction in daily cigarette consumption; point prevalent abstinence or ≥ 50% reduction in daily cigarette consumption at 24 weeks  Frequency of adverse events (AEs) or SAEs  Spirometry measurements (subset) at all other clinic visits (FVC, FEV1, and FEV1/FVC)  O2 cost diagram and COPD Assessment Test (subset) at all other clinic visits  Number of patients averaging ≥ 1 pill of varenicline/day |
| Starting date | Estimated start date: 1 June 2022. Estimated completion date: 7 March 2027 |
| Contact information | Carole Bohbot 514-340-8222 ext 22790  ASAP.Trial@ladydavis.ca, carole.bohbot@ladydavis.ca |
| Notes | New to 2022 update |

NCT05278065

| Study name | Complimentary electronic cigarettes for harm reduction among adult smokers with asthma (SWAP) |
| Methods | RCT  Setting and recruitment: Participants will be adults from the local community with persistent asthma symptoms who are regular combustible cigarette smokers and do not also regularly use ENDS. The study will recruit 30 non-treatment-seeking participants using flyers, advertisements, a website triaging visitors to the Center for Alcohol and Addiction Studies, and through targeted recruitment at community immunology clinic partners at Rhode Island Hospital, USA. |
| Participants | Actual enrollment: 17. Estimated enrolment: 30  Inclusion criteria: 21 to 65 years; Persistent asthma symptoms (i.e. episodic symptoms of airflow obstruction/airway hyper-responsiveness (AHR) as documented in review of medical history); Currently prescribed SABA medication; Past-year smoking of ≥ 5 cigarettes/day (CO ≥ 6 ppm at baseline); Zero breath alcohol during informed consent for participation  Exclusion criteria: Intention to quit smoking during the next 30 days or current engagement in any smoking cessation treatment; Regular EC/ENDS user or using ENDS > 2 days/week; Medical contraindication to nicotine; Pregnancy (due to toxicity of nicotine and tobacco products); Current alcohol dependence (AUDIT > 15); Urine-screened or past-month self-reported use of illicit substances (amphetamine, cocaine, methamphetamine, opioids, benzodiazepines); Current psychosis, mania, or suicidal ideation.  EC use at baseline: No  Motivated to quit smoking: No  Specific population characteristic: people with asthma |
| Interventions | EC: 4th generation and disposable cartridges  Arm 1: Electronic cigarette  Participants in this experimental condition will be provided with a 4th generation EC device and disposable cartridges. Participants will be provided with EC and 5% nicotine e-liquid cartridges for 8 weeks and encouraged at weekly assessments to use the EC any time they would normally smoke. Participants will be able to choose commercially available e-liquid flavours (tobacco) at each weekly assessment.  Arm 2: Smoking-as-usual  Participants in this assessment-only condition will continue smoking-as-usual. |
| Outcomes | Baseline, week 8, week 16. Eight weekly visits to complete follow-up assessments  cpd  EC use  Asthma symptoms  Pulmonary functioning, FEV, FVC, FEF25-75, PEF  CO. Level of exhaled CO assessed with Smokerlyzer  NNAL  Cotinine  Interleukin-6 (IL-6)  Tumour necrosis factor alpha (TNF-a)  Chemokine ligand 9 (CXCL9)  Matrix metallopeptidase 9 (MMP9) |
| Starting date | Start date: 1 May 2022. Study completion date: September 2024 |
| Contact information | Alexander W Sokolovsky, PhD 4018636629, alexander\_sokolovsky@brown.edu  Mary Ellen Fernandez, BA 4018635521, mary\_fernandez@brown.edu |
| Notes | New to 2022 update |

NCT05510154

| Study name | Impact of e-cigarette training on puff patterns, cigarette smoking, and health outcomes among smokers with COPD; COPD e-cigarette topography training |
| Methods | Design: RCT. An open-label, randomized clinical trial of e-cigarette training and training dose amongst smokers with COPD  Smokers with COPD (n = 45) stratified by e-cigarette use history (naïve vs current use) will be randomized (1:1:1) to receive 1) brief advice to switch to e-cigarettes, 2) single-session e-cigarette training, or 3) enhanced e-cigarette training.  Setting: University of Kansas Medical Center (KUMC) campus in Kansas City, Kansas (KS), USA  Study start date March 2022. Study end June 2023. |
| Participants | N = 45  Smokers with COPD (n = 45) stratified by e-cigarette use history (naïve vs current use) will be randomized (1:1:1) to receive 1) brief advice to switch to e-cigarettes, 2) single-session e-cigarette training, or 3) enhanced e-cigarette training.  Inclusion criteria: smokers or dual users diagnosed with COPD; ≥ 21 years old; speak and understand English; smoke on > 25 of the last 30 days for the past 3 months; willing to switch from cigarettes to the study e-cigarette for the duration of the study; have tried but failed to quit smoking in the last year; unwilling to make a pharmacotherapy-assisted quit attempt in the next 30 days  Exclusion criteria: smokers or dual users; use of tobacco products other than cigarettes, including e-cigarettes in the past 30 days; current use of cessation medications; pregnant, planning to become pregnant, or breastfeeding; recent history of cardiovascular or pulmonary events in the past 3 months; household member current or previously enrolled in the study |
| Interventions | EC. The study product is an e-cigarette device and is available for sale in the US.   1. Brief advice to switch to e-cigarettes 2. Single-session e-cigarette training 3. Enhanced e-cigarette training (3 real-time training sessions rather than 1) |
| Outcomes | 12 weeks  Changes in puff duration in seconds from pre- to post-e-cigarette training (time frame: 12 weeks)  Complete switch to e-cigarette (time frame: 12 weeks)  Change in spirometry FVC, FEV1, FEV1/FVC ratio, systolic blood pressure, change in diastolic blood pressure, change in COPD Assessment Test (CAT) score, respiratory symptoms score, change in 6-minute walk test distance |
| Starting date | Study start date March 2022 |
| Contact information | Eleanor Leavens, Assistant Professor, University of Kansas Medical Center |
| Notes | New to 2023 update |

NCT05555069

| Study name | The impact of menthol flavoring on switching in adult menthol smokers |
| Methods | Design: Randomized parallel assignment  Setting. USA; University of Kansas Medical Center |
| Participants | Estimated enrolment: 800 participants  Inclusion criteria: ≥ 21 years of age; smoke ≥ 5 cigarettes per day (CPD); smoke menthol cigarettes for ≥ 6 months; verified smoker (CO > 5ppm); functioning telephone; interested in switching to e-cigarettes  Exclusion criteria: interested in quitting smoking; use of other tobacco products in past 30 days (i.e. cigarillos, cigars, hookah, smokeless tobacco, pipes); e-cigarette use on ≥ 4 of the past 30 days; uncontrolled hypertension: BP ≥ 180 (systolic) or ≥ 105 (diastolic); use of smoking cessation pharmacotherapy in the month prior to enrolment; pregnant, contemplating getting pregnant, or breastfeeding  Motivated to quit: no |
| Interventions | EC: 4th generation nicotine salt-based pod-system e-cigarette in menthol versus tobacco-flavoured e-liquid  Arm 1. Menthol flavour electronic cigarette. 400 adult cigarette smokers will receive 12 weeks of menthol-flavoured electronic cigarettes.  Arm 2. Tobacco flavour electronic cigarette. 400 adult cigarette smokers will receive 12 weeks of tobacco-flavoured electronic cigarettes.  Participants will receive 12 weeks of menthol OR tobacco-flavoured electronic cigarettes to aid in switching from combustible cigarettes. Participants will be instructed on proper use of electronic cigarettes, educated about electronic cigarettes and participate in motivation enhancement and substituting electronic cigarettes for cigarettes. |
| Outcomes | Baseline, 12 weeks. Follow-up will continue to 26 weeks.  Outcomes at 12 weeks  1. Number of participants who switch from cigarettes to electronic cigarettes at week 12  Complete switching is defined as exclusive use of e-cigarettes, confirmed with CO < 6 ppm and predominant switching; defined as use of the e-cigarette with > 50% reduction in CPD. This will compare the effectiveness of menthol versus tobacco e-cigarettes in facilitating switching at week 12.  2. Assessment of respiratory symptoms using spirometry  Spirometry summarizing forced expiratory flow (FEF) 25-75% and the American Thoracic Society Questionnaire will assess acute respiratory symptoms experienced by cigarette and electronic cigarette smokers. This will help assess the tobacco harm reduction of electronic cigarettes.  3. Amount of e-liquid consumed |
| Starting date | 8 November 2022. Estimated primary completion date 30 June 2025 |
| Contact information | Tricia Snow, 816-398-8960 psnow@kumc.edu  PI Nicole Nollen, PhD, University of Kanas Medical Center |
| Notes | New to 2023 update |

NCT05610514

| Study name | Pulmonary and cardiac effects of e-cigarette use in pulmonary patients who smoke cigarettes |
| Methods | Design: Randomized, cross-over, open-label  Setting: Greater Burlington, VT, USA |
| Participants | Actual enrollment: 21. (Estimated enrolment: 25)  Inclusion criteria: men and women 40 years of age or older; current, every-day smoker (5 or more cigarettes per day for one year or longer) confirmed with intake CO of 8 ppm or greater; established pulmonary disease (chronic obstructive pulmonary disease (COPD), chronic bronchitis, emphysema, or asthma-COPD overlap syndrome) confirmed by physician diagnosis and/or current prescription of medication for treatment (i.e. LABA, LAMA, ± ICS, or combination); no intention to quit smoking within the next month  Exclusion criteria: patients who are medically unstable (unstable symptoms, changes in medications or hospitalizations within last 3 months); inability to conduct in-home measurements  Motivated to quit: no |
| Interventions | EC: JUUL/Vuse Alto and pods  Experimental: e-cigarette. Participants in this arm will smoke electronic cigarettes for 2 weeks. E-cigarettes (either JUUL or Vuse Alto) and pods (JUUL: Virginia tobacco flavour at 3% or 5% nicotine concentration; Vuse Alto: golden tobacco flavour at 1.8%, 2.4%, or 5% nicotine concentration) will be provided. In the EC arm, availability of e-cigarettes and altering the availability of financial incentives for abstaining from combustible cigarettes will be investigated.  No intervention: combustible cigarette. Participants in this arm will smoke their usual brand of combustible cigarettes for 2 weeks. |
| Outcomes | Baseline, 2 weeks, 4 weeks  Baseline and change from baseline: FEV1/FVC; lung reactance; oxygen saturation (SpO2); exhaled nitric oxide (FeNO); COPD; blood pressure; heart rate; tobacco use; Fagerstrom Test of Nicotine Dependence (FTND); Wisconsin Inventory of Smoking Dependence Motives-Brief (WISDM-Brief); Minnesota Tobacco Withdrawal Scale (MNWS); Questionnaire on Smoking Urges-Brief (QSU-Brief); health changes |
| Starting date | Starting date 28 April 2022; completion date December 2023 |
| Contact information | Brian R Katz, PhD, 8025511798, Brian.Katz@uvm.edu  Shannon O'connor, 8025511798, shannon.oconnor@uvm.edu |
| Notes | New to 2023 update |

NCT05703672

| Study name | 4th generation e-cigarettes in African American smokers: reducing harm and quitting combustible cigarettes in dual users  Brief title: Switching to e-cigarettes in African-American smokers |
| Methods | Design: Randomized, parallel-assessment interventional study  Setting: Missouri, USA. Swope Health Central, Kansas City, Missouri, United States, 64130. University of Kansas Medical Center, Kansas City, Missouri, United States, 64130 |
| Participants | Estimated N = 500  Inclusion criteria: African-American; ≥ 21 years of age; smoke > 5 cigarettes per day; smoked cigarettes for > 6 months; verified smoker (CO > 5 ppm); interested in switching to EC  Exclusion criteria: interested in quitting smoking; use of smoking cessation pharmacotherapy in the month prior to enrolment; use of other tobacco products in past 30 days (i.e. cigarillos, cigars, hookah, smokeless tobacco, pipes); EC use on > 4 of the past 30 days; uncontrolled hypertension: BP > 180 (systolic) or > 105 (diastolic); heart-related event in the past 30 days; medical contraindications to VAR: unstable cardiac condition (e.g. unstable angina or AMI) cardiac event, or stroke in the past 4 weeks; renal impairment; history of clinically significant allergic reactions; history of epilepsy or seizure disorder; hospitalized for psychiatric issue in past 30 days; active suicidal ideation; pregnant, contemplating getting pregnant, or breastfeeding  Motivated to quit: no |
| Interventions | EC: Nicotine salt pod-based e-cigarette in 5% nicotine  Arm 1: Experimental: varenicline and electronic cigarette  At the end of the 6-week open-label phase, dual users of cigarettes and e-cigarettes will receive 1 mg varenicline to take twice daily for 12 weeks. They will also receive an additional 12 weeks of the nicotine salt-based pod system e-cigarette. Drug: Varenicline Tartrate, 0.5 mg once daily for days 1 to 3, 0.5 mg twice daily for days 4 to 7 and 1.0 mg twice daily from day 8 through week 12. Electronic cigarette: nicotine salt pod-based e-cigarette in 5% nicotine  Arm 2: Placebo comparator: placebo and electronic cigarette  At the end of the 6-week open-label phase, dual users of cigarettes and e-cigarettes will receive placebo pills to take twice daily for 12 weeks. They will also receive an additional 12 weeks of the nicotine salt-based pod system e-cigarette.  Drug: placebo 1 pill (white) once daily for days 1 to 3, one pill (white) twice daily for days 4 to 7 and 1 pill (blue) twice daily from day 8 through week 12. Electronic cigarette: nicotine salt pod-based e-cigarette in 5% nicotine  Arm 3: Open-label electronic cigarette  All participants will receive an initial 6-week supply of the study electronic cigarette. Nicotine salt pod-based e-cigarette in 5% nicotine |
| Outcomes | Baseline, 6 weeks, 12 weeks. FU to 52 weeks  Reduction in toxicant exposure as measured by NNAL excretion from baseline to week 6  CO verified 7-day point prevalence abstinence from cigarettes at week 12 post-randomization |
| Starting date | Estimated study start date 30 June 2023. Estimated completion date 30 November 2024. |
| Contact information | Tricia Snow, MPH, 816-398-8960, psnow@kumc.edu |
| Notes | New to 2023 update |

NCT05815199

| Study name | Effectiveness and impact of counseling enhanced using electronic cigarettes for harm reduction in people with serious mental illness  Brief title: E-cigarettes for harm reduction among smokers with serious mental illness |
| Methods | Design: randomized, parallel-assignment. RCT  Setting: NYU Langone Health, USA |
| Participants | Estimated enrolment: 60  Inclusion criteria: currently smokes 5 or more CPD; age of at least 21 years; has SMI diagnosis (such as schizophrenia, schizoaffective disorders, bipolar disorder, depressive disorders, trauma and stressor-related disorders etc.) as determined using the MINI tool; interested in reducing CC smoking but not necessarily trying to quit  Exclusion criteria: pregnant /breastfeeding; used tobacco other than CC in the past 2 weeks (e.g. EC, cigarillo); currently engaged in an attempt to quit CC; change in dose of their psychotropic medication(s) in the last 30 days; meeting DSM-V criteria for current alcohol or substance use disorder except for nicotine use disorder and active mild alcohol or substance use disorders; past month suicidal ideation/suicide attempt and/or psychiatric hospitalization in the last 30 days  Population: people with serious mental illness (SMI)  Motivated to quit: interested in reducing but not interested in quitting |
| Interventions | EC: NJOY Ace Electronic Cigarette  Intervention period: 8 weeks  Arm 1. Experimental: e-cigarettes (EC)  Interventions: E-cigarette (EC) NJOY Ace; behavioural: harm-reduction counselling; behavioural: Ecological Momentary Intervention (EMI) text messaging  Arm 2. Active comparator: nicotine replacement therapy (NRT)  Interventions: Other: nicotine replacement therapy (NRT) (patches, lozenges and gum); behavioural: harm-reduction counselling; Behavioural: Ecological Momentary Intervention (EMI) text messaging  Description of behavioural intervention for both groups  Behavioural: harm-reduction counselling. At baseline, after randomization, participants will receive their first telehealth session (20 to 25 minutes) from a counsellor trained in motivational interviewing, harm reduction, and smoking cessation. Up to 5 additional sessions will be delivered, 15 to 20 minutes each.  Behavioural: Ecological Momentary Intervention (EMI) text messaging. EMI can be defined as delivering tailored interventions via electronic messages (i.e. regular text messages) that include personalized feedback based on real-time assessment responses and other contextual factors. EMI will take place throughout the intervention period. |
| Outcomes | Baseline, wk 4, wk 8, wk 12  Abstinence from CC (wk 4, wk 8, wk 12). Self-report (daily diary about smoking behaviour) and verified by exhaled carbon-monoxide (eCO) level (< 6 ppm)  Self-reported percent change in CPD (baseline to wk 8, baseline to wk 12)  Change in American Thoracic Questionnaire Score from baseline to wk 12. 8-item questionnaire assessing general thoracic pain  Change in Symptom Check Questionnaire Score from baseline to wk 12. 9-item assessment of chronic obstructive pulmonary disease (COPD) symptoms |
| Starting date | Estimated start date: July 2023. Estimated completion date: March 2024. |
| Contact information | Omar El-Shahawy, 646-501-3587, Omar.ElShahawy@nyulangone.org  Adetayo Fawole, 646-501-3568, Adetayo.fawole@nyulangone.org |
| Notes | New to 2023 update |

NCT05825924

| Study name | Randomized, two arm parallel, clinical trial to compare effectiveness of different tobacco harm reduction products in general adult population in low middle income countries |
| Methods | Design: randomized, parallel-assignment, 2-arm trial  Setting: low-middle-income countries |
| Participants | Estimated enrolment: 258  Inclusion criteria: at least of legal age allowed for smoking in the country, of either gender, regular smokers (minimum 10 cigarettes/day for at least a year) and interested in stopping smoking  Exclusion criteria: pregnant/breastfeeding; using other smoking cessation medications (including other forms of NRT other than patch, bupropion, clonidine, nortriptyline or varenicline); any contraindications to products such as cardiovascular history; major illness with prognosis of less than 1 year  Motivated to quit: yes |
| Interventions | EC: EC 18 mg/mL designed to resemble tobacco cigarettes, aerosol generator, sensor, battery and storage area for liquid. Disposable or rechargeable  Study Arm 1: 18 mg nicotine EC (ad libitum use) for 12 weeks after the nominated quit date  Free EC and sufficient nicotine cartridges (18 mg/mL) supply to last till next in-person visit. Participants will be instructed to use the device ad libitum 1 week before their quit day to familiarize themselves with its operation and on their designated quit day will stop smoking tobacco cigarettes and instead use the EC exclusively for the next 12 weeks. CC users often take 10 to 15 puffs over the course of 5 to 8 minutes, repeating this pattern with each cigarette. EC users may periodically use it throughout the day, and they may or may not take their puffs like those of traditional CCs.  Study Arm 2: 21 mg nicotine patches (one daily) for 12 weeks after the nominated quit date  21 mg nicotine patches supply to last until the next-person visit. Participants will use the nicotine patch daily for 1 week before their quit day to familiarize themselves with its use. On their designated quit day, they will stop smoking and use nicotine patches daily for the next 12 weeks. Usually, a full-strength patch (15 to 22 mg of nicotine) daily for 4 weeks is suggested for use in the majority of smokers, followed by a lower-strength patch (5 to 14 mg of nicotine) for an additional 4 weeks, depending on their body size and smoking habits.  The nicotine patches are applied on the skin and nicotine is delivered at a steady rate. After administration, the peak blood levels are achieved within 6 to 10 hours. The levels remain constant, reducing by 25% to 40% with use of patches once daily. The patch is typically administered every 24 hours for no longer than 12 weeks. The dose of the patches is often determined by daily cigarette consumption and level of addiction.  The duration of counselling will be at least 30 minutes on site. The duration of counselling through telephone will be at least 10 minutes. Participants will be scheduled for a screening visit and a baseline (BL) visit at the trial site. The participants will be scheduled for 8 study visits in total, including 5 treatment sessions and 3 follow-up visits, using both face-to-face interaction at the trial site as well as follow-up on telephone. |
| Outcomes | Weeks 1, 2, 4, 8, 12, 18, 24, and 52  7 day PP. Self-report having smoked no cigarettes in the past 7 days  Number of cigarettes smoked per day assessed using self-reported diaries  AEs (time frame 12 weeks). AEs evaluated using Naranjo Adverse Drug Reaction Probability Scale  Physical signs and symptoms of withdrawal using Fagerstrom test for nicotine dependence  Perception of the product |
| Starting date | Estimated study start date: September 2023. Estimated primary completion date: March 2025 |
| Contact information | Ather Mehmood, FCPS +92518314299, athermehmood70@gmail.com |
| Notes | New to 2023 update  Funded by: Foundation for a Smoke Free World INC |

NCT05881304

| Study name | Switching individuals in treatment for opioid use disorder who smoke cigarettes to the SREC |
| Methods | Design: waiting-list controlled RCT  Setting: Massachusetts General Hospital, USA |
| Participants | Estimated enrolment: 40  Inclusion criteria: 18 +; report daily cigarette smoking (≥ 10 cigarettes per day in the past week); not ready to quit smoking (not planning to quit in the next 30 days); willing to try EC; in stable buprenorphine (BUP) treatment for opioid use disorder at a Massachusetts General Hospital-affiliated primary care clinic (in treatment for ≥ 3 months without changes in BUP dose in the past 2 wks and planning to remain on current BUP treatment for ≥ 3 months)  Exclusion criteria: pregnant/breastfeeding; using non-cigarette nicotine or tobacco products (e.g. EC, cigarillos) recently (> 3 days in past 30 days); report past 30-day use of behavioural or pharmacologic smoking cessation aids; have an unstable psychiatric or medical condition  Motivated to quit: no  EC use at baseline: no |
| Interventions | EC: NIDA standardized research e-cigarette (SREC)  Arm 1. Experimental: Immediate standardized research EC (SREC) provision (iSREC)  Those randomized to the iSREC group will be provided a free 8-week supply of standardized research e-cigarettes (SRECs) and asked to try to switch completely to the SREC.  Arm 2. Active Comparator: Delayed SREC provision waiting-list control (WLC)  Those in the WLC condition will receive SREC provision after an 8-week delay. |
| Outcomes | Baseline, 2 wks, 8 wks  SREC for 8 weeks, either immediately (iSREC), or after an 8-week delay (waiting-list control [WLC]). They will be followed for an additional 4 weeks after SREC provision ends (to 12 weeks in iSREC and 20 weeks in WLC).  1) tobacco use behaviour (CPD, SREC use), 2) biomarkers (e.g. carbon monoxide, anabasine), 3) cigarette dependence and withdrawal, and 4) short-term health effects and tolerability (e.g. respiratory symptoms, substance use)  Change in cigarettes smoked per day (CPD) between randomized groups. Change in mean number of CPD in the past 7 days from baseline 2 to week 8 comparing between randomized groups (iSREC group vs WLC)  EC use during EC provision during the 8 wks of EC provision  Change in expired air carbon monoxide (CO) during EC provision. Change in expired air CO (ppm) from baseline to week 8 between randomized groups  Change in anabasine - during EC provision. Change in urine anabasine level (ng/mL)from baseline 1 to week 8 between randomized groups |
| Starting date | Estimated study start date: August 2023. Estimated completion date: December 2024 |
| Contact information | Joanna M Streck, PhD, 617-643-9977, jstreck@mgh.harvard.edu |
| Notes | New to 2023 update |

NCT05887947

| Study name | Impact of e-cigarette nicotine concentration on compensation, cigarette smoking, and biomarkers of exposure and harm in diverse smokers |
| Methods | Design: randomized cross-over  Setting: University of Kansas Medical Center, USA |
| Participants | Estimated enrolment: 48  Inclusion criteria: identify as non-Hispanic white or non-Hispanic African-American/black; willing to switch from CC to EC for 6 wks; smoke greater than or equal to 25 of the last 30 days for the past 3 months; not previously used an EC for > 30 days; exhaled CO of ≥ 6ppm at screener visit; willing to abstain from marijuana for 12 hours prior to in-person lab visits; willing to abstain from smoking and vaping for 12 hours prior to 3 in-person lab visits  Exclusion criteria: weekly use of EC over the last 6 months; use of tobacco products other than CC on ≥ 10 days in the past 30 days; use of EC on > 5 of the past 30 days; current use of cessation medications; pregnant/breastfeeding; past 30-day hospitalization/ER visit for psychiatric issue, seizure, stroke, or new heart problem; recent history of cardiovascular or pulmonary events in the past 3 months; treatment for alcohol or drug dependence in the past yr; current enrolment in a programme aimed at changing smoking patterns  Motivated to quit: not clear but excluded if using current cessation medication  EC use at baseline: no |
| Interventions | EC: Pod. Electronic cigarette nicotine concentrations 1.8% and 5%  Participants will complete 2 standardized, 10-puff vaping bouts over 5 mins followed by a 60-minute ad libitum vaping session, using 2 e-liquids that differ only by nicotine concentration (5% vs 1.8%) to examine the effect of nicotine concentration on in-lab compensatory puffing, nicotine exposure, and e-liquid consumption. In Phase 2, the same participants will be randomized to 5% or 1.8% nicotine e-liquid and instructed to switch completely for 6 weeks.  African-American  EC Nicotine Concentration Order: 1.8%, 5%, 1.8%  EC Nicotine Concentration Order: 1.8%, 5%, 5%  EC Nicotine Concentration Order: 5%, 1.8%, 1.8%  EC Nicotine Concentration Order: 5%, 1.8%, 5%  White  EC Nicotine Concentration Order: 1.8%, 5%, 1.8%  EC Nicotine Concentration Order: 1.8%, 5%, 5%  EC Nicotine Concentration Order: 5%, 1.8%, 1.8%  EC Nicotine Concentration Order: 5%, 1.8%, 5% |
| Outcomes | Baseline lab visit 1, lab visit 2. Phase 2: 6 weeks home use  Total inhaled volume. Time frame: 2 to 10 days between visits. Differences within participants in total inhaled volume in electronic cigarette puff topography during the pharmacokinetic portions of lab visit 1 and 2  Examine the impact of nicotine concentration on short-term, real-world EC use patterns and related biomarkers of exposure (e.g. exhaled carbon monoxide, 4-(methylnitrosamino)-1-(3-pyridyl)-1-butanol (NNAL), lung inflammatory markers) |
| Starting date | Starting date: 20 February 2023. Estimated primary completion date: 20 February 2025 |
| Contact information | Leah Lambart, MPH, 913-945-7862, llambart@kumc.edu  Eleanor Leavens, PhD, 913-588-3763, eleavens@kumc.edu |
| Notes | New to 2023 update |

NCT05960305

| Study name | A multi-site randomized actual use study of electronic nicotine delivery system (P12) products among current U.S. adult smokers to assess the relative impact of availability and use of different e-liquid flavors on changes in cigarette consumption  Brief title: CSD201204 An actual use study of P12 electronic nicotine delivery system among U.S. adult amokers |
| Methods | Multi-site, open-label, randomized, 3-arm, 8-week, prospective observational study  Setting USA |
| Participants | 1845 participants enrolled  Inclusion criteria: 21 to 60 years of age, inclusive, who are regular smokers (≥ 5 cigarettes/day on at least 20 of the past 30 days), indicate "an intention to use" (6 or higher on a 10-point Likert scale) for at least one Study IP flavour per arm across all 3 study arms.  Exclusion criteria: currently quitting or intending to quit within the next 3 months all tobacco or nicotine product use ("currently" is defined as within [≤] 30 days prior to pre-screening); regular ENDS user (using ENDS > 3 days per week, in the past 30 days), based on self-report; pregnant or breastfeeding; "poor" physical health; "poor" mental health; employees of a company that manufactures tobacco or ENDS products. |
| Interventions | Active comparator:  1. Tobacco flavour  2. Menthol flavour  3. NTNM flavour  For all can choose between 2 different flavour variants at 2 different nicotine levels (1.5% and 5%). 4 options per arm.  Real-life/naturalistic environments. Subjects will be able to choose freely among the Study IP available in 1 of the 3 study arms to which they are randomly assigned. The 3 study arms are organized by Study IP flavour categories: tobacco, menthol, and non-tobacco-non-menthol (NTNM). Subjects will self-report their ad libitum use of the Study IP as well as use of combustible cigarettes (CC) and any other tobacco- and nicotine-containing product (TNP) on a daily basis using an electronic diary (eDiary). |
| Outcomes | Baseline to 6 weeks  Number and proportion of subjects who reduce their cigarettes per day (CPD) (over 6 weeks). Number and proportion of subjects who reduce their cigarettes per day (CPD) consumption by at least 50% at Week 6 compared to baseline among all subjects who complete the study.  CPD mean per cent reduction over 6 weeks. CPD mean per cent reduction at Week 6 compared to baseline among all subjects who complete the study. |
| Starting date | Study start date: September 2023  Study completion 2024 |
| Contact information | RAI Services Company. Reynolds American. Tobacco Industry.  Study Director: Kristen Jordan, PhD  Contact information not provided in NCR record. |
| Notes | New to 2024 update |

NCT06063421

| Study name | Comparison of nicotine replacement therapy and electronic cigarettes for smoking cessation in Pakistan |
| Methods | RCT  Pragmatic, open-label, parallel design RCT  Setting: Pakistan |
| Participants | Inclusion criteria: 18 years or older; current smokers presenting to a cessation clinic expressing a desire to quit smoking; capacity to consent; can read and understand the instructions in Urdu and/or English and follow the study instructions and procedures.  Exclusion criteria: pregnant or breastfeeding ; using EC or NRT products; enrolled in another similar study; not willing to quit; have had a recent cardiovascular event like unstable angina, stroke or myocardial infarction in the past 2 weeks.  Motivated to quit |
| Interventions | EC vs NRT  EC: The intervention consists of the use of an EC device, Vaporesso Gen Air 40 Vape Kit (includes EC device, integrated battery, refillable tank, charging cable, coil replacements and user manual). Three e-liquid flavours will be provided; tobacco, menthol, and fruit flavour. All e-liquids will have a nicotine concentration of 18 to 20 mg/mL. EC will be provided for a total of 12 weeks.  NRT: NRT combination therapy with a transdermal nicotine patch (7, 14, or 21 mg) and an oral product: either gum or lozenge in 1, 2, or 4 mg strength. Usage will be in the form of a daily nicotine patch and ad libitum use of the faster-acting lozenge to curb nicotine cravings. Participants will be provided with a 12-week supply of NRT. |
| Outcomes | Baseline, 1, 4, 8, 24, and 52 weeks  Carbon monoxide (CO) validated sustained abstinence at 24, 52 weeks  Self-reported 7-day point prevalence abstinence at 4, 24, and 52 weeks  AEs, CPD, and product satisfaction rating, 1, 4, 8, 24, and 52 weeks post TQD  Intervention cost per participant (12 wks from TQD) |
| Starting date | Study posted October 2023. Updated March 2024. |
| Contact information | Fouad Aslam, MPH, 00447494700290, projectdirector@strategichealthresearch.org.uk  Aftab Ahmad, MA |
| Notes | New to 2024  Funding: Foundation for a Smoke Free World INC |

NCT06077240

| Study name | Effects of e-cigs vs pouches on cigarette smoking and addiction  Official title: Evaluating the effects of e-cigarettes versus oral nicotine pouches and product constituents (menthol flavor, nicotine concentration) on adult cigarette smoking and addiction |
| Methods | Randomised parallel assignment  Triple-masked (participant, investigator, outcomes assessor)  Setting: USA |
| Participants | Aim to recruit 256 adults who currently smoke CC and willing to switch  Inclusion criteria: 21+ years old; English literate; currently smoking cigarettes, biochemically confirmed; not planning a smoking cessation attempt or to use smoking cessation pharmacotherapies (NRT, bupropion, varenicline) in the next month  Exclusion criteria: currently using any smoking cessation services and/or pharmacotherapies; pregnant or breastfeeding; significant current medical or psychiatric condition; known hypersensitivity to propylene glycol |
| Interventions | EC 2.4% nicotine, menthol and tobacco flavours  EC 2.4% nicotine with tobacco flavour only  EC 5% nicotine with menthol and tobacco flavours  EC 5% nicotine with tobacco flavour only  Tobacco pouches 3 mg nicotine with menthol and tobacco flavours  Tobacco pouches with 3 mg nicotine with tobacco flavour only  Tobacco pouches with 6 mg nicotine with menthol and tobacco flavours  Tobacco pouches with 6 mg nicotine with tobacco flavour only |
| Outcomes | Baseline, 4, 6 weeks; 5 visits  Plan to share IPD  Abstinence, biochemically verified 7-day point-prevalent abstinence from cigarettes (to week 4)  CPD  Cigarette dependence measured using the 4-item PROMIS® Short Form v1.0 - Smoking Nicotine Dependence for All Smokers 4a. Each item is scored from 1 to 5 with the range of scores from 4 to 20 with higher scores representing greater cigarette dependence.  Use of non-combustible product.  Continued use of study product (to 6 weeks). |
| Starting date | First posted Oct 2023. Last update posted February 2024.  Starting date not stated. |
| Contact information | Lisa M. Fucito, lisa.fucito@yale.edu  Krysten W Bold, krysten.bold@yale.edu |
| Notes | New to 2024  Funding: National Institute on Drug Abuse (NIDA) |

NCT06111053

| Study name | Trial for harm reduction with incentives and vaping e-cigarettes  Official title: Harm reduction in smokers with obesity: impact of contingent incentives and e-cigarettes |
| Methods | Randomised, 2x2 factorial design  Setting: USA |
| Participants | Actual enrollment: 39. Estimated enrolment 36  Inclusion criteria: BMI ≥ 25 kg/m2; smoked ≥ 5 cigarettes/day during the past year; 21 or older; exhaled CO of > 6 ppm at baseline; willing to use ENDs for 6 weeks; daily access to a Bluetooth-enabled smartphone/tablet  Exclusion criteria: planning to set a smoking quit date in the next 30 days; receiving smoking cessation treatment of any kind in the past 30 days; using EC/ ENDS > 4 days per month; hospitalized for mental illness in past 30 days; heart-related event (e.g. heart attack, severe angina) in past 30 days; resides with another person enrolled in the study; pregnant, nursing, or planning to become pregnant in the next 6 months |
| Interventions | Interventions:  EC/ENDS: participants in active comparator groups that include ENDS will receive 6 weeks' worth of ENDS supplies.  Contingent incentives: participants in active comparator groups will receive incentives that vary based on participant abstinence from smoking, measured by a carbon monoxide breath sample.  No EC /ENDS: participants in active comparator groups labelled No ENDS will only receive information about the comparative risk of ENDS relative to combustible cigarettes.  Non-contingent incentives. Participants in active comparator groups labelled Non-Contingent Incentives will receive compensation for each breath sample provided throughout the study, with no variation.  Study arms:  1. Experimental: EC and Contingent Incentives  Participants in this arm will receive information about the comparative risk of EC relative to smoking as well as 6 weeks' worth of provisions of EC and will receive 4 weeks of monetary incentives for complete abstinence from smoking (after a controlled ramp down of smoking).  2. Experimental: No EC and Contingent Incentives  Participants in this arm will receive information about the comparative risk of EC relative to smoking and will receive 4 weeks of monetary incentives for complete abstinence from smoking (after a controlled ramp down of smoking).  3. Experimental: EC and Non-Contingent Incentives  Participants in this arm will receive information about the comparative risk of EC relative to smoking as well as 6 weeks' worth of provisions of EC and will receive monetary incentives for providing breath samples only (non-contingent on smoking status).  3. Experimental: No EC and Non-Contingent Incentives  Participants in this arm will receive information about the comparative risk of EC relative to smoking and will receive monetary incentives for providing breath samples only (non-contingent on smoking status). |
| Outcomes | Baseline 4, 6, and 12 weeks  At 4, 6, and 12 weeks  Use of EC and CC; abstinence; CO; CC and EC dependence; questionnaire comparing CC and EC; weight assessed; motivation to change; attitudes to EC using Comparing E-Cigarette and Cigarettes (CEAC) Questionnaire.  IPD: plan to share data |
| Starting date | October 2023  Estimated completion date: December 2024 |
| Contact information | Cara M Murphy, 1 (401) 203-5339, THRIVE@brown.edu |
| Notes | New to 2024 |

NCT06118502

| Study name | A clinical trial of adaptive treatment for early smoking cessation relapse (ADAPT) |
| Methods | Randomised sequential assignment, open-label  Setting: Alabama and South Carolina, USA. |
| Participants | Estimated enrolment 544  Treatment-seeking people who smoke |
| Interventions | NRT (patches and lozenges); varenicline. At 8 weeks some non-responders will be offered EC.  Treatments free for 12 weeks  Switching to a different medication: 4 weeks of the other FDA-approved option, either varenicline or combination NRT, with instructions to try to quit again at week 4.  Continued use of the same medication: 4 additional weeks of the same medication (varenicline or NRT) with instructions to try to quit again at week 4.  Switching to a harm-reduction tobacco product: 4 weeks of e-cigarette products with instructions to switch completely at Week 8.  Study arms  1. Adaptive Randomization 1 (switching to another medication): people who did not respond to 4 weeks of pharmacotherapy (either varenicline or combination NRT). After a 4-week course of pharmacotherapy, participants that are not responding to medication will receive 4 weeks of the other FDA-approved option, either varenicline or combination NRT, with instructions to try to quit again.  2. Non-Adaptive Randomization 1 (continued use of the same medication) : people who did not respond to 4 weeks of pharmacotherapy (either varenicline or combination NRT). After a 4-week course of pharmacotherapy, participants that are not responding to the medication will receive 4 additional weeks of the same medication with instructions to try to quit again.  3. Harm Reduction Randomization 2 (switching to a harm-reduction tobacco product): people who did not respond to two 4-week courses of pharmacotherapy (either varenicline or combination NRT or both). After two 4-week courses of pharmacotherapy, participants who are not responding to medication will be randomly assigned to a harm-reduction group (e-cigarettes). Participants assigned to the harm-reduction group will receive 4 weeks of e-cigarette product with instructions to switch completely.  4. Non-Adaptive Randomization 2 (continued use of the same medication): people who did not respond to two 4-week courses of pharmacotherapy (either varenicline or combination NRT or both sequentially). After two 4-week courses of pharmacotherapy, participants that are not responding to the medication will receive 4 additional weeks of the same medication with instructions to try to quit again. |
| Outcomes | Baseline, 4, 8, 12, 24 weeks. 8 surveys, all assessments remote.  Abstinence from CC. 7-day point prevalence abstinence. |
| Starting date | Posted November 2023 |
| Contact information | Tracy T Smith, 8438725164, smithtra@musc.edu  Matthew J Carpenter, 8438762436, carpente@musc.edu |
| Notes | New to 2024 |

NCT06169813

| Study name | E-cigarette harm reduction among PLWHA in South Africa |
| Methods | RCT  Open-label, parallel assignment  Setting: South Africa |
| Participants | Estimated N = 90  Inclusion criteria: adult PLWHA CC smokers; speaks Afrikaans, or Xhosa, or English; daily CC smoking (≥ 5 CPD); mobile phone; interested in reducing CC smoking but not necessarily trying to quit; receives HIV/AIDS care in one of the 8 selected clinics follow-up rates  Exclusion criteria: pregnant or breastfeeding; unable to provide consent; used tobacco products other than CC in the past 2 weeks (e.g. EC, cigarillo); currently engaged in an attempt to quit CC smoking; current major depressive or manic episode, current psychotic disorder, past-year suicide attempt, or current suicidal ideation with plan or intent |
| Interventions | EC: EC VUSE "Solo" single-use pods. Nicotine - 48 mg/mL (4.8% nicotine) concentration + phone counselling + EMI texting  NRT: NRT (daily patches and lozenges). NRT strength will be according to the established dosing guidelines for tobacco treatment. NRT is the standard of care in tobacco treatment and helped reduce CPD in prior trials + phone counselling + ecological momentary intervention (EMI) texting  Quit Line: participants will receive referral to the existing South African Quitline. Participants will receive information to contact the Quitline if participants so choose.  All groups receive counselling. Each participant will receive up to 5 motivational counselling sessions. The first session will also include orientation of EMA/EMI texting. |
| Outcomes | Baseline, 8 weeks, 3 months, 6 months  7-day point prevalence abstinence at 3 months. Abstinence will be verified by exhaled carbon monoxide and defined as no combustible cigarette use in the last 7 days  50% reduction in cigarettes per day (CPD), compared with baseline at 6 months, CPD will be self-reported  Change in American Thoracic Society Questionnaire score  Client Satisfaction Questionnaire (CSQ-8) score at 8 weeks and 3 months  Percent of patients who enrol in counselling at 6 months  FU rate at 3 and 6 months |
| Starting date | Estimated start date: Feb 2024  Estimated completion date: June 2024 |
| Contact information | Omar ElShahawy  Omar.ElShahawy@nyulangone.org  NYU Langone Health |
| Notes | New to 2024 |

NCT06260683

| Study name | A comprehensive evaluation of tobacco-flavored vs. non-tobacco flavored e-cigarettes on smoking behavior |
| Methods | RCT  Setting: Ohio State University Comprehensive Cancer Center, USA. |
| Participants | Estimated N = 1500  Inclusion Criteria: >= 21 years old; Smoke >= 5 cigarettes per day for the past year; Willing to use EC /NRT; fluent English; smartphone.  Exclusion Criteria: using smoking cessation medications/NRT/seeking treatment for smoking cessation; use EC > 4 days a month; lung disease, asthma, cystic fibrosis, heart disease or chronic obstructive pulmonary disease (COPD); unmanaged schizophrenia; past 3 month cardiac event or distress or stroke; pregnant/breastfeeding; uncontrolled high BP; serious angina pectoris or chest pain; allergy to propylene glycol or vegetable glycerin; serious underlying arrhythmias, irregular heartbeat or abnormal heart rhythm.  EC use at baseline: No (exclusion criteria: use EC > 4 days a month)  Motivated to quit smoking: willing to use EC/NRT although not currently seeking help to stop smoking. |
| Interventions | ARM I: preferred flavoured EC  ARM II: tobacco flavoured EC  ARM III: NRT (nicotine patches and lozenges)  All receive for 14 weeks, including a 2-week pre-switch period to become familiar with usage. All given questionnaire. Participants in all arms participate in discussions throughout the trial. |
| Outcomes | Baseline, 2, 6, 14 and 26 weeks  Intervention for 14 weeks. Participants in all arms are followed for 12-weeks after completion of study procedures.  CO week 14; CPD baseline to 26 weeks; Continued use of EC (14 weeks and 26 weeks). [Abstinence to week 14.] |
| Starting date | Start date: 10 April 2024. Estimated completion date: 12 April 2028. |
| Contact information | Theodore Wagener, Ohio State University Comprehensive Cancer Center. Theodore.Wagener@osumc.edu |
| Notes | Ongoing study new to 2025 update. |

NCT06264154

| Study name | The role of flavor in the substitutability of e-cigarettes for combustible cigarettes among persistent smokers |
| Methods | RCT  Between-subjects study  Setting: University of Pennsylvania, Philadelphia, USA |
| Participants | Estimated N 210    Inclusion: > 21 years of age and self-report smoking at least 5 cigarettes (menthol and/or non-menthol) per day for at least the last 12 months; 5 or more failed quit attempts and the use of smoking cessation medication on at least one prior attempt; Ever use of an e-cigarette; CO > 10 ppm; Not using any forms of nicotine regularly other than CC; willing to switch to EC for 6 weeks and use the assigned flavors.  Exclusion: Regular use of nicotine-containing products other than CC (e.g., chewing tobacco, snuff, snus, cigars, EC, etc.); enrollment in a smoking cessation program over the duration of the study; current use of smoking cessation medication; history of substance abuse (other than nicotine dependence) in the past 12 months; pregnant/breastfeeding; serious or unstable disease within the past year (e.g. cancer, heart disease); lifetime history of schizophrenia or psychosis.  EC use at baseline: no  Motivated to quit: yes (have tried to quit on > 5 occasions (inclusion criteria).) |
| Interventions | All participants are instructed to switch from smoking combustible cigarettes to using e-cigarettes for 6 weeks. Participants will receive an e-cigarette device and flavored nicotine pods according to their randomly assigned flavor.  1) EC fruit flavour (blueberry or watermelon-flavored pods)  2) EC tobacco flavour  3) EC menthol flavoured  All participants provided with EC and instructed to switch from smoking CC to using only the study provided nicotine EC pods. Participants will receive their supply of nicotine pods in 7-day increments, based on baseline smoking behaviour. |
| Outcomes | Time Frame: 42 days (days 8 - 49). 6 months |
| Starting date | Start date 26 August 2024. Estimated study completion date December 2027. |
| Contact information | Janet Audrain-McGovern, audrain@pennmedicine.upenn.edu |
| Notes | New ongoing study added to 2025 update. |

NCT06372899

| Study name | Noncombustible nicotine delivery systems as potential harm reduction tools for persistent cigarette smokers. Official title: Alternative nicotine delivery systems as potential harm reduction tools for persistent cigarette smokers |
| Methods | RCT  Setting: University of Pennsylvania, Philadelphia, USA |
| Participants | Estimated N: 200  Inclusion: 21+; 5 or more failed quit attempts and the use of smoking cessation medication on at least one prior attempts; CO) greater than 10 ppm; not using any forms of nicotine regularly other than cigarettes; willing to switch to e-cigarettes or nicotine pouches for 6 weeks.  Exclusion: history of substance abuse (other than nicotine dependence) in the past 12 months; pregnant/breastfeeding; serious or unstable disease within the past year (e.g. cancer, heart disease); lifetime history of schizophrenia or psychosis.  EC use at baseline: no  Motivated to quit: yes, have tried to quit on > 5 occasions (inclusion criteria). |
| Interventions | 1) EC. Tobacco, menthol, watermelon, and blueberry flavored nicotine pods.  2) Oral nicotine pouches. Original, mint, berry, and citrus flavored nicotine pouches.  Instructed to use study product for 6 weeks. Receive supply of study product in 7-day increments, based on baseline smoking behavior. |
| Outcomes | Baseline, 49 days, 6 months.  CPD baseline to the end of the six-week switch period and 6 months.  Changes in biomarkers of potential harm, assessed at baseline and the end of the six-week switch phase. CO, FEF, NNAL, 1-HOP. |
| Starting date | Starting date 2 October 2024. Estimated study completion date 31 March 2028. |
| Contact information | Janet Audrain-McGovern, Professor, University of Pennsylvania. audrain@pennmedicine.upenn.edu  Collaborator: National Cancer Institute. |
| Notes | New ongoing study added to 2025 update. |

NCT06373679

| Study name | Switch or quit R01. Official title: Non-cigarette tobacco products as harm reduction tools in smokers who failed to quit with traditional methods |
| Methods | RCT  Setting: Medical University of South Carolina |
| Participants | Estimated N 225  Inclusion Criteria: adults 21+ who previously had a quit attempt using FDA-approved pharmacotherapy.; interest in reducing harms from tobacco use or quitting smoking  Exclusion Criteria: pregnant / breastfeeding.  Motivated to quit: yes, all have previously tried to quit. |
| Interventions | 1) EC. Participants will choose between two different brands of EC and choose up to two different flavors. Participants will receive 11 weeks of EC products with instructions to switch completely at switch date.  2) Medication. Participants will choose between 1) combo NRT and 2) varenicline. The NRT will consist of transdermal NRT and nicotine lozenge. Participants will receive 11 weeks of FDA approved medication with instructions to quit smoking cigarettes at quit date. |
| Outcomes | Baseline, 11 weeks  Abstinence: Self-reported zero cigarettes in the past 7 days on timeline followback at Week 11 + expired CO < 6 ppm. CO for all? > 50% reduction in cigarette smoking. |
| Starting date | Starting date: 10 July 2024. Estimated completion date 1 May 2027. |
| Contact information | Tracy Smith, smithtra@musc.edu. Merritt McDonald mcdoname@musc.edu |
| Notes | New ongoing study added to 2025 update. |

NCT06534905

| Study name | Pilot randomized controlled trial of e-cigarette switching among older adults with opioid use disorder. Short title: E-cigarette switching older adults. |
| Methods | RCT  Setting: University of Maryland Addiction Treatment Center, Baltimore, Maryland, USA |
| Participants | Esimated N: 40  Inclusion Criteria: 50 years or older; currently in treatment for opioid use disorder for at least 3 months; currently use CC; expired air CO 8ppm; does not regularly use EC (regular use defined as use in the past month for 2 or more consecutive days); not pregnant or breastfeeding.  Exclusion Criteria: trying to stop smoking or have a plan to quit smoking; age 49 or younger; not currently using CC.  Not motivated to quit smoking.  Not regularly using EC.  Inclusion based specific population characteristic: 50 years + and in treatment for opioid use disorder . |
| Interventions | 1) EC (NJOY Ace, menthol or tobacco flavour depending on patient preference) + education on tobacco harms  2) Control standard advice. Brief advice to quit smoking (in alignment with recommendations by the American Society of Addiction Medicine). Inclusdes linking to a smoking cessation quitline. |
| Outcomes | Baseline, 2, 6, 8 weeks  CPD (self-report), intention to quit, assessments of tobacco and other substance use, health status, mood, and functioning. |
| Starting date | Start date 25 November 2024. Estimated completion December 2025. |
| Contact information | Bethea A Kleykamp, University of Maryland, Baltimore |
| Notes | New to 2025 update. |

NCT06543407

| Study name | Harm reduction for smokers with mental illness: RCT of e-cigarette provision with or without behavioral support to boost switching |
| Methods | RCT  Setting: 2 locations: Louisville, Kentucky USA , and Kalamazoo, Michigan USA  Sponsor: Dartmouth-Hitchcock Medical Center, New Hampshire, USA |
| Participants | Estimated N: 250  Inclusion: Diagnostic Criteria (must have one to be eligible): Schizophrenia; Bipolar disorder; Major Depressive Disorders; Posttraumatic disorder; Other anxiety disorders. Additional Inclusion Criteria:21 years +; CC user (at least 10 cigarettes/day); At least one quit attempt in the past 5 years using evidence- based pharmacotherapy or behavioral cessation support;Not currently interested in quitting.  Exclusion: Currently residing in a nursing home; Asthma; Cognitive impairment (score <26 on the Telephone Interview for Cognitive Status (TICS); current EC use (>once a week); Psychiatric instability (hospitalized in the past month); Current AND moderate to severe substance use disorder; Pregnant; Use of any smoked products other than cigarettes; Current unstable medical illness making EC unsafe (e.g., recent heart attack, cancer);  Motivated to quit: NR but have tried to quit at least once in past 5 years.  No EC use at baseline.  Inclusion based on specific population characteristics: people with mental illness |
| Interventions | 1) EC NJOY for the first 8 weeks  2) EC NJOY + behavioral support and coaching, protocolized intervention, SWITCH IT, for the first 8 weeks of the study.  Behavioral support for switching, 7-10 sessions with SWITCH IT coach delivered during the first 8 weeks of the study. SWITCH IT participants will also have the opportunity to receive supported "field trips" to explore EC options based on availability, cost, and preferences during week 4 and week 6. |
| Outcomes | Baseline to 8 weeks, 8 weeks to 26 weeeks  NNAL, CO,  Baseline to 8 weeks 8 weeks to 16 weeks  CC use (self-reported). |
| Starting date | Start date 1 October 2024. Estiated completion date 31 March 2028. |
| Contact information | Meghan M. Santos, MSW, meghan.m.santos@hitchcock.org  Gail Williams, MS, MFT, gail.williams@dartmouth.edu  Sarah Pratt, Dartmouth-Hitchcock Medical Center |
| Notes | New to 2025 update. |

NCT06554873

| Study name | Adaptive use of nicotine substitution to maintain smoking reduction/abstinence in nicotine responders |
| Methods | RCT  Setting: 2 locations: Charlotte, North Carolina, USA and Raleigh, North Carolina, USA.  Rose Research Center, LLC |
| Participants | Estimated N: 150  Inclusion Criteria: Healthy, adult CC user for ≥12 months ; CC at least 10 cpd; Screening eCO ≥ 10 ppm; aged 22 to 65 years.  Exclusion Criteria: unable to understand English; history or presence of clinically significant medical or psychiatric disease; Has used nicotine EC or any NRT (nicotine patch, nicotine gum, nicotine spray, nicotine inhaler, nicotine lozenge) or prescription smoking cessation medications, including, varenicline (Chantix\*) or bupropion (Zyban®) within the past 30 days; Pregnant/nursing; Participated in smoking cessation study in past year; Smokes or vapes cannabis >once a week; Cannabis Use Disorder Identification Test-Revised (CUDIT-R) score of 8 or greater.  No EC use at baseline.  Motivated to quit: NR |
| Interventions | To determine whether smokers who initially respond (within 2 weeks) to nicotine products (including NRT, EC, nicotine pouches) by reducing their smoking by ≥50% can be successfully maintained on use of these noncombustible nicotine alternatives to cigarettes for 6 months, and whether this results in sustained smoking reduction/abstinence.  Interventions: All groups offered: Nicoderm; Nicorette 4Mg Chewing Gum; Nicorette Lozenge Product; EC NJOY; on!  1) Nicotine Non-Responders  Participants that were not successful in reducing their expired carbon monoxide by the end of week 2 will not continue in the study.  2) Nicotine Responders - Group 1  Participants that were successful in reducing their expired carbon monoxide by the end of week 2 will be randomized to continued use of their choice of nicotine products for an additional 10 weeks (12-week total treatment period).  3) Nicotine Responders - Group 2  Participants that were successful in reducing their expired carbon monoxide by the end of week 2 will be randomized to continued use of their choice of nicotine products for an additional 22 weeks (24-week total treatment period). |
| Outcomes | Baseline, week 12, 24 , 36.  CC abstinence (CO confirmed) to week 24.  CO to week 24.  Study product use (to week 36). Cotinine to week 36. |
| Starting date | Start date 29 August 2024. Estimated completion date 31 December 2025 |
| Contact information | Derek Mercedes, derek.mercedes@roseresearchcenter.com  Sponsor: Rose Research Center, LLC. Collaborator: Global Action to End Smoking |
| Notes | New to 2025 update. |

NCT06614504

| Study name | Nicotine regulation for dual users of e-cigarettes and cigarettes (RDEC) |
| Methods | 2x2 factorial randomized controlled trial  Setting: 2 sites. Providence Rhode Island USA. Burlington, Vermont, USA. |
| Participants | Estimated N 308  Inclusion: regular use of tobacco; 21 year of age or older.  Exclusion: Pregnant / nursing; health conditions that could undermine ability to complete the study.  Inclusion based on specific population characteristics: dual users of CC and EC |
| Interventions | Varying the nicotine content of CC and varying the nicotine content of EC.  1) CC #1 plus EC #1 (Normal nicotine CC plus high nicotine content EC)  2) CC #1 plus EC #2 (Normal nicotine CC plus low nicotine content EC)  3) CC #2 plus EC #1 (Very low nicotine content CC plus high nicotine content EC)  4) CC #2 + EC #2 (Very low nicotine content CC plus low nicotine content EC) |
| Outcomes | Baseline, 12 weeks.  CPD. |
| Starting date | Start date 1 November 2024. Estimated completion date 30 September 2028 |
| Contact information | Emily Booth, emily.booth@med.uvm.edu. PI Elias Klemperer. |
| Notes | New ongoing study added to 2025 update. |

Polosa 2024

| Study name | Magnitude of cigarette substitution after initiation of e-cigarettes and its impact on biomarkers of exposure and potential harm in dual users: MAGNIFICAT trial |
| Methods | Two parallel study groups. Not randomised.  Setting: ambulatory (outpatient) setting. University of Catania, Italy  Recruitment: from the clinical study site subject pool and public advertisements. |
| Participants | Estimated N 300  EC group (express interest inquitting) 250. CC group (not interested in quitting) 50  Inclusion criteria: ≥ 19 years of age b. Solus smokers of CC (≥ 15 cigarettes/day); History of regular smoking for at least 12 consecutive months; Verified smoking status (eCO ≥ 7 ppm); Willingness to switch to a vaping product and to try reducing CCconsumption (Study Group A only)  Exclusion criteria: Intention to quit smoking within the next 30 days ; Known clinically significant cardiovascular, respiratory, psychiatric, or other major disorder ; Regular use of any medication; A significant history of alcohol or drug abuse; Use of any nicotine (e.g., EC, nicotine pouches) or tobacco product (e.g., heated tobacco products - HTPs, oral smokeless) other than their own CC within 3 months of screening; Use of nicotine replacement therapy or other smoking cessation therapies within 3 months of screening g. Pregnant / breast feeding; Active participation in another clinical trial.  Motivated to quit smoking: no (exclusion criteria intending to quit within next 30 dats).  EC use at baseline: no. |
| Interventions | 1) EC  2) Continued CC use |
| Outcomes | Baseline, 1 month, 3 months, 6 months.  Day −7 to Day −1: Screening Visit . Day 0: Baseline Visit (Visit 1). Day 30: Visit 2. Day 90: Visit 3. Day 180: Visit 4.  SAEs; AEs; CC use; CO; weight; BP; lung function.  Biomarkers: Acrolein (3-HPMA) • 1,3-Butadiene (MHBMA) • Propylene Oxide (2-HPMA) • Crotonaldehyde (HMPMA) • Benzene (SPMA) • Styrene (PHEMA) • Glycidol (DHPMA) • Isoprene (IPMA) • Toluene (SBMA) • Ethylene Oxide (HEMA) • Acrylonitrile (CEMA/CeVal) • Acrylamide (AAMA/GAMA/GlyVal) • Metabolites of polyaromatic hydrocarbons (benzo[a]pyrene, pyrene, phenanthrene, naphthalene) • Aromatic amines (3−/4-aminobiphenyl, 2-aminonaphthalene, ortho-toluidine) • total nicotine equivalents • (Methylnitrosamino)-1-(3-pyridyl)-1-butanol (NNAL) • N-nitrosonornicotine (NNN) • Propylene glycol; Eicosanoids in urine, • soluble intercellular adhesion molecule 1 (sICAM-1) in plasma, • growth differentiation factor 15 (GDF-15) in plasma.  Cardio-respiratory endpoints: VO2 max/Chester step test; Spirometry; Respiratory Symptoms Questionnaire (RSQ).  Non-targeted methods for the screening of the urinary exposome, the hemoglobin adductome and the breathome (exhaled breath )  Correlation of use behavior with exposure via BoE and BoPH in urine, plasma and exhaled breathin compliant subjects (biochemically verified with biomarkers of compliance)  Urinary creatinine for normalization of urinary biomarker concentrations.  EC (study product) use at all time points. Level of dual use (EC and CC) |
| Starting date | Start date February 2024. Estimated completion date early 2025. |
| Contact information | Riccardo Polosa polosa@unict.it Jakub Weglarz jakub.weglarz@eclatrbc.it |
| Notes | New ongoing study added to 2025 update.  Study funded through grant from the Foundation for a Smoke-Free World. |

Schiek 2024

| Study name | Combining app-based behavioral therapy with electronic cigarettes for smoking cessation: A study protocol for a single-arm mixed-methods pilot trial |
| Methods | Single arm  Setting: Faculty of Health/School of Medicine, Witten/Herdecke University, Germany  Recruitment: via web-based advertisements, flyers, and a study website. |
| Participants | Estimated N 70  Inclusion criteria: aged 18–65 years, report having smoked at least 5 cigarettes per day (CPD) for at least 12 months, are motivated to stop smoking (Motivation To Stop Scale (MTSS; [51]) > 4 points), have daily access to their own smartphone (iOS 15/Android 11 or more recent).  Exclusion criteria include self-reported current or planned pregnancy, breastfeeding, a self-reported allergy to vegetable glycerin or propylene glycol, drug and/or alcohol dependence, severe psychiatric or physical illness, a disease or medication associated with a contraindication to the use of EC, and medication that could affect the outcomes of the study (bupropion/ nortriptyline/ varenicline/ cytisine/ clonidine/ antidepressants). Surgery (with anesthesia) in the last 6 weeks, participation in any other smoking cessation program, current use of EC/tobacco heaters/alternative tobacco products/NRT for more than 5 days during the last 30 days, and the inability to consent.  Motivated to quit smoking: yes.  Not using EC at baseline. |
| Interventions | Nicotine EC pods + access to mHealth intervention nuumi app for at least 3 months. EC coupled with the app via Bluetooth, allowing for tracking of patterns of use. The behavioral therapy leverages evidence-based content informed by cognitive behavioral therapy and mindfulness-informed principles.  Nuumi is a self-guided digital therapeutic intervention comprising an app-based behavioral therapy and an EC connected to the app via Bluetooth. Initially, participants are asked to use the EC whenever they crave a cigarette (replacing CC with EC). Prompted to use the app-based behavioral therapy providing information on transitioning to the EC, and smoking cessation. App also contains information on gradual EC cessation, supporting abstinence of both CC and EC.  Participants are not required to quit smoking immediately after baseline; advised to switch from CC to EC either by choosing a quit date, or by gradually switching from CC to EC over a 2-week period.  EC closed system device, pods must be replaced with prefilled pods obtained through the manufacturer which can only be used after activation via the app. Participants will receive a kit including the EC, a charger, a power bank, pods, and manuals for the EC and the pods via mail. The amount of pods is equivalent to their respective CC consumption at study entry. EC two tobacco flavors. EC powered by a 450 mAh battery, nicotine strength 20 mg/ml to 0 mg/ml, decreasing in steps of two mg/ml. Prompted to start with 20 mg/ml pods and gradually use pods containing lower nicotine strength.  Participants will receive 10€ for each completed survey (t1- t4). For participation in the semi-structured interviews, participants can earn another 10€ for each interview for a total incentive of 60€ once the study has ended. |
| Outcomes | Baseline, 4, 8 , 12 weeks, and 24 weeks  Abstinence (7 day pp) at 12 and 24 weeks. AEs. Other smoking cessation-related outcomes, psychological outcomes, and acceptability of the nuumi intervention |
| Starting date | Study start October 2023. |
| Contact information | Helen Schiek, Institute for Integrative Health Care and Health Promotion (IGVF), Faculty of Health/School of Medicine, Witten/Herdecke University, Witten, Germany. helen.schiek@uni-wh.de |
| Notes | New ongoing study added to 2025 update. The EC has been developed and manufactured by the funder of this study.  Funding statement: "Open Access funding enabled and organized by Projekt DEAL. IGVF at Witten/Herdecke University received funding to perform this trial from Sanos Group GmbH, the manufacturer of the nuumi program consisting of the electronic cigarette device, the pods including the liquid solution, and the smoking cessation app. Sanos Group GmbH is financially supported by the European Union’s Fund for Regional Development and Investitionsbank Berlin for its technological innovation and social impact by the funding programs "Pro FIT – Early Stage Financing" and “Pro FIT – Project Financing”. The funder’s responsibilities included initiating contact with interested participants and approval of the final study design. The institute’s research team’s responsibilities included participant screening, data collection, data management, data analyses, interpretation of results, and writing manuscripts. The study funder has no role in data collection, management, analysis, or interpretation of the data, and in writing, submitting, and publishing of any and all resulting scientific manuscripts. These responsibilities were approved by the IRB. Contact information of the funder: Sanos Group GmbH. Luetzowstrasse 102 10785 Berlin, Germany." |

Walker 2023

| Study name | Cytisine and e-cigarettes with supportive text-messaging for smoking cessation (Cess@Tion) |
| Methods | RCT  Setting: community  University of Auckland, New Zealand |
| Participants | Estimated enrolment: 800  Inclusion criteria: daily smokers who live in New Zealand; motivated to quit smoking within the next 2 weeks and willing to use cytisine or an EC or both products; ≥ 18 years  Exclusion criteria: another person in their household currently enrolled in the study; pregnancy/breastfeeding; using smoking cessation medication (including EC daily for the last month); hypersensitivity to cytisine or nicotine EC; health condition e.g. renal impairment; tuberculosis; myocardial infarction, stroke, or severe angina, high BP, seizures; strong preference to use or not to use cytisine and/or EC in their quit attempt  For a full list, see NCT record. |
| Interventions | EC: pod device. Nicotine strength: 30 mg/mL (3%). Flavour: tobacco. Brand name: UpOx  Cytisine  Arm 1: Monotherapy (cytisine only)  12 weeks of cytisine: Participants allocated cytisine will be instructed to follow the manufacturer's 25-day dosing regimen, then follow a maintenance dose of cytisine from day 26 to week 12. Participants will also receive 6 months of text-based smoking cessation support.  Cytisine. Brand name: Tabex. Standard dosing of:   - Days 1 to 3: 1 tablet (1.5 mg) every 2 hours through the waking day (6 tablets/day) - Days 4 to 12: 1 tablet every 2.5 hours (5 tablets/day). Quit smoking date is day 5 - Days 13 to 16: 1 tablet every three hours (4 tablets/day) - Days 17 to 20: 1 tablet every 4 to 5 hours (3 tablets/day) - Days 21 to 25: 1 tablet every six hours (2 tablets/day)   Followed by a maintenance dose of cystine from day 26 to week 12 (1 tablet every 6 hours: 2 tablets/day)  Arm 2: Monotherapy (nicotine EC only)  12 weeks of a nicotine EC. Participants will also receive 6 months of text-based smoking cessation support.  Arm 3: Combination therapy (cytisine plus a nicotine EC)  12 weeks of cytisine (as above) and 12 weeks of a nicotine EC. Participants will also receive 6 months of text-based smoking cessation support. |
| Outcomes | Baseline, 3, 6, and 12 months post-quit date  Primary outcome: proportion of participants with verified continuous smoking abstinence CO confirmed  Self-reported continuous smoking abstinence; self-reported 7-day point prevalence smoking abstinence; change from baseline in the number of cigarettes smoked per day; health-related quality of life; cystine compliance; use of allocated treatment by participants; frequency of EC use, number of pods used; treatment switching; dual use; AEs; number of text-based behavioural support messages received by participants; marginal cost per quitter |
| Starting date | Study start date: 6 May 2022. Estimated primary completion date: February 2024 |
| Contact information | Natalie Walker, PhD 64-9-923-9884, n.walker@auckland.ac.nz  Chris Bullen, PhD MBChB 64-9-923-4730, c.bullen@auckland.ac.nz |
| Notes | New to 2022 update |

## Footnotes

LC:8-iso-PGF2a: an isoprostane  
 1-OHP: 1-hydroxypyrene  
 ACS: acute coronary syndrome  
 AE: adverse event  
 AHR: airway hyperresponsiveness  
 AMI: acute myocardial infarction  
 AUD: alcohol use disorder  
 AUDIT: AUDIT-C checklist terminology for alcohol dependence  
 BMI: body mass index  
 BP: blood pressure  
 BUP: buprenorphine  
 CAL: clinical attachment loss  
 CAR: continuous abstinence rate  
 CAT: Computerized Adaptive Testing OR Computer-Aided Tomography  
 CC: combustible cigarette  
 CCQ: Clinical COPD Questionnaire  
 CEMA: 2‐cyanoethylmercapturic acid  
 C-F NDS: combustion-free nicotine delivery systems  
 CK-MB: creatine kinase, heart specific isoenzyme  
 CMHT: Community Mental Health Team  
 CO: carbon monoxide  
 COPD: chronic obstructive pulmonary disease  
 COVID: COVID-19, disease caused by SARS-CoV-2  
 cpd/CPD: cigarettes per day  
 CRF: cardiovascular risk factors  
 CT: computed tomography  
 CVD: cardiovascular disease  
 CXCL9: CSCL9 (chemokine ligand 9)  
 DESC: DESC refers to a supportive housing project (see NCT03962660)  
 DNA: deoxyribonucleic acid  
 DSM-IV/5: Diagnostic and Statistical Manual of Mental Disorders-IV/5  
 EC: electronic cigarette  
 eCO: expired carbon monoxide  
 ECG: electrocardiogram  
 ECwN: electronic cigarette with nicotine  
 ECwoN: electronic cigarette without nicotine  
 EMI: ecological momentary intervention  
 ENDS: electronic nicotine delivery system  
 EQ-5D-5L: EuroQol 5 Dimension 5 Level  
 ER: emergency room  
 FDA: Food and Drug Administration  
 FEF: forced expiratory flow  
 FeNO: fractional exhaled nitric oxide  
 FEV1: forced expiratory volume  
 FPL: federal poverty level  
 FSH: follicle-stimulating hormone  
 FTND: Fagerström Test for Nicotine Dependence  
 FU: follow-up  
 FVC: forced vital capacity  
 GIF: graphics interchange format  
 GP: General Practitioner (Dr)  
 HaRTS-TRENDS: (trial name) Harm reduction for tobacco smoking with  
 HbA1c: haemoglobin A1C, glycosylated haemoglobin  
 HBsAg: hepatitis B surface antigen  
 HCV: hepatitis C  
 HDL: high-density lipoprotein  
 HEMA: 2-hydroxyethylmercapturic acid  
 HIV: human immunodeficiency virus  
 HMPMA: 3‐hydroxy‐1‐methyl propylmercapturic acid  
 HPB: Health Promotion Board  
 HPMA: hydroxypropylmercapturic acid  
 hs-CRP: high-sensitivity C-reactive protein  
 HTP: hydroxytryptophan  
 ICD-10: International Classification of Diseases, Tenth Edition  
 ICF: International Classification of Functioning  
 IL-6: Interleukin 6  
 iSREC: immediate standardized research e-cigarette  
 LDCT: low-dose computed tomography  
 LHC: lung health check  
 mCEQ: modified Cigarette Evaluation Questionnaire  
 MDD: major depressive disorder  
 MetS: metabolic syndrome  
 MHRA: Medicines and Healthcare products Regulatory Agency  
 MI: myocardial infarction  
 MINI: mini International Neuropsychiatric Interview  
 MMP9: matrix metallopeptidase 9  
 mMRC: modified Medical Research Council  
 MNWS: Minnesota Nicotine Withdrawal scale   
 mPES: multi-Parameter Evidence Synthesis  
 MPSS: mood and physical symptoms scale  
 MTSS: Motivation To Stop Scale  
 MTWS-R: Minnesota Tobacco Withdrawal Scale-R (15 items)  
 NHS: National Health Service  
 NIDA: National Institute on Drug Abuse  
 NNC: non-nicotine cigarette  
 NCCN: National Comprehensive Cancer Network  
 NNAL: carcinogen found in tobacco smoke (4‐(methylnitrosamino)‐1‐(3‐pyridyl)‐1‐butanol)  
 NNN: N'-nitrosonornicotine  
 NRT: nicotine replacement therapy  
 OHQoL-UK: Oral Health Quality of Life assessment United Kingdom  
 OUD: opioid use disorder  
 PANSS: Mean Positive and Negative Syndrome Scale  
 PATH: Population Assessment of Tobacco and Health  
 PEF: peak expiratory flow  
 PG: propylene glycol  
 PGEM: a stable metabolite of prostaglandin E2 (biomarker of inflammation)   
 PHQ-9: Patient Health Questionnaire 9  
 PI: principal investigator  
 PK: pharmacokinetic  
 PneT: PheT phenanthrene tetraol  
 PP(A): point prevalence (abstinence)  
 PPD: pocket probing depths  
 PROMPT: Community-Based Participatory Tobacco Dependence Strategy (PROMPT Project)  
 PS[E]CDI: Penn State Electronic Cigarette Dependence Index (e-cigarette dependence measure)  
 QN: NHS quit now programme  
 QoL: quality of life  
 q-PADDA: primer anchored DNA damage detection assay  
 QSU-Brief: Questionnaire of Smoking Urges  
 QTC: QT interval (time it takes for the electrical system to fire an impulse through the ventricles and then recharge)  
 RA: research assistant  
 RC: research cigarettes  
 RCT: randomised controlled trial  
 REDCAP: Research Electronic Data Capture (web application for surveys)  
 SABA: short-acting β2-agonists  
 SAE: serious adverse event  
 SC: e-salivary cotinine  
 SCP: smoking cessation programme  
 SES: socioeconomic status  
 SMI: serious mental illness  
 S-PMA: S-phenylmercapturic acid  
 SpO2: oxygen saturation  
 SREC: standardized research e-cigarette  
 SRMH: self-rated mental health  
 SSS: stop-smoking services  
 T2DM: type 2 diabetes  
 TC: tobacco cigarette  
 THP: tobacco heating products  
 TLFB: timeline follow back  
 TMS: transcranial magnetic stimulation  
 TNE: total nicotine equivalents  
 TNF-a: tumour necrosis factor alpha  
 TQD: target quit date  
 UC: usual care  
 USB: universal serial bus  
 V: volts  
 VAR: varenicline  
 VBA: very brief advice  
 VLNC: very low nicotine content  
 VNP: vaporized nicotine products  
 VO2: oxygen consumption  
 WBC: white blood cell  
 WISDM-Brief: Wisconsin Inventory of Smoking Dependence Motives-Brief  
 wk: week  
 WLC: waiting-list control  
 YLST: Yorkshire Lung Screening Trial  
 yr: year

## References to studies

### ACTRN12619001787178 {published data only}

- ACTRN12619001787178. Project NEAT: nicotine as treatment for tobacco smoking following discharge from residential withdrawal services. www.who.int/trialsearch/Trial2.aspx?TrialID=ACTRN12619001787178 (first received 18 November 2019).
- Bonevski B, Trigg J, Rich J, Williams E, Baker AL, Walker N, et al. Perceptions of nicotine products for tobacco smoking cessation following discharge from alcohol and other drug residential withdrawal services: a qualitative analysis. In: SRNT (Society for Research on Nicotine and Tobacco) 29th Annual Meeting 2023 Mar 1-4; San Antonio (TX), USA. SYM13-2. 2023:17.
- Trigg J, Rich J, Williams E, Gartner CE, Guillaumier A, Bonevski B. Perspectives on limiting tobacco access and supporting access to nicotine vaping products among clients of residential drug and alcohol treatment services in Australia. Tobacco Control 2023;Online:1-7. [DOI: 10.1136/tc-2023-058094]

### ACTRN12621000148875 {published data only}

- ACTRN12621000148875. HARMONY: harm reduction for opiates, nicotine and you. www.who.int/trialsearch/Trial2.aspx?TrialID=ACTRN12621000148875 (first received 12 February 2021). [CENTRAL: www.cochranelibrary.com/central/doi/10.1002/central/CN-02240640/full]
- Bonevski B, Jackson M, Austin E, Lintzeris N, Ezard N, Gartner C et al. HARMONY (harm reduction for opiates, nicotine and you) trial: protocol of a randomised controlled trial of the effectiveness of vaporised nicotine products for tobacco smoking cessation amongst NSW opiate agonist treatment clients. MedRxiv: the Preprint Server for Health Sciences 2024. [DOI: 10.1101/2024.06.21.24309014]
- Kypri K, Austin E, Jackson M, Wright K, Shui A, Li A. Vaping to quit smoking: qualitative study of people receiving opioid agonist treatment. Drug and Alcohol Review 2025;44(1):254-66. [DOI: 10.1111/dar.13953]

### ACTRN12625000179437 {published data only}

- ACTRN12625000179437. Puff vs pill: break the habit study: effect of nicotine vaping products vs varenicline on smoking cessation among people experiencing social disadvantage. https://trialsearch.who.int/Trial2.aspx?TrialID=ACTRN12625000179437 2025 (first received 15 April 2025).

### Berlin 2019 {published data only}

- Berlin I, Dautzenberg B, Lehmann B, Palmyre J, Liégey E, De Rycke Y, et al. Randomised, placebo-controlled, double-blind, double-dummy, multicentre trial comparing electronic cigarettes with nicotine to varenicline and to electronic cigarettes without nicotine: the ECSMOKE trial protocol. BMJ Open 2019;9(5):e028832.
- NCT03630614. Randomized trial of electronic cigarettes with or without nicotine in smoking cessation (ECSMOKE). clinicaltrials.gov/ct2/show/NCT03630614 (first received 15 August 2018).

### Cox 2022 {published data only}

- \*Cox S, Bauld L, Brown R, Carlise M, Ford A, Hajek P. Evaluating the effectiveness of e-cigarettes compared with usual care for smoking cessation when offered to smokers at homeless centres: protocol for a multi-centre cluster-randomized controlled trial in Great Britain. Addiction (Abingdon, England) 2022;117(7):2096-107. [DOI: 10.1111/add.15851]
- ISRCTN18566874. E-cigarettes vs usual care for smoking cessation when offered at homeless centres. Isrctn.com/ISRCTN18566874 (accessed 8 September 2022) (first received 12 October 2021). [DOI: 10.1186/ISRCTN18566874]

### El-Khoury 2021 {published data only}

- \*El-Khoury F, El Aarbaoui T, Heron M, Hejblum G, Metadieu B, Le Faou AL, et al. Smoking cessation using preference-based tools among socially disadvantaged smokers: study protocol for a pragmatic, multicentre randomised controlled trial. BMJ Open 2021;11(6):e048859. [DOI: 10.1136/bmjopen-2021-048859]
- Heron M, Le Faou AL, Ibanez G, Metadieu B, Melchior M, El-Khoury L, et al. Smoking cessation using preference-based tools: a mixed method pilot study of a novel intervention among smokers with low socioeconomic position. Addiction Science & Clinical Practice 2021;16(1):43. [DOI: 10.1186/s13722-021-00254-6]
- NCT04654585. Preference-based tools for smoking cessation among disadvantaged smokers, a pragmatic randomised controlled trial [Sevrage tabagique à l'aide des outils dédiés selon la préférence, un essai randomisé contrôlé pragmatique]. clinicaltrials.gov/ct2/show/NCT04654585 (first received 4 December 2020).

### Hameed 2024 {published data only}

- Hameed A, Malik D. Clinical study protocol on electronic cigarettes and nicotine pouches for smoking cessation in Pakistan: a randomized controlled trial. Trials 2024;25(1):9. [DOI: 10.1136/tc-2023-058094]
- NCT05715164. Electronic cigarettes and nicotine pouches for smoking cessation. NCT05715164.

### Holliday 2022 {published data only}

- Holliday R, Preshaw P, McColl E, Ryan V, Cherlin S, Wilson N, et al. Research protocol for the ENHANCE-D trial: enhancing dental health advice. Journal of Clinical Periodontology 2022;49(Suppl 23):79. [DOI: 10.1111/jcpe.13634]
- ISRCTN13158982. Enhancing dental health advice. isrctn.com/ISRCTN13158982 (accessed 10 May 2022). [DOI: 10.1186/ISRCTN1315898]
- Weke A, Holmes R, McColl E, Finch R, Butcher C, Holliday R. Delivering smoking cessation interventions in NHS primary dental care - lessons from the enhance-d trial. In: Society for Research on Nicotine and Tobacco (SRNT) 20-23 March 2024 Edinburgh UK. Vol. POS5-7 Rapids. 2024.

### Howard 2022 {published data only}

- \*Howard BC, McRobbie H, Petrie D, Barker D, Mendelsohn C, Anderson J, et al. Effectiveness, safety and cost-effectiveness of vaporized nicotine products versus nicotine replacement therapy for tobacco smoking cessation in a low-socioeconomic status Australian population: a study protocol for a randomized controlled trial. Trials 2022;23(1):777. [DOI: 10.1186/s13063-022-06644-8]
- ACTRN12621000076875. Vaporised nicotine products versus nicotine replacement therapy for tobacco smoking cessation among low-socioeconomic status smokers: a randomised controlled trial. trialsearch.who.int/Trial2.aspx?TrialID=ACTRN12621000076875 (first received 29 January 2021). [CENTRAL: www.cochranelibrary.com/es/central/doi/10.1002/central/CN-02240824/full]

### ISRCTN14068059 {published data only}

- ISRCTN14068059. E-cigarettes for smoking cessation and reduction in people with a mental illness. ISRCTN14068059 2024. [DOI: 10.1186/ISRCTN14068059]

### ISRCTN61193406 {published data only}

- ISRCTN61193406. Do e-cigarettes help smokers quit when not accompanied by intensive behavioural support? Who.int/Trialsearch/Trial2.aspx?TrialID=ISRCTN61193406 2020 (first received 11 August 2020).

### ISRCTN82413824 {published data only}

- ISRCTN82413824. Effectiveness of electronic cigarettes compared with combination nicotine replacement therapy for smoking cessation in patients with chronic obstructive pulmonary disease and effect on lung health (ECAL Trial). ISRCTN82413824.

### Lin 2024 {published data only}

- ChiCTR2100048156. Efficacy of electronic cigarettes vs varenicline and nicotine chewing gum as an aid to stop smoking: a randomized clinical trial. https://www.chictr.org.cn/showproj.html?proj=129413.
- Lin H-X, Liu Z, Hajek P, Zhang W-T, Wu Y, Zhu B-C et al. Efficacy of electronic cigarettes vs varenicline and nicotine chewing gum as an aid to stop smoking: a randomized clinical trial. JAMA Internal Medicine 2024;184(3):291-9. [DOI: 10.1001/jamainternmed.2023.7846]
- Liu Z. Notice of retraction: lin HX ET al. Efficacy of electronic cigarettes vs varenicline and nicotine chewing gum as an aid to stop smoking: a randomized clinical trial. JAMA intern med. 2024;184(3):291-299. JAMA Internal Medicine 2024;184(5):589. [DOI: 10.1001/jamainternmed.2024.1125]

### Malik 2023 {published data only}

- Malik M, Hussain A, Hashmi A, Khan W. Protocol for randomized, two arm parallel, clinical trial for effectiveness of THR products in LMIC. Pakistan Institute of Pharmaceutical Sciences 2023;14(4):1-5.

### Murray 2020 {published data only}

- ISRCTN63825779. Yorkshire enhanced stop smoking (YESS). isrctn.com 2018. [DOI: 10.1186/ISRCTN63825779]
- Murray RL, Brain K, Britton J, Quinn-Scoggins HD, Lewis S, McCutchan GM, et al. Yorkshire Enhanced Stop Smoking (YESS) study: a protocol for a randomised controlled trial to evaluate the effect of adding a personalised smoking cessation intervention to a lung cancer screening programme. BMJ Open 2020;10(9):e037086.
- NCT03750110. Yorkshire enhanced stop smoking study (YESS). https://clinicaltrials.gov/study/NCT03750110 2017.

### NCT01842828 {published data only}

- NCT01842828. E-cigarettes as an addition to multi-component treatment for tobacco dependence: a pilot study. clinicaltrials.gov/show/NCT01842828 (first received 16 July 2014).

### NCT02398487 {published data only}

- NCT02398487. Personal vaporizer vs cigalike (VAPECIG 2) [Head-to-head comparison of personal vaporizers versus cigalike: prospective 6-month randomized control design study]. clinicaltrials.gov/show/NCT02398487 (first received 25 March 2015).

### NCT02590393 {published data only}

- NCT02590393. The role of nicotine and non-nicotine alkaloids in e-cigarette use and dependence. clinicaltrials.gov/show/NCT02590393 (first received 17 February 2016).

### NCT03277495 {published data only}

- NCT03277495. Predictors and consequences of combustible cigarette smokers' switch to standardized research e-cigarettes. clinicaltrials.gov/ct2/show/NCT03277495 (first received 11 September 2017).

### NCT03625986 {published data only}

- NCT03625986. Does switching to nicotine containing electronic cigarettes reduce health risk markers. clinicaltrials.gov/ct2/show/NCT03625986 (first received 10 August 2018).

### NCT03862924 {published data only}

- NCT03862924. Health effects of the standardized research e-cigarette in smokers with HIV. https://clinicaltrials.gov/ct2/show/NCT03862924 (accessed 15th April 2025).

### NCT03962660 {published data only}

- NCT03962660. Harm reduction for tobacco smoking with support of tobacco-replacing electronic nicotine delivery systems (harts-TRENDS). clinicaltrials.gov/ct2/show/record/NCT03962660 (first received 24 May 2019).

### NCT04003805 {published data only}

- NCT04003805. Biomarkers of exposure and effect in standardized research e-cigarette (SREC) users. clinicaltrials.gov/ct2/show/NCT04003805 (first received 1 July 2019).

### NCT04058717 {published data only}

- NCT04058717. Low nicotine cigarettes plus electronic cigarettes [Randomized trial of low nicotine cigarettes plus electronic cigarettes in smokers]. clinicaltrials.gov/ct2/show/NCT04058717 (first received 15 August 2019).

### NCT04063267 {published data only}

- El-Shahawy O, He M, Gee KB, Bayron A, Stevens ER, Lee JD et al. Smoking harm reduction among persons in substance use disorder treatment programs: promising results from a pilot randomized trial. In: Society for Research on Nicotine and Tobacco (SRNT) 20-23 March 2024 Edinburgh UK. Vol. PPS25-3. 2024.
- NCT04063267. Electronic cigarettes as a harm reduction strategy in individuals with substance use disorder. clinicaltrials.gov/ct2/show/NCT04063267 (first received 21 August 2019).

### NCT04218708 {published data only}

- El Shahawy, He M, McNeely J, Spears CA, Gee K, Bayron A et al. Switching to e-cigarettes among people living with HIV/AIDS who smoke combustible cigarettes. In: Society for Research on Nicotine and Tobacco (SRNT) 20-23 March 2024 Edinburgh UK. 2024.
- NCT04218708. Electronic cigarettes as a harm reduction strategy among people living with HIV/AIDS. clinicaltrials.gov/ct2/show/NCT04218708 (first received 6 January 2020).

### NCT04238832 {published data only}

- NCT04238832. Salt-based e-cigarette and IQOS study. clinicaltrials.gov/show/NCT04238832 (first received 18 January 2020).

### NCT04452175 {published data only}

- Caponnetto P. 12-month randomized, double blind, controlled multicentre trial comparing changes in cigarette consumption after switching to high or low nicotine strength e-cigarettes oin smokers with schizophrenia spectrum disorders: protocol for the GENESIS trial. SYM3-2A, SRNT 30th Annual Meeting, New Orleans USA 2024 March 12-15.
- NCT04452175. Cigarette consumption after switchinG to high or low nicotine strENght E-cigaretteS In Smokers With Schizophrenia (GENESIS). clinicaltrials.gov/show/NCT04452175 (first received 30 June 2020).

### NCT04521647 {published data only}

- NCT04521647. Effects of menthol in e-cigarettes on smoking behaviors. clinicaltrials.gov/ct2/show/NCT04521647 (first received 20 August 2020).

### NCT04649645 {published data only}

- Conte G, Pacino SA, Urso S, Greiling D, Caponnetto P, Pedulla E, et al. Changes in oral health and dental esthetic in smokers switching to combustion-free nicotine alternatives: protocol for a multicenter and prospective randomized controlled trial. JMIR Research Protocols 2024;13:e53222. [DOI: 10.2196/53222]
- NCT04649645. International randomized controlled trial evaluating changes in oral health in smokers after switching to combustion-free nicotine delivery systems. clinicaltrials.gov/show/NCT04649645 (first received 02 December 2020).

### NCT04708106 {published data only}

- NCT04708106. Characterization of product use in smokers switching from cigarettes to a RELX electronic nicotine delivery system. clinicaltrials.gov/ct2/show/NCT04708106 (first posted 13 January 2021).

### NCT04709471 {published data only}

- NCT04709471. E-cigarette nicotine study. clinicaltrials.gov/ct2/show/NCT04709471 (first posted 14 January 2021).

### NCT04725656 {published data only}

- NCT04725656. Concentration impact nicotine salt (CINS). clinicaltrials.gov/ct2/show/NCT04725656 (first received 27 January 2021).

### NCT04946825 {published data only}

- NCT04946825. Quit smoking study for people who use e-cigarettes [A randomized controlled trial of smoking cessation treatment for young adult dual users of combustible and electronic cigarettes]. clinicaltrials.gov/ct2/show/NCT04946825 (first received 1 July 2021).

### NCT05023096 {published data only}

- NCT05023096. Potential effects of electronic nicotine delivery system flavor regulations on African American menthol smokers (RVA flavors) [Effects of electronic nicotine delivery system flavor regulations on tobacco behavior, toxicity, and abuse liability among African American menthol smokers]. clinicaltrials.gov/ct2/show/NCT05023096 (first received 26 August 2021).

### NCT05144542 {published data only}

- NCT05144542. Risk and benefits of electronic cigarettes to older smokers at high risk for lung cancer. www.clinicaltrials.gov/ct2/show/NCT05144542 (first received 3 December 2021).

### NCT05199480 {published data only}

- NCT05199480. Understanding the impact of cartridge-based electronic cigarettes and generated aerosols on cardiopulmonary health. clinicaltrials.gov/ct2/show/NCT05199480 (first received 20 January 2022).

### NCT05205811 {published data only}

- NCT05205811. The effects of combination zonisamide and bupropion on switching to an electronic cigarette [A randomized controlled trial to determine the effects of combination zonisamide and bupropion on switching to an electronic cigarette]. clinicaltrials.gov/ct2/show/NCT05205811 (first received 25 January 2022).

### NCT05206435 {published data only}

- NCT05206435. Methadone-maintained smokers switching to e-cigarettes (SHINE). clinicaltrials.gov/ct2/show/NCT05206435 (first received 25 January 2022).

### NCT05257629 {published data only}

- NCT05257629. Aggressive smoking cessation therapy post-acute coronary syndrome (ASAP) trial. www.clinicaltrials.gov/ct2/show/NCT05257629 (first received 25 February 2022).

### NCT05278065 {published data only}

- NCT05278065. E-cigarettes for harm reduction in adult with asthma (SWAP) [Complimentary electronic cigarettes for harm reduction among adult smokers with asthma]. clinicaltrials.gov/ct2/show/NCT05278065 (first received 14 March 2022).

### NCT05510154 {published data only}

- Leavens E, Wagener T, Ellerbeck E, Lambart L, Billinger S, Brown A. Changes in cigarette smoking and health outcomes among smokers with COPD during a 12-week switching. In: Society for Research on Nicotine and Tobacco (SRNT) 20-23 March 2024 Edinburgh UK. Vol. POS3-138 Rapids. 2024.
- NCT05510154. COPD e-cigarette topography training. clinicaltrials.gov/ct2/show/NCT05510154 (first received 22 August 2022).

### NCT05555069 {published data only}

- NCT05555069. The impact of menthol flavoring on switching in adult menthol smokers. clinicaltrials.gov/ct2/show/NCT05555069 (first received 26 September 2022).

### NCT05610514 {published data only}

- NCT05610514. Pulmonary and cardiac effects of e-cigarette use in pulmonary patients who smoke cigarettes. clinicaltrials.gov/ct2/show/NCT05610514 (first received 9 November 2022).

### NCT05703672 {published data only}

- NCT05703672. Switching to e-cigarettes in African-American smokers. clinicaltrials.gov/ct2/show/NCT05703672 (first received 30 January 2023).

### NCT05815199 {published data only}

- NCT05815199. E-cigarettes for harm reduction among smokers with serious mental illness. clinicaltrials.gov/study/NCT05815199 (first received 4 April 2023).

### NCT05825924 {published data only}

- NCT05825924. Effectiveness of different THR products in adult population. clinicaltrials.gov/ct2/show/NCT05825924 (first received 24 April 2023).

### NCT05881304 {published data only}

- NCT05881304. Switching individuals in treatment for opioid use disorder who smoke cigarettes to the SREC (SWITCHED). clinicaltrials.gov/ct2/show/NCT05881304 (first received 31 May 2023).

### NCT05887947 {published data only}

- NCT05887947. Impact of e-cigarette nicotine concentration on compensation. clinicaltrials.gov/ct2/show/NCT05887947 (first received 5 June 2023).

### NCT05960305 {published data only}

- NCT05960305. CSD201204 An actual use study of P12 electronic nicotine delivery system among U.S. adult amokers. https://classic.clinicaltrials.gov/ct2/show/NCT05960305 (first received September 2023).

### NCT06063421 {published data only}

- Comparison of nicotine replacement therapy and electronic cigarettes for smoking cessation in Pakistan. NCT06063421 2023.

### NCT06077240 {published data only}

- Effects of e-cigs vs pouches on cigarette smoking and addiction. NCT06077240.

### NCT06111053 {published data only}

- Trial for harm reduction with incentives and vaping e-cigarettes. NCT06111053 2023.

### NCT06118502 {published data only}

- A clinical trial of adaptive treatment for early smoking cessation relapse (ADAPT). NCT06118502 2023.

### NCT06169813 {published data only}

- E-cigarette harm reduction among PLWHA in south africa. NCT06169813 2023.

### NCT06260683 {published data only}

- NCT06260683. A comprehensive evaluation of tobacco-flavored vs. non-tobacco flavored e-cigarettes on smoking behavior. https://clinicaltrials.gov/ct2/show/NCT06264154 (accessed 15 April 2025).

### NCT06264154 {published data only}

- NCT06264154. The role of flavor in the substitutability of e-cigarettes for combustible cigarettes among persistent smokers. https://clinicaltrials.gov/ct2/show/NCT06264154 (accessed 15 April 2024).

### NCT06372899 {published data only}

- NCT06372899. Noncombustible nicotine delivery systems as potential harm reduction tools for persistent cigarette smokers. Official title: Alternative nicotine delivery systems as potential harm reduction tools for persistent cigarette smokers. https://clinicaltrials.gov/study/NCT06372899 (accessed 15 April 2025).

### NCT06373679 {published data only}

- NCT06373679. Switch or quit R01 [Non-cigarette tobacco products as harm reduction tools in smokers who failed to quit with traditional methods]. https://clinicaltrials.gov/ct2/show/NCT06373679 (accessed 16 April 2025).

### NCT06534905 {published data only}

- NCT06534905. E-cigarette switching older adults [Official title: pilot randomized controlled trial of e-cigarette switching among older adults with opioid use disorder]. https://clinicaltrials.gov/ct2/show/NCT06534905 (accessed 16 April 2025).

### NCT06543407 {published data only}

- NCT06543407. Harm reduction for smokers with mental illness. https://clinicaltrials.gov/ct2/show/NCT06543407 (accessed 16 April 2025).

### NCT06554873 {published data only}

- NCT06554873. Adaptive use of nicotine substitution to maintain smoking reduction/abstinence in nicotine responders. https://clinicaltrials.gov/ct2/show/NCT06554873 (accessed 16 April 2025).

### NCT06614504 {published data only}

- NCT06614504. Nicotine regulation for dual users of e-cigarettes and cigarettes (RDEC). https://clinicaltrials.gov/ct2/show/NCT06614504 (accessed 16 April 2025).

### Polosa 2024 {published data only}

- Belsey J, Weglarz J, Scherer M, Pluym N, Polosa R. Statistical analyses plan for "Magnitude of cigarette substitution after initiation of e-cigarettes and its impact on biomarkers of exposure and potential harm in dual users": MAGNIFICAT trial. Heliyon 2024;10(21):e39695. [DOI: 10.1016/j.heliyon.2024.e39695]
- Polosa R, Pluym N, Scherer M, Belsey J, Russell C, Caponnetto P, et al. Protocol for the "magnitude of cigarette substitution after initiation of e-cigarettes and its impact on biomarkers of exposure and potential harm in dual users" (MAGNIFICAT) study. Frontiers in Public Health 2024;12(101616579):1348389. [DOI: 10.3389/fpubh.2024.1348389]

### Schiek 2024 {published data only}

- \*Schiek H, Esch T, Michaelsen MM, Hoetger C. Combining app-based behavioral therapy with electronic cigarettes for smoking cessation: A study protocol for a single-arm mixed-methods pilot trial,. Addiction Science & Clinical Practice / 2024;19(1): 2024;19(52):1-20. [DOI: 10.1186/s13722-024-00483-5]
- German clinical trials register DRKS00032652, registered 09/15/2023. https://drks.de/search/de/trial/ DRKS00032652.

### Walker 2023 {published data only}

- \*Walker N, Calder A, Barnes J, Laking G, Parag V, Bullen C. Effectiveness of nicotine salt vapes, cytisine, and a combination of these products, for smoking cessation in New Zealand: protocol for a three-arm, pragmatic, community-based randomised controlled trial. BMC Public Health 2023;23(1):1760. [DOI: 10.1186/s12889-023-16665-w]
- NCT05311085. Cytisine and e-cigarettes with supportive text-messaging for smoking cessation (Cess@Tion). clinicaltrials.gov/ct2/show/NCT05311085 (first received 5 April 2022).
- Walker N, Calder A, Barnes J, Laking G, Parag V, Bullen C. Correction: Effectiveness of nicotine salt vapes, cytisine, and a combination of these products, for smoking cessation in New Zealand: protocol for a three-arm, pragmatic, community-based randomised controlled trial. BMC Public Health 2025;25(1):270. [DOI: 10.1186/s12889-025-21438-8]
