# Supplementary material for: Electronic cigarettes for smoking cessation
Source: Cochrane Database Syst Rev. 2025 Nov 10;2025(11):CD010216. doi: 10.1002/14651858.CD010216.pub10 (PMC12599494; doi:10.1002/14651858.CD010216.pub10)
Supplement: Supplementary file 6 — Supplementary material 6 Analyses [file CD010216-SUP-06-analyses.html]

Analyses


# Supplementary material 6 to: Electronic cigarettes for smoking cessation

Lindson N, Livingstone-Banks J, Butler AR, McRobbie H, Bullen CR, Hajek P, Wu AD, Begh R, Theodoulou A, Notley C, Rigotti NA, Turner T, Fanshawe T, Hartmann-Boyce J
  
https://doi.org/10.1002/14651858.CD010216.pub10

The material in this section has been supplied by the author(s) for publication under a Licence for Publication and the author(s) are solely responsible for the material. Cochrane has reviewed this material, but Cochrane has not copyedited, formatted or proofread. Cochrane accordingly gives no representations or warranties of any kind in relation to, and accepts no liability for any reliance on or use of, such material.

Back to top

# Analyses

## Analysis group 1: Nicotine EC versus NRT

| Analysis or subgroup title | No. of studies | No. of participants | Statistical method | Effect size |
| --- | --- | --- | --- | --- |
| 1.1 Smoking cessation | 9 | 2703 | Risk Ratio (M-H, Fixed, 95% CI) | 1.55 [1.28, 1.88] |
| 1.1.1 Not selected on pregnancy | 8 | 2384 | Risk Ratio (M-H, Fixed, 95% CI) | 1.55 [1.28, 1.87] |
| 1.1.2 Pregnant population | 1 | 319 | Risk Ratio (M-H, Fixed, 95% CI) | 1.78 [0.45, 6.97] |
| 1.2 Adverse events | 7 | 2241 | Risk Ratio (M-H, Random, 95% CI) | 1.00 [0.73, 1.37] |
| 1.3 Serious adverse events | 8 | 2950 | Risk Ratio (M-H, Random, 95% CI) | 1.22 [0.73, 2.03] |
| 1.4 Carbon monoxide (ppm) | 5 | 385 | Mean Difference (IV, Random, 95% CI) | −1.98 [−3.78, −0.18] |
| 1.4.1 Absolute values at follow-up | 1 | 110 | Mean Difference (IV, Random, 95% CI) | −1.87 [−5.15, 1.41] |
| 1.4.2 Change from baseline | 4 | 275 | Mean Difference (IV, Random, 95% CI) | −2.93 [−6.20, 0.34] |
| 1.5 Heart rate (bpm) | 2 | 166 | Mean Difference (IV, Random, 95% CI) | 0.53 [−1.76, 2.83] |
| 1.5.1 Absolute values at follow-up | 1 | 111 | Mean Difference (IV, Random, 95% CI) | −0.74 [−5.17, 3.69] |
| 1.5.2 Change from baseline | 1 | 55 | Mean Difference (IV, Random, 95% CI) | 1.00 [−1.69, 3.69] |
| 1.6 Systolic blood pressure | 2 | 166 | Mean Difference (IV, Random, 95% CI) | −1.62 [−3.59, 0.36] |
| 1.6.1 Absolute values at follow-up | 1 | 111 | Mean Difference (IV, Random, 95% CI) | 1.00 [−4.54, 6.54] |
| 1.6.2 Change from baseline | 1 | 55 | Mean Difference (IV, Random, 95% CI) | −2.00 [−4.11, 0.11] |
| 1.7 Blood oxygen saturation | 2 | 165 | Mean Difference (IV, Random, 95% CI) | −0.14 [−0.59, 0.30] |
| 1.7.1 Absolute values at follow-up | 1 | 110 | Mean Difference (IV, Random, 95% CI) | −0.20 [−0.72, 0.32] |
| 1.7.2 Change from baseline | 1 | 55 | Mean Difference (IV, Random, 95% CI) | 0.00 [−0.83, 0.83] |
| 1.8 3-HPMA (pmol/mg creatinine) | 1 | 111 | Mean Difference (IV, Random, 95% CI) | −807.00 [−2559.47, 945.47] |
| 1.8.1 Absolute values at follow-up | 1 | 111 | Mean Difference (IV, Random, 95% CI) | −807.00 [−2559.47, 945.47] |
| 1.9 NNAL (pmol/mg creatinine) | 1 | 110 | Mean Difference (IV, Random, 95% CI) | 0.00 [−0.53, 0.53] |
| 1.9.1 Absolute values at follow-up | 1 | 110 | Mean Difference (IV, Random, 95% CI) | 0.00 [−0.53, 0.53] |
| 1.10 2-HPMA (pmol/mg creatinine) | 1 | 111 | Mean Difference (IV, Random, 95% CI) | −109.00 [−474.52, 256.52] |
| 1.10.1 Absolute values at follow-up | 1 | 111 | Mean Difference (IV, Random, 95% CI) | −109.00 [−474.52, 256.52] |
| 1.11 HMPMA (pmol/mg creatinine) | 1 | 111 | Mean Difference (IV, Random, 95% CI) | −875.00 [−2300.93, 550.93] |
| 1.11.1 Absolute values at follow-up | 1 | 111 | Mean Difference (IV, Random, 95% CI) | −875.00 [−2300.93, 550.93] |
| 1.12 PheT (pmol/mg creatinine) | 1 | 109 | Mean Difference (IV, Random, 95% CI) | 0.40 [−0.67, 1.47] |
| 1.12.1 Absolute values at follow-up | 1 | 109 | Mean Difference (IV, Random, 95% CI) | 0.40 [−0.67, 1.47] |
| 1.13 CEMA (pmol/mg creatinine) | 1 | 111 | Mean Difference (IV, Random, 95% CI) | 37.00 [−121.50, 195.50] |
| 1.13.1 Absolute values at follow-up | 1 | 111 | Mean Difference (IV, Random, 95% CI) | 37.00 [−121.50, 195.50] |
| 1.14 AAMA (pmol/mg creatinine) | 1 | 109 | Mean Difference (IV, Random, 95% CI) | 32.00 [−109.35, 173.35] |
| 1.14.1 Absolute values at follow-up | 1 | 109 | Mean Difference (IV, Random, 95% CI) | 32.00 [−109.35, 173.35] |
| 1.15 FEV1 | 2 |  | Std. Mean Difference (IV, Random, 95% CI) | Totals not selected |
| 1.15.1 Change from baseline | 2 |  | Std. Mean Difference (IV, Random, 95% CI) | Totals not selected |
| 1.16 FEV1/FVC (%) | 2 | 81 | Mean Difference (IV, Random, 95% CI) | 10.15 [−24.36, 44.67] |
| 1.16.1 Change from baseline | 2 | 81 | Mean Difference (IV, Random, 95% CI) | 10.15 [−24.36, 44.67] |
| 1.17 PEF (L/min) | 1 | 55 | Mean Difference (IV, Random, 95% CI) | −3.00 [−27.09, 21.09] |
| 1.17.1 Change from baseline | 1 | 55 | Mean Difference (IV, Random, 95% CI) | −3.00 [−27.09, 21.09] |
| 1.18 Product use at 6+ months | 5 |  | Risk Ratio (M-H, Random, 95% CI) | Totals not selected |

## Analysis group 2: Nicotine EC versus varenicline

| Analysis or subgroup title | No. of studies | No. of participants | Statistical method | Effect size |
| --- | --- | --- | --- | --- |
| 2.1 Smoking cessation | 1 | 54 | Risk Ratio (M-H, Random, 95% CI) | 0.31 [0.11, 0.82] |
| 2.2 Serious adverse events | 1 | 54 | Risk Ratio (M-H, Random, 95% CI) | Not estimable |

## Analysis group 3: Nicotine EC versus non-nicotine EC + varenicline

| Analysis or subgroup title | No. of studies | No. of participants | Statistical method | Effect size |
| --- | --- | --- | --- | --- |
| 3.1 Smoking cessation | 1 | 305 | Risk Ratio (M-H, Random, 95% CI) | 0.73 [0.53, 1.01] |
| 3.2 Serious adverse events | 1 | 305 | Risk Ratio (M-H, Random, 95% CI) | 5.03 [0.24, 103.97] |

## Analysis group 4: Nicotine EC versus NRT + bupropion

| Analysis or subgroup title | No. of studies | No. of participants | Statistical method | Effect size |
| --- | --- | --- | --- | --- |
| 4.1 Smoking cessation | 1 | 38 | Risk Ratio (M-H, Random, 95% CI) | 0.89 [0.44, 1.81] |

## Analysis group 5: Nicotine EC versus heated tobacco

| Analysis or subgroup title | No. of studies | No. of participants | Statistical method | Effect size |
| --- | --- | --- | --- | --- |
| 5.1 Adverse events | 1 | 220 | Risk Ratio (M-H, Random, 95% CI) | 0.86 [0.68, 1.10] |
| 5.2 SAEs | 1 | 220 | Risk Ratio (M-H, Random, 95% CI) | Not estimable |
| 5.3 Carbon monoxide (ppm) | 2 | 267 | Mean Difference (IV, Random, 95% CI) | 1.00 [−1.04, 3.03] |
| 5.3.1 Absolute values at 12 weeks | 1 | 217 | Mean Difference (IV, Random, 95% CI) | 1.90 [−0.71, 4.51] |
| 5.3.2 Change at 1 month | 1 | 50 | Mean Difference (IV, Random, 95% CI) | −0.20 [−3.23, 2.83] |
| 5.4 VO2 Max at 12 weeks | 1 | 211 | Mean Difference (IV, Random, 95% CI) | 6.20 [−2.01, 14.41] |

## Analysis group 6: Nicotine EC versus oral nicotine pouches

| Analysis or subgroup title | No. of studies | No. of participants | Statistical method | Effect size |
| --- | --- | --- | --- | --- |
| 6.1 Serious adverse events | 1 | 26 | Risk Ratio (M-H, Random, 95% CI) | Not estimable |
| 6.2 Carbon monoxide (ppm) | 1 | 26 | Mean Difference (IV, Random, 95% CI) | −12.44 [−28.82, 3.94] |
| 6.2.1 Change from baseline | 1 | 26 | Mean Difference (IV, Random, 95% CI) | −12.44 [−28.82, 3.94] |

## Analysis group 7: Nicotine EC versus non-nicotine EC

| Analysis or subgroup title | No. of studies | No. of participants | Statistical method | Effect size |
| --- | --- | --- | --- | --- |
| 7.1 Smoking cessation | 7 | 1918 | Risk Ratio (M-H, Random, 95% CI) | 1.34 [1.06, 1.70] |
| 7.2 Adverse events | 5 | 840 | Risk Ratio (M-H, Random, 95% CI) | 1.01 [0.95, 1.08] |
| 7.3 Serious adverse events | 10 | 1717 | Risk Ratio (M-H, Random, 95% CI) | 0.98 [0.55, 1.73] |
| 7.4 Carbon monoxide (ppm) | 6 |  | Mean Difference (IV, Random, 95% CI) | Totals not selected |
| 7.4.1 Change from baseline | 3 |  | Mean Difference (IV, Random, 95% CI) | Totals not selected |
| 7.4.2 Absolute values at follow-up | 3 |  | Mean Difference (IV, Random, 95% CI) | Totals not selected |
| 7.5 Heart rate | 2 | 401 | Mean Difference (IV, Random, 95% CI) | −1.23 [−3.55, 1.08] |
| 7.5.1 Absolute values at follow-up | 2 | 401 | Mean Difference (IV, Random, 95% CI) | −1.23 [−3.55, 1.08] |
| 7.6 Systolic blood pressure | 2 | 401 | Mean Difference (IV, Random, 95% CI) | 2.50 [−0.45, 5.44] |
| 7.6.1 Absolute values at follow-up | 2 | 401 | Mean Difference (IV, Random, 95% CI) | 2.50 [−0.45, 5.44] |
| 7.7 NNAL (pmol/mg creatinine) | 2 | 363 | Mean Difference (IV, Random, 95% CI) | 4.13 [−9.21, 17.48] |
| 7.7.1 Change from baseline | 1 | 148 | Mean Difference (IV, Random, 95% CI) | 15.27 [−4.98, 35.52] |
| 7.7.2 Absolute values at follow-up | 1 | 215 | Mean Difference (IV, Random, 95% CI) | −0.03 [−0.46, 0.40] |
| 7.8 FeNO (ppb) | 1 | 90 | Mean Difference (IV, Random, 95% CI) | 2.35 [1.78, 2.92] |
| 7.8.1 Change from baseline | 1 | 90 | Mean Difference (IV, Random, 95% CI) | 2.35 [1.78, 2.92] |
| 7.9 FEV1 (l) | 2 |  | Std. Mean Difference (IV, Random, 95% CI) | Totals not selected |
| 7.9.1 Absolute values at follow-up | 2 |  | Std. Mean Difference (IV, Random, 95% CI) | Totals not selected |
| 7.10 FEV1/FVC | 2 | 348 | Mean Difference (IV, Random, 95% CI) | −0.14 [−1.76, 1.47] |
| 7.10.1 Absolute values at follow-up | 2 | 348 | Mean Difference (IV, Random, 95% CI) | −0.14 [−1.76, 1.47] |
| 7.11 FVC (l) | 1 | 260 | Std. Mean Difference (IV, Random, 95% CI) | 0.20 [−0.05, 0.44] |
| 7.11.1 Absolute values at follow-up | 1 | 260 | Std. Mean Difference (IV, Random, 95% CI) | 0.20 [−0.05, 0.44] |
| 7.12 PEF (l/s) | 1 | 260 | Std. Mean Difference (IV, Random, 95% CI) | 0.21 [−0.03, 0.45] |
| 7.12.1 Absolute values at follow-up | 1 | 260 | Std. Mean Difference (IV, Random, 95% CI) | 0.21 [−0.03, 0.45] |
| 7.13 FEF 25-75 (l/s) | 1 | 260 | Std. Mean Difference (IV, Random, 95% CI) | 0.17 [−0.07, 0.42] |
| 7.13.1 Absolute values at follow-up | 1 | 260 | Std. Mean Difference (IV, Random, 95% CI) | 0.17 [−0.07, 0.42] |
| 7.14 Product use at 6+ months | 3 | 874 | Risk Ratio (M-H, Random, 95% CI) | 1.14 [0.77, 1.69] |

## Analysis group 8: Nicotine EC versus behavioural support only/no support

| Analysis or subgroup title | No. of studies | No. of participants | Statistical method | Effect size |
| --- | --- | --- | --- | --- |
| 8.1 Smoking cessation | 11 | 6819 | Risk Ratio (M-H, Random, 95% CI) | 1.78 [1.42, 2.25] |
| 8.2 Adverse events | 8 | 2485 | Risk Ratio (M-H, Random, 95% CI) | 1.22 [0.96, 1.55] |
| 8.3 Serious adverse events | 15 | 4716 | Risk Ratio (M-H, Random, 95% CI) | 0.93 [0.67, 1.29] |
| 8.4 Carbon monoxide (ppm) | 14 |  | Mean Difference (IV, Random, 95% CI) | Totals not selected |
| 8.4.1 Change from baseline | 5 |  | Mean Difference (IV, Random, 95% CI) | Totals not selected |
| 8.4.2 Absolute values at follow-up | 9 |  | Mean Difference (IV, Random, 95% CI) | Totals not selected |
| 8.5 Heart rate (bpm) | 1 | 90 | Mean Difference (IV, Random, 95% CI) | 1.17 [−4.27, 6.61] |
| 8.5.1 Absolute values at follow-up | 1 | 90 | Mean Difference (IV, Random, 95% CI) | 1.17 [−4.27, 6.61] |
| 8.6 Systolic blood pressure | 3 | 298 | Mean Difference (IV, Random, 95% CI) | −1.64 [−7.97, 4.70] |
| 8.6.1 Change from baseline | 1 | 168 | Mean Difference (IV, Random, 95% CI) | −2.68 [−4.38, −0.98] |
| 8.6.2 Absolute values at follow-up | 2 | 130 | Mean Difference (IV, Random, 95% CI) | 1.11 [−25.99, 28.21] |
| 8.7 Blood oxygen saturation | 1 | 89 | Mean Difference (IV, Random, 95% CI) | 0.20 [−0.30, 0.70] |
| 8.7.1 Absolute values at follow-up | 1 | 89 | Mean Difference (IV, Random, 95% CI) | 0.20 [−0.30, 0.70] |
| 8.8 3-HPMA (SMD) | 2 | 474 | Std. Mean Difference (IV, Random, 95% CI) | −0.46 [−0.66, −0.26] |
| 8.8.1 Absolute values at follow-up | 1 | 90 | Std. Mean Difference (IV, Random, 95% CI) | −0.30 [−0.74, 0.13] |
| 8.8.2 Change from baseline | 1 | 384 | Std. Mean Difference (IV, Random, 95% CI) | −0.50 [−0.73, −0.27] |
| 8.9 NNAL (SMD) | 5 |  | Std. Mean Difference (IV, Random, 95% CI) | Totals not selected |
| 8.9.1 Absolute values at follow-up | 2 |  | Std. Mean Difference (IV, Random, 95% CI) | Totals not selected |
| 8.9.2 Change from baseline | 3 |  | Std. Mean Difference (IV, Random, 95% CI) | Totals not selected |
| 8.10 2-HPMA (pmol/mg creatinine) | 1 | 90 | Mean Difference (IV, Random, 95% CI) | −279.90 [−969.98, 410.18] |
| 8.10.1 Absolute values at follow-up | 1 | 90 | Mean Difference (IV, Random, 95% CI) | −279.90 [−969.98, 410.18] |
| 8.11 HMPMA (pmol/mg creatinine) | 1 | 90 | Mean Difference (IV, Random, 95% CI) | −1672.00 [−3857.37, 513.37] |
| 8.11.1 Absolute values at follow-up | 1 | 90 | Mean Difference (IV, Random, 95% CI) | −1672.00 [−3857.37, 513.37] |
| 8.12 PheT (pmol/mg creatinine) | 1 | 88 | Mean Difference (IV, Random, 95% CI) | −1.30 [−4.17, 1.57] |
| 8.12.1 Absolute values at follow-up | 1 | 88 | Mean Difference (IV, Random, 95% CI) | −1.30 [−4.17, 1.57] |
| 8.13 CEMA (pmol/mg creatinine) | 1 | 90 | Mean Difference (IV, Random, 95% CI) | 3.00 [−165.47, 171.47] |
| 8.13.1 Absolute values at follow-up | 1 | 90 | Mean Difference (IV, Random, 95% CI) | 3.00 [−165.47, 171.47] |
| 8.14 AAMA (pmol/mg creatinine) | 1 | 90 | Mean Difference (IV, Random, 95% CI) | −67.90 [−219.73, 83.93] |
| 8.14.1 Absolute values at follow-up | 1 | 90 | Mean Difference (IV, Random, 95% CI) | −67.90 [−219.73, 83.93] |
| 8.15 S-PMA (nanograms) | 1 | 384 | Mean Difference (IV, Random, 95% CI) | −1371.00 [−1995.23, −746.77] |
| 8.15.1 12 weeks | 1 | 384 | Mean Difference (IV, Random, 95% CI) | −1371.00 [−1995.23, −746.77] |
| 8.16 FEV1 (SMD) | 2 | 714 | Std. Mean Difference (IV, Random, 95% CI) | 0.15 [−0.14, 0.44] |
| 8.16.1 Change from baseline | 2 | 714 | Std. Mean Difference (IV, Random, 95% CI) | 0.15 [−0.14, 0.44] |
| 8.17 FEF 25-75 (litres/second)) | 2 | 555 | Mean Difference (IV, Random, 95% CI) | −0.03 [−0.27, 0.20] |
| 8.17.1 Change from baseline | 2 | 555 | Mean Difference (IV, Random, 95% CI) | −0.03 [−0.27, 0.20] |
| 8.18 PEF 25-75 (litres/minute) | 1 | 387 | Mean Difference (IV, Random, 95% CI) | −7.10 [−29.14, 14.94] |
| 8.18.1 Change from baseline | 1 | 387 | Mean Difference (IV, Random, 95% CI) | −7.10 [−29.14, 14.94] |
| 8.19 FEV1/FVC | 1 | 327 | Mean Difference (IV, Random, 95% CI) | 1.72 [0.74, 2.70] |
| 8.19.1 Change from baseline | 1 | 327 | Mean Difference (IV, Random, 95% CI) | 1.72 [0.74, 2.70] |

## Analysis group 9: Higher versus lower nicotine content

| Analysis or subgroup title | No. of studies | No. of participants | Statistical method | Effect size |
| --- | --- | --- | --- | --- |
| 9.1 Smoking cessation | 1 | 260 | Risk Ratio (M-H, Random, 95% CI) | 2.50 [0.80, 7.77] |
| 9.2 Adverse events | 1 | 68 | Risk Ratio (M-H, Random, 95% CI) | 0.90 [0.58, 1.40] |
| 9.3 Serious adverse events | 2 | 239 | Risk Ratio (M-H, Random, 95% CI) | 1.51 [0.51, 4.42] |
| 9.4 Carbon monoxide (ppm) | 3 | 348 | Mean Difference (IV, Random, 95% CI) | −0.92 [−1.71, −0.13] |
| 9.4.1 Change from baseline | 2 | 309 | Mean Difference (IV, Random, 95% CI) | −0.90 [−1.70, −0.10] |
| 9.4.2 Absolute values at follow-up | 1 | 39 | Mean Difference (IV, Random, 95% CI) | −1.66 [−6.65, 3.33] |
| 9.5 Heart rate | 2 | 408 | Mean Difference (IV, Random, 95% CI) | 0.36 [−1.91, 2.62] |
| 9.5.1 Change from baseline | 1 | 148 | Mean Difference (IV, Random, 95% CI) | −0.50 [−1.63, 0.63] |
| 9.5.2 Absolute values at follow-up | 1 | 260 | Mean Difference (IV, Random, 95% CI) | 1.92 [−0.89, 4.73] |
| 9.6 Systolic blood pressure | 2 | 408 | Mean Difference (IV, Random, 95% CI) | 0.86 [−0.77, 2.48] |
| 9.6.1 Change from baseline | 1 | 148 | Mean Difference (IV, Random, 95% CI) | 0.80 [−0.99, 2.59] |
| 9.6.2 Absolute values at follow-up | 1 | 260 | Mean Difference (IV, Random, 95% CI) | 1.13 [−2.76, 5.02] |
| 9.7 FeNO (ppb) | 1 | 93 | Mean Difference (IV, Random, 95% CI) | 0.30 [−0.37, 0.97] |
| 9.7.1 12 weeks | 1 | 93 | Mean Difference (IV, Random, 95% CI) | 0.30 [−0.37, 0.97] |
| 9.8 FEV1 (l) | 2 | 350 | Mean Difference (IV, Random, 95% CI) | 0.05 [−0.10, 0.20] |
| 9.8.1 Change from baseline | 1 | 90 | Mean Difference (IV, Random, 95% CI) | −0.01 [−0.11, 0.09] |
| 9.8.2 Absolute values at follow-up | 1 | 260 | Mean Difference (IV, Random, 95% CI) | 0.15 [−0.04, 0.34] |
| 9.9 FVC (l) | 2 | 350 | Mean Difference (IV, Random, 95% CI) | −0.05 [−0.16, 0.06] |
| 9.9.1 Change from baseline | 1 | 90 | Mean Difference (IV, Random, 95% CI) | −0.03 [−0.15, 0.09] |
| 9.9.2 Absolute values at follow-up | 1 | 260 | Mean Difference (IV, Random, 95% CI) | −0.12 [−0.36, 0.12] |
| 9.10 FEV1/FVC | 2 | 350 | Mean Difference (IV, Random, 95% CI) | 0.91 [0.18, 1.64] |
| 9.10.1 Change from baseline | 1 | 90 | Mean Difference (IV, Random, 95% CI) | 0.91 [0.15, 1.67] |
| 9.10.2 Absolute values at follow-up | 1 | 260 | Mean Difference (IV, Random, 95% CI) | 0.89 [−1.56, 3.34] |
| 9.11 PEF (l/s) | 1 | 260 | Std. Mean Difference (IV, Random, 95% CI) | 0.06 [−0.18, 0.31] |
| 9.11.1 Absolute values at follow-up | 1 | 260 | Std. Mean Difference (IV, Random, 95% CI) | 0.06 [−0.18, 0.31] |
| 9.12 FEF 25-75 (l/s) | 1 | 260 | Std. Mean Difference (IV, Random, 95% CI) | −0.02 [−0.26, 0.23] |
| 9.12.1 Absolute values at follow-up | 1 | 260 | Std. Mean Difference (IV, Random, 95% CI) | −0.02 [−0.26, 0.23] |
| 9.13 NNAL (pg/mg creatinine) at 24 weeks | 1 | 152 | Mean Difference (IV, Random, 95% CI) | −13.89 [−97.42, 69.64] |
| 9.14 Product use at 6+ months | 1 | 260 | Risk Ratio (M-H, Random, 95% CI) | 1.27 [0.95, 1.68] |

## Analysis group 10: Choice of flavours vs. tobacco flavour only

| Analysis or subgroup title | No. of studies | No. of participants | Statistical method | Effect size |
| --- | --- | --- | --- | --- |
| 10.1 Smoking cessation | 1 | 566 | Risk Ratio (M-H, Random, 95% CI) | 0.80 [0.54, 1.16] |
| 10.2 Product use at 6+ months | 1 | 522 | Risk Ratio (M-H, Random, 95% CI) | 1.10 [0.86, 1.40] |
| 10.3 Adverse events (EC as adjunct to VLNC) | 1 | 158 | Risk Ratio (M-H, Random, 95% CI) | 1.01 [0.88, 1.15] |
| 10.4 Serious adverse events (EC as adjunct to VLNC) | 1 | 158 | Risk Ratio (M-H, Random, 95% CI) | 0.44 [0.08, 2.34] |
| 10.5 Change in carbon monoxide (ppm; as adjunct to VLNC) | 1 | 124 | Mean Difference (IV, Random, 95% CI) | −4.52 [−11.06, 2.02] |
| 10.6 Change in NNAL (pmol/mg creatinine; as adjunct to VLNC) | 1 | 100 | Mean Difference (IV, Random, 95% CI) | −0.09 [−1.26, 1.08] |

## Analysis group 11: Tobacco vs. menthol flavour

| Analysis or subgroup title | No. of studies | No. of participants | Statistical method | Effect size |
| --- | --- | --- | --- | --- |
| 11.1 Serious adverse events | 1 | 300 | Risk Ratio (M-H, Random, 95% CI) | Not estimable |
| 11.2 NNAL (ng/g) | 1 | 232 | Mean Difference (IV, Random, 95% CI) | −26.10 [−66.73, 14.53] |
| 11.2.1 Change from baseline | 1 | 232 | Mean Difference (IV, Random, 95% CI) | −26.10 [−66.73, 14.53] |
| 11.3 FEV1 (% predicted) | 1 | 212 | Mean Difference (IV, Random, 95% CI) | −0.67 [−2.34, 1.00] |
| 11.3.1 Change from baseline | 1 | 212 | Mean Difference (IV, Random, 95% CI) | −0.67 [−2.34, 1.00] |
| 11.4 FEV1/FVC | 1 | 212 | Mean Difference (IV, Random, 95% CI) | −0.46 [−1.67, 0.75] |
| 11.4.1 Change from baseline | 1 | 212 | Mean Difference (IV, Random, 95% CI) | −0.46 [−1.67, 0.75] |

## Analysis group 12: Refillable versus cartridge

| Analysis or subgroup title | No. of studies | No. of participants | Statistical method | Effect size |
| --- | --- | --- | --- | --- |
| 12.1 Exhaled CO | 1 | 32 | Mean Difference (IV, Random, 95% CI) | 0.70 [−4.98, 6.38] |

## Analysis group 13: Nicotine salt EC versus free-base nicotine EC

| Analysis or subgroup title | No. of studies | No. of participants | Statistical method | Effect size |
| --- | --- | --- | --- | --- |
| 13.1 Smoking cessation | 1 | 285 | Risk Ratio (M-H, Random, 95% CI) | 1.25 [0.85, 1.83] |
| 13.2 Product use at 6+ months | 1 | 227 | Risk Ratio (M-H, Random, 95% CI) | 1.07 [0.82, 1.41] |

## Analysis group 14: Higher versus lower wattage

| Analysis or subgroup title | No. of studies | No. of participants | Statistical method | Effect size |
| --- | --- | --- | --- | --- |
| 14.1 Smoking cessation | 1 | 267 | Risk Ratio (M-H, Random, 95% CI) | 0.72 [0.30, 1.74] |
| 14.2 Adverse events | 1 | 267 | Risk Ratio (M-H, Random, 95% CI) | 0.92 [0.79, 1.06] |
| 14.3 Serious adverse events | 1 | 267 | Risk Ratio (M-H, Random, 95% CI) | 0.99 [0.14, 6.94] |

## Analysis group 15: Non-nicotine EC versus behavioural support only/no support

| Analysis or subgroup title | No. of studies | No. of participants | Statistical method | Effect size |
| --- | --- | --- | --- | --- |
| 15.1 Smoking cessation | 2 | 388 | Risk Ratio (M-H, Random, 95% CI) | 1.59 [0.80, 3.19] |
| 15.2 Adverse events | 1 | 248 | Risk Ratio (M-H, Random, 95% CI) | 1.28 [1.13, 1.44] |
| 15.3 Serious adverse events | 2 | 388 | Risk Ratio (M-H, Random, 95% CI) | 1.19 [0.33, 4.33] |

## Analysis group 16: Non-nicotine EC + NRT versus NRT

| Analysis or subgroup title | No. of studies | No. of participants | Statistical method | Effect size |
| --- | --- | --- | --- | --- |
| 16.1 Smoking cessation | 1 | 624 | Risk Ratio (M-H, Random, 95% CI) | 1.67 [0.50, 5.53] |
| 16.2 Adverse events | 1 | 344 | Risk Ratio (M-H, Random, 95% CI) | 0.70 [0.53, 0.91] |
| 16.3 Serious adverse events | 1 | 624 | Risk Ratio (M-H, Random, 95% CI) | 1.69 [0.60, 4.74] |

## Analysis group 17: Non-nicotine EC versus NRT

| Analysis or subgroup title | No. of studies | No. of participants | Statistical method | Effect size |
| --- | --- | --- | --- | --- |
| 17.1 Smoking cessation | 2 | 314 | Risk Ratio (M-H, Random, 95% CI) | 0.99 [0.64, 1.54] |
| 17.2 Adverse events | 1 | 132 | Risk Ratio (M-H, Random, 95% CI) | 0.33 [0.12, 0.87] |
| 17.3 Serious adverse events | 1 | 132 | Risk Ratio (M-H, Random, 95% CI) | Not estimable |
| 17.4 Change in carbon monoxide (ppm) at 6 months | 1 | 164 | Mean Difference (IV, Random, 95% CI) | 2.00 [−0.50, 4.50] |

## Analysis group 18: Advice to use e-cigarettes compared to no advice to use e-cigarettes

| Analysis or subgroup title | No. of studies | No. of participants | Statistical method | Effect size |
| --- | --- | --- | --- | --- |
| 18.1 Smoking cessation | 2 | 2652 | Risk Ratio (M-H, Random, 95% CI) | 1.02 [0.88, 1.19] |
| 18.2 Adverse events | 1 | 52 | Risk Ratio (M-H, Random, 95% CI) | 1.27 [0.72, 2.26] |
| 18.3 Serious adverse events | 1 | 52 | Risk Ratio (M-H, Random, 95% CI) | Not estimable |
| 18.4 Product use at 6+ months | 1 | 331 | Risk Ratio (M-H, Random, 95% CI) | 1.77 [0.83, 3.79] |

## Analysis group 19: Nicotine EC + NRT versus non-nicotine EC + NRT

| Analysis or subgroup title | No. of studies | No. of participants | Statistical method | Effect size |
| --- | --- | --- | --- | --- |
| 19.1 Smoking cessation | 2 | 1039 | Risk Ratio (M-H, Random, 95% CI) | 1.77 [1.07, 2.94] |
| 19.2 Adverse events | 2 | 677 | Risk Ratio (M-H, Random, 95% CI) | 1.11 [0.93, 1.32] |
| 19.3 Serious adverse events | 2 | 1069 | Risk Ratio (M-H, Random, 95% CI) | 0.66 [0.38, 1.14] |
| 19.4 Carbon monoxide (ppm) | 2 | 70 | Mean Difference (IV, Random, 95% CI) | −4.62 [−12.07, 2.82] |
| 19.4.1 change from baseline | 2 | 70 | Mean Difference (IV, Random, 95% CI) | −4.62 [−12.07, 2.82] |
| 19.5 FeNO (ppb) | 1 | 30 | Mean Difference (IV, Random, 95% CI) | −0.36 [−7.23, 6.51] |
| 19.5.1 6 months | 1 | 30 | Mean Difference (IV, Random, 95% CI) | −0.36 [−7.23, 6.51] |
| 19.6 FEV1 (%) | 1 | 32 | Mean Difference (IV, Random, 95% CI) | 0.05 [−0.01, 0.10] |
| 19.6.1 6 months | 1 | 32 | Mean Difference (IV, Random, 95% CI) | 0.05 [−0.01, 0.10] |
| 19.7 FVC (%) | 1 | 32 | Mean Difference (IV, Random, 95% CI) | 0.03 [−0.03, 0.09] |
| 19.7.1 6 months | 1 | 32 | Mean Difference (IV, Random, 95% CI) | 0.03 [−0.03, 0.09] |
| 19.8 Study product use at 6+ months | 1 | 9 | Risk Ratio (M-H, Random, 95% CI) | 1.25 [0.29, 5.35] |

## Analysis group 20: Nicotine EC + NRT versus NRT

| Analysis or subgroup title | No. of studies | No. of participants | Statistical method | Effect size |
| --- | --- | --- | --- | --- |
| 20.1 Smoking cessation | 2 | 980 | Risk Ratio (M-H, Random, 95% CI) | 3.57 [1.96, 6.51] |
| 20.2 Adverse events | 4 |  | Risk Ratio (M-H, Random, 95% CI) | Totals not selected |
| 20.3 Serious adverse events | 5 | 2352 | Risk Ratio (M-H, Random, 95% CI) | 1.24 [0.45, 3.41] |

## Analysis group 21: Nicotine EC + varenicline vs. varenicline

| Analysis or subgroup title | No. of studies | No. of participants | Statistical method | Effect size |
| --- | --- | --- | --- | --- |
| 21.1 Adverse events | 1 | 92 | Risk Ratio (M-H, Random, 95% CI) | 1.18 [0.84, 1.67] |
| 21.2 Serious adverse events | 1 | 92 | Risk Ratio (M-H, Random, 95% CI) | Not estimable |

## Analysis group 22: Nicotine EC + VLNC versus VLNC

| Analysis or subgroup title | No. of studies | No. of participants | Statistical method | Effect size |
| --- | --- | --- | --- | --- |
| 22.1 Adverse events | 1 | 243 | Risk Ratio (M-H, Random, 95% CI) | 0.97 [0.88, 1.07] |
| 22.2 Serious adverse events | 1 | 243 | Risk Ratio (M-H, Random, 95% CI) | 0.81 [0.23, 2.78] |
| 22.3 Change in carbon monoxide | 1 | 132 | Mean Difference (IV, Random, 95% CI) | −7.15 [−13.07, −1.23] |
| 22.4 Change in NNAL (pmol/mg creatinine) | 1 | 110 | Mean Difference (IV, Random, 95% CI) | 0.29 [−1.83, 2.41] |

# Figures and tables

Analysis 1.1: Smoking cessation


Study or Subgroup

1.1.1 Not selected on pregnancy
Bullen 2013
Hajek 2019
Klonizakis 2022
Kouroutzoglou 2024
Lee 2018
Myers-Smith 2022

Russell 2021\*
a

Russell 2021\*
b
Vojjala 2025

Subtotal
Total events:
Test for overall effect: Z = 4.47 (P < 0.00001)

Heterogeneity: Chi² = 7.89, df = 8 (P = 0.44); I² = 0%

1.1.2 Pregnant population

Hajek 2022
c

Subtotal
Total events:
Test for overall effect: Z = 0.82 (P = 0.41)

Heterogeneity: Not applicable

Total
Total events:
Test for overall effect: Z = 4.55 (P < 0.00001)
Test for subgroup differences: Chi² = 0.04, df = 1 (P = 0.85), I² = 0%

Heterogeneity: Chi² = 7.95, df = 9 (P = 0.54); I² = 0%

EC
Events
21
79
36
8
5
13
34
44
7
247
6
6
253
Total
289
438
84
19
20
68
140
145
63

1266
169

169

1435

NRT
Events
17
44
25
5
1
2
15
15
7
131
3
3
134
Total
295
446
82
19
10
67
70
71
58

1118
150

150

1268
Weight
11.6%
30.1%
17.5%
3.5%
0.9%
1.4%
13.8%
13.9%
5.0%

97.8%
2.2%

2.2%

100.0%

Risk Ratio
M-H, Fixed, 95% CI
1.26 [0.68 , 2.34]
1.83 [1.30 , 2.58]
1.41 [0.93 , 2.12]
1.60 [0.64 , 4.01]
2.50 [0.34 , 18.63]
6.40 [1.50 , 27.30]
1.13 [0.66 , 1.94]
1.44 [0.86 , 2.40]
0.92 [0.34 , 2.47]

1.55 [1.28 , 1.87]
1.78 [0.45 , 6.97]

1.78 [0.45 , 6.97]

1.55 [1.28 , 1.88]

Risk Ratio
M-H, Fixed, 95% CI


0.01

0.1

1

10

100


Favours NRT

Favours EC


Risk of Bias
A

+

+

+

?

+

+

?

?

+

+
B

+

+

+

?

+

+

?

?

−

+
C

+

+

+

+

+

+

+

+

+

+
D

+

+

+

?

+

+

+

+

−

+
E

+

+

+

?

+

+

+

+

+

+
F

+

+

?

?

+

+

?

?

+

+
G


Footnotes

a
FBNPs EC arm; control group split to avoid double-counting

b
NSP EC arm; control group split to avoid double-counting

c
This is a subset of data from participants followed up for 6 months or longer
Risk of bias legend

(A) Random sequence generation (selection bias)

(B) Allocation concealment (selection bias)

(C) Blinding of participants and personnel (performance bias)

(D) Blinding of outcome assessment (detection bias)

(E) Incomplete outcome data (attrition bias)

(F) Selective reporting (reporting bias)

(G) Other bias


Analysis 1.2: Adverse events


Study or Subgroup
Bullen 2013
Hajek 2022

Lee 2018
a
Myers-Smith 2022

Piper 2025
b
Smith 2025
Wagener 2023

Total (HKSJ
c
)
Total events:
Test for overall effect: T = 0.02, df = 6 (P = 0.98)

Heterogeneity: Tau² (DL
d
) = 0.04; Chi² = 14.13, df = 6 (P = 0.03); I² = 58%

Nicotine EC
Events
107
124
7
4
53
8
93
396
Total
241
556
19
60
108
18
175

1177

NRT
Events
96
118
5
2
16
10
87
334
Total
215
554
10
47
53
10
175

1064
Weight
23.7%
22.8%
5.0%
1.5%
12.6%
10.6%
23.8%

100.0%

Risk Ratio
M-H, Random, 95% CI
0.99 [0.81 , 1.22]
1.05 [0.84 , 1.31]
0.74 [0.31 , 1.73]
1.57 [0.30 , 8.19]
1.63 [1.03 , 2.56]
0.47 [0.28 , 0.79]
1.07 [0.87 , 1.31]

1.00 [0.73 , 1.37]

Risk Ratio
M-H, Random, 95% CI


0.01

0.1

1

10

100


Favours EC

Favours NRT


Risk of Bias
A

+

+

+

+

+

?

?
B

+

+

+

+

?

?

?
C

+

+

+

+

−

+

?
D

+

+

+

+

−

+

?
E

+

+

+

+

+

+

?
F

+

+

+

+

+

+

?
G


?

Footnotes

a
Data at 4 weeks post-operation; time from baseline not defined and likely to differ between participants

b
Intervention arm contains data from EC + placebo patch and EC + no patch study arms

c
CI calculated by Hartung-Knapp-Sidik-Jonkman (HKSJ) method.

d
Tau² calculated by DerSimonian and Laird method.
Risk of bias legend

(A) Random sequence generation (selection bias)

(B) Allocation concealment (selection bias)

(C) Blinding of participants and personnel (performance bias)

(D) Blinding of outcome assessment (detection bias)

(E) Incomplete outcome data (attrition bias)

(F) Selective reporting (reporting bias)

(G) Other bias


Analysis 1.3: Serious adverse events


Study or Subgroup
Bullen 2013
Hajek 2019
Hajek 2022

Lee 2018
a
Myers-Smith 2022

Piper 2025
b
Smith 2025
Wagener 2023

Total (HKSJ
c
)
Total events:
Test for overall effect: T = 1.06, df = 4 (P = 0.35)

Heterogeneity: Tau² (DL
d
) = 0.05; Chi² = 5.70, df = 4 (P = 0.22); I² = 30%

EC
Events
24
27
31
0
0
0
0
18
100
Total
241
356
564
19
60
108
18
175

1541

NRT
Events
14
19
37
0
0
0
1
10
81
Total
215
342
557
10
47
53
10
175

1409
Weight
22.4%
25.8%
32.7%
1.3%
17.8%

100.0%

Risk Ratio
M-H, Random, 95% CI
1.53 [0.81 , 2.88]
1.37 [0.77 , 2.41]
0.83 [0.52 , 1.31]
Not estimable
Not estimable
Not estimable
0.19 [0.01 , 4.34]
1.80 [0.86 , 3.79]

1.22 [0.73 , 2.03]

Risk Ratio
M-H, Random, 95% CI


0.01

0.1

1

10

100


Favours EC

Favours NRT


Risk of Bias
A

+

+

+

+

+

+

?

?
B

+

+

+

+

+

?

?

?
C

+

+

+

+

+

−

+

?
D

+

+

+

+

+

−

+

?
E

+

+

+

+

+

+

+

?
F

+

+

+

+

+

+

+

?
G


?

Footnotes

a
Data at 4 weeks post-operation; time from baseline not defined and likely to differ between participants

b
Intervention arm contains data from EC + placebo patch and EC + no patch study arms

c
CI calculated by Hartung-Knapp-Sidik-Jonkman (HKSJ) method.

d
Tau² calculated by DerSimonian and Laird method.
Risk of bias legend

(A) Random sequence generation (selection bias)

(B) Allocation concealment (selection bias)

(C) Blinding of participants and personnel (performance bias)

(D) Blinding of outcome assessment (detection bias)

(E) Incomplete outcome data (attrition bias)

(F) Selective reporting (reporting bias)

(G) Other bias


Analysis 1.4: Carbon monoxide (ppm)


Study or Subgroup

1.4.1 Absolute values at follow-up
Hatsukami 2020

Subtotal
Test for overall effect: Z = 1.12 (P = 0.26)

Heterogeneity: Not applicable

1.4.2 Change from baseline
Kerr 2020
Klonizakis 2022
Lee 2018
Smith 2025

Subtotal (Wald
a
)
Test for overall effect: Z = 1.75 (P = 0.08)

Heterogeneity: Tau² (REML
b
) = 3.45; Chi² = 4.01, df = 3 (P = 0.26); I² = 29%

Total (Wald
a
)
Test for overall effect: Z = 2.15 (P = 0.03)
Test for subgroup differences: Chi² = 0.20, df = 1 (P = 0.65), I² = 0%

Heterogeneity: Tau² (REML
b
) = 0.00; Chi² = 4.01, df = 4 (P = 0.40); I² = 0%

Nicotine EC
Mean
11.02
-10
-9
-2.1
-19.4
SD
8.96
10
9.35
12.2
19.9
Total
58

58
28
84
18
18

148

206

NRT
Mean
12.89
-7
-8
7.1
-11.9
SD
8.59
10
6.93
11
8.4
Total
52

52
27
82
8
10

127

179
Weight
30.1%

30.1%
11.6%
51.8%
3.6%
2.9%

69.9%

100.0%

Mean Difference
IV, Random, 95% CI
-1.87 [-5.15 , 1.41]

-1.87 [-5.15 , 1.41]
-3.00 [-8.29 , 2.29]
-1.00 [-3.50 , 1.50]
-9.20 [-18.68 , 0.28]
-7.50 [-18.07 , 3.07]

-2.93 [-6.20 , 0.34]

-1.98 [-3.78 , -0.18]

Mean Difference
IV, Random, 95% CI


-20

-10

0

10

20


Favours EC

Favours NRT


Footnotes

a
CI calculated by Wald-type method.

b
Tau² calculated by Restricted Maximum-Likelihood method.


Analysis 1.5: Heart rate (bpm)


Study or Subgroup

1.5.1 Absolute values at follow-up
Hatsukami 2020

Subtotal
Test for overall effect: Z = 0.33 (P = 0.74)

Heterogeneity: Not applicable

1.5.2 Change from baseline
Kerr 2020

Subtotal
Test for overall effect: Z = 0.73 (P = 0.47)

Heterogeneity: Not applicable

Total (Wald
a
)
Test for overall effect: Z = 0.45 (P = 0.65)
Test for subgroup differences: Chi² = 0.43, df = 1 (P = 0.51), I² = 0%

Heterogeneity: Tau² (REML
b
) = 0.00; Chi² = 0.43, df = 1 (P = 0.51); I² = 0%

Nicotine EC
Mean
74.81
0
SD
13.91
6
Total
58

58
28

28

86

NRT
Mean
75.55
-1
SD
9.72
4
Total
53

53
27

27

80
Weight
26.8%

26.8%
73.2%

73.2%

100.0%

Mean Difference
IV, Random, 95% CI
-0.74 [-5.17 , 3.69]

-0.74 [-5.17 , 3.69]
1.00 [-1.69 , 3.69]

1.00 [-1.69 , 3.69]

0.53 [-1.76 , 2.83]

Mean Difference
IV, Random, 95% CI


-4

-2

0

2

4


Favours EC

Favours NRT


Footnotes

a
CI calculated by Wald-type method.

b
Tau² calculated by Restricted Maximum-Likelihood method.


Analysis 1.6: Systolic blood pressure


Study or Subgroup

1.6.1 Absolute values at follow-up
Hatsukami 2020

Subtotal
Test for overall effect: Z = 0.35 (P = 0.72)

Heterogeneity: Not applicable

1.6.2 Change from baseline
Kerr 2020

Subtotal
Test for overall effect: Z = 1.85 (P = 0.06)

Heterogeneity: Not applicable

Total (Wald
a
)
Test for overall effect: Z = 1.61 (P = 0.11)
Test for subgroup differences: Chi² = 0.98, df = 1 (P = 0.32), I² = 0%

Heterogeneity: Tau² (REML
b
) = 0.00; Chi² = 0.98, df = 1 (P = 0.32); I² = 0%

Nicotine EC
Mean
123.1
-4
SD
13.3
4
Total
58

58
28

28

86

NRT
Mean
122.1
-2
SD
16.2
4
Total
53

53
27

27

80
Weight
12.7%

12.7%
87.3%

87.3%

100.0%

Mean Difference
IV, Random, 95% CI
1.00 [-4.54 , 6.54]

1.00 [-4.54 , 6.54]
-2.00 [-4.11 , 0.11]

-2.00 [-4.11 , 0.11]

-1.62 [-3.59 , 0.36]

Mean Difference
IV, Random, 95% CI


-4

-2

0

2

4


Favours EC

Favours NRT


Footnotes

a
CI calculated by Wald-type method.

b
Tau² calculated by Restricted Maximum-Likelihood method.


Analysis 1.7: Blood oxygen saturation


Study or Subgroup

1.7.1 Absolute values at follow-up
Hatsukami 2020

Subtotal
Test for overall effect: Z = 0.75 (P = 0.45)

Heterogeneity: Not applicable

1.7.2 Change from baseline
Kerr 2020

Subtotal
Test for overall effect: Z = 0.00 (P = 1.00)

Heterogeneity: Not applicable

Total (Wald
a
)
Test for overall effect: Z = 0.63 (P = 0.53)
Test for subgroup differences: Chi² = 0.16, df = 1 (P = 0.69), I² = 0%

Heterogeneity: Tau² (REML
b
) = 0.00; Chi² = 0.16, df = 1 (P = 0.69); I² = 0%

Nicotine EC
Mean
98.1
0
SD
1.5
2
Total
57

57
28

28

85

NRT
Mean
98.3
0
SD
1.3
1
Total
53

53
27

27

80
Weight
71.6%

71.6%
28.4%

28.4%

100.0%

Mean Difference
IV, Random, 95% CI
-0.20 [-0.72 , 0.32]

-0.20 [-0.72 , 0.32]
0.00 [-0.83 , 0.83]

0.00 [-0.83 , 0.83]

-0.14 [-0.59 , 0.30]

Mean Difference
IV, Random, 95% CI


-0.5

-0.25

0

0.25

0.5


Favours NRT

Favours EC


Footnotes

a
CI calculated by Wald-type method.

b
Tau² calculated by Restricted Maximum-Likelihood method.


Analysis 1.8: 3-HPMA (pmol/mg creatinine)


Study or Subgroup

1.8.1 Absolute values at follow-up
Hatsukami 2020

Subtotal
Test for overall effect: Z = 0.90 (P = 0.37)

Heterogeneity: Not applicable

Total
Test for overall effect: Z = 0.90 (P = 0.37)
Test for subgroup differences: Not applicable

Heterogeneity: Not applicable

Nicotine EC
Mean
4612
SD
4263
Total
58

58

58

NRT
Mean
5419
SD
5076
Total
53

53

53
Weight
100.0%

100.0%

100.0%

Mean Difference
IV, Random, 95% CI
-807.00 [-2559.47 , 945.47]

-807.00 [-2559.47 , 945.47]

-807.00 [-2559.47 , 945.47]

Mean Difference
IV, Random, 95% CI


-1000

-500

0

500

1000


Favours EC

Favours NRT


Analysis 1.9: NNAL (pmol/mg creatinine)


Study or Subgroup

1.9.1 Absolute values at follow-up
Hatsukami 2020

Subtotal
Test for overall effect: Z = 0.00 (P = 1.00)

Heterogeneity: Not applicable

Total
Test for overall effect: Z = 0.00 (P = 1.00)
Test for subgroup differences: Not applicable

Heterogeneity: Not applicable

Nicotine EC
Mean
1.2
SD
1.7
Total
57

57

57

NRT
Mean
1.2
SD
1.1
Total
53

53

53
Weight
100.0%

100.0%

100.0%

Mean Difference
IV, Random, 95% CI
0.00 [-0.53 , 0.53]

0.00 [-0.53 , 0.53]

0.00 [-0.53 , 0.53]

Mean Difference
IV, Random, 95% CI


-1

-0.5

0

0.5

1


Favours EC

Favours NRT


Analysis 1.10: 2-HPMA (pmol/mg creatinine)


Study or Subgroup

1.10.1 Absolute values at follow-up
Hatsukami 2020

Subtotal
Test for overall effect: Z = 0.58 (P = 0.56)

Heterogeneity: Not applicable

Total
Test for overall effect: Z = 0.58 (P = 0.56)
Test for subgroup differences: Not applicable

Heterogeneity: Not applicable

Nicotine EC
Mean
733.2
SD
855.6
Total
58

58

58

NRT
Mean
842.2
SD
1083.7
Total
53

53

53
Weight
100.0%

100.0%

100.0%

Mean Difference
IV, Random, 95% CI
-109.00 [-474.52 , 256.52]

-109.00 [-474.52 , 256.52]

-109.00 [-474.52 , 256.52]

Mean Difference
IV, Random, 95% CI


-200

-100

0

100

200


Favours EC

Favours NRT


Analysis 1.11: HMPMA (pmol/mg creatinine)


Study or Subgroup

1.11.1 Absolute values at follow-up
Hatsukami 2020

Subtotal
Test for overall effect: Z = 1.20 (P = 0.23)

Heterogeneity: Not applicable

Total
Test for overall effect: Z = 1.20 (P = 0.23)
Test for subgroup differences: Not applicable

Heterogeneity: Not applicable

Nicotine EC
Mean
3959
SD
3633
Total
58

58

58

NRT
Mean
4834
SD
3999
Total
53

53

53
Weight
100.0%

100.0%

100.0%

Mean Difference
IV, Random, 95% CI
-875.00 [-2300.93 , 550.93]

-875.00 [-2300.93 , 550.93]

-875.00 [-2300.93 , 550.93]

Mean Difference
IV, Random, 95% CI


-1000

-500

0

500

1000


Favours EC

Favours NRT


Analysis 1.12: PheT (pmol/mg creatinine)


Study or Subgroup

1.12.1 Absolute values at follow-up
Hatsukami 2020

Subtotal
Test for overall effect: Z = 0.73 (P = 0.46)

Heterogeneity: Not applicable

Total
Test for overall effect: Z = 0.73 (P = 0.46)
Test for subgroup differences: Not applicable

Heterogeneity: Not applicable

Nicotine EC
Mean
2.9
SD
2.8
Total
56

56

56

NRT
Mean
2.5
SD
2.9
Total
53

53

53
Weight
100.0%

100.0%

100.0%

Mean Difference
IV, Random, 95% CI
0.40 [-0.67 , 1.47]

0.40 [-0.67 , 1.47]

0.40 [-0.67 , 1.47]

Mean Difference
IV, Random, 95% CI


-1

-0.5

0

0.5

1


Favours EC

Favours NRT


Analysis 1.13: CEMA (pmol/mg creatinine)


Study or Subgroup

1.13.1 Absolute values at follow-up
Hatsukami 2020

Subtotal
Test for overall effect: Z = 0.46 (P = 0.65)

Heterogeneity: Not applicable

Total
Test for overall effect: Z = 0.46 (P = 0.65)
Test for subgroup differences: Not applicable

Heterogeneity: Not applicable

Nicotine EC
Mean
512
SD
443
Total
58

58

58

NRT
Mean
475
SD
409
Total
53

53

53
Weight
100.0%

100.0%

100.0%

Mean Difference
IV, Random, 95% CI
37.00 [-121.50 , 195.50]

37.00 [-121.50 , 195.50]

37.00 [-121.50 , 195.50]

Mean Difference
IV, Random, 95% CI


-100

-50

0

50

100


Favours EC

Favours NRT


Analysis 1.14: AAMA (pmol/mg creatinine)


Study or Subgroup

1.14.1 Absolute values at follow-up
Hatsukami 2020

Subtotal
Test for overall effect: Z = 0.44 (P = 0.66)

Heterogeneity: Not applicable

Total
Test for overall effect: Z = 0.44 (P = 0.66)
Test for subgroup differences: Not applicable

Heterogeneity: Not applicable

Nicotine EC
Mean
495.2
SD
390.9
Total
58

58

58

NRT
Mean
463.2
SD
361.8
Total
51

51

51
Weight
100.0%

100.0%

100.0%

Mean Difference
IV, Random, 95% CI
32.00 [-109.35 , 173.35]

32.00 [-109.35 , 173.35]

32.00 [-109.35 , 173.35]

Mean Difference
IV, Random, 95% CI


-500

-250

0

250

500


Favours EC

Favours NRT


Analysis 1.15: FEV1


Study or Subgroup

1.15.1 Change from baseline
Kerr 2020
Lee 2018

Nicotine EC
Mean
-0.04
292
SD
0.14
503
Total
28
18

NRT
Mean
0.03
-300
SD
0.14
549
Total
27
8

Std. Mean Difference
IV, Random, 95% CI
-0.49 [-1.03 , 0.04]
1.11 [0.21 , 2.00]

Std. Mean Difference
IV, Random, 95% CI


-2

-1

0

1

2


Favours NRT

Favours nicotine EC


Analysis 1.16: FEV1/FVC (%)


Study or Subgroup

1.16.1 Change from baseline
Kerr 2020
Lee 2018

Subtotal (Wald
a
)
Test for overall effect: Z = 0.58 (P = 0.56)

Heterogeneity: Tau² (REML
b
) = 416.58; Chi² = 2.05, df = 1 (P = 0.15); I² = 51%

Total (Wald
a
)
Test for overall effect: Z = 0.58 (P = 0.56)
Test for subgroup differences: Not applicable

Heterogeneity: Tau² (REML
b
) = 416.58; Chi² = 2.05, df = 1 (P = 0.15); I² = 51%

Nicotine EC
Mean
-0.2
2
SD
2.6
10.5
Total
28
18

46

46

NRT
Mean
0
-38.1
SD
3.6
79.2
Total
27
8

35

35
Weight
74.3%
25.7%

100.0%

100.0%

Mean Difference
IV, Random, 95% CI
-0.20 [-1.86 , 1.46]
40.10 [-15.00 , 95.20]

10.15 [-24.36 , 44.67]

10.15 [-24.36 , 44.67]

Mean Difference
IV, Random, 95% CI


-50

-25

0

25

50


Favours NRT

Favours nicotine EC


Footnotes

a
CI calculated by Wald-type method.

b
Tau² calculated by Restricted Maximum-Likelihood method.


Analysis 1.17: PEF (L/min)


Study or Subgroup

1.17.1 Change from baseline
Kerr 2020

Subtotal
Test for overall effect: Z = 0.24 (P = 0.81)

Heterogeneity: Not applicable

Total
Test for overall effect: Z = 0.24 (P = 0.81)
Test for subgroup differences: Not applicable

Heterogeneity: Not applicable

Nicotine EC
Mean
2
SD
49
Total
28

28

28

NRT
Mean
5
SD
42
Total
27

27

27
Weight
100.0%

100.0%

100.0%

Mean Difference
IV, Random, 95% CI
-3.00 [-27.09 , 21.09]

-3.00 [-27.09 , 21.09]

-3.00 [-27.09 , 21.09]

Mean Difference
IV, Random, 95% CI


-100

-50

0

50

100


Favours NRT

Favours nicotine EC


Analysis 1.18: Product use at 6+ months


Study or Subgroup
Bullen 2013
Hajek 2019
Lee 2018
Myers-Smith 2022

Russell 2021\*
a

Russell 2021\*
b

EC
Events
71
173
3
32
62
48
Total
241
356
18
59
124
103

NRT
Events
17
19
2
7
28
28
Total
215
342
9
47
61
60

Risk Ratio
M-H, Random, 95% CI
3.73 [2.27 , 6.12]
8.75 [5.58 , 13.72]
0.75 [0.15 , 3.72]
3.64 [1.77 , 7.50]
1.09 [0.79 , 1.51]
1.00 [0.71 , 1.40]

Risk Ratio
M-H, Random, 95% CI


0.05

0.2

1

5

20


Higher with NRT

Higher with EC

Footnotes

a
NSP EC arm; control arm split to avoid double-counting. Study product use and numbers at  by follow up correspondence.

b
FBNP EC arm; control group split to avoid double-counting. Study product use and numbers at follow up by correspondence.


Analysis 2.1: Smoking cessation


Study or Subgroup
Ioakeimidis 2018

Total
Total events:
Test for overall effect: Z = 2.34 (P = 0.02)

Heterogeneity: Not applicable

Nicotine EC
Events
4
4
Total
27

27

Varenicline
Events
13
13
Total
27

27
Weight
100.0%

100.0%

Risk Ratio
M-H, Random, 95% CI
0.31 [0.11 , 0.82]

0.31 [0.11 , 0.82]

Risk Ratio
M-H, Random, 95% CI


0.01

0.1

1

10

100


Favours varenicline

Favours nicotine EC


Risk of Bias
A

?
B

?
C

+
D

+
E

?
F

?
G

−

Risk of bias legend

(A) Random sequence generation (selection bias)

(B) Allocation concealment (selection bias)

(C) Blinding of participants and personnel (performance bias)

(D) Blinding of outcome assessment (detection bias)

(E) Incomplete outcome data (attrition bias)

(F) Selective reporting (reporting bias)

(G) Other bias


Analysis 2.2: Serious adverse events


Study or Subgroup

Ioakeimidis 2018
a

Total
Total events:
Test for overall effect: Not applicable

Heterogeneity: Not applicable

Nicotine EC
Events
0
0
Total
27

27

Varenicline
Events
0
0
Total
27

27
Weight

Risk Ratio
M-H, Random, 95% CI
Not estimable

Not estimable

Risk Ratio
M-H, Random, 95% CI


0.01

0.1

1

10

100


Favours EC

Favours varenicline

Footnotes

a
n followed up not reported; n randomized used as denominators


Analysis 3.1: Smoking cessation


Study or Subgroup

Tuisku 2024
a

Total
Total events:
Test for overall effect: Z = 1.89 (P = 0.06)

Heterogeneity: Not applicable

EC
Events
42
42
Total
152

152

non-nicotine EC + varenicline
Events
58
58
Total
153

153
Weight
100.0%

100.0%

Risk Ratio
M-H, Random, 95% CI
0.73 [0.53 , 1.01]

0.73 [0.53 , 1.01]

Risk Ratio
M-H, Random, 95% CI


0.01

0.1

1

10

100


Favours non-nicotine EC + varenicline

Favours EC


Footnotes

a
The EC group also received placebo 'varenicline' pills


Analysis 3.2: Serious adverse events


Study or Subgroup

Tuisku 2024
a

Total
Total events:
Test for overall effect: Z = 1.05 (P = 0.30)

Heterogeneity: Not applicable

EC
Events
2
2
Total
152

152

non-nicotine EC + varenicline
Events
0
0
Total
153

153
Weight
100.0%

100.0%

Risk Ratio
M-H, Random, 95% CI
5.03 [0.24 , 103.97]

5.03 [0.24 , 103.97]

Risk Ratio
M-H, Random, 95% CI


0.01

0.1

1

10

100


Favours EC

Favours non-nicotine EC + varenicline


Footnotes

a
The EC group also received placebo 'varenicline' pills


Analysis 4.1: Smoking cessation


Study or Subgroup
Kouroutzoglou 2024

Total
Total events:
Test for overall effect: Z = 0.33 (P = 0.74)

Heterogeneity: Not applicable

EC
Events
8
8
Total
19

19

NRT + bupropion
Events
9
9
Total
19

19
Weight
100.0%

100.0%

Risk Ratio
M-H, Random, 95% CI
0.89 [0.44 , 1.81]

0.89 [0.44 , 1.81]

Risk Ratio
M-H, Random, 95% CI


0.01

0.1

1

10

100


Favours NRT + bupropion

Favours EC


Analysis 5.1: Adverse events


Study or Subgroup
Caponnetto 2023\*

Total
Total events:
Test for overall effect: Z = 1.21 (P = 0.22)

Heterogeneity: Not applicable

Nicotine EC
Events
56
56
Total
110

110

Heated tobacco
Events
65
65
Total
110

110
Weight
100.0%

100.0%

Risk Ratio
M-H, Random, 95% CI
0.86 [0.68 , 1.10]

0.86 [0.68 , 1.10]

Risk Ratio
M-H, Random, 95% CI


0.01

0.1

1

10

100


Favours nicotine EC

Favours heated tobacco


Analysis 5.2: SAEs


Study or Subgroup
Caponnetto 2023\*

Total
Total events:
Test for overall effect: Not applicable

Heterogeneity: Not applicable

Nicotine EC
Events
0
0
Total
110

110

Heated tobacco
Events
0
0
Total
110

110
Weight

Risk Ratio
M-H, Random, 95% CI
Not estimable

Not estimable

Risk Ratio
M-H, Random, 95% CI


0.01

0.1

1

10

100


Favours nicotine EC

Favours heated tobacco


Analysis 5.3: Carbon monoxide (ppm)


Study or Subgroup

5.3.1 Absolute values at 12 weeks
Caponnetto 2023\*

Subtotal
Test for overall effect: Z = 1.43 (P = 0.15)

Heterogeneity: Not applicable

5.3.2 Change at 1 month
Ikonomidis 2024

Subtotal
Test for overall effect: Z = 0.13 (P = 0.90)

Heterogeneity: Not applicable

Total (Wald
a
)
Test for overall effect: Z = 0.96 (P = 0.34)
Test for subgroup differences: Chi² = 1.06, df = 1 (P = 0.30), I² = 5.6%

Heterogeneity: Tau² (REML
b
) = 0.12; Chi² = 1.06, df = 1 (P = 0.30); I² = 6%

Nicotine EC
Mean
10.1
-7.9
SD
11.3
5.1
Total
107

107
25

25

132

Heated tobacco
Mean
8.2
-7.7
SD
8
5.8
Total
110

110
25

25

135
Weight
56.9%

56.9%
43.1%

43.1%

100.0%

Mean Difference
IV, Random, 95% CI
1.90 [-0.71 , 4.51]

1.90 [-0.71 , 4.51]
-0.20 [-3.23 , 2.83]

-0.20 [-3.23 , 2.83]

1.00 [-1.04 , 3.03]

Mean Difference
IV, Random, 95% CI


-20

-10

0

10

20


Favours nicotine EC

Favours heated tobacco


Footnotes

a
CI calculated by Wald-type method.

b
Tau² calculated by Restricted Maximum-Likelihood method.


Analysis 5.4: VO2 Max at 12 weeks


Study or Subgroup
Caponnetto 2023\*

Total
Test for overall effect: Z = 1.48 (P = 0.14)

Heterogeneity: Not applicable

Nicotine EC
Mean
50.9
SD
31.9
Total
101

101

Heated tobacco
Mean
44.7
SD
28.7
Total
110

110
Weight
100.0%

100.0%

Mean Difference
IV, Random, 95% CI
6.20 [-2.01 , 14.41]

6.20 [-2.01 , 14.41]

Mean Difference
IV, Random, 95% CI


-100

-50

0

50

100


Favours heated tobacco

Favours nicotine EC


Analysis 6.1: Serious adverse events


Study or Subgroup
Avila 2024

Total
Total events:
Test for overall effect: Not applicable

Heterogeneity: Not applicable

Nicotine EC
Events
0
0
Total
14

14

ONP
Events
0
0
Total
12

12
Weight

Risk Ratio
M-H, Random, 95% CI
Not estimable

Not estimable

Risk Ratio
M-H, Random, 95% CI


0.01

0.1

1

10

100


Favours nicotine  EC

Favours ONP


Analysis 6.2: Carbon monoxide (ppm)


Study or Subgroup

6.2.1 Change from baseline
Avila 2024

Subtotal
Test for overall effect: Z = 1.49 (P = 0.14)

Heterogeneity: Not applicable

Total
Test for overall effect: Z = 1.49 (P = 0.14)
Test for subgroup differences: Not applicable

Heterogeneity: Not applicable

Nicotine EC
Mean [ppm]
-15.36
SD [ppm]
22.7
Total
14

14

14

ONP
Mean [ppm]
-2.92
SD [ppm]
19.9
Total
12

12

12
Weight
100.0%

100.0%

100.0%

Mean Difference
IV, Random, 95% CI [ppm]
-12.44 [-28.82 , 3.94]

-12.44 [-28.82 , 3.94]

-12.44 [-28.82 , 3.94]

Mean Difference
IV, Random, 95% CI [ppm]


-100

-50

0

50

100


Favours nicotine EC

Favours ONP


Analysis 7.1: Smoking cessation


Study or Subgroup
Bullen 2013
Caponnetto 2013a\*

Cobb 2021
a

Cobb 2021
b
Eisenberg 2020
Klonizakis 2022
Lucchiari 2022
Tuisku 2024

Total (Wald
c
)
Total events:
Test for overall effect: Z = 2.46 (P = 0.01)

Heterogeneity: Tau² (DL
d
) = 0.00; Chi² = 6.14, df = 7 (P = 0.52); I² = 0%

Nicotine EC
Events
21
22
10
4
5
36
15
42
155
Total
289
200
130
130
128
84
70
152

1183

Non-nicotine EC
Events
3
4
1
0
3
30
15
30
86
Total
73
100
65
65
127
82
70
153

735
Weight
4.0%
5.2%
1.3%
0.7%
2.8%
39.2%
13.9%
33.0%

100.0%

Risk Ratio
M-H, Random, 95% CI
1.77 [0.54 , 5.77]
2.75 [0.97 , 7.76]
5.00 [0.65 , 38.22]
4.53 [0.25 , 82.96]
1.65 [0.40 , 6.77]
1.17 [0.80 , 1.71]
1.00 [0.53 , 1.89]
1.41 [0.93 , 2.13]

1.34 [1.06 , 1.70]

Risk Ratio
M-H, Random, 95% CI


0.01

0.1

1

10

100


Favours non-nicotine EC

Favours nicotine EC


Risk of Bias
A

+

+

+

+

+

+

+

+
B

+

+

+

+

+

+

+

+
C

+

+

+

+

+

+

+

+
D

+

+

+

+

+

+

+

+
E

+

+

+

+

+

+

+

+
F

+

?

+

+

+

?

−

+
G


Footnotes

a
36 mg/mL arm; control group split to avoid double-counting

b
8 mg/mL arm; control group split to avoid double-counting

c
CI calculated by Wald-type method.

d
Tau² calculated by DerSimonian and Laird method.
Risk of bias legend

(A) Random sequence generation (selection bias)

(B) Allocation concealment (selection bias)

(C) Blinding of participants and personnel (performance bias)

(D) Blinding of outcome assessment (detection bias)

(E) Incomplete outcome data (attrition bias)

(F) Selective reporting (reporting bias)

(G) Other bias


Analysis 7.2: Adverse events


Study or Subgroup
Bullen 2013
Eisenberg 2020
Meier 2017
Okuyemi 2022

Rose 2023\*
a

Total (Wald
b
)
Total events:
Test for overall effect: Z = 0.26 (P = 0.79)

Heterogeneity: Tau² (DL
c
) = 0.00; Chi² = 0.34, df = 3 (P = 0.95); I² = 0%

Nicotine EC
Events
107
120
3
0
4
234
Total
241
128
24
109
11

513

Non-nicotine EC
Events
26
118
2
0
4
150
Total
57
127
24
106
13

327
Weight
4.1%
95.4%
0.1%
0.3%

100.0%

Risk Ratio
M-H, Random, 95% CI
0.97 [0.71 , 1.34]
1.01 [0.94 , 1.08]
1.50 [0.27 , 8.19]
Not estimable
1.18 [0.38 , 3.66]

1.01 [0.95 , 1.08]

Risk Ratio
M-H, Random, 95% CI


0.05

0.2

1

5

20


Favours nicotine EC

Favours non-nicotine EC


Risk of Bias
A

+

+

?

?

?
B

+

+

?

?

?
C

+

+

+

+

+
D

+

+

+

+

+
E

+

+

?

+

+
F

+

+

+

+

+
G


+

Footnotes

a
All participants receiving placebo patch

b
CI calculated by Wald-type method.

c
Tau² calculated by DerSimonian and Laird method.
Risk of bias legend

(A) Random sequence generation (selection bias)

(B) Allocation concealment (selection bias)

(C) Blinding of participants and personnel (performance bias)

(D) Blinding of outcome assessment (detection bias)

(E) Incomplete outcome data (attrition bias)

(F) Selective reporting (reporting bias)

(G) Other bias


Analysis 7.3: Serious adverse events


Study or Subgroup
Bullen 2013
Caponnetto 2013a\*

Cobb 2021
a

Cobb 2021
b
Eisenberg 2020
George 2019
Lucchiari 2022
Meier 2017
Okuyemi 2022

Rose 2023\*
c
Tuisku 2024

Total (Wald
d
)
Total events:
Test for overall effect: Z = 0.07 (P = 0.95)

Heterogeneity: Tau² (DL
e
) = 0.00; Chi² = 2.41, df = 5 (P = 0.79); I² = 0%

Nicotine EC
Events
24
0
8
5
3
0
0
0
0
1
2
43
Total
241
72
86
81
128
37
70
24
109
11
152

1011

Non-nicotine EC
Events
4
0
3
4
5
0
0
0
0
0
2
18
Total
57
45
37
37
127
37
70
24
106
13
153

706
Weight
31.2%
20.1%
20.5%
16.3%
3.4%
8.5%

100.0%

Risk Ratio
M-H, Random, 95% CI
1.42 [0.51 , 3.93]
Not estimable
1.15 [0.32 , 4.08]
0.57 [0.16 , 2.00]
0.60 [0.15 , 2.44]
Not estimable
Not estimable
Not estimable
Not estimable
3.50 [0.16 , 78.19]
1.01 [0.14 , 7.05]

0.98 [0.55 , 1.73]

Risk Ratio
M-H, Random, 95% CI


0.01

0.1

1

10

100


Favours nicotine EC

Favours non-nicotine EC


Risk of Bias
A

+

+

+

+

+

+

+

?

?

?

+
B

+

+

+

+

+

+

+

?

?

?

+
C

+

+

+

+

+

?

+

+

+

+

+
D

+

+

+

+

+

−

+

+

+

+

+
E

+

+

+

+

+

?

+

?

+

+

+
F

+

?

+

+

+

+

−

+

+

+

+
G


+


Footnotes

a
36 mg/mL; control group split to avoid double counting

b
8 mg/mL; control group split to avoid double counting

c
All participants receiving placebo patch

d
CI calculated by Wald-type method.

e
Tau² calculated by DerSimonian and Laird method.
Risk of bias legend

(A) Random sequence generation (selection bias)

(B) Allocation concealment (selection bias)

(C) Blinding of participants and personnel (performance bias)

(D) Blinding of outcome assessment (detection bias)

(E) Incomplete outcome data (attrition bias)

(F) Selective reporting (reporting bias)

(G) Other bias


Analysis 7.4: Carbon monoxide (ppm)


Study or Subgroup

7.4.1 Change from baseline

Cobb 2021
a
Klonizakis 2022

Rose 2023\*
b

7.4.2 Absolute values at follow-up

Caponnetto 2013a\*
c
Felicione 2019
Okuyemi 2022

Nicotine EC
Mean
-5.53
-9
-11.1
9.5
26.2
14.2
SD
2.7
9.35
16.6
4.2
11
8
Total
80
84
7
49
14
116

Non-nicotine EC
Mean
-3.88
-6
-2.2
7.3
20.4
15.4
SD
3.1
9.24
13
3.2
7.4
8
Total
69
82
10
41
11
114

Mean Difference
IV, Random, 95% CI
-1.65 [-2.59 , -0.71]
-3.00 [-5.83 , -0.17]
-8.90 [-23.60 , 5.80]
2.20 [0.67 , 3.73]
5.80 [-1.43 , 13.03]
-1.20 [-3.27 , 0.87]

Mean Difference
IV, Random, 95% CI


-20

-10

0

10

20


Favours nicotine EC

Favours non-nicotine EC

Footnotes

a
Data are for 36 mg/mL arm. In 8 mg/mL arm, -4.4, SD 3.1, n = 74

b
Comparison: Nicotine pod + placebo patch vs. non-nicotine pod + placebo patch

c
Data are 2.4% nicotine compared to no-nicotine; 1.8% nicotine arm reported elsewhere


Analysis 7.5: Heart rate


Study or Subgroup

7.5.1 Absolute values at follow-up

Caponnetto 2013a\*
a

Cobb 2021
b

Subtotal (Wald
c
)
Test for overall effect: Z = 1.04 (P = 0.30)

Heterogeneity: Tau² (REML
d
) = 0.00; Chi² = 0.48, df = 1 (P = 0.49); I² = 0%

Total (Wald
c
)
Test for overall effect: Z = 1.04 (P = 0.30)
Test for subgroup differences: Not applicable

Heterogeneity: Tau² (REML
d
) = 0.00; Chi² = 0.48, df = 1 (P = 0.49); I² = 0%

Nicotine EC
Mean
77.5
79.33
SD
12.2
11.65
Total
73
130

203

203

Non-nicotine EC
Mean
79.8
79.93
SD
10.8
12.38
Total
68
130

198

198
Weight
37.2%
62.8%

100.0%

100.0%

Mean Difference
IV, Random, 95% CI
-2.30 [-6.10 , 1.50]
-0.60 [-3.52 , 2.32]

-1.23 [-3.55 , 1.08]

-1.23 [-3.55 , 1.08]

Mean Difference
IV, Random, 95% CI


-20

-10

0

10

20


Favours nicotine EC

Favours non-nicotine EC


Footnotes

a
Data are 2.4% nicotine compared to no-nicotine; 1.8% nicotine arm reported elsewhere

b
Comparing highest (36mg) dose EC arm to no-nicotine EC arm; N at follow-up not provided, so N at baseline used

c
CI calculated by Wald-type method.

d
Tau² calculated by Restricted Maximum-Likelihood method.


Analysis 7.6: Systolic blood pressure


Study or Subgroup

7.6.1 Absolute values at follow-up

Caponnetto 2013a\*
a

Cobb 2021
b

Subtotal (Wald
c
)
Test for overall effect: Z = 1.66 (P = 0.10)

Heterogeneity: Tau² (REML
d
) = 0.00; Chi² = 0.35, df = 1 (P = 0.55); I² = 0%

Total (Wald
c
)
Test for overall effect: Z = 1.66 (P = 0.10)
Test for subgroup differences: Not applicable

Heterogeneity: Tau² (REML
d
) = 0.00; Chi² = 0.35, df = 1 (P = 0.55); I² = 0%

Nicotine EC
Mean
124.1
125.85
SD
17.7
14.82
Total
73
130

203

203

Non-nicotine EC
Mean
122.9
122.74
SD
13.6
14.56
Total
68
130

198

198
Weight
32.1%
67.9%

100.0%

100.0%

Mean Difference
IV, Random, 95% CI
1.20 [-3.99 , 6.39]
3.11 [-0.46 , 6.68]

2.50 [-0.45 , 5.44]

2.50 [-0.45 , 5.44]

Mean Difference
IV, Random, 95% CI


-20

-10

0

10

20


Favours nicotine EC

Favours non-nicotine EC


Footnotes

a
Data are 2.4% nicotine compared to no-nicotine; 1.8% nicotine arm reported elsewhere

b
Comparing highest (36mg) dose EC arm to no-nicotine EC arm; N at follow-up not provided, so N at baseline used

c
CI calculated by Wald-type method.

d
Tau² calculated by Restricted Maximum-Likelihood method.


Analysis 7.7: NNAL (pmol/mg creatinine)


Study or Subgroup

7.7.1 Change from baseline

Cobb 2021
a

Subtotal
Test for overall effect: Z = 1.48 (P = 0.14)

Heterogeneity: Not applicable

7.7.2 Absolute values at follow-up
Okuyemi 2022

Subtotal
Test for overall effect: Z = 0.14 (P = 0.89)

Heterogeneity: Not applicable

Total (Wald
b
)
Test for overall effect: Z = 0.61 (P = 0.54)
Test for subgroup differences: Chi² = 2.19, df = 1 (P = 0.14), I² = 54.4%

Heterogeneity: Tau² (REML
c
) = 63.65; Chi² = 2.19, df = 1 (P = 0.14); I² = 54%

Nicotine EC
Mean
-4.23
1.95
SD
72.48
1.47
Total
79

79
109

109

188

Non-nicotine EC
Mean
-19.5
1.98
SD
52.7
1.74
Total
69

69
106

106

175
Weight
27.2%

27.2%
72.8%

72.8%

100.0%

Mean Difference
IV, Random, 95% CI
15.27 [-4.98 , 35.52]

15.27 [-4.98 , 35.52]
-0.03 [-0.46 , 0.40]

-0.03 [-0.46 , 0.40]

4.13 [-9.21 , 17.48]

Mean Difference
IV, Random, 95% CI


-50

-25

0

25

50


Favours nicotine EC

Favours non-nicotine EC


Footnotes

a
Data for 36 mg/mL arm. 8 mg/mL arm -141.46 (SD 259.14), n = 73

b
CI calculated by Wald-type method.

c
Tau² calculated by Restricted Maximum-Likelihood method.


Analysis 7.8: FeNO (ppb)


Study or Subgroup

7.8.1 Change from baseline

Caponnetto 2013a\*
a

Subtotal
Test for overall effect: Z = 8.14 (P < 0.00001)

Heterogeneity: Not applicable

Total
Test for overall effect: Z = 8.14 (P < 0.00001)
Test for subgroup differences: Not applicable

Heterogeneity: Not applicable

Nicotine EC
Mean
2.8
SD
1.7
Total
49

49

49

Non-nicotine EC
Mean
0.45
SD
1
Total
41

41

41
Weight
100.0%

100.0%

100.0%

Mean Difference
IV, Random, 95% CI
2.35 [1.78 , 2.92]

2.35 [1.78 , 2.92]

2.35 [1.78 , 2.92]

Mean Difference
IV, Random, 95% CI


-4

-2

0

2

4


Favours nicotine EC

Favours non-nicotine EC


Footnotes

a
Data are 2.4% nicotine compared to no-nicotine; 1.8% nicotine arm reported elsewhere


Analysis 7.9: FEV1 (l)


Study or Subgroup

7.9.1 Absolute values at follow-up

Caponnetto 2013a\*
a

Cobb 2021
b

Nicotine EC
Mean
3.2
2.67
SD
0.9
0.81
Total
47
130

Non-nicotine EC
Mean
3.5
2.52
SD
0.9
0.71
Total
41
130

Std. Mean Difference
IV, Random, 95% CI
-0.33 [-0.75 , 0.09]
0.20 [-0.05 , 0.44]

Std. Mean Difference
IV, Random, 95% CI


-1

-0.5

0

0.5

1


Favours non-nicotine EC

Favours nicotine EC

Footnotes

a
Data are 2.4% nicotine compared to no-nicotine; 1.8% nicotine arm reported elsewhere

b
Comparing highest (36mg) dose EC arm to no-nicotine EC arm; N at follow-up not provided, so N at baseline used


Analysis 7.10: FEV1/FVC


Study or Subgroup

7.10.1 Absolute values at follow-up

Caponnetto 2013a\*
a

Cobb 2021
b

Subtotal (Wald
c
)
Test for overall effect: Z = 0.17 (P = 0.86)

Heterogeneity: Tau² (REML
d
) = 0.00; Chi² = 0.48, df = 1 (P = 0.49); I² = 0%

Total (Wald
c
)
Test for overall effect: Z = 0.17 (P = 0.86)
Test for subgroup differences: Not applicable

Heterogeneity: Tau² (REML
d
) = 0.00; Chi² = 0.48, df = 1 (P = 0.49); I² = 0%

Nicotine EC
Mean
80.3
78.65
SD
7.2
8.86
Total
47
130

177

177

Non-nicotine EC
Mean
81.2
78.36
SD
5.6
7.73
Total
41
130

171

171
Weight
36.3%
63.7%

100.0%

100.0%

Mean Difference
IV, Random, 95% CI
-0.90 [-3.58 , 1.78]
0.29 [-1.73 , 2.31]

-0.14 [-1.76 , 1.47]

-0.14 [-1.76 , 1.47]

Mean Difference
IV, Random, 95% CI


-4

-2

0

2

4


Favours non-nicotine EC

Favours nicotine EC


Footnotes

a
Data are 2.4% nicotine compared to no-nicotine; 1.8% nicotine arm reported elsewhere

b
Comparing highest (36mg) dose EC arm to no-nicotine EC arm; N at follow-up not provided, so N at baseline used

c
CI calculated by Wald-type method.

d
Tau² calculated by Restricted Maximum-Likelihood method.


Analysis 7.11: FVC (l)


Study or Subgroup

7.11.1 Absolute values at follow-up

Cobb 2021
a

Subtotal
Test for overall effect: Z = 1.58 (P = 0.11)

Heterogeneity: Not applicable

Total
Test for overall effect: Z = 1.58 (P = 0.11)
Test for subgroup differences: Not applicable

Heterogeneity: Not applicable

Nicotine EC
Mean
3.38
SD
0.9
Total
130

130

130

Non-nicotine EC
Mean
3.21
SD
0.82
Total
130

130

130
Weight
100.0%

100.0%

100.0%

Std. Mean Difference
IV, Random, 95% CI
0.20 [-0.05 , 0.44]

0.20 [-0.05 , 0.44]

0.20 [-0.05 , 0.44]

Std. Mean Difference
IV, Random, 95% CI


-1

-0.5

0

0.5

1


Favours non-nicotine EC

Favours nicotine EC


Footnotes

a
Comparing highest (36mg) dose EC arm to no-nicotine EC arm; N at follow-up not provided, so N at baseline used


Analysis 7.12: PEF (l/s)


Study or Subgroup

7.12.1 Absolute values at follow-up

Cobb 2021
a

Subtotal
Test for overall effect: Z = 1.70 (P = 0.09)

Heterogeneity: Not applicable

Total
Test for overall effect: Z = 1.70 (P = 0.09)
Test for subgroup differences: Not applicable

Heterogeneity: Not applicable

Nicotine EC
Mean
6.26
SD
1.66
Total
130

130

130

Non-nicotine EC
Mean
5.91
SD
1.65
Total
130

130

130
Weight
100.0%

100.0%

100.0%

Std. Mean Difference
IV, Random, 95% CI
0.21 [-0.03 , 0.45]

0.21 [-0.03 , 0.45]

0.21 [-0.03 , 0.45]

Std. Mean Difference
IV, Random, 95% CI


-1

-0.5

0

0.5

1


Favours non-nicotine EC

Favours nicotine EC


Footnotes

a
Comparing highest (36mg) dose EC arm to no-nicotine EC arm; N at follow-up not provided, so N at baseline used


Analysis 7.13: FEF 25-75 (l/s)


Study or Subgroup

7.13.1 Absolute values at follow-up

Cobb 2021
a

Subtotal
Test for overall effect: Z = 1.40 (P = 0.16)

Heterogeneity: Not applicable

Total
Test for overall effect: Z = 1.40 (P = 0.16)
Test for subgroup differences: Not applicable

Heterogeneity: Not applicable

Nicotine EC
Mean
2.6
SD
1.24
Total
130

130

130

Non-nicotine EC
Mean
2.4
SD
1.04
Total
130

130

130
Weight
100.0%

100.0%

100.0%

Std. Mean Difference
IV, Random, 95% CI
0.17 [-0.07 , 0.42]

0.17 [-0.07 , 0.42]

0.17 [-0.07 , 0.42]

Std. Mean Difference
IV, Random, 95% CI


-1

-0.5

0

0.5

1


Favours non-nicotine EC

Favours nicotine EC


Footnotes

a
Comparing highest (36mg) dose EC arm to no-nicotine EC arm; N at follow-up not provided, so N at baseline used


Analysis 7.14: Product use at 6+ months


Study or Subgroup
Bullen 2013

Cobb 2021
a

Cobb 2021
b
Eisenberg 2020

Total (HKSJ
c
)
Total events:
Test for overall effect: T = 1.08, df = 3 (P = 0.36)

Heterogeneity: Tau² (DL
d
) = 0.02; Chi² = 4.30, df = 3 (P = 0.23); I² = 30%

Nicotine EC
Events
71
49
62
37
219
Total
241
130
130
100

601

Non-nicotine EC
Events
20
23
24
21
88
Total
57
65
65
86

273
Weight
24.9%
25.5%
28.5%
21.2%

100.0%

Risk Ratio
M-H, Random, 95% CI
0.84 [0.56 , 1.26]
1.07 [0.72 , 1.58]
1.29 [0.90 , 1.86]
1.52 [0.96 , 2.38]

1.14 [0.77 , 1.69]

Risk Ratio
M-H, Random, 95% CI


0.05

0.2

1

5

20


Higher in non-nicotine EC

Higher in nicotine EC


Footnotes

a
8 mg/mL; control group split to avoid double-counting. Data provided as ITT with n randomized as denominator; those not followed up assumed to be not using study product

b
36 mg/mL; control group split to avoid double-counting. Data provided as ITT with n randomized as denominator; those not followed up assumed to be not using study product

c
CI calculated by Hartung-Knapp-Sidik-Jonkman (HKSJ) method.

d
Tau² calculated by DerSimonian and Laird method.


Analysis 8.1: Smoking cessation


Study or Subgroup

Auer 2024
a
Begh 2021
Carpenter 2023
Dawkins 2020
Eisenberg 2020
Halpern 2018

Holliday 2019
b
Lucchiari 2022
Pope 2024
Pratt 2022
Xu 2023\*

Total (HKSJ
c
)
Total events:
Test for overall effect: T = 5.58, df = 10 (P = 0.0002)

Heterogeneity: Tau² (DL
d
) = 0.02; Chi² = 11.47, df = 10 (P = 0.32); I² = 13%

Nicotine EC
Events
141
7
58
3
5
4
6
15
35
6
91
371
Total
622
164
427
48
128
1199
40
70
484
120
566

3868

Usual care
Events
102
3
17
0
1
0
2
10
20
2
14
171
Total
624
161
211
32
121
813
40
70
488
120
271

2951
Weight
40.6%
2.7%
14.8%
0.6%
1.1%
0.6%
2.0%
8.2%
13.9%
1.9%
13.6%

100.0%

Risk Ratio
M-H, Random, 95% CI
1.39 [1.10 , 1.74]
2.29 [0.60 , 8.70]
1.69 [1.01 , 2.82]
4.71 [0.25 , 88.30]
4.73 [0.56 , 39.88]
6.11 [0.33 , 113.24]
3.00 [0.64 , 13.98]
1.50 [0.72 , 3.11]
1.76 [1.03 , 3.01]
3.00 [0.62 , 14.57]
3.11 [1.81 , 5.36]

1.78 [1.42 , 2.25]

Risk Ratio
M-H, Random, 95% CI


0.01

0.1

1

10

100


Favours usual care

Favours nicotine EC


Risk of Bias
A

+

+

+

−

+

?

+

+

+

+

+
B

+

+

+

?

+

?

+

+

+

+

+
C

−

−

−

−

+

−

−

+

−

−

−
D

+

+

−

+

+

+

+

+

+

+

−
E

+

+

+

−

+

−

+

+

+

+

+
F

+

+

+

+

+

+

+

−

+

?

?
G


Footnotes

a
As NRT was not provided by the study, we classed this comparator arm as "behavioural support only."

b
Although participants were given a choice of nicotine concentration including 0 mg, none of the participants chose the non-nicotine e-liquid

c
CI calculated by Hartung-Knapp-Sidik-Jonkman (HKSJ) method.

d
Tau² calculated by DerSimonian and Laird method.
Risk of bias legend

(A) Random sequence generation (selection bias)

(B) Allocation concealment (selection bias)

(C) Blinding of participants and personnel (performance bias)

(D) Blinding of outcome assessment (detection bias)

(E) Incomplete outcome data (attrition bias)

(F) Selective reporting (reporting bias)

(G) Other bias


Analysis 8.2: Adverse events


Study or Subgroup
Auer 2024

Carpenter 2017
a
Carpenter 2023
Eisenberg 2020

Holliday 2019
b
Kale 2025
Piper 2025
Walele 2018\*

Total (HKSJ
c
)
Total events:
Test for overall effect: T = 1.95, df = 7 (P = 0.09)

Heterogeneity: Tau² (DL
d
) = 0.02; Chi² = 20.64, df = 7 (P = 0.004); I² = 66%

Nicotine EC
Events
272
20
180
120
5
12
34
271
914
Total
575
34
292
128
29
15
54
306

1433

Usual care
Events
229
8
86
88
0
10
8
80
509
Total
556
16
163
121
29
12
53
102

1052
Weight
20.7%
4.6%
18.2%
21.6%
0.2%
9.1%
3.5%
22.1%

100.0%

Risk Ratio
M-H, Random, 95% CI
1.15 [1.01 , 1.31]
1.18 [0.67 , 2.07]
1.17 [0.98 , 1.39]
1.29 [1.15 , 1.45]
11.00 [0.64 , 190.26]
0.96 [0.67 , 1.37]
4.17 [2.13 , 8.16]
1.13 [1.01 , 1.26]

1.22 [0.96 , 1.55]

Risk Ratio
M-H, Random, 95% CI


0.01

0.1

1

10

100


Favours nicotine EC

Favours usual care


Risk of Bias
A

+

?

+

+

+

+

+

+
B

+

?

+

+

+

+

?

+
C

−

−

−

+

−

−

−

−
D

+

−

−

+

+

+

−

−
E

+

+

+

+

+

+

+

+
F

+

?

+

+

+

+

+

+
G


+


Footnotes

a
24 mg EC arm included here; 16 mg data reported elsewhere

b
Participants offered choice of nicotine or no-nicotine EC; all chose nicotine-containing EC

c
CI calculated by Hartung-Knapp-Sidik-Jonkman (HKSJ) method.

d
Tau² calculated by DerSimonian and Laird method.
Risk of bias legend

(A) Random sequence generation (selection bias)

(B) Allocation concealment (selection bias)

(C) Blinding of participants and personnel (performance bias)

(D) Blinding of outcome assessment (detection bias)

(E) Incomplete outcome data (attrition bias)

(F) Selective reporting (reporting bias)

(G) Other bias


Analysis 8.3: Serious adverse events


Study or Subgroup
Auer 2024
Avila 2024
Begh 2021

Carpenter 2017
a
Carpenter 2023

Edmiston 2022\*
b
Eisenberg 2020
George 2019

Holliday 2019
c
Kale 2025
Piper 2025
Pope 2024
Pratt 2022
Pulvers 2020
Walele 2018\*

Total (Wald
d
)
Total events:
Test for overall effect: Z = 0.45 (P = 0.65)

Heterogeneity: Tau² (DL
e
) = 0.00; Chi² = 5.55, df = 6 (P = 0.48); I² = 0%

Nicotine EC
Events
25
0
11
0
1
0
3
0
0
0
0
25
2
0
5
72
Total
575
14
148
34
292
300
128
37
29
15
54
484
120
115
306

2651

Usual care
Events
31
0
6
0
0
0
4
0
0
0
0
25
7
0
0
73
Total
566
7
144
16
163
150
121
40
29
12
53
488
120
54
102

2065
Weight
40.4%
11.4%
1.0%
4.9%
36.6%
4.4%
1.3%

100.0%

Risk Ratio
M-H, Random, 95% CI
0.79 [0.47 , 1.33]
Not estimable
1.78 [0.68 , 4.70]
Not estimable
1.68 [0.07 , 40.98]
Not estimable
0.71 [0.16 , 3.10]
Not estimable
Not estimable
Not estimable
Not estimable
1.01 [0.59 , 1.73]
0.29 [0.06 , 1.35]
Not estimable
3.69 [0.21 , 66.17]

0.93 [0.67 , 1.29]

Risk Ratio
M-H, Random, 95% CI


0.01

0.1

1

10

100


Favours nicotine EC

Favours usual care


Risk of Bias
A

+

+

+

?

+

?

+

+

+

+

+

+

+

+

+
B

+

+

+

?

+

?

+

+

+

+

?

+

+

+

+
C

−

+

−

−

−

−

+

?

−

−

−

−

−

−

−
D

+

+

+

−

−

?

+

−

+

+

−

+

+

+

−
E

+

+

+

+

+

+

+

?

+

+

+

+

+

+

+
F

+

−

+

?

+

+

+

+

+

+

+

+

?

+

+
G


+


Footnotes

a
Data from 24 mg arm (0 events in 16 mg arm as well)

b
Menthol and tobacco flavour arms were combined

c
Participants offered choice of nicotine or no-nicotine EC; all chose nicotine-containing EC

d
CI calculated by Wald-type method.

e
Tau² calculated by DerSimonian and Laird method.
Risk of bias legend

(A) Random sequence generation (selection bias)

(B) Allocation concealment (selection bias)

(C) Blinding of participants and personnel (performance bias)

(D) Blinding of outcome assessment (detection bias)

(E) Incomplete outcome data (attrition bias)

(F) Selective reporting (reporting bias)

(G) Other bias


Analysis 8.4: Carbon monoxide (ppm)


Study or Subgroup

8.4.1 Change from baseline
Avila 2024
Holliday 2019
Ikonomidis 2024

Piper 2025
a
Pulvers 2020

8.4.2 Absolute values at follow-up
Adriaens 2014
Begh 2021
Carpenter 2017
Dawkins 2020
Hatsukami 2020
Ikonomidis 2020a
Ikonomidis 2020b
Ozga-Hess 2019
Pratt 2022

Nicotine EC
Mean
-15.36
-12
-7.9
-8.91
-8.13
6.4
25.3
22.4
16.8
11.02
5.6
5.9
18.3
21.8
SD
22.7
11
5.1
10.1
2.75
1.6
13.8
15.2
12.1
8.96
3.8
0.7
15.9
8.7
Total
14
29
25
54
114
31
148
42
39
58
20
20
18
108

Usual care
Mean
8.72
-5.8
1.2
-6.81
-0.37
14.7
23.8
32.9
18.1
16.96
10.2
13.6
19.7
21.9
SD
26.3
12.3
4.8
9.57
3.59
1.6
10.8
16.9
9.5
9.94
3.8
1.3
13.5
8.4
Total
7
29
50
53
54
15
144
19
21
32
20
20
16
105

Mean Difference
IV, Random, 95% CI
-24.08 [-46.90 , -1.26]
-6.20 [-12.21 , -0.19]
-9.10 [-11.50 , -6.70]
-2.10 [-5.83 , 1.63]
-7.76 [-8.84 , -6.68]
-8.30 [-9.29 , -7.31]
1.50 [-1.34 , 4.34]
-10.50 [-19.38 , -1.62]
-1.30 [-6.86 , 4.26]
-5.94 [-10.08 , -1.80]
-4.60 [-6.96 , -2.24]
-7.70 [-8.35 , -7.05]
-1.40 [-11.28 , 8.48]
-0.10 [-2.40 , 2.20]

Mean Difference
IV, Random, 95% CI


-20

-10

0

10

20


Favours EC

Favours usual care

Footnotes

a
Data provided by author. Values collapse NRT active and placebo conditions (authors report no interaction found). Values are at the end of 'Switch Week 1


Analysis 8.5: Heart rate (bpm)


Study or Subgroup

8.5.1 Absolute values at follow-up
Hatsukami 2020

Subtotal
Test for overall effect: Z = 0.42 (P = 0.67)

Heterogeneity: Not applicable

Total
Test for overall effect: Z = 0.42 (P = 0.67)
Test for subgroup differences: Not applicable

Heterogeneity: Not applicable

Nicotine EC
Mean
74.81
SD
13.91
Total
58

58

58

Usual care
Mean
73.64
SD
11.81
Total
32

32

32
Weight
100.0%

100.0%

100.0%

Mean Difference
IV, Random, 95% CI
1.17 [-4.27 , 6.61]

1.17 [-4.27 , 6.61]

1.17 [-4.27 , 6.61]

Mean Difference
IV, Random, 95% CI


-20

-10

0

10

20


Favours EC

Favours usual care


Analysis 8.6: Systolic blood pressure


Study or Subgroup

8.6.1 Change from baseline
Pulvers 2020

Subtotal
Test for overall effect: Z = 3.09 (P = 0.002)

Heterogeneity: Not applicable

8.6.2 Absolute values at follow-up
Hatsukami 2020
Ikonomidis 2020a

Subtotal (HKSJ
a
)
Test for overall effect: T = 0.52, df = 1 (P = 0.69)

Heterogeneity: Tau² (REML
b
) = 0.00; Chi² = 0.68, df = 1 (P = 0.41); I² = 0%

Total (HKSJ
a
)
Test for overall effect: T = 1.11, df = 2 (P = 0.38)
Test for subgroup differences: Chi² = 2.71, df = 1 (P = 0.10), I² = 63.2%

Heterogeneity: Tau² (REML
b
) = 2.16; Chi² = 2.62, df = 2 (P = 0.27); I² = 23%

Nicotine EC
Mean
1.07
123.1
128.7
SD
5.68
13.3
19.9
Total
114

114
58
20

78

192

Usual care
Mean
3.75
123.1
123.5
SD
5.03
13.2
15.1
Total
54

54
32
20

52

106
Weight
73.5%

73.5%
20.1%
6.4%

26.5%

100.0%

Mean Difference
IV, Random, 95% CI
-2.68 [-4.38 , -0.98]

-2.68 [-4.38 , -0.98]
0.00 [-5.71 , 5.71]
5.20 [-5.75 , 16.15]

1.11 [-25.99 , 28.21]

-1.64 [-7.97 , 4.70]

Mean Difference
IV, Random, 95% CI


-20

-10

0

10

20


Favours EC

Favours usual care


Footnotes

a
CI calculated by Hartung-Knapp-Sidik-Jonkman (HKSJ) method.

b
Tau² calculated by Restricted Maximum-Likelihood method.


Analysis 8.7: Blood oxygen saturation


Study or Subgroup

8.7.1 Absolute values at follow-up
Hatsukami 2020

Subtotal
Test for overall effect: Z = 0.79 (P = 0.43)

Heterogeneity: Not applicable

Total
Test for overall effect: Z = 0.79 (P = 0.43)
Test for subgroup differences: Not applicable

Heterogeneity: Not applicable

Nicotine EC
Mean
98.1
SD
1.5
Total
57

57

57

Usual care
Mean
97.9
SD
0.9
Total
32

32

32
Weight
100.0%

100.0%

100.0%

Mean Difference
IV, Random, 95% CI
0.20 [-0.30 , 0.70]

0.20 [-0.30 , 0.70]

0.20 [-0.30 , 0.70]

Mean Difference
IV, Random, 95% CI


-0.5

-0.25

0

0.25

0.5


Favours usual care

Favours EC


Analysis 8.8: 3-HPMA (SMD)


Study or Subgroup

8.8.1 Absolute values at follow-up

Hatsukami 2020
a

Subtotal
Test for overall effect: Z = 1.38 (P = 0.17)

Heterogeneity: Not applicable

8.8.2 Change from baseline

Walele 2018\*
b

Subtotal
Test for overall effect: Z = 4.28 (P < 0.0001)

Heterogeneity: Not applicable

Total (Wald
c
)
Test for overall effect: Z = 4.42 (P < 0.00001)
Test for subgroup differences: Chi² = 0.63, df = 1 (P = 0.43), I² = 0%

Heterogeneity: Tau² (REML
d
) = 0.00; Chi² = 0.63, df = 1 (P = 0.43); I² = 0%

Nicotine EC
Mean
4612
-530
SD
4263
1272.5
Total
58

58
284

284

342

Usual care
Mean
5926
96
SD
4298
1142.9
Total
32

32
100

100

132
Weight
22.0%

22.0%
78.0%

78.0%

100.0%

Std. Mean Difference
IV, Random, 95% CI
-0.30 [-0.74 , 0.13]

-0.30 [-0.74 , 0.13]
-0.50 [-0.73 , -0.27]

-0.50 [-0.73 , -0.27]

-0.46 [-0.66 , -0.26]

Std. Mean Difference
IV, Random, 95% CI


-2

-1

0

1

2


Favours EC

Favours usual care


Footnotes

a
Measured as pmol/mg creatinine

b
Measured as micrograms

c
CI calculated by Wald-type method.

d
Tau² calculated by Restricted Maximum-Likelihood method.


Analysis 8.9: NNAL (SMD)


Study or Subgroup

8.9.1 Absolute values at follow-up

Carpenter 2017
a

Hatsukami 2020
b

8.9.2 Change from baseline

Edmiston 2022\*
c

Pulvers 2020
d

Walele 2018\*
e

Nicotine EC
Mean
151.8
1.2
-172.1
-65.91
-76
SD
158.1
1.7
158
39.41
189.2
Total
41
57
232
114
284

Usual care
Mean
156.9
1.2
-6.3
14.23
6
SD
125.8
1
205.8
39.62
163.3
Total
19
31
128
54
100

Std. Mean Difference
IV, Random, 95% CI
-0.03 [-0.58 , 0.51]
0.00 [-0.44 , 0.44]
-0.94 [-1.16 , -0.71]
-2.02 [-2.41 , -1.63]
-0.45 [-0.68 , -0.22]

Std. Mean Difference
IV, Random, 95% CI


-2

-1

0

1

2


Favours EC

Favours usual care

Footnotes

a
Measured as pg/mL

b
Measured as pmol/mg creatinine

c
ng/g

d
pg/mL

e
Measured as nanograms


Analysis 8.10: 2-HPMA (pmol/mg creatinine)


Study or Subgroup

8.10.1 Absolute values at follow-up
Hatsukami 2020

Subtotal
Test for overall effect: Z = 0.79 (P = 0.43)

Heterogeneity: Not applicable

Total
Test for overall effect: Z = 0.79 (P = 0.43)
Test for subgroup differences: Not applicable

Heterogeneity: Not applicable

Nicotine EC
Mean
733.2
SD
855.6
Total
58

58

58

Usual care
Mean
1013.1
SD
1887.6
Total
32

32

32
Weight
100.0%

100.0%

100.0%

Mean Difference
IV, Random, 95% CI
-279.90 [-969.98 , 410.18]

-279.90 [-969.98 , 410.18]

-279.90 [-969.98 , 410.18]

Mean Difference
IV, Random, 95% CI


-1000

-500

0

500

1000


Favours EC

Favours usual care


Analysis 8.11: HMPMA (pmol/mg creatinine)


Study or Subgroup

8.11.1 Absolute values at follow-up
Hatsukami 2020

Subtotal
Test for overall effect: Z = 1.50 (P = 0.13)

Heterogeneity: Not applicable

Total
Test for overall effect: Z = 1.50 (P = 0.13)
Test for subgroup differences: Not applicable

Heterogeneity: Not applicable

Nicotine EC
Mean
3959
SD
3633
Total
58

58

58

Usual care
Mean
5631
SD
5701
Total
32

32

32
Weight
100.0%

100.0%

100.0%

Mean Difference
IV, Random, 95% CI
-1672.00 [-3857.37 , 513.37]

-1672.00 [-3857.37 , 513.37]

-1672.00 [-3857.37 , 513.37]

Mean Difference
IV, Random, 95% CI


-1000

-500

0

500

1000


Favours EC

Favours usual care


Analysis 8.12: PheT (pmol/mg creatinine)


Study or Subgroup

8.12.1 Absolute values at follow-up
Hatsukami 2020

Subtotal
Test for overall effect: Z = 0.89 (P = 0.37)

Heterogeneity: Not applicable

Total
Test for overall effect: Z = 0.89 (P = 0.37)
Test for subgroup differences: Not applicable

Heterogeneity: Not applicable

Nicotine EC
Mean
2.9
SD
2.8
Total
56

56

56

Usual care
Mean
4.2
SD
8
Total
32

32

32
Weight
100.0%

100.0%

100.0%

Mean Difference
IV, Random, 95% CI
-1.30 [-4.17 , 1.57]

-1.30 [-4.17 , 1.57]

-1.30 [-4.17 , 1.57]

Mean Difference
IV, Random, 95% CI


-20

-10

0

10

20


Favours EC

Favours usual care


Analysis 8.13: CEMA (pmol/mg creatinine)


Study or Subgroup

8.13.1 Absolute values at follow-up
Hatsukami 2020

Subtotal
Test for overall effect: Z = 0.03 (P = 0.97)

Heterogeneity: Not applicable

Total
Test for overall effect: Z = 0.03 (P = 0.97)
Test for subgroup differences: Not applicable

Heterogeneity: Not applicable

Nicotine EC
Mean
512
SD
443
Total
58

58

58

Usual care
Mean
509
SD
358
Total
32

32

32
Weight
100.0%

100.0%

100.0%

Mean Difference
IV, Random, 95% CI
3.00 [-165.47 , 171.47]

3.00 [-165.47 , 171.47]

3.00 [-165.47 , 171.47]

Mean Difference
IV, Random, 95% CI


-500

-250

0

250

500


Favours EC

Favours usual care


Analysis 8.14: AAMA (pmol/mg creatinine)


Study or Subgroup

8.14.1 Absolute values at follow-up
Hatsukami 2020

Subtotal
Test for overall effect: Z = 0.88 (P = 0.38)

Heterogeneity: Not applicable

Total
Test for overall effect: Z = 0.88 (P = 0.38)
Test for subgroup differences: Not applicable

Heterogeneity: Not applicable

Nicotine EC
Mean
495.2
SD
390.9
Total
58

58

58

Usual care
Mean
563.1
SD
328.2
Total
32

32

32
Weight
100.0%

100.0%

100.0%

Mean Difference
IV, Random, 95% CI
-67.90 [-219.73 , 83.93]

-67.90 [-219.73 , 83.93]

-67.90 [-219.73 , 83.93]

Mean Difference
IV, Random, 95% CI


-500

-250

0

250

500


Favours EC

Favours usual care


Analysis 8.15: S-PMA (nanograms)


Study or Subgroup

8.15.1 12 weeks
Walele 2018\*

Subtotal
Test for overall effect: Z = 4.30 (P < 0.0001)

Heterogeneity: Not applicable

Total
Test for overall effect: Z = 4.30 (P < 0.0001)
Test for subgroup differences: Not applicable

Heterogeneity: Not applicable

Nicotine EC
Mean
-1340
SD
3426.3
Total
284

284

284

Usual care
Mean
31
SD
2451.5
Total
100

100

100
Weight
100.0%

100.0%

100.0%

Mean Difference
IV, Random, 95% CI
-1371.00 [-1995.23 , -746.77]

-1371.00 [-1995.23 , -746.77]

-1371.00 [-1995.23 , -746.77]

Mean Difference
IV, Random, 95% CI


-1000

-500

0

500

1000


Favours EC

Favours usual care


Analysis 8.16: FEV1 (SMD)


Study or Subgroup

8.16.1 Change from baseline
Edmiston 2022\*
Walele 2018\*

Subtotal (Wald
a
)
Test for overall effect: Z = 1.00 (P = 0.32)

Heterogeneity: Tau² (REML
b
) = 0.03; Chi² = 3.29, df = 1 (P = 0.07); I² = 70%

Total (Wald
a
)
Test for overall effect: Z = 1.00 (P = 0.32)
Test for subgroup differences: Not applicable

Heterogeneity: Tau² (REML
b
) = 0.03; Chi² = 3.29, df = 1 (P = 0.07); I² = 70%

Nicotine EC
Mean
-0.7
-0.1
SD
6.2
0.9
Total
212
286

498

498

Usual care
Mean
-2.5
-0.1
SD
5.7
0.8
Total
115
101

216

216
Weight
49.9%
50.1%

100.0%

100.0%

Std. Mean Difference
IV, Random, 95% CI
0.30 [0.07 , 0.53]
0.00 [-0.23 , 0.23]

0.15 [-0.14 , 0.44]

0.15 [-0.14 , 0.44]

Std. Mean Difference
IV, Random, 95% CI


-0.5

-0.25

0

0.25

0.5


Favours usual care

Favours nicotine EC


Footnotes

a
CI calculated by Wald-type method.

b
Tau² calculated by Restricted Maximum-Likelihood method.


Analysis 8.17: FEF 25-75 (litres/second))


Study or Subgroup

8.17.1 Change from baseline
Pulvers 2020
Walele 2018\*

Subtotal (Wald
a
)
Test for overall effect: Z = 0.26 (P = 0.80)

Heterogeneity: Tau² (REML
b
) = 0.02; Chi² = 3.68, df = 1 (P = 0.06); I² = 73%

Total (Wald
a
)
Test for overall effect: Z = 0.26 (P = 0.80)
Test for subgroup differences: Not applicable

Heterogeneity: Tau² (REML
b
) = 0.02; Chi² = 3.68, df = 1 (P = 0.06); I² = 73%

Nicotine EC
Mean
-0.11
-0.1
SD
0.43
0.4
Total
114
286

400

400

Usual care
Mean
0.03
-0.2
SD
0.44
1
Total
54
101

155

155
Weight
54.6%
45.4%

100.0%

100.0%

Mean Difference
IV, Random, 95% CI
-0.14 [-0.28 , 0.00]
0.10 [-0.10 , 0.30]

-0.03 [-0.27 , 0.20]

-0.03 [-0.27 , 0.20]

Mean Difference
IV, Random, 95% CI


-4

-2

0

2

4


Favours usual care

Favours nicotine EC


Footnotes

a
CI calculated by Wald-type method.

b
Tau² calculated by Restricted Maximum-Likelihood method.


Analysis 8.18: PEF 25-75 (litres/minute)


Study or Subgroup

8.18.1 Change from baseline
Walele 2018\*

Subtotal
Test for overall effect: Z = 0.63 (P = 0.53)

Heterogeneity: Not applicable

Total
Test for overall effect: Z = 0.63 (P = 0.53)
Test for subgroup differences: Not applicable

Heterogeneity: Not applicable

Nicotine EC
Mean
11.7
SD
75.9
Total
286

286

286

Usual care
Mean
18.8
SD
103.6
Total
101

101

101
Weight
100.0%

100.0%

100.0%

Mean Difference
IV, Random, 95% CI
-7.10 [-29.14 , 14.94]

-7.10 [-29.14 , 14.94]

-7.10 [-29.14 , 14.94]

Mean Difference
IV, Random, 95% CI


-100

-50

0

50

100


Favours usual care

Favours nicotine EC


Analysis 8.19: FEV1/FVC


Study or Subgroup

8.19.1 Change from baseline
Edmiston 2022\*

Subtotal
Test for overall effect: Z = 3.44 (P = 0.0006)

Heterogeneity: Not applicable

Total
Test for overall effect: Z = 3.44 (P = 0.0006)
Test for subgroup differences: Not applicable

Heterogeneity: Not applicable

Nicotine EC
Mean
0.36
SD
4.5
Total
212

212

212

Usual care
Mean
-1.36
SD
4.21
Total
115

115

115
Weight
100.0%

100.0%

100.0%

Mean Difference
IV, Random, 95% CI
1.72 [0.74 , 2.70]

1.72 [0.74 , 2.70]

1.72 [0.74 , 2.70]

Mean Difference
IV, Random, 95% CI


-4

-2

0

2

4


Favours control

Favours nicotine EC


Analysis 9.1: Smoking cessation


Study or Subgroup

Cobb 2021
a

Total
Total events:
Test for overall effect: Z = 1.58 (P = 0.11)

Heterogeneity: Not applicable

Higher nicotine
Events
10
10
Total
130

130

Lower nicotine
Events
4
4
Total
130

130
Weight
100.0%

100.0%

Risk Ratio
M-H, Random, 95% CI
2.50 [0.80 , 7.77]

2.50 [0.80 , 7.77]

Risk Ratio
M-H, Random, 95% CI


0.01

0.1

1

10

100


Favours lower nicotine

Favours higher nicotine


Footnotes

a
36 v 8 mg/mL


Analysis 9.2: Adverse events


Study or Subgroup
Kanobe 2022\*

Total
Total events:
Test for overall effect: Z = 0.46 (P = 0.64)

Heterogeneity: Not applicable

Higher
Events
17
17
Total
33

33

Lower
Events
20
20
Total
35

35
Weight
100.0%

100.0%

Risk Ratio
M-H, Random, 95% CI
0.90 [0.58 , 1.40]

0.90 [0.58 , 1.40]

Risk Ratio
M-H, Random, 95% CI


0.01

0.1

1

10

100


Favours higher

Favours lower


Analysis 9.3: Serious adverse events


Study or Subgroup
Caponnetto 2013a\*

Cobb 2021
a

Total
Total events:
Test for overall effect: Z = 0.75 (P = 0.45)

Heterogeneity: Not applicable

Higher nicotine content
Events
0
8
8
Total
35
86

121

Lower nicotine content
Events
0
5
5
Total
37
81

118
Weight
100.0%

100.0%

Risk Ratio
M-H, Random, 95% CI
Not estimable
1.51 [0.51 , 4.42]

1.51 [0.51 , 4.42]

Risk Ratio
M-H, Random, 95% CI


0.01

0.1

1

10

100


Favours higher

Favours lower


Footnotes

a
36 v 8 mg/mL


Analysis 9.4: Carbon monoxide (ppm)


Study or Subgroup

9.4.1 Change from baseline
Caponnetto 2013a\*
Cobb 2021

Subtotal (Wald
a
)
Test for overall effect: Z = 2.21 (P = 0.03)

Heterogeneity: Tau² (REML
b
) = 0.00; Chi² = 0.95, df = 1 (P = 0.33); I² = 0%

9.4.2 Absolute values at follow-up
Kimber 2021

Subtotal
Test for overall effect: Z = 0.65 (P = 0.51)

Heterogeneity: Not applicable

Total (Wald
a
)
Test for overall effect: Z = 2.28 (P = 0.02)
Test for subgroup differences: Chi² = 0.09, df = 1 (P = 0.77), I² = 0%

Heterogeneity: Tau² (REML
b
) = 0.00; Chi² = 1.04, df = 2 (P = 0.59); I² = 0%

higher dose
Mean
-6
-5.53
10.55
SD
6.4
2.7
7.97
Total
76
80

156
20

20

176

lower dose
Mean
-5.8
-4.4
12.21
SD
3.4
3.1
7.94
Total
79
74

153
19

19

172
Weight
23.8%
73.7%

97.5%
2.5%

2.5%

100.0%

Mean Difference
IV, Random, 95% CI
-0.20 [-1.82 , 1.42]
-1.13 [-2.05 , -0.21]

-0.90 [-1.70 , -0.10]
-1.66 [-6.65 , 3.33]

-1.66 [-6.65 , 3.33]

-0.92 [-1.71 , -0.13]

Mean Difference
IV, Random, 95% CI


-2

-1

0

1

2


Favours higher dose

Favours lower dose


Footnotes

a
CI calculated by Wald-type method.

b
Tau² calculated by Restricted Maximum-Likelihood method.


Analysis 9.5: Heart rate


Study or Subgroup

9.5.1 Change from baseline
Caponnetto 2013a\*

Subtotal
Test for overall effect: Z = 0.87 (P = 0.38)

Heterogeneity: Not applicable

9.5.2 Absolute values at follow-up

Cobb 2021
a

Subtotal
Test for overall effect: Z = 1.34 (P = 0.18)

Heterogeneity: Not applicable

Total (Wald
b
)
Test for overall effect: Z = 0.31 (P = 0.76)
Test for subgroup differences: Chi² = 2.46, df = 1 (P = 0.12), I² = 59.4%

Heterogeneity: Tau² (REML
c
) = 1.74; Chi² = 2.46, df = 1 (P = 0.12); I² = 59%

higher dose
Mean
-1.7
79.33
SD
3.4
11.65
Total
73

73
130

130

203

lower dose
Mean
-1.2
77.41
SD
3.6
11.43
Total
75

75
130

130

205
Weight
64.7%

64.7%
35.3%

35.3%

100.0%

Mean Difference
IV, Random, 95% CI
-0.50 [-1.63 , 0.63]

-0.50 [-1.63 , 0.63]
1.92 [-0.89 , 4.73]

1.92 [-0.89 , 4.73]

0.36 [-1.91 , 2.62]

Mean Difference
IV, Random, 95% CI


-20

-10

0

10

20


Favours higher dose

Favours lower dose


Footnotes

a
N at follow-up not provided, so N at baseline used

b
CI calculated by Wald-type method.

c
Tau² calculated by Restricted Maximum-Likelihood method.


Analysis 9.6: Systolic blood pressure


Study or Subgroup

9.6.1 Change from baseline
Caponnetto 2013a\*

Subtotal
Test for overall effect: Z = 0.88 (P = 0.38)

Heterogeneity: Not applicable

9.6.2 Absolute values at follow-up

Cobb 2021
a

Subtotal
Test for overall effect: Z = 0.57 (P = 0.57)

Heterogeneity: Not applicable

Total (Wald
b
)
Test for overall effect: Z = 1.03 (P = 0.30)
Test for subgroup differences: Chi² = 0.02, df = 1 (P = 0.88), I² = 0%

Heterogeneity: Tau² (REML
c
) = 0.00; Chi² = 0.02, df = 1 (P = 0.88); I² = 0%

higher dose
Mean
-3.9
125.85
SD
5.7
14.82
Total
73

73
130

130

203

lower dose
Mean
-4.7
124.72
SD
5.4
17.08
Total
75

75
130

130

205
Weight
82.5%

82.5%
17.5%

17.5%

100.0%

Mean Difference
IV, Random, 95% CI
0.80 [-0.99 , 2.59]

0.80 [-0.99 , 2.59]
1.13 [-2.76 , 5.02]

1.13 [-2.76 , 5.02]

0.86 [-0.77 , 2.48]

Mean Difference
IV, Random, 95% CI


-20

-10

0

10

20


Favours higher dose

Favours lower dose


Footnotes

a
N at follow-up not provided, so N at baseline used

b
CI calculated by Wald-type method.

c
Tau² calculated by Restricted Maximum-Likelihood method.


Analysis 9.7: FeNO (ppb)


Study or Subgroup

9.7.1 12 weeks
Caponnetto 2013a\*

Subtotal
Test for overall effect: Z = 0.88 (P = 0.38)

Heterogeneity: Not applicable

Total
Test for overall effect: Z = 0.88 (P = 0.38)
Test for subgroup differences: Not applicable

Heterogeneity: Not applicable

higher dose
Mean
2.8
SD
1.7
Total
49

49

49

lower dose
Mean
2.5
SD
1.6
Total
44

44

44
Weight
100.0%

100.0%

100.0%

Mean Difference
IV, Random, 95% CI
0.30 [-0.37 , 0.97]

0.30 [-0.37 , 0.97]

0.30 [-0.37 , 0.97]

Mean Difference
IV, Random, 95% CI


-20

-10

0

10

20


Favours higher dose

Favours lower dose


Analysis 9.8: FEV1 (l)


Study or Subgroup

9.8.1 Change from baseline
Caponnetto 2013a\*

Subtotal
Test for overall effect: Z = 0.19 (P = 0.85)

Heterogeneity: Not applicable

9.8.2 Absolute values at follow-up

Cobb 2021
a

Subtotal
Test for overall effect: Z = 1.59 (P = 0.11)

Heterogeneity: Not applicable

Total (Wald
b
)
Test for overall effect: Z = 0.66 (P = 0.51)
Test for subgroup differences: Chi² = 2.18, df = 1 (P = 0.14), I² = 54.0%

Heterogeneity: Tau² (REML
c
) = 0.01; Chi² = 2.18, df = 1 (P = 0.14); I² = 54%

higher dose
Mean
0
2.67
SD
0.3
0.81
Total
47

47
130

130

177

lower dose
Mean
0.01
2.52
SD
0.2
0.71
Total
43

43
130

130

173
Weight
61.9%

61.9%
38.1%

38.1%

100.0%

Mean Difference
IV, Random, 95% CI
-0.01 [-0.11 , 0.09]

-0.01 [-0.11 , 0.09]
0.15 [-0.04 , 0.34]

0.15 [-0.04 , 0.34]

0.05 [-0.10 , 0.20]

Mean Difference
IV, Random, 95% CI


-2

-1

0

1

2


Favours lower dose

Favours higher dose


Footnotes

a
N at follow-up not provided, so N at baseline used

b
CI calculated by Wald-type method.

c
Tau² calculated by Restricted Maximum-Likelihood method.


Analysis 9.9: FVC (l)


Study or Subgroup

9.9.1 Change from baseline
Caponnetto 2013a\*

Subtotal
Test for overall effect: Z = 0.47 (P = 0.64)

Heterogeneity: Not applicable

9.9.2 Absolute values at follow-up

Cobb 2021
a

Subtotal
Test for overall effect: Z = 0.97 (P = 0.33)

Heterogeneity: Not applicable

Total (Wald
b
)
Test for overall effect: Z = 0.87 (P = 0.39)
Test for subgroup differences: Chi² = 0.42, df = 1 (P = 0.52), I² = 0%

Heterogeneity: Tau² (REML
c
) = 0.00; Chi² = 0.42, df = 1 (P = 0.52); I² = 0%

higher dose
Mean
-0.02
3.38
SD
0.3
0.9
Total
47

47
130

130

177

lower dose
Mean
0.01
3.5
SD
0.3
1.08
Total
43

43
130

130

173
Weight
79.1%

79.1%
20.9%

20.9%

100.0%

Mean Difference
IV, Random, 95% CI
-0.03 [-0.15 , 0.09]

-0.03 [-0.15 , 0.09]
-0.12 [-0.36 , 0.12]

-0.12 [-0.36 , 0.12]

-0.05 [-0.16 , 0.06]

Mean Difference
IV, Random, 95% CI


-2

-1

0

1

2


Favours lower dose

Favours higher dose


Footnotes

a
N at follow-up not provided, so N at baseline used

b
CI calculated by Wald-type method.

c
Tau² calculated by Restricted Maximum-Likelihood method.


Analysis 9.10: FEV1/FVC


Study or Subgroup

9.10.1 Change from baseline
Caponnetto 2013a\*

Subtotal
Test for overall effect: Z = 2.33 (P = 0.02)

Heterogeneity: Not applicable

9.10.2 Absolute values at follow-up

Cobb 2021
a

Subtotal
Test for overall effect: Z = 0.71 (P = 0.48)

Heterogeneity: Not applicable

Total (Wald
b
)
Test for overall effect: Z = 2.44 (P = 0.01)
Test for subgroup differences: Chi² = 0.00, df = 1 (P = 0.99), I² = 0%

Heterogeneity: Tau² (REML
c
) = 0.00; Chi² = 0.00, df = 1 (P = 0.99); I² = 0%

higher dose
Mean
0.96
78.65
SD
2
8.86
Total
47

47
130

130

177

lower dose
Mean
0.05
77.76
SD
1.7
11.14
Total
43

43
130

130

173
Weight
91.1%

91.1%
8.9%

8.9%

100.0%

Mean Difference
IV, Random, 95% CI
0.91 [0.15 , 1.67]

0.91 [0.15 , 1.67]
0.89 [-1.56 , 3.34]

0.89 [-1.56 , 3.34]

0.91 [0.18 , 1.64]

Mean Difference
IV, Random, 95% CI


-10

-5

0

5

10


Favours lower dose

Favours higher dose


Footnotes

a
N at follow-up not provided, so N at baseline used

b
CI calculated by Wald-type method.

c
Tau² calculated by Restricted Maximum-Likelihood method.


Analysis 9.11: PEF (l/s)


Study or Subgroup

9.11.1 Absolute values at follow-up

Cobb 2021
a

Subtotal
Test for overall effect: Z = 0.50 (P = 0.62)

Heterogeneity: Not applicable

Total
Test for overall effect: Z = 0.50 (P = 0.62)
Test for subgroup differences: Not applicable

Heterogeneity: Not applicable

Higher dose
Mean
6.26
SD
1.66
Total
130

130

130

Lower dose
Mean
6.15
SD
1.86
Total
130

130

130
Weight
100.0%

100.0%

100.0%

Std. Mean Difference
IV, Random, 95% CI
0.06 [-0.18 , 0.31]

0.06 [-0.18 , 0.31]

0.06 [-0.18 , 0.31]

Std. Mean Difference
IV, Random, 95% CI


-1

-0.5

0

0.5

1


Favours lower dose

Favours higher dose


Footnotes

a
N at follow-up not provided, so N at baseline used


Analysis 9.12: FEF 25-75 (l/s)


Study or Subgroup

9.12.1 Absolute values at follow-up

Cobb 2021
a

Subtotal
Test for overall effect: Z = 0.13 (P = 0.90)

Heterogeneity: Not applicable

Total
Test for overall effect: Z = 0.13 (P = 0.90)
Test for subgroup differences: Not applicable

Heterogeneity: Not applicable

Higher dose
Mean
2.6
SD
1.24
Total
130

130

130

Lower dose
Mean
2.62
SD
1.27
Total
130

130

130
Weight
100.0%

100.0%

100.0%

Std. Mean Difference
IV, Random, 95% CI
-0.02 [-0.26 , 0.23]

-0.02 [-0.26 , 0.23]

-0.02 [-0.26 , 0.23]

Std. Mean Difference
IV, Random, 95% CI


-1

-0.5

0

0.5

1


Favours lower dose

Favours higher dose


Footnotes

a
N at follow-up not provided, so N at baseline used


Analysis 9.13: NNAL (pg/mg creatinine) at 24 weeks


Study or Subgroup
Cobb 2021

Total
Test for overall effect: Z = 0.33 (P = 0.74)

Heterogeneity: Not applicable

Higher nicotine
Mean
-155.35
SD
266.1
Total
79

79

Lower nicotine
Mean
-141.46
SD
259.14
Total
73

73
Weight
100.0%

100.0%

Mean Difference
IV, Random, 95% CI
-13.89 [-97.42 , 69.64]

-13.89 [-97.42 , 69.64]

Mean Difference
IV, Random, 95% CI


-100

-50

0

50

100


Favours higher nicotine

Favours lower nicotine


Analysis 9.14: Product use at 6+ months


Study or Subgroup
Cobb 2021

Total
Total events:
Test for overall effect: Z = 1.62 (P = 0.11)

Heterogeneity: Not applicable

Higher nicotine
Events
62
62
Total
130

130

Lower nicotine
Events
49
49
Total
130

130
Weight
100.0%

100.0%

Risk Ratio
M-H, Random, 95% CI
1.27 [0.95 , 1.68]

1.27 [0.95 , 1.68]

Risk Ratio
M-H, Random, 95% CI


0.01

0.1

1

10

100


Higher use in lower nicotine

Higher use in higher nicotine


Analysis 10.1: Smoking cessation


Study or Subgroup
Xu 2023\*

Total
Total events:
Test for overall effect: Z = 1.18 (P = 0.24)

Heterogeneity: Not applicable

Choice of flavours
Events
40
40
Total
281

281

Tobacco flavour
Events
51
51
Total
285

285
Weight
100.0%

100.0%

Risk Ratio
M-H, Random, 95% CI
0.80 [0.54 , 1.16]

0.80 [0.54 , 1.16]

Risk Ratio
M-H, Random, 95% CI


0.01

0.1

1

10

100


Favours tobacco flavour

Favours choice of flavours


Analysis 10.2: Product use at 6+ months


Study or Subgroup
Xu 2023\*

Total
Total events:
Test for overall effect: Z = 0.74 (P = 0.46)

Heterogeneity: Not applicable

Choice of flavours
Events
91
91
Total
261

261

Tobacco flavour
Events
83
83
Total
261

261
Weight
100.0%

100.0%

Risk Ratio
M-H, Random, 95% CI
1.10 [0.86 , 1.40]

1.10 [0.86 , 1.40]

Risk Ratio
M-H, Random, 95% CI


0.01

0.1

1

10

100


Higher with  tobacco flavour

Higher with choice of flavours


Analysis 10.3: Adverse events (EC as adjunct to VLNC)


Study or Subgroup
Higgins 2024

Total
Total events:
Test for overall effect: Z = 0.10 (P = 0.92)

Heterogeneity: Not applicable

Choice of flavour
Events
72
72
Total
84

84

Tobacco flavour
Events
63
63
Total
74

74
Weight
100.0%

100.0%

Risk Ratio
M-H, Random, 95% CI
1.01 [0.88 , 1.15]

1.01 [0.88 , 1.15]

Risk Ratio
M-H, Random, 95% CI


0.2

0.5

1

2

5


Favours choice of flavour

Favours tobacco flavour


Analysis 10.4: Serious adverse events (EC as adjunct to VLNC)


Study or Subgroup
Higgins 2024

Total
Total events:
Test for overall effect: Z = 0.96 (P = 0.34)

Heterogeneity: Not applicable

Choice of flavour
Events
2
2
Total
84

84

Tobacco flavour
Events
4
4
Total
74

74
Weight
100.0%

100.0%

Risk Ratio
M-H, Random, 95% CI
0.44 [0.08 , 2.34]

0.44 [0.08 , 2.34]

Risk Ratio
M-H, Random, 95% CI


0.02

0.1

1

10

50


Favours choice of flavour

Favours tobacco flavour


Analysis 10.5: Change in carbon monoxide (ppm; as adjunct to VLNC)


Study or Subgroup
Higgins 2024

Total
Test for overall effect: Z = 1.35 (P = 0.18)

Heterogeneity: Not applicable

Choice of flavours
Mean
-6.42
SD
20.67
Total
69

69

Tobacco flavour
Mean
-1.9
SD
16.48
Total
55

55
Weight
100.0%

100.0%

Mean Difference
IV, Random, 95% CI
-4.52 [-11.06 , 2.02]

-4.52 [-11.06 , 2.02]

Mean Difference
IV, Random, 95% CI


-20

-10

0

10

20


Favours choice of flavours

Favours tobacco flavour


Analysis 10.6: Change in NNAL (pmol/mg creatinine; as adjunct to VLNC)


Study or Subgroup
Higgins 2024

Total
Test for overall effect: Z = 0.15 (P = 0.88)

Heterogeneity: Not applicable

Choice of flavours
Mean
-0.5
SD
3.73
Total
53

53

Tobacco flavour
Mean
-0.41
SD
2.1
Total
47

47
Weight
100.0%

100.0%

Mean Difference
IV, Random, 95% CI
-0.09 [-1.26 , 1.08]

-0.09 [-1.26 , 1.08]

Mean Difference
IV, Random, 95% CI


-10

-5

0

5

10


Favours choice of flavours

Favours tobacco flavour


Analysis 11.1: Serious adverse events


Study or Subgroup
Edmiston 2022\*

Total
Total events:
Test for overall effect: Not applicable

Heterogeneity: Not applicable

Tobacco
Events
0
0
Total
150

150

Menthol
Events
0
0
Total
150

150
Weight

Risk Ratio
M-H, Random, 95% CI
Not estimable

Not estimable

Risk Ratio
M-H, Random, 95% CI


0.01

0.1

1

10

100


Favours tobacco

Favours menthol


Analysis 11.2: NNAL (ng/g)


Study or Subgroup

11.2.1 Change from baseline
Edmiston 2022\*

Subtotal
Test for overall effect: Z = 1.26 (P = 0.21)

Heterogeneity: Not applicable

Total
Test for overall effect: Z = 1.26 (P = 0.21)
Test for subgroup differences: Not applicable

Heterogeneity: Not applicable

Tobacco
Mean
-185.6
SD
157.8
Total
112

112

112

Menthol
Mean
-159.5
SD
157.8
Total
120

120

120
Weight
100.0%

100.0%

100.0%

Mean Difference
IV, Random, 95% CI
-26.10 [-66.73 , 14.53]

-26.10 [-66.73 , 14.53]

-26.10 [-66.73 , 14.53]

Mean Difference
IV, Random, 95% CI


-100

-50

0

50

100


Favours tobacco

Favours menthol


Analysis 11.3: FEV1 (% predicted)


Study or Subgroup

11.3.1 Change from baseline
Edmiston 2022\*

Subtotal
Test for overall effect: Z = 0.79 (P = 0.43)

Heterogeneity: Not applicable

Total
Test for overall effect: Z = 0.79 (P = 0.43)
Test for subgroup differences: Not applicable

Heterogeneity: Not applicable

Tobacco
Mean
-1.04
SD
6.2
Total
100

100

100

Menthol
Mean
-0.37
SD
6.2
Total
112

112

112
Weight
100.0%

100.0%

100.0%

Mean Difference
IV, Random, 95% CI
-0.67 [-2.34 , 1.00]

-0.67 [-2.34 , 1.00]

-0.67 [-2.34 , 1.00]

Mean Difference
IV, Random, 95% CI


-100

-50

0

50

100


Favours menthol

Favours tobacco


Analysis 11.4: FEV1/FVC


Study or Subgroup

11.4.1 Change from baseline
Edmiston 2022\*

Subtotal
Test for overall effect: Z = 0.75 (P = 0.45)

Heterogeneity: Not applicable

Total
Test for overall effect: Z = 0.75 (P = 0.45)
Test for subgroup differences: Not applicable

Heterogeneity: Not applicable

Tobacco
Mean
0.12
SD
4.47
Total
100

100

100

Menthol
Mean
0.58
SD
4.47
Total
112

112

112
Weight
100.0%

100.0%

100.0%

Mean Difference
IV, Random, 95% CI
-0.46 [-1.67 , 0.75]

-0.46 [-1.67 , 0.75]

-0.46 [-1.67 , 0.75]

Mean Difference
IV, Random, 95% CI


-4

-2

0

2

4


Favours menthol

Favours tobacco


Analysis 12.1: Exhaled CO


Study or Subgroup

Kimber 2021
a

Total
Test for overall effect: Z = 0.24 (P = 0.81)

Heterogeneity: Not applicable

Refillable
Mean
10.6
SD
8
Total
20

20

Cartridge
Mean
9.9
SD
7.9
Total
12

12
Weight
100.0%

100.0%

Mean Difference
IV, Random, 95% CI
0.70 [-4.98 , 6.38]

0.70 [-4.98 , 6.38]

Mean Difference
IV, Random, 95% CI


-100

-50

0

50

100


Favours refillable

Favours cartridge


Footnotes

a
This is using data from the 'Tank18' (higher nicotine) refillable arm. Exhaled CO was higher in the 'Tank6' (lower nicotine) arm (12.2, SD 7.9). Result not sensitive to choice of arm.


Analysis 13.1: Smoking cessation


Study or Subgroup
Russell 2021\*

Total
Total events:
Test for overall effect: Z = 1.14 (P = 0.25)

Heterogeneity: Not applicable

Nicotine salt
Events
44
44
Total
145

145

Free-base nicotine
Events
34
34
Total
140

140
Weight
100.0%

100.0%

Risk Ratio
M-H, Random, 95% CI
1.25 [0.85 , 1.83]

1.25 [0.85 , 1.83]

Risk Ratio
M-H, Random, 95% CI


0.01

0.1

1

10

100


Favours free-base

Favours nicotine salt


Analysis 13.2: Product use at 6+ months


Study or Subgroup
Russell 2021\*

Total
Total events:
Test for overall effect: Z = 0.51 (P = 0.61)

Heterogeneity: Not applicable

NSP
Events
62
62
Total
124

124

FBNP
Events
48
48
Total
103

103
Weight
100.0%

100.0%

Risk Ratio
M-H, Random, 95% CI
1.07 [0.82 , 1.41]

1.07 [0.82 , 1.41]

Risk Ratio
M-H, Random, 95% CI


0.01

0.1

1

10

100


Higher with FBNP

Higher with NSP


Analysis 14.1: Smoking cessation


Study or Subgroup
NCT03113136

Total
Total events:
Test for overall effect: Z = 0.73 (P = 0.47)

Heterogeneity: Not applicable

Higher wattage 
Events
8
8
Total
134

134

Lower wattage
Events
11
11
Total
133

133
Weight
100.0%

100.0%

Risk Ratio
M-H, Random, 95% CI
0.72 [0.30 , 1.74]

0.72 [0.30 , 1.74]

Risk Ratio
M-H, Random, 95% CI


0.01

0.1

1

10

100


Favours lower wattage

Favours higher wattage


Analysis 14.2: Adverse events


Study or Subgroup
NCT03113136

Total
Total events:
Test for overall effect: Z = 1.22 (P = 0.22)

Heterogeneity: Not applicable

Higher wattage
Events
95
95
Total
134

134

Lower wattage
Events
103
103
Total
133

133
Weight
100.0%

100.0%

Risk Ratio
M-H, Random, 95% CI
0.92 [0.79 , 1.06]

0.92 [0.79 , 1.06]

Risk Ratio
M-H, Random, 95% CI


0.01

0.1

1

10

100


Favours higher wattage

Favours lower wattage


Analysis 14.3: Serious adverse events


Study or Subgroup
NCT03113136

Total
Total events:
Test for overall effect: Z = 0.01 (P = 0.99)

Heterogeneity: Not applicable

Higher wattage
Events
2
2
Total
134

134

Lower wattage
Events
2
2
Total
133

133
Weight
100.0%

100.0%

Risk Ratio
M-H, Random, 95% CI
0.99 [0.14 , 6.94]

0.99 [0.14 , 6.94]

Risk Ratio
M-H, Random, 95% CI


0.01

0.1

1

10

100


Favours higher wattage

Favours lower wattage


Analysis 15.1: Smoking cessation


Study or Subgroup
Eisenberg 2020
Lucchiari 2022

Total (Wald
a
)
Total events:
Test for overall effect: Z = 1.32 (P = 0.19)

Heterogeneity: Tau² (DL
b
) = 0.00; Chi² = 0.29, df = 1 (P = 0.59); I² = 0%

Non-nicotine EC
Events
3
15
18
Total
127
70

197

Usual care
Events
1
10
11
Total
121
70

191
Weight
9.5%
90.5%

100.0%

Risk Ratio
M-H, Random, 95% CI
2.86 [0.30 , 27.10]
1.50 [0.72 , 3.11]

1.59 [0.80 , 3.19]

Risk Ratio
M-H, Random, 95% CI


0.01

0.1

1

10

100


Favours usual care

Favours non-nicotine EC


Footnotes

a
CI calculated by Wald-type method.

b
Tau² calculated by DerSimonian and Laird method.


Analysis 15.2: Adverse events


Study or Subgroup
Eisenberg 2020

Total
Total events:
Test for overall effect: Z = 4.03 (P < 0.0001)

Heterogeneity: Not applicable

Non-nicotine EC
Events
118
118
Total
127

127

behavioural support only/no support
Events
88
88
Total
121

121
Weight
100.0%

100.0%

Risk Ratio
M-H, Random, 95% CI
1.28 [1.13 , 1.44]

1.28 [1.13 , 1.44]

Risk Ratio
M-H, Random, 95% CI


0.1

0.2

0.5

1

2

5

10


Favours non-nicotine

Favours behavioural


Analysis 15.3: Serious adverse events


Study or Subgroup
Eisenberg 2020
Lucchiari 2022

Total
Total events:
Test for overall effect: Z = 0.27 (P = 0.79)

Heterogeneity: Not applicable

Non-nicotine EC
Events
5
0
5
Total
127
70

197

behavioural support only/no support
Events
4
0
4
Total
121
70

191
Weight
100.0%

100.0%

Risk Ratio
M-H, Random, 95% CI
1.19 [0.33 , 4.33]
Not estimable

1.19 [0.33 , 4.33]

Risk Ratio
M-H, Random, 95% CI


0.01

0.1

1

10

100


Favours non-nicotine

Favours behavioural


Analysis 16.1: Smoking cessation


Study or Subgroup

Walker 2020
a

Total
Total events:
Test for overall effect: Z = 0.84 (P = 0.40)

Heterogeneity: Not applicable

Non-nicotine EC + NRT
Events
20
20
Total
499

499

NRT
Events
3
3
Total
125

125
Weight
100.0%

100.0%

Risk Ratio
M-H, Random, 95% CI
1.67 [0.50 , 5.53]

1.67 [0.50 , 5.53]

Risk Ratio
M-H, Random, 95% CI


0.01

0.1

1

10

100


Favours NRT alone

Favours EC + NRT


Footnotes

a
this represents an EC + patch versus patch only comparison


Analysis 16.2: Adverse events


Study or Subgroup
Walker 2020

Total
Total events:
Test for overall effect: Z = 2.63 (P = 0.009)

Heterogeneity: Not applicable

Non-nicotine EC + NRT
Events
116
116
Total
290

290

NRT
Events
31
31
Total
54

54
Weight
100.0%

100.0%

Risk Ratio
M-H, Random, 95% CI
0.70 [0.53 , 0.91]

0.70 [0.53 , 0.91]

Risk Ratio
M-H, Random, 95% CI


0.01

0.1

1

10

100


Favours EC + NRT

Favours NRT


Analysis 16.3: Serious adverse events


Study or Subgroup
Walker 2020

Total
Total events:
Test for overall effect: Z = 1.00 (P = 0.32)

Heterogeneity: Not applicable

Non-nicotine EC + NRT
Events
27
27
Total
499

499

NRT
Events
4
4
Total
125

125
Weight
100.0%

100.0%

Risk Ratio
M-H, Random, 95% CI
1.69 [0.60 , 4.74]

1.69 [0.60 , 4.74]

Risk Ratio
M-H, Random, 95% CI


0.01

0.1

1

10

100


Favours EC+NRT

Favours NRT


Analysis 17.1: Smoking cessation


Study or Subgroup
Klonizakis 2022

Lee 2019
a

Total (Wald
b
)
Total events:
Test for overall effect: Z = 0.03 (P = 0.98)

Heterogeneity: Tau² (DL
c
) = 0.04; Chi² = 1.57, df = 1 (P = 0.21); I² = 36%

Non-nicotine EC
Events
30
16
46
Total
82
75

157

NRT
Events
25
21
46
Total
82
75

157
Weight
58.3%
41.7%

100.0%

Risk Ratio
M-H, Random, 95% CI
1.20 [0.78 , 1.85]
0.76 [0.43 , 1.34]

0.99 [0.64 , 1.54]

Risk Ratio
M-H, Random, 95% CI


0.01

0.1

1

10

100


Favours NRT

Favours non-nicotine EC


Footnotes

a
0.01 mg/mL of nicotine in e-liquid

b
CI calculated by Wald-type method.

c
Tau² calculated by DerSimonian and Laird method.


Analysis 17.2: Adverse events


Study or Subgroup

Lee 2019
a

Total
Total events:
Test for overall effect: Z = 2.23 (P = 0.03)

Heterogeneity: Not applicable

Non-nicotine EC
Events
5
5
Total
71

71

NRT
Events
13
13
Total
61

61
Weight
100.0%

100.0%

Risk Ratio
M-H, Random, 95% CI
0.33 [0.12 , 0.87]

0.33 [0.12 , 0.87]

Risk Ratio
M-H, Random, 95% CI


0.01

0.1

1

10

100


Favours non-nicotine EC

Favours NRT


Footnotes

a
0.01 mg/mL of nicotine in e-liquid; length of follow-up not defined but presumably over study period


Analysis 17.3: Serious adverse events


Study or Subgroup

Lee 2019
a

Total
Total events:
Test for overall effect: Not applicable

Heterogeneity: Not applicable

Non-nicotine EC
Events
0
0
Total
71

71

NRT
Events
0
0
Total
61

61
Weight

Risk Ratio
M-H, Random, 95% CI
Not estimable

Not estimable

Risk Ratio
M-H, Random, 95% CI


0.01

0.1

1

10

100


Favours non-nicotine EC

Favours NRT

Footnotes

a
0.01 mg/mL of nicotine in e-liquid; length of follow-up not defined but presumably over study period


Analysis 17.4: Change in carbon monoxide (ppm) at 6 months


Study or Subgroup
Klonizakis 2022

Total
Test for overall effect: Z = 1.57 (P = 0.12)

Heterogeneity: Not applicable

Non-nicotine EC
Mean
-6
SD
9.24
Total
82

82

NRT
Mean
-8
SD
6.93
Total
82

82
Weight
100.0%

100.0%

Mean Difference
IV, Random, 95% CI
2.00 [-0.50 , 4.50]

2.00 [-0.50 , 4.50]

Mean Difference
IV, Random, 95% CI


-10

-5

0

5

10


Favours non-nicotine EC

Favours NRT


Analysis 18.1: Smoking cessation


Study or Subgroup
Elling 2023
Martinez 2021

Total (Wald
a
)
Total events:
Test for overall effect: Z = 0.32 (P = 0.75)

Heterogeneity: Tau² (DL
b
) = 0.00; Chi² = 0.28, df = 1 (P = 0.60); I² = 0%

Advice to use EC to quit
Events
28
249
277
Total
157
1167

1324

Advice does not include EC
Events
34
237
271
Total
174
1154

1328
Weight
10.9%
89.1%

100.0%

Risk Ratio
M-H, Random, 95% CI
0.91 [0.58 , 1.43]
1.04 [0.89 , 1.22]

1.02 [0.88 , 1.19]

Risk Ratio
M-H, Random, 95% CI


0.85

0.9

1

1.1

1.2


Favours no EC advice

Favours EC advice


Footnotes

a
CI calculated by Wald-type method.

b
Tau² calculated by DerSimonian and Laird method.


Analysis 18.2: Adverse events


Study or Subgroup
Vickerman 2022

Total
Total events:
Test for overall effect: Z = 0.83 (P = 0.41)

Heterogeneity: Not applicable

Advice to use EC to quit
Events
14
14
Total
26

26

Advice does not include EC
Events
11
11
Total
26

26
Weight
100.0%

100.0%

Risk Ratio
M-H, Random, 95% CI
1.27 [0.72 , 2.26]

1.27 [0.72 , 2.26]

Risk Ratio
M-H, Random, 95% CI


0.01

0.1

1

10

100


Favours EC advice

Favours no EC advice


Analysis 18.3: Serious adverse events


Study or Subgroup
Vickerman 2022

Total
Total events:
Test for overall effect: Not applicable

Heterogeneity: Not applicable

Advice to use EC to quit
Events
0
0
Total
26

26

Advice does not include EC
Events
0
0
Total
26

26
Weight

Risk Ratio
M-H, Random, 95% CI
Not estimable

Not estimable

Risk Ratio
M-H, Random, 95% CI


0.01

0.1

1

10

100


Favours EC advice

Favours no EC advice


Analysis 18.4: Product use at 6+ months


Study or Subgroup
Elling 2023

Total
Total events:
Test for overall effect: Z = 1.48 (P = 0.14)

Heterogeneity: Not applicable

Advice to use EC to quit
Events
16
16
Total
157

157

Advice does not include EC
Events
10
10
Total
174

174
Weight
100.0%

100.0%

Risk Ratio
M-H, Random, 95% CI
1.77 [0.83 , 3.79]

1.77 [0.83 , 3.79]

Risk Ratio
M-H, Random, 95% CI


0.1

0.2

0.5

1

2

5

10


Higher with no EC-advice

Higher with EC advice


Analysis 19.1: Smoking cessation


Study or Subgroup

Baldassarri 2018
a

Walker 2020
a

Total (Wald
b
)
Total events:
Test for overall effect: Z = 2.21 (P = 0.03)

Heterogeneity: Tau² (DL
c
) = 0.00; Chi² = 0.03, df = 1 (P = 0.87); I² = 0%

Nicotine EC + NRT
Events
4
35
39
Total
20
500

520

Non-nicotine EC + NRT
Events
2
20
22
Total
20
499

519
Weight
10.3%
89.7%

100.0%

Risk Ratio
M-H, Random, 95% CI
2.00 [0.41 , 9.71]
1.75 [1.02 , 2.98]

1.77 [1.07 , 2.94]

Risk Ratio
M-H, Random, 95% CI


0.01

0.1

1

10

100


Favours non-nicotine EC

Favours nicotine EC


Footnotes

a
this represents an EC + patch versus non-nicotine EC + patch comparison

b
CI calculated by Wald-type method.

c
Tau² calculated by DerSimonian and Laird method.


Analysis 19.2: Adverse events


Study or Subgroup
Rose 2023\*
Walker 2020

Total (Wald
a
)
Total events:
Test for overall effect: Z = 1.16 (P = 0.24)

Heterogeneity: Tau² (DL
b
) = 0.00; Chi² = 0.29, df = 1 (P = 0.59); I² = 0%

Nicotine EC + NRT
Events
21
138
159
Total
37
317

354

Non-nicotine EC + NRT
Events
15
116
131
Total
33
290

323
Weight
14.0%
86.0%

100.0%

Risk Ratio
M-H, Random, 95% CI
1.25 [0.78 , 1.99]
1.09 [0.90 , 1.31]

1.11 [0.93 , 1.32]

Risk Ratio
M-H, Random, 95% CI


0.1

0.2

0.5

1

2

5

10


Favours nicotine EC

Favours non-nicotine EC


Footnotes

a
CI calculated by Wald-type method.

b
Tau² calculated by DerSimonian and Laird method.


Analysis 19.3: Serious adverse events


Study or Subgroup
Rose 2023\*
Walker 2020

Total (Wald
a
)
Total events:
Test for overall effect: Z = 1.49 (P = 0.14)

Heterogeneity: Tau² (DL
b
) = 0.00; Chi² = 0.01, df = 1 (P = 0.90); I² = 0%

Nicotine EC + NRT
Events
2
18
20
Total
37
500

537

Non-nicotine EC + NRT
Events
3
27
30
Total
33
499

532
Weight
10.2%
89.8%

100.0%

Risk Ratio
M-H, Random, 95% CI
0.59 [0.11 , 3.34]
0.67 [0.37 , 1.19]

0.66 [0.38 , 1.14]

Risk Ratio
M-H, Random, 95% CI


0.01

0.1

1

10

100


Favours nicotine EC

Favours non-nicotine EC


Footnotes

a
CI calculated by Wald-type method.

b
Tau² calculated by DerSimonian and Laird method.


Analysis 19.4: Carbon monoxide (ppm)


Study or Subgroup

19.4.1 change from baseline
Baldassarri 2018
Rose 2023\*

Subtotal (Wald
a
)
Test for overall effect: Z = 1.22 (P = 0.22)

Heterogeneity: Tau² (REML
b
) = 22.69; Chi² = 4.26, df = 1 (P = 0.04); I² = 77%

Total (Wald
a
)
Test for overall effect: Z = 1.22 (P = 0.22)
Test for subgroup differences: Not applicable

Heterogeneity: Tau² (REML
b
) = 22.69; Chi² = 4.26, df = 1 (P = 0.04); I² = 77%

Nicotine EC + NRT
Mean
-9.5
-11.1
SD
3.9
12.8
Total
13
26

39

39

Non-nicotine EC + NRT
Mean
-8.1
-2
SD
3.4
10.2
Total
12
19

31

31
Weight
58.1%
41.9%

100.0%

100.0%

Mean Difference
IV, Random, 95% CI
-1.40 [-4.26 , 1.46]
-9.10 [-15.83 , -2.37]

-4.62 [-12.07 , 2.82]

-4.62 [-12.07 , 2.82]

Mean Difference
IV, Random, 95% CI


-20

-10

0

10

20


Favours nicotine EC

Favours non-nicotine EC


Footnotes

a
CI calculated by Wald-type method.

b
Tau² calculated by Restricted Maximum-Likelihood method.


Analysis 19.5: FeNO (ppb)


Study or Subgroup

19.5.1 6 months
Baldassarri 2018

Subtotal
Test for overall effect: Z = 0.10 (P = 0.92)

Heterogeneity: Not applicable

Total
Test for overall effect: Z = 0.10 (P = 0.92)
Test for subgroup differences: Not applicable

Heterogeneity: Not applicable

Nicotine EC
Mean
2.75
SD
10.5
Total
12

12

12

Non-nicotine EC
Mean
3.11
SD
7.45
Total
18

18

18
Weight
100.0%

100.0%

100.0%

Mean Difference
IV, Random, 95% CI
-0.36 [-7.23 , 6.51]

-0.36 [-7.23 , 6.51]

-0.36 [-7.23 , 6.51]

Mean Difference
IV, Random, 95% CI


-10

-5

0

5

10


Favours nicotine EC

Favours non-nicotine EC


Analysis 19.6: FEV1 (%)


Study or Subgroup

19.6.1 6 months
Baldassarri 2018

Subtotal
Test for overall effect: Z = 1.67 (P = 0.10)

Heterogeneity: Not applicable

Total
Test for overall effect: Z = 1.67 (P = 0.10)
Test for subgroup differences: Not applicable

Heterogeneity: Not applicable

Nicotine EC
Mean
0.0085
SD
0.057
Total
13

13

13

Non-nicotine EC
Mean
-0.037
SD
0.097
Total
19

19

19
Weight
100.0%

100.0%

100.0%

Mean Difference
IV, Random, 95% CI
0.05 [-0.01 , 0.10]

0.05 [-0.01 , 0.10]

0.05 [-0.01 , 0.10]

Mean Difference
IV, Random, 95% CI


-0.1

-0.05

0

0.05

0.1


Favours non-nicotine EC

Favours nicotine EC


Analysis 19.7: FVC (%)


Study or Subgroup

19.7.1 6 months
Baldassarri 2018

Subtotal
Test for overall effect: Z = 1.09 (P = 0.28)

Heterogeneity: Not applicable

Total
Test for overall effect: Z = 1.09 (P = 0.28)
Test for subgroup differences: Not applicable

Heterogeneity: Not applicable

Nicotine EC
Mean
0.0108
SD
0.065
Total
13

13

13

Non-nicotine EC
Mean
-0.0216
SD
0.103
Total
19

19

19
Weight
100.0%

100.0%

100.0%

Mean Difference
IV, Random, 95% CI
0.03 [-0.03 , 0.09]

0.03 [-0.03 , 0.09]

0.03 [-0.03 , 0.09]

Mean Difference
IV, Random, 95% CI


-0.1

-0.05

0

0.05

0.1


Favours non-nicotine EC

Favours nicotine EC


Analysis 19.8: Study product use at 6+ months


Study or Subgroup
Baldassarri 2018

Total
Total events:
Test for overall effect: Z = 0.30 (P = 0.76)

Heterogeneity: Not applicable

Nicotine EC
Events
2
2
Total
4

4

Non-nicotine EC
Events
2
2
Total
5

5
Weight
100.0%

100.0%

Risk Ratio
M-H, Random, 95% CI
1.25 [0.29 , 5.35]

1.25 [0.29 , 5.35]

Risk Ratio
M-H, Random, 95% CI


0.01

0.1

1

10

100


Higher with non nicotine EC

Higher with nicotine EC


Analysis 20.1: Smoking cessation


Study or Subgroup

Morphett 2022b
a

Walker 2020
b

Total (Wald
c
)
Total events:
Test for overall effect: Z = 4.16 (P < 0.0001)

Heterogeneity: Tau² (DL
d
) = 0.00; Chi² = 0.16, df = 1 (P = 0.69); I² = 0%

Nicotine EC + NRT
Events
36
35
71
Total
181
500

681

NRT
Events
9
3
12
Total
174
125

299
Weight
73.4%
26.6%

100.0%

Risk Ratio
M-H, Random, 95% CI
3.85 [1.91 , 7.74]
2.92 [0.91 , 9.33]

3.57 [1.96 , 6.51]

Risk Ratio
M-H, Random, 95% CI


0.01

0.1

1

10

100


Favours NRT

Favours nicotine EC + NRT


Footnotes

a
Based on quit data at 6 months (prior to study cross-over). NRT arm includes patch and gum/lozenge; EC arm includes patch and EC.

b
This represents an EC + patch versus patch only

c
CI calculated by Wald-type method.

d
Tau² calculated by DerSimonian and Laird method.


Analysis 20.2: Adverse events


Study or Subgroup

Bonevski 2021
a

Morphett 2022a
b
Piper 2025
Walker 2020

Nicotine EC + NRT
Events
15
146
31
138
Total
25
619
54
317

NRT
Events
10
225
16
31
Total
25
944
53
54

Risk Ratio
M-H, Random, 95% CI
1.50 [0.84 , 2.67]
0.99 [0.82 , 1.19]
1.90 [1.19 , 3.04]
0.76 [0.58 , 0.99]

Risk Ratio
M-H, Random, 95% CI


0.2

0.5

1

2

5


Favours EC + NRT

Favours NRT

Footnotes

a
NRT not matched between arms

b
Two NRT only arms combined for comparator


Analysis 20.3: Serious adverse events


Study or Subgroup

Bonevski 2021
a

Morphett 2022a
b
NCT02918630
Piper 2025
Walker 2020

Total (Wald
c
)
Total events:
Test for overall effect: Z = 0.42 (P = 0.67)

Heterogeneity: Tau² (DL
d
) = 0.00; Chi² = 0.33, df = 1 (P = 0.56); I² = 0%

Nicotine EC + NRT
Events
1
0
0
0
18
19
Total
25
619
3
54
500

1201

NRT
Events
0
0
0
0
4
4
Total
25
944
4
53
125

1151
Weight
10.2%
89.8%

100.0%

Risk Ratio
M-H, Random, 95% CI
3.00 [0.13 , 70.30]
Not estimable
Not estimable
Not estimable
1.13 [0.39 , 3.27]

1.24 [0.45 , 3.41]

Risk Ratio
M-H, Random, 95% CI


0.01

0.1

1

10

100


Favours EC+NRT

Favours NRT


Footnotes

a
NRT not matched between arms

b
Two NRT only arms combined for comparator

c
CI calculated by Wald-type method.

d
Tau² calculated by DerSimonian and Laird method.


Analysis 21.1: Adverse events


Study or Subgroup
Tattan-Birch 2023

Total
Total events:
Test for overall effect: Z = 0.97 (P = 0.33)

Heterogeneity: Not applicable

Nicotine EC + varenicline
Events
31
31
Total
48

48

Varenicline
Events
24
24
Total
44

44
Weight
100.0%

100.0%

Risk Ratio
M-H, Random, 95% CI
1.18 [0.84 , 1.67]

1.18 [0.84 , 1.67]

Risk Ratio
M-H, Random, 95% CI


0.01

0.1

1

10

100


Favours EC + varenicline

Favours varenicline


Analysis 21.2: Serious adverse events


Study or Subgroup
Tattan-Birch 2023

Total
Total events:
Test for overall effect: Not applicable

Heterogeneity: Not applicable

Nicotine EC + varenicline
Events
0
0
Total
48

48

Varenicline
Events
0
0
Total
44

44
Weight

Risk Ratio
M-H, Random, 95% CI
Not estimable

Not estimable

Risk Ratio
M-H, Random, 95% CI


0.01

0.1

1

10

100


Favours EC + varenicline

Favours varenicline


Analysis 22.1: Adverse events


Study or Subgroup

Higgins 2024
a

Total
Total events:
Test for overall effect: Z = 0.63 (P = 0.53)

Heterogeneity: Not applicable

Nicotine EC + VLNC
Events
135
135
Total
158

158

VLNC
Events
75
75
Total
85

85
Weight
100.0%

100.0%

Risk Ratio
M-H, Random, 95% CI
0.97 [0.88 , 1.07]

0.97 [0.88 , 1.07]

Risk Ratio
M-H, Random, 95% CI


0.2

0.5

1

2

5


Favours EC + VLNC

Favours VLNC


Footnotes

a
EC arm combines data from preferred flavour and tobacco flavour arms


Analysis 22.2: Serious adverse events


Study or Subgroup

Higgins 2024
a

Total
Total events:
Test for overall effect: Z = 0.34 (P = 0.73)

Heterogeneity: Not applicable

Nicotine EC + VLNC
Events
6
6
Total
158

158

VLNC
Events
4
4
Total
85

85
Weight
100.0%

100.0%

Risk Ratio
M-H, Random, 95% CI
0.81 [0.23 , 2.78]

0.81 [0.23 , 2.78]

Risk Ratio
M-H, Random, 95% CI


0.2

0.5

1

2

5


Favours EC + VLNC

Favours VLNC


Footnotes

a
EC arm combines data from preferred flavour and tobacco flavour arms


Analysis 22.3: Change in carbon monoxide


Study or Subgroup

Higgins 2024
a

Total
Test for overall effect: Z = 2.37 (P = 0.02)

Heterogeneity: Not applicable

EC + VLNC
Mean
-6.42
SD
20.67
Total
69

69

VLNC
Mean
0.73
SD
13.6
Total
63

63
Weight
100.0%

100.0%

Mean Difference
IV, Random, 95% CI
-7.15 [-13.07 , -1.23]

-7.15 [-13.07 , -1.23]

Mean Difference
IV, Random, 95% CI


-20

-10

0

10

20


Favours EC + VLNC

Favours VLNC


Footnotes

a
The EC + VLNC data comes from the preferred flavour study arm only (i.e. excluding the tobacco flavour arm data)


Analysis 22.4: Change in NNAL (pmol/mg creatinine)


Study or Subgroup

Higgins 2024
a

Total
Test for overall effect: Z = 0.27 (P = 0.79)

Heterogeneity: Not applicable

EC + VLNC
Mean
-0.5
SD
3.73
Total
53

53

VLNC
Mean
-0.79
SD
7.21
Total
57

57
Weight
100.0%

100.0%

Mean Difference
IV, Random, 95% CI
0.29 [-1.83 , 2.41]

0.29 [-1.83 , 2.41]

Mean Difference
IV, Random, 95% CI


-10

-5

0

5

10


Favours EC + VLNC

Favours VLNC


Footnotes

a
The EC + VLNC data comes from the preferred flavour study arm only (i.e. excluding the tobacco flavour arm data)
