# Supplementary material for: Electronic cigarettes for smoking cessation
Source: Cochrane Database Syst Rev. 2025 Nov 10;2025(11):CD010216. doi: 10.1002/14651858.CD010216.pub10 (PMC12599494; doi:10.1002/14651858.CD010216.pub10)
Supplement: Supplementary file 8 — Supplementary material 8 Protocol for living systematic review [file CD010216-SUP-08-other.html]

Protocol for living systematic review


# Supplementary material 8 to: Electronic cigarettes for smoking cessation

Lindson N, Livingstone-Banks J, Butler AR, McRobbie H, Bullen CR, Hajek P, Wu AD, Begh R, Theodoulou A, Notley C, Rigotti NA, Turner T, Fanshawe T, Hartmann-Boyce J
  
https://doi.org/10.1002/14651858.CD010216.pub10

The material in this section has been supplied by the author(s) for publication under a Licence for Publication and the author(s) are solely responsible for the material. Cochrane has reviewed this material, but Cochrane has not copyedited, formatted or proofread. Cochrane accordingly gives no representations or warranties of any kind in relation to, and accepts no liability for any reliance on or use of, such material.

Back to top

# Protocol for living systematic review

### Justification for ‘Living Review’ status

Living systematic reviews (LSRs) offer a new approach to updating reviews, in which the review is continually updated by incorporating relevant new evidence as it becomes available [1]. Previous versions of this Cochrane review of electronic cigarettes (ECs) for smoking cessation have informed policy worldwide [2, 3]. This update has found high degrees of uncertainty (low- and very low-certainty evidence) for most outcomes, due to the small number of included randomized controlled trials, and the resulting imprecision in effect estimates. This means that some conclusions are likely to change substantially as new evidence emerges.

On average, Cochrane reviews are updated every three to four years. For EC, where the evidence base is rapidly evolving, this schedule impedes the ability of the review to provide the most up-to-date evidence to decision-makers. As EC use, availability, and design changes, policymakers are frequently drawing on this review to inform decisions, so it is imperative that it is up-to-date to ensure decisions are being made on the basis of the entirety of the evidence. Regular updates have the potential to strengthen the existing conclusions of the review or to change conclusions where conflicting evidence or evidence on new outcomes emerges (e.g. comparisons between EC and other interventions; longer-term safety data).

### Objective of the change to ‘Living Review’ status

To implement approved Cochrane LSR methods to provide an up-to-date, accessible, engaging, and unbiased review of the evidence on the effect and safety of using EC to quit smoking.

### LSR methodological considerations

The methods outlined below are specific to maintaining this review of *Electronic cigarettes for smoking cessation* as an LSR on the Cochrane Library. These methods will be ‘active’ immediately upon publication of this update. Core review methods, such as the criteria for considering studies in the review and assessment of risks of bias, are unchanged and are detailed in the main body of the review. Below we outline the methods for which specific considerations apply as a result of the change to ‘living’ status.

#### Search methods for identification of studies

We will conduct database searches monthly, beginning December 2020. These searches will be of the Cochrane Central Register of Controlled Trials (CENTRAL), MEDLINE, Embase, PsycINFO, and clinical trial registries, as detailed in the main body of the review. The funders of this LSR – Cancer Research UK (CRUK) - already run monthly searches of the EC evidence, and so we will work alongside their health information officer to ensure that we are identifying all the relevant literature with our searches. We will review our search strategies on an ongoing basis every 12 months, as indexing terms and keywords may change, and new search filters may be published. Such changes will be managed by input from experienced information specialists.

#### Selection of studies

We will immediately screen any new citations retrieved by the monthly searches using [99], undertaking dual screening of title and abstract, and then full text, by independent review authors. Where we find multiple citations of the same study, we will group them into one study record with a single study ID. One review author (AB) will contact corresponding authors of potentially relevant ongoing studies as they are identified and ask them to advise when results are available, or to share early or unpublished data. Based on the information and projected time scales shared, we will contact corresponding authors on an ongoing basis to retrieve new evidence as it becomes available.

#### Data synthesis

Whenever we identify new studies relevant to the review, we will extract the relevant data and assess the risks of bias as detailed in the main body of the review. We will highlight the availability of this new evidence on both the Cochrane Library and on our own dedicated website. We will incorporate the new data into meta-analyses and tables in RevMan [4] and supplementary data files, and carry out GRADE assessments [5]. We will conduct a full update of the review (full incorporation and interpretation of all new data within the review and re-publishing) when the accumulating evidence leads to changes in any one of:

- the direction of effect or clinical significance of the findings for one or more outcomes;
- the certainty (e.g. GRADE rating) of one or more outcomes;
- the availability of studies investigating new settings, populations, interventions, comparisons, or outcomes.

Formal sequential meta-analysis approaches will not be used for updated meta-analyses, in line with Cochrane guidance for LSRs.

### Future updates of review methods

The LSR approach acknowledges that reviews may cease to need to be ‘living’ over time, as the review findings become stable, or the question is no longer a priority for decision-makers [1]. We continue to evaluate the LSR approach, including the likely benefits of and challenges to continuing this methodology for this evidence base, and whether such an approach remains warranted. We held evaluation meetings in 2022, 2023, and 2024 and plan to hold a meeting in 2025. If the evidence is high certainty for all outcomes and all comparisons at that point, meaning further studies are judged very unlikely to impact the effect estimate, we would consider ceasing living mode for this review. If, as is more likely, some or all outcomes are not yet certain, we will facilitate discussions within the author team and Cochrane, as well as engaging with a wider PPI panel and key decision-makers, e.g. policymakers, in order to determine next steps. If the decision is made to continue in living mode, we will review, and if necessary revise, the living review methods described in this Appendix before continuing.

## References

1. Brooker J, Synnot A, McDonald S, Elliott J, Turner T, Hodder R, et al. Guidance for the production and publication of Cochrane living systematic reviews: Cochrane Reviews in living mode; December 2019. community.cochrane.org/sites/default/files/uploads/inline-files/Transform/201912\_LSR\_Revised\_Guidance.pdf (accessed 30 July 2020).
2. Hartmann-Boyce J, McRobbie H, Bullen C, Begh R, Stead LF, Hajek P. Electronic cigarettes for smoking cessation. Cochrane Database of Systematic Reviews 2016, Issue 9. Art. No: CD010216. [DOI: 10.1002/14651858.CD010216.pub3]
3. McRobbie H, Bullen C, Hartmann-Boyce J, Hajek P. Electronic cigarettes for smoking cessation and reduction. Cochrane Database of Systematic Reviews 2014, Issue 12. Art. No: CD010216. [DOI: 10.1002/14651858.CD010216.pub2]
4. Review Manager Web (RevMan Web). Version 4.12.0. The Cochrane Collaboration, 2022. Available at revman.cochrane.org.
5. GRADEpro GDT. Version accessed 30 July 2020. Hamilton (ON): McMaster University (developed by Evidence Prime), 2020. Available at gradepro.org.
