# Supplementary material for: Electronic cigarettes for smoking cessation
Source: Cochrane Database Syst Rev. 2025 Nov 10;2025(11):CD010216. doi: 10.1002/14651858.CD010216.pub10 (PMC12599494; doi:10.1002/14651858.CD010216.pub10)
Supplement: Supplementary file 9 — Supplementary material 9 Toxins/carcinogen names and abbreviations [file CD010216-SUP-09-other.html]

Toxins/carcinogen names and abbreviations


# Supplementary material 9 to: Electronic cigarettes for smoking cessation

Lindson N, Livingstone-Banks J, Butler AR, McRobbie H, Bullen CR, Hajek P, Wu AD, Begh R, Theodoulou A, Notley C, Rigotti NA, Turner T, Fanshawe T, Hartmann-Boyce J
  
https://doi.org/10.1002/14651858.CD010216.pub10

The material in this section has been supplied by the author(s) for publication under a Licence for Publication and the author(s) are solely responsible for the material. Cochrane has reviewed this material, but Cochrane has not copyedited, formatted or proofread. Cochrane accordingly gives no representations or warranties of any kind in relation to, and accepts no liability for any reliance on or use of, such material.

Back to top

# Toxins/carcinogen names and abbreviations

| Abbreviation | Name |
| --- | --- |
| - | 1-Hydroxyfluorene |
| - | 1-Hydroxyphenanthrene |
| - | 1-Hydroxypyrene |
| 2-HPMA | 2-hydroxypropylmercapturic acid |
| - | 2-Hydroxyfluorene |
| - | 2-Hydroxyphenanthrene |
| - | 2-Naphthol |
| - | 3-, 4-Hydroxyphenanthrenes |
| 3-HPMA | 3-hydroxypropylmercapturic acid |
| - | 3-Hydroxyfluorene |
| AAMA | *N*-acetyl-*S*-(carbamoylethyl)-L-cysteine (synonym: 2-carbamoylethylmercapturic acid) |
| CEMA/CNEMA | 2-cyanoethylmercapturic acid; referred to as 'acrylonitrile' in Pulvers 2018 |
| - | Formic acid |
| HEMA | 2-hydroxyethylmercapturic acid |
| HMPMA/HPMMA | 3-hydroxy-1-methyl propylmercapturic acid |
| MHBMA | 2-hydroxy-3-buten-1-ylmercapturic acid |
| MMA | N-nitrosodimethyamine |
| NNAL | 4-(methylnitrosamino)-1-(3-pyridyl)-1-butanol |
| PheT | Phenanthrene tetraol |
| PMA | phenylmercapturic acid; referred to as 'benzene' in Pulvers 2018 |
| S-PMA | S-phenylmercapturic acid |
