# Supplementary material for: Electronic cigarettes for smoking cessation
Source: Cochrane Database Syst Rev. 2025 Nov 10;2025(11):CD010216. doi: 10.1002/14651858.CD010216.pub10 (PMC12599494; doi:10.1002/14651858.CD010216.pub10)
Supplement: Supplementary file 10 — Supplementary material 10 Changes in methods between living review updates [file CD010216-SUP-10-other.html]

Changes in methods between living review updates


# Supplementary material 10 to: Electronic cigarettes for smoking cessation

Lindson N, Livingstone-Banks J, Butler AR, McRobbie H, Bullen CR, Hajek P, Wu AD, Begh R, Theodoulou A, Notley C, Rigotti NA, Turner T, Fanshawe T, Hartmann-Boyce J
  
https://doi.org/10.1002/14651858.CD010216.pub10

The material in this section has been supplied by the author(s) for publication under a Licence for Publication and the author(s) are solely responsible for the material. Cochrane has reviewed this material, but Cochrane has not copyedited, formatted or proofread. Cochrane accordingly gives no representations or warranties of any kind in relation to, and accepts no liability for any reliance on or use of, such material.

Back to top

# Changes in methods between living review updates

The protocol did not specify a minimum follow-up period for data on adverse events. As of the 2016 update, we amended the Methods section to clarify that we will exclude follow-up data at less than a week.

The original version of this review included reduction as a secondary outcome. The 2016 update removed reduction as an outcome, to bring the review into line with other reviews of cessation treatments produced by the Cochrane Tobacco Addiction Group and to prevent substantial overlap with the update of the Group's review of interventions for harm reduction.

As prespecified in the 2016 update, in the 2020 update, we excluded non-intervention studies. In the 2020 update, we also added an appendix with a protocol setting out our plans to convert this review into a living systematic review in the future.

As specified in an amendment in June 2021, we now include a new secondary outcome: number of people still using study product (EC or pharmacotherapy) at longest follow-up (6+ months).

There were too few studies to conduct subgroup analyses to investigate differences between studies, such as: intensity of behavioural support used; EC type (e.g. cartridge; refillable; pod; disposable); instructions for EC use (e.g. length of provision, choice); and type of participant (e.g. experience of EC use). For most analyses, there were also too few studies to conduct funnel plots.

In the update published in 2024, we added a new outcome - association between withdrawal and cessation measured at six months or longer - and three network meta-analyses (which have since been removed). These were prespecified in a published protocol that can be found at the following link: https://osf.io/gznsr.

As of 2025, we changed our meta-analyses from fixed-effect to random-effects models in accordance with evolving guidance. We also removed the three network meta-analyses introduced in the 2023 update as announced in our February 2025 protocol update.

In the next update of this review, in response to editorial feedback from Cochrane and because of the growth in the RCT evidence based, we will no longer include single arm studies where all participants receive EC (see September 2025 protocol update).
