# Supplementary material for: Electronic cigarettes for smoking cessation
Source: Cochrane Database Syst Rev. 2025 Nov 10;2025(11):CD010216. doi: 10.1002/14651858.CD010216.pub10 (PMC12599494; doi:10.1002/14651858.CD010216.pub10)
Supplement: Supplementary file 11 — Supplementary material 11 Adverse events data not contributing to meta‐analyses [file CD010216-SUP-11-other.html]

Adverse events data not contributing to meta-analyses


# Supplementary material 11 to: Electronic cigarettes for smoking cessation

Lindson N, Livingstone-Banks J, Butler AR, McRobbie H, Bullen CR, Hajek P, Wu AD, Begh R, Theodoulou A, Notley C, Rigotti NA, Turner T, Fanshawe T, Hartmann-Boyce J
  
https://doi.org/10.1002/14651858.CD010216.pub10

The material in this section has been supplied by the author(s) for publication under a Licence for Publication and the author(s) are solely responsible for the material. Cochrane has reviewed this material, but Cochrane has not copyedited, formatted or proofread. Cochrane accordingly gives no representations or warranties of any kind in relation to, and accepts no liability for any reliance on or use of, such material.

Back to top

# Adverse events data not contributing to meta-analyses

Randomized controlled and crossover trials[1]

|  |  |  |  |  |
| --- | --- | --- | --- | --- |
| **Study ID** | **Intervention/ comparator** | **Time point** | **Data** | **Between-group difference**[2] **(↑ more AEs in EC/higher dose EC arm; ↔ equivocal; ↓ fewer AEs in EC/higher dose arm)** |
| Adriaens 2014 | All received EC by final follow-up | 8 months | Did not systematically collect data on AEs but did collect 'complaints' through online diaries; across all groups bad taste, dry mouth/throat, irritated mouth/throat, dizziness, headache, nausea, and increased heart rate/palpitations; rates were not provided | ↓ |
| Avila 2024 | Nicotine EC v ONP;  Nicotine EC v no support (continued combustible cigarette) | 4 weeks | “Participants in the nicotine pouch group were more likely to report having a cough throughout the day and shortness of breath when exercising or walking up the stairs every day in the first week of the intervention period (N = 5 for cough and N = 6 for shortness of breath) than those in the EC group (N = 3 for cough and N = 1 for shortness of breath). The frequency of cough and shortness of breath decreased and became similar across groups by week 4.” | ↓ |
| Baldassarri 2018 | Nicotine EC + NRT v no-nicotine EC + NRT | NS | AEs only reported narratively, across whole sample. The most commonly reported side effects among all participants were cough (30%), sore throat (22.5%), increased appetite (17.5%), and vivid dreams (17.5%) (no significant differences by treatment group). | ↔ |
| Begh 2021 | Nicotine EC versus usual care | 2 months, n = 157 per group | AEs by type. n reporting any AE in past 24 hr 90.4% control, 92.1% EC  Throat/mouth irritation, control: 12.8, EC: 16.6%  Cough, control: 30.6%, EC: 26.1%  Headache, control: 14%, EC: 13.4%  Palpitations, control: 8.2%, EC: 9.6%  Nausea, control: 5.1%, EC: 4.5%  Dry mouth, control: 28.7%, EC: 26.1%  Dizziness, control: 7.7%, EC: 10.9%  Shortness of breath, control: 31.2%, EC: 24.3%  Stomach pain, control: 24.6%, EC: 7.7% | ↑ throat irritation  ↓ cough  ↓ headache  ↑ palpitations  ↓ nausea  ↓ dry mouth  ↑ dizziness  ↓ shortness of breath  ↓ stomach pain |
| Bonafont Reyes 2022 | Nicotine EC v NRT | 12 weeks | “There was a trend towards decreased dyspnea and COPD symptoms… in the EC arm compared to the NRT arm” | ↓ |
| Caponnetto 2013a\* | Nicotine EC v no-nicotine EC | 3 and 12 months | No difference in frequency of AEs between groups. 5 most frequently reported at baseline: cough (26%), dry mouth (22%), shortness of breath (20%), throat irritation (17%), and headache (17%). In all groups the frequency of AEs decreased significantly over time, with the exception of throat irritation. | ↔- |
| Edmiston 2022\* | Nicotine EC (comparison of flavours) v behavioural support only | 12 weeks | “33 total AEs were reported by 13 test 1 subjects; 3 subjects in test 1… experienced an AE definitely related to study product” (no breakdown by group) |  |
| Felicione 2019 | Nicotine EC v non-nicotine EC | 4 weeks | Total n = 25. Number of participants reporting: headaches (n = 8), throat irritation (n = 6), nausea (n = 4), dry mouth (n = 3), coughing (n = 3), dizziness (n = 2), shortness of breath (n = 2). 52% of participants reported one of these effects during intervention period. Compared frequency between groups: “items with the largest effect sizes included throat irritation (p=.12, OR = 5.6) and headache P = .10; OR = 4.5). Other side effect items revealed small effect sizes, with ORs ranging from 0.04 to 1.7 (Ps = 0.10 to 0.86).” | ↑ throat irritation  ↑ headache |
| Hajek 2019 | Nicotine EC v NRT | Weeks 1 to 6 | Only prespecified outcomes collected. Shortness of breath: 21% EC; 22.9% NRT. Wheezing 23.5% EC; 21.1% NRT. Cough 30.8% EC; 39.8% NRT. Phlegm 25.1% EC; 36.9% NRT. Nausea: 31% EC; 38% NRT. Throat/mouth irritation: 65% EC; 51% NRT. Sleep disturbances 64% EC; 68% NRT. *Of these SEVERE: nausea 6.6% EC; 6.5% NRT. Throat/mouth irritation 5.9% EC, 3.8% NRT. Sleep disturbances 13% in both arms.* | Shortness of breath ↓  Wheezing ↑  Cough ↓  Phlegm ↓  Nausea↓  Throat/mouth irritation ↑  Sleep disturbances ↓ |
| Ioakeimidis 2018 | Nicotine EC v varenicline | 12 weeks | N = 27 per arm. Nausea 1 EC; 3 varenicline. Sleep disorders 2 EC; 2 varenicline. | Nausea ↓  Sleep disorders ↔ |
| Kimber 2021 | Nicotine EC (comparisons by nicotine level and device type) | 2 weeks | “There was no main effect of time on the overall adverse effects reported following EC use in the lab sessions, F(1.51, 70.92) = 0.875, p = .394, ƞ2p = .018, no main effect of condition F(2, 47) = 0.864, p = .428, ƞ2p = .035, no significant time X condition interaction F(3.02, 70.92) = 0.636, p = .595, ƞ2p = .026…For the overall adverse effects, there were no changes over time or differences between conditions.” | ↔ |
| Kumral 2016 | Nicotine EC v CBT | 3 months | Only measured sinonasal symptoms. SNOT 22 (lower = better) Significant declines in both groups between baseline and 3 months with significantly lower scores in non-EC group at 3m (P = 0.0001) | Sinonasal symptoms ↑ |
| Lucchiari 2022 | Nicotine EC v non-nicotine EC[3] | 6 months | 6 months; N = 52 nicotine; 51 non-nicotine. Burning throat: 15.9% nicotine; 5.6% non-nicotine. Cough 5.8% nicotine, 2.8% non-nicotine. Nausea 5.8% nicotine, 7% non-nicotine. Headache 0% nicotine; 1.4% non-nicotine. Insomnia 1.4% nicotine; 0 non-nicotine. Stomach ache 4.3% nicotine; 4.2% non-nicotine. Confusion 1.4% nicotine; 0 non-nicotine.    12 months: denominators unclear. Burning throat: 7.8% nicotine; 1.2% non-nicotine. Cough 3.8% nicotine, 0% non-nicotine. Nausea 4.1% nicotine, 5% non-nicotine. Stomach ache 4.3% nicotine; 4.5% non-nicotine. | Burning throat 6m ↑; 12m ↑  Cough 6m ↑; 12m ↑  Nausea 6m ↓; 12m ↔  Headache 6m ↓  Insomnia 6m ↑  Stomach ache 6m ↔; 12m ↔  Confusion 6m ↑ |
| Morris 2022\* | Nicotine EC, variations on nicotine strength, flavour, and salt vs. freebase | 9 days | “a total of 35 mild and three moderate AEs were reported by 27 (34%) subjects across study products. The most frequent event was headache, experienced by six (8%) subjects. All remaining events were reported by four or fewer (≤ 5%) subjects each. The PIs considered two events {throat tightness [*my*blu™ Intense Fresh Melon 2.5%] and oropharyngeal discomfort [*my*blu™ Gold Leaf 2.4%]} to be likely related to study product and one event {headache [*my*blu™ Intense Fresh Mint 2.4%]} to be possibly related. All remaining events were considered to be unlikely related/ unrelated to study product.” |  |
| Ozga-Hess 2019 | Nicotine EC v traditional cigarette | 4 weeks | Participants were asked to rate throat irritation, cough, dry mouth, nausea, and dizziness on scale of 0 - 100 (high score = more serious). Throat irritation: 37.4 (32.2) EC, 30.87 (28.8) TC. Cough 44.7 (32.2) EC, 40.2 (32.2) TC. Dry mouth: 46.9 (30.3) EC, 41.8 (31.3) TC. Comparison not provided for nausea and dizziness, but authors state: “The effects of nausea (56.2% vs. 65.8-75.3%, respectively) and dizziness (56.9% vs. 63.6-74.7%, respectively) were reported on slightly fewer days for ECIG+OB participants who made the choice to quit, relative to other subgroups (p’s < .05).” | Throat irritation ↑  Cough ↑  Dry mouth ↑ |
| Pratt 2022 | Nicotine EC v behavioural support only | 26 weeks | Average number of symptoms 1.9 (SD 2.2) in EC group and 2.1 (SD 2.0) in control. No statistically significant between group difference. | ↔ |
| Pope 2024 | Nicotine EC v referral to stop smoking services only | 26 weeks | In the EC arm 136 of 310 participants (43.9%) experienced dry cough, and 104 of 310 participants (33.5%) experienced throat & mouth irritation. In the referral information arm 138 of 292 (47.2%) participants experienced dry cough, and 117 of 293 participants (33.5%) experienced throat & mouth irritation | Dry cough ↔  Throat & mouth irritation ↔ |
| Pulvers 2020 | Nicotine EC v traditional cigarette | 2, 6 weeks | Respiratory symptoms as measured with the American Thoracic Society Questionnaire “The e-cigarette group had significantly greater reductions in …respiratory symptoms (RR, 0.63 [95%CI, 0.47-0.85]; P = .002) …than the cigarettes as usual group.” | ↓ |
| Rabenstein 2024 | Nicotine EC v counselling + NRT | 3 months | “The e-cigarette group showed significantly more irritation of the throat than the control group (p = 0.04), while participants in the control group reported a greater increase in appetite (p = 0.003). Regarding the other clinical symptoms, there were no significant differences between the two groups, nor were there significant differences in the sense of smell and taste.” | Throat irritation ↑  Increased appetite ↓  Clinical symptoms ↔  Sense of smell/taste ↔ |
| Tseng 2016 | Nicotine EC v non-nicotine EC | 1 and 3 weeks | “Reported side effects”: 34.1% for intervention and 17.5% for placebo group at week 1 (P = .09); 22.5% for intervention and 10.3% for placebo group at week 3 (P = .14). “Common side effects included mouth or throat irritation, cough, insomnia or difficulty sleeping, abnormal dreams, headache and fatigue.” | ↑ |
| Tuisku 2024 | Nicotine EC v varenicline v placebo | 52 weeks | AEs leading to discontinuation of a study treatment: EC 15 (9.9%), Varenicline 27 (17.6%), placebo 14 (9.2%). | Vs varenicline ↓  Vs placebo ↑ |
| Vojjala 2025 | Nicotine EC v NRT | 6 months | Chronic obstructive pulmonary disease Assessment Test (CAT) – mean baseline score: EC 20, NRT 21; mean score at 6 months: EC 16, NRT 22; mean change from baseline: EC -4, NRT -1.  Asthma control test score (ACT) – mean baseline score 16 for both study arms; mean scores at 6 months: EC 19, NRT 17; mean change from baseline at 6 months: EC 3, NRT 1. | CAT ↓  ACT ↓ |
| Yingst 2020 | Nicotine EC (36 mg/ml; refillable) versus nicotine EC (24 mg/ml; cig-a-like) | 3 weeks | N = 17. Score range 0 (not at all) to 10 (extremely)  Dizziness: 0.9 (1.4) refillable; 0.7 (1.0) cartridge  Nausea 0.5 (1.1) refillable; 0.5 (1.1) cartridge | Dizziness ↑  Nausea ↔ |

## Cohort studies (all nicotine EC)

|  |  |  |  |
| --- | --- | --- | --- |
| **Study ID** | **Time point** | **Data** | **Frequency over time**[4] **(↓ decline; ↔ equivocal; ↑ increase)** |
| Edwards 2023 | 6 months | N followed up = 26. 41 events recorded during the duration of the study. 27 were considered to be possibly, probably or definitely related to the study treatment. Of these 27 adverse events 21 (78%) occurred within the first 4 weeks of the treatment period. % experiencing the following AEs reported: throat irritation 29.6%; headache 25.9%; cough/chest irritation 18.5%; nausea 14.8%; breathing difficulty 3.7%; gastroesophageal reflux & oesophagistis 3.7%; heart palpitations 3.7%. | ↓ |
| Caponnetto 2013b\* | 1 year | Nausea 2/14 (14.4%) participants,  throat irritation 2/14 (14.4%) participants, headache 2/14 participants, dry cough in 4/14 (28.6%) participants. “Diminished substantially by week-24” | ↓ |
| Caponnetto 2021\* | 24 weeks | 7.8% reported dry cough during the course of the study, 5.4% reported headache, and 2.7% reported throat irritation. No data on frequency over time. |  |
| Auer 2024 | 1 week & 6 months | 1 week; N followed up = 405: “The most commonly reported symptoms were dry mouth (34%, 95% CI: 29-39%), mouth/throat irritation (23%, 95% CI: 19-27%), and cough (25%, 95% CI: 21-29%). Participants rarely reported headache (7%, 95% CI: 4-9%), shortness of breath (3%, 95% CI: 1-5%), dizziness (4%, 95% CI: 2-6%), and heart palpitations/tachycardia (2%, 95% CI: 1-3%).”  6 months; N followed up = 256: “The most commonly reported symptoms were less frequent compared to the week 1 visit: dry mouth (18%, 95% CI: 14-23%, OR=0.27, 95 % CI 0.17 to 0.43), mouth/throat irritation (11%, 95% CI: 7-15%,OR=0.36, 95 % CI 0.22 to 0.59), cough (12%, 95% CI: 8-16%, OR=0.50, 95 % CI 0.33 to 0.77). Other symptoms displayed no statistically significant changes after 6 months.” | Dry mouth, mouth/throat irritation; cough ↓    Headache, shortness of breath, dizziness; heart palpitations/tachycardia ↔ |
| Goniewicz 2017 | 2 weeks | “Significant improvements in chest tightness (35% baseline, 10% week 1, 5% week 2; *p* = .024) and visual disturbances (25% baseline, 5% week 1, 0% week 2; *p* = .020) were observed. Subjects reported non-significant improvement in daytime cough (80% baseline, 70% week 1, 65% week 2), difficulty concentrating (65% baseline, 35% weeks 1 and 2, respectively), irritability (60% baseline, 50% week 1, 40% week 2), and presence of phlegm (75% baseline, 65% week 1, 50% week 2).” | ↓ |
| Hajek 2015a | 4 weeks | Throat irritation and minor coughing reported (incidence not quantified). One incident of a leak from the EVOD system which resulted in mouth irritation; medical treatment was not sought and the incident was resolved by washing the lip with water. |  |
| Hickling 2019 | 6 weeks | Most commonly reported adverse effects (endorsed in response to named adverse effects): throat irritation (13/46), dry cough (9/46), dry mouth (7/46). “No significant changes in the reporting of adverse effects between baseline and week 6 (all p > 0.05).” | ↔ |
| Humair 2014 | NS | Reports only that participants did not experience any AEs |  |
| NCT02648178 | NS | Reports no events in 19 participants |  |
| Nides 2014\* | 12 weeks | 12 participants experienced 15 AEs and all but one (throat irritation) were classified as mild |  |
| Oncken 2015[5] | 2 weeks | Cough in 19% of participants (5/27) , mouth/throat irritation in 15% (4/27), nausea in 4% (1/27), headache in 4% (1/27), and “other” in 4% (1/27) (irritability, stomach cramps). One severe adverse event (itchy throat and cough) in participant with a history of childhood asthma; the participant was discontinued from EC use and symptoms resolved. |  |
| Polosa 2011\* | 2 years | Most commonly reported AEs were throat irritation (8.7%), mouth irritation (8.7%), dry cough (13.1%), dry mouth (4.3%), and headache (4.3%); stable throughout the study. Dizziness and nausea reported at the start of the study but disappeared by 24 months. | Throat/mouth irritation ↔  Cough ↔  Dry mouth ↔  Headache ↔  Dizziness ↓  Nausea ↓ |
| Polosa 2014b\* | 6 months | Throat/mouth irritation (35.6%), dry throat/mouth (28.9%), headache (26.7%) and dry cough (22.2%) were frequently reported at study start but all decreased in frequency over time | ↓ |
| Pratt 2016 | 4 weeks | 58% of participants (11/19) reported at least one AE during study period. “Self-reported side effects were generally mild and short-lived…including dry/sore throat, mild nausea, and cough. Among those who reported side effects, 55% experienced only one symptom for only one week, 37% experienced more than one symptom for 1–2weeks, and only 1 person had a symptom for >2 weeks (mild cough for 4 weeks)” | ↓ |
| Scheibein 2020 | 12 weeks | N = 9. 1 participant each reported runny nose, bleeding nose, slight sweating and dizziness, increased phlegm, burning sensation. 3 reported coughing. 4/9 reported improvements in physical symptoms (breathing better n = 3; less coughing n = 1; being more ‘active’ n = 1; having more ‘energy’ n = 1). |  |
| Sifat 2024 | 8 weeks | No participants in either study arm (n = 30 in each) reported any adverse events. | ↔ |
| Stein 2016 | 9 weeks | 4/12 (33%) participants. 4 reported “headaches,” three reported “coughing” “increased appetite,” “difficulty concentrating,” and “anxious or nervous,” two reported “change in how things taste,” “sore throat,” and “sleep problems,” and one reported “dry mouth,” “dizziness,” and “desire or craving to smoke.” |  |
| Van Staden 2013\* | 2 weeks | Little data reported. One dropout due to illness (headache and fever) |  |
| Wadia 2016 | 2 weeks | None of the participants reported AEs |  |

[1] Excludes NCT02918630 2016 where sample size very small (4 int; 3 control) and AEs reported in detail but not able to synthesise. See NCT record for full information.

[2] Where specified

[3] Third non EC arm did not have AE data collected

[4] Where reported

[5] Randomized crossover arm but all groups received nicotine EC so included in ‘cohort’ table for our purposes
