# Supplementary material for: Electronic cigarettes for smoking cessation
Source: Cochrane Database Syst Rev. 2025 Nov 10;2025(11):CD010216. doi: 10.1002/14651858.CD010216.pub10 (PMC12599494; doi:10.1002/14651858.CD010216.pub10)
Supplement: Supplementary file 12 — Supplementary material 12 Serious adverse events data not contributing to meta‐analyses [file CD010216-SUP-12-other.html]

Serious adverse events data not contributing to meta-analyses


# Supplementary material 12 to: Electronic cigarettes for smoking cessation

Lindson N, Livingstone-Banks J, Butler AR, McRobbie H, Bullen CR, Hajek P, Wu AD, Begh R, Theodoulou A, Notley C, Rigotti NA, Turner T, Fanshawe T, Hartmann-Boyce J
  
https://doi.org/10.1002/14651858.CD010216.pub10

The material in this section has been supplied by the author(s) for publication under a Licence for Publication and the author(s) are solely responsible for the material. Cochrane has reviewed this material, but Cochrane has not copyedited, formatted or proofread. Cochrane accordingly gives no representations or warranties of any kind in relation to, and accepts no liability for any reliance on or use of, such material.

Back to top

# Serious adverse events data not contributing to meta-analyses

|  |  |  |  |  |  |
| --- | --- | --- | --- | --- | --- |
| **Study ID** | **Study design** | **Intervention/ comparator** | **Time point** | **Data (SAEs)** | **Between group difference (RCT) (↑**  **more SAEs in intervention arm; ↔ equivocal; ↓ fewer SAEs in intervention arm)**    **Direction over time (cohort) (↓ decline in frequency)** |
| Edwards 2023 | Cohort | Nicotine EC | 6 months | No serious adverse events or expeditable events occurred. |  |
| Bullen 2013 | RCT | Nicotine EC v non-nicotine EC v NRT | 6 months | None occurred which were considered related to study treatment. No further information available. |  |
| Caponnetto 2013b\* | Cohort | Nicotine EC | 1 year | None occurred |  |
| Caponnetto 2021\* | Cohort | Nicotine EC | 24 weeks | None occurred |  |
| Hickling 2019 | Cohort | Nicotine EC | 6 weeks | (Recruited from mental health settings) Five SAEs during study; all were psychiatric hospitalisations; all were considered unrelated to the study intervention |  |
| Higgins 2024 | RCT | nicotine EC + very low nicotine content cigarettes (VLNC) v VLNC | 16 weeks | One SAE related or probably related to study product in EC plus VLNC arm. None in VLNC alone arm. | ↑ |
| Humair 2014 | Cohort | Nicotine EC | Unclear (longest follow-up 1 year) | None reported |  |
| Dawkins 2020 | RCT | Nicotine EC v usual care | 24 weeks | Not formally assessed. A&E visits reported: “low but constant use of emergency and hospital services in both arms. Usual care (n= 32), 4-7 participants visited A & E at different time points. The number of visits ranged from one to nine times. In the EC arm (n =48), 5-7 participants visited A & E at different time points. The number of visits ranged from one to six times.”  “in the UC arm, at least five participants at baseline, two at 4 weeks, one at 12 weeks and one at 24 weeks were admitted following the A & E visit. In the EC arm, at least two participants at baseline, three at 4 weeks, and one at 12 weeks were admitted following the A & E visit.” | ↔ |
| Kanobe 2022\* | Cohort | Nicotine EC |  | None reported |  |
| Morris 2022\* | RCT | Nicotine EC, variations on nicotine strength, flavour, and salt vs. freebase | 9 days | None occurred |  |
| NCT02648178 | Cohort | Nicotine EC | NS | Reports 1 event (death) (n = 19). No further detail provided. |  |
| Polosa 2011\* | Cohort | Nicotine EC | 6 months | None occurred during the study |  |
| Sifat 2024 | RCT | Nicotine EC v nicotine EC + incentives | 8 weeks | None reported |  |
| Valentine 2018 | Cohort | Nicotine EC | 8 weeks | “No serious adverse events were reported” |  |
