# Supplementary material for: Electronic cigarettes for smoking cessation
Source: Cochrane Database Syst Rev. 2025 Nov 10;2025(11):CD010216. doi: 10.1002/14651858.CD010216.pub10 (PMC12599494; doi:10.1002/14651858.CD010216.pub10)
Supplement: Supplementary file 13 — Supplementary material 13 Carbon monoxide data not contributing to meta‐analyses [file CD010216-SUP-13-other.html]

Carbon monoxide data not contributing to meta-analyses


# Supplementary material 13 to: Electronic cigarettes for smoking cessation

Lindson N, Livingstone-Banks J, Butler AR, McRobbie H, Bullen CR, Hajek P, Wu AD, Begh R, Theodoulou A, Notley C, Rigotti NA, Turner T, Fanshawe T, Hartmann-Boyce J
  
https://doi.org/10.1002/14651858.CD010216.pub10

The material in this section has been supplied by the author(s) for publication under a Licence for Publication and the author(s) are solely responsible for the material. Cochrane has reviewed this material, but Cochrane has not copyedited, formatted or proofread. Cochrane accordingly gives no representations or warranties of any kind in relation to, and accepts no liability for any reliance on or use of, such material.

Back to top

# Carbon monoxide data not contributing to meta-analyses

Randomized and crossover trials, nicotine EC v comparator group [1]

|  |  |  |  |  |
| --- | --- | --- | --- | --- |
| **Study ID** | **Intervention/ comparator** | **Time point** | **Data** | **Between-group difference**[2] **(↑ higher in EC/higher dose EC arm; ↔ equivocal; ↓ lower in EC/higher dose arm)** |
| Czoli 2019 | Nicotine EC v traditional cigarette | 1 week | Compared to baseline (dual use):  EC -41%  Traditional cigarette +21% | ↓ |
| George 2019 | Nicotine EC v non-nicotine EC v traditional cigarette | 4 weeks | eCO measured but not reported in detail. Lowest tertile of CO at end of study was in those with “best compliance with EC and least dual use” | NE |
| Katz 2025 | Nicotine EC v combustible cigarettes | 2 weeks |  |  |
| Meier 2017 | Nicotine EC v non-nicotine EC | 1 week | Randomized crossover trial. eCO: Naturalistic (whilst smoking, no EC) mean 11.4 (SE 1.3), Nicotine EC 11.6 (SE 1.3), Non-nicotine EC 10.2 (SE 1.3) | ↑ |
| NCT03113136 | Low Wattage EC (LWe) v High Wattage EC v combustible cigarettes | 4 and 12 weeks | “At 4 and 12 weeks, the CO levels were reduced in dual users of combustible tobacco and e-cigarettes (LWe or HWe), compared to exclusive combustible tobacco users.” | ↓ |
| Tseng 2016 | Nicotine EC v non-nicotine EC | 1 and 3 weeks | No between-group difference in eCO was observed at any time point, no further data available | ↔ |
| Walele 2018\* | Nicotine EC versus conventional cigarette | 2 weeks | “At baseline, eCO was at 20.3 ppm (±8.4) in EVP subjects and at 21.3 ppm (±9.2) in CC subjects. In the EVP group, eCO levels dropped to 7.4 ppm at Week 1 and were between 7.6 and 9.0 ppm from Week 2 to EoS (eCO levels in non-smokers are typically ≤6 ppm (Bedfront, 2016)). In the CC group, eCO remained at levels close to baseline during the whole study (21.3–23.3 ppm).” | ↓ |
| Walker 2020 | Nicotine EC + NRT v non-nicotine EC + NRT v NRT only | 6 months | 20% nicotine EC arm; 10% non-nicotine EC arm; 4% NRT only arm had eCO < 8ppm at follow-up | ↓ |
| Yingst 2020 | Nicotine EC (36 mg/ml; refillable) versus nicotine EC (24 mg/ml; cig-a-like) | 1 week | N = 17  “CO significantly decreased from baseline during the button-operated ENDS use period from 24.1 ppm (SD = 12.2) to 18.4 ppm (SD = 13.8) (p = .03), but did not significantly decrease during the cigalike use period (22.4 ppm (SD = 12.8) vs. 18.1 ppm (SD = 13.8) (p = .07)). Total CO reduction was not different between ENDS devices (p= .75).” | ↔ |

##

## Studies in which all groups received nicotine EC with no between-group difference in concentration

|  |  |  |  |
| --- | --- | --- | --- |
| **Study ID** | **Time point** | **Data** | **Direction over time**[3] **(↓ decline; ↔ equivocal; ↑ increase)** |
| Caponnetto 2013b\* | 1 year | eCO reported graphically, “smoking reduction with “Categoria” e-Cigarette use was associated to a substantial decrease in the level of eCO”. In figure, reductions from baseline in all groups, but particularly pronounced in people who quit smoking or reduced cigarette consumption by >50% | ↓ |
| Caponnetto 2021\* | 24 weeks | In the 37 completers, mean change in CO from baseline -24.74 ppm (from 33.23 (SD 10.97) at baseline to 9.29 (SD 8.61) at follow-up) | ↓ |
| Coffey 2020 | 4 weeks | Baseline (n=1022): mean eCO 13.94; 4 week follow-up (n=567): mean eCO 5.05. “Of those who provided a reading on both occasions (n = 567), mean CO dropped significantly from 13.9 to 5.05 ppm (paired t = 28.3, p < .001).” | ↓ |
| Goniewicz 2017 | 2 weeks | Report significant decline in eCO from baseline (P < 0.05) at 1 and 2 weeks | ↓ |
| Hickling 2019 | 6 weeks[4] | Reduction from mean eCO (ppm) 25.67 (SD 17.16, n = 45) at baseline to 22.09 (SD 15.93, n = 45) at 6 weeks. At 24 weeks, no change (mean 26, SD 16.78, n = 31) | ↓ 6 weeks  ↔ 24 weeks |
| Hoeppner 2024 | 3 months | Baseline: 23.8, 3 months: 28.9, change from baseline: +5.1 | ↑ |
| Ikonomidis 2018[5] | 1 month | Reductions in eCO in people using EC + conventional cigarettes (baseline mean 15 ppm, SD 0.6, n = 24; 1 month mean 12.5, SD 0.6, n = 24); people using EC only (baseline mean 13.9, SD 0.7, n = 42; 1 month mean 4.2, SD 0.6, n = 42); “noncompliant” group (baseline mean 18.5, SD 0.7, n = 4, 1 month mean 15.1, SD 0.6, n = 4). (Slight increase in controls (given no intervention): baseline mean 15.3, SD 1, n = 20; 1 month mean 16.4, SD 0.7, n = 20.) | ↓ |
| Martner 2019 | 4 weeks | Reported for individual participants (n = 12), no averages given. “Following baseline, five participants showed immediate reductions in CO and high levels of EC use. Three showed more gradual reductions in CO and gradual increases in EC use. Two participants showed initial, but brief, reductions in CO and high levels of EC use, and two failed to appreciably reduce CO levels and showed relatively low EC use.” (data for other participants lost or withdrawn) | NE |
| McRobbie 2015 | 4 weeks | Mean change from baseline in eCO (ppm): -12 (95% CI -16 to -8), n = 33. Point estimate for reduction same in abstinent and smoking participants at 4 weeks | ↓ |
| Pacifici 2015 | 1, 4, 8 months | At one month, EC users showed a significant decline in eCO; there was no significant change in non-EC users (people who had opted not to use the EC provided). At four and eight months, exhaled CO had declined in EC and non-EC users. | ↓ |
| Pericot-Valverde 2025 | 8 weeks | Baseline: 21.5, 8 weeks: 14.8, change from baseline: -7.5 | ↓ |
| Polosa 2011\* | 2 years | Significant reduction in the average eCO across the whole cohort of 23.5 to 8 ppm at 24 months (P = 0.011) | ↓ |
| Polosa 2014b\* | 6 months | Measured eCO, report results graphically by group; at 24 weeks, CO appears to have significantly reduced amongst quitters and people reducing cigarette consumption by at least 50%, and appears to have remained stable in people who continued smoking at least half as many cigarettes as they had at baseline | ↓ quitters and people reducing cigarette consumption  ↔ people who continued smoking at least half as many cigarettes as they had at baseline |
| Pratt 2016 | 4 weeks | Baseline mean eCO 27.27 ppm (n = 19, no SD provided). At week 4, mean 15.21 (n = 19) | ↓ |
| Pulvers 2018 | 4 weeks | Significant change in eCO (P < 0.001). Baseline mean 14.3 ppm, SD 12.7, n= 40. 4 weeks: mean 8.9, SD 8.35, n = 40. | ↓ |
| Scheibein 2020 | 4, 8, 12 weeks | Week 1 (baseline): 21.89 (14.41) ppm, n = 23  Week 4: 13.78 (8.76) ppm, n = 17  Week 8: 15.78 (7.41) ppm, n = 12  Week 12: 15.56 (10.29) ppm, n = 9 | ↓ |
| Sifat 2024 | 8 weeks | EC + incentives arm: baseline: 14.57, 8 weeks: 8.69, change from baseline: -7.23  EC arm: baseline: 14.07, 8 weeks: 8.86, change from baseline: -4.57 | ↓ |
| Smith 2020 | 1 week | Study arms varied by PG/VG ratio. Reduced eCO in all groups. Combined mean: baseline 33.1 (SD 18.3), n = 30; 1 week 26.5 (SD 16.1), n = 28) | ↓ |
| Strasser 2016 | 10 days | Randomized to 4 different brands of EC (similar nicotine content). eCO reductions in all arms. Blu: Baseline mean 19.2 (SD 9.8, n = 6), day 10 mean 3.2 (2.2, n = 5)). Green smoke: Baseline 21.6 (8.8, n = 6), day 10 mean 4.0 (3.4, n = 5). V2: baseline mean 22.0 (8.1, n = 6), day 10 mean 4.8 (3.5, n = 5). White cloud: baseline mean 15.5 (3.5, n = 6); day 10 mean 1.5 (0.7, n = 2) | ↓ |
| Valentine 2018 | 8 weeks | Data provided in figures only, estimated baseline mean eCO (ppm) 9.3, SD 7.1, n = 50. 8 weeks: 8.5, 95% CI 7.1 to 9.9, n = 31 | ↓ |
| Van Staden 2013\* | 2 weeks | Smokers who switched to EC had significant reduction in arterial (1.95%, 95% CI 0.47 to 3.44; P = 0.01) and venous (1.87%, 95% CI 0.38 to 3.36; P = 0.02) carboxyhaemoglobin levels. | ↓ |
| Walele 2018\*[6] | 2 years | “The mean (±SD) eCO level in all subjects at Month 1 was 8.7 ppm (±6.5), and steadily decreased to reach 4.1 ppm (±3.1) at Month 24.” | ↓ |

[1] eCO: exhaled carbon monoxide; NS: not specified

[2] NE: not estimable

[3] NE: not estimable

[4] EC provided for 6 weeks; eCO measured at weeks 1 - 10 and 24

[5] Acute cross-over trial followed by ‘chronic phase’ so treated as cohort for purposes of this review

[6] Short term RCT (see first table); all participants then given nicotine EC hence inclusion in this table, as well
