# Supplementary material for: Electronic cigarettes for smoking cessation
Source: Cochrane Database Syst Rev. 2025 Nov 10;2025(11):CD010216. doi: 10.1002/14651858.CD010216.pub10 (PMC12599494; doi:10.1002/14651858.CD010216.pub10)
Supplement: Supplementary file 14 — Supplementary material 14 Heart rate data not contributing to meta‐analyses [file CD010216-SUP-14-other.html]

Heart rate data not contributing to meta-analyses


# Supplementary material 14 to: Electronic cigarettes for smoking cessation

Lindson N, Livingstone-Banks J, Butler AR, McRobbie H, Bullen CR, Hajek P, Wu AD, Begh R, Theodoulou A, Notley C, Rigotti NA, Turner T, Fanshawe T, Hartmann-Boyce J
  
https://doi.org/10.1002/14651858.CD010216.pub10

The material in this section has been supplied by the author(s) for publication under a Licence for Publication and the author(s) are solely responsible for the material. Cochrane has reviewed this material, but Cochrane has not copyedited, formatted or proofread. Cochrane accordingly gives no representations or warranties of any kind in relation to, and accepts no liability for any reliance on or use of, such material.

Back to top

# Heart rate data not contributing to meta-analyses

Randomized trials, nicotine EC v comparator group

|  |  |  |  |  |
| --- | --- | --- | --- | --- |
| **Study ID** | **Intervention/ comparator** | **Time point** | **Data** | **Between group difference**[1] **(↑ higher in EC/higher dose EC arm; ↔ equivocal; ↓ lower in EC/higher dose arm)** |
| Caponnetto 2023\* | Nicotine EC v heated tobacco | 12 weeks | “No significant changes in the mean resting heart rate, blood pressure, and BMI during product use were observed between and within study groups.” | ↔ |
| Cobb 2021 | EC (nicotine, non-nicotine groups combined) v QuitSmart cigarette substitute | 12 weeks | Substitute baseline mean: 79.6; mean change at 12 weeks: -1.89  EC baseline mean: 82.1; mean change at 12 weeks: -0.86 | ↑ |
| George 2019 | Nicotine EC v non-nicotine EC | 4 weeks | “No significant trend in difference among the three arms”  Broke down results by smoking characteristics at baseline. Smoked ≤ 20 pack years (n = 31): increased by 2.6 beats/min (95% CI: 0.3 to 5.0) for EC-nicotine; 5.2 beats/min (95% CI: 0.6 to 10.0) for nicotine-free. >20 pack-years (n = 82): decreased by 2.8 beats/min (95% CI: -5.2 to -0.4) for EC nicotine; decreased by 5.6 beats/min (95% CI: -10.4 to -0.8) for no-nicotine EC | ↔ |
| Walele 2018\* | Nicotine EC versus conventional cigarette | 2 weeks | “no clinically significant changes” | NE |

## Studies in which all groups received nicotine EC with no between-group difference in concentration

|  |  |  |  |
| --- | --- | --- | --- |
| **Study ID** | **Time point** | **Data** | **Direction over time**[2] **(↓ decline; ↔ equivocal; ↑ increase)** |
| Caponnetto 2021\* | 12 weeks | N = 40, reduction of 7.84 bpm (from 80.13 (SD 9.32) to 72.3 (SD 6.18)) | ↓ |
| Hickling 2019 | 6 weeks[3] | Mean: baseline 80.65 bpm (SD 16.01); week 6 82 bpm, SD 15.76 (n = 46) | ↑ |
| Ikonomidis 2018[4] | 1 month | E Cig+con-cig; baseline mean: 70; 1 month mean: 72 E-cig only; baseline mean: 73; 1 month mean: 70 Noncompliant; baseline mean: 71; 1 month mean: 70 Controls; baseline mean: 71; 1 month mean: 70 | Mixed across groups |
| Oncken 2015 | 2 weeks | “no significant changes” | ↔ |
| Van Staden 2013\* | 2 weeks | “no significant changes” | ↔ |
| Walele 2018\*[5] | 2 years | Baseline: 72.3 Month 1: 71.4 Month 6: 71.6 Month 12: 71.1 Month 18: 69.1 Month 24: 70.4 | ↓ |

[1] NE: not estimable

[2] NE: not estimable

[3] EC provided for 6 weeks; HR measured at weeks 1 to 10 and 24

[4] Acute crossover trial followed by ‘chronic phase’ so treated as cohort for purposes of this review

[5] Short term RCT (see first table); all participants then given nicotine EC hence inclusion in this table, as well
