# Supplementary material for: Electronic cigarettes for smoking cessation
Source: Cochrane Database Syst Rev. 2025 Nov 10;2025(11):CD010216. doi: 10.1002/14651858.CD010216.pub10 (PMC12599494; doi:10.1002/14651858.CD010216.pub10)
Supplement: Supplementary file 15 — Supplementary material 15 Blood pressure data not contributing to meta‐analyses [file CD010216-SUP-15-other.html]

Blood pressure data not contributing to meta-analyses


# Supplementary material 15 to: Electronic cigarettes for smoking cessation

Lindson N, Livingstone-Banks J, Butler AR, McRobbie H, Bullen CR, Hajek P, Wu AD, Begh R, Theodoulou A, Notley C, Rigotti NA, Turner T, Fanshawe T, Hartmann-Boyce J
  
https://doi.org/10.1002/14651858.CD010216.pub10

The material in this section has been supplied by the author(s) for publication under a Licence for Publication and the author(s) are solely responsible for the material. Cochrane has reviewed this material, but Cochrane has not copyedited, formatted or proofread. Cochrane accordingly gives no representations or warranties of any kind in relation to, and accepts no liability for any reliance on or use of, such material.

Back to top

# Blood pressure data not contributing to meta-analyses

## Randomized trials, nicotine EC v comparator group

|  |  |  |  |  |
| --- | --- | --- | --- | --- |
| **Study ID** | **Intervention/ comparator** | **Time point** | **Data** | **Between group difference**[1] **(↑ higher in EC/higher dose EC arm; ↔ equivocal; ↓ lower in EC/higher dose arm)** |
| Caponnetto 2023\* | Nicotine EC v heated tobacco | 12 weeks | “No significant changes in the mean resting heart rate, blood pressure, and BMI during product use were observed between and within study groups.” | ↔ |
| George 2019 | Nicotine EC v non-nicotine EC | 4 weeks | “When both EC groups were combined, there was a greater reduction in systolic blood pressure in the EC group than in the TC group, both in smokers of ≤20 pack-years (EC: -4.41 mm Hg; 95% CI: -7.91 to -0.91 vs. TC: -2.86 mm Hg; 95% CI: -8.09 to 2.38; p = 0.59) and >20 pack-years (EC: -7.75 mm Hg; 95% CI: -11.56 to -3.93 vs. TC: -1.37 mm Hg; 95% CI: -5.32 to 2.59; p = 0.04).” | NE |
| Katz 2025 | Nicotine EC versus conventional cigarette | 2 weeks | “Diastolic blood pressure, the only cardiac measure that significantly changed across phases (F(2,41) = 3.54, p = 0.038), was lower after the e-cigarette phase than after the cigarette phase (p = 0.03).” | ↓ |
| Veldheer 2019 | EC (nicotine, non-nicotine groups combined) v QuitSmart cigarette substitute | 12 weeks |  | ↓ |
| Walele 2018\* | Nicotine EC versus conventional cigarette | 2 weeks | “No clinically significant changes” | NE |

## Studies in which all groups received nicotine EC with no between-group difference in concentration

|  |  |  |  |
| --- | --- | --- | --- |
| **Study ID** | **Time point** | **Data** | **Direction over time**[2] **(↓ decline; ↔ equivocal; ↑ increase)** |
| Caponnetto 2021\* | 24 weeks | Reduction in mean SBP of 12.07 (from 133.5 (SD 9.9) at baseline to 121.4 (SD 8.6) at follow up). N = 40 | ↓ |
| Hickling 2019 | 6 weeks[3] | Mean: baseline SBP 120 (SD 15.25); week 6 121 (SD 16.12). n=46 | ↑ |
| Ikonomidis 2018[4] | 1 month | E Cig+con-cig; basline mean: 124.91; month mean: 123.1 E-cig only; basline mean: 124.1; month mean: 122.2 Noncompliant; basline mean: 135.8; month mean: 134.6 Controls; basline mean: 133.6; month mean: 133.9 | Mixed across groups |
| Oncken 2015 | 2 weeks | “No significant changes” | ↔ |
| Van Staden 2013\* | 2 weeks | “no significant changes” | ↔ |
| Walele 2018\*[5] | 2 years | Baseline mean: 121.5 Month 1 mean: 120.7 Month 6 mean: 120.5 Month 12 mean: 123.2 Month 18 mean: 122.5 Month 24 mean: 122.4 | ↑ |

[1] NE: not estimable

[2] NE: not estimable

[3] EC provided for 6 weeks; BP measured at weeks 1-10 and 24

[4] Acute crossover trial followed by ‘chronic phase’ so treated as cohort for purposes of this review

[5] Short term RCT (see first table); all participants then given nicotine EC hence inclusion in this table, as well
