# Supplementary material for: Electronic cigarettes for smoking cessation
Source: Cochrane Database Syst Rev. 2025 Nov 10;2025(11):CD010216. doi: 10.1002/14651858.CD010216.pub10 (PMC12599494; doi:10.1002/14651858.CD010216.pub10)
Supplement: Supplementary file 16 — Supplementary material 16 Data on known toxicants/carcinogens from studies not contributing to meta‐analyses [file CD010216-SUP-16-other.html]

Data on known toxicants/carcinogens from studies not contributing to meta-analyses


# Supplementary material 16 to: Electronic cigarettes for smoking cessation

Lindson N, Livingstone-Banks J, Butler AR, McRobbie H, Bullen CR, Hajek P, Wu AD, Begh R, Theodoulou A, Notley C, Rigotti NA, Turner T, Fanshawe T, Hartmann-Boyce J
  
https://doi.org/10.1002/14651858.CD010216.pub10

The material in this section has been supplied by the author(s) for publication under a Licence for Publication and the author(s) are solely responsible for the material. Cochrane has reviewed this material, but Cochrane has not copyedited, formatted or proofread. Cochrane accordingly gives no representations or warranties of any kind in relation to, and accepts no liability for any reliance on or use of, such material.

Back to top

# Data on known toxicants/carcinogens from studies not contributing to meta-analyses

|  |  |  |  |
| --- | --- | --- | --- |
| **Study ID** | **Time point** | **Data** | **Direction over time**[2] **(↓ decline; ↔ equivocal; ↑ increase)** |
| Czoli 2019 | 1 week | Compared to dual use (baseline):  1-HOP EC -31%; tobacco cigarette +23%  NNAL EC -30%; tobacco cigarette +8% | ↓ 1-HOP  ↓ NNAL |
| Goniewicz 2017 | 2 weeks | 1-Hydroxyfluorene, baseline mean: 1414; week 2 mean: 592 1-Hydroxyphenanthrene, baseline mean: 488; week 2 mean: 584 1-Hydroxypyrene, baseline mean: 778; week 2 mean: 746 2-HPMA, baseline mean: 45; week 2 mean: 21 2-Hydroxyfluorene, baseline mean: 1029; week 2 mean: 842 2-Hydroxyphenanthrene, baseline mean: 655; week 2 mean: 968 2-Naphthol, baseline mean: 24; week 2 mean: 19 3-, 4-Hydroxyphenanthrenes, baseline mean: 1314; week 2 mean: 1410 3-HPMA, baseline mean: 937; week 2 mean: 410 3-Hydroxyfluorene, baseline mean: 679; week 2 mean: 451 AAMA, baseline mean: 254; week 2 mean: 110 CNEMA, baseline mean: 212; week 2 mean: 45 HEMA, baseline mean: 3821; week 2 mean: 1480 HPMMA, baseline mean: 1857; week 2 mean: 616 MHBMA, baseline mean: 1912; week 2 mean: 305 SPMA, baseline mean: 792; week 2 mean: 188 | ↓ 1-Hydroxyfluorene  ↑ 1-Hydroxyphenanthrene  ↓ 1-Hydroxypyrene  ↓ 2-HPMA  ↓ 2-Hydroxyfluorene  ↑ 2-Hydroxyphenanthrene  ↓ 2-Naphthol  ↑ 3-, 4-Hydroxyphenanthrenes  ↓ 3-HPMA  ↓ 3-Hydroxyfluorene  ↓ AAMA  ↓ CNEMA  ↓ HEMA  ↓ HPMMA  ↓ MHBMA  ↓ SPMA |
| Hickling 2019 | 6 weeks | 3HPMA (ng/ml), baseline mean: 2438.25; week 6 mean: 1180.75 Formic Acid, baseline mean: 6.6375; week 6 mean: 7.775 | ↓ 3-HPMA  ↑ Formic acid |
| Ikonomidis 2020b | 1 month | “…reduction of MDA (1.22 ± 0.1 vs 1.09 ± 0.1 μmol/L, P = 0.03)” in EC group. No change reported in cig group | ↓MDA |
| McRobbie 2015 | 4 weeks | Of the 33 people that completed four-week follow-up, 16 were EC users only, and 17 were dual users. Both groups showed a significant decrease in 3-HMPA in ng/mg creatinine (EC users: 1623 (SD 850) to 343 (SD 178), P < 0.001; Dual users: 2443 (SD 1105) to 969 (SD 807), P < 0.001). | ↓3-HMPA |
| Morris 2022\* | 9 days | “All BoE, except for nicotine equivalents, demonstrated statistically significant reductions from baseline levels (ranging from 72 to 97%).” No information provided on differences between study conditions. | ↓NNAL  ↓ 3-HPMA  ↓ S-PMA  ↓ CEMA  ↓ HEMA  ↓ 3-HMPMA  ↓ MHBMA  ↓ o-tol  ↓ 1-AN  ↓ 2-AN  ↓ NNN  ↓ 1-OHP  ↓ 3-OH B[a]P |
| Pratt 2022 | 8 weeks | Nicotine EC v no intervention comparison. “NNAL decreased more over time in the e-cigarette group. Closer examination of the longitudinal differences in NNAL revealed that the e-cigarette group had significantly lower NNAL at 4 weeks (estimate=0.54; se=0.23; t=2.37;p<.02), but the group difference was attenuated at 8 weeks (estimate=0.42; se=0.23; t=1.83; p<.07)” | ↓ NNAL |
| Pulvers 2018 | 4 weeks | “Statistically significant differences in log-transformed NNAL levels (mean difference = 0.35, 95% CI = 0.09–0.69, p < .01). Of the eight VOCs measured, there were two significant decreases in log-transformed values of the benzene metabolite, PMA (mean differences = 0.57, 95%CI = 0.12–1.03, p = .01), and the acrylonitrile metabolite, CNEMA (mean differences = 0.60, 95%CI = 0.25–0.96, p = .001).” See paper for crude data on all outcomes. | ↓ NNAL  ↓ PMA  ↓ HEMA  ↑ MMA  ↓ CNEMA  ↓ 3-HPMA  ↓ 2-HPMA  ↓ AAMA  ↓ HPMMA |

NS: not specified; all studies provided all participants with nicotine EC unless otherwise stated

[2] Where reported
