# Supplementary material for: Electronic cigarettes for smoking cessation
Source: Cochrane Database Syst Rev. 2025 Nov 10;2025(11):CD010216. doi: 10.1002/14651858.CD010216.pub10 (PMC12599494; doi:10.1002/14651858.CD010216.pub10)
Supplement: Supplementary file 17 — Supplementary material 17 Data on lung function from studies not contributing to meta‐analyses [file CD010216-SUP-17-other.html]

Data on lung function from studies not contributing to meta-analyses


# Supplementary material 17 to: Electronic cigarettes for smoking cessation

Lindson N, Livingstone-Banks J, Butler AR, McRobbie H, Bullen CR, Hajek P, Wu AD, Begh R, Theodoulou A, Notley C, Rigotti NA, Turner T, Fanshawe T, Hartmann-Boyce J
  
https://doi.org/10.1002/14651858.CD010216.pub10

The material in this section has been supplied by the author(s) for publication under a Licence for Publication and the author(s) are solely responsible for the material. Cochrane has reviewed this material, but Cochrane has not copyedited, formatted or proofread. Cochrane accordingly gives no representations or warranties of any kind in relation to, and accepts no liability for any reliance on or use of, such material.

Back to top

# Data on lung function from studies not contributing to meta-analyses

|  |  |  |  |  |
| --- | --- | --- | --- | --- |
| **Study ID** | **Comparison** | **Time point** | **Data** | **Direction over time (↓ decline; ↔ equivocal; ↑ increase)** |
| Cobb 2021 | EC (nicotine, non-nicotine groups combined) v QuitSmart cigarette substitute (sub) | 12 weeks | FEV1 in litres  Substitute, baseline mean: 2.48; mean change at 12 weeks: 0.01 EC, baseline mean: 2.7; mean change at 12 weeks: 0  FEV1% predicted  Substitute, baseline mean: 76.2; mean change at 12 weeks: 1.1 EC, baseline mean: 79.9; mean change at 12 weeks: -0.28  FVC litres  Substitute, baseline mean: 3.23; mean change at 12 weeks: -0.04 EC, baseline mean: 3.42; mean change at 12 weeks: 0.02  Forced expiratory flow at 25–75% of FVC  Substitute, baseline mean: 2.3; mean change at 12 weeks: 0.06 EC, baseline mean: 2.56; mean change at 12 weeks: -0.04  Forced expiratory time in seconds  Substitute, baseline mean: 5.09; mean change at 12 weeks: -0.26 EC, baseline mean: 4.59; mean change at 12 weeks: 0.27 | ↔ FEV1 in litres  ↓ FEV1% predicted  ↑ FVC litres  ↓ Forced expiratory flow at 25–75% of FVC  ↑ Forced expiratory time in seconds |
| Hickling 2019 | n/a – all participants provided nicotine EC (for 6 weeks) | 6, 10, 24 weeks | Mean peak flow  Baseline: 450.646 Week 6: 450.2039 Week 10: 452.4058 Week 24: 463.3345 | ↑ |
| Katz 2025 | Nicotine EC v combustible cigarettes | 2 weeks | “Changes in objective (spirometry, oscillometry) and self-reported (CAT, SGRQ-C) pulmonary measures were minor and did not meet thresholds for statistical significance.” |  |
| Oncken 2015 | n/a – all participants provided nicotine EC | 2 weeks | “no significant differences” in airway function (Raw or sGaw) over the course of the two weeks compared to baseline (P > 0.09), or five minutes after inhalation of either type of EC (P > 0.1). | ↔ |
| Smith 2025 | Nicotine EC v NRT | 4 weeks | “Respiratory Health Symptoms improved in both groups. Respiratory Health reduced from 12.2 (SD = 8) to 5.8 (SD = 6.8) in the e-cigarette group (a 52% reduction, n = 17), and from 15.7 (SD = 9.9) to 11.2 (SD = 10.7) in the NRT group (a 29% reduction, n = 10). This corresponds to an MD in the change of 1.8 favoring the e-cigarette group (95% CI: −5.7 to 9.5; Figure 2).” | ↓ Respiratory health symptoms |
