# Supplementary material for: Electronic cigarettes for smoking cessation
Source: Cochrane Database Syst Rev. 2025 Nov 10;2025(11):CD010216. doi: 10.1002/14651858.CD010216.pub10 (PMC12599494; doi:10.1002/14651858.CD010216.pub10)
Supplement: Supplementary file 18 — Supplementary material 18 Product use at 6+ months for data not contributing to meta‐analyses [file CD010216-SUP-18-other.html]

Product use at 6+ months for data not contributing to meta-analyses


# Supplementary material 18 to: Electronic cigarettes for smoking cessation

Lindson N, Livingstone-Banks J, Butler AR, McRobbie H, Bullen CR, Hajek P, Wu AD, Begh R, Theodoulou A, Notley C, Rigotti NA, Turner T, Fanshawe T, Hartmann-Boyce J
  
https://doi.org/10.1002/14651858.CD010216.pub10

The material in this section has been supplied by the author(s) for publication under a Licence for Publication and the author(s) are solely responsible for the material. Cochrane has reviewed this material, but Cochrane has not copyedited, formatted or proofread. Cochrane accordingly gives no representations or warranties of any kind in relation to, and accepts no liability for any reliance on or use of, such material.

Back to top

# Product use at 6+ months for data not contributing to meta-analyses

**RCTs**

|  |  |  |  |  |
| --- | --- | --- | --- | --- |
| **Study ID** | **Comparison** | **Time point (months)** | **Data** | **Between group difference (↑**  **Higher product use in intervention arm; ↔ equivocal; ↓ lower product use in intervention arm)** |
| Auer 2024 | EC v. behavioural support only | 6 months | At 6 months 368 of the 552 (67%) participants contacted in the intervention arm were using EC. | n/a |
| Begh 2021 | EC v. no treatment | 8 months | 35% (52/148) in EC arm using EC at 8 months. | n/a |
| Carpenter 2023 | EC v. no treatment | 6 months | At 6 months 44% of the EC group were still using an EC; however, it is unclear whether the denominator was number randomised (427) or number followed up (292), making it impossible to calculate absolute numbers. | n/a |
| Dawkins 2020 | EC v. behavioural support only | 6 months | 56% (27/48 (randomised)) or 77% (27/35 (FU)) in EC arm using EC at 6 months. | n/a |
| Holliday 2019 | EC v. no treatment | 6 months | 72% (21/29) in EC arm using EC at 6 months. | n/a |
| Kouroutzoglou 2024 | EC v. NRT | 6 months | “at 6 months among users of EC 6 continued vaping nicotine alone, 7 continued to smoke tobacco cigarettes while vaping and 4 continued smoking cigarettes alone. Nevertheless only 2 out of 19 participants in that group stayed abstinent from both combustible cigarette smoking and vaping.” Rates not reported for NRT. |  |
| Martinez 2021 | Advice to use nicotine EC to quit vs standard quitting advice | 6 | 64% in the targeted booklet arm, 66% in the generic booklet arm were still using EC at 6 months follow-up (participants were dual users at baseline) | ↔ |
| Pope 2024 | EC v. behavioural support only | 6 months | At 6 months 125 of 317 (39.4%) participants contacted in the EC arm were using EC | n/a |
| Pulvers 2020 | EC v. no treatment | 6 months | 44% 55/126 (randomised) or 57% (55/96 (at FU)) were still using EC (EC and dual use) at 6 months in the EC arm | n/a |
| Smith 2025 | EC v NRT | 4 weeks | EC arm Use 1 per week: 85%, use at least 4 days/week: 70%  NRT 1 x per week: 80% patch, 70% lozenge, 60% patch + lozenge  NRT at least 4 days/week: 60% patch, 40% lozenge, 40% (patch + lozenge) | ↑ |
| Walker 2020 | Nicotine EC + nicotine patch vs non-nicotine EC + nicotine patch | 6 | 36% (179/500 (randomised)) or 57% (179/317 (at FU)) were still using EC in nicotine EC + patch arm at 6 months  24% 152/624 (randomised) or 49% (152/308 at FU) were still using non nicotine EC in the non-nicotine + patch arm | ↑ |
| Xu 2023\* | EC v. behavioural support only | 12 months | At 12 months 174 of the 566 randomised reported using nicotine EC | n/a |

## Cohort studies (all nicotine EC)

|  |  |  |  |
| --- | --- | --- | --- |
| **Study ID** | **Time point (months)** | **Data** | **Frequency over time**[1] **(↓ decline; ↔ equivocal; ↑ increase)** |
| Edwards 2023 | 6 | 80% (24/30 (randomised)) or 92% (24/26 (at FU)) still using EC at 6 month follow-up. EC type: refillable. Country: Australia. | ↓ |
| Caponnetto 2013b\* | 12 | 64% (9/14 randomised and followed up) still using EC at 12 month follow-up. EC type: cig-a-like. Country: Italy. | ↓ |
| Ely 2013 | 6 | 42% (20/48 (randomised)) or 46% (20/44 (at FU)) still using EC at 6 month follow-up. EC type: cig-a-like. Country: USA. | ↓ |
| Pacifici 2015 | 8 | 77% (26/34 (randomised and followed up)) still using EC at 6 month follow-up. EC type: refillable. Country: Italy. | ↓ |
| Polosa 2011\* | 6 | 55% (22/40 (randomised)) or 82% (22/27(at FU)) still using EC at 6 month follow-up. EC type: cig-a-like. Country: Italy. | ↓ |
| Price 2022 | 12 | 5% of the 871 participants provided with an e-cigarette, charger, and fluid were still using an e-cigarette at 12 month follow-up. 56% (43/77 at FU) were still using EC. The 43 people were using EC with nicotine, EC without nicotine and dual EC CC use.) EC type: refillable. Country: England, UK | ↓ |
